# Metabolic surgery mitigates early kidney injury in obese youth with diabetes by suppressing mTORC1/JAK/STAT signaling

**DOI:** 10.1172/JCI198545

**Published:** 2026-02-03

**Authors:** Abhijit S. Naik, Fadhl M. Alakwaa, Viji Nair, Phillip J. McCown, Jennifer A. Schaub, Edgar A. Otto, Rajasree Menon, Francesca Annese, Ye Ji Choi, Hailey E. Hampson, Thomas H. Inge, John Hartman, Sean Eddy, Cathy Smith, Jeffrey B. Hodgin, Ken Inoki, Swayam Prakash Srivastava, Kareem Al-Fagih, Shota Yoshida, Jesse A. Goodrich, Melanie G. Cree, Phoom Narongkiatikhun, Long Yuan, Kalie L. Tommerdahl, Pottumarthi Prasad, Daniël H. van Raalte, Megan M. Kelsey, Justin R. Ryder, Tyler J. Dobbs, Patricia Ladd, Subramaniam Pennathur, Robert G. Nelson, Yusuke Okabayashi, Victor G. Puelles, Jenna Ferrence-Salo, Jeffrey A. Beamish, Frank C. Brosius, Kristen J. Nadeau, Laura Pyle, Matthias Kretzler, Petter Bjornstad

**Affiliations:** 1 Division of Nephrology, Department of Internal Medicine, and; 2 Department of Computational Medicine and Bioinformatics, University of Michigan, Ann Arbor, Michigan, USA.; 3 Universita degli Studi di Bari Aldo Moro, Bari, Puglia, Italy.; 4 Division of Metabolism, Endocrinology, and Nutrition, Department of Medicine, and; 5 University of Washington Medicine Diabetes Institute, University of Washington, Seattle, Washington, USA.; 6 Department of Surgery, Lurie Children’s Hospital, Chicago, Illinois, USA.; 7 Department of Pathology,; 8 Life Sciences Institute, and; 9 Department of Molecular & Integrative Physiology, University of Michigan, Ann Arbor, Michigan, USA.; 10 Department of Population and Public Health Sciences, Keck School of Medicine, University of Southern California, Los Angeles, California, USA.; 11 Division of Pediatric Endocrinology, Department of Pediatrics, University of Colorado Anschutz Medical Campus, Aurora, Colorado, USA.; 12 Division of Nephrology, Department of Internal Medicine, Faculty of Medicine, Chiang Mai University, Chiang Mai, Thailand.; 13 Department of Immunology, Johns Hopkins University School of Medicine, Baltimore, Maryland, USA.; 14 Division of Endocrinology, Department of Pediatrics, University of Washington, Seattle, Washington, USA.; 15 Seattle Children’s Research Institute, Seattle, Washington, USA.; 16 Department of Radiology, NorthShore University Health System, Evanston, Illinois, USA.; 17 Diabetes Center, Department of Internal Medicine, Amsterdam University Medical Center, Amsterdam, Netherlands.; 18 Division of Radiology, University of Colorado Anschutz Medical Campus, Aurora, Colorado, USA.; 19 Research Division, Joslin Diabetes Center, Boston, Massachusetts, USA.; 20 III. Department of Medicine, University Medical Center Hamburg-Eppendorf, Hamburg, Germany.; 21 Hamburg Center for Kidney Health (HCKH), University Medical Center Hamburg-Eppendorf, Hamburg, Germany.; 22 Division of Nephrology and Hypertension, Department of Internal Medicine, The Jikei University School of Medicine, Tokyo, Japan.; 23 Department of Clinical Medicine, Aarhus University, Aarhus, Denmark.; 24 Department of Pathology, Aarhus University Hospital, Aarhus, Denmark.; 25 Department of Medicine, University of Arizona, Tucson, Arizona, USA.; 26 Department of Physiology, University of Michigan, Ann Arbor, Michigan, USA.

**Keywords:** Metabolism, Nephrology, Chronic kidney disease, Diabetes, Obesity

## Abstract

**BACKGROUND:**

Youth with type 2 diabetes (T2D) and severe obesity face high risk of diabetic kidney disease, which metabolic bariatric surgery (MBS) can mitigate. This study explores structural and molecular changes in kidneys after vertical sleeve gastrectomy (VSG), a form of MBS.

**METHODS:**

We performed paired analyses, including metabolic profiling, kidney volume assessment, histological evaluation, and single-cell RNA sequencing (scRNA-seq), on kidney biopsies from 5 youth with T2D and obesity pre- and 12 months post-VSG in the IMPROVE-T2D (Impact of Metabolic surgery on Pancreatic, Renal and cardiOVascular hEalth in youth with T2D) cohort. Circulating proteomics with kidney transcriptomics were linked using data from an independent cohort of youth with obesity, with or without T2D, undergoing MBS in Teen-Longitudinal Assessment of Bariatric Surgery (Teen-LABS, *n* = 64).

**RESULTS:**

After VSG, participants lost weight and had improvements in insulin sensitivity and metabolic parameters. Kidney changes included reduced renal hyperfiltration, total kidney volume, mesangial matrix area, and microalbuminuria. scRNA-seq in proximal tubule (PT) and thick ascending limb cells indicated repression of glycolysis, gluconeogenesis, and tricarboxylic acid (TCA) cycle genes, with upregulation of AMP-activated protein kinase (AMPK) and forkhead box O3 (FOXO3). Decreased metabolic signaling aligned with reduced ribosomal phosphorylated S6K (pS6K), suggesting attenuated mTORC1 activity. Janus kinase/signal transducer and activator of transcription (JAK/STAT) pathway activation in PT was diminished, correlating with lower circulating ligands from Teen-LABS proteomic data.

**CONCLUSION:**

MBS/VSG prompts kidney molecular adaptations, providing potential targets for nonsurgical interventions against obesity- and diabetes-associated kidney disease.

**FUNDING:**

University of Washington with the American Diabetes Association, University of Michigan with Chan Zuckerberg Initiative, and Breakthrough T1D.

## Abstract 2

Structural and transcriptomic changes before and after vertical sleeve gastrectomy in kidneys of obese youth with diabetes revealed mechanisms for non-surgical interventions against kidney disease

## Graphical abstract

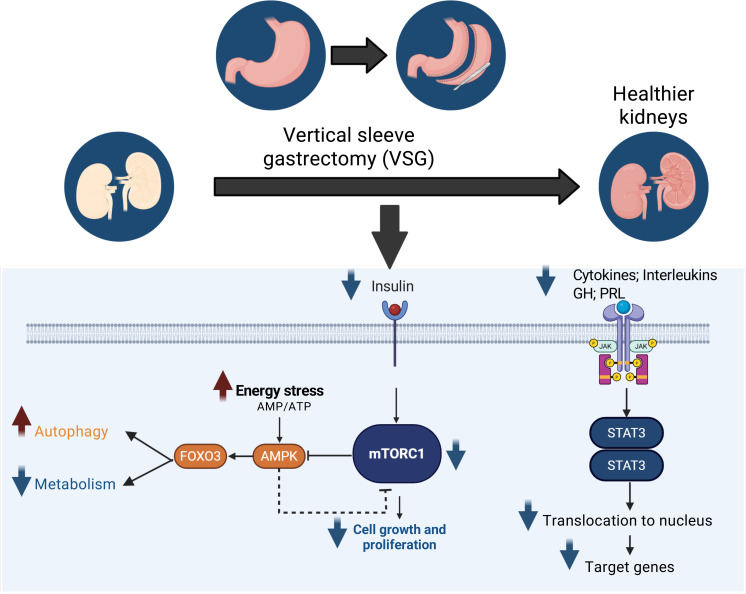

## Introduction

The increasing prevalence of obesity among youth has contributed to a dramatic rise in youth-onset type 2 diabetes (T2D) (1–4), a distinct and aggressive metabolic phenotype that includes severe insulin resistance and a rapid deterioration of β cell function (3). This phenotype is also associated with an exceptionally high risk for diabetic kidney disease (DKD) in early adulthood (4). Compared with adult-onset T2D, youth-onset T2D is characterized by more severe kidney structural lesions, a higher rate of kidney failure, and increased mortality, independent of diabetes duration and age (5, 6). However, the molecular mechanisms underlying disease severity in these youth remain poorly understood.

Metabolic bariatric surgery (MBS) is an important therapeutic option for some adolescents given limitations of lifestyle and pharmacological therapies in the care of youth with T2D (3, 7, 8). Significant improvements in weight, body composition, insulin sensitivity, β cell function, glycemic control, blood pressure, heart rate, dyslipidemia, glomerular hyperfiltration, and elevated urinary albumin excretion have been reported following Roux-en-Y gastric bypass (RYGB) or vertical sleeve gastrectomy (VSG) in youth with T2D (9–12). These effects may reduce the frequency and severity of diabetes complications, including DKD, in these youths.

**Figure 1:**
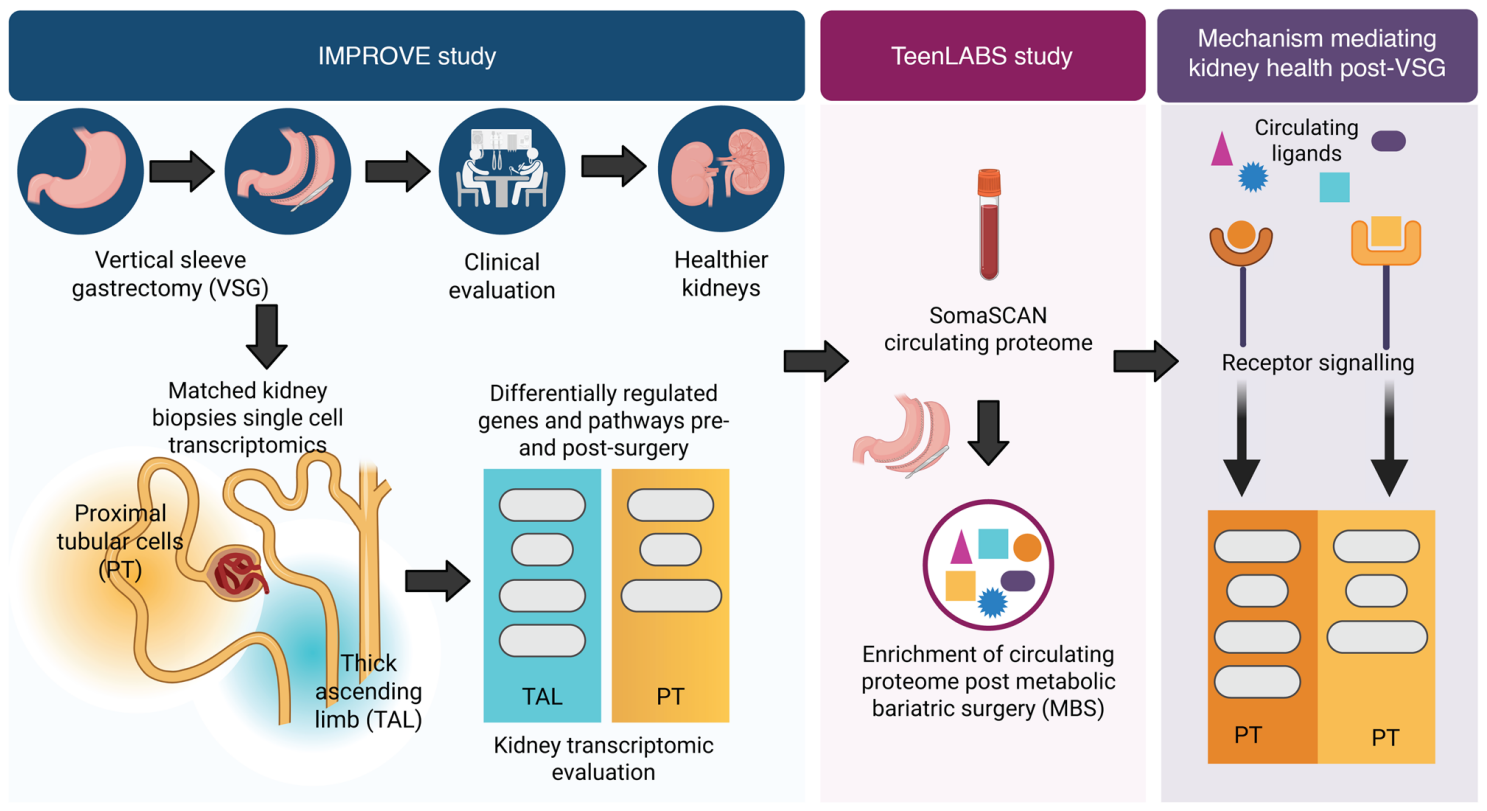
Overall study approach. A subset of participants in the IMPROVE-T2D cohort (*n* = 5) underwent research kidney biopsies before and 1 year after VSG. Transcriptomic data from these biopsies were used to identify molecular pathways associated with weight loss and improved metabolic and kidney function parameters post-VSG. The Teen-Longitudinal Assessment of Bariatric Surgery (Teen-LABS) cohort (*n* = 64) with data available on changes in serum proteins associated with MBS allowed the linkage of intrarenal molecular pathways to the circulating proteome. Created in BioRender. Subramanian, L. (2025) https://BioRender.com/s2l15o2

**Figure 2:**
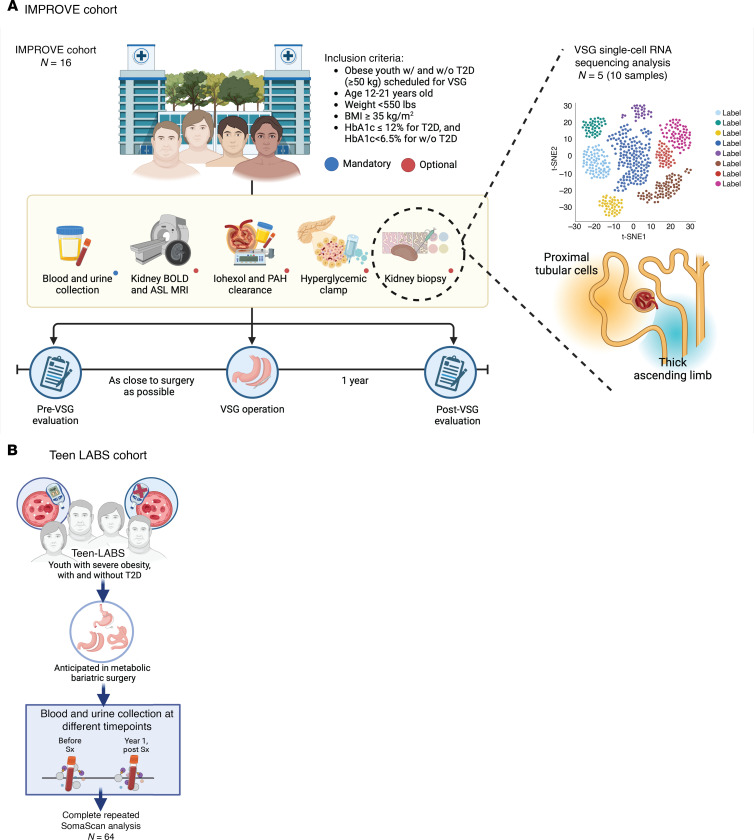
Cohort characteristics. (**A**) The IMPROVE-T2D and (**B**) Teen-LABS cohorts both included youth with T2D and obesity. Participants in the IMPROVE-T2D cohort underwent VSG, while in the Teen-LABS cohort participants underwent MBS, either VSG or RYGB. Five participants from IMPROVE-T2D contributed kidney biopsies, while Teen-LABS cohort participants were not biopsied. Created in BioRender. Narongkiatikhun, P. (2025) https:// BioRender.com/klppq6d (**A**) and https:// BioRender.com/097bo5r (**B**).

In this study (Figure 1), kidney tissue transcriptomic data from research kidney biopsies collected before and 12 months after VSG were combined with comprehensive clinical, morphological, and metabolic profiling data from participants in the Impact of Metabolic surgery on Pancreatic, Renal, and cardiOVascular hEalth in youth with Type 2 Diabetes (IMPROVE-T2D). Data were analyzed (Figure 2A) to characterize the kidney-specific effects of VSG and to identify molecular mechanisms associated with improved metabolic profile and the observed kidney structural and functional changes. Circulating proteomic data pre- and post-MBS from youth with obesity, both with and without T2D, from the Teen-Longitudinal Assessment of Bariatric Surgery (Teen-LABS, Figure 2B, n = 63) were found consistent with the tissue findings from IMPROVE-T2D (13). Collectively, these results identify the potent effect of MBS to reprogram kidney metabolism and facilitate resolution of inflammatory pathways. The mechanisms identified in this study provide leads for evaluating nonsurgical therapies that could effectively ameliorate or prevent early kidney injury in T2D.

## Results

Paired research kidney biopsies were obtained pre- and 12 months post-VSG from 5 youths with T2D in the IMPROVE-T2D cohort (Table 1). Given the small sample size, data distributions of continuous variables are presented as medians with their interquartile range (IQR; 25%–75%), and *P* values were not calculated. There was a 31% reduction in body mass index (BMI) (median 41.5 to 28.6 kg/m^2^) while the absolute glomerular filtration rate (GFR) by iohexol clearance decreased by 23.5% (228 to 175 mL/min), with a 12.2% reduction (171 to 150 mL/min/1.73 m^2^) when normalized for body surface area (BSA). Median urine albumin-to-creatinine ratio (UACR) decreased by 57%, while MRI-assessed total kidney volume (TKV) decreased by 13% (355.9 to 319.6 mL). These reductions in kidney volume were accompanied by reduction in glomerular nuclear count (20%), mesangial matrix area (12.8%), and mesangial index (8.4%). There were no reductions in glomerular and mesangial volume.

In parallel, circulating fasting plasma insulin levels decreased by 62.9% (IQR: 17.0 to 6.3 mg/dL), glycated hemoglobin (HbA1c) decreased by 24% (IQR: 6.0% to 5.4%), and fasting glucose levels dropped by 18.2% (IQR: 108 to 88.3 mg/dL). Insulin secretion improved substantially, with the acute insulin response to glucose (AIRg) increasing by 566% (IQR: 49.8 to 331.7 μU/mL × min) and the acute C-peptide response to glucose increasing by 350% (IQR: 0.8 to 3.6 ng/mL × min). Glucose disposal in response to endogenous hyperinsulinemia, as measured by the raw M value, increased by 224% (IQR: 2.1 to 6.8 mg/kg/min), and insulin sensitivity index (M/I) increased by 60% (IQR: 0.05 to 0.08 mg/kg/min per μU/mL).

Clinical characteristics and metabolic changes in these 5 pairs of biopsies were similar to the 11 IMPROVE-T2D participants who did not opt to provide research biopsies (Supplemental Table 1; supplemental material available online with this article; https://doi.org/10.1172/JCI198545DS1). Specific approaches to the evaluation of cellular pathways in kidney cells in response to VSG (Figure 1) and the cohorts (Figure 2) are schematically outlined and described more fully in Methods.

### scRNA-sequencing analysis and distribution of cell types across the nephron.

**Figure 3:**
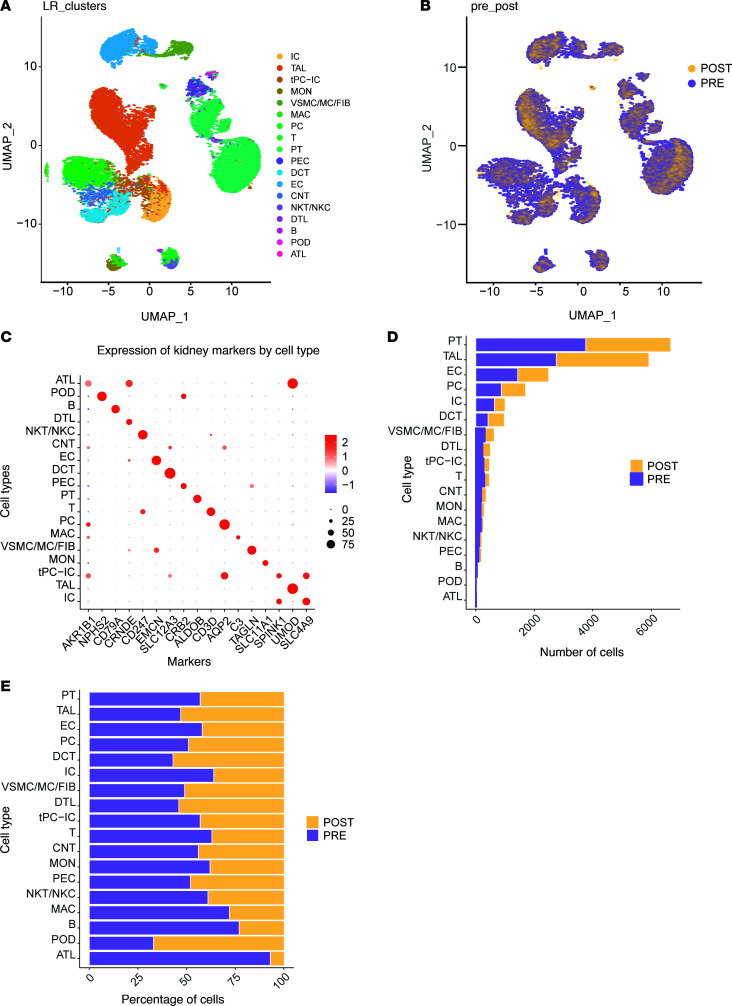
Single-cell data analysis. (**A**) Uniform manifold approximation and projection (UMAP) visualization of 23,598 cells pooled from the 5 pairs of participants in the IMPROVE-T2D cohort, organized into 18 distinct clusters. (**B**) The same UMAP, with color-coded cells based on whether they originated from participants before (purple) or after VSG (yellow). (**C**) Key cell type markers used for cell annotation. (**D**) The number and (**E**) percentage of each cell type before and after VSG. Cell type abbreviations: POD, podocyte; PEC, parietal epithelial cell; PT, proximal tubule; DTL, descending thin loop of Henle; ATL, ascending thin loop of Henle; TAL, thick ascending loop of Henle; DCT, distal convoluted tubule; CNT, connecting tubule; PC, principal cell; IC, intercalated cell; EC, endothelial cell; FIB, fibroblast; VSMC, vascular smooth muscle cell; MC, mesangial cell; B, B cell; T, T cell; NK T/C, natural killer T cell; tPC-IC, transient between PC and IC.

From the 5 pairs of biopsies, we isolated 23,598 high-quality cells that clustered into 18 distinct cell types (Figure 3A) with similar cell type distribution in pre- and post-VSG samples (Figure 3B). Cluster annotations were based on expression of key cell type markers (14–16) (Figure 3C). Glomerular cell types, like podocytes (18 cells), were inadequately represented for meaningful analysis. The absolute number (Figure 3D) and percentage (Figure 3E) of the different tubular epithelial cells, and EC that serve as nontubular internal controls, were similar pre- and post-VSG. Supplemental Tables 2 and 3 list the differentially expressed genes (DEGs) in PT and TAL cells, respectively.

All comparative analyses were between post- and pre-VSG samples (*n* = 23,598 cells). Healthy controls (HC; *n* = 19,735 cells, Supplemental Figure 1) were used as a reference to assess the direction of change of post-VSG samples.

### Transcriptional changes associated with VSG in PT and TAL.

**Figure 4:**
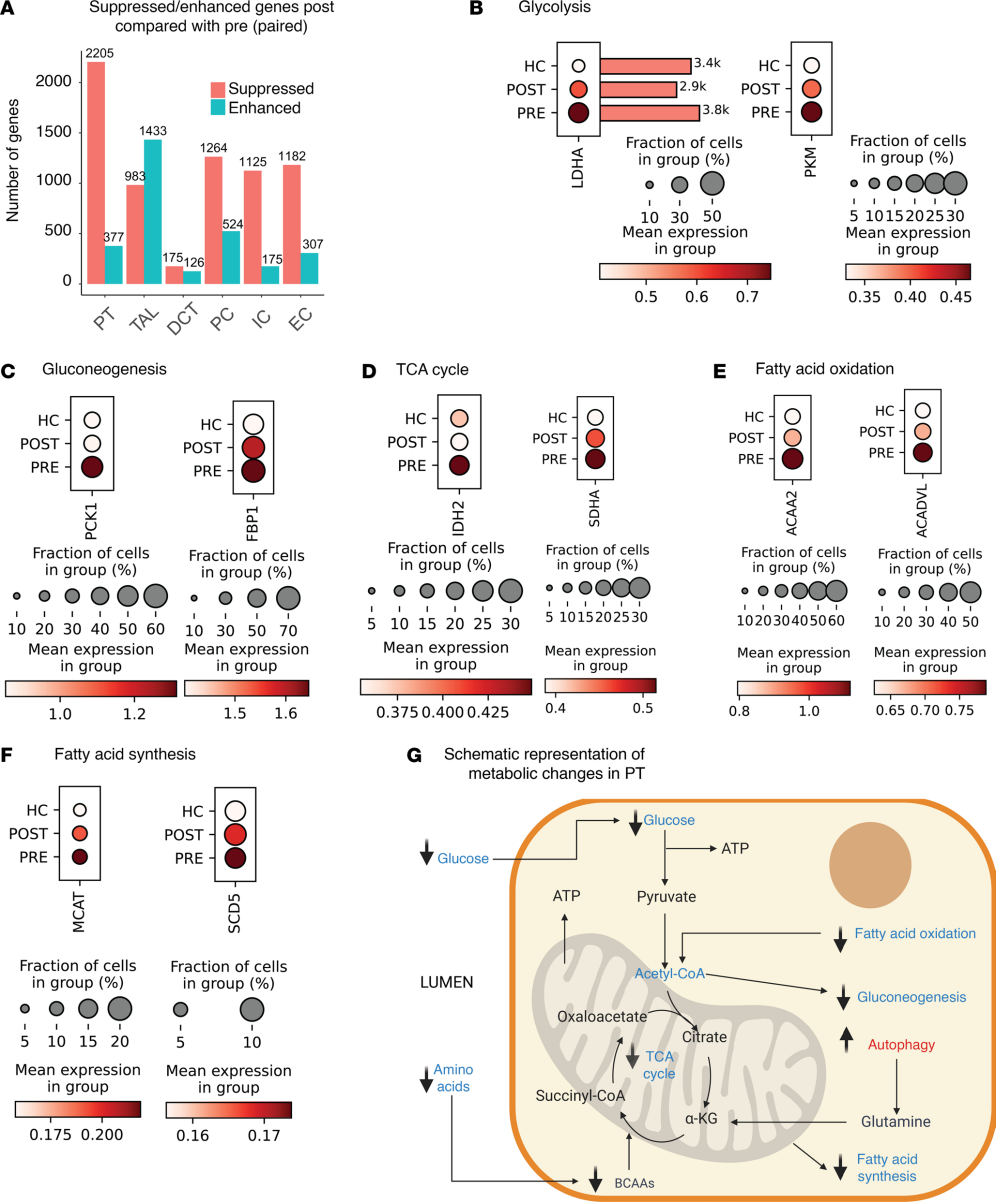
Transcriptomic changes in kidney tubular cells after VSG highlight metabolic reprogramming. (**A**) Bar plot of paired analysis comparing suppressed (pink) and enhanced (blue) genes post-VSG relative to pre-VSG by cell type. Dot plot comparison of expression levels between HC, pre-VSG, and post-VSG in PT cells of representative downregulated genes in metabolic pathways (**B**) glycolysis, (**C**) gluconeogenesis, (**D**) TCA cycle, (**E**) fatty acid oxidation, and (**F**) fatty acid synthesis. (**G**) A schematic summary of the adaptive transcriptional changes in PT cells post-VSG. BioRender. Subramanian, L. (2025) https://BioRender.com/rwlnvr4 *LDHA,* lactate dehydrogenase isozyme A; *PKM,* pyruvate kinase; *PCK1,* phosphoenolpyruvate carboxykinase 1; *FBP1,* fructose-1,6-bisphosphatase 1; *IDH2,* isocitrate dehydrogenase isozyme 22; *SDHA,* succinate dehydrogenase A; *ACAA2,* acetyl-CoA acyltransferase 2; *ACADVL,* very long-chain acyl-CoA dehydrogenase; *MCAT,* malonyl-CoA-acyl carrier protein transacylase; *SCD5,* stearoyl-CoA desaturase 5.

PT and TAL had the highest DEGs between pre- and post-VSG (Figure 4A). PT exhibited 2,582 DEGs comprising 2,205 enhanced and 377 suppressed genes post-VSG, while TAL had 2,416 DEGs, with 983 enhanced and 1,433 suppressed (Figure 4A), providing a strong rationale for further exploration of these 2 tubular cell types. Distal nephron segments, including PC, IC, and EC, also exhibited expression differences.

Pathway enrichment analysis of downregulated genes in PT using Reactome (Supplemental Figure 2A) identified reduction in several metabolism-related pathways, including TCA cycle, oxidative phosphorylation, glycolysis, and gluconeogenesis. There was also reduction in pathways related to protein translation and cellular responses to stress. To demonstrate this at the gene level, expression changes in representative genes for metabolism-related pathways were evaluated. There was reduced expression post-VSG of genes involved in glycolysis, such as lactate dehydrogenase isozyme A (*LDHA*, *P* < 0.0001) and pyruvate kinase (*PKM*, *P* < 0.0001) (Figure 4B); gluconeogenesis, such as phosphoenolpyruvate carboxykinase 1 (*PCK1*, *P* < 0.0001) and fructose-1,6-bisphosphatase 1 (*FBP1*, *P* = 0.01) (Figure 4C); TCA cycle, such as isocitrate dehydrogenase isozyme 2 (*IDH2*, *P* < 0.0001) and succinate dehydrogenase A (*SDHA*, *P* < 0.0001) (Figure 4D); and fatty acid oxidation, such as acetyl-CoA acyltransferase 2 (*ACAA2*, *P* < 0.0001) and very long-chain acyl-CoA dehydrogenase (*ACADVL*, *P* < 0.0001) (Figure 4E). There was decreased expression of mitochondrial malonyl-CoA–acyl carrier protein transacylase (*MCAT*, *P* = 0.008) and stearoyl-CoA desaturase 5 (*SCD5*, *P* < 0.0001), both involved in fatty acid synthesis (Figure 4F). Figure 4G schematically summarizes the observed transcriptional changes in PT, including the reduction in mechanistic target of rapamycin complex 1 (mTORC1) signaling after VSG (described later in detail). Increases in pathways related to developmental biology and signaling by receptor tyrosine kinases (RTKs) were identified among enhanced genes in PT (Supplemental Figure 2B).

Like PT, TAL cells showed downregulation of metabolism-associated transcripts after VSG. Pathway enrichment analysis of the 983 suppressed genes in TAL (Figure 4A) identified TCA cycle, respiratory electron transport, ATP synthesis by chemiosmotic coupling, and heat production by uncoupling proteins as the most enriched pathways while pathways related to protein translation were also downregulated (Supplemental Figure 3A).

**Figure 5:**
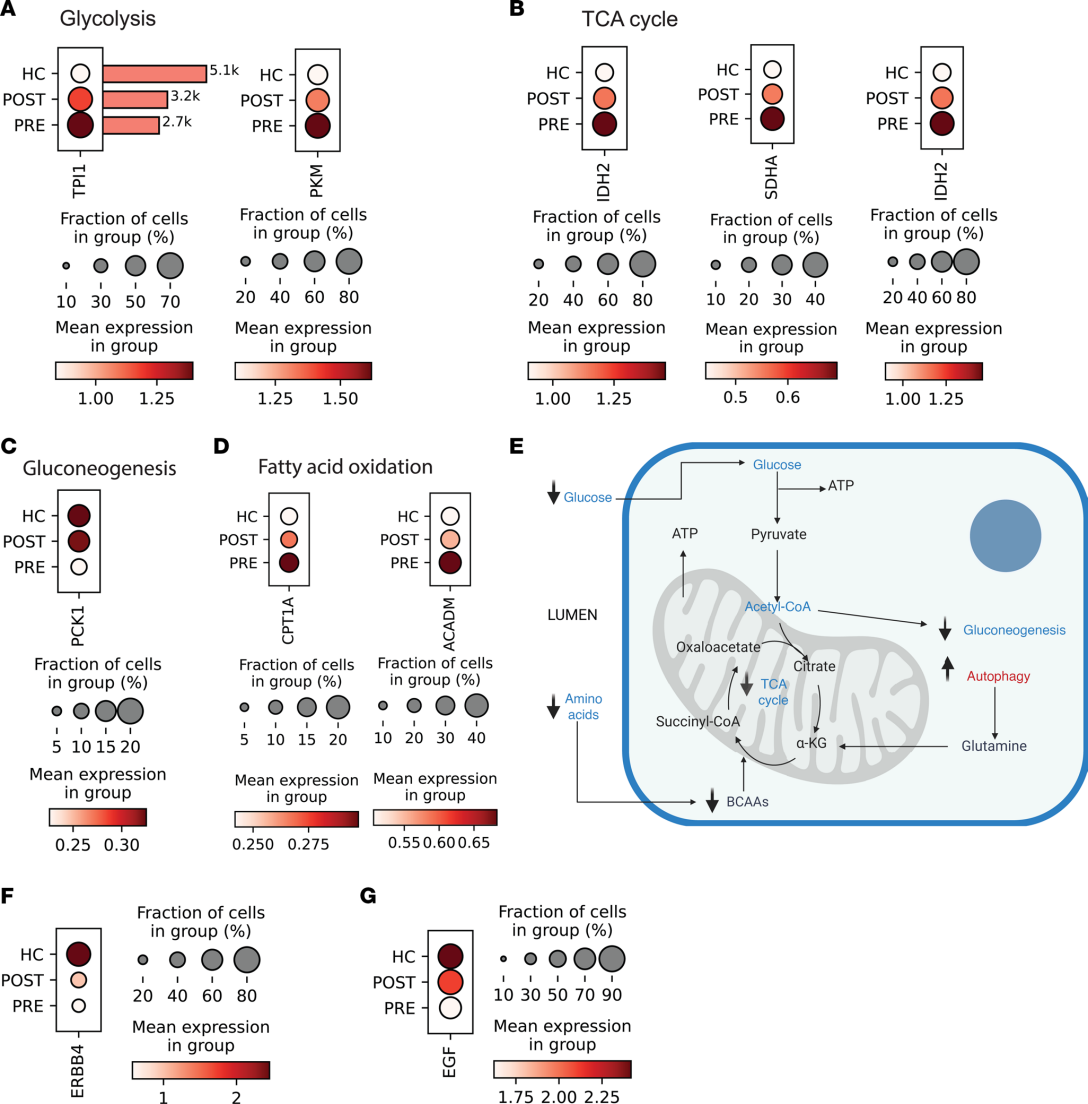
Transcriptional changes in TAL cells after VSG highlight metabolic suppression and signaling activation. Dot plot comparison of expression levels between HC, pre-VSG, and post-VSG in TAL cells of downregulated representative genes in metabolic pathways (**A**) glycolysis, (**B**) TCA cycle, (**C**) gluconeogenesis, and (**D**) fatty acid oxidation. (**E**) Schematic summarizing major downregulated metabolic pathways. BioRender. Naik, A. (2026) https://BioRender.com/nudhhxr Upregulated genes included (**F**) Erb-B2 receptor tyrosine kinase 4 (*ERBB4*) and (**G**) epidermal growth factor (*EGF*). *TPI1*, triosephosphate isomerase; *PKM,* pyruvate kinase; *PCK1,* phosphoenolpyruvate carboxykinase 1; *IDH2,* isocitrate dehydrogenase isozyme 22; *SDHA,* succinate dehydrogenase A; *CPT1A,* carnitine palmitoyltransferase 1; *ACADM,* medium-chain acyl-CoA dehydrogenase.

As in PT cells, representative genes of enriched metabolic pathways in TAL cells demonstrated reduced expression of genes associated with glycolysis (Figure 5A), including triosephosphate isomerase (*TPI1*, *P* ≤ 0.0001) and *PKM* (*P* < 0.0001). Additionally, genes associated with the TCA cycle, such as *IDH2*, *P* < 0.0001, and *SDHA*, *P* = 0.003 (Figure 5B); gluconeogenesis, such as *PCK1*, *P* = 0.002 (Figure 5C); and fatty acid oxidation, such as carnitine palmitoyltransferase 1 (*CPT1A*, *P* < 0.0001) and medium-chain acyl-CoA dehydrogenase (*ACADM*, *P* < 0.0001), were suppressed (Figure 5D). The schematic in Figure 5E summarizes the transcriptional changes in TAL post-VSG. Among the significantly upregulated genes in TAL cells were Erb-B2 receptor tyrosine kinase 4 (*ERBB4*; Figure 5F) and epidermal growth factor (*EGF*; Figure 5G), consistent with signaling that promotes tubular recovery. Like PT cells, signaling by RTKs and signal transduction were among the most enriched upregulated pathways (Supplemental Figure 3B).

### Evaluation of mTORC1 activity using downstream protein phosphorylation readout.

**Figure 6:**
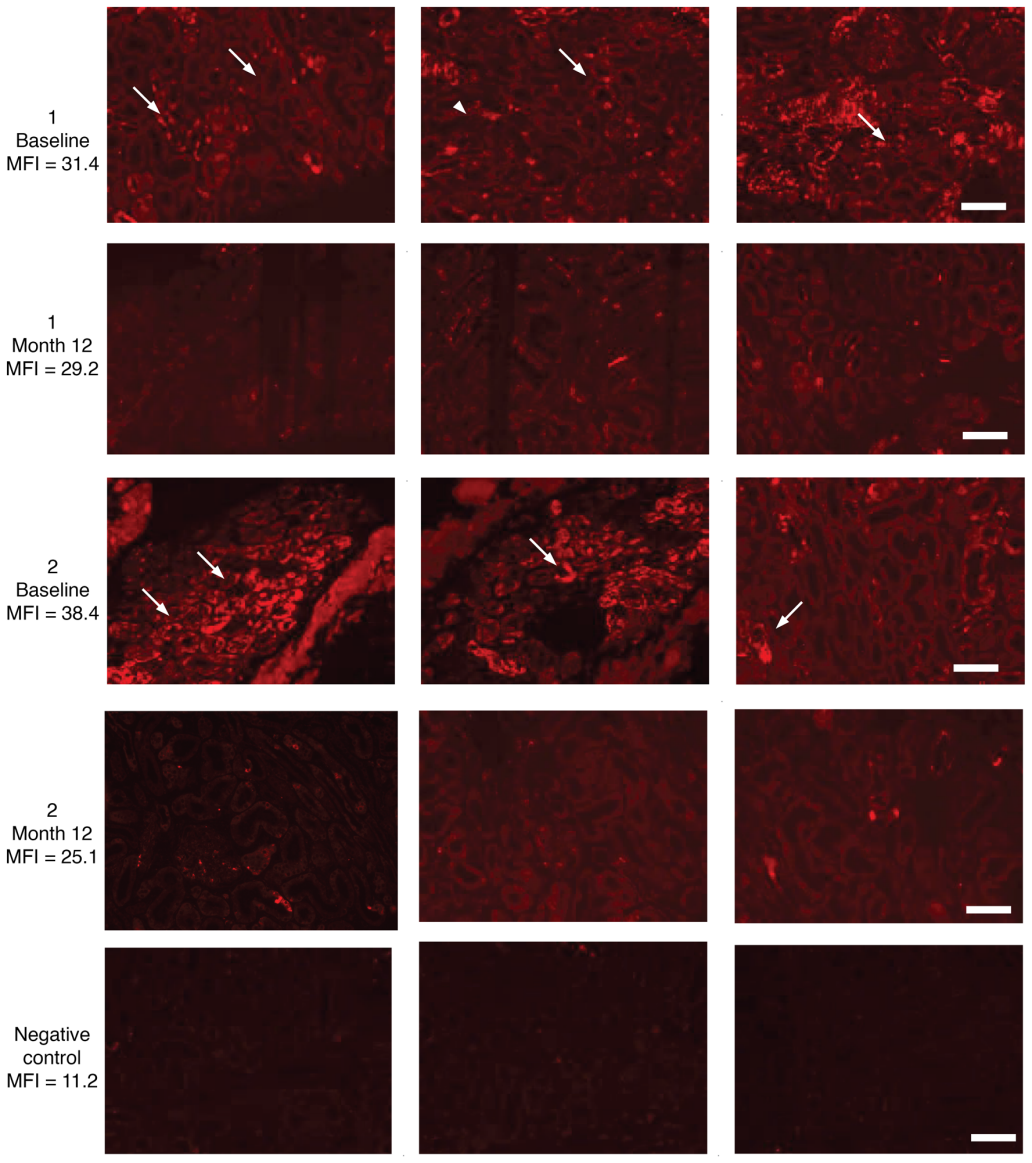
Reduction in rpS6 staining in baseline and 12-month kidney biopsies from youth with T2D. Immunofluorescence composite images depict rpS6 staining in baseline and 12-month kidney biopsies from youth with T2D enrolled in the IMPROVE-T2D study (*n* = 2 of 3 representative patients). Compared with baseline (arrows), immunofluorescence staining for rpS6 is substantially reduced post-VSG in kidney tubules. The kidney biopsy from participant 2 was not processed for RNA-Seq analysis, unlike participants 1 and 3, due to insufficient tissue availability; however, participant 2 had adequate paired tissue samples available for immunofluorescence studies. Scale bar = 100 μm. The mean fluorescence intensity (MFI) is the average fluorescence intensity across multiple stained sections for each sample.

Based on the transcriptomic changes and observed physiologic responses that strongly suggested reduction in mTORC1 signaling in PT and TAL post-VSG, we evaluated mTORC1 signaling activity in kidney tissue using immunofluorescence (IF) staining for a downstream substrate of the mTORC1-S6K1 pathway, ribosomal phosphorylated S6 (rpS6 S240/S244) (Figure 6, 2 representative patients). In the pre-VSG samples the average mean fluorescence intensity (MFI; arbitrary units) across a total of 14 sections in the 3 studied patients was 34.9. The immunofluorescent stains were predominantly localized to tubular epithelial cells and glomeruli, including podocytes, consistent with mTORC1 pathway activity. In contrast, 12 months post-VSG rpS6 IF intensity across 15 sections from the 3 patients was lower at 28.3 (*P* = 0.19) with reduced spatial distribution.

### Shared transcriptional response in PT and TAL after VSG.

**Figure 7:**
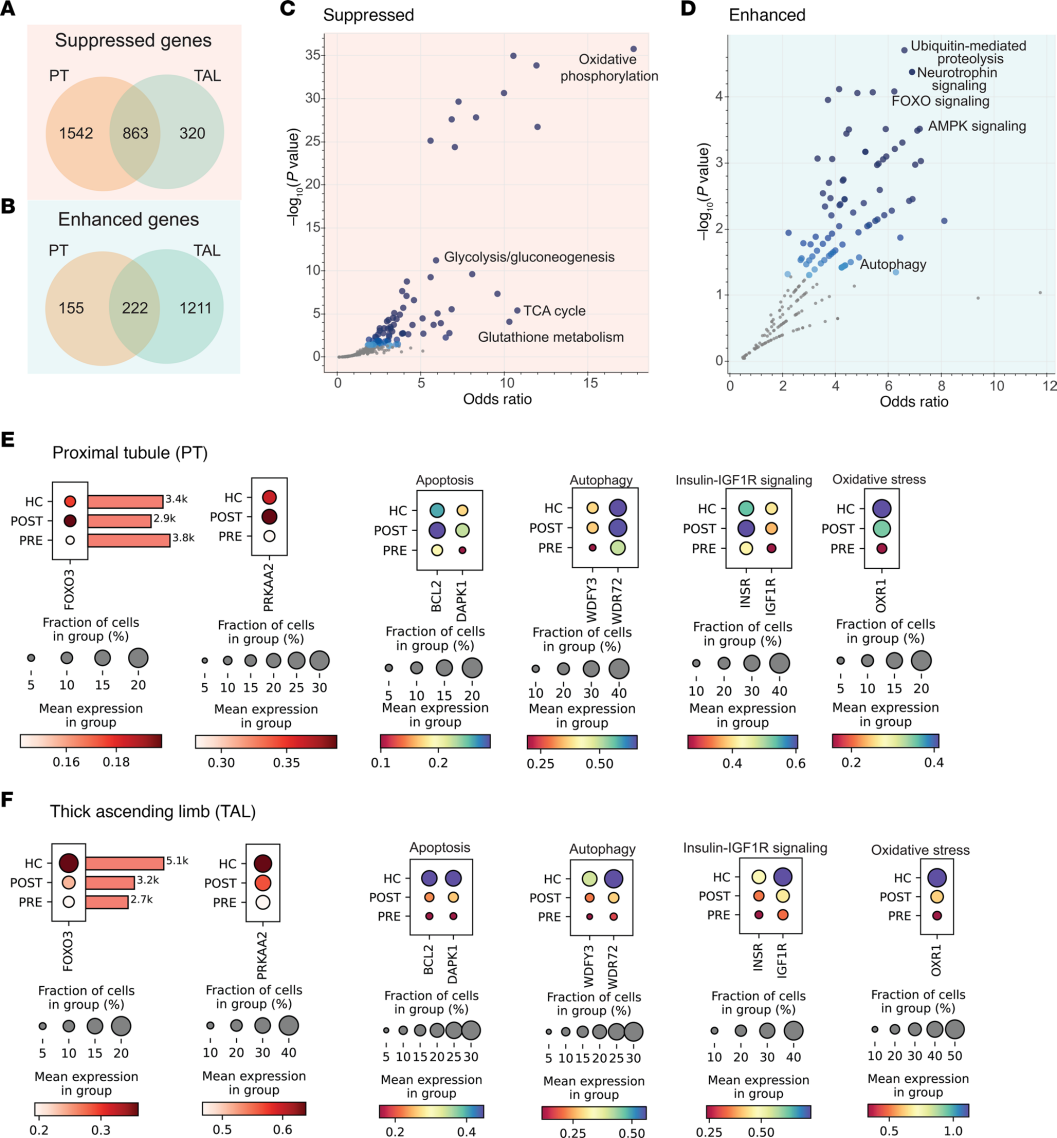
Venn diagrams and Kyoto Encyclopedia of Genes and Genomes pathway enrichment analyses of suppressed and enhanced genes in PT and TAL. (**A**) Venn diagram illustrating the overlap of genes suppressed in the PT and TAL. The intersection contains 863 suppressed genes, where 1,542 genes are uniquely suppressed in PT and 320 are uniquely suppressed in TAL. (**B**) Venn diagram showing the overlap of genes enhanced in PT and TAL. The intersection includes 222 enhanced genes, with 155 genes uniquely enhanced in PT and 1,211 uniquely enhanced in TAL. (**C**) Kyoto Encyclopedia of Genes and Genomes (KEGG) 2011 pathway enrichment analysis of the suppressed gene intersection. The top pathways related to metabolism include oxidative phosphorylation, glycolysis, the TCA cycle, and glutathione metabolism. (**D**) KEGG 2011 pathway enrichment analysis of the shared enhanced genes. The top pathways include ubiquitin-mediated proteolysis, neurotrophin signaling, FOXO signaling, and AMPK signaling. (**E**) Expression levels of protein kinase AMP-activated catalytic subunit α2 (*PRKAA2*) and forkhead box O3 (FOXO3) in the PT along with representative genes related to apoptosis like BCL2 (*BCL2*, *P* < 0.05); death associated protein kinase 1 (*DAPK1*, *P* < 0.05); autophagy, including WD repeat and FYVE domain containing 3 (*WDFY3*, *P* < 0.05) and WD repeat domain 72 (*WDR72*, *P* < 0.05); insulin receptor signaling (*INSR*, *P* < 0.05); insulin-like growth factor 1 receptor (*IGF-1R*, *P* < 0.05); and resistance to oxidative stress, including oxidation resistance 1 (*OXR1*, *P* < 0.05). (**F**) Same set of genes in the TAL.

Given the similarities in gene expression and pathway changes post-VSG in PT and TAL, particularly in metabolic and signaling pathways (Supplemental Figures 2 and 3), we examined the overlap of DEGs between these 2 cell types to identify shared response pathways, which may highlight core mechanisms of action across the nephron. A total of 863 genes were downregulated, and 222 genes were upregulated in both PT and TAL post-VSG (Figure 7, A and B). Pathway enrichment of these DEGs was performed using Kyoto Encyclopedia of Genes and Genomes (KEGG; version 2021) (17). Top downregulated pathways included oxidative phosphorylation (OR: 17.08, *P* ≤ 0.0001), the TCA cycle (OR: 10.72, *P* ≤ 0.0001), glycolysis/gluconeogenesis (OR: 5.81, *P* = 0.0001), and glutathione signaling pathway (OR: 4.12, *P* = 0.02) (Supplemental Table 4 and Figure 7C). Top upregulated pathways included ubiquitin-mediated proteolysis (OR: 6.34, *P* = 0.004), neurotrophin signaling (OR: 6.62, *P* = 0.004), forkhead box O3 (FOXO3) mediated signaling (OR: 5.97, *P* = 0.004), and adenosine monophosphate protein kinase (AMPK) signaling (OR: 5.67, *P* = 0.007) (Supplemental Table 4 and Figure 7D).

In the overlap analysis, both AMP-activated catalytic subunit α2 (*PRKAA2*) and *FOXO3* were upregulated in the PT and TAL (Figure 7, E and F, and Supplemental Table 5). We then further evaluated the expression of processes known to be regulated by AMPK/FOXO3 (18). In addition to the genes related to metabolic adaptation discussed earlier, there was upregulation of genes related to apoptosis like BCL2 apoptosis regulator (*BCL2*, *P* < 0.05); death associated protein kinase 1 (*DAPK1*, *P* < 0.05); autophagy including WD repeat and FYVE domain containing 3 (*WDFY3*, *P* < 0.05) and WD repeat domain 72 (*WDR72*, *P* < 0.05); insulin receptor signaling (*INSR*, *P* < 0.05); insulin-like growth factor 1 receptor (*IGF-1R*, *P* < 0.05); and resistance to oxidative stress, including oxidation resistance 1 (*OXR1*, *P* < 0.05), after VSG. Consistent with these data key cellular processes, such as autophagy (OR: 4.17, *P* = 0.02), apoptosis (OR: 4.01, *P* = 0.03), insulin-signaling pathway (OR: 4.91, *P* = 0.01), and longevity regulating pathways, which is closely related to signaling through IGF-1R (OR: 5.7, *P* = 0.01), were upregulated (Supplemental Table 6). There was also a trend toward increased mitophagy (OR: 4.25, *P* = 0.10).

### In vitro evaluation of nutritional deprivation on renal tubular cells.

To support the hypothesis that the metabolic reprogramming observed in tubular cells in kidneys reflected reduced serum glucose, amino acids, and growth factors post-VSG (19–24), we performed in vitro experiments examining the effects of serial dilutions of epithelial basal media on HK-2 human kidney epithelial cells. Progressive nutrient withdrawal induced a stepwise reduction in pS6K1/S6K1 and corresponding increase in pAMPK/AMPK ratio (Supplemental Figure 4). At 2.5% medium, mTORC1 activity was nearly abolished, comparable to 50 ng/mL rapamycin. Importantly, pAkt/Akt ratio remained unchanged (~0.83–0.98), indicating that reduced mTORC1 activity reflects direct nutrient sensing rather than Akt/PI3K-mediated effects. Severe nutrient deprivation also induced robust AMPK activation (10- to 12-fold vs. 4-fold with rapamycin). These in vitro findings recapitulate our in vivo observations and provide mechanistic support that decreased caloric intake post-VSG directly modulates mTORC1 and AMPK signaling in tubular epithelium.

### Pathway activities associated with VSG across PT.

**Figure 8:**
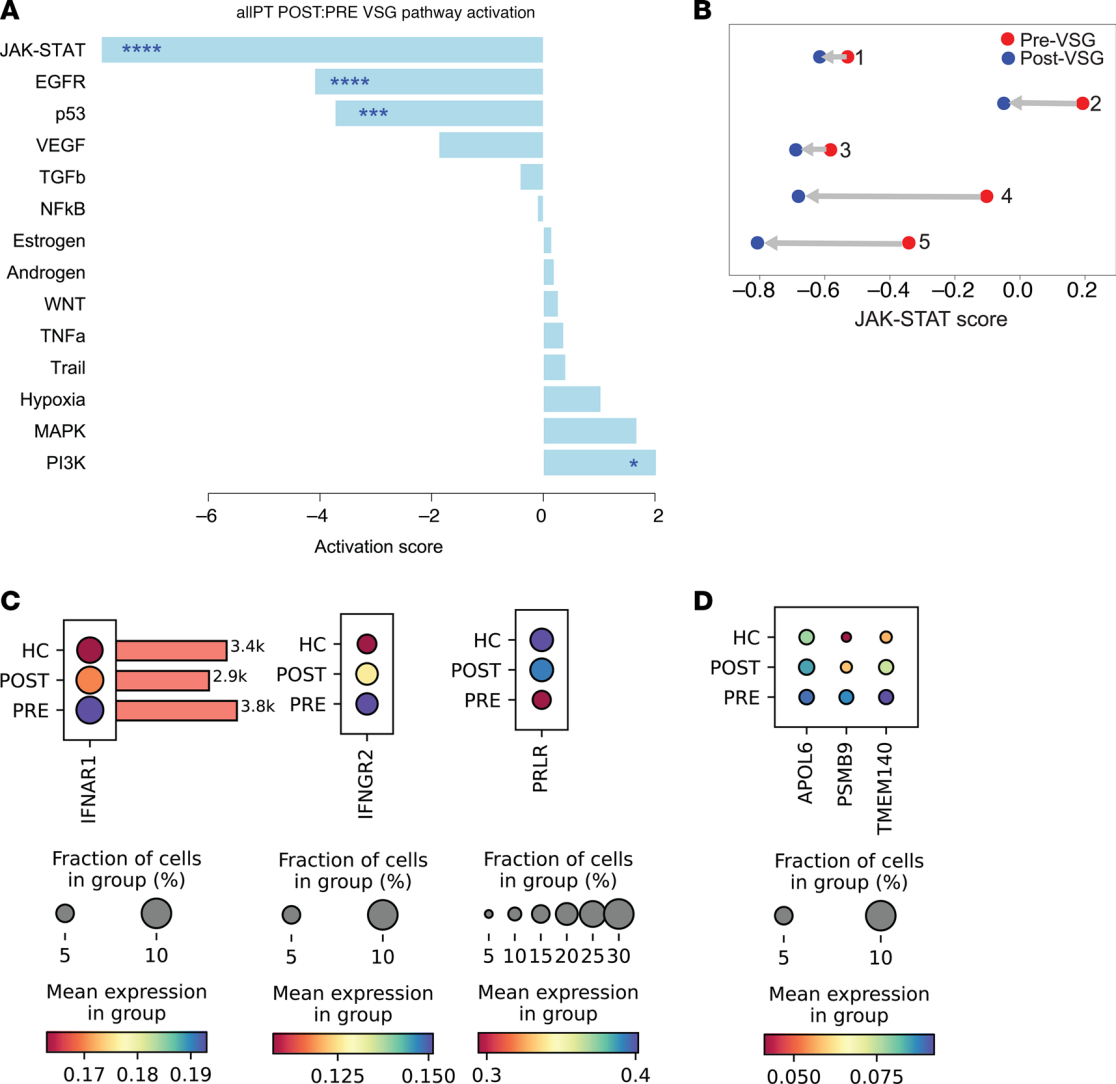
Pathway enrichment analysis of pseudobulk differential gene expression in proximal tubular epithelial cells. (**A**) The data reveal significant enrichment of the JAK/STAT signaling pathway, EGFR-mediated signaling, and p53 signaling, all of which were markedly reduced following VSG. *P <.05, **P <.01, ***P <.001, ****P <.0001. Wald’s test was used to determine *P* values. (**B**) JAK/STAT activation was assessed at the individual patient level using the single-sample gene set enrichment analysis (ssGSEA) method. A consistent reduction in JAK/STAT activation was observed across all patients post-VSG. (**C**) Expression levels of *IFNAR1* and *IFNGR2* (type 1 and type 2 interferon receptors, respectively), which signal through the JAK/STAT pathway, were significantly reduced post-VSG. This reduction was accompanied by decreased circulating levels of a type 1 interferon (IFNA7) but not type 2 interferons. Additionally, an increased expression of prolactin receptor (*PRLR*), a paralog of *GH1*, was noted. This upregulation is hypothesized to result from reduced circulating prolactin (PRL), triggering a compensatory negative feedback loop. (**D**) Average change in expression of 3 genes, apolipoprotein L6 (*APOL6*), proteasome 20S subunit β9 (*PSMB9*), and transmembrane protein 140 (*TMEM140*), which are among the genes used to calculate JAK/STAT activation score.

To evaluate additional pathways affected post-VSG, with a focus on therapeutically relevant signaling, we utilized the Pathway Responsive GENes (PROGENy) tool. PROGENy analysis of the pseudobulk PT profiles revealed that the major signaling pathways dysregulated post-VSG were Janus kinase/signal transducer and activator of transcription (JAK/STAT) pathway, epidermal growth factor (EGFR), and p53 signaling pathways (Figure 8A). The previously published 17-gene JAK/STAT intrarenal signature quantifying downstream JAK/STAT pathway activity (25, 26) was used to show a decrease in JAK/STAT activity score from pre- to post-VSG in each participant (Figure 8B and Supplemental Table 7).

To evaluate whether the observed reduction in intrarenal JAK/STAT signaling correlated with systemic reductions in circulating JAK/STAT ligands post-VSG, we analyzed data from a larger cohort (Teen-LABS; *n* = 64), where plasma SomaScan proteomic data collected within 2 weeks before and 1 year after MBS were available (13). Supplemental Table 8 summarizes demographic and clinical information for this Teen-LABS cohort.

In Table 2 summarizes changes after VSG in levels of circulating ligands that signal via JAK/STAT that met an FDR threshold of less than 0.05 in the Teen-LABS cohort. Ligands with significantly reduced circulating levels included cytokines such as IL2, IL6, IL23, and IFNA7 (27–30). Additionally, we observed reductions in levels of circulating growth hormone receptor (GHR), a marker of growth hormone bioactivity on the SomaScan platform (31), and of prolactin (PRL), both of which bind receptors that signal via JAK/STAT (32–34).

In evaluating the corresponding receptors for these circulating ligands in the PT, we observed reduced expression of the type 1 interferon receptor (*IFNAR1*, *P* < 0.0001) and a type 2 interferon receptor (*IFNGR2*, *P* < 0.0001) (Figure 8C), consistent with reduced active signaling from ligands not produced in the PT and likely to be of systemic origin. Known transcripts for JAK/STAT signaling receptors for other circulating ligands, including IL2, IL6, IL12, IL23, and leptin, were not expressed in the PT. GHR protein is expressed in the PT (The Human Protein Atlas), but *GHR* mRNA was not differentially expressed in PT. However, there was significantly increased expression of the prolactin receptor (*PRLR*) in PT after VSG (Figure 8C). Finally, in Figure 8D, expression changes in 3 representative genes from the 17-gene JAK/STAT intrarenal signature are shown as a visual for underlying changes encapsulated in the score presented in Figure 8B.

## Discussion

The paired evaluation of kidney biopsies pre- and post-VSG provided an opportunity to identify molecular mechanisms underlying kidney recovery in youth with T2D and/or severe obesity following rapid weight loss. Improvements in clinical and metabolic parameters were accompanied by regression of early kidney disease markers, including kidney hypertrophy, glomerular hyperfiltration, mesangial matrix expansion, and albuminuria, underscoring the efficacy of VSG in alleviating early kidney injury. Integrated transcriptomic and proteomic analyses of kidney tissue highlighted attenuation of mTORC1 activity and transcriptional evidence, which suggested increased AMPK- and FOXO3-mediated signaling post-VSG. Further, suppression of intrarenal JAK/STAT signaling emerged as another key pathway linked with kidney recovery, suggesting potential targets for therapeutic intervention in these youth at high risk for kidney disease.

Given the observed regression in kidney hypertrophy, improved kidney and metabolic function parameters, and reduced expression of metabolism-related transcripts post-VSG, we focused on the mTORC1 signaling pathway. mTORC1 is a master regulator of cell growth and metabolism, and its hyperactivation is observed in DKD (35–40). Increased intrarenal mTORC1-mediated signaling activation is linked to podocyte damage, proteinuria, tubular cell injury, and increased kidney fibrosis critical to the progression of DKD (35–38). Further, reducing intrarenal mTORC1-mediated activity was associated with attenuation of kidney disease by reducing rates of oxidative phosphorylation, anaerobic glycolysis, and changes in ROS-related processes (41), consistent with the transcriptional changes in this study. Sodium-glucose cotransporter 2 inhibitors (SGLT2i), which reduce the entry of tubular glucose into PT cells, have been shown to suppress glycolysis, gluconeogenesis, TCA cycle, and fatty acid oxidation transcripts in PT, which are associated with decreased mTORC1 activity (16). The nutrient deprivation in vitro model in this study indicates that the reduced activation of the mTORC1 pathway post-VSG might, in part, lead to similar molecular phenotypes resulting from VSG and SGLT2i treatments (Supplemental Figure 5).

Despite similarities in transcriptomic responses in PT and TAL with VSG and SGLT2i treatments (16), there were some key differences. Transcripts associated with glycolysis, gluconeogenesis, and TCA cycle were downregulated in PT post-VSG but were upregulated in TAL with SGLT2i treatment (16). A possible explanation for this difference could be that the increase in sodium and glucose delivery to TAL is much higher with SGLT2i treatment while likely reduced or unchanged post-VSG. Despite these differences, *EGF* expression in TAL increased with both treatments. Increased urinary EGF is a prognostic biomarker for better kidney outcomes (42, 43); however, beneficial effects of increased EGF expression in the nephron have not yet been confirmed. Long-term follow-up studies involving MBS and SGLT2i treatment may shed light on increased EGF expression in kidneys and long-term kidney function.

To delineate core mechanisms of kidney recovery, we evaluated shared transcriptional changes in PT and TAL post-VSG and identified enhanced AMPK/FOXO3 signaling in both cell types. The relationship between AMPK/FOXO3 and mTORC1 involves complex interactions that regulate cellular energy balance, growth, and survival (44–46). Activation of mTORC1 can directly inhibit AMPK while activation of AMPK, which acts as an energy and glucose metabolite sensing protein, can inhibit mTORC1 activity promoting cell survival under nutritional stress conditions (44–46). Thus, there exists a negative feedback loop between mTORC1 and AMPK (44). Upon activation, AMPK/FOXO3 signaling triggers a comprehensive metabolic reprogramming of cells that reduces cellular energy demands, increases energy production, and optimizes nutrient utilization by enhancing cellular nutrient turnover by autophagy and mitophagy (47, 48). In addition, the shared transcriptomic data were consistent with a reduction in cellular energy demands post-VSG, with suppression in genes and pathways associated with oxidative phosphorylation, glycolysis, and TCA cycle. There was also suppression of energy-intensive biosynthetic processes, including gluconeogenesis and lipid, cholesterol, protein, and DNA synthesis. Consistent with increased nutrient turnover in the context of decreased mTORC1 activity, there was a significant increase in autophagy pathways and a trend toward increased mitophagy. Reduced mTORC1 pathway activity with increase in autophagy and mitophagy pathways have been shown to be associated with improved kidney function in animal and SGLT2i treatment studies (49, 50).

However, changes in ROS-related processes such as glutathione metabolism were in the opposite direction to that predicted with enhanced AMPK/FOXO3 (48). One possible explanation is that, by 1 year post-VSG, reduced metabolic activity had led to decreased ROS generation, thereby reducing the need for transcripts that buffer against oxidative stress. Notably, while majority of ROS-related genes were downregulated, OXR1 expression increased, highlighting the complexity of ROS buffering regulation. An important caveat is that several of these metabolic changes have also been ascribed to reduced mTORC1 signaling; thus, the underlying mechanism driving the observed ROS changes cannot be definitively established by this study.

PROGENy pathway activity mapping identified changes in JAK/STAT transcriptional activation. This decrease was confirmed in the paired kidney biopsies using a kidney-focused 17-gene signature specific to the canonical JAK/STAT pathway (25, 26). Increased JAK/STAT signaling has been implicated in the pathogenesis of DKD, and targeting this pathway has been shown in diabetic mice to have therapeutic benefits in ameliorating kidney disease (51–54). In humans, a phase II randomized, double-blind, dose-response study utilizing baricitinib (a JAK1 and JAK2 inhibitor) demonstrated significant reductions in proteinuria and inflammatory cytokines in both urine and blood (55). However, awaiting phase III studies, JAK/STAT inhibitors are yet to be used clinically to treat DKD.

Other known drivers of kidney hypertrophy and JAK/STAT-mediated signaling pathways could also have contributed to the observed improvements in kidney-related clinical assessments post-VSG (31). For example, by 12 months post-VSG, GH bioactivity, measured by the significant reduction in circulating GHR on the SomaScan platform, was reduced in the Teen-LABS cohort (Table 2). In addition to its known role in driving kidney hypertrophy through GHR-mediated IGF-1 signaling (56), GH also signals through the JAK/STAT signaling pathway (57). Thus, a reduction in its bioactivity could contribute to the regression in kidney hypertrophy and concomitant reduction in JAK/STAT signaling, suggesting GHR-mediated signaling in kidneys as another potential therapeutic target. The apparent discrepancy between reduced circulating GH bioactivity with no change in *GHR* mRNA expression in the PT could be the result of unchanged promoter activity, transcription factor binding, or epigenetic modifications at the *GHR* locus, consistent with prior studies assessing kidney GHR expression in the setting of reduced circulating GH (58). A limitation of this study is the absence of protein expression data in kidneys.

**Figure 9:**
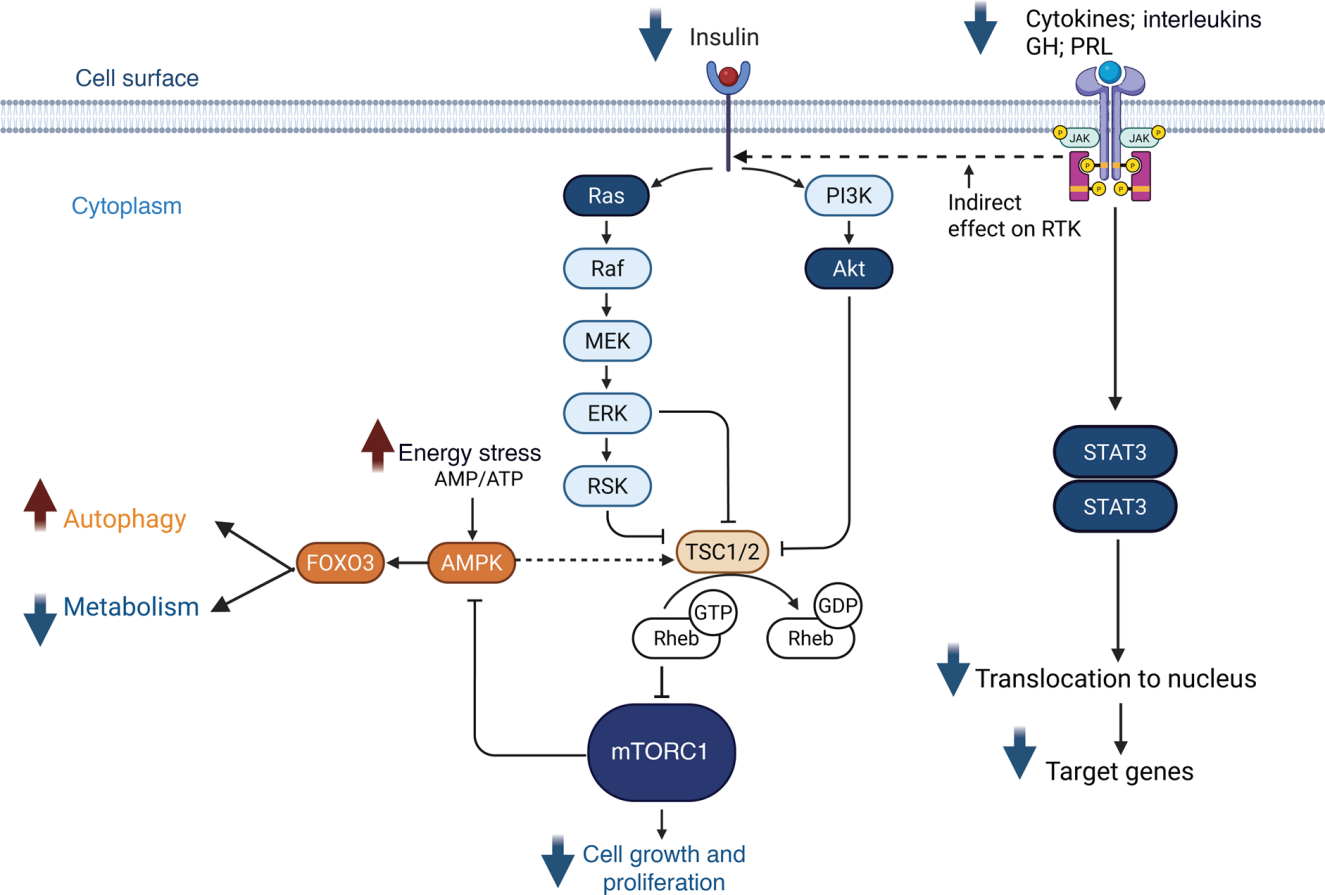
Integrated signaling pathways regulating intrarenal mTORC1 and JAK/STAT signaling in response to reduced insulin, relative energy stress, and reduced circulating ligands that activate the JAK/STAT pathway. Reduced insulin availability leads to diminished activation of Ras and PI3K, subsequently decreasing signaling through the Raf/MEK/ERK/RSK and Akt branches. Both ERK/RSK and Akt negatively regulate the TSC1/2 complex. Reduced ERK/RSK and Akt signaling enhances TSC1/2 activity, which attenuates activation of Rheb-GTP and consequently inhibits mTORC1, thereby suppressing cell growth and proliferation. Under conditions of lowered blood glucose and relative energy stress (elevated AMP/ATP ratio) post-VSG, AMPK can be activated and inhibits mTORC1 directly, as well as indirectly via activation of TSC1/2. mTORC1 can inhibit AMPK in a reciprocal feedback loop. AMPK activation further promotes FOXO3, which drives transcriptional programs that enhance autophagy, mitophagy, apoptosis, and ROS defense while suppressing metabolic activity. Thus, mTORC1 and AMPK/FOXO3 pathways exhibit opposing responses to energy and growth factor signals, coordinating anabolic and stress-adaptive cellular programs. Additionally, reduced levels of circulating cytokines, interleukins, prolactin (PRL), and growth hormone (GH) result in decreased activation of their respective receptors and the downstream intrarenal JAK/STAT signaling cascade. The JAK/STAT pathway can also interface with the Ras/MAPK and PI3K/Akt pathways (dashed horizontal line), further modulating the integrated cellular response. BioRender. Naik, A. (2025) https://BioRender.com/6fif35r PI3K, phosphatidylinositol-3-kinase; Akt, v-akt murine thymoma viral oncogene homolog 1; Ras, rat sarcoma virus oncogene homolog; Raf, rapidly accelerated fibrosarcoma; MEK, mitogen-activated protein kinase; ERK, extracellular signal–regulated kinase; RSK, ribosomal S6 kinase; mTORC1, mechanistic target of rapamycin complex 1; TSC1/2, tuberous sclerosis complex 1 and 2; Rheb, ras homolog enriched in brain; AMPK, AMP-activated protein kinase; FOXO3, forkhead box O3; AMP/ATP, adenosine monophosphate/adenosine triphosphate.

Circulating prolactin, a GH paralog that also signals through the JAK/STAT pathway, was also reduced in the Teen-LABS cohort (Table 2). Prolactin is localized along with its receptor in the PT and is thought to play a role in driving hypertrophy in some organs, though its role in kidney hypertrophy is yet to be established (32–34, 59–61). Plasma leptin, another activator of the JAK/STAT pathway, was also significantly reduced (62). In addition, there was a decrease in several interleukins and the type 1 interferon IFNA7 (Table 2) (27–30). Reductions in one or several of these ligands might contribute to the observed reduction in JAK/STAT signaling in kidneys post-VSG. Figure 9 schematically summarizes potential molecular mechanisms underlying PT recovery post-VSG. This study focused on PT and TAL since these had the highest DEGs. However, gene expression changes occurred post-VSG in several other cell types, including DCT, PC, IC, and EC. These and other glomerular cell types are yet to be evaluated and are planned for future studies.

Our findings raise the possibility that MBS including VSG, potentially through caloric restriction, directly and/or indirectly, elicits broader cellular reprogramming effects consistent with known protective paradigms in kidney biology. In fact, these data mirror core mechanisms seen in preclinical models of caloric restriction, where suppression of mTORC1, activation of AMPK/FOXO3, and enhanced autophagy have been shown to protect against diabetic nephropathy and renal aging (46, 63–67). In preclinical studies, caloric restriction has consistently attenuated glomerulosclerosis, reduced renal fibrosis, and improved mitochondrial homeostasis through nutrient-sensing and stress-response pathways that closely parallel those observed here post-VSG. A similar molecular phenotype has also been observed with the use of glucagon-like peptide 1 receptor agonists (GLP-1RAs) (68–70) which also cause caloric restriction due to reduced appetite, delayed gastric emptying, or early satiety (71). Thus, the effects of MBS intersect with those of GLP1-RA or caloric restriction and modulate the same signaling nodes, including mTORC1, JAK/STAT, and FOXO3/AMPK. The presence of a shared molecular phenotype, accompanied by preservation of kidney function, suggests that targeting the above signaling pathways could preserve kidney function in youth and adults.

This paired biopsy study revealed mechanistic insights with therapeutic implications. The molecular findings were complemented by tissue morphology analysis, kidney size assessment, kidney function evaluation, and metabolic profiling, providing multiple data streams in an integrated systems biology approach. Although only 5 of the 15 patients in the IMPROVE-T2D cohort underwent sequential kidney biopsies, these paired biopsies minimized variability by allowing direct pre- and post-VSG comparisons within the same participant. This paired approach is particularly relevant given the scarcity of tissue-level data in young persons with T2D and obesity. While the expression of several metabolism-related genes was downregulated, metabolite data are lacking to validate our findings. However, previous studies have demonstrated that even small transcriptional changes corresponded with metabolic flux studies in DKD and that transcriptional changes correlate with protein and metabolite levels (72, 73). Furthermore, the reduction of downstream rpS6K staining indicative of reduced mTORC1 activity also aligns with the observed transcriptomic data. To explore the molecular basis of our tissue-based observations, specifically effects of the circulating proteome on kidney transcriptomics, we leveraged the Teen-LABS cohort. A limitation of the Teen-LABS study was that many participants did not have diabetes. Furthermore, some participants underwent MBS in the form of RYGB instead of VSG. Nonetheless, the plasma proteomic analysis from the Teen-LABS study enhanced and generalized the transcriptional findings in the IMPROVE-T2D study.

Epigenetic alterations, such as DNA methylation, can affect genes related to inflammation and fibrosis. Genes such as hypermethylation of Ras protein activator like 1 (*RASAL1*) and hypomethylation of myo-inositol oxygenase (*MIOX*) have been shown to contribute to fibrosis and oxidative stress. Additionally, histone modifications in the form of acetylation and methylation are also linked to both pro-fibrotic and inflammation-related genes (74–77). Notably, DNA methylation, for example, via increased DNA methyltransferases (DNMT1) leads to hypermethylation of negative regulators of mTOR, resulting in mTORC1 pathway activation, leading to increased inflammation in DKD. Targeting DNMT1 or reversing the methylation was shown to reduce immune cell–driven inflammation and showed improved outcomes in experimental models (78). However, we did not assess these patients to test for epigenetic modifications. Similarly, another limitation is that no metabolomic profiling was done in this study. However, based on the observed pathway changes (reduced mTORC1, enhanced AMPK/FOXO3), we predict that there would be decreased branched-chain amino acids (which activate mTORC1), reduced TCA cycle intermediates reflecting normalized energy metabolism, and lower lipotoxic species (di-/triglycerides). These predictions align with emerging data from bariatric cohorts (79).

In summary, our study reveals fundamental molecular mechanisms contributing to kidney recovery following VSG in youths with T2D and severe obesity. We identified key regulatory networks, particularly the mTORC1-, AMPK/FOXO3-, and JAK/STAT-mediated signaling pathways in PT and TAL, that appear to orchestrate metabolic reprogramming and facilitate recovery from early kidney injury. These findings advance our understanding of how VSG drives the beneficial kidney effects in youth-onset T2D and offer promising therapeutic targets for treating diabetes and obesity-related kidney disease in these high-risk youth.

## Methods

### Sex as a biological variable.

Both male and female participants were enrolled and included in the analysis. The sample size, *n* = 5, was too small to make any meaningful comparisons.

### IMPROVE-T2D.

Adolescents 12–21 years with confirmed antibody-negative, youth-onset T2D (negative for insulin, glutamic acid dehydrogenase, islet cell, and zinc transporter antibodies) and obesity, scheduled for VSG, were recruited from the pediatric MBS clinic at Children’s Hospital Colorado. Participants could opt in for an optional research biopsy before and 12 months after VSG (9). HC transcriptomic data were derived from kidney biopsy tissue from 6 healthy adult volunteers from a separate study and used as reference for baseline expression levels (16). No statistical comparisons were made between VSG and HC. No new nephroprotective drugs, such as renin-angiotensin system blockers and SGLT2i, were prescribed during the study period post-VSG. Participants maintained medications, including angiotensin converting enzyme inhibitors or angiotensin receptor blockers, prescribed prior to study enrollment, per standard of care.

### GFR and renal plasma flow by iohexol and p-aminohippurate clearances and UACR.

Iohexol was administered by bolus IV injection (5 mL of 300 mg/mL [Omnipaque 300, GE Healthcare, now Cytiva]). Blood collections for iohexol plasma disappearance were drawn at +120, +150, +180, +210, and +240 minutes (80) and detailed in Supplemental Methods. UACR was calculated as (urine albumin [mg]/urine creatinine [g]) measured fasting from spot urine samples before and after renal clearance assessment and averaged. Participants with average UACR ≥ 30 mg/g were considered to have elevated albuminuria.

### Insulin secretion and insulin sensitivity by modified hyperglycemic clamp.

A hyperglycemic clamp was performed on participants resting in bed to determine insulin secretion and insulin sensitivity (9, 81) as detailed in Supplemental Methods.

### Multiparametric and functional kidney MRI.

Participants underwent a multiparametric kidney MRI scan on a Skyra Siemens Magnetom 3 T scanner. The conventional exam included T1 and T2 weighted axial and coronal images. TKV of each kidney was computed using Analyze software (Analyze 14.0, Mayo).

### Ultrasound-guided kidney biopsies and tissue processing.

Ultrasound-guided percutaneous kidney biopsies were performed by one of two highly experienced interventional radiologists. Per local protocol, up to 4 passages were allowed to obtain 3 biopsy cores. Each core was immediately assessed for the presence of cortex by gross examination and digital imaging. Kidney tissue was placed in specific fixatives and shipped to the University of Michigan, according to a modified version of the Kidney Precision Medicine Project (KPMP) Pathology (Protocol https://drive.google.com/file/d/1wX1sy5GCzLJzA7jSwEyySb5m5gV4N9T5/view).

### Quantitative morphometrics.

Light microscopy sections were scanned at ×40 using the Aperio GT450 DX Scanner from Leica Biosystems for pathological diagnosis. For quantitative assessment of glomerular and mesangial volume and mesangial nuclear count, all glomeruli present in a 3 μm formalin-fixed, paraffin-embedded section of each specimen were stained with periodic acid–Schiff and assessed using quantitative morphometrics as previously described (82, 83). Mesangial index was expressed as percentage mesangial matrix area per glomerular volume.

### IF staining.

The paraffin-embedded sections were deparaffinized and subjected to heat-induced antigen retrieval with a sodium citrate buffer (pH 6.0), then incubated in blocking buffer containing 2% BSA and 50% CAS-Block reagent (008120, Thermo Fisher Scientific) for 1 hour at room temperature followed by rabbit monoclonal phospho-S6 antibody (S240/S244, 5364, Cell Signaling Technology) at 1:500 dilution in the blocking buffer overnight at 4°C. The phospho-S6 signal was visualized using anti-rabbit Alexa Fluor 594 secondary antibody (catalog A-11012; Lot 3112802, Invitrogen).

### Single-cell RNA sequencing of kidney tissue.

scRNA-seq profiles were obtained using KPMP protocols (14) and detailed in Supplemental Methods. Clusters were annotated based on previously established kidney cell markers (14–16).

### Differential expression analysis at the single-cell level.

To identify transcripts associated with VSG surgery, we performed differential gene expression analysis using the limma (v3.48.3) and edgeR (v3.34.0) R packages. Raw count matrices were converted to log_2_ counts per million (logCPM) using the cpm function in edgeR with a prior count of 3 to stabilize variance and reduce the impact of zero inflation. A paired linear model was fitted using lmFit function in limma, with surgery condition (before vs. after) as the main variable and patient ID as a blocking factor to account for repeated measures. Empirical Bayes moderation was applied using eBayes function from limma to improve variance estimation. Statistical significance was assessed using the Benjamini-Hochberg procedure, and transcripts with FDR-adjusted *P* < 0.05 were considered differentially expressed. Genes with log_2_FC < 0 were defined as “suppressed” with VSG and those with log_2_FC > 0 as “enhanced.” The analysis was performed separately for each cell type to identify cell type–specific changes.

### Pathway enrichment analysis.

All significantly suppressed and enhanced transcripts were subjected to enrichment analysis using the enrichR package (version 3.0) and the Reactome_2022 gene set database. Pathways were considered significant if they had a *P* value below 0.05 and included at least 5 differentially expressed transcripts. Enriched pathways were visualized and categorized to highlight biological processes relevant to VSG-induced transcriptional changes. For the shared transcriptomic analysis between PT and TAL cells, we used the KEGG 2021 (17).

### Pseudobulk mRNA expression profiles.

To minimize the risk of false discovery in single-cell data after the aforementioned processing steps, the resulting count matrix was reduced to its equivalent pseudobulk representation by aggregating counts for each cell into its corresponding sample × cell type bin over 10 samples and 33 cell types (clusters) (84). Thus, the original matrix of 29,772 cells and 23,598 features (genes) was reduced to a matrix of the same number of genes and 309 sample/cell type bins (of a possible 330, with 21 unpopulated). The source and derived pseudobulk matrices were confirmed to have identical counts (122,844,981 total). From this, cell type–specific expression matrices were extracted; 5 PT subtypes were combined into a single PT expression matrix, and similarly a composite TAL matrix was generated from the 3 TAL subtypes. The resulting PT matrix comprised 28,063,977 total counts over 6,652 cells, and the TAL matrix 28,796,492 counts from 5,903 cells. With certain caveats when sparse data from less abundant cell types were considered, these cell type–specific counts matrices were treated similar to bulk RNA-seq data and analyzed using standard bulk RNA-seq differential expression tools (DESeq2), with the individual as the statistical unit.

### JAK/STAT pathway activity.

JAK/STAT pathway activity was inferred using PROGENy v1.11.3 (85) and the mlm (multivariate linear model) method in the decoupleR_2.9.7 package for R (86). PROGENy leverages a comprehensive compendium of publicly available datasets from various signaling perturbation experiments. Unlike traditional gene set enrichment analyses that focus on the expression levels of genes downstream of canonical signaling pathways, PROGENy reflects pathway activation states (85). Differential expression analysis was first performed with limma (v3.48.3) on the cell type–specific expression matrices, contrasting pre- and post-VSG gene expression, and *t* statistics therefrom were then regressed by the run_mlm function in decoupleR across PROGENy’s 14 canonical expression perturbation profiles. The obtained coefficients were interpreted as activation scores for each of the pathways. Their *P* values describe the goodness of fit between the differential expression profile under test and those of the pathways in the PROGENy database (experimentally derived) and not influenced by the number of cases in the groups under comparison.

JAK/STAT activation was also assessed using the single-sample gene set enrichment analysis (ssGSEA) method in the GSVA R package, version 1.40.1 (87). ssGSEA is a nonparametric method that uses rank-order statistics of a gene set within the larger population of measured genes to assign a score to each case individually. Here, a previously published (25, 26) set of 17 JAK/STAT target genes (*APOL1*, *APOL6*, *CCDC68*, *CCL2*, *CTSS*, *CXCL9*, *CXCL10*, *GBP1*, *GBP2*, *ICAM1*, *IDO1*, *IRF1*, *ITK*, *PDCD1LG2*, *PSMB9*, *TMEM140*, *TRIM21*) was evaluated for each of 5 patients in the post- versus pre-VSG conditions. These scores were then compared between the 2 groups using a Welch 2-sample paired *t* test.

### Teen-LABS.

The Teen-LABS study was a prospective, longitudinal, multicenter observational study enrolling adolescents undergoing MBS between March 2007 and February 2012 with overall methods and baseline characteristics of the cohort previously reported (10). In 2021, longitudinal multiprotein signatures were measured in stored plasma specimens from 64 of the original 242 participants with plasma samples available at baseline and at least 1 follow-up time point. In this cohort 73% had undergone RYGB, 22% VSG, and 4.7% laparoscopic adjustable band. Of these patients, 37% had diabetes. The plasma proteomics was performed using the SomaScan 7K platform (SomaLogic) at Washington University, St. Louis, Missouri, USA. Internal controls were run with each sample and were normalized for intraplate and interplate variation (88). The SomaScan 7K platform comprises 7,604 aptamers corresponding to 6,596 human proteins.

### Statistics.

Clinical characteristics were summarized with mean and standard deviation for normally distributed continuous variables, median and interquartile ranges for non-normally distributed continuous variables, and count and percentage for categorical variables. Changes in circulating proteins between baseline and 1 year after MBS were estimated using linear mixed effects models with a contrast applied to the estimated marginal means. Estimated marginal means are expressed as percentage change from baseline. A *P* value of less than 0.05 was statistically significant.

### Study approval.

Informed consent was obtained by qualified research personnel. The IMPROVE-T2D study was approved by the Colorado Multiple Institutional Review Board (ClinicalTrials.gov ID NCT03620773). Participants ages 18 years or older signed the consent form, and participants below 18 years signed the assent form in addition to the parent(s) or legal guardian signing the consent form. The Teen-LABS study was approved by the institutional review board at Ann and Robert H. Lurie Children’s Hospital of Chicago. Written informed consents were collected prior to participation.

### Data availability.

Gene expression data can be accessed from National Center for Biotechnology Information Gene Expression Omnibus GSE315877. A cellxgene instance has been created and is available at https://cellxgene.miktmc.org/pre_post_vsg

## Author contributions

FMA and ASN contributed equally to the development of this manuscript. The order is based on the lead taken by ASN in writing and coordinating the manuscript. MK and PB were instrumental in designing and envisaging the substudy and acquiring the funding to carry out the substudy that generated these data and therefore are the shared senior authors on this manuscript. PB, MK, ASN, VN, FMA, SE, KJN, THI, and MGC designed the primary IMPROVE-T2D and Teen-LABS research studies. PB, MK, JH, YJC, LP, PL, JAB, JFS, SY, KAF, THI, KJN, TJD, THI, JBH, KI, EAO, RM, YO, MGC, HEH, JJR, TJD, and SPS acquired or generated the data. ASN, FMA, PJM, JAS, JH, RM, YJC, LP, FA, JBH, KI, CS, MMK, LY, PP, and SPS analyzed the data. ASN, VN, SE, JAS, FA, KLT, KI, JAG, RGN, FCB, KJN, MK, PB, SP, DHVR, VGP, and YO played a significant role in interpreting the findings. ASN, MK, PB, KJN, FCB, RGN, and PN wrote the initial draft. All authors have reviewed and approved the final draft of the manuscript.

## Funding support

This work is the result of NIH funding, in whole or in part, and is subject to the NIH Public Access Policy. Through acceptance of this federal funding, the NIH has been given a right to make the work publicly available in PubMed Central.

- University of Washington with the American Diabetes Association.
- University of Michigan with Chan Zuckerberg Initiative.
- Breakthrough T1D.

## Supplementary Material

Supplemental data

ICMJE disclosure forms

Unedited blot and gel images

Supporting data values

## Figures and Tables

**Table 1:**
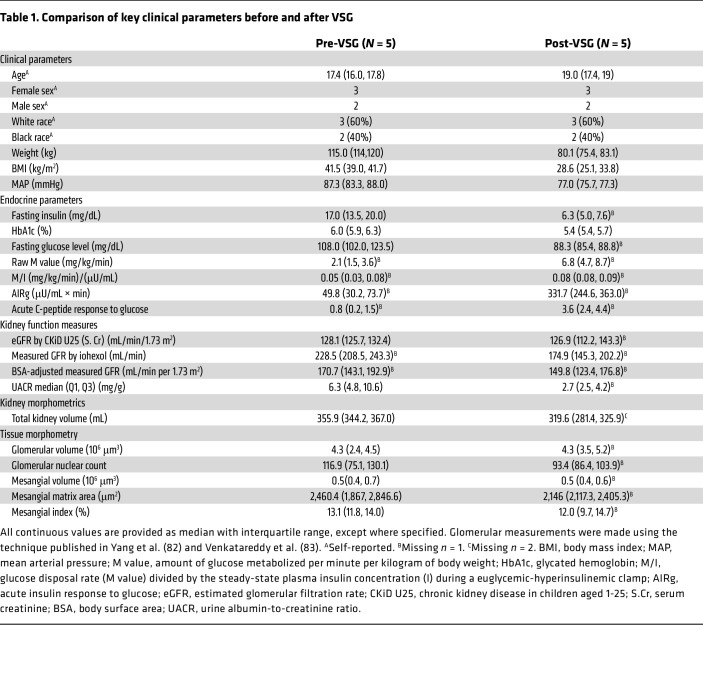
Comparison of key clinical parameters before and after VSG

**Table 2:**
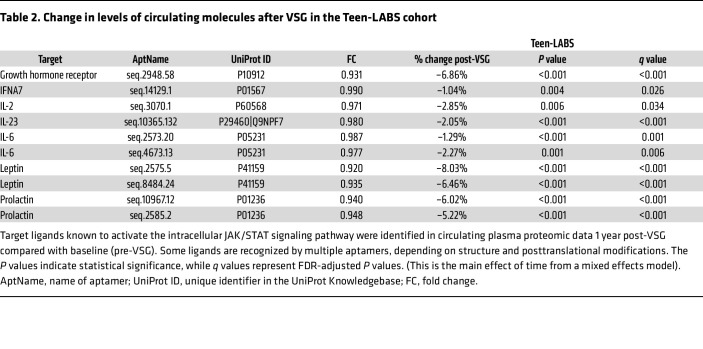
Change in levels of circulating molecules after VSG in the Teen-LABS cohort

## Version 1 02/10/2026

In-Press Preview

## Version 2 04/01/2026

Electronic publication
