# Supplementary material for: Metabolic surgery mitigates early kidney injury in obese youth with diabetes by suppressing mTORC1/JAK/STAT signaling
Source: J Clin Invest. 2026 Feb 3;136(7):e198545. doi: 10.1172/JCI198545 (PMC13038200; doi:10.1172/JCI198545)
Supplement: Supplemental data [file jci-136-198545-s126.pdf]

**List of supplementary materials**

**Supplementary Figures:**

**Supplementary Figure S1.** Single cell RNAsequencing cell type clusters by kidney biopsy group

**Supplementary Figure S2.** Reactome pathway analysis of proximal tubule (PT) Cells.

**Supplementary Figure S3.** Reactome pathway analysis of thick ascending limb (TAL) cells.

**Supplementary Figure S4:** Nutrient deprivation model in HK-2 cells

**Supplementary Figure S5:** Integrated signaling pathways comparing regulation of intrarenal mTORC1 and JAK-STAT signaling in response to reduced insulin, relative energy stress, reduction in circulating ligands versus those with SGLT2i.

**Supplementary Methods**

**Supplementary References**

**Conflict of Interest**

**Supplementary Tables:**

**Supplementary Table S1.** Clinical and demographic characteristics of participants without biopsies in the IMPROVE cohort

**Supplemental Table S2.** Differentially expressed genes Post vs. Pre in the PT

**Supplementary Table S3.** Differentially expressed genes Post vs. Pre in the TAL

**Supplementary Table S4.** Shared enhanced and suppressed pathways in PT and TAL. KEGG 2021 enrichment analysis of 863 suppressed and 222 enhanced genes in both PT and TAL.

**Supplementary Table S5.** All suppressed and enhanced genes in the PT only, TAL only, common to identify overlapping genes

**Supplementary Table S6.** Enhanced and suppressed pathways using KEGG 2021

**Supplementary Table S7.** JAK/STAT signature genes

**Supplementary Table S8.** TEEN-Lab's demographics and clinical characteristics

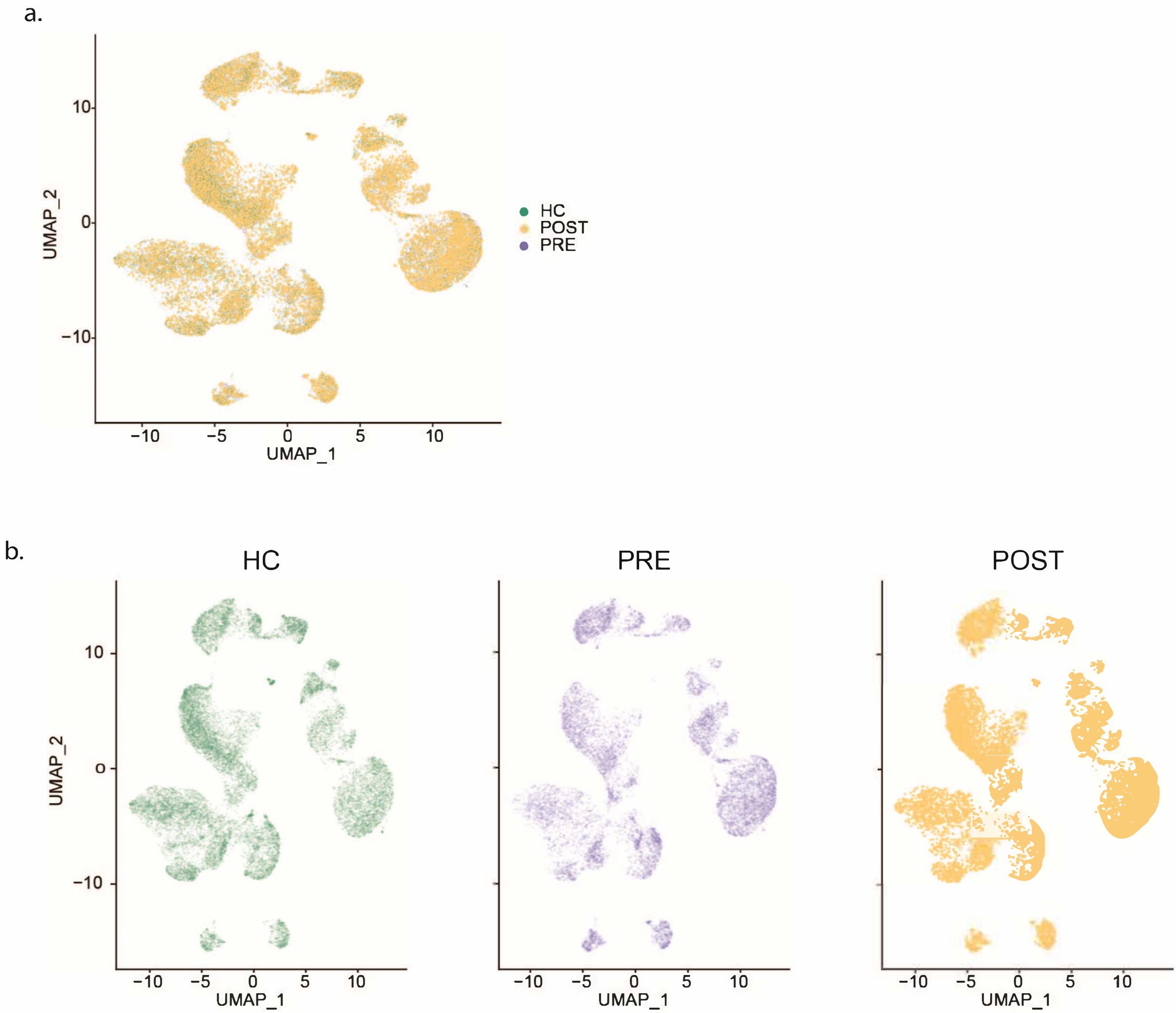


**Supplementary Figure S1. Single cell RNAsequencing cell type clusters by kidney biopsy group - healthy controls, biopsies pre and post VSG of IMPROVE participants**
A) A UMAP plot showing the different kidney cell types identified in the biopsies with representation from all through groups.
B) A UMAP plot displaying the distribution of kidney cells based on group classification: healthy controls (HC), pre-VSG, and post-VSG. Cells are colored according to their group.

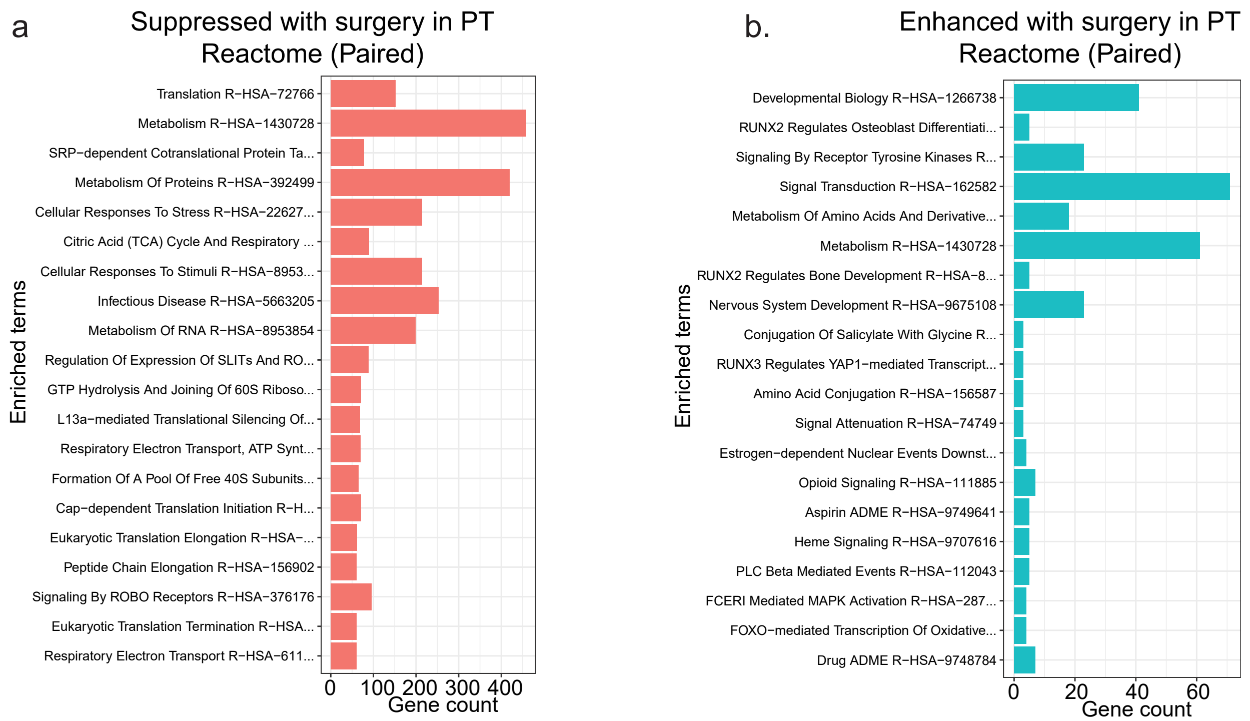


**Supplementary Figure S2: Reactome pathway analysis of proximal tubule (PT) Cells.** Reactome analysis of enriched pathways in the PT based on differentially expressed genes (FDR p-value < 0.05). **A.** Bar plot of the top 20 enriched pathways associated with suppressed genes in PT cells post-VSG surgery. **B.** Bar plot of the top 20 enriched pathways associated with enhanced genes in PT cells post-VSG surgery.

logFC

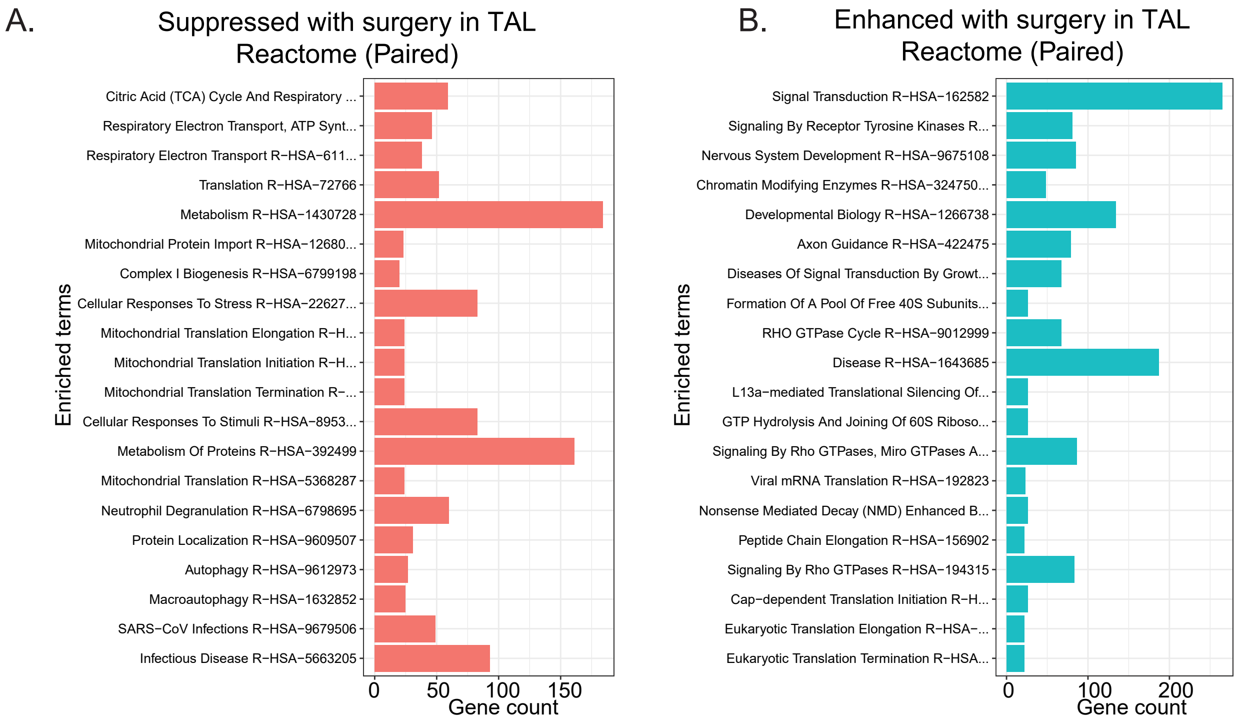


**Supplementary Figure S3: Reactome pathway analysis of thick ascending limb (TAL) cells.** Reactome analysis of enriched pathways in the TAL based on differentially expressed genes (FDR p-value < 0.05). **A.** Bar plot of the top 20 enriched pathways associated with suppressed genes in PT cells post-VSG surgery. **B.** Bar plot of the top 20 enriched pathways associated with enhanced genes in PT cells post-VSG surgery.

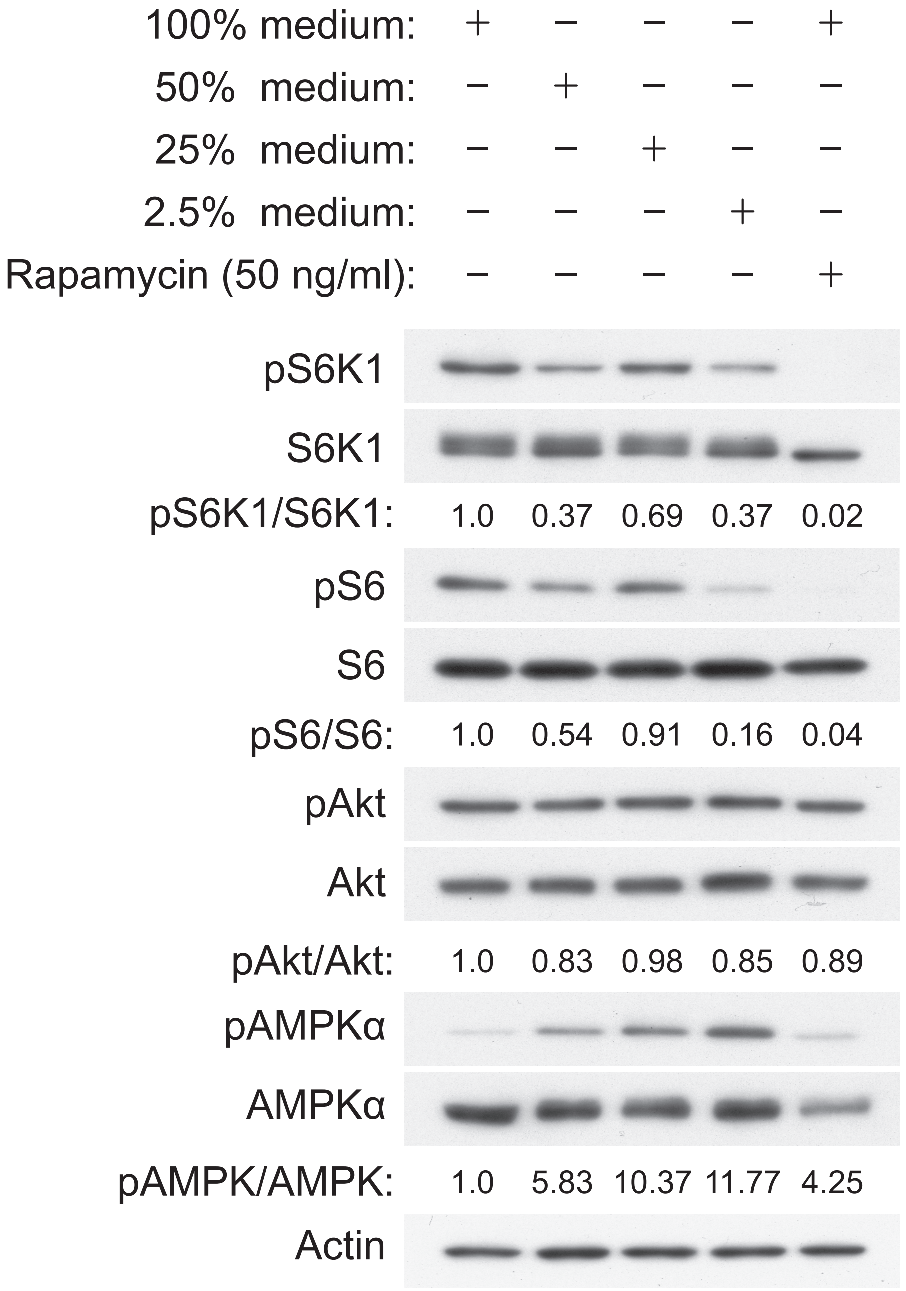


**Supplementary Figure S4: Nutrient deprivation model in HK2 cells.** Effects of nutrient deprivation and rapamycin on mTORC1, Akt, and AMPK signaling pathways. HK-2 cells were cultured for 48 hours under the following conditions (from left to right): Group 1 normal culture growth media (GM)-100%; Group 2 culture media diluted with RPMI1640 (1:1)-50%; Group 3 culture media diluted with RPMI1640 (1:3)-25%; Group 4 culture media diluted with RPMI1640 (1:40)-2.5%; Group 5 culture media plus rapamycin (50 ng/ml).

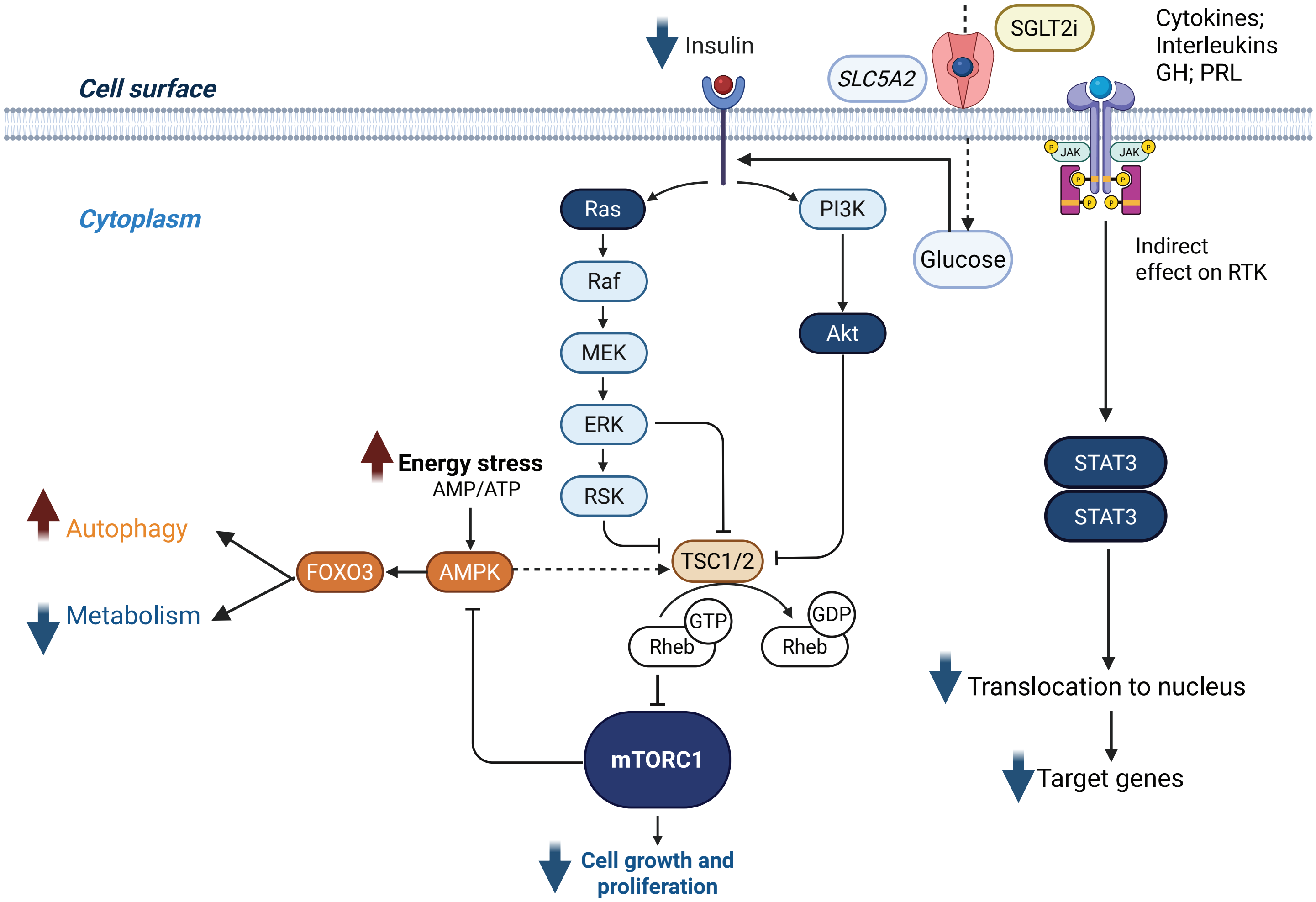


**Supplementary Figure S5: Integrated signaling pathways comparing regulation of intrarenal mTORC1 and JAK-STAT signaling in response to reduced insulin, relative energy stress, reduction in circulating ligands versus those with SGLT2i.** SGLT2i treatment reduces glucose entry into the PT leading to decreased activation of PI3K-Akt and the Ras-Raf-MEK-ERK signaling leading to reduced mTORC1 signaling. Created in BioRender. Naik, A. (2026) https://BioRender.com/ess1ha2

## Supplementary Methods

### *Glomerular filtration rate (GFR) and renal plasma flow (RPF) by iohexol*

The Jødal-Brøchner-Mortensen (JBM) equation was used to calculate the GFR. GFR was measured during mild hyperglycemia (goal blood glucose 190 mg/dL [10.6 mmol/l]), achieved by a modified hyperglycemic clamp with paired 20% dextrose, chosen to maintain steady-state glycemic concentrations during kidney measures. Iohexol measurements were performed by high-performance liquid chromatography (HPLC, Waters, Milford, MA) at the National Institute of Diabetes and Digestive and Kidney Diseases (NIDDK) laboratory in Phoenix, Arizona, as described previously(1).

### *Insulin secretion and insulin sensitivity by modified hyperglycemic clamp*

A 20% dextrose infusion was titrated to maintain hyperglycemia (≈190-200 mg/dL) for 240 min. Plasma glucose concentrations were measured at the bedside every 10 minutes by a glucose oxidase technique using a Stat Strip glucometer (Nova Biomedical). Samples were collected from a heated, arterialized hand vein. Insulin, free fatty acids (FFA), and C-peptide were collected at baseline (-10, -5 min), during the 10 min following an IV bolus of 20% dextrose with a target to achieve 190-200 mg/dL, as well as every 5-10 minutes during the steady-state from 190-240 min. To assess first-phase insulin secretion in response to acute hyperglycemia, the acute insulin response to glucose (AIRg) was calculated as the incremental area under the curve (AUC) for insulin levels during the first 10 minutes following glucose infusion, using the trapezoidal rule. Similarly, the acute C-peptide response to glucose (ACPRg) was estimated by computing the incremental AUC for C-peptide levels during the same initial 10-minute period. Both calculations subtracted the basal (pre-infusion) insulin and C-peptide levels respectively. The M-value, an index of glucose disposal rate used to assess insulin sensitivity, was calculated from the mean glucose infusion rate during the steady-state of the hyperglycemic clamp,

adjusted for body weight (mg/kg/min). The body composition by DXA (mg/lean kg/min) was measured using standard methods on a Hologic device (Waltham, MA) to determine lean and fat mass. A measure of insulin sensitivity, normalized by steady state insulin, was then calculated by dividing the M-value by the mean plasma insulin concentration during the steady state period of the clamp (M/I).

***Sample processing and single-cell RNA sequencing of kidney tissue***

Single cells were isolated from frozen tissues using Liberase TL at 37°C for 12 minutes. The single-cell suspension was immediately transferred to the University of Michigan Advanced Genomics Core facility for further processing. Sample demultiplexing, barcode processing, and gene expression quantifications were performed with the 10X Cell Ranger v6 pipeline using the hg38 GRCh38-2020-A reference genome. To remove ambient mRNA from the data, the Cell Ranger count matrices were processed using SoupX_1.5with default parameters(2). The resulting matrices were processed as previously described whereby cells were included only if gene counts were between 500 and 5000, with fewer than 50% mitochondrial genes. Individual matrices were then integrated using RunHarmony embedded in Seurat, version 4.0.0(3-7).

***HK-2 cell culture and treatments***

Human proximal tubule epithelial cells (HK-2 cells) were used at passage 5 and cultured in epithelial basal media (REBM Basal Medium (Cat# CC-3191, Lonza Biosciences) with REGM SingleQuots supplements (Cat# CC-4127, Lonza Biosciences) and with 10% fetal bovine serum. HK-2 cells were seeded at a density of 60,000 cells per well in six-well plates. At the start of the experiment, HK-2 cells were divided into 5 groups: Group 1 HK-2 cells cultured with normal culture growth media (GM)-100%; Group 2 HK-2 cells cultured with culture media diluted with RPMI1640 (1:1)-50%; Group 3 HK-2 cells cultured with culture media diluted with RPMI1640 (1:3)-25%; Group 4 HK-2 cells cultured with culture media diluted with RPMI1640

(1:40)-2.5%; Group 5 HK-2 cells cultured with culture media plus rapamycin (50 ng/ml). All Groups were treated for 48h. After 48h, mRNA and protein were isolated with TRIzol (Cat# 15596026, ThermoFisher Scientific) and NP40 lysis buffer (10 mM Tris-HCl [pH 7.5], 2 mM EDTA, 100 mM NaCl, 1% NP-40, 60 mM NaF, 10 mM pyrophosphate, 10 mM glycerophosphate, and a mixture of protease inhibitors (Roche), respectively.

***Immunoblotting***

Cells were harvested with NP40 lysis buffer (10 mM Tris-HCl [pH 7.5], 2 mM EDTA, 100 mM NaCl, 1% NP-40, 60 mM NaF, 10 mM pyrophosphate, 10 mM glycerophosphate, and a mixture of protease inhibitors (Roche). Lysates were then boiled in SDS sample buffer (20 mM Tris, pH 6.8, 2% SDS, 0.01% bromophenol blue, 10% glycerol, 5% 2-mercaptoethanol) and subjected to SDS-PAGE and immunoblotting. The SDS-PAGE gel was run in Tris-Glycine running buffer (25 mM Tris, 192 mM glycine, 0.1% SDS) at a voltage of 100V, and then the proteins were transferred to PVDF membrane in the transfer buffer (25 mM Tris, 192 mM glycine, 20% methanol) at a constant 350 mA for 3 hours at 4 °C. The membranes were incubated with the primary antibodies, Anti-pS6K1 (T389) (CST, Cat#9205), Anti-S6K1 (CST, Cat#2708), Anti-pS6 (S240/244) (CST, Cat#2215), Anti-S6 (CST, Cat#2217), Anti-pAkt (T389) (CST, Cat#9205), Anti-Akt (CST, Cat#9272), anti-pAMPK (T172) (CST, Cat#2535), anti-AMPK (CST, Cat#2532), or Ant-beta-actin (CST, Cat#3700). After incubation with the primary antibodies overnight at 4 °C and the secondary antibody for 2 hours at room temperature, the membrane was washed four times with 1 × TTBS buffer (20 mM Tris HCl pH 7.4, 150 mM NaCl, 0.1% Tween-20 (w/v)) before applying to enhanced chemiluminescence (Cat#RPN2232, GE).

**Supplementary References**


1. Nelson RG, Bennett PH, Beck GJ, Tan M, Knowler WC, Mitch WE, et al. Development and progression of renal disease in Pima Indians with non-insulin-dependent diabetes mellitus. Diabetic Renal Disease Study Group. *N Engl J Med.* 1996;335(22):1636-42.
2. Young MD, and Behjati S. SoupX removes ambient RNA contamination from droplet-based single-cell RNA sequencing data. *GigaScience.* 2020;9(12):giaa151.
3. Hao Y, Hao S, Andersen-Nissen E, Mauck WM, Zheng S, Butler A, et al. Integrated analysis of multimodal single-cell data. *Cell.* 2021;184(13):3573-87.
4. Butler A, Hoffman P, Smibert P, Papalexi E, and Satija R. Integrating single-cell transcriptomic data across different conditions, technologies, and species. *Nat Biotechnol.* 2018;36(5):411-20.
5. Satija R, Farrell JA, Gennert D, Schier AF, and Regev A. Spatial reconstruction of single-cell gene expression data. *Nature Biotechnology.* 2015;33(5):495-502.
6. Stuart T, Butler A, Hoffman P, Hafemeister C, Papalexi E, Mauck WM, III, et al. Comprehensive Integration of Single-Cell Data. *Cell.* 2019;177(7):1888-902.e21.
7. Hao Y, Stuart T, Kowalski MH, Choudhary S, Hoffman P, Hartman A, et al. Dictionary learning for integrative, multimodal and scalable single-cell analysis. *Nature Biotechnology.* 2024;42(2):293-304.

**Conflict of Interest**

ASN has a patent pending (PCT/US 24/25164) for the use of GHR and IGF-1R inhibitors for mitigating risk of kidney allograft and kidney failure filed through the University of Michigan. ASN also reports consulting agreements with Vera therapeutics. SE reports grants and contracts through the University of Michigan with AstraZeneca, NovoNordisk, Eli Lilly, Boehringer Ingelheim, Moderna, Certa, Chinook, Angion, Travere, Regeneron, IONIS, Maze, Sanofi, Dimerix, Roche-Genentech and Vera Therapeutics, and royalties from Astra Zeneca administered through the University of Michigan. SE has a patent for gene fusion and gene variant identification in oncology assigned to Life Technologies Corporation (now ThermoFisher). MKR reports grants and contracts through the University of Michigan with Chan Zuckerberg Initiative, Breakthrough T1D, Alport Foundation, amfAR, AstraZeneca, NovoNordisk, Eli Lilly, Gilead, Janssen, Boehringer Ingelheim, Moderna, European Union Innovative Medicine Initiative, Certa, Chinook, Angion, RenalytixAI, Travere, Regeneron, IONIS, Maze, Sanofi, Dimerix, Roche-Genentech and Vera Therapeutics; consulting fees through the University of Michigan from Janssen, NovoNordisk, Otsuka, Alexion, Variant Bio and Novartis. MKR served on the NIH-NCATS council, is the committee chair for the American Society of Nephrology Program and is on the board of Nephcure Kidney International. In addition, MKR and VN have licensed patent PCT/EP2014/073413 “Biomarkers and methods for progression prediction for chronic kidney disease.” JAS reports consulting fees from NovoNordisk, Klick media. FB reports research consulting fees from MAKScientific (Boston, Massachusetts, USA). PB reports grants and contracts through the University of Washington Breakthrough T1D, Novo Nordisk and American Diabetes Association. PB reports serving or having served as a consultant for AstraZeneca, Bayer, Bristol-Myers Squibb, Boehringer Ingelheim, Eli-Lilly, LG Chemistry, Sanofi, Novo Nordisk, and Horizon Pharma. PB also serves or has served on the advisory boards and/or steering committees of AstraZeneca, Bayer, Boehringer Ingelheim, Novo Nordisk, Eli-Lilly and XORTX. PB serves as DMC Chair for Bayer. PB reports trial funding from AstraZeneca, Novo Nordisk, Eli Lilly, Boehringer-Ingelheim, Merck, and Horizon Pharma/Amgen. PB serves as an associate editor of Journal of American Society of Nephrology, and board member of European Diabetic Nephropathy Study Group. DVR reports consultancy fees from: Astra Zeneca; Bayer; Boehringer Ingelheim, Eli Lilly; Merck, Novo Nordisk. DVR received research funding from Astra Zeneca; Boehringer Ingelheim, Eli Lilly; Merck, Novo Nordisk. DVR serves as deputy editor of Journal of European Journal of Endocrinology. MGC has received consulting fees from NovoNordisk. LP serves as statistical editor for the American Society of Nephrology journals, Diabetes Care, and the Journal of Clinical Endocrinology and Metabolism. THI declares the following consulting relationships: Wolters Kluwer, Teleflex, Medtronic, Eli Lilly, Hologic. KLT reports grants and contracts through the University of Washington with the American Diabetes Association. JRR receives a donation of drug and placebo for a clinical trial from Boehringer Ingelheim, receives research funding from Eli Lilly and Recordati, and serves on the advisory board for Calorify.

**Supplementary Table S1. Comparisons of clinical characteristics before and after surgery of IMPROVE participants without biopsy or with only one biopsy**

| | Baseline (N=11) | 12 Months Post Surgery (N=11) | Difference (N=11) | p value |
|---|---|---|---|---|
| **Sex[a]** | | | | NaN1 |
| Female | 7 (63.6%) | 7 (63.6%) | 0 (0.0%) | |
| Male | 4 (36.4%) | 4 (36.4%) | 0 (0.0%) | |
| **Age[a] (years)** | | | | < 0.0012 |
| Mean (SD) | 16.725 (1.861) | 18.099 (1.740) | 1.375 (0.367) | |
| Range | 14.020 - 19.070 | 15.370 - 20.150 | 1.060 - 2.300 | |
| **Race[a]** | | | | NaN1 |
| Black or African American | 1 (9.1%) | 1 (9.1%) | 0 (0.0%) | |
| More Than One | 1 (9.1%) | 1 (9.1%) | 0 (0.0%) | |
| Other | 1 (9.1%) | 1 (9.1%) | 0 (0.0%) | |
| White | 8 (72.7%) | 8 (72.7%) | 0 (0.0%) | |
| **Ethnicity** | | | | NaN1 |
| Hispanic or Latino | 8 (72.7%) | 8 (72.7%) | 0 (0.0%) | |
| Not Hispanic or Latino | 3 (27.3%) | 3 (27.3%) | 0 (0.0%) | |
| **eGFR by CKD-EPI Creatinine 2021** | | | | 0.7812 |
| N-Miss | 0 | 5 | 5 | |
| Mean (SD) | 135.055 (14.591) | 130.020 (11.182) | 0.929 (7.745) | |
| Range | 95.372 - 151.895 | 110.671 - 144.751 | -22.446 | |
| **Weight (kg)** | | | | < 0.0012 |
| Mean (SD) | 138.645 (23.107) | 109.518 (17.038) | -29.127 (19.343) | |
| Range | 104.200 - 190.000 | 85.800 - 139.400 | -73.8 | |
| **Body Mass Index (kg/m^2)** | | | | < 0.0012 |
| Mean (SD) | 48.728 (9.657) | 38.449 (7.388) | -10.279 (6.863) | |
| Range | 39.123 - 69.789 | 28.635 - 53.758 | -25.769 | |
| **Body fat percentage (BOD POD) (%)** | | | | 0.0022 |
| N-Miss | 1 | 4 | 4 | |
| Mean (SD) | 50.570 (5.432) | 39.271 (5.291) | -10.243 (5.294) | |
| Range | 38.800 - 55.600 | 33.200 - 48.600 | -13.3 | |
| **GFR from plasma clearance** | | | | 0.8492 |
| N-Miss | 2 | 5 | 5 | |
| Mean (SD) | 290.411 (77.076) | 243.683 (55.222) | -6.317 (77.214) | |
| Range | 160.100 - 387.900 | 167.700 - 325.800 | -190.5 | |
| **BSA indexed GFR from plasma clearance (mL/min/1.73m^2)** | | | | 0.4772 |
| N-Miss | 2 | 5 | 5 | |
| Mean (SD) | 193.689 (54.442) | 182.867 (33.363) | 15.200 (48.452) | |
| Range | 93.200 - 267.400 | 139.500 - 242.200 | -121.3 | |
| **Filtration fraction** | | | | 0.3232 |

| | | | | |
|---|---|---|---|---|
| N-Miss | 3 | 8 | 8 | |
| Mean (SD) | 0.291 (0.095) | 0.300 (0.055) | 0.036 (0.048) | |
| Range | 0.127 - 0.412 | 0.263 - 0.364 | 0.002 - 0.092 | |
| **Urine Albumin-Creatinine Ratio** | | | | 0.0783 |
| N-Miss | 0 | 4 | 4 | |
| Median | 13.378 | 7.288 | -7.85 | |
| Q1, Q3 | 8.527, 38.201 | 4.762, 83.399 | -95.306, -1.720 | |
| Range | 1.228 - 474.510 | 3.248 - 299.390 | -190.922 | |
| **Fasting Insulin** | | | | 0.0084 |
| N-Miss | 0 | 1 | 1 | |
| Median | 13 | 6.75 | -12.5 | |
| Q1, Q3 | 9.500, 37.750 | 3.125, 9.500 | -32.500, -3.750 | |
| Range | 6.000 - 79.000 | 1.500 - 15.500 | -75 | |
| **diabetes** | | | | NaN1 |
| 0 | 0 (0.0%) | 0 (0.0%) | 0 (NaN%) | |
| 1 | 11 (100.0%) | 11 (100.0%) | 0 (0.0%) | |
| **Albuminuria Category** | | | | NaN1 |
| N-Miss | 0 | 4 | 4 | |
| A1 | 7 (63.6%) | 4 (57.1%) | 0 (0.0%) | |
| A2 | 2 (18.2%) | 3 (42.9%) | 0 (0.0%) | |
| A3 | 2 (18.2%) | 0 (0.0%) | 2 (100.0%) | |
| **Elevated Albuminuria** | | | | NaN1 |
| N-Miss | 0 | 4 | 4 | |
| No | 7 (63.6%) | 4 (57.1%) | 0 (0.0%) | |
| Yes | 4 (36.4%) | 3 (42.9%) | 0 (0.0%) | |
| **Mean Arterial Pressure (MAP)** | | | | 0.0242 |
| Mean (SD) | 91.819 (7.803) | 84.787 (8.112) | -7.032 (8.767) | |
| Range | 82.000 - 106.670 | 70.330 - 99.670 | -26.67 | |
| **Systolic Blood Pressure (mmHg)** | | | | 0.0352 |
| Mean (SD) | 126.364 (10.230) | 118.909 (7.049) | -7.455 (10.113) | |
| Range | 118.000 - 152.000 | 107.000 - 131.000 | -38 | |
| **Diastolic Blood Pressure (mmHg)** | | | | 0.0532 |
| Mean (SD) | 74.545 (7.581) | 67.727 (9.799) | -6.818 (10.284) | |
| Range | 64.000 - 88.000 | 52.000 - 84.000 | -34 | |
| **Fasting Blood Glucose (mg/dL)** | | | | 0.0042 |
| N-Miss | 0 | 1 | 1 | |
| Mean (SD) | 138.955 (55.880) | 92.650 (17.952) | -33.200 (27.530) | |
| Range | 91.500 - 270.000 | 76.500 - 140.000 | -79 | |
| **HbA1C (%)** | | | | 0.0162 |
| Mean (SD) | 7.027 (1.453) | 6.045 (0.716) | -0.982 (1.120) | |
| Range | 5.600 - 9.900 | 5.300 - 7.400 | -3.5 | |
| **Acute insulin response to glucose** | | | | 0.0842 |
| N-Miss | 1 | 5 | 5 | |
| Mean (SD) | 83.522 (82.596) | 396.800 (354.231) | 291.000 (330.978) | |

| | | | | |
|---|---|---|---|---|
| Range | 0.010 - 217.200 | 82.200 - 1050.000 | -914.4 | |
| **Acute C-peptide response to glucose** | | | | 0.1042 |
| N-Miss | 1 | 5 | 5 | |
| Mean (SD) | 1.423 (1.311) | 4.628 (3.315) | 2.688 (3.315) | |
| Range | 0.010 - 3.490 | 2.230 - 11.160 | -9.25 | |
| **Raw M-value** | | | | 0.0552 |
| N-Miss | 1 | 5 | 5 | |
| Mean (SD) | 3.703 (2.449) | 8.613 (2.601) | 4.220 (4.149) | |
| Range | 0.030 - 7.752 | 6.227 - 13.139 | 0.416 - 10.891 | |
| **m_i** | | | | 0.1342 |
| N-Miss | 1 | 5 | 5 | |
| Mean (SD) | 0.062 (0.040) | 0.134 (0.116) | 0.069 (0.095) | |
| Range | 0.012 - 0.132 | 0.047 - 0.360 | -0.291 | |
| **volume_left_comb** | | | | 0.7462 |
| N-Miss | 3 | 6 | 6 | |
| Mean (SD) | 208.457 (51.252) | 185.607 (58.174) | -6.751 (43.403) | |
| Range | 135.290 - 291.439 | 134.164 - 278.809 | -109.046 | |
| **volume_right_comb** | | | | 0.0202 |
| N-Miss | 2 | 5 | 5 | |
| Mean (SD) | 204.798 (54.475) | 162.681 (48.377) | -34.857 (25.393) | |
| Range | 138.311 - 314.149 | 127.339 - 254.225 | -68.912 | |
| **tkv_comb** | | | | 0.0022 |
| N-Miss | 2 | 6 | 6 | |
| Mean (SD) | 390.093 (86.221) | 310.554 (83.622) | -64.128 (18.632) | |
| Range | 273.601 - 515.371 | 254.225 - 456.993 | -47.444 | |

[a]Self-reported.1. McNemar’s Chi-squared test; 2. Paired t-test; 3. Wilcoxon signed rank exact test; 4. Wilcoxon signed rank test with continuity correction

**Supplementary Table S2. Differentially expressed genes Post vs. Pre in the PT**

| gene | logFC_post_pre | P.Value_post_pre | adj.P.Value_post_pre |
|---|---|---|---|
| TXNIP | -0.251445373 | 9.2128E-131 | 3.7772E-127 |
| MDK | -0.155093054 | 2.506E-130 | 5.1372E-127 |
| HIST1H4C | -0.250681713 | 7.8639E-115 | 1.0747E-111 |
| G0S2 | -0.22969026 | 7.948E-113 | 8.1467E-110 |
| MPC2 | -0.257750644 | 3.9239E-107 | 3.2176E-104 |
| KRT19 | -0.174292152 | 1.3246E-100 | 9.0512E-98 |
| CXCL14 | -0.388725063 | 8.48327E-98 | 4.96877E-95 |
| WFDC2 | -0.216718854 | 1.58291E-94 | 8.11239E-92 |
| DEFB1 | -0.277769384 | 5.13902E-90 | 2.34111E-87 |
| CLU | -0.29196735 | 7.41927E-89 | 3.0419E-86 |
| CTSD | -0.214652549 | 1.76933E-86 | 6.59476E-84 |
| H3F3B | -0.250931456 | 4.41398E-83 | 1.50811E-80 |
| IGFBP7 | -0.362855101 | 9.22787E-80 | 2.91033E-77 |
| PPDPF | -0.249088357 | 2.08569E-74 | 6.10808E-72 |
| H3F3A | -0.243264895 | 7.32845E-74 | 2.00311E-71 |
| HLA-B | -0.187837095 | 1.58993E-73 | 4.0742E-71 |
| SPP1 | -0.315854352 | 2.40571E-72 | 5.80199E-70 |
| HLA-C | -0.163769048 | 8.7094E-72 | 1.98381E-69 |
| CD24 | -0.247642585 | 1.21209E-65 | 2.61556E-63 |
| ISCU | -0.18484132 | 1.03297E-62 | 2.11759E-60 |
| CAPG | -0.094936972 | 1.37128E-60 | 2.67727E-58 |
| BSG | -0.169786128 | 1.71001E-60 | 3.18683E-58 |
| PDE4D | 0.176353766 | 3.92931E-60 | 7.00442E-58 |
| KRT7 | -0.102825004 | 1.13511E-59 | 1.93914E-57 |
| HLA-A | -0.207087316 | 3.5543E-59 | 5.82906E-57 |
| CLDN7 | -0.093459891 | 4.54652E-59 | 7.16952E-57 |
| B2M | -0.300044599 | 1.39333E-54 | 2.1158E-52 |
| VEGFB | -0.104191691 | 4.27911E-53 | 6.26584E-51 |
| BHMT | -0.241246182 | 5.23046E-53 | 7.39479E-51 |
| PPIB | -0.128480698 | 1.45673E-51 | 1.99087E-49 |
| PTH2R | 0.129358607 | 2.68835E-51 | 3.55556E-49 |
| ODC1 | -0.113645605 | 2.97676E-50 | 3.81397E-48 |
| RBP4 | -0.182229039 | 8.93468E-50 | 1.11007E-47 |
| CD81 | -0.128926724 | 4.93997E-49 | 5.95702E-47 |
| SPINT2 | -0.149883242 | 5.23094E-49 | 6.12767E-47 |
| CALR | -0.12460089 | 7.33698E-48 | 8.356E-46 |
| FLNA | -0.068376624 | 1.93097E-47 | 2.13973E-45 |
| PCK1 | -0.243219137 | 6.41387E-47 | 6.92023E-45 |
| CTSL | -0.105154462 | 2.73234E-46 | 2.87246E-44 |
| MLEC | -0.086782247 | 3.61378E-46 | 3.70412E-44 |
| PERP | -0.071291841 | 9.89108E-46 | 9.89108E-44 |
| KRT8 | -0.104428754 | 1.30609E-44 | 1.27499E-42 |
| TACSTD2 | -0.128467091 | 3.23564E-44 | 3.08515E-42 |
| ALB | 0.245636673 | 3.36709E-44 | 3.13751E-42 |

| | | | |
|---|---|---|---|
| GAPDH | -0.267231903 | 8.02506E-44 | 7.31172E-42 |
| MAL | -0.108368655 | 1.19092E-43 | 1.06147E-41 |
| FABP1 | -0.301671574 | 1.33895E-43 | 1.16802E-41 |
| OCIAD2 | -0.189186347 | 2.19984E-43 | 1.87903E-41 |
| TSC22D1 | -0.100189115 | 7.9756E-43 | 6.67346E-41 |
| AFP | 0.125443922 | 1.42922E-42 | 1.17196E-40 |
| PROC | -0.050728167 | 1.26813E-41 | 1.01948E-39 |
| ATRAID | -0.097123948 | 1.44219E-41 | 1.13711E-39 |
| LRPAP1 | -0.091641889 | 1.87229E-41 | 1.44838E-39 |
| SAT1 | -0.194169049 | 3.42275E-41 | 2.59876E-39 |
| EPCAM | -0.115245353 | 3.75764E-41 | 2.80115E-39 |
| LAMB1 | -0.066071988 | 3.86526E-41 | 2.82992E-39 |
| SMIM24 | -0.172031133 | 8.43529E-41 | 5.98256E-39 |
| NPC2 | -0.11580983 | 8.46314E-41 | 5.98256E-39 |
| PDIA6 | -0.080625775 | 1.5041E-40 | 1.04522E-38 |
| LAMTOR1 | -0.106063666 | 1.81921E-40 | 1.24313E-38 |
| RPS4X | -0.256976297 | 2.22713E-40 | 1.49692E-38 |
| ARF1 | -0.098300157 | 2.63086E-40 | 1.73976E-38 |
| RPL3 | -0.27460109 | 4.64508E-40 | 3.02299E-38 |
| DAD1 | -0.123552801 | 1.33013E-39 | 8.52116E-38 |
| CYSTM1 | -0.155643529 | 1.66985E-39 | 1.05329E-37 |
| DUSP15 | -0.047867717 | 1.88524E-39 | 1.17113E-37 |
| ATP6V0E1 | -0.127213426 | 4.03885E-39 | 2.47154E-37 |
| OST4 | -0.164431243 | 6.35524E-39 | 3.83184E-37 |
| TIMP1 | -0.057906553 | 9.83406E-39 | 5.84343E-37 |
| RORA | 0.097845068 | 1.45864E-38 | 8.54345E-37 |
| IFITM3 | -0.090224942 | 1.5937E-38 | 9.20304E-37 |
| UBE2M | -0.071303186 | 1.09611E-37 | 6.24174E-36 |
| BTG1 | -0.08949794 | 1.59564E-37 | 8.96184E-36 |
| MDH1 | -0.135318251 | 2.79236E-37 | 1.54712E-35 |
| MCCD1 | -0.083586055 | 4.78744E-37 | 2.61713E-35 |
| PTPRF | -0.047543645 | 6.46544E-37 | 3.48794E-35 |
| CYS1 | -0.086491084 | 8.50253E-37 | 4.52732E-35 |
| ACTG1 | -0.183751944 | 9.43328E-37 | 4.95852E-35 |
| SLC6A13 | -0.112841243 | 1.74415E-36 | 9.05191E-35 |
| ITGB1 | -0.076570958 | 3.35018E-36 | 1.71697E-34 |
| CCNI | -0.122913369 | 5.55324E-36 | 2.8109E-34 |
| GRN | -0.083310909 | 1.85788E-35 | 9.28938E-34 |
| PTH1R | -0.156496943 | 2.56081E-35 | 1.26498E-33 |
| TMEM176 | -0.142366606 | 3.98039E-35 | 1.94281E-33 |
| CTSH | -0.108355067 | 4.14891E-35 | 2.00124E-33 |
| ATP6V1F | -0.14487122 | 1.09167E-34 | 5.20446E-33 |
| ADGRG1 | -0.064377371 | 3.16558E-34 | 1.49183E-32 |
| HMGB2 | -0.047556868 | 6.80017E-34 | 3.16826E-32 |
| NDUFA4 | -0.195023958 | 1.19462E-33 | 5.50329E-32 |
| CTSC | -0.123698553 | 1.29186E-33 | 5.88516E-32 |
| NACA | -0.215460482 | 3.05656E-33 | 1.37713E-31 |

| | | | |
|---|---|---|---|
| ITM2B | -0.268066968 | 3.45925E-33 | 1.54162E-31 |
| ESRRG | 0.06681631 | 3.5217E-33 | 1.55258E-31 |
| PARD3 | 0.068454526 | 9.1165E-33 | 3.97635E-31 |
| TMED9 | -0.050922337 | 1.37944E-32 | 5.95337E-31 |
| YBX1 | -0.129547634 | 3.14754E-32 | 1.34426E-30 |
| TMED4 | -0.080339311 | 3.57536E-32 | 1.51124E-30 |
| SLC3A2 | -0.103574104 | 7.02107E-32 | 2.93738E-30 |
| SEC11C | -0.053977349 | 7.78426E-32 | 3.22378E-30 |
| ACAA2 | -0.122524698 | 1.15903E-31 | 4.75203E-30 |
| CPVL | -0.066020526 | 7.00936E-31 | 2.84538E-29 |
| SDHD | -0.114404265 | 7.29204E-31 | 2.93111E-29 |
| PLCL1 | 0.070473104 | 9.91609E-31 | 3.94718E-29 |
| PEBP1 | -0.203554239 | 1.34577E-30 | 5.30542E-29 |
| CFI | -0.074048917 | 1.8911E-30 | 7.38428E-29 |
| NUCKS1 | -0.099306171 | 3.34625E-30 | 1.2943E-28 |
| COMT | -0.05437254 | 3.77516E-30 | 1.44656E-28 |
| RPL15 | -0.200995256 | 4.4562E-30 | 1.69171E-28 |
| PGRMC1 | -0.102431646 | 6.06983E-30 | 2.28315E-28 |
| TMEM38B | -0.043784238 | 1.4224E-29 | 5.30167E-28 |
| RPL14 | -0.199873657 | 1.49142E-29 | 5.50885E-28 |
| GLRX5 | -0.097792242 | 2.00775E-29 | 7.3498E-28 |
| HNRNPA1 | -0.127852338 | 2.13016E-29 | 7.72889E-28 |
| LDHA | -0.088941835 | 2.52647E-29 | 9.03354E-28 |
| EEF1A1 | -0.279198654 | 2.5338E-29 | 9.03354E-28 |
| CYB5R3 | -0.064912188 | 3.11976E-29 | 1.10267E-27 |
| SOD3 | -0.065281054 | 4.42969E-29 | 1.55229E-27 |
| KDELR1 | -0.075428266 | 4.98067E-29 | 1.73057E-27 |
| BEX3 | -0.112677096 | 1.1502E-28 | 3.96287E-27 |
| KCNK5 | -0.05090937 | 2.26597E-28 | 7.74206E-27 |
| CRYAB | -0.207029763 | 2.62777E-28 | 8.90401E-27 |
| APLP2 | -0.10987709 | 3.23846E-28 | 1.08833E-26 |
| MGST3 | -0.141082578 | 3.3272E-28 | 1.10907E-26 |
| COX7C | -0.209625446 | 3.95584E-28 | 1.30798E-26 |
| MAGED2 | -0.063209717 | 4.28897E-28 | 1.40678E-26 |
| TSPAN33 | -0.074306153 | 8.79052E-28 | 2.86041E-26 |
| RPL30 | -0.2212757 | 9.43391E-28 | 3.04559E-26 |
| PKHD1 | 0.110191823 | 1.3213E-27 | 4.23227E-26 |
| C4orf3 | -0.121503703 | 1.41953E-27 | 4.5117E-26 |
| TPM1 | -0.133323645 | 1.62625E-27 | 5.12896E-26 |
| NDFIP1 | -0.065706652 | 1.83965E-27 | 5.75767E-26 |
| MAGI1 | 0.042846643 | 4.12087E-27 | 1.27997E-25 |
| LEPROT | -0.084858011 | 5.03085E-27 | 1.55086E-25 |
| GPRC5C | -0.054668853 | 5.798E-27 | 1.77401E-25 |
| ZBTB20 | 0.127016348 | 6.31272E-27 | 1.9172E-25 |
| DYNLT1 | -0.103119273 | 6.89053E-27 | 2.07729E-25 |
| REEP5 | -0.064544222 | 7.21083E-27 | 2.15799E-25 |
| SEC61B | -0.114244544 | 8.93431E-27 | 2.6544E-25 |

| | | | |
|---|---|---|---|
| HLA-E | -0.053665831 | 9.125E-27 | 2.69155E-25 |
| WWOX | 0.051620739 | 1.17581E-26 | 3.44343E-25 |
| CLDN4 | -0.065966131 | 1.37657E-26 | 4.00278E-25 |
| PRDX6 | -0.116172796 | 1.79955E-26 | 5.19589E-25 |
| TMEM35B | -0.042833665 | 2.07851E-26 | 5.95935E-25 |
| MGLL | -0.056426585 | 2.54129E-26 | 7.2356E-25 |
| XIST | -0.078313787 | 3.08267E-26 | 8.71651E-25 |
| STAT1 | -0.041424465 | 3.21681E-26 | 9.0335E-25 |
| SAP18 | -0.120194877 | 3.69919E-26 | 1.03175E-24 |
| C11orf54 | 0.176318133 | 4.54574E-26 | 1.25929E-24 |
| AIF1L | -0.075524542 | 5.76911E-26 | 1.58747E-24 |
| ANP32E | -0.040094022 | 1.11471E-25 | 3.04686E-24 |
| RAN | -0.078177318 | 1.28993E-25 | 3.50246E-24 |
| GUK1 | -0.107336134 | 1.45199E-25 | 3.91656E-24 |
| FHIT | 0.072268649 | 1.8126E-25 | 4.8573E-24 |
| SPCS1 | -0.091292875 | 2.29617E-25 | 6.11319E-24 |
| ATP6V0C | -0.110897365 | 2.58397E-25 | 6.83502E-24 |
| COX6A1 | -0.129380635 | 2.65334E-25 | 6.96713E-24 |
| DSG2 | -0.036243698 | 2.6679E-25 | 6.96713E-24 |
| ATP1B1 | -0.161422084 | 3.1337E-25 | 8.13175E-24 |
| NENF | -0.08167132 | 3.74088E-25 | 9.64629E-24 |
| PTTG1IP | -0.053716477 | 4.21594E-25 | 1.08033E-23 |
| CAMK2N1 | -0.102407283 | 4.89264E-25 | 1.24595E-23 |
| EMC7 | -0.043551205 | 5.68668E-25 | 1.43922E-23 |
| SRSF9 | -0.085452962 | 8.14389E-25 | 2.04846E-23 |
| ATP6V0D1 | -0.06639572 | 1.12345E-24 | 2.80862E-23 |
| PRAP1 | -0.1405024 | 1.56836E-24 | 3.89714E-23 |
| CYBA | -0.115261449 | 1.78408E-24 | 4.40646E-23 |
| CLPTM1L | -0.057289473 | 1.80165E-24 | 4.4232E-23 |
| TSPAN1 | -0.103157619 | 2.13403E-24 | 5.20805E-23 |
| SDC4 | -0.070304281 | 2.50615E-24 | 6.08E-23 |
| UQCRFS1 | -0.097689847 | 2.69365E-24 | 6.49646E-23 |
| MYL6 | -0.157298523 | 2.81104E-24 | 6.73993E-23 |
| PTMA | -0.177046788 | 2.99365E-24 | 7.13604E-23 |
| CD63 | -0.132880311 | 3.28863E-24 | 7.79387E-23 |
| RNF7 | -0.076364738 | 3.40947E-24 | 8.03382E-23 |
| OXR1 | 0.066806115 | 3.49706E-24 | 8.19311E-23 |
| TMEM14C | -0.080965151 | 4.15125E-24 | 9.67054E-23 |
| TINAGL1 | -0.059952313 | 4.38354E-24 | 1.0154E-22 |
| GNG5 | -0.107487226 | 4.96203E-24 | 1.14294E-22 |
| FTH1 | -0.306487121 | 5.54843E-24 | 1.27087E-22 |
| CBR1 | -0.076932143 | 6.43666E-24 | 1.46613E-22 |
| TM7SF2 | -0.045112872 | 9.91647E-24 | 2.24627E-22 |
| S100A1 | -0.058418336 | 1.17583E-23 | 2.64885E-22 |
| NEDD4L | 0.071285348 | 1.20998E-23 | 2.71088E-22 |
| MARCKS | -0.049058281 | 1.40748E-23 | 3.13624E-22 |
| HSP90AB1 | -0.113185105 | 1.50851E-23 | 3.34318E-22 |

| | | | |
|---|---|---|---|
| SPCS2 | -0.066799363 | 1.68586E-23 | 3.71615E-22 |
| PLEKHA5 | 0.087484514 | 2.47784E-23 | 5.4327E-22 |
| TMEM258 | -0.09011466 | 3.59958E-23 | 7.85016E-22 |
| GNAS | -0.094716601 | 3.66237E-23 | 7.94482E-22 |
| TMEM59 | -0.097680616 | 4.26969E-23 | 9.21354E-22 |
| DBI | -0.13651076 | 5.11281E-23 | 1.09751E-21 |
| BEX2 | -0.064006499 | 5.34056E-23 | 1.14043E-21 |
| METTL9 | -0.084036673 | 5.64204E-23 | 1.19857E-21 |
| SRP14 | -0.141587211 | 6.72489E-23 | 1.42124E-21 |
| PRXL2A | -0.06626947 | 7.77513E-23 | 1.63477E-21 |
| FAM200B | -0.065328364 | 1.10509E-22 | 2.31167E-21 |
| FOLR1 | -0.058456547 | 1.18363E-22 | 2.46339E-21 |
| SELENOS | -0.040099091 | 1.21865E-22 | 2.52348E-21 |
| BRK1 | -0.079040631 | 1.37296E-22 | 2.82872E-21 |
| TMEM179 | -0.041878715 | 1.59184E-22 | 3.26327E-21 |
| GCSH | -0.113248143 | 1.76688E-22 | 3.60409E-21 |
| CDH16 | -0.061806327 | 1.87417E-22 | 3.80401E-21 |
| ARL6IP5 | -0.047092764 | 1.93957E-22 | 3.91737E-21 |
| SEC62 | -0.081526823 | 2.23953E-22 | 4.50101E-21 |
| FRMD3 | 0.037780755 | 2.73171E-22 | 5.46342E-21 |
| TMSB10 | -0.200493196 | 2.97262E-22 | 5.91637E-21 |
| PRCP | -0.038218453 | 3.4347E-22 | 6.80304E-21 |
| YWHAB | -0.081582527 | 3.59894E-22 | 7.09407E-21 |
| NDUFA13 | -0.133010373 | 3.67212E-22 | 7.20369E-21 |
| DYNLL1 | -0.116815891 | 6.04449E-22 | 1.18012E-20 |
| PCSK1N | -0.136734666 | 6.08149E-22 | 1.18171E-20 |
| STK19 | -0.035682692 | 6.75124E-22 | 1.30566E-20 |
| METRNL | -0.033238367 | 7.39456E-22 | 1.42337E-20 |
| TMEM14B | -0.073428598 | 1.00937E-21 | 1.93385E-20 |
| GLYAT | 0.122288591 | 1.13918E-21 | 2.1724E-20 |
| SLC2A9 | 0.039722748 | 1.16443E-21 | 2.21026E-20 |
| BHMT2 | -0.125414045 | 1.48946E-21 | 2.81419E-20 |
| FAHD1 | -0.070578306 | 1.58369E-21 | 2.97849E-20 |
| ATP6V1G1 | -0.100497605 | 1.70456E-21 | 3.19118E-20 |
| HIGD1A | -0.061670126 | 1.86119E-21 | 3.46858E-20 |
| LRMDA | 0.057759123 | 1.95739E-21 | 3.63136E-20 |
| GADD45A | -0.070135403 | 2.26113E-21 | 4.17597E-20 |
| CES2 | -0.060521408 | 2.43294E-21 | 4.47312E-20 |
| HMGN2 | -0.090561772 | 2.54574E-21 | 4.65962E-20 |
| PTPRM | 0.045765174 | 3.65482E-21 | 6.6599E-20 |
| CCPG1 | -0.040322614 | 3.81356E-21 | 6.9184E-20 |
| P4HB | -0.054893995 | 4.20494E-21 | 7.59482E-20 |
| NBDY | -0.054349427 | 5.38059E-21 | 9.67562E-20 |
| S100A13 | -0.081521307 | 5.75181E-21 | 1.0298E-19 |
| BEX4 | -0.060325587 | 5.87649E-21 | 1.04755E-19 |
| IER3IP1 | -0.068151312 | 6.27011E-21 | 1.11288E-19 |
| PTPRG | 0.048539684 | 6.42618E-21 | 1.13566E-19 |

| | | | |
|---|---|---|---|
| MIDN | -0.033983486 | 6.7232E-21 | 1.18305E-19 |
| HMGB1 | -0.104704263 | 7.10169E-21 | 1.24431E-19 |
| SLC35F6 | -0.030513974 | 7.88723E-21 | 1.37607E-19 |
| TMA7 | -0.123322262 | 8.59938E-21 | 1.49396E-19 |
| PPP1R14B | -0.060774202 | 8.77578E-21 | 1.51817E-19 |
| RAB2A | -0.070175725 | 9.3434E-21 | 1.60958E-19 |
| NGRN | -0.047289394 | 1.04542E-20 | 1.7934E-19 |
| RAB5IF | -0.051707276 | 1.14199E-20 | 1.9509E-19 |
| NDUFA12 | -0.067365677 | 1.24441E-20 | 2.11705E-19 |
| BTF3 | -0.112898618 | 1.30915E-20 | 2.21799E-19 |
| GINM1 | -0.055416521 | 1.32462E-20 | 2.23496E-19 |
| APP | -0.078729536 | 1.46515E-20 | 2.46193E-19 |
| AMACR | -0.070847453 | 1.67712E-20 | 2.80366E-19 |
| GLIS3 | 0.044193012 | 1.68219E-20 | 2.80366E-19 |
| RCN2 | -0.036420115 | 2.1546E-20 | 3.57646E-19 |
| S100A16 | -0.046739003 | 2.17647E-20 | 3.5982E-19 |
| RPS13 | -0.169270617 | 4.40914E-20 | 7.26003E-19 |
| CYCS | -0.066907739 | 4.53974E-20 | 7.44517E-19 |
| CMBL | -0.125579917 | 5.41725E-20 | 8.81741E-19 |
| OSTC | -0.054924489 | 5.41948E-20 | 8.81741E-19 |
| VKORC1 | -0.053616393 | 7.31357E-20 | 1.1852E-18 |
| ATP6V0B | -0.064632583 | 7.77337E-20 | 1.25358E-18 |
| LMAN1 | -0.055191371 | 7.79668E-20 | 1.25358E-18 |
| PPIC | -0.030245447 | 1.06453E-19 | 1.70491E-18 |
| SPINT1 | -0.037660044 | 1.11299E-19 | 1.77559E-18 |
| ASAH1 | -0.074237427 | 1.14789E-19 | 1.82417E-18 |
| UBE2V1 | -0.052587069 | 1.2791E-19 | 2.02483E-18 |
| BCAM | -0.070733076 | 1.46212E-19 | 2.30566E-18 |
| MET | -0.041862587 | 1.5697E-19 | 2.46582E-18 |
| CREG1 | -0.0457835 | 1.58574E-19 | 2.4815E-18 |
| COX7A2L | -0.086197145 | 1.60306E-19 | 2.49907E-18 |
| DAPK1 | 0.034158972 | 1.87961E-19 | 2.91909E-18 |
| CALM3 | -0.073564337 | 1.96605E-19 | 3.04182E-18 |
| HSP90B1 | -0.089776293 | 2.07631E-19 | 3.20032E-18 |
| SERINC2 | -0.035610824 | 2.73551E-19 | 4.20059E-18 |
| TP53I13 | -0.03143898 | 3.08362E-19 | 4.71748E-18 |
| EIF5A | -0.074818026 | 3.53773E-19 | 5.39208E-18 |
| NCL | -0.064217074 | 4.86327E-19 | 7.38496E-18 |
| TMCO1 | -0.053244137 | 5.07106E-19 | 7.66745E-18 |
| HNF4G | 0.038367521 | 5.0867E-19 | 7.66745E-18 |
| MSRB2 | -0.078038491 | 5.1528E-19 | 7.73864E-18 |
| TMEM205 | -0.058902665 | 5.18263E-19 | 7.75504E-18 |
| HMGN1 | -0.086631099 | 5.72858E-19 | 8.54079E-18 |
| TMEM176 | -0.097198892 | 6.31235E-19 | 9.37705E-18 |
| NDUFB5 | -0.078245035 | 6.51448E-19 | 9.64238E-18 |
| SMCO4 | -0.030470922 | 6.81994E-19 | 1.00582E-17 |
| ANAPC16 | -0.080890797 | 7.3026E-19 | 1.07314E-17 |

| | | | |
|---|---|---|---|
| SELENOF | -0.048061119 | 9.34097E-19 | 1.36779E-17 |
| SRP9 | -0.082775879 | 1.16615E-18 | 1.7015E-17 |
| ATP6V0E2 | -0.059758661 | 1.21357E-18 | 1.76442E-17 |
| CHID1 | -0.038136008 | 1.53737E-18 | 2.22729E-17 |
| SPINT1-AS | -0.031666567 | 1.55512E-18 | 2.24506E-17 |
| TSTD1 | -0.082411955 | 1.59819E-18 | 2.29914E-17 |
| TUSC3 | -0.029353043 | 1.76543E-18 | 2.53086E-17 |
| NME3 | -0.061615283 | 1.81542E-18 | 2.59346E-17 |
| LAPTM4B | -0.033127821 | 2.19883E-18 | 3.13028E-17 |
| RPL41 | -0.217076635 | 2.27122E-18 | 3.22215E-17 |
| TOMM6 | -0.083637256 | 2.31753E-18 | 3.27651E-17 |
| NDUFA1 | -0.114740671 | 2.34755E-18 | 3.30754E-17 |
| NDUFB4 | -0.093598907 | 2.6587E-18 | 3.73311E-17 |
| GNG11 | -0.035203222 | 2.72148E-18 | 3.80821E-17 |
| MAML2 | 0.05841905 | 2.74887E-18 | 3.83346E-17 |
| NDUFAB1 | -0.08917662 | 2.94119E-18 | 4.07454E-17 |
| NINJ1 | -0.060624116 | 2.94162E-18 | 4.07454E-17 |
| SEC11A | -0.06768971 | 3.7507E-18 | 5.17773E-17 |
| TEX264 | -0.04476433 | 3.89498E-18 | 5.35886E-17 |
| IMMP2L | 0.053423266 | 4.25369E-18 | 5.83282E-17 |
| SDSL | -0.04538983 | 4.88829E-18 | 6.68067E-17 |
| DHRS7 | -0.036184155 | 5.3376E-18 | 7.27048E-17 |
| SLC43A2 | -0.046477576 | 5.99188E-18 | 8.13467E-17 |
| MORN2 | -0.066165226 | 6.34944E-18 | 8.59166E-17 |
| HEXB | -0.030461274 | 7.3445E-18 | 9.90542E-17 |
| IMPA2 | -0.088015136 | 7.40903E-18 | 9.95968E-17 |
| EID1 | -0.07354577 | 8.31613E-18 | 1.11425E-16 |
| ERRFI1 | 0.035567988 | 9.12897E-18 | 1.21918E-16 |
| TSPAN6 | -0.031036132 | 9.62858E-18 | 1.28173E-16 |
| B4GALT1 | -0.037598871 | 1.03307E-17 | 1.37074E-16 |
| SNRPN | -0.067249584 | 1.18669E-17 | 1.5695E-16 |
| PTPRD | 0.05382582 | 1.19354E-17 | 1.57348E-16 |
| RER1 | -0.043785923 | 1.49267E-17 | 1.96152E-16 |
| CALML3 | -0.057263259 | 1.54191E-17 | 2.01976E-16 |
| TMEM243 | -0.033479853 | 1.58974E-17 | 2.07578E-16 |
| SLC39A1 | -0.027982142 | 2.01741E-17 | 2.62583E-16 |
| MICOS10 | -0.094583456 | 2.07021E-17 | 2.68603E-16 |
| C7orf50 | -0.058990658 | 2.22589E-17 | 2.87891E-16 |
| CPE | -0.052384583 | 2.34853E-17 | 3.02798E-16 |
| GSTP1 | -0.128508226 | 2.39347E-17 | 3.07625E-16 |
| USP2 | -0.046574056 | 2.42709E-17 | 3.10971E-16 |
| SET | -0.071178637 | 2.57199E-17 | 3.2851E-16 |
| TRPM3 | 0.049045657 | 3.28269E-17 | 4.17982E-16 |
| SLC38A2 | 0.038986414 | 3.2948E-17 | 4.18226E-16 |
| KANK1 | 0.032883453 | 4.34178E-17 | 5.49423E-16 |
| TTC28 | 0.03660991 | 4.50366E-17 | 5.68154E-16 |
| LSM4 | -0.066274734 | 4.74449E-17 | 5.967E-16 |

| | | | |
|---|---|---|---|
| SDF2L1 | -0.034452081 | 5.10806E-17 | 6.4046E-16 |
| MYDGF | -0.036044657 | 5.36468E-17 | 6.70586E-16 |
| AP2S1 | -0.065652014 | 5.39254E-17 | 6.72019E-16 |
| SRSF7 | -0.04414403 | 5.85159E-17 | 7.27015E-16 |
| MRPL34 | -0.075954631 | 6.24604E-17 | 7.73679E-16 |
| TOMM5 | -0.079743465 | 6.49697E-17 | 8.02337E-16 |
| CFDP1 | 0.093595511 | 6.7433E-17 | 8.30256E-16 |
| LMAN2 | -0.043637813 | 6.98221E-17 | 8.57097E-16 |
| PFDN2 | -0.055908947 | 7.14212E-17 | 8.74111E-16 |
| RPS10 | -0.127160873 | 7.75962E-17 | 9.46858E-16 |
| BANF1 | -0.052801978 | 8.43615E-17 | 1.02636E-15 |
| SSR2 | -0.056527698 | 8.5836E-17 | 1.04121E-15 |
| TNFSF10 | -0.088312486 | 1.08294E-16 | 1.30975E-15 |
| TMED10 | -0.041490645 | 1.20185E-16 | 1.44928E-15 |
| PROM1 | -0.046563902 | 1.39386E-16 | 1.6759E-15 |
| NEU1 | -0.033894632 | 1.41493E-16 | 1.69626E-15 |
| KLF10 | -0.029351949 | 1.43117E-16 | 1.71073E-15 |
| UQCR11 | -0.118318699 | 1.44146E-16 | 1.71802E-15 |
| KTN1 | -0.067791959 | 1.46823E-16 | 1.74485E-15 |
| WDFY3 | 0.045594341 | 1.48512E-16 | 1.75982E-15 |
| RTN4 | -0.08372348 | 1.50036E-16 | 1.77277E-15 |
| HMGN3 | -0.089522214 | 1.53464E-16 | 1.80805E-15 |
| GGACT | 0.057242665 | 1.69038E-16 | 1.98583E-15 |
| TP53I3 | -0.035600421 | 1.88724E-16 | 2.21077E-15 |
| ATP5MC3 | -0.125231172 | 1.91483E-16 | 2.2308E-15 |
| TMBIM4 | -0.052196209 | 1.91522E-16 | 2.2308E-15 |
| SARAF | -0.063141338 | 1.96629E-16 | 2.2838E-15 |
| TTC36 | 0.045576346 | 1.98775E-16 | 2.30219E-15 |
| ZNF667-AS | -0.027454899 | 1.99336E-16 | 2.30219E-15 |
| COX6C | -0.11477016 | 2.00313E-16 | 2.30698E-15 |
| RPL5 | -0.141040088 | 2.07356E-16 | 2.3814E-15 |
| C11orf58 | -0.059448114 | 2.15298E-16 | 2.46571E-15 |
| CST3 | -0.09333439 | 2.20811E-16 | 2.52179E-15 |
| SDF2 | -0.029915849 | 2.51262E-16 | 2.8616E-15 |
| RAPGEF2 | 0.029554965 | 2.58604E-16 | 2.93705E-15 |
| KRT10 | -0.064684096 | 2.72317E-16 | 3.08425E-15 |
| PYURF | -0.05247389 | 3.01577E-16 | 3.40624E-15 |
| NCOA2 | 0.031277726 | 3.32282E-16 | 3.74274E-15 |
| DDX5 | -0.06908324 | 3.46464E-16 | 3.89178E-15 |
| MZT2B | -0.09173426 | 3.68362E-16 | 4.12646E-15 |
| RBFOX2 | 0.03016834 | 4.11788E-16 | 4.60035E-15 |
| RGN | -0.056608881 | 4.42124E-16 | 4.92584E-15 |
| ARSD | -0.030665603 | 4.81524E-16 | 5.35026E-15 |
| POLR2H | -0.041895613 | 5.40025E-16 | 5.98406E-15 |
| RRBP1 | -0.035629773 | 5.817E-16 | 6.42849E-15 |
| GPC6 | 0.0392053 | 6.30239E-16 | 6.94618E-15 |
| TIMM8B | -0.070244737 | 6.49669E-16 | 7.14114E-15 |

| | | | |
|---|---|---|---|
| DMKN | -0.034898643 | 6.52851E-16 | 7.15635E-15 |
| CLN5 | -0.031109097 | 6.54966E-16 | 7.15635E-15 |
| SOX4 | -0.063532648 | 6.56289E-16 | 7.15635E-15 |
| SERPINA1 | 0.087031206 | 6.70482E-16 | 7.29172E-15 |
| MAL2 | -0.025538574 | 7.47111E-16 | 8.10358E-15 |
| TMEM230 | -0.058669657 | 8.2889E-16 | 8.96688E-15 |
| PGAM1 | -0.051350208 | 9.05829E-16 | 9.77342E-15 |
| COPS9 | -0.083636423 | 9.33349E-16 | 1.00439E-14 |
| COL4A2 | -0.049246225 | 9.54561E-16 | 1.02453E-14 |
| PON2 | -0.026342611 | 9.5771E-16 | 1.02523E-14 |
| ATP6AP2 | -0.045857192 | 9.76174E-16 | 1.04227E-14 |
| MAF | 0.108871975 | 1.0273E-15 | 1.09401E-14 |
| TSC22D3 | -0.031576167 | 1.0363E-15 | 1.10073E-14 |
| SH3BGRL | -0.046633235 | 1.12096E-15 | 1.18758E-14 |
| TM2D2 | -0.027785594 | 1.15338E-15 | 1.21878E-14 |
| SSR4 | -0.063370394 | 1.16411E-15 | 1.22696E-14 |
| CA12 | -0.05993256 | 1.17108E-15 | 1.23114E-14 |
| CFL1 | -0.088278693 | 1.20723E-15 | 1.26589E-14 |
| ANK3 | 0.06388374 | 1.49366E-15 | 1.56225E-14 |
| IER5L | -0.027987217 | 1.74002E-15 | 1.81529E-14 |
| PLLP | -0.026095817 | 1.854E-15 | 1.92929E-14 |
| CNP | -0.034122944 | 1.93453E-15 | 2.00799E-14 |
| COMMD6 | -0.087013132 | 2.2972E-15 | 2.37842E-14 |
| TCTN1 | -0.026661417 | 2.32036E-15 | 2.39635E-14 |
| UQCRH | -0.102868948 | 2.42364E-15 | 2.49672E-14 |
| PKM | -0.050632549 | 2.47706E-15 | 2.54475E-14 |
| TXNL4A | -0.049017013 | 2.48469E-15 | 2.54475E-14 |
| PLAAT4 | -0.048273167 | 2.48889E-15 | 2.54475E-14 |
| VAMP2 | -0.046189291 | 2.51963E-15 | 2.56977E-14 |
| GTF3C6 | -0.055206273 | 2.74907E-15 | 2.79682E-14 |
| PDCD6 | -0.046859043 | 2.94261E-15 | 2.98632E-14 |
| CALM2 | -0.073308278 | 3.07672E-15 | 3.1147E-14 |
| APOM | -0.075626119 | 3.33914E-15 | 3.37204E-14 |
| ARL1 | -0.043836851 | 3.90628E-15 | 3.93508E-14 |
| PHB | -0.082970008 | 4.25489E-15 | 4.27575E-14 |
| PDIA3 | -0.047964183 | 4.32836E-15 | 4.33894E-14 |
| CNN3 | -0.061677732 | 4.35068E-15 | 4.35068E-14 |
| CPEB4 | 0.055679807 | 4.57612E-15 | 4.56499E-14 |
| CD46 | -0.051644777 | 4.72303E-15 | 4.7001E-14 |
| PLEKHA7 | 0.034045394 | 4.81495E-15 | 4.77997E-14 |
| PTPRK | 0.032392551 | 5.21273E-15 | 5.16236E-14 |
| PAH | 0.114186959 | 6.98023E-15 | 6.89613E-14 |
| ARPC3 | -0.069568758 | 7.08063E-15 | 6.97666E-14 |
| NDUFA5 | -0.070947658 | 7.09577E-15 | 6.97666E-14 |
| S100A6 | -0.111138895 | 7.45913E-15 | 7.31637E-14 |
| COMMD8 | -0.045733253 | 7.6697E-15 | 7.50495E-14 |
| TGOLN2 | -0.049699163 | 8.41483E-15 | 8.21448E-14 |

| | | | |
|---|---|---|---|
| ID4 | -0.046899206 | 8.80678E-15 | 8.57668E-14 |
| ACSM2A | 0.095072955 | 8.92865E-15 | 8.67476E-14 |
| GPHN | 0.045171976 | 9.03381E-15 | 8.75618E-14 |
| VCP | -0.043256165 | 9.23678E-15 | 8.93179E-14 |
| OAZ1 | -0.101155438 | 9.93944E-15 | 9.58864E-14 |
| METRN | -0.046097662 | 1.00022E-14 | 9.62652E-14 |
| HNRNPH1 | -0.070403912 | 1.20005E-14 | 1.15227E-13 |
| SLC22A2 | -0.070418863 | 1.27351E-14 | 1.21995E-13 |
| NDUFB9 | -0.067138893 | 1.35149E-14 | 1.29163E-13 |
| RPL4 | -0.089392329 | 1.37098E-14 | 1.30722E-13 |
| PCBD1 | -0.088007152 | 1.5049E-14 | 1.43157E-13 |
| EXOC4 | 0.031135595 | 1.6139E-14 | 1.53171E-13 |
| NDUFB2 | -0.11354213 | 1.82461E-14 | 1.72769E-13 |
| RPL19 | -0.155742919 | 1.86158E-14 | 1.75864E-13 |
| MRFAP1 | -0.054468831 | 1.99552E-14 | 1.88083E-13 |
| SLC25A39 | -0.059769001 | 2.00602E-14 | 1.8864E-13 |
| BHLHE40 | -0.0322615 | 2.31651E-14 | 2.17338E-13 |
| BNC2 | 0.046097854 | 2.34594E-14 | 2.19597E-13 |
| C1orf210 | -0.026495501 | 2.36426E-14 | 2.20808E-13 |
| NPM1 | -0.090582797 | 2.39731E-14 | 2.23222E-13 |
| PRDX4 | -0.024679597 | 2.401E-14 | 2.23222E-13 |
| ITGB1BP1 | -0.028264897 | 2.66424E-14 | 2.46868E-13 |
| EDF1 | -0.102427464 | 2.66738E-14 | 2.46868E-13 |
| DPM3 | -0.047660195 | 2.82636E-14 | 2.60993E-13 |
| APH1A | -0.032956599 | 2.87507E-14 | 2.64894E-13 |
| MFSD10 | -0.025054015 | 2.95336E-14 | 2.71497E-13 |
| ITM2C | -0.037713406 | 2.96644E-14 | 2.7209E-13 |
| ACADVL | -0.064947835 | 3.15911E-14 | 2.89115E-13 |
| RPS8 | -0.14818281 | 3.583E-14 | 3.27179E-13 |
| RPS18 | -0.169550944 | 3.74909E-14 | 3.41584E-13 |
| COX7A2 | -0.090454627 | 4.21515E-14 | 3.83196E-13 |
| EMC10 | -0.03618498 | 4.5153E-14 | 4.09574E-13 |
| C9orf16 | -0.042771382 | 4.61027E-14 | 4.17265E-13 |
| PSMA7 | -0.066506924 | 5.15966E-14 | 4.65317E-13 |
| TXNDC17 | -0.081630632 | 5.16388E-14 | 4.65317E-13 |
| DDOST | -0.029748909 | 5.19434E-14 | 4.67035E-13 |
| TMEM147 | -0.038719565 | 5.22775E-14 | 4.6901E-13 |
| APOC2 | -0.034597908 | 5.60257E-14 | 5.0154E-13 |
| BCAP31 | -0.046994588 | 5.74988E-14 | 5.13606E-13 |
| UQCRB | -0.109797382 | 6.76936E-14 | 6.03356E-13 |
| SNF8 | -0.045034279 | 7.13177E-14 | 6.34279E-13 |
| NME1 | -0.037347033 | 7.40521E-14 | 6.57172E-13 |
| SSB | -0.041087328 | 7.48792E-14 | 6.63077E-13 |
| MESD | -0.024713712 | 7.52252E-14 | 6.64706E-13 |
| SMIM7 | -0.044327477 | 7.5561E-14 | 6.66237E-13 |
| SEM1 | -0.066279881 | 7.74977E-14 | 6.81847E-13 |
| OGDHL | -0.053036062 | 9.1147E-14 | 8.0022E-13 |

| | | | |
|---|---|---|---|
| PPP3CA | 0.028712669 | 1.01082E-13 | 8.85547E-13 |
| MORF4L1 | -0.07305734 | 1.01484E-13 | 8.8717E-13 |
| PSAP | -0.071516387 | 1.0947E-13 | 9.54951E-13 |
| CD74 | -0.068113783 | 1.10429E-13 | 9.61274E-13 |
| IFT57 | -0.03661643 | 1.18157E-13 | 1.02503E-12 |
| COPRS | -0.042024881 | 1.18254E-13 | 1.02503E-12 |
| H2AFJ | -0.049994889 | 1.26511E-13 | 1.0943E-12 |
| PPM1G | -0.031170364 | 1.27096E-13 | 1.09704E-12 |
| NR2F6 | -0.035181563 | 1.27851E-13 | 1.10123E-12 |
| SNX3 | -0.063923094 | 1.38754E-13 | 1.19264E-12 |
| ANXA2 | -0.041806696 | 1.39723E-13 | 1.19846E-12 |
| H2AFY | -0.032718252 | 1.42457E-13 | 1.21936E-12 |
| COX20 | -0.058243554 | 1.48203E-13 | 1.2659E-12 |
| SSR3 | -0.043658729 | 1.58508E-13 | 1.35111E-12 |
| FUCA2 | -0.032609112 | 1.73883E-13 | 1.47909E-12 |
| EHBP1 | 0.026088767 | 1.74831E-13 | 1.48408E-12 |
| UNC50 | -0.028250999 | 1.76821E-13 | 1.49786E-12 |
| SELENOH | -0.069494016 | 1.81394E-13 | 1.53343E-12 |
| HLA-DRA | -0.042337216 | 1.97968E-13 | 1.6701E-12 |
| NDUFC2 | -0.074950579 | 2.04248E-13 | 1.71954E-12 |
| PKLR | -0.037917208 | 2.30339E-13 | 1.93523E-12 |
| REXO2 | -0.061329461 | 2.30873E-13 | 1.93575E-12 |
| BORCS7 | -0.042336545 | 2.3875E-13 | 1.9977E-12 |
| BLOC1S2 | -0.025998015 | 2.49806E-13 | 2.08596E-12 |
| DTD1 | -0.027688852 | 2.52394E-13 | 2.10328E-12 |
| CENPX | -0.046118483 | 2.54587E-13 | 2.11725E-12 |
| TRIM8 | -0.038174764 | 2.56801E-13 | 2.13134E-12 |
| LGALS3BP | -0.029292876 | 2.7196E-13 | 2.2526E-12 |
| KIAA1958 | 0.028162775 | 2.80292E-13 | 2.31693E-12 |
| MAST4 | 0.044297994 | 2.85935E-13 | 2.35882E-12 |
| NKAIN4 | -0.032546082 | 3.16764E-13 | 2.60789E-12 |
| UQCR10 | -0.10226474 | 3.22733E-13 | 2.65171E-12 |
| COMTD1 | 0.033222714 | 3.40746E-13 | 2.79412E-12 |
| CTSB | -0.087641168 | 3.92748E-13 | 3.21411E-12 |
| CETN2 | -0.041639013 | 3.94371E-13 | 3.22096E-12 |
| COX5B | -0.10540154 | 4.28084E-13 | 3.48935E-12 |
| DSTN | -0.089215701 | 4.41797E-13 | 3.59399E-12 |
| TSPAN3 | -0.038918599 | 4.46584E-13 | 3.62573E-12 |
| CLIC1 | -0.066233878 | 4.71539E-13 | 3.82077E-12 |
| MGAT4B | -0.03436662 | 5.762E-13 | 4.6596E-12 |
| AC100810. | -0.043472935 | 5.92098E-13 | 4.77874E-12 |
| MRPS26 | -0.049659996 | 6.12357E-13 | 4.93254E-12 |
| AIG1 | -0.051405731 | 6.18316E-13 | 4.97077E-12 |
| ORMDL1 | -0.027356274 | 6.76422E-13 | 5.42726E-12 |
| WBP1 | -0.032945328 | 6.91334E-13 | 5.53608E-12 |
| PICALM | 0.050591357 | 7.33947E-13 | 5.86586E-12 |
| WASF2 | -0.03091501 | 8.10074E-13 | 6.46168E-12 |

| | | | |
|---|---|---|---|
| CITED2 | -0.045877468 | 8.23535E-13 | 6.5563E-12 |
| PIGR | -0.073156735 | 8.87233E-13 | 7.04972E-12 |
| SLC17A1 | 0.036403209 | 9.32441E-13 | 7.3946E-12 |
| CD9 | -0.043874323 | 9.82876E-13 | 7.77952E-12 |
| CIAO2B | -0.066553136 | 1.037E-12 | 8.19209E-12 |
| TUBA1B | -0.038941772 | 1.19808E-12 | 9.44639E-12 |
| TM9SF1 | -0.027491957 | 1.21962E-12 | 9.5978E-12 |
| EMC3 | -0.024917612 | 1.27153E-12 | 9.98709E-12 |
| TMEM9 | -0.03120689 | 1.29078E-12 | 1.01189E-11 |
| PRODH2 | 0.108591978 | 1.30133E-12 | 1.01822E-11 |
| PKP4 | 0.039804984 | 1.3605E-12 | 1.06248E-11 |
| DYNC2LI1 | -0.034599547 | 1.47776E-12 | 1.15187E-11 |
| CNPY2 | -0.041590985 | 1.55462E-12 | 1.20948E-11 |
| RPL23A | -0.107315425 | 1.56733E-12 | 1.21705E-11 |
| RPS6 | -0.13591006 | 1.71942E-12 | 1.33263E-11 |
| RAB1B | -0.038284497 | 1.79434E-12 | 1.38808E-11 |
| ARF4 | -0.039510008 | 1.88066E-12 | 1.45211E-11 |
| GTF2A2 | -0.039856831 | 1.90648E-12 | 1.46928E-11 |
| ATP6V1D | -0.046797848 | 1.9167E-12 | 1.47438E-11 |
| CYB5A | -0.143161185 | 1.93554E-12 | 1.48609E-11 |
| ERP44 | -0.023342041 | 2.06925E-12 | 1.58578E-11 |
| KCNJ16 | -0.049629171 | 2.5172E-12 | 1.92547E-11 |
| VWA8 | 0.026380879 | 2.98971E-12 | 2.28265E-11 |
| LAPTM4A | -0.098005203 | 3.06657E-12 | 2.33697E-11 |
| C18orf32 | -0.056417492 | 3.1755E-12 | 2.4155E-11 |
| AKAP12 | -0.031119492 | 3.25337E-12 | 2.47015E-11 |
| RWDD1 | -0.038568858 | 3.62617E-12 | 2.74812E-11 |
| EIF3H | -0.052426843 | 3.77843E-12 | 2.85822E-11 |
| SDF4 | -0.030221743 | 3.86233E-12 | 2.91631E-11 |
| NDUFC1 | -0.09868039 | 4.07609E-12 | 3.07206E-11 |
| HINT1 | -0.11241869 | 4.17299E-12 | 3.13931E-11 |
| LRP10 | -0.027071767 | 4.32648E-12 | 3.24882E-11 |
| ARL16 | -0.032209359 | 4.35661E-12 | 3.26547E-11 |
| S100A10 | -0.083962237 | 4.44747E-12 | 3.32749E-11 |
| MRPL33 | -0.059841309 | 4.50634E-12 | 3.36539E-11 |
| FAM162A | -0.054658688 | 4.6611E-12 | 3.47464E-11 |
| WDR72 | 0.059771703 | 4.74103E-12 | 3.52781E-11 |
| RPL7A | -0.133958701 | 4.9115E-12 | 3.64804E-11 |
| PTMS | -0.085040889 | 5.01934E-12 | 3.72139E-11 |
| RPS3A | -0.139136331 | 5.05697E-12 | 3.74252E-11 |
| EMC4 | -0.026685149 | 5.19976E-12 | 3.84127E-11 |
| FTCD | 0.083266351 | 5.24789E-12 | 3.86985E-11 |
| UGT3A1 | -0.038889708 | 5.83925E-12 | 4.29819E-11 |
| COTL1 | -0.088273531 | 5.93257E-12 | 4.35905E-11 |
| ACTB | -0.099878836 | 6.05747E-12 | 4.44287E-11 |
| ARHGAP1 | -0.023381471 | 6.1613E-12 | 4.51095E-11 |
| RBM3 | -0.040949161 | 6.34399E-12 | 4.63643E-11 |

| | | | |
|---|---|---|---|
| ABCB1 | -0.037471836 | 6.51089E-12 | 4.74994E-11 |
| NDUFB3 | -0.070171542 | 6.97868E-12 | 5.08216E-11 |
| KRT18 | -0.058739714 | 8.13868E-12 | 5.91641E-11 |
| TMEM208 | -0.038367491 | 8.96286E-12 | 6.50402E-11 |
| SLC25A5 | -0.10127556 | 9.12075E-12 | 6.6069E-11 |
| SNHG29 | -0.106434746 | 9.51262E-12 | 6.87861E-11 |
| RPL6 | -0.113557211 | 9.94963E-12 | 7.18195E-11 |
| ALDH1A1 | -0.055967063 | 9.96757E-12 | 7.18225E-11 |
| RALBP1 | -0.029408044 | 1.01167E-11 | 7.27694E-11 |
| TCF12 | 0.027940606 | 1.04009E-11 | 7.45314E-11 |
| SAR1A | -0.032383292 | 1.04071E-11 | 7.45314E-11 |
| FTX | 0.036153209 | 1.04162E-11 | 7.45314E-11 |
| UBXN4 | -0.037993602 | 1.06932E-11 | 7.63802E-11 |
| RPS15A | -0.13602407 | 1.07204E-11 | 7.64411E-11 |
| SLC44A4 | -0.02728526 | 1.08924E-11 | 7.75327E-11 |
| TSPYL1 | -0.049322827 | 1.10773E-11 | 7.8712E-11 |
| ZFYVE21 | -0.024982308 | 1.12079E-11 | 7.95025E-11 |
| ELOC | -0.044818566 | 1.21556E-11 | 8.60761E-11 |
| RNASEK | -0.08356012 | 1.30894E-11 | 9.25287E-11 |
| RPS19 | -0.126674885 | 1.33658E-11 | 9.43195E-11 |
| COMMD2 | -0.022840388 | 1.36381E-11 | 9.60762E-11 |
| ZFAS1 | -0.053286651 | 1.49272E-11 | 1.04804E-10 |
| MYL12A | -0.062660147 | 1.49282E-11 | 1.04804E-10 |
| MBD5 | 0.026868515 | 1.50426E-11 | 1.05427E-10 |
| SNRPG | -0.067481698 | 1.56419E-11 | 1.0944E-10 |
| LSM1 | -0.027605301 | 1.60219E-11 | 1.11908E-10 |
| RPS7 | -0.128402295 | 1.77586E-11 | 1.23827E-10 |
| FAT1 | -0.042731789 | 1.79658E-11 | 1.25059E-10 |
| RSRC2 | -0.030196684 | 1.81955E-11 | 1.26443E-10 |
| TMEM50A | -0.035411675 | 1.82799E-11 | 1.26775E-10 |
| PDCD5 | -0.038481796 | 1.83051E-11 | 1.26775E-10 |
| CDIPT | -0.025057059 | 1.93531E-11 | 1.33808E-10 |
| TOMM7 | -0.089908325 | 2.11023E-11 | 1.45656E-10 |
| ZCRB1 | -0.033793949 | 2.50941E-11 | 1.72917E-10 |
| PLAAT3 | -0.066355692 | 2.55014E-11 | 1.75429E-10 |
| LRRC19 | -0.034952339 | 2.71907E-11 | 1.86737E-10 |
| TM7SF3 | -0.048707999 | 2.73378E-11 | 1.87146E-10 |
| ARHGEF28 | 0.035713382 | 2.73415E-11 | 1.87146E-10 |
| SERP1 | -0.049693132 | 2.8452E-11 | 1.94422E-10 |
| LINC01578 | -0.038307889 | 2.92099E-11 | 1.99269E-10 |
| CISD1 | -0.057395953 | 2.95456E-11 | 2.01224E-10 |
| XRCC5 | -0.040176202 | 2.96484E-11 | 2.01589E-10 |
| ATP5PF | -0.094764563 | 3.19769E-11 | 2.17062E-10 |
| PSMA5 | -0.041420631 | 3.84068E-11 | 2.60278E-10 |
| LRBA | 0.029250314 | 3.8569E-11 | 2.60945E-10 |
| SLC35F5 | -0.037002125 | 3.87059E-11 | 2.6144E-10 |
| SRI | -0.034911017 | 4.30188E-11 | 2.90094E-10 |

| | | | |
|---|---|---|---|
| FOXP1 | 0.027102142 | 4.33029E-11 | 2.9153E-10 |
| ARL8A | -0.025808724 | 4.40863E-11 | 2.96318E-10 |
| ADAM9 | -0.02192595 | 4.48967E-11 | 3.01271E-10 |
| SBDS | -0.043126051 | 5.02242E-11 | 3.3647E-10 |
| AVP | 0.03582753 | 5.20879E-11 | 3.48385E-10 |
| SLC6A8 | -0.028758708 | 5.22441E-11 | 3.48861E-10 |
| NUCB1 | -0.041063954 | 5.26429E-11 | 3.50952E-10 |
| MRPS36 | -0.059219631 | 5.29338E-11 | 3.52247E-10 |
| PARK7 | -0.073324461 | 5.30089E-11 | 3.52247E-10 |
| RASSF4 | -0.038796796 | 5.34775E-11 | 3.54786E-10 |
| SLC16A12 | 0.041699805 | 5.42944E-11 | 3.5941E-10 |
| KANSL1L | 0.031978168 | 5.43927E-11 | 3.5941E-10 |
| ARRDC3 | -0.047853975 | 5.44375E-11 | 3.5941E-10 |
| SCARB2 | -0.030054846 | 5.72679E-11 | 3.7749E-10 |
| KDELR2 | -0.036423856 | 5.80679E-11 | 3.82148E-10 |
| CMPK1 | -0.042575989 | 6.20032E-11 | 4.07393E-10 |
| GSTK1 | -0.053701226 | 6.26463E-11 | 4.10959E-10 |
| OS9 | -0.028052807 | 6.54821E-11 | 4.28876E-10 |
| DYNLT3 | -0.033458345 | 7.00371E-11 | 4.57978E-10 |
| SNRPE | -0.046298062 | 7.15728E-11 | 4.67275E-10 |
| PSENEN | -0.029300565 | 7.33831E-11 | 4.78332E-10 |
| BTF3L4 | -0.031412743 | 7.90416E-11 | 5.14398E-10 |
| MRPS10 | -0.027901612 | 8.10508E-11 | 5.26637E-10 |
| TRAPPC4 | -0.032839874 | 8.54009E-11 | 5.54025E-10 |
| TFCP2L1 | -0.032476293 | 8.58777E-11 | 5.56238E-10 |
| ACSM2B | 0.078995434 | 9.03751E-11 | 5.83991E-10 |
| DNAJC15 | -0.042060567 | 9.04474E-11 | 5.83991E-10 |
| ANP32B | -0.032012612 | 9.09143E-11 | 5.86083E-10 |
| CCND1 | -0.03997608 | 1.13515E-10 | 7.30631E-10 |
| PPA1 | -0.046769876 | 1.14699E-10 | 7.37097E-10 |
| POP5 | -0.031143377 | 1.19545E-10 | 7.67035E-10 |
| CREB5 | 0.030267618 | 1.30416E-10 | 8.35476E-10 |
| KAZN | 0.024145087 | 1.37888E-10 | 8.81966E-10 |
| TNFRSF19 | -0.021830995 | 1.38268E-10 | 8.8302E-10 |
| RBIS | -0.045052437 | 1.40082E-10 | 8.93212E-10 |
| FTL | -0.290614043 | 1.42552E-10 | 9.07551E-10 |
| ATP5F1A | -0.068691746 | 1.43113E-10 | 9.09712E-10 |
| ZFP36L2 | -0.035807585 | 1.56017E-10 | 9.902E-10 |
| MRPL57 | -0.064643702 | 1.57689E-10 | 9.99265E-10 |
| ADAMTS9- | 0.047537289 | 1.62805E-10 | 1.0301E-09 |
| STAU2 | 0.023289007 | 1.72566E-10 | 1.09017E-09 |
| ATP5IF1 | -0.096419703 | 1.76969E-10 | 1.11627E-09 |
| LAMP1 | -0.047865355 | 1.80082E-10 | 1.134E-09 |
| TSPAN4 | -0.025092214 | 1.80333E-10 | 1.134E-09 |
| HLA-DRB1 | -0.044149475 | 1.81327E-10 | 1.1385E-09 |
| RPSA | -0.085986292 | 1.84452E-10 | 1.15635E-09 |
| SPX | -0.033443045 | 1.88489E-10 | 1.17986E-09 |

| | | | |
|---|---|---|---|
| PFN2 | -0.029229434 | 1.91183E-10 | 1.19489E-09 |
| GNPDA1 | -0.044691225 | 1.95557E-10 | 1.22037E-09 |
| SRSF3 | -0.048931948 | 2.01619E-10 | 1.25629E-09 |
| MRPL3 | -0.034734777 | 2.03196E-10 | 1.26419E-09 |
| GOLIM4 | -0.023145265 | 2.11374E-10 | 1.31308E-09 |
| VDAC1 | -0.061821769 | 2.29973E-10 | 1.42646E-09 |
| GOLGB1 | -0.037179214 | 2.3095E-10 | 1.42929E-09 |
| CEBPD | -0.044390515 | 2.31126E-10 | 1.42929E-09 |
| ERBB2 | -0.029879643 | 2.33259E-10 | 1.4403E-09 |
| CLINT1 | -0.035373798 | 2.36722E-10 | 1.45949E-09 |
| SMIM10L1 | -0.024784557 | 2.38449E-10 | 1.46793E-09 |
| TLE5 | -0.042160031 | 2.39592E-10 | 1.47276E-09 |
| ARG2 | -0.037604652 | 2.55852E-10 | 1.57035E-09 |
| HEBP2 | -0.03786765 | 2.65039E-10 | 1.62431E-09 |
| SNRPF | -0.043327269 | 2.68337E-10 | 1.64206E-09 |
| NOL7 | -0.038688772 | 2.68964E-10 | 1.64344E-09 |
| VDAC3 | -0.037120578 | 2.84259E-10 | 1.73432E-09 |
| SUB1 | -0.064188407 | 2.91798E-10 | 1.77767E-09 |
| C19orf53 | -0.061610313 | 2.99547E-10 | 1.82217E-09 |
| CHCHD1 | -0.044206806 | 3.29187E-10 | 1.99951E-09 |
| TBC1D5 | 0.026820261 | 3.3048E-10 | 2.00439E-09 |
| NME2 | -0.076421257 | 3.34399E-10 | 2.02516E-09 |
| COQ9 | -0.034402653 | 3.80827E-10 | 2.30294E-09 |
| FCGRT | -0.055312737 | 3.84827E-10 | 2.3237E-09 |
| STRAP | -0.038186568 | 3.95633E-10 | 2.38544E-09 |
| COX4I1 | -0.091770793 | 4.04005E-10 | 2.43233E-09 |
| FKBP4 | -0.036701507 | 4.07527E-10 | 2.44994E-09 |
| MRPL13 | -0.038165031 | 4.21564E-10 | 2.53062E-09 |
| MRPS23 | -0.034227236 | 4.23196E-10 | 2.5367E-09 |
| EBAG9 | -0.02146394 | 4.27208E-10 | 2.55701E-09 |
| ILF2 | -0.03488954 | 4.36066E-10 | 2.60623E-09 |
| VPS29 | -0.045616607 | 5.07699E-10 | 3.02994E-09 |
| RBM39 | -0.040612329 | 5.1214E-10 | 3.052E-09 |
| AGMAT | -0.084401211 | 5.51227E-10 | 3.28016E-09 |
| POMP | -0.059766855 | 5.77422E-10 | 3.43106E-09 |
| GATD3A | -0.025715061 | 6.09042E-10 | 3.61371E-09 |
| PTGES3 | -0.055338291 | 6.24378E-10 | 3.69935E-09 |
| TAPBP | -0.02580665 | 6.48495E-10 | 3.8367E-09 |
| CIRBP | -0.061379549 | 6.55281E-10 | 3.87126E-09 |
| SLC25A11 | -0.040493664 | 6.76088E-10 | 3.98843E-09 |
| RAB13 | -0.035201523 | 7.02549E-10 | 4.13858E-09 |
| RPL11 | -0.129419312 | 7.48831E-10 | 4.40489E-09 |
| HNRNPA0 | -0.028197106 | 7.59655E-10 | 4.46216E-09 |
| CISD2 | -0.030962042 | 7.6954E-10 | 4.51375E-09 |
| UBTF | -0.022878203 | 7.70779E-10 | 4.51456E-09 |
| CXXC5 | -0.036210012 | 7.83646E-10 | 4.58338E-09 |
| TMX4 | -0.027586643 | 8.5229E-10 | 4.97776E-09 |

| | | | |
|---|---|---|---|
| SLCO4C1 | 0.032973077 | 8.67411E-10 | 5.05887E-09 |
| PPCS | -0.040741542 | 8.71022E-10 | 5.07271E-09 |
| SELENOT | -0.032321445 | 8.84401E-10 | 5.14332E-09 |
| JUND | -0.055860153 | 9.83591E-10 | 5.71207E-09 |
| RPS27A | -0.132855091 | 1.00536E-09 | 5.83021E-09 |
| DUS1L | -0.030184606 | 1.0109E-09 | 5.8541E-09 |
| XBP1 | -0.026040909 | 1.02033E-09 | 5.90036E-09 |
| PRKCSH | -0.02895128 | 1.05514E-09 | 6.08969E-09 |
| NUDCD2 | -0.030286066 | 1.05604E-09 | 6.08969E-09 |
| CLDN3 | -0.048862085 | 1.07947E-09 | 6.21607E-09 |
| SOD1 | -0.083610285 | 1.15829E-09 | 6.66059E-09 |
| LGALS3 | -0.031426723 | 1.2134E-09 | 6.96771E-09 |
| COMMD3 | -0.024539278 | 1.22267E-09 | 7.00486E-09 |
| SMIM15 | -0.026836877 | 1.22329E-09 | 7.00486E-09 |
| PARL | -0.025169892 | 1.23672E-09 | 7.07191E-09 |
| GTF3A | -0.036112685 | 1.26613E-09 | 7.22997E-09 |
| STOM | -0.035795229 | 1.27499E-09 | 7.27044E-09 |
| UQCRQ | -0.093165973 | 1.33482E-09 | 7.60103E-09 |
| RWDD4 | -0.023319483 | 1.37782E-09 | 7.83503E-09 |
| KRTCAP2 | -0.042115587 | 1.3917E-09 | 7.903E-09 |
| GGCT | -0.030552663 | 1.41483E-09 | 8.02322E-09 |
| NPL | -0.045100455 | 1.43835E-09 | 8.14535E-09 |
| IFT20 | -0.024144578 | 1.45333E-09 | 8.21886E-09 |
| FBXO22 | -0.021391119 | 1.57216E-09 | 8.87858E-09 |
| TMSB4X | -0.060147713 | 1.69686E-09 | 9.56962E-09 |
| SDC2 | -0.030667741 | 1.7536E-09 | 9.87607E-09 |
| CALML4 | -0.046498197 | 1.82309E-09 | 1.02533E-08 |
| PIK3CB | 0.024850686 | 1.82933E-09 | 1.02743E-08 |
| ILVBL | -0.02454365 | 1.92828E-09 | 1.08153E-08 |
| TNRC6A | 0.029645195 | 2.06494E-09 | 1.15659E-08 |
| RAB34 | -0.02197159 | 2.13319E-09 | 1.19319E-08 |
| RABGAP1L | 0.023263344 | 2.19412E-09 | 1.2256E-08 |
| METTL23 | -0.026974433 | 2.21256E-09 | 1.23422E-08 |
| SLIRP | -0.058796159 | 2.30478E-09 | 1.28391E-08 |
| RPS3 | -0.107339107 | 2.4227E-09 | 1.34777E-08 |
| CSRP2 | -0.031708547 | 2.44862E-09 | 1.36035E-08 |
| CD59 | -0.050288036 | 2.46081E-09 | 1.36527E-08 |
| RPL17 | -0.109543995 | 2.46835E-09 | 1.3676E-08 |
| FGGY | 0.026513871 | 2.50354E-09 | 1.38522E-08 |
| PSMD6 | -0.023745538 | 2.58656E-09 | 1.42923E-08 |
| PSMB8 | -0.019966512 | 2.76527E-09 | 1.52592E-08 |
| SGCB | -0.026507368 | 2.77612E-09 | 1.52985E-08 |
| SULT1C2 | -0.02755216 | 2.85546E-09 | 1.57146E-08 |
| GNG10 | -0.034383514 | 2.90308E-09 | 1.59553E-08 |
| RPL26 | -0.129481223 | 3.01971E-09 | 1.6574E-08 |
| SDHC | -0.048906054 | 3.04089E-09 | 1.6668E-08 |
| CCDC115 | -0.021604768 | 3.05507E-09 | 1.67233E-08 |

| | | | |
|---|---|---|---|
| RPLP0 | -0.080256386 | 3.23677E-09 | 1.76944E-08 |
| DNAJC19 | -0.039624092 | 3.25382E-09 | 1.77639E-08 |
| FKBP1A | -0.03922214 | 3.35394E-09 | 1.82861E-08 |
| SURF1 | -0.038622067 | 3.50892E-09 | 1.91057E-08 |
| SCAMP2 | -0.02733218 | 3.60459E-09 | 1.96005E-08 |
| ATP5MC1 | -0.061279373 | 3.97643E-09 | 2.15939E-08 |
| HNRNPA2I | -0.056285005 | 4.06211E-09 | 2.20106E-08 |
| TM9SF3 | -0.03311786 | 4.0639E-09 | 2.20106E-08 |
| DNAJC3 | -0.02322518 | 4.17681E-09 | 2.25923E-08 |
| YBX3 | -0.027243405 | 4.2003E-09 | 2.26894E-08 |
| ANXA5 | -0.040902378 | 4.5386E-09 | 2.44846E-08 |
| CBWD1 | -0.027843079 | 4.8506E-09 | 2.61333E-08 |
| CREBRF | 0.025572634 | 5.30268E-09 | 2.85315E-08 |
| GLIS2 | -0.022423604 | 5.31242E-09 | 2.85464E-08 |
| NDUFA9 | -0.028257749 | 5.32314E-09 | 2.85666E-08 |
| RSRP1 | -0.026007757 | 5.41225E-09 | 2.90068E-08 |
| FLRT3 | -0.037036659 | 5.44539E-09 | 2.91464E-08 |
| EI24 | -0.027500507 | 5.60399E-09 | 2.99561E-08 |
| TNFRSF1A | -0.022577534 | 5.74084E-09 | 3.06344E-08 |
| MIA3 | -0.019594347 | 5.74582E-09 | 3.06344E-08 |
| HSD17B12 | -0.023101881 | 5.75361E-09 | 3.06361E-08 |
| MIEN1 | -0.032779094 | 5.9379E-09 | 3.15764E-08 |
| HSPA5 | -0.036525084 | 6.50234E-09 | 3.45332E-08 |
| ARHGAP42 | 0.020936529 | 6.53861E-09 | 3.46808E-08 |
| ABL1 | 0.019599275 | 6.59847E-09 | 3.49532E-08 |
| SYAP1 | -0.030413801 | 6.6186E-09 | 3.49701E-08 |
| PDIA4 | -0.025323389 | 6.61874E-09 | 3.49701E-08 |
| AUTS2 | 0.020081816 | 6.8122E-09 | 3.5946E-08 |
| GSTA2 | -0.107493678 | 7.02322E-09 | 3.70118E-08 |
| CLIC4 | 0.061711156 | 7.34489E-09 | 3.86573E-08 |
| SRSF5 | -0.038780293 | 7.46808E-09 | 3.92553E-08 |
| LAMA5 | -0.020296974 | 7.85207E-09 | 4.12209E-08 |
| RBP5 | -0.087896767 | 8.00489E-09 | 4.19694E-08 |
| TFPI | -0.031546159 | 8.32353E-09 | 4.35843E-08 |
| TCTA | -0.02105938 | 9.10615E-09 | 4.76215E-08 |
| GET1 | -0.020920541 | 9.55091E-09 | 4.98837E-08 |
| RPL10 | -0.129897088 | 9.64056E-09 | 5.02879E-08 |
| FAM107B | -0.044413991 | 9.67382E-09 | 5.03973E-08 |
| PSMD7 | -0.022747247 | 1.04298E-08 | 5.42666E-08 |
| SPATA20 | -0.018367301 | 1.06071E-08 | 5.51193E-08 |
| SNRPD1 | -0.028576977 | 1.07671E-08 | 5.58272E-08 |
| UFC1 | -0.037975858 | 1.07706E-08 | 5.58272E-08 |
| CBX6 | -0.025835095 | 1.11308E-08 | 5.76214E-08 |
| FDX1 | -0.047117125 | 1.11725E-08 | 5.77016E-08 |
| CLTRN | 0.061790587 | 1.11744E-08 | 5.77016E-08 |
| TIMM13 | -0.053413049 | 1.12513E-08 | 5.80257E-08 |
| PTGR1 | -0.056646509 | 1.13296E-08 | 5.83562E-08 |

| | | | |
|---|---|---|---|
| GPT | 0.027756011 | 1.15814E-08 | 5.95782E-08 |
| TSEN34 | -0.025895294 | 1.17479E-08 | 6.03587E-08 |
| LINC00472 | 0.048609775 | 1.17666E-08 | 6.03794E-08 |
| HPD | -0.10217471 | 1.18503E-08 | 6.07325E-08 |
| KIF9 | -0.028512868 | 1.22233E-08 | 6.25664E-08 |
| RNASE4 | -0.022993541 | 1.22783E-08 | 6.27695E-08 |
| GNPTG | -0.020539542 | 1.24107E-08 | 6.33671E-08 |
| GTF2H5 | -0.044897386 | 1.26447E-08 | 6.44816E-08 |
| RPL9 | -0.099271745 | 1.30354E-08 | 6.63913E-08 |
| RPS23 | -0.122187042 | 1.32846E-08 | 6.75767E-08 |
| ELF3 | -0.025357541 | 1.36131E-08 | 6.91617E-08 |
| SERPINE2 | -0.022668097 | 1.36599E-08 | 6.9314E-08 |
| STAT3 | -0.022936027 | 1.37214E-08 | 6.95399E-08 |
| SMIM20 | -0.030229841 | 1.40505E-08 | 7.11196E-08 |
| CWC15 | -0.030827515 | 1.43067E-08 | 7.23272E-08 |
| PDK2 | -0.038076798 | 1.44008E-08 | 7.27135E-08 |
| MRPL32 | -0.03008182 | 1.48098E-08 | 7.46155E-08 |
| RPN2 | -0.028588397 | 1.48139E-08 | 7.46155E-08 |
| LYRM1 | -0.029651466 | 1.49707E-08 | 7.53128E-08 |
| MRPL36 | -0.030472492 | 1.50552E-08 | 7.5645E-08 |
| INSR | 0.043378225 | 1.5754E-08 | 7.90594E-08 |
| NT5C3B | -0.022300433 | 1.58685E-08 | 7.95364E-08 |
| COMMD7 | -0.03010431 | 1.60259E-08 | 8.02275E-08 |
| PNN | -0.020317164 | 1.61395E-08 | 8.06974E-08 |
| MAP1LC3A | -0.02498297 | 1.63519E-08 | 8.1612E-08 |
| HPGD | -0.039841852 | 1.63622E-08 | 8.1612E-08 |
| LINC00667 | -0.019473404 | 1.66991E-08 | 8.31914E-08 |
| EIF4G2 | -0.049098735 | 1.74124E-08 | 8.66396E-08 |
| LSM6 | -0.0306342 | 1.76694E-08 | 8.78118E-08 |
| PPIG | -0.027624794 | 1.78519E-08 | 8.8611E-08 |
| COL4A1 | -0.028094025 | 1.82512E-08 | 9.04834E-08 |
| NDUFA6 | -0.04374358 | 1.84031E-08 | 9.11264E-08 |
| SLC1A1 | 0.029910114 | 1.92849E-08 | 9.53775E-08 |
| CITED4 | -0.02609303 | 1.93546E-08 | 9.56071E-08 |
| HOXB2 | -0.018468548 | 1.94619E-08 | 9.60214E-08 |
| IFT22 | -0.031369407 | 1.95905E-08 | 9.65014E-08 |
| SF3B6 | -0.047579328 | 1.96063E-08 | 9.65014E-08 |
| VPS36 | -0.021491328 | 1.99646E-08 | 9.81471E-08 |
| NAA20 | -0.036771837 | 2.04169E-08 | 1.00251E-07 |
| COA5 | -0.032755106 | 2.04703E-08 | 1.00393E-07 |
| MPC1 | -0.047904832 | 2.0653E-08 | 1.01168E-07 |
| POU3F3 | -0.024479542 | 2.08411E-08 | 1.01967E-07 |
| FNIP2 | 0.034298746 | 2.09266E-08 | 1.02263E-07 |
| RPL26L1 | -0.029216746 | 2.20784E-08 | 1.07763E-07 |
| RPL37 | -0.108663971 | 2.23991E-08 | 1.09199E-07 |
| PLXNB2 | -0.033018348 | 2.28536E-08 | 1.11282E-07 |
| GSN | 0.02237041 | 2.33439E-08 | 1.13535E-07 |

| | | | |
|---|---|---|---|
| CDC42 | -0.051689469 | 2.34162E-08 | 1.13752E-07 |
| RPL27A | -0.083290899 | 2.35642E-08 | 1.14335E-07 |
| ABRACL | -0.035800562 | 2.41278E-08 | 1.16931E-07 |
| YWHAQ | -0.033942303 | 2.42233E-08 | 1.17256E-07 |
| SRP19 | -0.024846762 | 2.46161E-08 | 1.19017E-07 |
| ANKRD17 | 0.023310158 | 2.65305E-08 | 1.28121E-07 |
| STAG1 | 0.019944345 | 2.73177E-08 | 1.31768E-07 |
| SUMF2 | -0.027329486 | 2.79711E-08 | 1.34761E-07 |
| PRRC2C | -0.028146449 | 2.80355E-08 | 1.34912E-07 |
| SOX6 | 0.030429013 | 2.86403E-08 | 1.37662E-07 |
| ATP5PD | -0.055371255 | 2.94956E-08 | 1.41606E-07 |
| HSPE1 | -0.091813987 | 2.98864E-08 | 1.43315E-07 |
| PDZD11 | -0.019454661 | 3.24862E-08 | 1.556E-07 |
| HACD3 | -0.023993455 | 3.27803E-08 | 1.56825E-07 |
| TAF10 | -0.037882909 | 3.28725E-08 | 1.57083E-07 |
| NUTF2 | -0.025497601 | 3.29827E-08 | 1.57426E-07 |
| JMJD1C | 0.027353164 | 3.39018E-08 | 1.61625E-07 |
| LMNA | -0.029388826 | 3.49248E-08 | 1.66137E-07 |
| SDHAF3 | -0.028328762 | 3.49526E-08 | 1.66137E-07 |
| CAPN2 | -0.019287915 | 3.49697E-08 | 1.66137E-07 |
| FAM136A | -0.021916222 | 3.61575E-08 | 1.7158E-07 |
| UBE2I | -0.02673576 | 3.70129E-08 | 1.75437E-07 |
| DPM2 | -0.02006803 | 3.76596E-08 | 1.78296E-07 |
| SLC16A9 | -0.074425217 | 3.86023E-08 | 1.82549E-07 |
| ACTN4 | -0.047002473 | 4.01287E-08 | 1.89454E-07 |
| PTPMT1 | -0.021774155 | 4.0155E-08 | 1.89454E-07 |
| CCDC198 | -0.040042632 | 4.06946E-08 | 1.91779E-07 |
| NDUFS4 | -0.03850087 | 4.18697E-08 | 1.9709E-07 |
| LINC02027 | 0.021967397 | 4.19796E-08 | 1.97381E-07 |
| CFAP36 | -0.027162753 | 4.23084E-08 | 1.98699E-07 |
| TRHDE | 0.02212438 | 4.28663E-08 | 2.01089E-07 |
| PPP2R3A | 0.02286253 | 4.4336E-08 | 2.07746E-07 |
| PDGFC | 0.026256043 | 4.43982E-08 | 2.078E-07 |
| SELENOM | -0.036369926 | 4.51341E-08 | 2.11003E-07 |
| CDK14 | 0.027255259 | 4.70547E-08 | 2.19732E-07 |
| HPCAL1 | -0.028619436 | 4.76501E-08 | 2.22259E-07 |
| IDH2 | -0.036814011 | 4.80274E-08 | 2.23764E-07 |
| SMIM19 | -0.0361387 | 4.94491E-08 | 2.30127E-07 |
| SIPA1L1 | 0.022724824 | 4.98055E-08 | 2.31522E-07 |
| TMEM165 | -0.019709319 | 5.04844E-08 | 2.34412E-07 |
| RDH14 | -0.01747092 | 5.11466E-08 | 2.37218E-07 |
| EZR | -0.051050561 | 5.35282E-08 | 2.47984E-07 |
| TIMMDC1 | -0.028301886 | 5.48718E-08 | 2.53921E-07 |
| SURF4 | -0.026416473 | 5.50691E-08 | 2.54547E-07 |
| SON | -0.038675692 | 5.52828E-08 | 2.55247E-07 |
| LAMTOR2 | -0.043918557 | 5.56109E-08 | 2.56473E-07 |
| GGT6 | -0.02580089 | 5.60889E-08 | 2.58387E-07 |

| | | | |
|---|---|---|---|
| SFT2D2 | -0.022652512 | 5.85478E-08 | 2.69355E-07 |
| FTO | 0.018535977 | 5.86011E-08 | 2.69355E-07 |
| RPAIN | -0.021033162 | 5.90864E-08 | 2.71281E-07 |
| HNRNPR | -0.02475575 | 6.23757E-08 | 2.86063E-07 |
| GABARAPL | -0.046835114 | 6.35692E-08 | 2.91211E-07 |
| OCIAD1 | -0.035048846 | 6.63228E-08 | 3.03486E-07 |
| PRLR | 0.03500605 | 6.65635E-08 | 3.04248E-07 |
| PLEKHJ1 | -0.029381119 | 6.66708E-08 | 3.04399E-07 |
| FAM229B | -0.019818134 | 6.68043E-08 | 3.04509E-07 |
| BCAS3 | 0.020695568 | 6.68435E-08 | 3.04509E-07 |
| CAMTA1 | -0.052263404 | 6.88785E-08 | 3.13431E-07 |
| CSNK1A1 | -0.030985817 | 7.19313E-08 | 3.2696E-07 |
| CYC1 | -0.039088214 | 7.2958E-08 | 3.3126E-07 |
| MBNL2 | 0.027301483 | 7.40817E-08 | 3.3599E-07 |
| SEC61A1 | -0.020219102 | 7.41971E-08 | 3.36141E-07 |
| PNKD | -0.042005119 | 7.50772E-08 | 3.39753E-07 |
| TMEM141 | -0.045733633 | 7.71503E-08 | 3.4875E-07 |
| LINC00665 | -0.020012964 | 7.96635E-08 | 3.59714E-07 |
| BAX | -0.029463429 | 8.09554E-08 | 3.65145E-07 |
| QDPR | -0.058146872 | 8.1991E-08 | 3.6941E-07 |
| UBL5 | -0.074906997 | 8.25404E-08 | 3.71477E-07 |
| LAP3 | -0.036674146 | 8.48216E-08 | 3.81325E-07 |
| PDE8A | 0.019494662 | 8.51537E-08 | 3.82399E-07 |
| RASSF7 | -0.019938974 | 8.52981E-08 | 3.82628E-07 |
| SUMO2 | -0.05706924 | 8.63508E-08 | 3.86855E-07 |
| RPS27L | -0.07662376 | 8.64291E-08 | 3.86855E-07 |
| MLF2 | -0.037994808 | 8.80725E-08 | 3.93781E-07 |
| CDHR5 | -0.032490009 | 9.30176E-08 | 4.15438E-07 |
| NIT1 | -0.023970167 | 9.65444E-08 | 4.3072E-07 |
| NUDT9 | -0.017401381 | 9.72101E-08 | 4.33219E-07 |
| TMEM219 | -0.041374523 | 1.00559E-07 | 4.47658E-07 |
| DYNC1H1 | -0.030962568 | 1.02594E-07 | 4.5622E-07 |
| SERF2 | -0.099896464 | 1.0275E-07 | 4.56418E-07 |
| SNHG8 | -0.056014669 | 1.0327E-07 | 4.58234E-07 |
| PTS | -0.027392887 | 1.09068E-07 | 4.83438E-07 |
| FAM234A | -0.017852815 | 1.10376E-07 | 4.88706E-07 |
| MBD2 | -0.022185229 | 1.11033E-07 | 4.91083E-07 |
| ETNK2 | -0.037698139 | 1.11404E-07 | 4.92129E-07 |
| NCSTN | -0.017561613 | 1.11509E-07 | 4.92129E-07 |
| MRPL20 | -0.047913005 | 1.16028E-07 | 5.1152E-07 |
| CCDC107 | -0.02168938 | 1.16915E-07 | 5.14877E-07 |
| PIM3 | -0.020992444 | 1.22974E-07 | 5.40979E-07 |
| CMC2 | -0.031215294 | 1.24472E-07 | 5.46981E-07 |
| FBXO17 | -0.022661009 | 1.25268E-07 | 5.49893E-07 |
| NSMCE1 | -0.023304526 | 1.25939E-07 | 5.52245E-07 |
| FKBP3 | -0.025255446 | 1.30325E-07 | 5.70868E-07 |
| TBCB | -0.026740717 | 1.31118E-07 | 5.73727E-07 |

| | | | |
|---|---|---|---|
| TM9SF2 | -0.027220138 | 1.31601E-07 | 5.75228E-07 |
| CBX5 | -0.023312029 | 1.32038E-07 | 5.76525E-07 |
| PLGRKT | -0.020496511 | 1.33799E-07 | 5.83589E-07 |
| CRIM1 | -0.032086072 | 1.35342E-07 | 5.89237E-07 |
| TMEM263 | -0.019653341 | 1.35381E-07 | 5.89237E-07 |
| IMMT | -0.025731909 | 1.3585E-07 | 5.90654E-07 |
| HOMER3 | -0.025786983 | 1.42065E-07 | 6.17019E-07 |
| DNMT3L | -0.022482239 | 1.43059E-07 | 6.20645E-07 |
| ERGIC3 | -0.027988108 | 1.43203E-07 | 6.20645E-07 |
| DDX39B | -0.024121653 | 1.46793E-07 | 6.35535E-07 |
| MPST | 0.043971912 | 1.48691E-07 | 6.43072E-07 |
| TMEM167 | -0.031496222 | 1.50935E-07 | 6.5209E-07 |
| ALDH2 | -0.060990226 | 1.5152E-07 | 6.53931E-07 |
| BCAS2 | -0.019616372 | 1.52324E-07 | 6.56707E-07 |
| CTNNB1 | -0.035327348 | 1.53388E-07 | 6.60602E-07 |
| CDH6 | -0.033751121 | 1.54082E-07 | 6.62891E-07 |
| SEPTIN11 | -0.023257762 | 1.55078E-07 | 6.66478E-07 |
| EIF1 | -0.089539934 | 1.60117E-07 | 6.87411E-07 |
| ACY1 | 0.080326271 | 1.61005E-07 | 6.90502E-07 |
| ITGAE | -0.021661002 | 1.62778E-07 | 6.97379E-07 |
| CCDC47 | -0.019651039 | 1.663E-07 | 7.11724E-07 |
| CD151 | -0.033628979 | 1.67093E-07 | 7.14371E-07 |
| AGRN | -0.017607986 | 1.73053E-07 | 7.39079E-07 |
| CAT | -0.052844816 | 1.79511E-07 | 7.65864E-07 |
| GALNT11 | -0.043535809 | 1.80245E-07 | 7.68194E-07 |
| SMIM32 | -0.035915031 | 1.81757E-07 | 7.73836E-07 |
| MAP3K1 | 0.021869956 | 1.82652E-07 | 7.76839E-07 |
| C1orf122 | -0.035123023 | 1.83733E-07 | 7.80629E-07 |
| VAMP8 | -0.05684919 | 1.89161E-07 | 8.02139E-07 |
| MRPL17 | -0.027995946 | 1.89187E-07 | 8.02139E-07 |
| EIF4H | -0.025793127 | 1.94103E-07 | 8.22129E-07 |
| MRPS18C | -0.030077423 | 1.94744E-07 | 8.23995E-07 |
| RPS9 | -0.096136335 | 1.97969E-07 | 8.36778E-07 |
| TP53TG1 | -0.020199425 | 2.07563E-07 | 8.76423E-07 |
| EPRS | -0.017364914 | 2.07949E-07 | 8.77149E-07 |
| ACOT2 | -0.028468233 | 2.08694E-07 | 8.79389E-07 |
| ALDH4A1 | -0.056161946 | 2.13796E-07 | 8.99965E-07 |
| ATP6AP1 | -0.024288889 | 2.15586E-07 | 9.06025E-07 |
| SLC25A4 | -0.029475134 | 2.15678E-07 | 9.06025E-07 |
| BCL7C | -0.025920437 | 2.20962E-07 | 9.27273E-07 |
| TMEM106 | -0.020609423 | 2.22466E-07 | 9.3263E-07 |
| MALAT1 | -0.255592623 | 2.43142E-07 | 1.01826E-06 |
| RPL8 | -0.09657415 | 2.45734E-07 | 1.02807E-06 |
| PEF1 | -0.018237181 | 2.50173E-07 | 1.04558E-06 |
| SLC35D2 | -0.017563512 | 2.58803E-07 | 1.08054E-06 |
| ATP1A1 | -0.054737428 | 2.62421E-07 | 1.09453E-06 |
| MRPS14 | -0.020024678 | 2.66979E-07 | 1.11241E-06 |

| | | | |
|---|---|---|---|
| ALDH1L1 | 0.044530178 | 2.67528E-07 | 1.11357E-06 |
| LETM1 | -0.018528742 | 2.67979E-07 | 1.11431E-06 |
| ARL3 | -0.034331316 | 2.69413E-07 | 1.11914E-06 |
| PBDC1 | -0.01716267 | 2.75746E-07 | 1.14429E-06 |
| BICC1 | 0.049462347 | 2.78033E-07 | 1.15261E-06 |
| ABHD17A | -0.023887257 | 2.78666E-07 | 1.15407E-06 |
| SS18L2 | -0.022342728 | 2.81677E-07 | 1.16492E-06 |
| CHD4 | -0.022414887 | 2.81854E-07 | 1.16492E-06 |
| SBF2 | 0.017552003 | 2.82427E-07 | 1.16611E-06 |
| MED13L | 0.020230407 | 2.8271E-07 | 1.16611E-06 |
| PFN1 | -0.061241075 | 2.83311E-07 | 1.16741E-06 |
| BIN1 | -0.039869743 | 2.8714E-07 | 1.18165E-06 |
| HIST1H1C | -0.028960525 | 2.87343E-07 | 1.18165E-06 |
| C1orf43 | -0.038201478 | 3.05989E-07 | 1.25707E-06 |
| TMED2 | -0.026375291 | 3.11171E-07 | 1.27708E-06 |
| SPCS3 | -0.021011018 | 3.22989E-07 | 1.32387E-06 |
| GOT1 | -0.029252639 | 3.23218E-07 | 1.32387E-06 |
| AFM | 0.024685102 | 3.26767E-07 | 1.33707E-06 |
| ERP29 | -0.037979078 | 3.28391E-07 | 1.34238E-06 |
| MXD4 | -0.025707392 | 3.30547E-07 | 1.34984E-06 |
| DENND1A | 0.018950871 | 3.32282E-07 | 1.35558E-06 |
| PSMG2 | -0.028749474 | 3.34191E-07 | 1.36201E-06 |
| TERF2IP | -0.021398159 | 3.34634E-07 | 1.36246E-06 |
| HNRNPA3 | -0.032967927 | 3.52085E-07 | 1.43209E-06 |
| M6PR | -0.020798562 | 3.54853E-07 | 1.44192E-06 |
| CHP1 | -0.026992451 | 3.59248E-07 | 1.45833E-06 |
| RDH11 | -0.024998361 | 3.69717E-07 | 1.49935E-06 |
| PDCD4 | -0.017427064 | 3.81382E-07 | 1.54512E-06 |
| COMMD9 | -0.01885798 | 3.84562E-07 | 1.55647E-06 |
| PCMT1 | -0.019173772 | 3.8839E-07 | 1.57041E-06 |
| TUFM | -0.041213177 | 3.89972E-07 | 1.57525E-06 |
| JPT1 | -0.017551826 | 3.92094E-07 | 1.58227E-06 |
| JPX | -0.022558106 | 3.9615E-07 | 1.59707E-06 |
| DUT | -0.032609284 | 4.04681E-07 | 1.62985E-06 |
| TMED5 | -0.024105292 | 4.12931E-07 | 1.66145E-06 |
| HSD11B2 | -0.018476212 | 4.20639E-07 | 1.69081E-06 |
| SLC4A4 | 0.058925252 | 4.23713E-07 | 1.70149E-06 |
| MKNK2 | -0.019949742 | 4.47134E-07 | 1.79212E-06 |
| GNAI2 | -0.024869759 | 4.47155E-07 | 1.79212E-06 |
| LRRK2 | 0.028032969 | 4.496E-07 | 1.80015E-06 |
| AUP1 | -0.017099691 | 4.55465E-07 | 1.82186E-06 |
| EEF1E1 | -0.025447098 | 4.58931E-07 | 1.83393E-06 |
| DDB1 | -0.022120199 | 4.6075E-07 | 1.83941E-06 |
| MRPL51 | -0.043545054 | 4.61751E-07 | 1.84161E-06 |
| SLC28A1 | 0.025429955 | 4.63464E-07 | 1.84665E-06 |
| SEPHS1 | -0.020825295 | 4.68992E-07 | 1.86686E-06 |
| MRPS15 | -0.034373758 | 4.72668E-07 | 1.87967E-06 |

| | | | |
|---|---|---|---|
| COA6 | -0.024780771 | 4.8111E-07 | 1.91117E-06 |
| DNAJC21 | -0.020772871 | 4.81522E-07 | 1.91117E-06 |
| SNX2 | -0.028070861 | 4.89972E-07 | 1.94283E-06 |
| RPL28 | -0.095335051 | 5.00419E-07 | 1.98233E-06 |
| RAB4A | -0.024733059 | 5.20715E-07 | 2.06075E-06 |
| HDDC2 | -0.030404474 | 5.21546E-07 | 2.06204E-06 |
| RPL36AL | -0.061813713 | 5.22088E-07 | 2.0622E-06 |
| DHDH | 0.030090587 | 5.30507E-07 | 2.09343E-06 |
| RPS4Y1 | -0.040908681 | 5.37521E-07 | 2.11907E-06 |
| TPR | -0.018598029 | 5.42809E-07 | 2.13787E-06 |
| BAD | -0.029154444 | 5.51286E-07 | 2.16917E-06 |
| CTSF | -0.02003619 | 5.64133E-07 | 2.21759E-06 |
| RPL29 | -0.091933433 | 5.64759E-07 | 2.21793E-06 |
| AIFM1 | -0.036369898 | 5.69043E-07 | 2.23261E-06 |
| MRPS6 | -0.026443387 | 5.70888E-07 | 2.23771E-06 |
| DBP | -0.021319656 | 5.99279E-07 | 2.34664E-06 |
| RNF130 | -0.022662862 | 5.99825E-07 | 2.34664E-06 |
| PIK3R1 | -0.019104747 | 6.1746E-07 | 2.41333E-06 |
| MPV17 | -0.019287675 | 6.30602E-07 | 2.46235E-06 |
| RACK1 | -0.082062278 | 6.41317E-07 | 2.50181E-06 |
| CCDC25 | -0.022367032 | 6.71842E-07 | 2.6184E-06 |
| RHBDD2 | -0.017356589 | 6.73995E-07 | 2.62429E-06 |
| NDUFV2 | -0.051328644 | 6.78936E-07 | 2.63983E-06 |
| TMEM251 | -0.020035824 | 6.79274E-07 | 2.63983E-06 |
| BX284668. | -0.023005377 | 6.91513E-07 | 2.68485E-06 |
| SIGIRR | 0.047983623 | 6.94827E-07 | 2.69517E-06 |
| YWHAH | -0.016745168 | 7.03695E-07 | 2.72699E-06 |
| FKBP2 | 0.017876388 | 7.18756E-07 | 2.78272E-06 |
| COX7B | -0.072749978 | 7.19969E-07 | 2.78479E-06 |
| DNAJA2 | -0.025731768 | 7.30731E-07 | 2.82375E-06 |
| PHPT1 | -0.052898876 | 7.61893E-07 | 2.94139E-06 |
| HCFC1R1 | -0.030926918 | 7.84035E-07 | 3.02403E-06 |
| GOSR2 | -0.022673145 | 7.86525E-07 | 3.03078E-06 |
| TMX2 | -0.015460994 | 7.88198E-07 | 3.03438E-06 |
| PPID | -0.016713114 | 8.01402E-07 | 3.08232E-06 |
| GNAQ | 0.020093529 | 8.07685E-07 | 3.10357E-06 |
| UBE2N | -0.023415889 | 8.14607E-07 | 3.12724E-06 |
| MDM2 | -0.016944489 | 8.27655E-07 | 3.17359E-06 |
| ATP2A2 | -0.017872563 | 8.2823E-07 | 3.17359E-06 |
| ECHDC3 | -0.035213201 | 8.30249E-07 | 3.17836E-06 |
| CAPNS1 | -0.034978301 | 8.38323E-07 | 3.20627E-06 |
| RFC1 | -0.020847426 | 8.60115E-07 | 3.28655E-06 |
| PSMD2 | -0.023612895 | 8.84319E-07 | 3.37589E-06 |
| PDAP1 | -0.027662597 | 8.91308E-07 | 3.39941E-06 |
| TRPS1 | 0.021555756 | 8.99035E-07 | 3.42569E-06 |
| ZNRD1 | -0.01814402 | 9.242E-07 | 3.51831E-06 |
| PLXDC2 | 0.026264497 | 9.64775E-07 | 3.66937E-06 |

| | | | |
|---|---|---|---|
| ANKIB1 | 0.026877932 | 9.6665E-07 | 3.67309E-06 |
| RNF152 | 0.049469995 | 9.70173E-07 | 3.68306E-06 |
| PROS1 | -0.021269023 | 9.80884E-07 | 3.72028E-06 |
| TMEM139 | -0.022110245 | 1.00938E-06 | 3.82481E-06 |
| EIF3L | -0.028212607 | 1.0108E-06 | 3.82666E-06 |
| ALG5 | -0.017401006 | 1.02546E-06 | 3.8786E-06 |
| SSBP1 | -0.036945266 | 1.06637E-06 | 4.02959E-06 |
| MSMO1 | -0.020225932 | 1.06883E-06 | 4.03518E-06 |
| HEBP1 | -0.023132512 | 1.11282E-06 | 4.19737E-06 |
| NOP10 | -0.04342875 | 1.13903E-06 | 4.29231E-06 |
| PLPBP | -0.02367213 | 1.19811E-06 | 4.50854E-06 |
| HNMT | 0.043511054 | 1.19861E-06 | 4.50854E-06 |
| ARF5 | -0.025751679 | 1.23275E-06 | 4.63268E-06 |
| TFG | -0.022619517 | 1.26339E-06 | 4.7435E-06 |
| SPATS2L | -0.034294336 | 1.2733E-06 | 4.77632E-06 |
| GANAB | -0.019412473 | 1.28347E-06 | 4.81008E-06 |
| NAPA | -0.025292954 | 1.29323E-06 | 4.84223E-06 |
| DDX18 | -0.024763515 | 1.30183E-06 | 4.87E-06 |
| GRHPR | 0.049065074 | 1.3113E-06 | 4.89917E-06 |
| MYO9A | 0.024339733 | 1.31202E-06 | 4.89917E-06 |
| CAPZB | -0.030843206 | 1.32783E-06 | 4.95371E-06 |
| NAP1L4 | -0.025316837 | 1.33174E-06 | 4.96375E-06 |
| GMDS | -0.018400522 | 1.35509E-06 | 5.04619E-06 |
| CALD1 | -0.037110742 | 1.41673E-06 | 5.27097E-06 |
| MPDU1 | -0.016053953 | 1.42181E-06 | 5.28492E-06 |
| TCF25 | -0.026451941 | 1.42306E-06 | 5.28492E-06 |
| OXT | 0.021957515 | 1.43867E-06 | 5.33806E-06 |
| SLC2A4RG | -0.04205121 | 1.45524E-06 | 5.39464E-06 |
| BOLA3 | -0.031738831 | 1.45927E-06 | 5.4047E-06 |
| PRDX2 | -0.055600173 | 1.55138E-06 | 5.74067E-06 |
| SRRM2 | -0.032398067 | 1.57834E-06 | 5.83518E-06 |
| VCAM1 | -0.053055342 | 1.58839E-06 | 5.86702E-06 |
| GCC2 | -0.024689447 | 1.59324E-06 | 5.87963E-06 |
| FAF1 | 0.016399813 | 1.59738E-06 | 5.88961E-06 |
| ADH5 | -0.041655117 | 1.60476E-06 | 5.91152E-06 |
| MDH2 | -0.038054262 | 1.61791E-06 | 5.9546E-06 |
| FAM13A | 0.022749734 | 1.62116E-06 | 5.96122E-06 |
| MARF1 | 0.017565883 | 1.64277E-06 | 6.03528E-06 |
| TIPRL | -0.019313158 | 1.68902E-06 | 6.19961E-06 |
| RDH5 | -0.016350298 | 1.72159E-06 | 6.31353E-06 |
| AGPAT1 | -0.018735181 | 1.72885E-06 | 6.33448E-06 |
| LSM12 | -0.020182649 | 1.75055E-06 | 6.40826E-06 |
| TMED1 | -0.01684224 | 1.76736E-06 | 6.46402E-06 |
| SUPT16H | -0.018355714 | 1.78259E-06 | 6.51391E-06 |
| ARPC2 | -0.029141739 | 1.799E-06 | 6.56803E-06 |
| ZDHHC4 | -0.017446935 | 1.81132E-06 | 6.6069E-06 |
| CDC37 | -0.021520675 | 1.81287E-06 | 6.6069E-06 |

| | | | |
|---|---|---|---|
| HNRNPH3 | -0.022651662 | 1.85886E-06 | 6.7685E-06 |
| SLC37A4 | -0.03085267 | 1.87683E-06 | 6.82786E-06 |
| BUD31 | -0.028170709 | 1.92257E-06 | 6.98806E-06 |
| TXN2 | -0.032901447 | 1.97176E-06 | 7.16052E-06 |
| DSP | -0.023661203 | 2.00395E-06 | 7.27099E-06 |
| MPHOSPH | -0.019088973 | 2.0356E-06 | 7.37928E-06 |
| LPP | 0.031550564 | 2.04467E-06 | 7.40559E-06 |
| RAB8A | -0.01895547 | 2.08668E-06 | 7.5511E-06 |
| DPCD | -0.016590556 | 2.10279E-06 | 7.6027E-06 |
| SMIM30 | -0.025776467 | 2.1542E-06 | 7.78171E-06 |
| RAB14 | -0.027160759 | 2.16827E-06 | 7.82564E-06 |
| C11orf52 | -0.019982657 | 2.2041E-06 | 7.94795E-06 |
| NDUFS5 | -0.059096556 | 2.22642E-06 | 8.02136E-06 |
| CMAS | -0.016837724 | 2.24965E-06 | 8.09796E-06 |
| NR2F1 | 0.031042529 | 2.3705E-06 | 8.52549E-06 |
| RREB1 | -0.018458656 | 2.39388E-06 | 8.60201E-06 |
| RPL35A | -0.087062722 | 2.39746E-06 | 8.60735E-06 |
| RSL1D1 | -0.027781961 | 2.50697E-06 | 8.99265E-06 |
| UBE2E2 | 0.023882767 | 2.57252E-06 | 9.21968E-06 |
| HNF4A | -0.037744386 | 2.64557E-06 | 9.47322E-06 |
| KIAA1522 | -0.014550915 | 2.66676E-06 | 9.54077E-06 |
| FUNDC2 | -0.026030175 | 2.71213E-06 | 9.69462E-06 |
| PHB2 | -0.030975075 | 2.73566E-06 | 9.7702E-06 |
| KRR1 | -0.016728541 | 2.75447E-06 | 9.82869E-06 |
| ZNF706 | -0.030034781 | 2.75683E-06 | 9.82869E-06 |
| PSMA6 | -0.0269505 | 2.80719E-06 | 9.99955E-06 |
| SLC36A2 | 0.031516136 | 2.8195E-06 | 1.00347E-05 |
| OAF | -0.017295103 | 2.89599E-06 | 1.0298E-05 |
| NUDT16 | -0.023664742 | 2.91574E-06 | 1.03592E-05 |
| WSB2 | -0.017029488 | 2.92701E-06 | 1.03903E-05 |
| COX14 | -0.049460866 | 2.94579E-06 | 1.04479E-05 |
| DNAJC1 | -0.016059328 | 2.98589E-06 | 1.05809E-05 |
| NDUFA7 | -0.039571594 | 3.10287E-06 | 1.0986E-05 |
| TMEM18 | -0.016851578 | 3.14555E-06 | 1.11275E-05 |
| THUMPD1 | -0.020984771 | 3.1626E-06 | 1.11781E-05 |
| CAPN3 | 0.032346298 | 3.1763E-06 | 1.12169E-05 |
| PSAT1 | -0.044567732 | 3.23799E-06 | 1.14249E-05 |
| PIGP | -0.018943716 | 3.29764E-06 | 1.16254E-05 |
| METTL5 | -0.019117484 | 3.30669E-06 | 1.16473E-05 |
| COX11 | -0.024632529 | 3.31425E-06 | 1.16639E-05 |
| LSM10 | -0.019560453 | 3.31939E-06 | 1.1672E-05 |
| RTRAF | -0.031327235 | 3.37415E-06 | 1.18543E-05 |
| SDHA | -0.031594876 | 3.47835E-06 | 1.221E-05 |
| ERI3 | -0.027550574 | 3.49573E-06 | 1.22605E-05 |
| ACP1 | -0.03215727 | 3.51792E-06 | 1.23278E-05 |
| BSCL2 | -0.022971329 | 3.53267E-06 | 1.23689E-05 |
| PRKCA | 0.016663418 | 3.55144E-06 | 1.2424E-05 |

| | | | |
|---|---|---|---|
| IVD | -0.028898842 | 3.56492E-06 | 1.24605E-05 |
| RAB18 | -0.02484856 | 3.58028E-06 | 1.24999E-05 |
| GATAD1 | -0.018075798 | 3.58229E-06 | 1.24999E-05 |
| SNX1 | -0.018670208 | 3.58634E-06 | 1.25034E-05 |
| HMOX1 | -0.027226315 | 3.60806E-06 | 1.25581E-05 |
| TSTA3 | -0.017909887 | 3.60817E-06 | 1.25581E-05 |
| PRDX5 | -0.058998718 | 3.63118E-06 | 1.26275E-05 |
| CHMP2A | -0.040207869 | 3.63441E-06 | 1.2628E-05 |
| HADHA | -0.032000336 | 3.6511E-06 | 1.26753E-05 |
| SRM | -0.016094575 | 3.75895E-06 | 1.30386E-05 |
| NREP | -0.024475033 | 3.7659E-06 | 1.30517E-05 |
| TMEM98 | -0.01743412 | 3.79264E-06 | 1.31333E-05 |
| ARMC10 | -0.019051705 | 3.85231E-06 | 1.33287E-05 |
| NDUFAF4 | -0.017298643 | 3.88621E-06 | 1.34346E-05 |
| TRIR | -0.04009512 | 3.90979E-06 | 1.35048E-05 |
| CIAO1 | -0.023238534 | 3.96611E-06 | 1.36878E-05 |
| SELENOW | -0.042967016 | 3.9915E-06 | 1.37638E-05 |
| FLYWCH2 | -0.017545083 | 4.00031E-06 | 1.37826E-05 |
| VAPB | -0.015644847 | 4.063E-06 | 1.39868E-05 |
| RAB5C | -0.036058069 | 4.14395E-06 | 1.42535E-05 |
| SEL1L | -0.016973107 | 4.1669E-06 | 1.43205E-05 |
| TCEAL4 | -0.029460161 | 4.24524E-06 | 1.45775E-05 |
| MED10 | -0.016857389 | 4.32178E-06 | 1.48279E-05 |
| GOSR1 | -0.017843387 | 4.36586E-06 | 1.49613E-05 |
| IMMP1L | -0.018562052 | 4.36796E-06 | 1.49613E-05 |
| CDV3 | -0.02175284 | 4.40297E-06 | 1.50686E-05 |
| PKD2 | 0.016392592 | 4.47366E-06 | 1.52978E-05 |
| ERGIC2 | -0.015678395 | 4.54425E-06 | 1.55262E-05 |
| TLN1 | -0.018896899 | 4.58283E-06 | 1.5645E-05 |
| TMEM126 | -0.022872209 | 4.62855E-06 | 1.57879E-05 |
| MFSD4B | 0.019930855 | 4.63536E-06 | 1.5798E-05 |
| C17orf49 | -0.015243791 | 4.68979E-06 | 1.59702E-05 |
| EPN1 | -0.025007112 | 4.87879E-06 | 1.66E-05 |
| PNISR | -0.0254345 | 4.9435E-06 | 1.68063E-05 |
| NDUFS6 | -0.048466246 | 4.99063E-06 | 1.69524E-05 |
| SLC25A23 | -0.017115732 | 5.06451E-06 | 1.71777E-05 |
| FUOM | -0.041248505 | 5.06532E-06 | 1.71777E-05 |
| HNRNPU | -0.034210329 | 5.07244E-06 | 1.71876E-05 |
| SLC51B | -0.018628669 | 5.10858E-06 | 1.72958E-05 |
| HMGN4 | -0.017557743 | 5.12813E-06 | 1.73476E-05 |
| RFK | -0.020066322 | 5.26629E-06 | 1.78003E-05 |
| ITPR2 | 0.018631327 | 5.4484E-06 | 1.84007E-05 |
| HOOK2 | -0.018452769 | 5.46176E-06 | 1.84306E-05 |
| MIGA1 | -0.016994799 | 5.47655E-06 | 1.84653E-05 |
| TOMM22 | -0.027261256 | 5.54638E-06 | 1.86854E-05 |
| DNALI1 | -0.015153474 | 5.57637E-06 | 1.8771E-05 |
| MGST1 | -0.049444384 | 5.58292E-06 | 1.87721E-05 |

| | | | |
|---|---|---|---|
| CCNL2 | -0.015456931 | 5.58929E-06 | 1.87721E-05 |
| MAGEF1 | -0.016468748 | 5.59042E-06 | 1.87721E-05 |
| SUPT4H1 | -0.026697607 | 5.61369E-06 | 1.88348E-05 |
| CLDN10 | -0.030335746 | 5.74134E-06 | 1.92345E-05 |
| SELENOK | -0.034429172 | 5.74499E-06 | 1.92345E-05 |
| YIF1A | -0.022488838 | 5.74689E-06 | 1.92345E-05 |
| MRPL43 | -0.028099212 | 5.79489E-06 | 1.93793E-05 |
| RPS2 | -0.067431375 | 5.89618E-06 | 1.9702E-05 |
| SUOX | -0.01659353 | 5.95324E-06 | 1.98765E-05 |
| MRPS21 | -0.040947729 | 5.97614E-06 | 1.99367E-05 |
| NAPEPLD | 0.018445665 | 6.04755E-06 | 2.01585E-05 |
| TMEM30A | -0.017698245 | 6.2565E-06 | 2.08308E-05 |
| CRYM | -0.028764954 | 6.2605E-06 | 2.08308E-05 |
| MAOB | 0.030837789 | 6.26893E-06 | 2.08308E-05 |
| SLC5A2 | -0.028722915 | 6.26957E-06 | 2.08308E-05 |
| TNS3 | 0.020863228 | 6.29673E-06 | 2.09041E-05 |
| ATP6V0A1 | -0.023572938 | 6.33089E-06 | 2.0994E-05 |
| ECH1 | -0.046980059 | 6.33405E-06 | 2.0994E-05 |
| KLHL36 | -0.016239401 | 6.36692E-06 | 2.10859E-05 |
| NDUFB6 | -0.031296749 | 6.39694E-06 | 2.11683E-05 |
| HSBP1 | -0.039680864 | 6.47634E-06 | 2.14137E-05 |
| ZBTB7A | -0.015898572 | 6.50006E-06 | 2.14748E-05 |
| C2orf74 | -0.018583664 | 6.51759E-06 | 2.15154E-05 |
| CCT2 | -0.023320315 | 6.63011E-06 | 2.18524E-05 |
| CTTN | -0.023695004 | 6.63034E-06 | 2.18524E-05 |
| NDUFB10 | -0.047720741 | 6.64254E-06 | 2.1875E-05 |
| CALM1 | -0.066425735 | 6.7968E-06 | 2.23651E-05 |
| FRAS1 | 0.018091871 | 6.93076E-06 | 2.27876E-05 |
| MIR99AHG | 0.019655817 | 7.03743E-06 | 2.31198E-05 |
| ELP5 | -0.019552009 | 7.22409E-06 | 2.3714E-05 |
| STUB1 | -0.034494362 | 7.25205E-06 | 2.37867E-05 |
| SMARCE1 | -0.019656926 | 7.33982E-06 | 2.40554E-05 |
| RNF146 | -0.016943961 | 7.38372E-06 | 2.41799E-05 |
| TBC1D19 | 0.015281219 | 7.41373E-06 | 2.42588E-05 |
| DFFA | -0.016897278 | 7.45734E-06 | 2.43821E-05 |
| MRPL22 | -0.024081783 | 7.69069E-06 | 2.5125E-05 |
| CGNL1 | 0.027008013 | 7.70049E-06 | 2.5137E-05 |
| PUM2 | 0.0188363 | 7.73187E-06 | 2.52193E-05 |
| TPM3 | -0.027287383 | 7.76583E-06 | 2.53099E-05 |
| AGPAT3 | -0.030593821 | 7.82427E-06 | 2.54743E-05 |
| SCO1 | -0.015456027 | 7.82869E-06 | 2.54743E-05 |
| DDRGK1 | -0.01836425 | 7.85218E-06 | 2.55305E-05 |
| AKIRIN1 | -0.016633408 | 7.99552E-06 | 2.59759E-05 |
| SIVA1 | -0.033903047 | 8.11886E-06 | 2.63417E-05 |
| CARHSP1 | 0.032768698 | 8.12094E-06 | 2.63417E-05 |
| JTB | -0.030384055 | 8.20613E-06 | 2.65969E-05 |
| AOC1 | -0.037861402 | 8.24118E-06 | 2.66894E-05 |

| | | | |
|---|---|---|---|
| WWTR1 | 0.022077873 | 8.26191E-06 | 2.67355E-05 |
| SERBP1 | -0.036771982 | 8.43657E-06 | 2.72791E-05 |
| CLTA | -0.044177687 | 8.44414E-06 | 2.72821E-05 |
| COQ4 | -0.024460247 | 8.53309E-06 | 2.75478E-05 |
| HBP1 | 0.020206889 | 8.59435E-06 | 2.77237E-05 |
| ATXN7L3B | -0.021377347 | 8.6243E-06 | 2.77874E-05 |
| MRPS7 | -0.024148897 | 8.62765E-06 | 2.77874E-05 |
| PAIP1 | -0.021167909 | 8.67696E-06 | 2.79169E-05 |
| HEXA | -0.020539308 | 8.68149E-06 | 2.79169E-05 |
| SLC13A2 | -0.019572459 | 8.78632E-06 | 2.82319E-05 |
| FNDC3B | 0.019315374 | 9.12052E-06 | 2.92828E-05 |
| BAG1 | -0.033143289 | 9.38335E-06 | 3.00982E-05 |
| TMEM134 | -0.022729227 | 9.38916E-06 | 3.00982E-05 |
| TRIOBP | -0.014322778 | 9.42309E-06 | 3.01833E-05 |
| RNF187 | -0.025179763 | 9.6574E-06 | 3.09097E-05 |
| AKR7A2 | -0.04359579 | 9.68066E-06 | 3.096E-05 |
| VDAC2 | -0.030644833 | 9.76372E-06 | 3.11913E-05 |
| SFT2D1 | -0.021552585 | 9.76821E-06 | 3.11913E-05 |
| SRRM1 | -0.026773567 | 9.92726E-06 | 3.16745E-05 |
| EEF1G | -0.063042621 | 1.00625E-05 | 3.20811E-05 |
| ACSL1 | -0.03291803 | 1.02882E-05 | 3.27752E-05 |
| PSMD11 | -0.022575571 | 1.03085E-05 | 3.27938E-05 |
| PLEKHB2 | -0.030361872 | 1.03101E-05 | 3.27938E-05 |
| TBC1D4 | 0.018305244 | 1.03229E-05 | 3.28091E-05 |
| RTL8A | -0.017880805 | 1.05853E-05 | 3.36171E-05 |
| SLC30A9 | -0.02206267 | 1.07735E-05 | 3.41883E-05 |
| UROS | -0.025863394 | 1.09905E-05 | 3.48493E-05 |
| BCL2 | 0.021849617 | 1.09998E-05 | 3.48493E-05 |
| SLC66A2 | -0.016814033 | 1.10077E-05 | 3.48493E-05 |
| ATP5PO | -0.040460515 | 1.10182E-05 | 3.48493E-05 |
| ARPC5 | -0.020485853 | 1.10243E-05 | 3.48493E-05 |
| TBC1D1 | -0.019255663 | 1.14999E-05 | 3.63249E-05 |
| DNPH1 | -0.056583823 | 1.16737E-05 | 3.68455E-05 |
| S100A11 | -0.054636122 | 1.16985E-05 | 3.68952E-05 |
| TUG1 | -0.016603064 | 1.17774E-05 | 3.70967E-05 |
| PSMD12 | -0.020392191 | 1.17805E-05 | 3.70967E-05 |
| DERA | -0.027989511 | 1.18127E-05 | 3.71697E-05 |
| ZDHHC12 | -0.014640127 | 1.18589E-05 | 3.72865E-05 |
| KHK | -0.065785928 | 1.20109E-05 | 3.77354E-05 |
| G3BP2 | -0.024731363 | 1.22667E-05 | 3.85096E-05 |
| MYO15B | 0.02030212 | 1.24936E-05 | 3.91917E-05 |
| GLG1 | -0.024823697 | 1.25381E-05 | 3.93014E-05 |
| UPK3BL1 | -0.015413413 | 1.26169E-05 | 3.95182E-05 |
| POLR2C | -0.021079708 | 1.26282E-05 | 3.95234E-05 |
| COQ5 | -0.015476354 | 1.26759E-05 | 3.96423E-05 |
| CAP1 | -0.021331909 | 1.28405E-05 | 4.01265E-05 |
| CNDP2 | -0.045586048 | 1.28871E-05 | 4.02415E-05 |

| | | | |
|---|---|---|---|
| CYHR1 | -0.017144597 | 1.29017E-05 | 4.02563E-05 |
| GCA | -0.014773512 | 1.30349E-05 | 4.06412E-05 |
| SLBP | -0.014421294 | 1.31254E-05 | 4.08923E-05 |
| GPR108 | -0.015670152 | 1.31779E-05 | 4.10245E-05 |
| COPS5 | -0.018852087 | 1.34898E-05 | 4.19638E-05 |
| ATP6V1E1 | -0.035916177 | 1.35168E-05 | 4.20029E-05 |
| BNIP3L | -0.030524815 | 1.35229E-05 | 4.20029E-05 |
| MBNL1 | 0.023265012 | 1.35926E-05 | 4.21875E-05 |
| WASHC3 | -0.020079339 | 1.3652E-05 | 4.23006E-05 |
| BUD23 | -0.018046702 | 1.36568E-05 | 4.23006E-05 |
| HSPA4 | -0.02234773 | 1.366E-05 | 4.23006E-05 |
| MCFD2 | -0.016006488 | 1.38176E-05 | 4.27564E-05 |
| RBMS1 | 0.017099716 | 1.4224E-05 | 4.39806E-05 |
| SEC22B | -0.020659248 | 1.42566E-05 | 4.40481E-05 |
| SVBP | -0.01429867 | 1.42913E-05 | 4.41221E-05 |
| PINK1 | -0.021677563 | 1.43082E-05 | 4.41411E-05 |
| CCDC58 | -0.019184721 | 1.47314E-05 | 4.54125E-05 |
| ATXN7L1 | 0.016003646 | 1.4787E-05 | 4.55497E-05 |
| RPS5 | -0.06739015 | 1.48141E-05 | 4.55989E-05 |
| ACBD3 | -0.015283378 | 1.49992E-05 | 4.61337E-05 |
| SH3YL1 | -0.043338792 | 1.50103E-05 | 4.61337E-05 |
| SH3D19 | 0.017151197 | 1.51204E-05 | 4.64371E-05 |
| CSNK1D | -0.017278164 | 1.52202E-05 | 4.67088E-05 |
| MED8 | -0.0154098 | 1.54571E-05 | 4.74002E-05 |
| CHMP5 | -0.031263552 | 1.59176E-05 | 4.8776E-05 |
| PLA2G12A | -0.017335872 | 1.61133E-05 | 4.93388E-05 |
| PLD3 | -0.015729204 | 1.61701E-05 | 4.94757E-05 |
| DGUOK | -0.01896858 | 1.63072E-05 | 4.98581E-05 |
| SCD5 | -0.016943765 | 1.63574E-05 | 4.99741E-05 |
| TRAM1 | -0.026206253 | 1.64068E-05 | 5.00722E-05 |
| EHHADH | -0.036822686 | 1.64139E-05 | 5.00722E-05 |
| C5orf15 | -0.018162586 | 1.65579E-05 | 5.0474E-05 |
| C1D | -0.026285456 | 1.71484E-05 | 5.22351E-05 |
| DPY30 | -0.030069422 | 1.73078E-05 | 5.26816E-05 |
| SLC3A1 | -0.051650978 | 1.74108E-05 | 5.29557E-05 |
| NUDT16L1 | -0.020660333 | 1.7646E-05 | 5.36314E-05 |
| CTSZ | -0.019387192 | 1.77868E-05 | 5.40191E-05 |
| NDUFAF3 | -0.037374006 | 1.78232E-05 | 5.40897E-05 |
| SMS | -0.034063941 | 1.81165E-05 | 5.4939E-05 |
| ATP5MC2 | -0.058567503 | 1.82431E-05 | 5.5282E-05 |
| AC026803. | 0.023325401 | 1.85605E-05 | 5.62024E-05 |
| POLDIP2 | -0.025729525 | 1.87517E-05 | 5.67395E-05 |
| SUMO1 | -0.037014634 | 1.879E-05 | 5.68135E-05 |
| WDR45B | -0.019400998 | 1.88355E-05 | 5.69091E-05 |
| NIPSNAP1 | -0.027349783 | 1.89027E-05 | 5.70701E-05 |
| FH | -0.019579226 | 1.9195E-05 | 5.79098E-05 |
| CNBP | -0.028584641 | 1.95662E-05 | 5.89863E-05 |

| | | | |
|---|---|---|---|
| OIP5-AS1 | -0.017457882 | 1.96073E-05 | 5.90667E-05 |
| BRPF3 | 0.014607556 | 1.99076E-05 | 5.99276E-05 |
| NAT8 | -0.065185245 | 2.0049E-05 | 6.03089E-05 |
| TSR2 | -0.015948632 | 2.03236E-05 | 6.10901E-05 |
| UBE2A | -0.016351258 | 2.05378E-05 | 6.16887E-05 |
| LSM3 | -0.038269473 | 2.06426E-05 | 6.19581E-05 |
| SERPINF2 | -0.020085076 | 2.07171E-05 | 6.21361E-05 |
| ARPC5L | -0.016340824 | 2.08026E-05 | 6.2347E-05 |
| RPL27 | -0.063117045 | 2.16233E-05 | 6.47594E-05 |
| FNBP1L | -0.020058187 | 2.17041E-05 | 6.49538E-05 |
| UBXN1 | -0.029053106 | 2.2111E-05 | 6.61233E-05 |
| BBIP1 | -0.015471998 | 2.23436E-05 | 6.67488E-05 |
| CHCHD10 | -0.058796498 | 2.23527E-05 | 6.67488E-05 |
| TRIP11 | -0.017950919 | 2.29105E-05 | 6.83648E-05 |
| SLC22A12 | -0.035785545 | 2.31056E-05 | 6.88967E-05 |
| EIF1AX | -0.032657989 | 2.31728E-05 | 6.90468E-05 |
| GLB1 | -0.014529522 | 2.34113E-05 | 6.97067E-05 |
| FKBP8 | -0.030117668 | 2.37163E-05 | 7.05636E-05 |
| RPL22 | -0.065081368 | 2.3976E-05 | 7.12846E-05 |
| ST13 | 0.053235388 | 2.44631E-05 | 7.26801E-05 |
| DLG1 | 0.015860599 | 2.45342E-05 | 7.28386E-05 |
| STMP1 | -0.02593987 | 2.47465E-05 | 7.33791E-05 |
| KIFC3 | 0.018466515 | 2.4752E-05 | 7.33791E-05 |
| PRUNE2 | 0.026009573 | 2.48908E-05 | 7.37198E-05 |
| GPAA1 | -0.02195612 | 2.49029E-05 | 7.37198E-05 |
| PRKDC | -0.018118947 | 2.5245E-05 | 7.46785E-05 |
| NDUFA8 | -0.026021804 | 2.5894E-05 | 7.65432E-05 |
| DHRS4-AS1 | -0.027949253 | 2.59587E-05 | 7.66299E-05 |
| CCDC186 | -0.018069463 | 2.59607E-05 | 7.66299E-05 |
| AP2M1 | -0.032144161 | 2.60277E-05 | 7.67724E-05 |
| DHRS12 | -0.015763792 | 2.61041E-05 | 7.69423E-05 |
| NBN | -0.013980154 | 2.65038E-05 | 7.80644E-05 |
| DCTN3 | -0.02665163 | 2.68528E-05 | 7.90355E-05 |
| GLRX | -0.049437319 | 2.72484E-05 | 8.01424E-05 |
| ADIRF | -0.070303539 | 2.72905E-05 | 8.02087E-05 |
| NUDT4 | -0.016650094 | 2.73476E-05 | 8.03189E-05 |
| SRA1 | -0.017139757 | 2.74804E-05 | 8.06513E-05 |
| LTBP3 | -0.015946011 | 2.7913E-05 | 8.18622E-05 |
| TTC3 | -0.022892473 | 2.80875E-05 | 8.22635E-05 |
| MSN | -0.023192565 | 2.809E-05 | 8.22635E-05 |
| AP3S2 | -0.014717637 | 2.81591E-05 | 8.24071E-05 |
| PRMT1 | -0.01741043 | 2.84921E-05 | 8.33222E-05 |
| CFLAR | -0.023900323 | 2.88328E-05 | 8.42584E-05 |
| FAM32A | -0.018685092 | 2.89778E-05 | 8.46216E-05 |
| WDR83OS | -0.03775045 | 2.90459E-05 | 8.47602E-05 |
| FGFR3 | -0.01741584 | 2.92604E-05 | 8.53256E-05 |
| ABCC4 | 0.019170662 | 2.94115E-05 | 8.56791E-05 |

| | | | |
|---|---|---|---|
| BNIP3 | -0.041877714 | 2.94235E-05 | 8.56791E-05 |
| CDKN2AIPI | -0.015649901 | 2.94711E-05 | 8.5757E-05 |
| PARP4 | -0.017077351 | 2.99526E-05 | 8.70963E-05 |
| POLR2L | -0.060336799 | 3.0394E-05 | 8.83169E-05 |
| CCAR1 | -0.015035507 | 3.07217E-05 | 8.92061E-05 |
| PDHB | -0.022401222 | 3.10228E-05 | 9.00167E-05 |
| GUCD1 | -0.019743524 | 3.15735E-05 | 9.15499E-05 |
| PPARGC1A | 0.015752366 | 3.16675E-05 | 9.17146E-05 |
| NTPCR | -0.017063865 | 3.16751E-05 | 9.17146E-05 |
| POP4 | -0.0149025 | 3.17849E-05 | 9.19675E-05 |
| ARFGEF1 | 0.017150683 | 3.18584E-05 | 9.21153E-05 |
| POLR2J | -0.027092074 | 3.19147E-05 | 9.22129E-05 |
| MRPS16 | -0.023246255 | 3.35394E-05 | 9.6839E-05 |
| TNS1 | 0.026065399 | 3.39412E-05 | 9.79302E-05 |
| CAMK2D | 0.016736629 | 3.43397E-05 | 9.90103E-05 |
| YY1 | -0.020978536 | 3.47695E-05 | 0.000100179 |
| FUS | -0.022249973 | 3.53491E-05 | 0.000101777 |
| TALDO1 | -0.03165673 | 3.54096E-05 | 0.00010188 |
| ZSWIM7 | -0.021758918 | 3.58937E-05 | 0.000103201 |
| PHAX | -0.014615962 | 3.60013E-05 | 0.000103426 |
| MRPL15 | -0.019704114 | 3.60224E-05 | 0.000103426 |
| IGF1R | 0.02484484 | 3.64383E-05 | 0.000104513 |
| NDUFS2 | -0.031001629 | 3.64521E-05 | 0.000104513 |
| EBPL | -0.014288471 | 3.70949E-05 | 0.000106282 |
| MLLT10 | 0.013942251 | 3.71231E-05 | 0.000106288 |
| ITGB8 | -0.035371183 | 3.72105E-05 | 0.000106464 |
| CHDH | -0.028939269 | 3.76875E-05 | 0.000107754 |
| HSD17B10 | -0.02879946 | 3.77432E-05 | 0.000107838 |
| MYL6B | -0.025199699 | 3.86749E-05 | 0.000110423 |
| MICOS13 | -0.036487716 | 3.92432E-05 | 0.000111967 |
| TSPO | -0.02401873 | 3.94653E-05 | 0.000112523 |
| ETFRF1 | -0.020519364 | 4.01422E-05 | 0.000114373 |
| ATP5MG | -0.060111542 | 4.0172E-05 | 0.000114379 |
| EIF5 | -0.028675093 | 4.04177E-05 | 0.000114998 |
| SMAD3 | 0.013518438 | 4.13362E-05 | 0.00011753 |
| TMEM150A | -0.020172856 | 4.22007E-05 | 0.000119905 |
| CDK5RAP3 | -0.014212289 | 4.32084E-05 | 0.000122683 |
| APEX1 | -0.024429719 | 4.33682E-05 | 0.000123052 |
| DEXI | -0.01677361 | 4.39776E-05 | 0.000124695 |
| SH3BP2 | -0.014975134 | 4.46764E-05 | 0.000126588 |
| CHCHD2 | -0.053034949 | 4.51745E-05 | 0.000127911 |
| ANK2 | 0.053580805 | 4.61131E-05 | 0.000130479 |
| PLS1 | -0.015774563 | 4.61929E-05 | 0.000130614 |
| FMC1 | -0.030725996 | 4.67349E-05 | 0.000132056 |
| RNF213 | -0.017953364 | 4.69197E-05 | 0.000132487 |
| PSMA1 | -0.025057383 | 4.80492E-05 | 0.000135583 |
| SMARCA5 | -0.017186813 | 4.8138E-05 | 0.00013574 |

| | | | |
|---|---|---|---|
| RABAC1 | -0.029017568 | 4.84532E-05 | 0.000136515 |
| ALDH8A1 | -0.026565723 | 4.84796E-05 | 0.000136515 |
| SNX7 | -0.015901216 | 4.8909E-05 | 0.00013763 |
| KIAA1217 | 0.016066976 | 4.90498E-05 | 0.000137931 |
| TMEM50B | -0.014496255 | 4.96765E-05 | 0.000139598 |
| RPL23 | -0.054016577 | 4.97212E-05 | 0.000139628 |
| LINC01697 | -0.020921395 | 4.98934E-05 | 0.000140016 |
| IARS | -0.015940522 | 5.04998E-05 | 0.00014162 |
| CDC26 | -0.023169768 | 5.11973E-05 | 0.000143478 |
| ASPH | -0.015939641 | 5.13921E-05 | 0.000143926 |
| VBP1 | -0.016211205 | 5.14308E-05 | 0.000143936 |
| SEMA5A | 0.023142658 | 5.23162E-05 | 0.000146223 |
| FIS1 | -0.046633515 | 5.23194E-05 | 0.000146223 |
| RHEB | -0.024991517 | 5.23672E-05 | 0.000146257 |
| PTRH1 | -0.014544005 | 5.28075E-05 | 0.000147387 |
| LTBR | -0.013943072 | 5.28636E-05 | 0.000147443 |
| MAZ | -0.017360186 | 5.29911E-05 | 0.000147698 |
| NR1H2 | -0.015521667 | 5.50856E-05 | 0.000153431 |
| EIF1B | -0.034053604 | 5.61343E-05 | 0.000156246 |
| RAB21 | -0.023364727 | 5.64499E-05 | 0.000157018 |
| PSMB1 | -0.03578681 | 5.65167E-05 | 0.000157097 |
| DCTD | -0.013110698 | 5.66165E-05 | 0.000157265 |
| VPS4B | -0.015054366 | 5.6657E-05 | 0.000157265 |
| ERH | -0.032034908 | 5.6692E-05 | 0.000157265 |
| MACC1 | -0.018097675 | 5.76986E-05 | 0.000159949 |
| PDCD2 | -0.015337007 | 5.78311E-05 | 0.000160208 |
| NARS | -0.027735018 | 5.79011E-05 | 0.000160293 |
| PECR | -0.019032931 | 5.88183E-05 | 0.000162723 |
| CTSA | -0.028819062 | 5.90847E-05 | 0.00016335 |
| KIF1B | 0.014676549 | 5.93045E-05 | 0.000163739 |
| CHMP2B | -0.017237278 | 5.93054E-05 | 0.000163739 |
| PPM1K | 0.017389162 | 5.93675E-05 | 0.0001638 |
| EGLN1 | 0.015832858 | 6.07245E-05 | 0.000167431 |
| VMP1 | -0.017556898 | 6.09538E-05 | 0.000167951 |
| ICMT | -0.014563809 | 6.14214E-05 | 0.000169125 |
| IGBP1 | -0.021714564 | 6.34597E-05 | 0.000174621 |
| TOR1AIP2 | -0.016979359 | 6.49272E-05 | 0.000178539 |
| KL | 0.036835704 | 6.58922E-05 | 0.000181071 |
| MRPL49 | -0.015545674 | 6.60486E-05 | 0.000181379 |
| IFNGR2 | -0.013395493 | 6.65474E-05 | 0.000182627 |
| LRP2 | -0.059182539 | 6.71409E-05 | 0.000184132 |
| FAM3C | -0.015024201 | 6.81898E-05 | 0.000186884 |
| NEDD8 | -0.03962998 | 6.83905E-05 | 0.000187309 |
| EIF2AK1 | -0.017198762 | 6.94206E-05 | 0.000189948 |
| ERGIC1 | -0.016393842 | 6.94467E-05 | 0.000189948 |
| SPART | -0.021351417 | 7.0289E-05 | 0.000192123 |
| NPR3 | -0.0349094 | 7.08329E-05 | 0.000193481 |

| | | | |
|---|---|---|---|
| MAPRE1 | -0.013804571 | 7.13466E-05 | 0.000194754 |
| ARL6IP1 | -0.024002929 | 7.14607E-05 | 0.000194936 |
| CSDE1 | -0.034140086 | 7.25246E-05 | 0.000197707 |
| UBE2Z | -0.014169092 | 7.27423E-05 | 0.000198168 |
| SLC39A4 | -0.025036303 | 7.29714E-05 | 0.00019866 |
| EIF3D | -0.021324797 | 7.36932E-05 | 0.000200492 |
| ALKBH5 | -0.019366788 | 7.44032E-05 | 0.00020229 |
| CEBPZ | -0.016824926 | 7.51607E-05 | 0.000204214 |
| SNRPA1 | -0.015387333 | 7.57879E-05 | 0.000205782 |
| MRPL41 | -0.043256255 | 7.59081E-05 | 0.000205972 |
| ZFAND3 | 0.017923426 | 7.60937E-05 | 0.000206339 |
| HP1BP3 | -0.023767316 | 7.68682E-05 | 0.000208301 |
| RTF2 | -0.020926559 | 7.69623E-05 | 0.000208349 |
| KHSRP | -0.016315898 | 7.69876E-05 | 0.000208349 |
| PPFIBP1 | -0.026285848 | 7.83521E-05 | 0.000211902 |
| PRKAA2 | 0.022638254 | 7.88841E-05 | 0.0002132 |
| TMEM106 | -0.021433863 | 7.9059E-05 | 0.000213337 |
| HNRNPAB | -0.018271861 | 7.90612E-05 | 0.000213337 |
| TAF9 | -0.014495446 | 7.90906E-05 | 0.000213337 |
| PLEKHF2 | -0.016739978 | 7.97413E-05 | 0.000214881 |
| PGLS | -0.025939159 | 7.97707E-05 | 0.000214881 |
| GLUD1 | -0.034963013 | 7.98205E-05 | 0.000214881 |
| DDT | -0.054453179 | 8.03699E-05 | 0.000216218 |
| TSN | -0.014673432 | 8.0653E-05 | 0.000216838 |
| HTATSF1 | -0.01296999 | 8.08156E-05 | 0.000217132 |
| ZNF280D | 0.013710589 | 8.18381E-05 | 0.000219736 |
| SNRNP25 | -0.015948293 | 8.26749E-05 | 0.000221837 |
| DNAJC30 | -0.014652625 | 8.31898E-05 | 0.000223073 |
| RPS12 | -0.087790345 | 8.41529E-05 | 0.000225508 |
| MAP2K2 | -0.022311806 | 8.44257E-05 | 0.000226091 |
| SLC27A2 | -0.029594044 | 8.48567E-05 | 0.000227097 |
| HM13 | -0.015213149 | 8.56228E-05 | 0.000228998 |
| SNRPD3 | -0.026812747 | 8.66884E-05 | 0.000231696 |
| RNF13 | -0.01756901 | 8.69381E-05 | 0.000232212 |
| ZMAT2 | -0.018243499 | 8.75073E-05 | 0.000233581 |
| CPT2 | -0.014043939 | 8.79148E-05 | 0.000234516 |
| AGAP1 | 0.016693669 | 8.80886E-05 | 0.000234827 |
| GRB2 | -0.020042987 | 8.83383E-05 | 0.00023521 |
| CHPT1 | -0.023545093 | 8.8347E-05 | 0.00023521 |
| CLDN2 | -0.028643059 | 8.93267E-05 | 0.000237638 |
| FERMT2 | -0.014545395 | 8.9375E-05 | 0.000237638 |
| PPP1CB | -0.02735891 | 9.0579E-05 | 0.000240683 |
| H2AFV | -0.024273987 | 9.18588E-05 | 0.000243925 |
| DDX24 | -0.024157579 | 9.33981E-05 | 0.000247853 |
| HOXA9 | -0.016863843 | 9.65185E-05 | 0.000255968 |
| ANAPC5 | -0.01743056 | 9.73915E-05 | 0.000258116 |
| VAMP5 | -0.013689235 | 9.82933E-05 | 0.000260303 |

| | | | |
|---|---|---|---|
| CLCN3 | -0.014364377 | 9.83464E-05 | 0.000260303 |
| ANAPC11 | -0.031056996 | 9.84584E-05 | 0.000260303 |
| IFRD2 | -0.016078752 | 9.84706E-05 | 0.000260303 |
| UBAC2 | -0.011944208 | 9.91296E-05 | 0.000261876 |
| ARHGAP24 | -0.029717577 | 9.98348E-05 | 0.000263569 |
| GPN3 | -0.013450982 | 0.000100598 | 0.000265413 |
| SLC47A2 | -0.015199084 | 0.000100727 | 0.000265582 |
| YDJC | -0.013255354 | 0.000102245 | 0.000269412 |
| TRAPPC3 | -0.015808273 | 0.000103156 | 0.000271638 |
| MRPL50 | -0.016506138 | 0.000103901 | 0.000273425 |
| TYW3 | -0.013618909 | 0.000104158 | 0.000273923 |
| ZFP91 | -0.014955287 | 0.000106249 | 0.000279245 |
| IGF2R | -0.018049234 | 0.000106457 | 0.000279611 |
| TSNAX | -0.015463427 | 0.000107 | 0.000280857 |
| GSK3A | -0.013720229 | 0.000107635 | 0.000282345 |
| AGTRAP | -0.017796725 | 0.000110254 | 0.000289028 |
| PPP1R35 | -0.012888682 | 0.000110524 | 0.000289504 |
| FAM120AO | -0.013191328 | 0.000110576 | 0.000289504 |
| SMARCA4 | -0.012571765 | 0.000111136 | 0.000290783 |
| MRPS35 | -0.020680998 | 0.000112084 | 0.000293077 |
| FAU | -0.06549776 | 0.000112192 | 0.000293173 |
| WWP1 | 0.014270905 | 0.000113267 | 0.000295792 |
| PCNA | -0.014143961 | 0.000113694 | 0.00029672 |
| DNAJC8 | -0.022371646 | 0.000114497 | 0.000298625 |
| AP1S1 | -0.022292606 | 0.000116602 | 0.000303922 |
| SMC3 | -0.013424417 | 0.000117274 | 0.000305479 |
| IFNAR1 | -0.015438577 | 0.000117628 | 0.000306118 |
| TXNDC9 | -0.014274942 | 0.000117669 | 0.000306118 |
| TACC1 | 0.013083687 | 0.000117981 | 0.000306736 |
| RAC1 | -0.033698515 | 0.000118327 | 0.000307349 |
| PTK2 | 0.015410561 | 0.000118367 | 0.000307349 |
| ACE2 | -0.020053228 | 0.000118712 | 0.000308049 |
| NPEPPS | 0.017978169 | 0.000118859 | 0.000308237 |
| ZC3H15 | -0.022556593 | 0.000120752 | 0.000312948 |
| PDS5A | 0.014605085 | 0.000120897 | 0.000313125 |
| SHISA5 | -0.013374777 | 0.000121344 | 0.000314084 |
| COPZ1 | -0.023266836 | 0.000123208 | 0.000318707 |
| HERPUD1 | -0.028490332 | 0.000123724 | 0.000319843 |
| EIF2AK2 | -0.014797673 | 0.000123968 | 0.000320269 |
| ERBB3 | -0.019693591 | 0.000126002 | 0.000325319 |
| VPS25 | -0.018364554 | 0.000126976 | 0.000327627 |
| LRRC8D | 0.014408234 | 0.000127874 | 0.000329737 |
| AP2B1 | -0.021029697 | 0.000128939 | 0.000332274 |
| MCL1 | -0.013778836 | 0.000129368 | 0.000333173 |
| SNAPC5 | -0.01306916 | 0.000130558 | 0.000336025 |
| POP7 | -0.013506142 | 0.000132689 | 0.000341295 |
| GMPR2 | -0.017541833 | 0.000133698 | 0.000343675 |

| | | | |
|---|---|---|---|
| SPG7 | -0.013022043 | 0.000134707 | 0.000346052 |
| RPL37A | -0.060991354 | 0.00013511 | 0.000346869 |
| MYL12B | -0.048225423 | 0.000135561 | 0.00034771 |
| TMEM238 | -0.019303288 | 0.000135607 | 0.00034771 |
| SNX30 | 0.017863692 | 0.000136779 | 0.000350495 |
| RBX1 | -0.033458877 | 0.000137359 | 0.000351762 |
| CHCHD5 | -0.026996617 | 0.000138702 | 0.000354981 |
| PPIA | -0.057894841 | 0.000139319 | 0.000356337 |
| CLTB | -0.026684479 | 0.000139674 | 0.000357021 |
| SCCPDH | -0.015733153 | 0.000140654 | 0.000359303 |
| NDUFB7 | -0.044250951 | 0.000142157 | 0.000362915 |
| GPD1 | -0.037125657 | 0.000142958 | 0.000364734 |
| ZCCHC10 | 0.013549527 | 0.000143335 | 0.000365468 |
| GAA | -0.013014353 | 0.0001445 | 0.000368209 |
| HDHD2 | -0.0132817 | 0.000146347 | 0.000372686 |
| THUMPD3 | -0.013851803 | 0.000146832 | 0.000373687 |
| TOB1 | -0.020173771 | 0.000147276 | 0.000374584 |
| VEGFA | -0.017449893 | 0.000148489 | 0.000377437 |
| VAT1 | -0.018566687 | 0.000151111 | 0.000383862 |
| SLC16A10 | 0.01500229 | 0.000151959 | 0.000385779 |
| IFI27L2 | -0.014094019 | 0.000155178 | 0.000393707 |
| THBS1 | 0.025181107 | 0.00016282 | 0.00041284 |
| PKIG | -0.021966624 | 0.000166521 | 0.000421963 |
| GLYR1 | -0.01785252 | 0.000168003 | 0.000425456 |
| AK3 | -0.021435211 | 0.000170029 | 0.000430321 |
| DPEP1 | -0.047505284 | 0.000170639 | 0.000431597 |
| GPC4 | -0.014536419 | 0.000172699 | 0.000436538 |
| HABP4 | -0.013920471 | 0.000173918 | 0.000439348 |
| GET3 | -0.024082882 | 0.000175754 | 0.000443713 |
| ZNRF2 | -0.016128031 | 0.00017698 | 0.000446535 |
| RPL10A | -0.056340219 | 0.000178344 | 0.000449699 |
| SWI5 | -0.015797436 | 0.000178533 | 0.000449899 |
| EIF2S1 | -0.014913863 | 0.000185345 | 0.000466778 |
| SEC63 | -0.013191685 | 0.000185533 | 0.000466965 |
| EEF1B2 | -0.052430705 | 0.00018598 | 0.000467802 |
| CUTA | -0.033047273 | 0.000189971 | 0.000477547 |
| PNRC2 | -0.018539201 | 0.00019014 | 0.000477681 |
| FZD1 | -0.013881403 | 0.000194156 | 0.00048747 |
| SSH2 | 0.016546649 | 0.00019696 | 0.000494207 |
| FKBP5 | -0.020868101 | 0.000197178 | 0.000494452 |
| EPHX1 | -0.020271283 | 0.000198005 | 0.000496222 |
| RANBP1 | -0.019506808 | 0.000198683 | 0.000497618 |
| PDZK1 | -0.047836549 | 0.000199837 | 0.000500201 |
| DAP3 | -0.014436338 | 0.000206609 | 0.000516838 |
| GOLPH3 | 0.014565455 | 0.000208841 | 0.000522103 |
| RAB10 | -0.020637679 | 0.000214343 | 0.000535532 |
| GNB2 | -0.019890736 | 0.000214657 | 0.000535989 |

| | | | |
|---|---|---|---|
| ZNF428 | -0.024364177 | 0.000216413 | 0.000540044 |
| XPO1 | -0.014667777 | 0.000217309 | 0.00054195 |
| GLRX3 | -0.014599712 | 0.000219973 | 0.000548262 |
| TSSC4 | -0.012514021 | 0.000221873 | 0.000552661 |
| THOC7 | -0.020070911 | 0.000223884 | 0.000557331 |
| DPP7 | -0.017616269 | 0.000229672 | 0.000571394 |
| USP22 | -0.018425715 | 0.000236006 | 0.000586795 |
| MCUR1 | -0.024791251 | 0.00023671 | 0.000588188 |
| XPR1 | 0.013308681 | 0.000239481 | 0.000594713 |
| RNF181 | -0.030855859 | 0.000248062 | 0.000615572 |
| LIME1 | 0.04769106 | 0.000248181 | 0.000615572 |
| RBCK1 | -0.016016588 | 0.000249279 | 0.000617923 |
| PTGES2 | -0.016456478 | 0.000253025 | 0.00062683 |
| MRPL9 | -0.013627966 | 0.000254092 | 0.000629093 |
| PSMG3 | -0.013064307 | 0.00025482 | 0.000630514 |
| ZNF330 | -0.015321623 | 0.000255371 | 0.000631495 |
| SKP1 | -0.045925864 | 0.000255864 | 0.000632334 |
| MALSU1 | -0.015840601 | 0.000257652 | 0.00063637 |
| YWHAE | -0.042622321 | 0.00025804 | 0.000636943 |
| TFAM | -0.014543806 | 0.000264452 | 0.000652379 |
| CDC37L1 | -0.013480226 | 0.000265147 | 0.000653699 |
| SSU72 | -0.024563315 | 0.000268205 | 0.000660841 |
| YIPF3 | -0.017052392 | 0.000271375 | 0.000668252 |
| SYNJ2BP | -0.014334239 | 0.000271787 | 0.000668863 |
| RPN1 | -0.019656944 | 0.000272411 | 0.000669996 |
| COA8 | -0.0180231 | 0.00027447 | 0.000674656 |
| COPE | -0.030174419 | 0.000275365 | 0.000676452 |
| NDUFAF8 | -0.029213479 | 0.000276768 | 0.000679491 |
| ARPC4 | -0.021403857 | 0.000278529 | 0.000683405 |
| PPP1R9A | 0.012361309 | 0.000278954 | 0.00068382 |
| TRMT10C | -0.013501809 | 0.000279032 | 0.00068382 |
| TMEM123 | -0.017700736 | 0.000279464 | 0.00068447 |
| DLST | -0.014199911 | 0.000281787 | 0.000689748 |
| MRPL18 | -0.020142955 | 0.000282423 | 0.000690891 |
| MRPS18B | -0.020761685 | 0.000287483 | 0.00070285 |
| LCOR | 0.012519452 | 0.000288928 | 0.000705963 |
| NQO2 | -0.039940313 | 0.000290279 | 0.00070884 |
| PLXNB1 | -0.013822224 | 0.000290452 | 0.000708841 |
| FGD5-AS1 | -0.017426105 | 0.000293815 | 0.000716621 |
| FAM3A | -0.011782539 | 0.000294783 | 0.000718555 |
| ISCA2 | -0.019077439 | 0.000297312 | 0.000724289 |
| PDHA1 | -0.020206964 | 0.000301967 | 0.000735194 |
| NUBP2 | -0.015730242 | 0.000304184 | 0.00074015 |
| SYNGR2 | -0.014987246 | 0.000304943 | 0.000741559 |
| DHX29 | -0.017355063 | 0.000306653 | 0.000745274 |
| ARMT1 | -0.018320513 | 0.000308946 | 0.000750402 |
| PAFAH1B1 | -0.017617228 | 0.00031019 | 0.000752977 |

| | | | |
|---|---|---|---|
| TIMM17A | -0.012737036 | 0.000311116 | 0.000754779 |
| SEC61G | -0.03521255 | 0.000312498 | 0.000757683 |
| WASL | -0.022017619 | 0.000313855 | 0.000760524 |
| ATP2B1 | -0.012108181 | 0.000314211 | 0.000760937 |
| OSBPL8 | 0.026085648 | 0.000318329 | 0.000770362 |
| SRSF2 | -0.019130054 | 0.000318479 | 0.000770362 |
| RPS25 | -0.064288359 | 0.000319439 | 0.000772229 |
| TST | 0.039087566 | 0.000323041 | 0.000780202 |
| NORAD | -0.025520125 | 0.000323118 | 0.000780202 |
| NAPRT | 0.024731202 | 0.000330751 | 0.000798163 |
| IDS | -0.013628206 | 0.000337018 | 0.000812808 |
| LZIC | -0.014713035 | 0.000337652 | 0.00081369 |
| PDLIM5 | 0.016592964 | 0.00033778 | 0.00081369 |
| CLMN | 0.014253924 | 0.000342546 | 0.000824685 |
| WSB1 | -0.015463247 | 0.00034315 | 0.000825654 |
| FAM104B | -0.015634747 | 0.000343767 | 0.000826311 |
| RIOK3 | -0.019537559 | 0.000343826 | 0.000826311 |
| MIR4458H | -0.023886528 | 0.000344976 | 0.000828588 |
| BLOC1S5 | -0.012865185 | 0.000346622 | 0.000831851 |
| HIP1R | -0.013282184 | 0.00034674 | 0.000831851 |
| CTBP1 | -0.014802596 | 0.000348923 | 0.000836599 |
| MMP24OS | -0.028359866 | 0.000353994 | 0.000848262 |
| RPS28 | 0.081300166 | 0.000356957 | 0.000854802 |
| EAPP | -0.017224431 | 0.000357141 | 0.000854802 |
| LINC02294 | 0.018390328 | 0.000359676 | 0.000860368 |
| BICDL1 | 0.018495097 | 0.000360556 | 0.000861972 |
| UBE2R2 | -0.017020162 | 0.000362537 | 0.000866202 |
| TAF1D | -0.017295939 | 0.000363733 | 0.000868398 |
| MSRB1 | -0.027176251 | 0.00036388 | 0.000868398 |
| CFAP298 | -0.015173095 | 0.000365339 | 0.000871372 |
| ZNF91 | -0.014363892 | 0.000365668 | 0.000871652 |
| SEPHS2 | -0.033487116 | 0.000367311 | 0.000875059 |
| USP14 | -0.014816901 | 0.000368321 | 0.000876955 |
| UBE2H | -0.017956402 | 0.000368708 | 0.000877367 |
| TCEA1 | -0.022516209 | 0.00037449 | 0.000890608 |
| ACAA1 | -0.049529293 | 0.00038168 | 0.00090718 |
| ARL5A | -0.020725291 | 0.000391824 | 0.000930753 |
| CCNC | -0.017528035 | 0.00039415 | 0.000935736 |
| PSMB5 | -0.025923648 | 0.000399137 | 0.000946877 |
| PRR13 | -0.033836897 | 0.000399305 | 0.000946877 |
| CNIH4 | -0.01430491 | 0.000401312 | 0.000951087 |
| SRSF6 | -0.017382833 | 0.000404801 | 0.0009588 |
| RCC1L | -0.012617113 | 0.000406311 | 0.000961821 |
| PRKAR1A | -0.024929733 | 0.000407544 | 0.000964185 |
| ISCA1 | -0.019377064 | 0.000408284 | 0.000965378 |
| FAM177A1 | -0.025105839 | 0.000410551 | 0.000970177 |
| WDR45 | -0.015671267 | 0.000415423 | 0.000980723 |

| | | | |
|---|---|---|---|
| CUEDC2 | -0.01815876 | 0.000415492 | 0.000980723 |
| SMIM12 | -0.013763237 | 0.000418065 | 0.000986229 |
| MEA1 | -0.014322131 | 0.000427641 | 0.00100824 |
| SYPL1 | -0.022333753 | 0.000428674 | 0.001010095 |
| TTC1 | -0.016292221 | 0.000436038 | 0.001026353 |
| DENR | -0.014209333 | 0.000436075 | 0.001026353 |
| SFXN4 | -0.01348727 | 0.000442526 | 0.001040939 |
| TGFBR3 | 0.014318903 | 0.000446099 | 0.001048743 |
| MANBAL | -0.011928393 | 0.000447911 | 0.001052398 |
| LSM7 | -0.021767531 | 0.000451335 | 0.001059836 |
| RAB11FIP5 | -0.015421491 | 0.000452933 | 0.001062979 |
| ACAT1 | 0.043721825 | 0.000455111 | 0.001067479 |
| PPIF | -0.012139116 | 0.000455532 | 0.001067857 |
| ASB8 | -0.013385818 | 0.000460784 | 0.00107955 |
| NDUFA2 | -0.039080354 | 0.000467429 | 0.001094494 |
| DOCK8-AS: | -0.023906145 | 0.000469849 | 0.001099533 |
| TOMM20 | -0.030699573 | 0.000473497 | 0.001106305 |
| TCEAL8 | -0.021238055 | 0.00047353 | 0.001106305 |
| THAP5 | -0.012353556 | 0.000473552 | 0.001106305 |
| GNG12 | -0.021797664 | 0.000485057 | 0.001132536 |
| DALRD3 | -0.01159792 | 0.000485603 | 0.001133165 |
| C15orf40 | -0.015732965 | 0.000490769 | 0.001144096 |
| UQCC2 | -0.022102291 | 0.000490845 | 0.001144096 |
| RPL7L1 | -0.015578554 | 0.000492491 | 0.00114728 |
| SARS | -0.017174461 | 0.000498057 | 0.001159457 |
| FAM172A | 0.012456754 | 0.000498284 | 0.001159457 |
| TRMT112 | -0.035644643 | 0.000499673 | 0.001162029 |
| CKB | -0.018960876 | 0.000500472 | 0.001163228 |
| ZKSCAN1 | -0.014259455 | 0.000506155 | 0.001175772 |
| BBOX1 | -0.048286394 | 0.000507266 | 0.001177684 |
| EIF3M | -0.020411268 | 0.000523403 | 0.00121446 |
| DCDC2 | 0.016370071 | 0.000530373 | 0.001229938 |
| SH3BGRL3 | -0.022655169 | 0.000538509 | 0.001248098 |
| LYRM2 | -0.016744244 | 0.00054012 | 0.001251126 |
| SPAG16 | -0.016026977 | 0.000542067 | 0.001254927 |
| ARID5B | 0.022218045 | 0.00054812 | 0.001268222 |
| CCDC167 | -0.012132634 | 0.000549174 | 0.001269946 |
| DCXR | 0.062265269 | 0.000553993 | 0.001280367 |
| PPP1R1A | -0.016410842 | 0.000557058 | 0.001286725 |
| MAPK6 | -0.012332372 | 0.000562208 | 0.001297891 |
| RPL24 | -0.064853351 | 0.000562766 | 0.001298448 |
| GSTZ1 | -0.013708263 | 0.000564031 | 0.001300634 |
| NUDT5 | -0.015546813 | 0.000568684 | 0.001310095 |
| CLSTN1 | -0.017063241 | 0.000568773 | 0.001310095 |
| TPCN1 | -0.016251747 | 0.000571252 | 0.001315066 |
| CYP4F3 | -0.016883735 | 0.000573303 | 0.001319048 |
| POLD2 | -0.016072149 | 0.000577477 | 0.001327907 |

| | | | |
|---|---|---|---|
| TCN2 | -0.019743464 | 0.000581632 | 0.001336711 |
| CMTM6 | -0.01216009 | 0.000587303 | 0.001348986 |
| UGT2B7 | 0.066525323 | 0.000594563 | 0.001364899 |
| CENPS | -0.014829624 | 0.000599846 | 0.001376257 |
| COA1 | -0.015661706 | 0.000601927 | 0.001380259 |
| GLS | 0.04056737 | 0.000605984 | 0.001388784 |
| SSR1 | -0.014841667 | 0.000606607 | 0.001389435 |
| A4GALT | -0.012237277 | 0.000608472 | 0.001392928 |
| PNPO | -0.019514725 | 0.000610647 | 0.001397127 |
| MRPL47 | -0.016798759 | 0.00061378 | 0.001403513 |
| SF1 | -0.018381085 | 0.000616764 | 0.001409551 |
| NFKBIA | -0.016302172 | 0.000621772 | 0.001420203 |
| TKT | -0.020315669 | 0.000627324 | 0.001432088 |
| ETF1 | -0.012616606 | 0.000639129 | 0.001458225 |
| PLG | -0.029520931 | 0.000646647 | 0.001474556 |
| ACP5 | -0.020987663 | 0.000659218 | 0.001502386 |
| ERBIN | 0.017706382 | 0.000662272 | 0.001508509 |
| HADHB | -0.02570944 | 0.000666061 | 0.001516296 |
| AKR7A3 | -0.034787029 | 0.00067039 | 0.001525306 |
| DDX6 | -0.016952396 | 0.000677878 | 0.001541487 |
| URGCP | 0.011796458 | 0.00068361 | 0.001553659 |
| HOOK1 | -0.01700765 | 0.000687858 | 0.001561818 |
| PTPN13 | 0.013798149 | 0.00068797 | 0.001561818 |
| ILF3-DT | -0.011809174 | 0.000688343 | 0.001561818 |
| EPB41L4A | 0.012461866 | 0.00069822 | 0.001583353 |
| TSR3 | -0.017899399 | 0.000699213 | 0.001584729 |
| HMG20B | -0.015360237 | 0.000709895 | 0.001608048 |
| ARGLU1 | -0.018297467 | 0.000710353 | 0.001608199 |
| MST1 | -0.012019319 | 0.000712812 | 0.001612875 |
| PRPF6 | -0.01316512 | 0.000723567 | 0.001636307 |
| ELF1 | 0.011574929 | 0.000728963 | 0.001647602 |
| DAO | -0.022279898 | 0.000730446 | 0.001650043 |
| GNB1 | -0.019456828 | 0.000733451 | 0.00165592 |
| PIN1 | -0.01790865 | 0.000742052 | 0.001674415 |
| PSMA2 | -0.026966093 | 0.000743871 | 0.001677597 |
| ARPC1B | -0.011602011 | 0.00074496 | 0.00167913 |
| CHL1 | -0.013923744 | 0.000746092 | 0.001680757 |
| PRDX3 | -0.035956358 | 0.000749019 | 0.001686425 |
| DHX36 | -0.013354573 | 0.0007538 | 0.001696257 |
| UFL1 | -0.012828306 | 0.000788548 | 0.001773476 |
| NCBP2AS2 | -0.013623866 | 0.00078955 | 0.001774756 |
| FOXO3 | 0.013147117 | 0.000792195 | 0.001779606 |
| MCEE | -0.014390699 | 0.000792576 | 0.001779606 |
| WTAP | -0.015975318 | 0.000795928 | 0.001786155 |
| UQCRC2 | -0.024069212 | 0.000797313 | 0.001788285 |
| TCP1 | -0.019171492 | 0.000798287 | 0.001789491 |
| PPP6R2 | 0.015856534 | 0.000799275 | 0.001790725 |

| | | | |
|---|---|---|---|
| UBE2B | -0.022350836 | 0.000799771 | 0.001790859 |
| ACOX1 | -0.021397978 | 0.000802886 | 0.001796851 |
| BPHL | -0.028164221 | 0.000812074 | 0.001816423 |
| HIGD2A | -0.029466812 | 0.000822582 | 0.001838923 |
| GIPC2 | 0.029728431 | 0.000823961 | 0.001840139 |
| RPL31 | -0.033113968 | 0.000824023 | 0.001840139 |
| THY1 | 0.037269286 | 0.000834251 | 0.001861964 |
| RERG | 0.015402174 | 0.000845677 | 0.001886439 |
| RRAGA | -0.01648981 | 0.0008492 | 0.001893269 |
| CBY1 | -0.011500203 | 0.00084984 | 0.001893666 |
| RMDN1 | -0.013846477 | 0.000860347 | 0.001916035 |
| SLC22A5 | -0.013796036 | 0.000863669 | 0.001922391 |
| AIMP1 | -0.016337449 | 0.000882194 | 0.001962029 |
| ZNHIT3 | -0.013425036 | 0.000882435 | 0.001962029 |
| UBE2V2 | -0.014199762 | 0.000889867 | 0.001977483 |
| DECR1 | -0.025048632 | 0.000898421 | 0.00199541 |
| NIPSNAP2 | -0.01324933 | 0.000919401 | 0.002040901 |
| GTF2B | -0.011841785 | 0.000920762 | 0.002042816 |
| MBTPS1 | -0.01286659 | 0.000924322 | 0.002049605 |
| QTRT1 | -0.016017057 | 0.000924978 | 0.002049951 |
| JAGN1 | -0.012715414 | 0.000940812 | 0.002083917 |
| THYN1 | -0.012955267 | 0.000946185 | 0.002094686 |
| PAQR7 | -0.016452994 | 0.000951178 | 0.002104603 |
| SNRNP200 | -0.01380508 | 0.000961484 | 0.002126258 |
| PPIL4 | -0.010989658 | 0.000973383 | 0.002151413 |
| STK25 | -0.014367758 | 0.000975317 | 0.002154525 |
| SYNRG | -0.012131966 | 0.000980488 | 0.002164781 |
| KLF13 | -0.014883571 | 0.000987851 | 0.002179865 |
| DNAJB9 | -0.014385969 | 0.0009916 | 0.002186223 |
| COA3 | -0.026209183 | 0.000991799 | 0.002186223 |
| TRAPPC1 | -0.022722654 | 0.000995615 | 0.002193456 |
| RALGAPA1 | 0.010817448 | 0.000996186 | 0.002193535 |
| UPB1 | -0.017649471 | 0.001006049 | 0.002214064 |
| IQGAP1 | -0.011084904 | 0.001014066 | 0.00223051 |
| ZNF638 | 0.015390765 | 0.001017636 | 0.002237163 |
| MMUT | -0.014429003 | 0.001023309 | 0.002248428 |
| NOX4 | 0.027974136 | 0.00102606 | 0.002253266 |
| LEPROTL1 | -0.013054205 | 0.001035981 | 0.002273834 |
| COL18A1 | -0.024160197 | 0.001052248 | 0.002308302 |
| FYTTD1 | -0.011059304 | 0.001055509 | 0.002313869 |
| DNAJC4 | -0.014307057 | 0.001055914 | 0.002313869 |
| TMBIM6 | -0.052715106 | 0.001059171 | 0.00231909 |
| DDIT3 | -0.011937865 | 0.001059428 | 0.00231909 |
| TCEA2 | -0.017264374 | 0.001071388 | 0.002344019 |
| CLPTM1 | -0.016956922 | 0.001083537 | 0.002369334 |
| RBM4 | -0.017981532 | 0.00108541 | 0.002372164 |
| AURKAIP1 | -0.034260174 | 0.001087553 | 0.002375582 |

| | | | |
|---|---|---|---|
| PLEKHA3 | -0.011457787 | 0.001088968 | 0.002376781 |
| PFKL | -0.02296233 | 0.001089261 | 0.002376781 |
| NRIP1 | 0.018207672 | 0.00109219 | 0.002381903 |
| TRPM7 | 0.012129641 | 0.001095404 | 0.002387643 |
| FAM174C | -0.019095787 | 0.001096551 | 0.002388872 |
| NSL1 | -0.011116616 | 0.001098025 | 0.002390814 |
| BOD1L1 | -0.010885273 | 0.001099907 | 0.002393641 |
| ANAPC13 | -0.02054 | 0.001116172 | 0.002427749 |
| SARNP | -0.013805622 | 0.001117175 | 0.002428641 |
| SSNA1 | -0.02162444 | 0.001152511 | 0.00250413 |
| MAGT1 | -0.012016488 | 0.001155044 | 0.002508277 |
| MRPL40 | -0.021261728 | 0.001155643 | 0.002508277 |
| HSPA8 | -0.038766334 | 0.001166498 | 0.002530498 |
| RHOBTB1 | 0.017672015 | 0.001177342 | 0.002552672 |
| AAMDC | -0.016120825 | 0.001182932 | 0.002563436 |
| HERC4 | 0.010781284 | 0.001189771 | 0.002576894 |
| GATD3B | -0.023232969 | 0.001194687 | 0.002586175 |
| ECI1 | -0.02424556 | 0.001199085 | 0.002594326 |
| TMEM259 | -0.011759517 | 0.00120807 | 0.002612387 |
| NDUFB8 | -0.03464436 | 0.001208741 | 0.002612461 |
| ADH6 | -0.017627928 | 0.001217919 | 0.00263091 |
| ABLIM1 | 0.016973389 | 0.001229384 | 0.002654279 |
| SRSF8 | -0.014871266 | 0.001231031 | 0.002656435 |
| ALDH6A1 | 0.051352014 | 0.001238777 | 0.002671744 |
| ENO1 | -0.038927364 | 0.001241624 | 0.002676476 |
| CASC4 | -0.014754349 | 0.001252693 | 0.002698919 |
| NUDC | -0.019607115 | 0.001269123 | 0.002732881 |
| ATP6V1H | -0.013361597 | 0.001276012 | 0.002746271 |
| PPP2R5C | -0.015405064 | 0.001294612 | 0.002784843 |
| TOX4 | -0.01116396 | 0.001304949 | 0.002805606 |
| RAP1B | -0.018140685 | 0.001309412 | 0.002813725 |
| MAN1A1 | 0.014123907 | 0.001319744 | 0.002834441 |
| C14orf119 | -0.013008577 | 0.00132405 | 0.002842201 |
| NFAT5 | 0.012413402 | 0.001333304 | 0.002860569 |
| ADIPOR1 | -0.012986005 | 0.001337723 | 0.002868548 |
| KIAA0232 | -0.012082225 | 0.00135577 | 0.002905728 |
| AGPAT5 | -0.010751953 | 0.001363522 | 0.002920816 |
| RDH10 | -0.012943466 | 0.001367829 | 0.002928512 |
| RBMX | -0.015737583 | 0.001395821 | 0.002986883 |
| TM4SF5 | 0.011981286 | 0.001409058 | 0.00301239 |
| NEO1 | -0.011908476 | 0.001409794 | 0.00301239 |
| NFE2L1 | -0.019325167 | 0.001409946 | 0.00301239 |
| HPN | -0.024720623 | 0.001415699 | 0.003023107 |
| DYNC1I2 | -0.018453063 | 0.001422187 | 0.003035381 |
| SRSF10 | -0.014413045 | 0.001431591 | 0.003053863 |
| ADRM1 | -0.015483794 | 0.001435665 | 0.00306096 |
| USP16 | -0.011610571 | 0.001467372 | 0.003126936 |

| | | | |
|---|---|---|---|
| CTXN3 | -0.031572335 | 0.001470215 | 0.003131367 |
| WDR1 | -0.015157458 | 0.00148824 | 0.003168112 |
| MAPK10 | 0.014464204 | 0.001499789 | 0.00319104 |
| JOSD2 | -0.013140539 | 0.001513614 | 0.003218784 |
| RERE | 0.017059528 | 0.001521673 | 0.003234245 |
| FNTA | -0.011767844 | 0.001548428 | 0.003289407 |
| APIP | -0.013490746 | 0.0015534 | 0.003298261 |
| FASTK | -0.013550521 | 0.001569631 | 0.003330998 |
| MRPL44 | -0.015022041 | 0.001579308 | 0.003349799 |
| MRPS24 | -0.027769717 | 0.001599422 | 0.003389989 |
| GLO1 | -0.016559211 | 0.00159991 | 0.003389989 |
| DGCR6L | -0.019154794 | 0.001603742 | 0.003396354 |
| PAXX | -0.01257482 | 0.001643075 | 0.003477261 |
| RAB11FIP3 | 0.035249872 | 0.001643642 | 0.003477261 |
| GGNBP2 | -0.01269848 | 0.001654675 | 0.003498797 |
| CBX1 | -0.01100438 | 0.001657296 | 0.003502533 |
| CCDC124 | -0.013303522 | 0.001661633 | 0.00350989 |
| SLC48A1 | -0.009907503 | 0.00166691 | 0.003519223 |
| SUCLG1 | -0.062493724 | 0.001671242 | 0.003526552 |
| EMC6 | -0.017501249 | 0.001678624 | 0.003540309 |
| PSMG1 | -0.013573921 | 0.001685204 | 0.003552357 |
| THNSL2 | -0.013935016 | 0.001692043 | 0.003564942 |
| TMEM192 | -0.015199476 | 0.001698204 | 0.003576084 |
| DAZAP2 | 0.023514361 | 0.001714177 | 0.003607867 |
| SUGCT | 0.018039883 | 0.001733995 | 0.003647706 |
| FBXO11 | 0.010726036 | 0.001738149 | 0.00365457 |
| ANTKMT | -0.01954921 | 0.001741045 | 0.003658783 |
| FDX2 | -0.012767678 | 0.00174477 | 0.003664731 |
| RPL18 | -0.052653055 | 0.001794387 | 0.003767019 |
| SLC25A3 | -0.040310122 | 0.001797168 | 0.003770925 |
| ATF7IP | 0.011293477 | 0.001809822 | 0.003795536 |
| AMT | -0.011684614 | 0.001812692 | 0.00379961 |
| AK4 | 0.041301413 | 0.001813896 | 0.003800191 |
| ZBTB16 | -0.013058769 | 0.00181536 | 0.003801315 |
| CD164 | -0.026268611 | 0.00182284 | 0.00381503 |
| BLCAP | -0.012287103 | 0.001839045 | 0.003846981 |
| PRSS8 | -0.010384161 | 0.001848709 | 0.003865226 |
| CHMP4B | -0.013401953 | 0.001890799 | 0.003951211 |
| TPI1 | -0.039242959 | 0.001905783 | 0.003980493 |
| MYOM3 | -0.011335357 | 0.001911479 | 0.003990183 |
| AAK1 | -0.017509345 | 0.001912368 | 0.003990183 |
| GAREM1 | 0.012591729 | 0.00191427 | 0.003992119 |
| FYN | -0.012887932 | 0.001919362 | 0.004000705 |
| HNRNPC | -0.022377028 | 0.0019411 | 0.004043959 |
| NUDT3 | -0.010302331 | 0.001948014 | 0.004056301 |
| BIRC6 | 0.014096068 | 0.001963366 | 0.004086193 |
| CDIP1 | -0.013923692 | 0.001970995 | 0.004099989 |

| | | | |
|---|---|---|---|
| SMARCB1 | -0.011174702 | 0.001974301 | 0.004104783 |
| SNX6 | -0.015679827 | 0.002031775 | 0.004222138 |
| SRP72 | -0.012675497 | 0.002058299 | 0.004275088 |
| OSER1 | -0.010318204 | 0.00207491 | 0.004307407 |
| BZW1 | -0.019907025 | 0.002081291 | 0.004318467 |
| N4BP2L2 | -0.022781944 | 0.002083459 | 0.004320779 |
| SPIN1 | 0.015502118 | 0.002087064 | 0.004326067 |
| AL138826. | 0.025848861 | 0.00209695 | 0.004344364 |
| FBXW5 | -0.018157204 | 0.00211433 | 0.004378158 |
| GALE | -0.011659826 | 0.002120724 | 0.004389182 |
| XRCC6 | -0.017168038 | 0.002122791 | 0.004391243 |
| KCNJ15 | 0.034463112 | 0.002131424 | 0.004406877 |
| NFIB | 0.024778642 | 0.002135925 | 0.004413958 |
| RPL13A | 0.041275092 | 0.002145786 | 0.004432102 |
| LINC00671 | -0.019916112 | 0.00215426 | 0.004447365 |
| SNU13 | -0.023546293 | 0.002162589 | 0.004462312 |
| PDZK1IP1 | -0.055690872 | 0.002177967 | 0.004491783 |
| ASPA | 0.016196163 | 0.002194527 | 0.004523661 |
| MAOA | -0.024240984 | 0.002199464 | 0.004530619 |
| C15orf61 | -0.013498804 | 0.002200113 | 0.004530619 |
| AP2A1 | -0.012786251 | 0.002202003 | 0.004532235 |
| LY6E | -0.020863018 | 0.002205198 | 0.004536534 |
| TAF7 | -0.013780327 | 0.002217037 | 0.004558602 |
| MAGOH | -0.016034008 | 0.002226559 | 0.004575886 |
| SDR39U1 | -0.011572239 | 0.002236891 | 0.004594815 |
| RTCA | -0.014467639 | 0.00225154 | 0.004621507 |
| MRPS17 | -0.011405643 | 0.002252139 | 0.004621507 |
| IAH1 | -0.013682008 | 0.002255479 | 0.004626044 |
| ADM2 | -0.011769878 | 0.002263194 | 0.004638462 |
| TRAPPC6A | -0.019778781 | 0.002263796 | 0.004638462 |
| RTCB | -0.014978462 | 0.002272905 | 0.004654801 |
| MCTS1 | -0.012915967 | 0.002290233 | 0.004687947 |
| FIBP | -0.016036855 | 0.002296782 | 0.004699006 |
| GPX4 | -0.049929166 | 0.002310595 | 0.004723095 |
| TAB2 | 0.010994394 | 0.002311987 | 0.004723095 |
| PSMC2 | -0.01269846 | 0.002312013 | 0.004723095 |
| ENTPD5 | -0.017346301 | 0.002327685 | 0.004752743 |
| TMEM70 | -0.011734922 | 0.002330119 | 0.004755344 |
| MKRN1 | -0.010536397 | 0.002353959 | 0.004801608 |
| CCT6A | -0.019265775 | 0.002365557 | 0.004821423 |
| SUMO3 | -0.014954594 | 0.002366025 | 0.004821423 |
| ANP32A | -0.016407522 | 0.002411983 | 0.004911634 |
| ZBTB8OS | -0.013079568 | 0.00241269 | 0.004911634 |
| USE1 | 0.01330176 | 0.002417863 | 0.004919722 |
| MRPL4 | -0.015110197 | 0.002453024 | 0.004988788 |
| NUMB | 0.014441242 | 0.002457331 | 0.004995071 |
| PGD | -0.011076195 | 0.002472569 | 0.005023555 |

| | | | |
|---|---|---|---|
| JMJD8 | -0.012728733 | 0.002505518 | 0.005087975 |
| AC244090. | -0.012076797 | 0.002514831 | 0.005104361 |
| PPP1R16A | 0.03196215 | 0.002524475 | 0.005120803 |
| ATP2C1 | -0.009754536 | 0.00252543 | 0.005120803 |
| OTUD6B-A | -0.016169995 | 0.002545418 | 0.005158782 |
| EIF5B | -0.014674902 | 0.002580225 | 0.00522674 |
| URM1 | -0.010778705 | 0.002594042 | 0.005252135 |
| PCMTD2 | -0.00996538 | 0.002599204 | 0.005259988 |
| DYNLL2 | 0.018930467 | 0.002601263 | 0.005261557 |
| ANXA6 | -0.012528226 | 0.002607593 | 0.005271762 |
| ZNF385B | 0.013080069 | 0.002618742 | 0.005291692 |
| SUSD6 | -0.010025225 | 0.002629319 | 0.005310447 |
| BCKDK | -0.012529325 | 0.002665748 | 0.005381373 |
| MRPL11 | -0.014771028 | 0.002672697 | 0.005392745 |
| FGD4 | 0.012834403 | 0.002677354 | 0.005399485 |
| DIP2C | 0.014232087 | 0.002682636 | 0.005407477 |
| CLRN3 | -0.012570303 | 0.00268895 | 0.005415615 |
| COMMD4 | -0.015772158 | 0.002689315 | 0.005415615 |
| HSPA4L | -0.011596623 | 0.002694925 | 0.005424247 |
| LITAF | -0.01115702 | 0.00271292 | 0.005456027 |
| PPP2R2D | -0.010944983 | 0.002713375 | 0.005456027 |
| STT3B | -0.011072355 | 0.002719435 | 0.005465532 |
| TMEM126. | -0.012412877 | 0.002732635 | 0.005489371 |
| RPS21 | -0.057863113 | 0.002748875 | 0.005519289 |
| TMEM14A | -0.012990139 | 0.00275587 | 0.005530626 |
| CYREN | -0.010038684 | 0.002757525 | 0.005531238 |
| MIOX | 0.075850016 | 0.002782222 | 0.005578049 |
| CLNS1A | -0.013581665 | 0.002805662 | 0.005621164 |
| MRPL16 | -0.013498774 | 0.002806469 | 0.005621164 |
| HOGA1 | 0.0173896 | 0.002830115 | 0.005665758 |
| PHC2 | -0.011653543 | 0.002842295 | 0.005687364 |
| ATP5MF | -0.038165318 | 0.002859091 | 0.005718182 |
| HAX1 | -0.015115582 | 0.002866556 | 0.005730317 |
| RETREG2 | -0.01256998 | 0.00288555 | 0.005764582 |
| IMP4 | -0.01287274 | 0.002886509 | 0.005764582 |
| CDK10 | -0.01334778 | 0.002942466 | 0.005873472 |
| SREK1 | -0.010003978 | 0.002985454 | 0.005956381 |
| PBX1 | 0.01727752 | 0.002995525 | 0.005973566 |
| SDCBP | -0.015460105 | 0.003023718 | 0.006026857 |
| KIF5B | -0.013259051 | 0.003045739 | 0.006067799 |
| COA4 | -0.022747756 | 0.003078483 | 0.006130054 |
| WWC1 | 0.013831893 | 0.003083338 | 0.006136741 |
| SUCLG2 | -0.02424252 | 0.003090736 | 0.00614848 |
| STRN3 | 0.011041882 | 0.003096551 | 0.00615706 |
| GK | 0.027716282 | 0.003100223 | 0.006161373 |
| HSPB1 | 0.034584809 | 0.003102819 | 0.006163546 |
| ARFGEF2 | 0.010029987 | 0.00310646 | 0.00616779 |

| | | | |
|---|---|---|---|
| MMAB | -0.014705235 | 0.003115223 | 0.006182195 |
| ENPEP | 0.025986935 | 0.003120399 | 0.00618947 |
| DYNLRB1 | 0.03237858 | 0.003163714 | 0.006272354 |
| NME4 | -0.011694504 | 0.003205946 | 0.00635301 |
| HDHD3 | -0.019444775 | 0.003231296 | 0.006400151 |
| H1FX | -0.013613112 | 0.003238801 | 0.006411919 |
| MRPL28 | -0.015923514 | 0.00325191 | 0.006434763 |
| MED4 | -0.010809009 | 0.003263372 | 0.006454329 |
| TXNDC5 | -0.009556463 | 0.003265123 | 0.006454679 |
| GLUL | -0.013383207 | 0.003280585 | 0.006482119 |
| SPAG7 | -0.01617695 | 0.003304599 | 0.006524307 |
| SF3B4 | -0.010174473 | 0.003305118 | 0.006524307 |
| OSBP | -0.012180145 | 0.00331696 | 0.006544532 |
| RPS29 | -0.055700507 | 0.003319512 | 0.006546417 |
| NF1 | 0.013084899 | 0.003338854 | 0.006581395 |
| FREM2 | 0.01308824 | 0.003348648 | 0.006597529 |
| LDHB | -0.048273753 | 0.003350542 | 0.00659809 |
| IDI1 | -0.011226859 | 0.00335555 | 0.00660478 |
| HSDL2 | -0.016333684 | 0.003361103 | 0.006612535 |
| PCBP1 | 0.029103193 | 0.003377648 | 0.006641897 |
| SLC25A42 | -0.014049257 | 0.003382472 | 0.006648195 |
| AFG3L2 | -0.015415057 | 0.003407955 | 0.006695073 |
| PSME2 | -0.017447702 | 0.003410846 | 0.006697542 |
| RPS17 | 0.034537454 | 0.003412931 | 0.006698429 |
| COX6B1 | -0.040129499 | 0.00342675 | 0.006722332 |
| GOLGA7 | -0.014702261 | 0.003444337 | 0.006753603 |
| TAX1BP3 | -0.012116087 | 0.003448584 | 0.006758698 |
| OARD1 | -0.013664438 | 0.003466436 | 0.006790439 |
| CAPN1 | -0.010180791 | 0.003480352 | 0.006814443 |
| PFDN1 | -0.01350149 | 0.003484459 | 0.006818822 |
| PAPLN | -0.011298865 | 0.003485915 | 0.006818822 |
| SNHG32 | -0.014213613 | 0.003541709 | 0.006924658 |
| PEX2 | -0.011695556 | 0.003551391 | 0.006938606 |
| RHOA | -0.021666773 | 0.003552228 | 0.006938606 |
| TPD52L2 | -0.009534795 | 0.003555275 | 0.006941251 |
| TMPO | -0.010372074 | 0.003574867 | 0.006973291 |
| ANXA7 | -0.020579797 | 0.003575087 | 0.006973291 |
| RBM6 | 0.010395813 | 0.003594614 | 0.007008044 |
| INO80C | -0.010324164 | 0.00359671 | 0.007008797 |
| RBM25 | -0.011018475 | 0.003602936 | 0.007017595 |
| NUDT22 | -0.015638857 | 0.003616948 | 0.007041542 |
| TRPT1 | -0.0120403 | 0.003619181 | 0.00704207 |
| GBE1 | 0.011907732 | 0.003620654 | 0.00704207 |
| MRPL12 | -0.018579402 | 0.003630084 | 0.007057063 |
| SNX9 | -0.012025901 | 0.003669537 | 0.00713038 |
| SLC25A36 | 0.017912381 | 0.003680959 | 0.007149187 |
| PFDN5 | -0.043684625 | 0.003688436 | 0.007160316 |

| | | | |
|---|---|---|---|
| INTS10 | -0.010284758 | 0.003710726 | 0.007200179 |
| SEC13 | -0.012089017 | 0.003723227 | 0.007221017 |
| LBR | -0.010906013 | 0.003742595 | 0.007255149 |
| HIPK3 | 0.01632049 | 0.003758926 | 0.007283363 |
| ARF3 | -0.015190187 | 0.003761801 | 0.00728549 |
| SIL1 | -0.01020034 | 0.003764169 | 0.007286634 |
| MAEA | -0.009703363 | 0.003778236 | 0.007310415 |
| C9orf78 | -0.015363894 | 0.003792211 | 0.007333994 |
| ARHGEF10 | -0.010495346 | 0.003856544 | 0.007454895 |
| BOD1 | -0.01061139 | 0.003903183 | 0.007541494 |
| CNKSR3 | 0.013736791 | 0.003916411 | 0.007563488 |
| CIAO2A | -0.01660818 | 0.003938325 | 0.007602228 |
| ECI2 | -0.029393354 | 0.003966129 | 0.007652296 |
| HAGH | -0.028295802 | 0.003981629 | 0.007678589 |
| SCOC | -0.017697215 | 0.004027032 | 0.007762498 |
| MACROD1 | 0.025063642 | 0.00411204 | 0.007922633 |
| STOML2 | -0.020156423 | 0.004134604 | 0.007962366 |
| MRPL19 | -0.016253074 | 0.004138493 | 0.007966113 |
| SCP2 | -0.031297525 | 0.004165463 | 0.008014264 |
| CDK2AP2 | -0.013970726 | 0.00417149 | 0.008022097 |
| PSMA3-AS | -0.009566315 | 0.004227164 | 0.00812535 |
| PPP4C | -0.01511986 | 0.004247022 | 0.008159695 |
| PHGDH | 0.022321321 | 0.004304998 | 0.008267209 |
| TMEM248 | -0.011490303 | 0.004337898 | 0.00832649 |
| NFIA | 0.023094768 | 0.004396178 | 0.008434409 |
| TRIP6 | -0.016986698 | 0.00443961 | 0.008513753 |
| ABCD4 | -0.009722772 | 0.004453169 | 0.00853576 |
| SLC22A18A | -0.011783376 | 0.004462639 | 0.008549916 |
| CAPZA1 | -0.011310926 | 0.004468033 | 0.008556252 |
| SLC40A1 | -0.01677398 | 0.004491741 | 0.008597636 |
| TBCA | -0.030728134 | 0.004498573 | 0.008606695 |
| ISOC1 | -0.010337212 | 0.004597913 | 0.008792651 |
| EPB41L5 | 0.011368469 | 0.004603913 | 0.00880002 |
| SPRYD4 | -0.010528784 | 0.004630173 | 0.00884609 |
| SDHAF1 | -0.009172544 | 0.004633999 | 0.008848902 |
| SOS2 | 0.011298636 | 0.004635961 | 0.008848902 |
| PIAS1 | 0.009684159 | 0.004652119 | 0.008875611 |
| PTRHD1 | -0.013348623 | 0.004660204 | 0.008886901 |
| SMAD5 | 0.01346235 | 0.004666978 | 0.008895681 |
| RPP25L | -0.010358033 | 0.004682661 | 0.008921019 |
| ADI1 | -0.04128258 | 0.004684623 | 0.008921019 |
| GTPBP6 | -0.014536584 | 0.004689017 | 0.008925241 |
| KIAA1109 | 0.01225931 | 0.004694163 | 0.008930891 |
| CTDSPL | -0.011208366 | 0.004717059 | 0.008970288 |
| MRPS25 | -0.015335425 | 0.004723122 | 0.008974718 |
| MARCH5 | -0.010123393 | 0.004723766 | 0.008974718 |
| LYPLAL1 | 0.0226576 | 0.004750162 | 0.009020688 |

| | | | |
|---|---|---|---|
| SYNE2 | -0.026200072 | 0.004760091 | 0.009035358 |
| HSBP1L1 | -0.013052233 | 0.004783955 | 0.009076453 |
| PNPLA4 | -0.013885991 | 0.004790069 | 0.00908385 |
| CANX | -0.025424672 | 0.004828741 | 0.009152953 |
| C3orf85 | -0.017228225 | 0.00485064 | 0.009190213 |
| CCNG2 | -0.012596159 | 0.004859073 | 0.00920194 |
| MITF | 0.011054383 | 0.004891915 | 0.009259857 |
| TPRG1L | -0.014707248 | 0.004909692 | 0.009289218 |
| ZCCHC14 | 0.009641375 | 0.004913182 | 0.009291535 |
| PSMD1 | -0.010960771 | 0.00498877 | 0.009430132 |
| NBEAL1 | 0.010876609 | 0.005008781 | 0.009463596 |
| PHLDB2 | 0.011844327 | 0.005024477 | 0.009488879 |
| CCDC50 | -0.012254434 | 0.005034068 | 0.009502615 |
| LIMS1 | -0.011372378 | 0.005047094 | 0.009522818 |
| KLHDC2 | -0.017154248 | 0.005061276 | 0.009542863 |
| MSRA | -0.031318041 | 0.005062372 | 0.009542863 |
| BRD2 | -0.014087109 | 0.00510554 | 0.009619814 |
| CCT3 | -0.020038322 | 0.005129401 | 0.009660332 |
| TGIF1 | -0.010514281 | 0.005272226 | 0.009922526 |
| RUFY1 | -0.009248443 | 0.00527346 | 0.009922526 |
| SLC25A30 | 0.01061906 | 0.005288489 | 0.00994624 |
| PITHD1 | -0.009960363 | 0.005305868 | 0.00997435 |
| SUMF1 | 0.008827712 | 0.00531566 | 0.009988179 |
| CHMP1B | -0.0109835 | 0.005350008 | 0.010043734 |
| SF3B2 | -0.013407805 | 0.005350126 | 0.010043734 |
| PTOV1 | -0.014451259 | 0.005356227 | 0.010050585 |
| MEAF6 | -0.012511512 | 0.005378103 | 0.010087018 |
| AHNAK | -0.018796118 | 0.005454849 | 0.010226283 |
| NIFK | -0.009339962 | 0.005458516 | 0.01022848 |
| IARS2 | -0.011654908 | 0.005509734 | 0.01031974 |
| RFXANK | -0.009889445 | 0.005519822 | 0.010333913 |
| AGGF1 | -0.010037121 | 0.005639017 | 0.010552245 |
| ATN1 | -0.009462478 | 0.005662615 | 0.010587084 |
| R3HCC1 | -0.009978741 | 0.005662799 | 0.010587084 |
| CMYA5 | -0.010435576 | 0.005676692 | 0.010608221 |
| GNA11 | -0.01136586 | 0.005717539 | 0.010679685 |
| NCOA4 | -0.017525359 | 0.005726194 | 0.010690982 |
| TUBB4B | -0.027522692 | 0.005737648 | 0.01070749 |
| IFT43 | -0.012805993 | 0.005742709 | 0.01071206 |
| PLCXD2 | -0.015098427 | 0.005795042 | 0.010804762 |
| SLC25A10 | -0.018473099 | 0.005823324 | 0.010852559 |
| XRN2 | -0.0128294 | 0.005850919 | 0.010899031 |
| NCOA7 | 0.017158095 | 0.005856871 | 0.010905165 |
| SOS1 | 0.008964968 | 0.005907862 | 0.010995113 |
| YIPF4 | -0.011267702 | 0.005917682 | 0.011008392 |
| KANSL1 | 0.010710196 | 0.005932181 | 0.011028393 |
| GCDH | 0.01152741 | 0.005933813 | 0.011028393 |

| | | | |
|---|---|---|---|
| RUVBL1 | -0.009865825 | 0.005939451 | 0.011029044 |
| ANAPC15 | -0.012928286 | 0.005939544 | 0.011029044 |
| HDDC3 | -0.009918245 | 0.005967683 | 0.011076279 |
| RPRD2 | 0.011036811 | 0.005985735 | 0.011104757 |
| UBE2E3 | -0.014667171 | 0.006016008 | 0.011155872 |
| MORF4L2 | -0.0165878 | 0.006055655 | 0.011224316 |
| ABHD10 | -0.014091411 | 0.006091543 | 0.011283812 |
| ENPP2 | -0.010992835 | 0.006093259 | 0.011283812 |
| IK | -0.012906503 | 0.00617258 | 0.011425543 |
| ACO1 | -0.024661809 | 0.006234299 | 0.011534579 |
| C11orf1 | -0.015253107 | 0.006318702 | 0.011685466 |
| DPP4 | -0.020034054 | 0.006350826 | 0.011739579 |
| TACO1 | -0.010825569 | 0.006377412 | 0.011783412 |
| P3H2 | 0.010617444 | 0.006395387 | 0.0118113 |
| RAB11B | -0.013803613 | 0.006464047 | 0.011932729 |
| COX5A | -0.025859464 | 0.006566741 | 0.012116849 |
| GSTM4 | -0.011302946 | 0.006616427 | 0.012203037 |
| RBM47 | 0.021574405 | 0.006649284 | 0.012254373 |
| CREB3 | -0.009261531 | 0.006650239 | 0.012254373 |
| COPS7A | -0.010207568 | 0.0066537 | 0.012255243 |
| PATJ | 0.018328572 | 0.006663413 | 0.012267622 |
| RNF167 | -0.0095353 | 0.006679101 | 0.012290984 |
| HERC1 | 0.008714542 | 0.006742647 | 0.012402356 |
| UCHL3 | -0.009860631 | 0.006763003 | 0.012434221 |
| IMPACT | -0.009479494 | 0.006767698 | 0.012437276 |
| DR1 | -0.009242968 | 0.006819497 | 0.012526854 |
| ZFP36L1 | -0.02567853 | 0.006853408 | 0.012583507 |
| CSTB | -0.024426369 | 0.006883057 | 0.01263229 |
| CYB5B | -0.009791018 | 0.006910122 | 0.012676286 |
| PSMF1 | -0.01082066 | 0.006927235 | 0.012701996 |
| BIVM | -0.00932506 | 0.006941192 | 0.012721899 |
| SLC2A2 | 0.014146089 | 0.006954097 | 0.012739855 |
| SGK1 | -0.011566398 | 0.007033269 | 0.012879143 |
| SIKE1 | -0.010099174 | 0.007077911 | 0.012955105 |
| TXNL1 | -0.015829103 | 0.007123386 | 0.013032522 |
| USP34 | 0.010908487 | 0.007137988 | 0.013053412 |
| AHSA1 | -0.012277425 | 0.00714491 | 0.013060246 |
| GATAD2A | -0.009144622 | 0.007170576 | 0.01310132 |
| EIF4A3 | -0.010230624 | 0.007181409 | 0.013115268 |
| EEF2 | -0.031984508 | 0.007200281 | 0.013143878 |
| KMT2A | -0.010182262 | 0.007223593 | 0.013180566 |
| TMEM33 | -0.010866304 | 0.007273743 | 0.013266168 |
| SGTA | -0.009479562 | 0.007299009 | 0.01330633 |
| TMEM245 | 0.012525258 | 0.007319352 | 0.013337485 |
| PMPCB | -0.013307314 | 0.007346513 | 0.013381032 |
| HNRNPDL | -0.019012504 | 0.007359593 | 0.013398904 |
| POLR1D | -0.0169551 | 0.007364985 | 0.013402769 |

| | | | |
|---|---|---|---|
| ARPC1A | -0.01398263 | 0.007379691 | 0.013423572 |
| CPD | -0.009063572 | 0.007404329 | 0.013462416 |
| DMAC1 | -0.013608155 | 0.007422636 | 0.013489719 |
| TNFRSF21 | -0.011792453 | 0.007469318 | 0.013565803 |
| AC087482. | -0.013111591 | 0.007471118 | 0.013565803 |
| SURF2 | -0.010724734 | 0.00753176 | 0.013669861 |
| CCS | -0.015315375 | 0.007604885 | 0.013796472 |
| PAX2 | -0.013040285 | 0.007647906 | 0.013868383 |
| DDX46 | -0.010727102 | 0.007666587 | 0.013895844 |
| SYNE1 | 0.014526643 | 0.00767251 | 0.013895844 |
| LGMN | -0.023817151 | 0.007673217 | 0.013895844 |
| C1QBP | -0.023570416 | 0.007682498 | 0.013906508 |
| WLS | -0.013352299 | 0.007691079 | 0.013915898 |
| SREK1IP1 | -0.009420533 | 0.007783962 | 0.014073201 |
| CDK4 | -0.011529015 | 0.007784883 | 0.014073201 |
| GPT2 | -0.010074271 | 0.007790899 | 0.014074468 |
| KCMF1 | -0.009607449 | 0.007792449 | 0.014074468 |
| RPL36A | -0.040307592 | 0.007810363 | 0.014100611 |
| NMRK1 | -0.015033377 | 0.007834044 | 0.014137139 |
| RNF10 | -0.012411282 | 0.007842773 | 0.014146664 |
| MDM4 | -0.010071349 | 0.007877658 | 0.01420334 |
| CFAP410 | -0.009274226 | 0.007884164 | 0.014208824 |
| CDH2 | -0.014522698 | 0.007905009 | 0.014240131 |
| EXOC7 | -0.009191662 | 0.007980233 | 0.014366748 |
| TNFRSF11I | -0.015108431 | 0.007985322 | 0.014366748 |
| ALPK2 | -0.01033516 | 0.007985809 | 0.014366748 |
| LSM2 | -0.011132799 | 0.007990145 | 0.014368243 |
| MRPS11 | -0.012620422 | 0.007996121 | 0.014372685 |
| RGL1 | 0.011441681 | 0.008054473 | 0.014471227 |
| ZGPAT | 0.023174221 | 0.008061782 | 0.014478014 |
| NR2F2 | -0.01667244 | 0.008066318 | 0.014479817 |
| FUT6 | 0.011541323 | 0.008155273 | 0.014633093 |
| PPP1R21 | 0.012041082 | 0.008230371 | 0.014758812 |
| SSX2IP | -0.010203171 | 0.008232538 | 0.014758812 |
| FAAP20 | -0.017174431 | 0.008267171 | 0.014814423 |
| ILF3 | -0.011571818 | 0.008382128 | 0.01501386 |
| MCAT | -0.011449727 | 0.008418625 | 0.015072647 |
| LMO7 | -0.010162034 | 0.008446105 | 0.015115247 |
| CSNK2A1 | -0.01277883 | 0.008505167 | 0.01520637 |
| ANKHD1 | 0.010921809 | 0.008507393 | 0.01520637 |
| RPL34 | -0.058617895 | 0.008508149 | 0.01520637 |
| MAP2K3 | -0.009638468 | 0.0085292 | 0.015237351 |
| COL27A1 | -0.008805521 | 0.008556619 | 0.015279677 |
| ZNF580 | -0.010798068 | 0.008585303 | 0.015324224 |
| ZBED5-AS1 | -0.009871041 | 0.008613085 | 0.015367123 |
| NCOR1 | -0.011736175 | 0.008624806 | 0.015376159 |
| PSMD14 | -0.01110561 | 0.00862565 | 0.015376159 |

| | | | |
|---|---|---|---|
| ARL2BP | -0.010143193 | 0.008676823 | 0.015460658 |
| AC118549. | 0.009411084 | 0.008687365 | 0.015472717 |
| PPP1R2 | -0.010302584 | 0.008698225 | 0.015485333 |
| PTBP3 | 0.011595389 | 0.008731563 | 0.015537937 |
| EIF3K | -0.029668255 | 0.008790778 | 0.015636525 |
| MAGI2-AS: | -0.009671811 | 0.00887262 | 0.015775257 |
| REEP6 | -0.011471635 | 0.008920727 | 0.015853914 |
| DNAJC7 | -0.013140929 | 0.009037862 | 0.016055128 |
| SMIM27 | -0.010705581 | 0.009065188 | 0.016096696 |
| ELOB | -0.035275276 | 0.009111012 | 0.016171061 |
| NCBP2 | -0.010252236 | 0.009121941 | 0.016183453 |
| SFXN1 | -0.015229016 | 0.009269786 | 0.016438635 |
| TBX2 | -0.011121621 | 0.009298363 | 0.016482183 |
| CD2BP2 | -0.010348567 | 0.009304725 | 0.016486333 |
| KPNA6 | -0.008542214 | 0.00931133 | 0.016490908 |
| RBBP7 | -0.011499635 | 0.009332464 | 0.016521201 |
| KLF6 | 0.011521391 | 0.009476782 | 0.016769446 |
| RETREG1 | -0.011261178 | 0.00950158 | 0.016806073 |
| SEC23B | -0.008646432 | 0.009511927 | 0.016811886 |
| CRIPT | -0.012298844 | 0.009513067 | 0.016811886 |
| ZNF106 | -0.01046889 | 0.009545488 | 0.016861914 |
| PLPP3 | -0.009144015 | 0.009587462 | 0.016928767 |
| LSM5 | -0.016466044 | 0.009608187 | 0.016958058 |
| XPC | -0.010131862 | 0.009642241 | 0.017010839 |
| PSMC1 | -0.012504463 | 0.009722532 | 0.017145111 |
| RABL6 | -0.012216053 | 0.009741081 | 0.017170435 |
| BCAR1 | -0.009830493 | 0.009897687 | 0.017438984 |
| DNAJC12 | -0.012938869 | 0.009907354 | 0.017448518 |
| CINP | -0.009416562 | 0.00993051 | 0.017481791 |
| SEC31A | -0.015833317 | 0.009942205 | 0.017494866 |
| SDC1 | -0.016455837 | 0.009963516 | 0.017524846 |
| ARMCX3 | -0.009140865 | 0.010028824 | 0.017632152 |
| SLC34A1 | 0.020533847 | 0.010046374 | 0.017655436 |
| PRKRA | -0.008365119 | 0.01006687 | 0.017683877 |
| TPMT | -0.020682174 | 0.010126615 | 0.017781209 |
| MFF | -0.011575789 | 0.01013968 | 0.017796528 |
| CCT5 | -0.013258149 | 0.010161671 | 0.017827493 |
| POLR2K | -0.018946739 | 0.010179495 | 0.017851124 |
| CHD9 | 0.013640033 | 0.01028221 | 0.018023541 |
| HSD17B4 | -0.011680871 | 0.010314599 | 0.018072587 |
| SRP54 | -0.010621291 | 0.010378962 | 0.018177592 |
| METTL7A | 0.024765528 | 0.010499004 | 0.018379982 |
| ANPEP | -0.027985524 | 0.010516018 | 0.018401909 |
| OLA1 | -0.011461386 | 0.010548263 | 0.01845046 |
| SLC44A1 | -0.00937721 | 0.010709939 | 0.018725266 |
| CZIB | -0.011937708 | 0.01080804 | 0.018888731 |
| ATPAF1 | -0.010253567 | 0.01081637 | 0.018895235 |

| | | | |
|---|---|---|---|
| BAIAP2 | -0.009394136 | 0.010878393 | 0.01899549 |
| TMEM183. | -0.01035392 | 0.010980025 | 0.019164795 |
| PKN2 | -0.011291066 | 0.01114237 | 0.01943988 |
| P4HA2 | -0.010121886 | 0.011157112 | 0.019457321 |
| NDUFS7 | -0.022910567 | 0.011274883 | 0.019651216 |
| MVB12A | -0.009413558 | 0.011277881 | 0.019651216 |
| ZDHHC2 | -0.00859772 | 0.011295993 | 0.019674414 |
| UFD1 | -0.009759175 | 0.011532003 | 0.020076948 |
| ACAD8 | -0.010662726 | 0.011656433 | 0.020284963 |
| BLOC1S6 | -0.010178387 | 0.011813101 | 0.020548159 |
| BCKDHB | 0.010428108 | 0.011817697 | 0.020548159 |
| GABARAPL | -0.014778017 | 0.011858756 | 0.020610809 |
| CERS4 | 0.008738293 | 0.01190653 | 0.020678087 |
| NTN4 | 0.017293568 | 0.011907552 | 0.020678087 |
| AQP1 | -0.038070258 | 0.011926642 | 0.020702469 |
| SNHG6 | -0.016914978 | 0.011946947 | 0.020728938 |
| AKAP9 | -0.014200059 | 0.011965778 | 0.020752829 |
| GIPC1 | -0.008913303 | 0.012032924 | 0.02086046 |
| CERS2 | -0.017150787 | 0.012122898 | 0.021007558 |
| SESN2 | -0.010169855 | 0.012142063 | 0.021031878 |
| TUBG1 | -0.009947586 | 0.012149401 | 0.021035703 |
| MRPS28 | -0.0142897 | 0.012276629 | 0.021247015 |
| ASB9 | -0.009456271 | 0.012292199 | 0.021264986 |
| LIFR | -0.008897929 | 0.012309505 | 0.021285943 |
| EPHA7 | 0.010300735 | 0.012444044 | 0.021509519 |
| TTC19 | -0.008722154 | 0.012470805 | 0.021546692 |
| TLK1 | -0.008637425 | 0.012485717 | 0.02156337 |
| KMT2C | 0.009329799 | 0.012492388 | 0.021565807 |
| PCYOX1 | -0.018520901 | 0.012595643 | 0.021734906 |
| VPS35 | -0.012932945 | 0.012663041 | 0.021842015 |
| NSD1 | -0.009018882 | 0.012686701 | 0.021873622 |
| POLR2I | 0.020334329 | 0.012698956 | 0.021885548 |
| NUB1 | -0.008558479 | 0.012714886 | 0.021903796 |
| CPM | 0.010896593 | 0.012734549 | 0.021920624 |
| TMED7 | -0.010268998 | 0.012735348 | 0.021920624 |
| CHURC1 | -0.016971678 | 0.01275589 | 0.021946768 |
| MSH3 | 0.01365106 | 0.012793076 | 0.022001514 |
| ESF1 | -0.009699326 | 0.012823896 | 0.022045272 |
| ECHS1 | -0.042174631 | 0.013003913 | 0.022345366 |
| GLTPD2 | 0.013158835 | 0.013019551 | 0.022362865 |
| MAPK1IP1 | -0.014045706 | 0.013078763 | 0.022453947 |
| MPG | -0.00932038 | 0.013083531 | 0.022453947 |
| DCAF8 | -0.010214394 | 0.013090239 | 0.022456059 |
| RPS14 | -0.047240306 | 0.013150649 | 0.022550256 |
| EPHX2 | -0.016686378 | 0.013172992 | 0.022579125 |
| WBP11 | -0.009351615 | 0.013219585 | 0.022649519 |
| UBE2W | 0.011049464 | 0.013272663 | 0.02273096 |

| | | | |
|---|---|---|---|
| SERPINB1 | -0.008971205 | 0.013325984 | 0.022812749 |
| HOXB7 | -0.009392148 | 0.013356182 | 0.022854902 |
| MRPL14 | -0.01684844 | 0.013520595 | 0.023124878 |
| QARS | 0.010044064 | 0.013525234 | 0.023124878 |
| ARHGEF12 | 0.014480159 | 0.013554818 | 0.0231658 |
| SPTSSA | -0.008397831 | 0.013599415 | 0.023232334 |
| NDUFS1 | -0.016116925 | 0.013615904 | 0.023250814 |
| NFS1 | -0.008852647 | 0.013733299 | 0.023441518 |
| BCL7A | -0.009764371 | 0.013753711 | 0.02346659 |
| SNX17 | -0.011442154 | 0.013800372 | 0.023536409 |
| RENBP | 0.012761087 | 0.013818529 | 0.023557575 |
| HDGF | -0.014223148 | 0.013859977 | 0.023618415 |
| CA2 | 0.027391636 | 0.013944084 | 0.023751867 |
| RAD50 | -0.009732827 | 0.013976284 | 0.02379683 |
| SUDS3 | -0.008133243 | 0.01399098 | 0.023811962 |
| NHP2 | -0.015115348 | 0.014025665 | 0.023861089 |
| DAB2 | -0.031359285 | 0.014180863 | 0.024115113 |
| LRRFIP1 | -0.009685235 | 0.014256372 | 0.024233468 |
| GPI | -0.014130435 | 0.014497771 | 0.024633594 |
| ITGA1 | -0.008270227 | 0.014517739 | 0.024657304 |
| C19orf25 | -0.008750757 | 0.014530495 | 0.02466875 |
| EPS8L2 | -0.015895052 | 0.014562366 | 0.024711397 |
| FBP1 | -0.037918242 | 0.01456767 | 0.024711397 |
| CYB5R1 | -0.008761543 | 0.01463654 | 0.024817955 |
| CDC42BPB | -0.008078687 | 0.014653453 | 0.024836361 |
| NDUFB1 | 0.033101482 | 0.014682403 | 0.02487336 |
| UBE2D2 | -0.013939851 | 0.014687416 | 0.02487336 |
| AKAP13 | 0.008908369 | 0.014741415 | 0.024954501 |
| AOPEP | 0.013578484 | 0.014890342 | 0.025196204 |
| UBB | -0.037801828 | 0.014908682 | 0.02521683 |
| TECR | -0.01657955 | 0.015017658 | 0.025390679 |
| DCTN6 | -0.010608669 | 0.015097401 | 0.025514981 |
| ARID1B | 0.010717049 | 0.015207881 | 0.025691106 |
| FAM168B | -0.009927946 | 0.015323775 | 0.025866491 |
| MRPS22 | -0.008264203 | 0.015324318 | 0.025866491 |
| PSMD13 | -0.009994671 | 0.015345279 | 0.025891211 |
| ARHGAP29 | -0.015018873 | 0.015419464 | 0.026005678 |
| IL6ST | -0.015700385 | 0.015430265 | 0.026013194 |
| FAM210B | -0.009604343 | 0.015502424 | 0.026124101 |
| ADAR | -0.01123621 | 0.015519783 | 0.026135202 |
| NT5C | -0.013756592 | 0.01552176 | 0.026135202 |
| CCNY | 0.010100083 | 0.015564018 | 0.02619309 |
| CIR1 | -0.010093934 | 0.015568917 | 0.02619309 |
| PSIP1 | -0.008768189 | 0.015665436 | 0.026340646 |
| IP6K2 | -0.008717681 | 0.015669472 | 0.026340646 |
| NNT | -0.009096353 | 0.015754136 | 0.026472114 |
| TRAPPC5 | -0.021206308 | 0.015790793 | 0.026522552 |

| | | | |
|---|---|---|---|
| KMO | 0.011828718 | 0.01579709 | 0.026522552 |
| ROMO1 | -0.024818096 | 0.015878765 | 0.026648767 |
| LAMTOR5 | -0.038199279 | 0.015929623 | 0.026723181 |
| PSMD9 | -0.015467827 | 0.016033089 | 0.026882636 |
| TSFM | -0.011709378 | 0.016037787 | 0.026882636 |
| ATP5F1B | -0.027760652 | 0.016183423 | 0.027115666 |
| LSM14A | -0.011654338 | 0.016219532 | 0.027165066 |
| ACTR3 | -0.011495769 | 0.016473898 | 0.027579821 |
| PPP1R11 | -0.00910951 | 0.01653934 | 0.027678079 |
| YAP1 | 0.008592252 | 0.016614526 | 0.027792557 |
| SORBS2 | 0.012087469 | 0.016712147 | 0.027944455 |
| CAMLG | -0.01102489 | 0.016783161 | 0.028051757 |
| EIF3I | -0.01417509 | 0.016976687 | 0.028363659 |
| LACTB2 | -0.015534272 | 0.01703613 | 0.028451378 |
| PTCD3 | -0.007964889 | 0.017129697 | 0.028595992 |
| HNRNPM | 0.014033273 | 0.017160153 | 0.028635176 |
| GCLM | -0.010114643 | 0.017177325 | 0.028652169 |
| USF2 | -0.009371382 | 0.017261342 | 0.028780603 |
| MRPL42 | -0.011890134 | 0.017296748 | 0.028827914 |
| GALK1 | -0.017511631 | 0.017399962 | 0.028988153 |
| ZNF32 | -0.008386488 | 0.017516705 | 0.029170793 |
| ZBTB44 | 0.013771317 | 0.017556703 | 0.029221365 |
| DHRS4 | -0.014221973 | 0.017561328 | 0.029221365 |
| STX16 | -0.00765824 | 0.017770111 | 0.029556777 |
| KIAA1191 | -0.017983698 | 0.017894717 | 0.029751963 |
| CYFIP1 | -0.007617296 | 0.017915582 | 0.029774579 |
| RO60 | -0.010389337 | 0.017935789 | 0.029796084 |
| CNIH1 | -0.008522919 | 0.018015378 | 0.02991618 |
| NR1H4 | -0.013516315 | 0.018112191 | 0.03006477 |
| ACLY | 0.011731099 | 0.018120018 | 0.030065591 |
| VAMP3 | -0.011045802 | 0.018135867 | 0.030079715 |
| RPP21 | -0.010441419 | 0.018320761 | 0.030365315 |
| ATP5ME | 0.036655474 | 0.018322876 | 0.030365315 |
| MARC2 | -0.015559006 | 0.018474374 | 0.030604013 |
| POR | -0.011096589 | 0.018580371 | 0.030767174 |
| SIK2 | 0.008735784 | 0.018612183 | 0.030807408 |
| SCAF11 | -0.013435308 | 0.018630103 | 0.030824625 |
| PPP2CA | -0.010267113 | 0.01864169 | 0.030831354 |
| GLOD4 | -0.012487709 | 0.018654383 | 0.030839907 |
| TXN | -0.042963523 | 0.018696615 | 0.030897267 |
| DCTN2 | -0.009706387 | 0.018847753 | 0.031134483 |
| ZRANB2 | -0.008979712 | 0.019016354 | 0.031400343 |
| PPP1R16B | 0.009711052 | 0.019058766 | 0.031457705 |
| SERINC1 | -0.014218461 | 0.01907724 | 0.031475526 |
| EIF2S3 | -0.015556195 | 0.01920124 | 0.031667371 |
| NSRP1 | 0.008770638 | 0.019663053 | 0.03241597 |
| EMX2OS | -0.011833196 | 0.019673722 | 0.032420522 |

| | | | |
|---|---|---|---|
| TTC38 | 0.017821352 | 0.019759706 | 0.032549134 |
| RNF14 | -0.008450548 | 0.019799721 | 0.03260195 |
| CCNG1 | -0.01650603 | 0.019860991 | 0.032689709 |
| ZFAND5 | -0.011442458 | 0.020040066 | 0.032971216 |
| NASP | -0.008100663 | 0.020226967 | 0.033259375 |
| PTPRA | -0.009341586 | 0.020231434 | 0.033259375 |
| RHOQ | -0.009154231 | 0.020434955 | 0.033580488 |
| KMT5B | -0.007959272 | 0.020481714 | 0.033643842 |
| SLC25A1 | -0.015523243 | 0.020510637 | 0.033677858 |
| HDLBP | -0.015164582 | 0.020673113 | 0.033931049 |
| ATE1 | 0.00897 | 0.020721858 | 0.033997446 |
| PHYHD1 | -0.010570885 | 0.020746857 | 0.034024845 |
| GSR | -0.011092007 | 0.020936958 | 0.034322881 |
| UTP4 | 0.010299192 | 0.021041654 | 0.034480727 |
| CHMP4A | -0.012010153 | 0.02111841 | 0.034592681 |
| TUBA1C | -0.012623627 | 0.021268747 | 0.034825025 |
| SNRNP35 | -0.007716932 | 0.021322302 | 0.034898778 |
| ACY3 | 0.018423296 | 0.021348674 | 0.034927998 |
| LINC01874 | 0.015949379 | 0.021394234 | 0.034988576 |
| PRPF40A | -0.010117659 | 0.021450382 | 0.035066415 |
| TOP2B | -0.00905299 | 0.021652207 | 0.035382244 |
| NFYB | -0.008097143 | 0.021931359 | 0.035824132 |
| MARCH7 | -0.008392037 | 0.022153983 | 0.036173369 |
| HPF1 | -0.007689313 | 0.022277538 | 0.036360631 |
| UQCC3 | -0.013046514 | 0.022407522 | 0.036558234 |
| NRBP1 | -0.008630896 | 0.022434194 | 0.036587191 |
| FAM92A | -0.008329912 | 0.022473324 | 0.036634299 |
| UQCRC1 | -0.018895288 | 0.022480981 | 0.036634299 |
| LCMT1 | -0.008746947 | 0.022489886 | 0.036634299 |
| COPB2 | -0.009811426 | 0.022532315 | 0.036688837 |
| URI1 | -0.010359807 | 0.022585297 | 0.036760508 |
| UPF3A | -0.008583524 | 0.022648419 | 0.036848618 |
| BRD7 | -0.008924207 | 0.022830946 | 0.037130852 |
| UFSP2 | -0.010001625 | 0.022903101 | 0.037233431 |
| JUNB | -0.018220212 | 0.023051052 | 0.037459101 |
| NDUFS8 | -0.022585262 | 0.023093995 | 0.037514017 |
| AKR1C1 | 0.01353425 | 0.023284115 | 0.03780787 |
| MKLN1 | 0.010653743 | 0.02330819 | 0.037831979 |
| PFDN6 | 0.010190093 | 0.023356766 | 0.037895821 |
| CDC123 | -0.007776259 | 0.02343567 | 0.0380088 |
| CDC42BPA | 0.009504917 | 0.023573454 | 0.038217146 |
| C8orf82 | -0.012693408 | 0.023676365 | 0.038368813 |
| SKIL | -0.00914959 | 0.023721451 | 0.038426689 |
| PPP2R1A | -0.014329144 | 0.023754788 | 0.038465493 |
| WBP2 | -0.012571611 | 0.024074307 | 0.038967493 |
| EIF4A1 | -0.019406209 | 0.02415484 | 0.039082417 |
| NAA38 | -0.01972772 | 0.024211103 | 0.039157996 |

| | | | |
|---|---|---|---|
| RARRES2 | -0.013411399 | 0.024244751 | 0.039196956 |
| SPG21 | -0.012706864 | 0.024275758 | 0.039231615 |
| MRLN | 0.014089614 | 0.024491875 | 0.039565282 |
| IVNS1ABP | 0.01132513 | 0.025438766 | 0.041078747 |
| HRAS | -0.008322126 | 0.02561822 | 0.041352244 |
| ABCE1 | -0.007599036 | 0.025742984 | 0.041537282 |
| TOB2 | -0.00929509 | 0.025872648 | 0.041730077 |
| KIAA2013 | -0.008684024 | 0.025929948 | 0.04180605 |
| G6PC | 0.012458969 | 0.025950661 | 0.041823 |
| ZNF24 | -0.008701708 | 0.026128039 | 0.042092321 |
| MAPK1 | 0.010025528 | 0.026377943 | 0.042478228 |
| MED31 | 0.009232177 | 0.026557732 | 0.042750963 |
| MAP3K11 | -0.007363401 | 0.026750356 | 0.043044136 |
| ZFAND6 | 0.012306758 | 0.026852094 | 0.043190893 |
| PRKAG1 | -0.007777018 | 0.026960109 | 0.043347626 |
| THRAP3 | -0.010387325 | 0.027070109 | 0.043507427 |
| SPPL3 | 0.007480487 | 0.027093991 | 0.043528747 |
| AP1G1 | -0.00963448 | 0.027140211 | 0.043585925 |
| ALDH9A1 | -0.015870597 | 0.027192993 | 0.043653592 |
| PSMD8 | -0.0166325 | 0.02756521 | 0.044233801 |
| AC137056. | 0.009049584 | 0.027724342 | 0.044471753 |
| STX8 | -0.009348695 | 0.027858635 | 0.044652782 |
| ABI2 | -0.008126029 | 0.02785898 | 0.044652782 |
| OGA | -0.011253936 | 0.027891294 | 0.044687106 |
| REPIN1 | -0.007612326 | 0.028070105 | 0.044956028 |
| MRPL52 | -0.018571491 | 0.028292714 | 0.045294857 |
| ARL2 | -0.019499154 | 0.028353318 | 0.045374163 |
| PTEN | -0.011672212 | 0.028533269 | 0.045644324 |
| SNX29 | 0.012320651 | 0.028575914 | 0.045694714 |
| RNF114 | -0.010214423 | 0.028753402 | 0.045933092 |
| ATG101 | -0.007388481 | 0.028756499 | 0.045933092 |
| TWF1 | -0.00859778 | 0.028764632 | 0.045933092 |
| RPS20 | -0.019374485 | 0.0287698 | 0.045933092 |
| TIMM17B | -0.011309684 | 0.029016259 | 0.046308548 |
| DRAP1 | -0.012981594 | 0.029776427 | 0.047503249 |
| RXRA | -0.008479747 | 0.029901645 | 0.047684459 |
| WIPI1 | -0.00752188 | 0.029996142 | 0.047816556 |
| GPANK1 | -0.007733339 | 0.030537998 | 0.048654433 |
| TK2 | -0.008575509 | 0.030545491 | 0.048654433 |
| TATDN1 | -0.010136151 | 0.030608902 | 0.048736504 |
| SPNS2 | 0.010182969 | 0.030636743 | 0.048761898 |
| SESN3 | -0.009986001 | 0.030684259 | 0.048818572 |
| SPRYD7 | -0.008150411 | 0.030814975 | 0.049007524 |
| TEAD1 | 0.01099685 | 0.030951299 | 0.049205244 |
| MCRIP1 | -0.012122289 | 0.030979621 | 0.049231181 |
| HINT2 | -0.019883972 | 0.031173128 | 0.049519498 |
| RILPL2 | -0.007649287 | 0.031430442 | 0.049908913 |

| | | | |
|---|---|---|---|
| SF3B1 | -0.013485748 | 0.031857992 | 0.050568241 |
| FGFR4 | -0.007769762 | 0.031996518 | 0.050768468 |
| TIAL1 | -0.008867752 | 0.032066958 | 0.050860552 |
| ACTR10 | -0.01002124 | 0.032080353 | 0.050862122 |
| FNIP1 | 0.00721445 | 0.032447187 | 0.051423837 |
| PACSIN2 | -0.008707556 | 0.032567598 | 0.051594727 |
| DPM1 | -0.007695101 | 0.032894039 | 0.052091757 |
| SMIM26 | -0.01692962 | 0.032988925 | 0.05222185 |
| SLC13A3 | -0.021879862 | 0.033080378 | 0.05234641 |
| ATP5F1E | -0.04118861 | 0.033222132 | 0.05255044 |
| IGFBP4 | -0.020419531 | 0.033491167 | 0.052955566 |
| MRPL58 | -0.009475691 | 0.033554197 | 0.053034775 |
| RNPS1 | -0.008644598 | 0.033610463 | 0.053103236 |
| IYD | -0.009083469 | 0.033862735 | 0.053481207 |
| DNAJB6 | -0.012578505 | 0.03391695 | 0.053546205 |
| CTBP2 | -0.006858933 | 0.034056295 | 0.0537455 |
| ETFA | -0.016806392 | 0.034254069 | 0.054036815 |
| HELZ | 0.007695356 | 0.034419555 | 0.054276991 |
| IQGAP2 | -0.010754106 | 0.034496526 | 0.054377453 |
| BAZ2B | 0.007619597 | 0.034527918 | 0.054406021 |
| NSD3 | -0.008046986 | 0.03481723 | 0.054840816 |
| BPTF | -0.009412407 | 0.034866318 | 0.054897044 |
| TRA2A | -0.008002221 | 0.034910706 | 0.054945833 |
| RALB | -0.008466785 | 0.035103865 | 0.055228644 |
| CPQ | -0.00989647 | 0.035165029 | 0.055303652 |
| ITGA6 | -0.009436357 | 0.035180156 | 0.055306228 |
| RAB32 | -0.007903207 | 0.035203772 | 0.05532138 |
| ATP5F1C | -0.022984021 | 0.035216781 | 0.05532138 |
| SEPTIN2 | -0.015218411 | 0.035455212 | 0.055674595 |
| DIO1 | -0.011922232 | 0.035679903 | 0.056005973 |
| SMTNL2 | -0.007525133 | 0.035822552 | 0.056208367 |
| WDR6 | -0.008840346 | 0.035887225 | 0.056288302 |
| COMMD1( | -0.009036335 | 0.036015919 | 0.056468554 |
| PCGF2 | -0.009279595 | 0.036162674 | 0.056676974 |
| NFE2L2 | -0.01241501 | 0.036598819 | 0.057326026 |
| DYNC1LI2 | -0.008760831 | 0.036605765 | 0.057326026 |
| KPNB1 | -0.009119217 | 0.036618747 | 0.057326026 |
| DDX1 | -0.01117989 | 0.036651637 | 0.057338031 |
| MXI1 | 0.008793296 | 0.036654385 | 0.057338031 |
| FXR1 | -0.012136333 | 0.036732321 | 0.05743803 |
| ACIN1 | -0.007184069 | 0.036831179 | 0.057570658 |
| ORC4 | 0.008726387 | 0.037015512 | 0.057836738 |
| ATG3 | -0.007523252 | 0.037239204 | 0.05816409 |
| NUDT8 | 0.012230733 | 0.037396135 | 0.058386959 |
| AKAP8L | -0.006692547 | 0.037481613 | 0.058498141 |
| ZNF503 | -0.010148817 | 0.037929611 | 0.059148226 |
| PMPCA | -0.007670054 | 0.037940194 | 0.059148226 |

| | | | |
|---|---|---|---|
| TRIM47 | -0.009629372 | 0.037941423 | 0.059148226 |
| FXYD2 | -0.059066017 | 0.038186945 | 0.059508352 |
| AP3D1 | -0.008709208 | 0.038204246 | 0.059512693 |
| DENND10 | -0.009505024 | 0.038237826 | 0.05954238 |
| SZRD1 | -0.007848872 | 0.038333251 | 0.05966831 |
| MGST2 | -0.013159264 | 0.038624544 | 0.060098911 |
| LRRC41 | -0.006898331 | 0.038739531 | 0.060254961 |
| RPL13 | -0.045879825 | 0.038828048 | 0.060369738 |
| SNHG18 | -0.010802321 | 0.038993854 | 0.060600024 |
| AKR1A1 | -0.028569874 | 0.039005723 | 0.060600024 |
| NELFCD | -0.00775687 | 0.039220395 | 0.060900275 |
| RTL8C | -0.00722406 | 0.039228689 | 0.060900275 |
| SNX5 | -0.014049195 | 0.039422591 | 0.061178132 |
| PSMC5 | -0.015725097 | 0.039524118 | 0.061296613 |
| PHYH | -0.017705781 | 0.03952884 | 0.061296613 |
| REST | -0.007226403 | 0.039545853 | 0.06129981 |
| GON7 | -0.007685018 | 0.040033528 | 0.0620323 |
| GPAT3 | 0.013227898 | 0.040438899 | 0.062636753 |
| APOC3 | -0.012270666 | 0.040504395 | 0.062714509 |
| RPS11 | 0.027066749 | 0.040655334 | 0.062924451 |
| FBXO9 | -0.009551749 | 0.04079003 | 0.063109104 |
| LINC02532 | 0.013823316 | 0.040844126 | 0.063168961 |
| LMBRD1 | -0.011027687 | 0.04098542 | 0.063363582 |
| ANXA4 | -0.015851928 | 0.041023309 | 0.063398254 |
| TSG101 | -0.009899689 | 0.041103856 | 0.063498797 |
| SHMT2 | -0.007826786 | 0.041119415 | 0.063498909 |
| NCEH1 | 0.007193002 | 0.04120878 | 0.063612951 |
| MYH9 | -0.008412705 | 0.04122667 | 0.063616616 |
| DHTKD1 | -0.010987812 | 0.041327129 | 0.063729246 |
| DCAF7 | -0.009254703 | 0.041330748 | 0.063729246 |
| GABARAP | -0.025760399 | 0.041347187 | 0.063730626 |
| MED28 | -0.006785425 | 0.041372389 | 0.063745507 |
| COPS8 | -0.007272347 | 0.041433569 | 0.06381579 |
| KIDINS220 | -0.006789177 | 0.041462105 | 0.06383576 |
| TUSC2 | -0.011841739 | 0.041516239 | 0.063895112 |
| TBC1D15 | 0.007001084 | 0.041735718 | 0.064208797 |
| EIF2A | -0.010133568 | 0.04208275 | 0.064718407 |
| LINC01139 | -0.007546312 | 0.042817709 | 0.065824 |
| PLD1 | 0.007314708 | 0.043020894 | 0.066111569 |
| RNLS | 0.007557616 | 0.043135448 | 0.066262772 |
| RPL38 | 0.031153472 | 0.043416811 | 0.066670009 |
| PAM | 0.008784898 | 0.043695485 | 0.067072815 |
| MED11 | -0.008834817 | 0.043839528 | 0.067268736 |
| CCDC59 | -0.00713471 | 0.043930946 | 0.067383794 |
| JKAMP | -0.006825341 | 0.044283393 | 0.067898995 |
| MAP3K12 | -0.009158377 | 0.044385741 | 0.068030481 |
| PPP4R3A | 0.007274262 | 0.044402655 | 0.068030973 |

| | | | |
|---|---|---|---|
| GPAT4 | 0.006721471 | 0.044526118 | 0.068194651 |
| SDHAF2 | -0.00741644 | 0.044582587 | 0.068255641 |
| NPTN | -0.009000178 | 0.044986814 | 0.068848801 |
| ZNF770 | -0.009567317 | 0.045340915 | 0.069364833 |
| SELENOP | 0.029258484 | 0.045658153 | 0.06981107 |
| AFF4 | 0.009318064 | 0.045666656 | 0.06981107 |
| MGAT4A | -0.008763504 | 0.045812432 | 0.070007817 |
| FNDC3A | 0.009247878 | 0.045931162 | 0.070163102 |
| XPNPEP2 | -0.013526974 | 0.045966429 | 0.070190823 |
| EIF3E | 0.018361869 | 0.046062786 | 0.070311774 |
| IDH1 | -0.016670289 | 0.046199325 | 0.070493945 |
| FAM120A | -0.012079361 | 0.04631043 | 0.070637189 |
| PGPEP1 | 0.012560751 | 0.046636323 | 0.071107818 |
| WAC | 0.010129114 | 0.046861597 | 0.071424739 |
| SRSF11 | -0.0101091 | 0.047109673 | 0.071776164 |
| TEX30 | -0.008237671 | 0.047513202 | 0.072364089 |
| EHD1 | 0.009206422 | 0.047652221 | 0.072548869 |
| TTC39B | 0.00780251 | 0.047671433 | 0.072551178 |
| RHOB | -0.013515859 | 0.047767351 | 0.072670181 |
| SVIP | -0.009197167 | 0.047857561 | 0.072780416 |
| IMPAD1 | -0.00650289 | 0.047914591 | 0.072840127 |
| COBL | 0.00642593 | 0.048016283 | 0.072967665 |
| GRB10 | 0.007730353 | 0.048615592 | 0.073851029 |
| HGD | 0.014460762 | 0.04880883 | 0.074117112 |
| FAM199X | -0.006896989 | 0.048832731 | 0.074125953 |
| TUT4 | 0.008061236 | 0.049203928 | 0.074661771 |
| ADAM10 | 0.007720184 | 0.049233722 | 0.074679342 |
| SORD | -0.016238395 | 0.049368085 | 0.074855454 |
| APEH | 0.010265455 | 0.049486695 | 0.07500756 |
| VAPA | -0.013962783 | 0.049608924 | 0.075165036 |
| SGK3 | 0.008285678 | 0.049702593 | 0.07527914 |
| BUB3 | -0.006804723 | 0.049875486 | 0.075513107 |
| OSBPL10 | 0.00747737 | 0.050157663 | 0.075912299 |
| APPL1 | -0.008105313 | 0.050274606 | 0.076061212 |
| SHMT1 | -0.021285262 | 0.050442912 | 0.076287694 |
| HSPA1B | 0.012451043 | 0.050645441 | 0.076565748 |
| PSMA3 | -0.012441541 | 0.050684777 | 0.076596972 |
| LLGL2 | -0.009334573 | 0.050733762 | 0.07664275 |
| LACTB | -0.006809346 | 0.050951791 | 0.076943773 |
| CPPED1 | -0.008623125 | 0.051219611 | 0.077319737 |
| MOB1A | -0.013330036 | 0.052090732 | 0.078605815 |
| ACADS | -0.009350193 | 0.052115086 | 0.078613633 |
| AFTPH | 0.011016908 | 0.052324395 | 0.078900338 |
| POLR2J3 | -0.007038103 | 0.05236274 | 0.07892913 |
| CDK16 | -0.006467554 | 0.05252299 | 0.079136152 |
| UBC | -0.021508521 | 0.052538684 | 0.079136152 |
| TCEAL9 | -0.010102372 | 0.053082971 | 0.079926618 |

| | | | |
|---|---|---|---|
| NAA50 | 0.008901715 | 0.053283856 | 0.080199637 |
| UBE2J2 | -0.007273754 | 0.053384033 | 0.080320931 |
| CFAP97 | -0.009547704 | 0.053742871 | 0.08083117 |
| PDXDC1 | 0.010822714 | 0.053818849 | 0.080915761 |
| CHMP1A | -0.007156396 | 0.054161139 | 0.081400539 |
| DNAJC13 | 0.006973043 | 0.05493014 | 0.082526044 |
| PIPOX | -0.013678324 | 0.055012326 | 0.082619245 |
| GSK3B | 0.006318524 | 0.055704572 | 0.083604542 |
| SLC17A5 | -0.007736719 | 0.05572234 | 0.083604542 |
| PQBP1 | -0.007675744 | 0.055729564 | 0.083604542 |
| ALDH7A1 | -0.014639663 | 0.055785279 | 0.083657514 |
| LENG8 | -0.006069949 | 0.056093241 | 0.084088587 |
| SLC7A7 | -0.021222199 | 0.05624756 | 0.084289107 |
| SMPDL3A | 0.009430554 | 0.056302143 | 0.084340076 |
| CSNK1G2 | -0.006396452 | 0.056489685 | 0.084590105 |
| CORO1B | -0.008244747 | 0.056516756 | 0.084599744 |
| GALK2 | 0.007185736 | 0.056930613 | 0.085188143 |
| PAX8 | -0.013646441 | 0.057146012 | 0.085479259 |
| LAMP2 | -0.01283081 | 0.057365499 | 0.085776275 |
| RRAGC | -0.006533363 | 0.057683508 | 0.086211308 |
| G3BP1 | 0.009671022 | 0.057698495 | 0.086211308 |
| NCOA1 | 0.006481633 | 0.057723857 | 0.086217783 |
| TMOD3 | -0.007745159 | 0.058357231 | 0.087132063 |
| CDC40 | -0.00765404 | 0.058435792 | 0.087217599 |
| KIF21A | -0.008965093 | 0.058915342 | 0.087901347 |
| UBXN6 | -0.012034273 | 0.058962573 | 0.087939814 |
| MPLKIP | -0.012542164 | 0.05900763 | 0.087960049 |
| CLPP | -0.010396908 | 0.059019047 | 0.087960049 |
| ASPSCR1 | -0.008341061 | 0.05911288 | 0.08806788 |
| MRPS9 | -0.006430053 | 0.059554245 | 0.088693209 |
| ZNF148 | 0.009082439 | 0.059724135 | 0.088894518 |
| CPNE3 | -0.010404639 | 0.05973278 | 0.088894518 |
| BDH2 | -0.023092125 | 0.059874561 | 0.089073186 |
| PRMT2 | 0.007957612 | 0.060064813 | 0.089323806 |
| PRNP | -0.010601809 | 0.06032029 | 0.089671207 |
| RB1CC1 | 0.007369751 | 0.060630181 | 0.090099218 |
| LASP1 | -0.0092416 | 0.060666395 | 0.090120369 |
| PDLIM2 | -0.010269683 | 0.060902058 | 0.090410325 |
| ATP5MPL | -0.024539868 | 0.060905687 | 0.090410325 |
| MRPL35 | -0.007666533 | 0.061288374 | 0.09094547 |
| LINC02802 | -0.009124489 | 0.061353893 | 0.091009755 |
| PAFAH1B2 | -0.006759388 | 0.061514058 | 0.091214335 |
| TERF1 | -0.006589441 | 0.061781126 | 0.091577229 |
| FARP1 | -0.015526253 | 0.061873083 | 0.091680391 |
| CIB1 | -0.017822468 | 0.062085626 | 0.09196209 |
| ANKRD11 | -0.00736186 | 0.062455378 | 0.092476363 |
| NUDT14 | -0.009254622 | 0.062525887 | 0.092529856 |

| | | | |
|---|---|---|---|
| TUBGCP2 | -0.009340621 | 0.062536642 | 0.092529856 |
| TINAG | 0.013222689 | 0.06256943 | 0.092544972 |
| EIF4E2 | -0.009809376 | 0.063170821 | 0.093400781 |
| ARSB | -0.006472057 | 0.063243286 | 0.093474216 |
| SEPTIN7 | -0.009787336 | 0.063321447 | 0.093556011 |
| ALAD | -0.008867133 | 0.063372518 | 0.093597739 |
| ANXA11 | -0.010328766 | 0.063745857 | 0.094115238 |
| NAPSA | -0.011988789 | 0.063851732 | 0.094237617 |
| ZNF704 | 0.006900644 | 0.06402578 | 0.094460489 |
| UXT | -0.014219067 | 0.064506442 | 0.0951354 |
| ENSA | -0.010717234 | 0.064996897 | 0.095824264 |
| GNS | -0.011071482 | 0.065402414 | 0.096387454 |
| PLS3 | -0.006841624 | 0.065707628 | 0.09680247 |
| RNF135 | -0.006596432 | 0.066671066 | 0.098172716 |
| FRG1 | -0.006854426 | 0.066685613 | 0.098172716 |
| RAB29 | -0.012736323 | 0.066748927 | 0.098230654 |
| CTDSP1 | -0.008720216 | 0.066897246 | 0.098413602 |
| UBE2G2 | -0.006638464 | 0.067383232 | 0.099091511 |
| PPP2CB | -0.011119537 | 0.067406396 | 0.099091511 |
| SECISBP2 | -0.0074647 | 0.067652862 | 0.099418185 |
| FBXW11 | 0.00785707 | 0.067781645 | 0.099571746 |
| SH3RF1 | 0.006792543 | 0.067941224 | 0.099770422 |
| YME1L1 | -0.008411587 | 0.068530379 | 0.100599554 |
| ZNF644 | 0.007340999 | 0.068607209 | 0.100676291 |
| NDRG1 | -0.015793406 | 0.068692562 | 0.100765475 |
| VRK3 | -0.006567529 | 0.068922333 | 0.101066368 |
| NBR1 | 0.009800796 | 0.069138969 | 0.101347791 |
| ATP5PB | -0.013701547 | 0.069295629 | 0.101534969 |
| OXLD1 | -0.007268323 | 0.06931619 | 0.101534969 |
| XRN1 | 0.005886704 | 0.069452075 | 0.101697681 |
| RALY | -0.007578846 | 0.069675001 | 0.101987685 |
| HIBCH | 0.016864666 | 0.069879999 | 0.102216368 |
| STON2 | 0.00643989 | 0.069881093 | 0.102216368 |
| RAD21 | 0.008248494 | 0.070135869 | 0.102552448 |
| SNAPIN | -0.006134745 | 0.070207688 | 0.102620863 |
| FN3K | -0.007446366 | 0.070352364 | 0.102766126 |
| CTNNA1 | 0.013113614 | 0.070357199 | 0.102766126 |
| GSPT1 | -0.008379869 | 0.070564183 | 0.103031748 |
| RAB30-DT | -0.00683021 | 0.070651158 | 0.103122018 |
| LSM8 | -0.008697675 | 0.070716314 | 0.103180387 |
| RAB3IP | 0.009732781 | 0.071305025 | 0.104002348 |
| LYPLA1 | -0.009594916 | 0.071582561 | 0.104370022 |
| RABGGTB | -0.010644806 | 0.071809711 | 0.104663994 |
| MRPS18A | -0.007019013 | 0.071866786 | 0.104709959 |
| PSMC6 | -0.009112346 | 0.071964199 | 0.104814642 |
| CRAT | -0.008999535 | 0.072051848 | 0.104905035 |
| VEZF1 | -0.005933442 | 0.072193674 | 0.105074214 |

| | | | |
|---|---|---|---|
| TXNRD1 | 0.006486621 | 0.072603681 | 0.105633461 |
| TOP1 | -0.010719296 | 0.072779305 | 0.10585142 |
| SMAD2 | 0.006483823 | 0.072857352 | 0.105927356 |
| AMOTL2 | -0.006204947 | 0.073700739 | 0.107115573 |
| DNAJA1 | -0.012394932 | 0.073995841 | 0.10750636 |
| CLCN7 | -0.006307736 | 0.074161961 | 0.107709543 |
| STXBP3 | -0.007314797 | 0.074200734 | 0.107727695 |
| XAB2 | -0.009648129 | 0.074319613 | 0.107862094 |
| HMOX2 | -0.008072615 | 0.07453552 | 0.108137167 |
| FBXO3 | -0.007203065 | 0.074729384 | 0.10833738 |
| DNPEP | -0.006655655 | 0.07474776 | 0.10833738 |
| SCYL1 | 0.006660616 | 0.074752792 | 0.10833738 |
| PAK2 | -0.008227096 | 0.07478538 | 0.10834631 |
| RSL24D1 | 0.013042729 | 0.074864629 | 0.108422812 |
| EIF2S2 | -0.010166426 | 0.074953233 | 0.108512802 |
| RAD23A | -0.010256426 | 0.075015887 | 0.108565174 |
| CD99 | -0.006967297 | 0.075358076 | 0.109021917 |
| FGFR1OP2 | -0.007767194 | 0.075625407 | 0.109370078 |
| EIF3J | 0.009703219 | 0.075866973 | 0.109659978 |
| PANK1 | 0.007992771 | 0.07588225 | 0.109659978 |
| LARP4B | 0.006416782 | 0.075906102 | 0.109659978 |
| ASPDH | 0.015313813 | 0.075977765 | 0.109709453 |
| PSMB6 | -0.015573045 | 0.076021252 | 0.109709453 |
| ITCH | 0.007236604 | 0.076045194 | 0.109709453 |
| SMAD4 | -0.006728282 | 0.076047382 | 0.109709453 |
| NAGK | -0.006120015 | 0.076132437 | 0.109793525 |
| MRPS33 | -0.011732757 | 0.076208725 | 0.1098649 |
| THAP7 | -0.006455919 | 0.076259688 | 0.109899726 |
| GPR155 | -0.008965811 | 0.076914998 | 0.110805162 |
| VPS13C | -0.007601964 | 0.077362203 | 0.111390944 |
| FAM204A | -0.007527748 | 0.077389861 | 0.111390944 |
| EXOC3 | -0.005874162 | 0.077403122 | 0.111390944 |
| KDM5A | -0.005859434 | 0.077460371 | 0.111434218 |
| PRELID1 | -0.019418421 | 0.077822915 | 0.111916503 |
| QPRT | -0.018244471 | 0.078328272 | 0.112603758 |
| PABPC4 | 0.007091403 | 0.07851805 | 0.112837015 |
| CDKN1C | -0.009945791 | 0.078564154 | 0.112863712 |
| SLC13A1 | -0.01688453 | 0.079046529 | 0.113516907 |
| SUCLA2 | -0.009428886 | 0.079224594 | 0.113732786 |
| SERINC3 | -0.00572491 | 0.079433755 | 0.113993138 |
| ROGDI | 0.006662903 | 0.079630113 | 0.114234942 |
| GPS2 | -0.009669892 | 0.079767732 | 0.11439234 |
| MRPS2 | -0.007580491 | 0.079822669 | 0.1144311 |
| ZHX3 | 0.007873967 | 0.080108529 | 0.114799487 |
| ACBD5 | -0.00777423 | 0.080135642 | 0.114799487 |
| HADH | -0.015082174 | 0.080363725 | 0.115086019 |
| TNPO1 | 0.007230028 | 0.080553728 | 0.115317837 |

| | | | |
|---|---|---|---|
| PCGF5 | 0.007447658 | 0.080656765 | 0.115425039 |
| LPIN2 | 0.008576635 | 0.080830553 | 0.11563338 |
| GTF2F1 | -0.006083437 | 0.081097122 | 0.115974259 |
| PCMTD1 | -0.010398567 | 0.081130529 | 0.115981579 |
| BCL7B | -0.006187841 | 0.081249839 | 0.116111656 |
| RTN3 | -0.007774604 | 0.081355629 | 0.116174232 |
| CAST | -0.010907832 | 0.081359287 | 0.116174232 |
| NECAB2 | -0.007136135 | 0.081378633 | 0.116174232 |
| HSD17B8 | -0.00803542 | 0.081695307 | 0.116585715 |
| CMTM4 | -0.006649649 | 0.082294476 | 0.117399914 |
| DHRS3 | 0.007963107 | 0.082539559 | 0.117671168 |
| RAB11A | -0.009474613 | 0.082542019 | 0.117671168 |
| PLSCR4 | 0.006402766 | 0.082639486 | 0.117769167 |
| YPEL5 | -0.008404223 | 0.082823341 | 0.117990165 |
| FAM20C | -0.010772986 | 0.083143056 | 0.11840449 |
| ADSS | -0.006753673 | 0.083558033 | 0.118954144 |
| NAA10 | -0.008274403 | 0.083606683 | 0.11898209 |
| PAQR5 | -0.007629315 | 0.08366966 | 0.119030398 |
| PUM3 | 0.006348128 | 0.083699715 | 0.119031852 |
| CCNH | 0.005793015 | 0.084092164 | 0.119548499 |
| PALM | 0.00896972 | 0.084387127 | 0.119923507 |
| SLC47A1 | 0.010720041 | 0.084414449 | 0.119923507 |
| YTHDF2 | -0.007157232 | 0.08470734 | 0.12029792 |
| EIF3F | -0.01280785 | 0.084771729 | 0.120347676 |
| RRP7A | -0.005916809 | 0.084965593 | 0.120581146 |
| NFIC | -0.009958502 | 0.085117401 | 0.12075479 |
| SMIM4 | -0.011156974 | 0.085576856 | 0.1213604 |
| RPS15 | -0.034647103 | 0.085603482 | 0.1213604 |
| KIAA0100 | -0.00657279 | 0.085741719 | 0.121503732 |
| RPS27 | -0.041180225 | 0.085763854 | 0.121503732 |
| GPX3 | -0.062051546 | 0.086210804 | 0.122094749 |
| TMF1 | -0.006202875 | 0.086449433 | 0.122390427 |
| MAX | -0.006604185 | 0.086964324 | 0.123076882 |
| HSPA1A | 0.014291775 | 0.087198217 | 0.123365317 |
| GATM | 0.041080368 | 0.087296635 | 0.123461954 |
| PFDN4 | -0.006518508 | 0.087334643 | 0.123473116 |
| GCHFR | -0.015905795 | 0.087669769 | 0.12390419 |
| ETFB | -0.021205473 | 0.087789171 | 0.124030186 |
| CCNL1 | -0.005857337 | 0.08790921 | 0.124156997 |
| TRUB2 | -0.006076771 | 0.088169901 | 0.124482298 |
| PIN4 | -0.010620356 | 0.088402495 | 0.124730704 |
| PREPL | -0.006999899 | 0.088406689 | 0.124730704 |
| CFL2 | -0.008094494 | 0.088593112 | 0.124950725 |
| RFNG | -0.006243846 | 0.089089434 | 0.125607524 |
| SLC22A18 | -0.007971866 | 0.089329771 | 0.125903081 |
| LINC02754 | -0.011324838 | 0.089795999 | 0.126516699 |
| FAM133B | -0.005981294 | 0.089893757 | 0.126610926 |

| | | | |
|---|---|---|---|
| FDFT1 | -0.006472698 | 0.089954467 | 0.126611288 |
| GLDC | -0.006663806 | 0.08997139 | 0.126611288 |
| ZSCAN16-A | -0.012065483 | 0.089986657 | 0.126611288 |
| MYCBP2 | 0.005751697 | 0.090632607 | 0.127476394 |
| ZNF593 | -0.009454722 | 0.090683328 | 0.127503993 |
| PCM1 | -0.007413473 | 0.090737363 | 0.127536232 |
| DICER1 | -0.006698196 | 0.090933898 | 0.127768671 |
| HAAO | 0.009206948 | 0.091088358 | 0.127941852 |
| NIPBL | 0.006336812 | 0.091326952 | 0.12823305 |
| SNX14 | 0.006884251 | 0.09144941 | 0.128361034 |
| ATOX1 | -0.025339891 | 0.091621382 | 0.128558407 |
| ATP6V1A | -0.010378274 | 0.091710859 | 0.128639933 |
| HMGCL | -0.012181375 | 0.092255773 | 0.129360009 |
| GHITM | -0.016911465 | 0.092704007 | 0.129944078 |
| RIT1 | -0.007493773 | 0.092849204 | 0.130103123 |
| ZMYM4 | 0.005620868 | 0.093539612 | 0.131025763 |
| SHTN1 | -0.006083151 | 0.093688779 | 0.131189888 |
| GDA | -0.009543704 | 0.0939365 | 0.131491858 |
| AFMID | -0.010550965 | 0.094028093 | 0.131535788 |
| ATP6V1B2 | -0.013343058 | 0.094032047 | 0.131535788 |
| PUF60 | -0.007674207 | 0.094145179 | 0.13164009 |
| BCKDHA | -0.007857963 | 0.094170825 | 0.13164009 |
| NPLOC4 | -0.006985343 | 0.094254551 | 0.131712222 |
| KDM2A | 0.005459258 | 0.095219819 | 0.13301576 |
| SELENBP1 | -0.008904438 | 0.095317831 | 0.133107325 |
| ANKRD33B | -0.006191912 | 0.095388921 | 0.133125445 |
| RBM17 | -0.006785558 | 0.095395746 | 0.133125445 |
| NEAT1 | -0.02525592 | 0.095470751 | 0.133184784 |
| SLC22A11 | -0.007945573 | 0.095733812 | 0.133506337 |
| BABAM2 | 0.006114834 | 0.096171286 | 0.134070817 |
| STK16 | -0.006599031 | 0.097268411 | 0.13555421 |
| DCTPP1 | -0.005502317 | 0.097339568 | 0.135607281 |
| XIAP | -0.008388538 | 0.097816738 | 0.136225756 |
| FBXL5 | 0.014151459 | 0.098145154 | 0.136636718 |
| GPBP1L1 | 0.006674835 | 0.098212494 | 0.136684055 |
| COPS4 | -0.00596659 | 0.098333629 | 0.136806203 |
| ESRRA | -0.009868234 | 0.098404477 | 0.13685833 |
| TCEA3 | 0.011062921 | 0.098619342 | 0.137110648 |
| CUBN | 0.029314532 | 0.09936321 | 0.13809802 |
| KLHL24 | 0.006695405 | 0.099711853 | 0.138525796 |
| PTPA | -0.007786058 | 0.099738573 | 0.138525796 |
| LARP1 | -0.009825105 | 0.099858982 | 0.138646064 |
| DUSP23 | -0.012314722 | 0.099994253 | 0.138786878 |
| WDR34 | -0.007042925 | 0.100218352 | 0.139010777 |
| SMG1 | 0.005889934 | 0.100253527 | 0.139010777 |
| SORT1 | -0.005472858 | 0.100257285 | 0.139010777 |
| C1orf115 | 0.008199981 | 0.100604983 | 0.139445716 |

| | | | |
|---|---|---|---|
| PLEKHA1 | -0.008012487 | 0.100836949 | 0.139720004 |
| TJP2 | 0.006569881 | 0.101073619 | 0.14000062 |
| UBE2L3 | -0.009843601 | 0.101495471 | 0.140537464 |
| VPS13A | 0.008435473 | 0.101873697 | 0.141013557 |
| SYF2 | 0.011525545 | 0.102198608 | 0.141415557 |
| RGS14 | -0.006084343 | 0.102251488 | 0.141440993 |
| TAF15 | 0.005715716 | 0.102886325 | 0.142271141 |
| KDM3B | 0.005385015 | 0.103105478 | 0.142526116 |
| DCAF6 | 0.005799802 | 0.103314384 | 0.142766759 |
| GSDMD | -0.007562018 | 0.1038242 | 0.143422918 |
| EIF4E | -0.00651728 | 0.104092318 | 0.143744865 |
| VEZT | -0.005124621 | 0.104517686 | 0.144256653 |
| C12orf10 | -0.007786458 | 0.104533297 | 0.144256653 |
| PGM1 | -0.006580166 | 0.104686637 | 0.144419653 |
| BABAM1 | -0.006970531 | 0.105024177 | 0.144831939 |
| HDAC2 | 0.006381108 | 0.105056143 | 0.144831939 |
| REPS2 | 0.006557251 | 0.10567226 | 0.145632358 |
| PSMB2 | -0.009608022 | 0.10712252 | 0.147581429 |
| SLC5A12 | 0.017731886 | 0.107264133 | 0.147726888 |
| JUN | -0.012992448 | 0.107529483 | 0.148033497 |
| WDFY3-AS | -0.006530276 | 0.107558972 | 0.148033497 |
| PAIP2B | 0.005957518 | 0.10802727 | 0.148628123 |
| C5orf24 | -0.00998005 | 0.108268086 | 0.148909477 |
| ZNF33B | 0.006079796 | 0.10892189 | 0.149758468 |
| VAV3 | -0.008326271 | 0.109385222 | 0.150345092 |
| RPS16 | 0.022705672 | 0.109435611 | 0.150363943 |
| TMEM161 | -0.00578018 | 0.10950394 | 0.150407422 |
| YIPF6 | -0.005813854 | 0.110222462 | 0.151343635 |
| CCT8 | -0.009504561 | 0.110397517 | 0.151533251 |
| DAP | 0.009559447 | 0.110610353 | 0.151774581 |
| SNHG14 | 0.009977059 | 0.110961604 | 0.152205613 |
| DMTN | -0.006003288 | 0.111141523 | 0.15240142 |
| LONP2 | 0.005989283 | 0.111770211 | 0.153212258 |
| RDX | 0.010833454 | 0.112205463 | 0.153757486 |
| ATP5F1D | 0.022631706 | 0.11234483 | 0.153897028 |
| PRR5 | -0.006965788 | 0.112478529 | 0.154028714 |
| EMG1 | -0.005416833 | 0.112656341 | 0.1542207 |
| NKTR | -0.005822282 | 0.113065744 | 0.154729489 |
| PPP6R3 | 0.00601909 | 0.113634763 | 0.155456299 |
| CPNE1 | -0.00631699 | 0.113905761 | 0.155775057 |
| HOXD-AS2 | -0.007350047 | 0.114529594 | 0.156575971 |
| COPG1 | -0.006083417 | 0.115291935 | 0.157537928 |
| ARHGDIA | -0.009994427 | 0.115347748 | 0.157537928 |
| OCEL1 | -0.007917984 | 0.115348503 | 0.157537928 |
| ZFAND2B | -0.006903736 | 0.115697209 | 0.157961557 |
| OPTN | 0.010164825 | 0.116026669 | 0.158358636 |
| KCTD3 | -0.005244512 | 0.117100037 | 0.159770434 |

| | | | |
|---|---|---|---|
| RBM5 | 0.005205024 | 0.11729845 | 0.159987906 |
| MRPL54 | -0.012438333 | 0.117369438 | 0.160031491 |
| SPAG9 | -0.010560512 | 0.117551666 | 0.160226672 |
| ZBED1 | -0.005499835 | 0.117624364 | 0.160272481 |
| AFDN | -0.006920883 | 0.11840152 | 0.161235158 |
| FAM192A | -0.005699513 | 0.118409527 | 0.161235158 |
| DNAJB14 | -0.005820088 | 0.118935568 | 0.161874288 |
| MSI2 | 0.007469075 | 0.118957861 | 0.161874288 |
| MCRIP2 | -0.01156824 | 0.119081548 | 0.161988834 |
| GPS1 | -0.008451669 | 0.119185974 | 0.162077113 |
| KIF3B | -0.005524393 | 0.119265899 | 0.162132024 |
| ITPRID2 | 0.007651953 | 0.119708712 | 0.162680053 |
| MAN1A2 | -0.006394053 | 0.120047097 | 0.163085851 |
| TIMM9 | -0.006657591 | 0.120514186 | 0.163666169 |
| PWWP3A | -0.005151841 | 0.120720655 | 0.163892281 |
| PHF20L1 | -0.005180034 | 0.121080029 | 0.164325759 |
| POLR2E | -0.009191585 | 0.121180122 | 0.16440718 |
| MAP1LC3E | -0.009187178 | 0.121787393 | 0.165176417 |
| ALPL | 0.006923506 | 0.122194925 | 0.165657119 |
| COPS6 | -0.009054539 | 0.122222631 | 0.165657119 |
| MPP1 | -0.006771723 | 0.1223021 | 0.16571005 |
| AAMP | -0.007602799 | 0.122533508 | 0.165968742 |
| CCNB1IP1 | -0.006654602 | 0.122872154 | 0.166372468 |
| RAB17 | 0.010401965 | 0.123465945 | 0.167121286 |
| CRBN | -0.006707721 | 0.123596082 | 0.167242224 |
| ALDOB | 0.052606613 | 0.123679612 | 0.167271961 |
| RPLP1 | 0.034579712 | 0.123699655 | 0.167271961 |
| PPIP5K2 | -0.005513584 | 0.123761553 | 0.167300484 |
| RAB7A | -0.009822691 | 0.124134396 | 0.167749184 |
| GOT2 | -0.006441958 | 0.125130514 | 0.169039574 |
| COX16 | -0.010273823 | 0.126239778 | 0.170481914 |
| CHTOP | -0.004915908 | 0.126463521 | 0.170727836 |
| NUMA1 | -0.008489164 | 0.127768528 | 0.17243284 |
| TAX1BP1 | 0.010964099 | 0.128502696 | 0.173319059 |
| SAFB | -0.005118663 | 0.128509742 | 0.173319059 |
| RNMT | -0.006528949 | 0.128647292 | 0.173447517 |
| SNX13 | 0.004907257 | 0.1289392 | 0.173783932 |
| EIF4G1 | -0.006988331 | 0.129014716 | 0.173828569 |
| PNPLA8 | -0.005600652 | 0.129130193 | 0.173927001 |
| RAB9A | -0.006021215 | 0.12948891 | 0.174352883 |
| MOB4 | -0.005532647 | 0.130610456 | 0.175805275 |
| APRT | -0.014839774 | 0.131127083 | 0.176442744 |
| COBLL1 | -0.008333463 | 0.131340254 | 0.176671602 |
| MRPL46 | -0.006915842 | 0.131585997 | 0.176944109 |
| SDHB | -0.011684553 | 0.131923408 | 0.177339663 |
| NR3C1 | 0.00753631 | 0.132034981 | 0.177393951 |
| HIF1A | -0.00674255 | 0.132050326 | 0.177393951 |

| | | | |
|---|---|---|---|
| TPGS1 | 0.007921534 | 0.132157921 | 0.17748034 |
| DHX30 | -0.006571103 | 0.132643264 | 0.178073799 |
| WIPI2 | -0.007003038 | 0.132735718 | 0.178139589 |
| PPP1R7 | -0.006660938 | 0.133163551 | 0.178655287 |
| IDNK | 0.00884668 | 0.133407279 | 0.178915712 |
| PRRC2B | -0.006610077 | 0.133444938 | 0.178915712 |
| SMDT1 | -0.015078659 | 0.134098418 | 0.179710497 |
| NAXE | -0.007227325 | 0.134125395 | 0.179710497 |
| HNRNPD | -0.008536094 | 0.134318825 | 0.179910874 |
| AASDHPPT | -0.006970558 | 0.137219654 | 0.183736309 |
| DOCK1 | 0.004933752 | 0.13754564 | 0.184112675 |
| SFXN2 | 0.008880393 | 0.137905027 | 0.184533489 |
| HECTD1 | -0.006330591 | 0.138053086 | 0.184669234 |
| MRPS12 | -0.00832339 | 0.138096554 | 0.184669234 |
| TFDP2 | -0.00795336 | 0.139102784 | 0.185954161 |
| GRINA | 0.008706286 | 0.139442005 | 0.186346878 |
| APOO | -0.005505336 | 0.141744596 | 0.189362283 |
| METTL26 | 0.010154977 | 0.142055712 | 0.189716097 |
| MVP | 0.008236944 | 0.142171883 | 0.189809418 |
| CLYBL | 0.007383505 | 0.142225043 | 0.18981858 |
| ACO2 | 0.011750378 | 0.142313395 | 0.189874688 |
| ID2 | 0.008119313 | 0.142575583 | 0.190162619 |
| TPD52 | -0.007454872 | 0.143269694 | 0.191026258 |
| NET1 | -0.00550845 | 0.144244195 | 0.192263069 |
| RPL32 | -0.031197027 | 0.144398 | 0.192405525 |
| ARFGAP2 | -0.005245361 | 0.144683223 | 0.192722942 |
| NDUFA3 | -0.017996273 | 0.144935385 | 0.192996128 |
| RPL36 | 0.027589388 | 0.145080779 | 0.193077219 |
| IL32 | 0.015679824 | 0.145090466 | 0.193077219 |
| EIF4EBP2 | 0.010344732 | 0.146059872 | 0.194304178 |
| SUCNR1 | 0.006703073 | 0.146236898 | 0.194476575 |
| ECHDC2 | -0.011884411 | 0.146561726 | 0.194845355 |
| CKS2 | -0.005977622 | 0.147446867 | 0.195958559 |
| PJA2 | -0.007865832 | 0.148118349 | 0.196787178 |
| PRDX1 | 0.023222377 | 0.14956911 | 0.198650259 |
| SLC39A5 | -0.008392891 | 0.149648272 | 0.19867751 |
| UGP2 | -0.010251752 | 0.149686544 | 0.19867751 |
| NINL | -0.00507318 | 0.149903479 | 0.198901056 |
| PET100 | -0.013551323 | 0.15079158 | 0.200014714 |
| RNF5 | -0.010644159 | 0.150851975 | 0.200030109 |
| MAPT | 0.008412881 | 0.150944621 | 0.200088246 |
| STAG2 | 0.006862677 | 0.151955955 | 0.201363741 |
| MLLT6 | -0.004998802 | 0.152667114 | 0.202240765 |
| CC2D2A | 0.005792015 | 0.153133363 | 0.20279289 |
| FBXL15 | -0.006966884 | 0.154566168 | 0.204571169 |
| TRIP12 | -0.006152602 | 0.154575971 | 0.204571169 |
| SLC5A3 | 0.005440109 | 0.154776061 | 0.204769877 |

| | | | |
|---|---|---|---|
| IER2 | -0.01279071 | 0.154875614 | 0.20483549 |
| NMRAL1 | -0.005244592 | 0.155660218 | 0.205806803 |
| FARSA | -0.004729898 | 0.155871103 | 0.205969727 |
| RBM42 | -0.005898095 | 0.155883918 | 0.205969727 |
| ASL | 0.009536757 | 0.156147066 | 0.206250957 |
| RAB5A | -0.00467784 | 0.156633879 | 0.206764362 |
| TPP1 | -0.00716957 | 0.156636612 | 0.206764362 |
| WDR26 | 0.004900556 | 0.156839835 | 0.206965987 |
| YES1 | -0.006010465 | 0.15804841 | 0.208453192 |
| BFAR | -0.005068814 | 0.15806853 | 0.208453192 |
| LARP1B | 0.004960373 | 0.158146273 | 0.208488656 |
| RMND1 | 0.006199756 | 0.158409168 | 0.208706581 |
| CDK18 | 0.005892612 | 0.158413385 | 0.208706581 |
| MAT2A | -0.010098843 | 0.15855053 | 0.208820165 |
| PAICS | -0.005423507 | 0.159036427 | 0.209392855 |
| PUM1 | 0.0071977 | 0.159915741 | 0.210482998 |
| POLD4 | -0.008658836 | 0.160531388 | 0.211225511 |
| USP15 | 0.004789398 | 0.160774823 | 0.211477952 |
| EPS15 | 0.005201709 | 0.161059383 | 0.211784307 |
| USP47 | 0.005177532 | 0.161498865 | 0.212294116 |
| POLR2B | -0.005384759 | 0.161738056 | 0.212473173 |
| ZNF358 | 0.006780376 | 0.161738725 | 0.212473173 |
| SUGT1 | -0.00635456 | 0.162059543 | 0.212782806 |
| NAGLU | -0.005576795 | 0.162127449 | 0.212782806 |
| TNFRSF14 | -0.006621227 | 0.162130118 | 0.212782806 |
| MALT1 | 0.004606035 | 0.16236059 | 0.213017093 |
| DNM2 | 0.006321435 | 0.162884766 | 0.21363645 |
| IFIT1 | -0.005772954 | 0.162992122 | 0.213708891 |
| ELAVL1 | -0.005659737 | 0.16518185 | 0.216510737 |
| SPTBN1 | -0.009565397 | 0.166155807 | 0.217717741 |
| JADE1 | 0.006517076 | 0.166210748 | 0.217720149 |
| OSBPL1A | -0.007141079 | 0.167470264 | 0.219299931 |
| OBSL1 | -0.004700303 | 0.169431508 | 0.221797313 |
| KIAA0319L | -0.004829782 | 0.169616037 | 0.221968002 |
| TMEM174 | -0.008327455 | 0.169703565 | 0.222011684 |
| ESPN | -0.008193308 | 0.171292205 | 0.224018514 |
| EMX2 | -0.010197371 | 0.17145055 | 0.224154099 |
| DNAJB2 | -0.005863204 | 0.172784846 | 0.225826544 |
| PABPN1 | -0.007685971 | 0.173383116 | 0.226536258 |
| COPS2 | -0.006884227 | 0.173776192 | 0.226977505 |
| AKR1C3 | -0.013576603 | 0.174000191 | 0.227197702 |
| RPS19BP1 | -0.011365641 | 0.174257883 | 0.227461739 |
| ABAT | -0.009181946 | 0.174388175 | 0.227559363 |
| ABHD14A | -0.00758108 | 0.174935223 | 0.228177428 |
| RAB1A | -0.010346824 | 0.17497313 | 0.228177428 |
| MOCS2 | -0.009729729 | 0.176875337 | 0.230520241 |
| ADSL | -0.004800679 | 0.176882116 | 0.230520241 |

| | | | |
|---|---|---|---|
| LRPPRC | 0.008808601 | 0.177150893 | 0.230797159 |
| BCR | -0.004935339 | 0.178361325 | 0.232300328 |
| SLC25A13 | -0.006544654 | 0.17843021 | 0.232316247 |
| PDCD10 | -0.006572561 | 0.178623261 | 0.232493768 |
| MEF2A | 0.005757372 | 0.178750972 | 0.232586158 |
| LARS | -0.006376223 | 0.179370391 | 0.233318085 |
| ZFAND1 | -0.004946158 | 0.17970032 | 0.233651471 |
| SNW1 | -0.004454312 | 0.179740668 | 0.233651471 |
| RHOC | -0.011079343 | 0.179813788 | 0.233672434 |
| GLYATL1 | -0.014902229 | 0.180057545 | 0.233915062 |
| MED13 | 0.005315537 | 0.181210513 | 0.235338329 |
| COX17 | 0.012256761 | 0.181293761 | 0.235371887 |
| SMPD1 | -0.005194947 | 0.181698904 | 0.235823205 |
| WDR82 | -0.00445432 | 0.181811814 | 0.235895075 |
| SNTB2 | 0.005567352 | 0.182059216 | 0.236131372 |
| CYP4A11 | -0.009599814 | 0.182109121 | 0.236131372 |
| NFU1 | -0.005603497 | 0.182362473 | 0.236385121 |
| ATF7 | 0.004471212 | 0.183622859 | 0.237853013 |
| PPP4R3B | -0.006895279 | 0.18362837 | 0.237853013 |
| ZNF518A | -0.004542533 | 0.183668936 | 0.237853013 |
| TPT1 | 0.036440646 | 0.183927195 | 0.238112252 |
| EPB41L3 | 0.007736509 | 0.184318191 | 0.238543114 |
| HSPH1 | -0.005567214 | 0.18534424 | 0.239795325 |
| NR1D2 | -0.004440702 | 0.18559311 | 0.240041562 |
| HNRNPUL1 | -0.005323733 | 0.186442601 | 0.241064227 |
| FLOT1 | -0.006667403 | 0.18661859 | 0.241215706 |
| UBE3A | -0.006101593 | 0.187022671 | 0.241661819 |
| AZI2 | -0.004725674 | 0.187909442 | 0.242731164 |
| APPBP2 | 0.00438293 | 0.18838775 | 0.24327237 |
| MZT2A | -0.008338258 | 0.18879492 | 0.243721402 |
| PMVK | -0.008192897 | 0.188935376 | 0.24382595 |
| NUP58 | -0.004752881 | 0.189396837 | 0.244344566 |
| ERF | -0.004727073 | 0.189567908 | 0.244488337 |
| OGDH | -0.008890297 | 0.19050493 | 0.245619564 |
| HIBADH | 0.009606261 | 0.190756753 | 0.245866925 |
| TCL6 | 0.007017621 | 0.191833463 | 0.247176995 |
| PTAR1 | 0.006015603 | 0.19238154 | 0.247805314 |
| KIF13B | 0.004622888 | 0.193542451 | 0.249222377 |
| CCDC106 | -0.005518311 | 0.193737459 | 0.249395159 |
| RASSF8 | 0.005227922 | 0.194462351 | 0.25024973 |
| CLCN5 | -0.007896581 | 0.195924755 | 0.252052556 |
| SLC12A7 | -0.005863994 | 0.196505184 | 0.252719967 |
| PWP1 | -0.004565732 | 0.196881509 | 0.253124549 |
| RTKN | -0.006419596 | 0.197947255 | 0.254414968 |
| PRPF4B | -0.006363946 | 0.198138459 | 0.25458091 |
| HYI | 0.007508682 | 0.198677542 | 0.255193585 |
| EXOSC6 | -0.005307556 | 0.198810075 | 0.255283841 |

| | | | |
|---|---|---|---|
| PPP2R2A | -0.004924182 | 0.199978373 | 0.25670361 |
| ACVR1B | -0.004792387 | 0.200455914 | 0.257236071 |
| RPL12 | -0.025088229 | 0.200850832 | 0.257654128 |
| AK2 | -0.00907533 | 0.200907377 | 0.257654128 |
| FUZ | -0.006427366 | 0.203641038 | 0.261078254 |
| BRWD1 | 0.004657093 | 0.205763228 | 0.263716548 |
| MRPS34 | -0.009503256 | 0.206098754 | 0.264030197 |
| USP9X | 0.00592623 | 0.206136747 | 0.264030197 |
| BLOC1S1 | -0.014787784 | 0.206677548 | 0.264640208 |
| PPM1A | 0.006226423 | 0.206872399 | 0.264807005 |
| U2SURP | 0.005482128 | 0.207659858 | 0.265730696 |
| MECP2 | 0.004602753 | 0.207772641 | 0.265730696 |
| PPIE | -0.004376167 | 0.207788441 | 0.265730696 |
| NTMT1 | -0.004951394 | 0.209402764 | 0.267711672 |
| RIN2 | 0.005425565 | 0.210017631 | 0.268414054 |
| ALG13 | -0.00454687 | 0.21085317 | 0.269397942 |
| USH1C | -0.008272486 | 0.21202685 | 0.27081311 |
| TM2D1 | -0.004232506 | 0.212629677 | 0.271498497 |
| SLC7A9 | -0.00679723 | 0.213295096 | 0.272263354 |
| CNPPD1 | -0.005538002 | 0.213441923 | 0.272365977 |
| POLR2F | -0.007433255 | 0.213926178 | 0.272784208 |
| RPL39 | 0.026143766 | 0.213956838 | 0.272784208 |
| PDK3 | -0.004613465 | 0.213969272 | 0.272784208 |
| SLTM | -0.00544949 | 0.214162125 | 0.272945202 |
| HEXIM1 | -0.00552764 | 0.214381798 | 0.273140265 |
| SCYL2 | -0.004764036 | 0.214593613 | 0.273325198 |
| ESD | -0.010319828 | 0.215148537 | 0.273946895 |
| H2AFZ | -0.010306513 | 0.215946844 | 0.274878007 |
| MLX | -0.004504575 | 0.217421492 | 0.276669186 |
| PXMP2 | -0.010923355 | 0.218053055 | 0.277386759 |
| GSS | -0.006236028 | 0.218295513 | 0.277609058 |
| CCDC12 | -0.004690993 | 0.219108646 | 0.278556728 |
| BORCS8 | -0.004092746 | 0.219749574 | 0.279284951 |
| GFER | -0.004731136 | 0.220282205 | 0.279875129 |
| NDUFS3 | -0.008247847 | 0.221166019 | 0.280910991 |
| SNRPB | -0.006383509 | 0.221715345 | 0.28152094 |
| ITGB3BP | 0.004145709 | 0.22183843 | 0.28152094 |
| RBL2 | -0.004310628 | 0.221852233 | 0.28152094 |
| LGR4 | 0.005067117 | 0.222082501 | 0.281725945 |
| GOLGA4 | 0.007423086 | 0.222920927 | 0.282702073 |
| MAT2B | 0.006360113 | 0.22329641 | 0.283090687 |
| XPO7 | 0.004203638 | 0.224256475 | 0.284219952 |
| PPP1CC | -0.005669515 | 0.224397086 | 0.284310276 |
| STXBP2 | 0.00525764 | 0.224495003 | 0.284346467 |
| PPA2 | 0.006568563 | 0.225455902 | 0.285475354 |
| MYO6 | -0.008774465 | 0.226155317 | 0.286272553 |
| ATXN10 | -0.005103794 | 0.22631315 | 0.286383925 |

| | | | |
|---|---|---|---|
| CLUH | 0.005548894 | 0.227330633 | 0.28758272 |
| EIF4B | 0.011058836 | 0.228248902 | 0.288655304 |
| DDX3X | -0.007320732 | 0.228407483 | 0.288766784 |
| ABCC6 | -0.005621614 | 0.228539209 | 0.288844253 |
| SRSF1 | -0.005181005 | 0.228829293 | 0.289121757 |
| FBXO34 | 0.003936723 | 0.228971369 | 0.289212142 |
| EPB41L1 | 0.005366634 | 0.2293766 | 0.289634758 |
| POLD3 | -0.004510696 | 0.229669272 | 0.289915029 |
| PAWR | -0.005327159 | 0.230940736 | 0.291430292 |
| UBE2K | 0.006163084 | 0.231806643 | 0.292432995 |
| TAOK1 | 0.004382844 | 0.232137121 | 0.292759827 |
| RBM8A | -0.007393443 | 0.234029074 | 0.295035909 |
| HOXC10 | -0.005817322 | 0.234085808 | 0.295035909 |
| CLTC | 0.009145266 | 0.235541807 | 0.296779781 |
| MAGED1 | -0.004719265 | 0.237546492 | 0.299213708 |
| FUBP1 | -0.003813146 | 0.238334706 | 0.300114341 |
| IST1 | 0.005611198 | 0.238649514 | 0.300418486 |
| WDR18 | 0.006862049 | 0.238778602 | 0.300488725 |
| HAO2 | 0.011448237 | 0.239770754 | 0.301644704 |
| SMIM1 | -0.007189744 | 0.240476865 | 0.302440229 |
| ATIC | -0.004274382 | 0.240750733 | 0.302691814 |
| DLD | -0.006356064 | 0.242422533 | 0.304700302 |
| ACTR2 | 0.007303563 | 0.242530985 | 0.304743193 |
| LDHD | -0.005420758 | 0.243618217 | 0.306015529 |
| SHLD2 | 0.006009512 | 0.245102611 | 0.307785821 |
| AC010642. | -0.005326404 | 0.246468318 | 0.309406033 |
| C8orf33 | -0.004124178 | 0.247572068 | 0.310696504 |
| LYPLA2 | -0.005506936 | 0.248962676 | 0.312279782 |
| GSTO1 | -0.009845152 | 0.248986002 | 0.312279782 |
| LAMTOR4 | -0.011024131 | 0.249703167 | 0.313083481 |
| PRRC2A | -0.003842952 | 0.25101189 | 0.314628172 |
| DPYSL2 | 0.005016179 | 0.251925799 | 0.315677193 |
| RNASEH2C | 0.008660649 | 0.252573042 | 0.316391528 |
| TARS | -0.004217817 | 0.256102237 | 0.320714469 |
| OSBPL9 | 0.005353913 | 0.256229753 | 0.320776179 |
| TRAPPC2L | 0.007586697 | 0.256807976 | 0.321240536 |
| SRSF4 | -0.003714566 | 0.25681512 | 0.321240536 |
| SEPTIN10 | 0.0041484 | 0.256835726 | 0.321240536 |
| TRA2B | -0.004772951 | 0.256919725 | 0.321247597 |
| EIF4A2 | -0.010782025 | 0.257657572 | 0.322071965 |
| DYNC1LI1 | -0.004121393 | 0.259216945 | 0.323922424 |
| TTYH3 | -0.004957191 | 0.259860527 | 0.324546413 |
| TMEM160 | -0.008001349 | 0.259874603 | 0.324546413 |
| DDX10 | 0.006614 | 0.260465813 | 0.325185698 |
| BROX | 0.004760472 | 0.26091909 | 0.325652441 |
| COX8A | -0.01758488 | 0.261769315 | 0.326614179 |
| IBTK | -0.003900512 | 0.261856131 | 0.326623102 |

| | | | |
|---|---|---|---|
| NAP1L1 | 0.006994758 | 0.262398226 | 0.327199734 |
| SNAP23 | -0.003920622 | 0.263183268 | 0.328078869 |
| RBKS | 0.005087775 | 0.263388523 | 0.328234938 |
| PPARA | -0.006321149 | 0.263563263 | 0.328352896 |
| ADNP | -0.004080664 | 0.263942855 | 0.328706989 |
| TOMM70 | -0.004740624 | 0.264007833 | 0.328706989 |
| MRPS5 | -0.004636789 | 0.265527361 | 0.330498537 |
| UFM1 | -0.006972134 | 0.265645858 | 0.33054568 |
| VMA21 | -0.004062648 | 0.265954094 | 0.330828818 |
| SYNCRIP | 0.005518595 | 0.266875582 | 0.331874397 |
| UCHL5 | 0.003811652 | 0.267167348 | 0.332136485 |
| TMEM19 | -0.003920194 | 0.267780828 | 0.332757906 |
| EIF3A | -0.006227576 | 0.267829534 | 0.332757906 |
| PRPF8 | -0.004915268 | 0.268027958 | 0.332903553 |
| MICU2 | 0.006113965 | 0.26834909 | 0.333201475 |
| CRLS1 | 0.005407676 | 0.268839711 | 0.333709602 |
| SLC25A46 | 0.004164973 | 0.270899791 | 0.336164994 |
| TLN2 | 0.006925653 | 0.271059062 | 0.336260863 |
| DRAM1 | -0.003582915 | 0.271927923 | 0.337236687 |
| LRRFIP2 | -0.003693395 | 0.272118449 | 0.337370922 |
| ASB13 | -0.004468849 | 0.272656572 | 0.337935896 |
| PGRMC2 | -0.005464251 | 0.273803874 | 0.339255329 |
| MPDZ | 0.004119994 | 0.274694524 | 0.340256057 |
| SAR1B | -0.006436578 | 0.276961666 | 0.342960686 |
| VPS28 | -0.009958409 | 0.277320246 | 0.343301029 |
| MICU1 | 0.006654553 | 0.277775278 | 0.343760531 |
| TDP2 | -0.006482827 | 0.278265743 | 0.344263593 |
| TBC1D13 | -0.004760962 | 0.278575393 | 0.344542718 |
| AC010913. | -0.004420469 | 0.279013854 | 0.344876937 |
| TADA3 | -0.003995648 | 0.279013795 | 0.344876937 |
| SFPQ | -0.006499271 | 0.279840916 | 0.345794983 |
| RBBP6 | 0.003487884 | 0.280118601 | 0.346033824 |
| OAZ2 | -0.003732288 | 0.280432654 | 0.346317435 |
| NUDT12 | 0.004026786 | 0.282066765 | 0.348230574 |
| SMARCC2 | -0.003595203 | 0.2823356 | 0.34836728 |
| THAP4 | -0.004422024 | 0.282347432 | 0.34836728 |
| TOM1L1 | -0.005301546 | 0.282483937 | 0.348430848 |
| GIGYF2 | 0.003613696 | 0.283275481 | 0.349302096 |
| CEBPZOS | -0.005670561 | 0.283861488 | 0.349919453 |
| LAMB2 | -0.004504036 | 0.284112347 | 0.350123422 |
| POGZ | 0.003793698 | 0.284295071 | 0.350225612 |
| JUP | -0.004997683 | 0.284368133 | 0.350225612 |
| CMC1 | -0.005431162 | 0.284451534 | 0.350225612 |
| SUZ12 | -0.004071926 | 0.285517804 | 0.351432901 |
| FLOT2 | 0.003829833 | 0.285894213 | 0.351790599 |
| RCAN1 | -0.004826956 | 0.287568386 | 0.353744489 |
| CUX1 | 0.006594219 | 0.287685134 | 0.353781958 |

| | | | |
|---|---|---|---|
| CEP350 | 0.004352868 | 0.287795151 | 0.35381113 |
| CRYL1 | 0.011812296 | 0.28828792 | 0.354310692 |
| MYH10 | -0.003672936 | 0.289092089 | 0.355192558 |
| DHRS1 | 0.003775887 | 0.28999957 | 0.35620079 |
| EIF3G | -0.00791489 | 0.290242352 | 0.356392226 |
| NDUFB11 | -0.010207174 | 0.292314101 | 0.358828686 |
| HNRNPK | -0.009916206 | 0.292772073 | 0.359283299 |
| DHX9 | -0.004644787 | 0.292889874 | 0.359320312 |
| PPP1R10 | -0.004559281 | 0.294753926 | 0.361498981 |
| MRPL45 | -0.003841045 | 0.295180081 | 0.361913377 |
| ZMYM2 | 0.003832356 | 0.295589627 | 0.362307166 |
| PARD6B | 0.007324804 | 0.29651493 | 0.363332699 |
| GTF2I | -0.008087325 | 0.297168956 | 0.364025312 |
| GLYCTK | -0.004646756 | 0.297459781 | 0.364272731 |
| EIF3B | -0.004243022 | 0.297886528 | 0.364686404 |
| ATP5MD | -0.015579637 | 0.2982711 | 0.365048212 |
| AGPS | 0.003804652 | 0.298612142 | 0.365356545 |
| FRA10AC1 | -0.00354123 | 0.29971365 | 0.366594858 |
| POLE4 | -0.005123589 | 0.300660491 | 0.367643309 |
| SNHG7 | 0.006026088 | 0.301592883 | 0.368673471 |
| STX7 | -0.006186205 | 0.301723839 | 0.368723618 |
| DDAH2 | -0.006098765 | 0.302015162 | 0.368969655 |
| NCKAP1 | -0.005661704 | 0.303662863 | 0.370819249 |
| TEN1 | -0.003773319 | 0.303765438 | 0.370819249 |
| TOLLIP | -0.005937754 | 0.303800453 | 0.370819249 |
| ATMIN | -0.003762308 | 0.304000052 | 0.370952444 |
| SP1 | 0.00381492 | 0.304328814 | 0.371216517 |
| SLC17A3 | 0.006011618 | 0.304397544 | 0.371216517 |
| MCCC2 | -0.005216658 | 0.304821079 | 0.371579868 |
| ITPA | -0.003883984 | 0.30487675 | 0.371579868 |
| TIMM10 | -0.006681624 | 0.305836065 | 0.372638296 |
| PPFIA1 | -0.00347623 | 0.306321729 | 0.373119159 |
| UBR2 | 0.003435085 | 0.306734516 | 0.373510994 |
| GJB1 | -0.004019095 | 0.307632963 | 0.374493809 |
| STARD10 | 0.004745822 | 0.308095379 | 0.3749454 |
| GALM | 0.007411892 | 0.308322152 | 0.375110037 |
| GMFB | -0.003557929 | 0.309765993 | 0.376754842 |
| ARL6IP4 | -0.010618142 | 0.31064529 | 0.377712245 |
| PTP4A2 | -0.006014839 | 0.310900306 | 0.377803501 |
| PNPLA2 | -0.004100681 | 0.310904637 | 0.377803501 |
| TJP1 | -0.003915487 | 0.311097945 | 0.377926392 |
| PSMG4 | -0.003741035 | 0.312375003 | 0.379342164 |
| MARCH6 | -0.005080715 | 0.312448412 | 0.379342164 |
| BTBD2 | -0.003996624 | 0.314933342 | 0.38224268 |
| SP3 | 0.003513845 | 0.315071492 | 0.38224268 |
| SQSTM1 | -0.009325847 | 0.315117136 | 0.38224268 |
| HNRNPH2 | -0.004735286 | 0.315386711 | 0.382456526 |

| | | | |
|---|---|---|---|
| SLC38A10 | -0.00379003 | 0.316025809 | 0.383118219 |
| SLC6A19 | 0.00917021 | 0.31719067 | 0.384416715 |
| HIST1H2A( | -0.005002298 | 0.318680166 | 0.386107767 |
| RABEP1 | -0.003444849 | 0.319207095 | 0.386631932 |
| SMARCA1 | -0.00364664 | 0.319537738 | 0.386840397 |
| UBA52 | 0.014803572 | 0.319567908 | 0.386840397 |
| SH3GLB1 | -0.005003466 | 0.31968178 | 0.386864019 |
| DTNBP1 | 0.003450385 | 0.320099767 | 0.387255546 |
| ACSS2 | -0.004903615 | 0.322254191 | 0.389746956 |
| CMIP | 0.004634727 | 0.322812765 | 0.390307383 |
| STX3 | 0.005239955 | 0.323393445 | 0.3908942 |
| MRPL23 | 0.00445272 | 0.324033705 | 0.391552664 |
| NUPR1 | -0.009302317 | 0.324478797 | 0.391974976 |
| WAPL | 0.003666507 | 0.324602816 | 0.392009292 |
| GPBP1 | -0.005015443 | 0.324793669 | 0.392124277 |
| ACTR6 | 0.00352346 | 0.326697367 | 0.394306507 |
| ANGPTL3 | -0.006944145 | 0.327888396 | 0.395627552 |
| MRPL24 | -0.004713797 | 0.32885086 | 0.396672117 |
| COPA | -0.004630782 | 0.329785315 | 0.397682291 |
| AKAP11 | -0.003683694 | 0.330016435 | 0.397843982 |
| AP3B1 | -0.003644312 | 0.331116014 | 0.399052222 |
| DHRS4L2 | -0.011038601 | 0.331494844 | 0.399391379 |
| LETMD1 | -0.003427162 | 0.331802081 | 0.399644105 |
| EMC2 | -0.004313002 | 0.334537131 | 0.40282004 |
| CCT7 | 0.004872458 | 0.334981747 | 0.403236983 |
| SLC30A1 | -0.003263621 | 0.336217468 | 0.4046057 |
| WDR13 | -0.005457145 | 0.336472582 | 0.404793893 |
| RPS24 | -0.020036082 | 0.336602632 | 0.404831561 |
| LUC7L2 | -0.004124768 | 0.336888874 | 0.405006622 |
| ROCK2 | -0.003524725 | 0.336950775 | 0.405006622 |
| LINC00467 | -0.003969865 | 0.337044535 | 0.405006622 |
| BCLAF1 | -0.003951995 | 0.337692024 | 0.405665777 |
| ATRX | -0.00492581 | 0.338760898 | 0.406830604 |
| MRPL2 | -0.004913606 | 0.340695204 | 0.408978299 |
| AHCYL1 | -0.005999369 | 0.340748748 | 0.408978299 |
| CCT4 | 0.005855805 | 0.341727754 | 0.409965624 |
| YBEY | -0.003383188 | 0.341771342 | 0.409965624 |
| MIF | 0.017158344 | 0.345102655 | 0.413840334 |
| USP8 | -0.004208054 | 0.345231194 | 0.413840334 |
| NSMF | -0.004508898 | 0.345304337 | 0.413840334 |
| TPRKB | -0.004513142 | 0.346927394 | 0.415664032 |
| TMEM256 | -0.009700839 | 0.34763793 | 0.416393664 |
| HSPA9 | -0.007143684 | 0.347910922 | 0.416598943 |
| YTHDF3 | 0.003739291 | 0.348764344 | 0.417498923 |
| ATP6V1C1 | -0.004715359 | 0.349941392 | 0.41878567 |
| TRADD | -0.003664291 | 0.351493638 | 0.420520548 |
| ATF6 | -0.003291983 | 0.352082521 | 0.421102198 |

| | | | |
|---|---|---|---|
| VTI1B | -0.007588702 | 0.35219197 | 0.421110259 |
| AGFG1 | -0.003406218 | 0.352376729 | 0.421208335 |
| ALKBH7 | -0.008650266 | 0.355228585 | 0.4244935 |
| LARP6 | 0.00372497 | 0.356744734 | 0.426066675 |
| ARCN1 | -0.003642411 | 0.356752902 | 0.426066675 |
| TMEM203 | -0.003316099 | 0.357161845 | 0.426327847 |
| CLPX | -0.003475741 | 0.35717955 | 0.426327847 |
| EWSR1 | -0.003953419 | 0.357839313 | 0.426991031 |
| PTPN3 | 0.003193449 | 0.358254175 | 0.427361687 |
| TUBB | 0.006099342 | 0.359818509 | 0.429102934 |
| RAB5B | 0.003194908 | 0.360752743 | 0.430091958 |
| EIF4EBP1 | -0.003644851 | 0.362768662 | 0.432369626 |
| CHD2 | -0.00373899 | 0.364122855 | 0.433857514 |
| EBP | -0.004033566 | 0.36483488 | 0.434579607 |
| SKAP2 | 0.004650388 | 0.366450004 | 0.436376711 |
| RMND5A | -0.003148958 | 0.367076577 | 0.436995925 |
| AZGP1 | -0.007189141 | 0.367267408 | 0.437096189 |
| MAP4K3-D | 0.003921892 | 0.368155309 | 0.43802576 |
| TAGLN2 | 0.004878312 | 0.369152398 | 0.439060082 |
| FRMD4B | 0.004076226 | 0.369261631 | 0.439060082 |
| LARP4 | 0.004896768 | 0.369345908 | 0.439060082 |
| CHMP3 | -0.005681322 | 0.37225741 | 0.442392864 |
| RPS6KA3 | 0.00371585 | 0.372688811 | 0.442777203 |
| USO1 | -0.003527937 | 0.373470819 | 0.443577739 |
| PRKACB | -0.003318738 | 0.374623611 | 0.444818073 |
| AHCTF1 | -0.003005364 | 0.375764599 | 0.446043676 |
| COPB1 | 0.004614996 | 0.376397167 | 0.446665234 |
| SMIM2-AS | -0.004119497 | 0.37680347 | 0.447018005 |
| MAP3K13 | -0.006189685 | 0.378035326 | 0.448230838 |
| CSPP1 | 0.003550206 | 0.378044449 | 0.448230838 |
| FAM207A | -0.003452447 | 0.379012868 | 0.449249135 |
| KHDRBS1 | -0.005283281 | 0.379971381 | 0.450255104 |
| CUL5 | -0.004543337 | 0.380419648 | 0.450656041 |
| MMADHC | 0.00400656 | 0.380714685 | 0.450875277 |
| TCAIM | 0.003276574 | 0.382548117 | 0.452915761 |
| A1CF | -0.004770513 | 0.383338437 | 0.453720436 |
| RARS | -0.003010605 | 0.383982359 | 0.45435142 |
| VPS4A | -0.003720044 | 0.384671515 | 0.455035549 |
| ABHD14B | -0.005487688 | 0.385117154 | 0.455431304 |
| ASS1 | 0.016091741 | 0.385339804 | 0.455563206 |
| TSKU | -0.003652852 | 0.385555978 | 0.455687377 |
| SNRPB2 | -0.005827191 | 0.387026661 | 0.457293749 |
| METAP2 | -0.004422476 | 0.388054654 | 0.458333141 |
| GRPEL1 | -0.004885048 | 0.388129919 | 0.458333141 |
| QKI | 0.003975348 | 0.390326222 | 0.460671216 |
| BLVRB | -0.007661325 | 0.390429307 | 0.460671216 |
| AP3S1 | -0.003208744 | 0.390446945 | 0.460671216 |

| | | | |
|---|---|---|---|
| CBX3 | 0.005278835 | 0.39129204 | 0.46153549 |
| SPOP | -0.003156229 | 0.392085903 | 0.462338856 |
| MME | 0.010521645 | 0.39248635 | 0.462677986 |
| CALCOCO2 | -0.003321997 | 0.392862484 | 0.462988268 |
| RBBP4 | -0.004397338 | 0.393374216 | 0.463458128 |
| DBT | -0.003523539 | 0.394603117 | 0.464772416 |
| FCAMR | -0.003099893 | 0.394819403 | 0.464893611 |
| LMO4 | -0.003076624 | 0.395305323 | 0.465332135 |
| IRF2BP2 | 0.004160054 | 0.395718431 | 0.465684721 |
| LPAR6 | 0.003011779 | 0.39654141 | 0.466519306 |
| RSBN1L | 0.002911646 | 0.397319499 | 0.467300616 |
| SMARCC1 | 0.004008108 | 0.397505076 | 0.467384805 |
| UGDH | -0.003915497 | 0.398122592 | 0.467976671 |
| BMPR2 | 0.002759203 | 0.40043083 | 0.470555003 |
| CTDNEP1 | -0.00354266 | 0.400844715 | 0.470813276 |
| PPP1CA | -0.004823134 | 0.400880279 | 0.470813276 |
| GULP1 | 0.002942105 | 0.402194626 | 0.47222164 |
| SND1 | -0.002895846 | 0.402514833 | 0.47232817 |
| FBL | 0.00307012 | 0.402515762 | 0.47232817 |
| DDAH1 | 0.006993776 | 0.403012211 | 0.472690311 |
| DHPS | 0.003288364 | 0.403054958 | 0.472690311 |
| PMF1 | 0.003815104 | 0.403632328 | 0.473117901 |
| BRI3 | -0.010857339 | 0.403650345 | 0.473117901 |
| PSMD3 | -0.002868103 | 0.404005886 | 0.473399295 |
| LIN7C | -0.003662015 | 0.404335067 | 0.47364965 |
| SAMM50 | -0.003819752 | 0.405291415 | 0.474634334 |
| NEDD9 | -0.003130859 | 0.406341091 | 0.475727719 |
| WWC2 | 0.003412488 | 0.40698996 | 0.476332647 |
| MAP4 | -0.003299623 | 0.407161123 | 0.476332647 |
| TFEC | -0.005266093 | 0.407206323 | 0.476332647 |
| DCUN1D1 | -0.00287795 | 0.409645268 | 0.479048944 |
| ARMC1 | -0.003080578 | 0.409897516 | 0.479207247 |
| GALNT14 | 0.003862496 | 0.410269588 | 0.479505505 |
| OGT | 0.002868665 | 0.410552189 | 0.479699053 |
| DDX56 | -0.002763401 | 0.411667296 | 0.480864933 |
| AHCY | 0.006078666 | 0.41618365 | 0.486001984 |
| CRK | -0.003125912 | 0.416607512 | 0.486358428 |
| MRPS31 | -0.003428593 | 0.416950541 | 0.48662033 |
| ACADSB | -0.005777978 | 0.417280473 | 0.486866801 |
| ST3GAL1 | -0.003661116 | 0.417760808 | 0.487288567 |
| COX7A1 | -0.004042679 | 0.418365071 | 0.487854605 |
| RAB40B | 0.003348075 | 0.419172498 | 0.488657163 |
| LLPH | 0.003206104 | 0.420170771 | 0.489681683 |
| RNPEPL1 | 0.002851104 | 0.421087062 | 0.490610103 |
| PDSS2 | 0.002926486 | 0.422066549 | 0.491611605 |
| OAT | 0.004866844 | 0.423008138 | 0.492568409 |
| ACBD6 | 0.002706865 | 0.423311114 | 0.492629133 |

| | | | |
|---|---|---|---|
| CGGBP1 | 0.003640095 | 0.423312169 | 0.492629133 |
| CLK1 | -0.002756073 | 0.423420747 | 0.492629133 |
| NRP1 | 0.003423835 | 0.424598633 | 0.493859402 |
| EPB41L4A- | -0.004243244 | 0.424723221 | 0.493864211 |
| SLC25A6 | 0.009394444 | 0.425925029 | 0.495010577 |
| HSD17B14 | -0.006251137 | 0.426003029 | 0.495010577 |
| MRPL38 | -0.004372869 | 0.426071299 | 0.495010577 |
| SLC16A4 | 0.007170255 | 0.426355647 | 0.49520061 |
| SORBS3 | 0.002720613 | 0.426962427 | 0.495764926 |
| NUDT21 | -0.002880088 | 0.428294084 | 0.497170369 |
| ACADM | 0.008265383 | 0.431433732 | 0.500673167 |
| DCTN4 | 0.002904873 | 0.432434391 | 0.501692418 |
| GGH | -0.005024433 | 0.433112585 | 0.502247578 |
| HDAC6 | 0.003452254 | 0.433157912 | 0.502247578 |
| RASSF8-AS | -0.003892409 | 0.435110396 | 0.50436885 |
| CTNND1 | 0.003764227 | 0.435502703 | 0.504550675 |
| ACSM3 | 0.004160849 | 0.435513376 | 0.504550675 |
| RAB22A | -0.002692187 | 0.436440553 | 0.505481997 |
| CRYZ | 0.005742637 | 0.437413969 | 0.50646633 |
| ILRUN | 0.00285065 | 0.4382074 | 0.507241767 |
| ZC3HAV1 | -0.002764995 | 0.438382572 | 0.507301311 |
| GAS5 | -0.004957721 | 0.438638534 | 0.507454286 |
| ETHE1 | 0.003112299 | 0.439599295 | 0.508422316 |
| CNOT4 | -0.002672109 | 0.440715552 | 0.509569589 |
| BOK | 0.00296288 | 0.441200924 | 0.509986971 |
| GAMT | -0.007085899 | 0.443029408 | 0.511956193 |
| ANKRD10 | -0.002720696 | 0.443703016 | 0.512590128 |
| SNRPD2 | -0.005472637 | 0.445411849 | 0.514419319 |
| GRSF1 | -0.003079791 | 0.445582449 | 0.514471428 |
| SF3B5 | -0.006390948 | 0.447084833 | 0.515973173 |
| ITGAV | -0.003080453 | 0.447295596 | 0.515973173 |
| AIP | -0.0037796 | 0.447308552 | 0.515973173 |
| NOSIP | -0.003158807 | 0.447386495 | 0.515973173 |
| HNRNPL | -0.003083293 | 0.448418574 | 0.517018041 |
| AZIN1 | 0.00272107 | 0.448763954 | 0.517270793 |
| RING1 | -0.002581987 | 0.449351462 | 0.517802415 |
| NOL11 | -0.002629725 | 0.450269469 | 0.518708622 |
| UBE2G1 | -0.002946601 | 0.450390901 | 0.518708622 |
| UBA1 | -0.004084403 | 0.450696748 | 0.518915098 |
| COASY | -0.002693268 | 0.451463136 | 0.519631914 |
| CAPRIN1 | -0.003668515 | 0.451572808 | 0.519631914 |
| HNRNPF | -0.004860344 | 0.451840404 | 0.519793955 |
| PSMB4 | -0.005049145 | 0.452888204 | 0.520853194 |
| AARS | -0.00277505 | 0.453942342 | 0.521848843 |
| IDH3B | -0.003836897 | 0.454008493 | 0.521848843 |
| LHPP | 0.003072401 | 0.454767537 | 0.522574804 |
| RNF186 | -0.003842649 | 0.455043493 | 0.522745397 |

| | | | |
|---|---|---|---|
| R3HDM2 | 0.003264162 | 0.455209648 | 0.522789792 |
| PEPD | -0.011303171 | 0.457225032 | 0.524957332 |
| PABPC1 | 0.007081565 | 0.457592007 | 0.52508605 |
| B4GALT5 | -0.002940353 | 0.457593282 | 0.52508605 |
| RSRC1 | -0.002463445 | 0.460448136 | 0.528214146 |
| ECSIT | -0.003199815 | 0.460967409 | 0.528618791 |
| BEND7 | -0.003202844 | 0.461058731 | 0.528618791 |
| LINC01510 | -0.004237014 | 0.461296681 | 0.52874375 |
| KARS | -0.002737409 | 0.461861309 | 0.529242976 |
| PAM16 | -0.003146441 | 0.463298742 | 0.530741784 |
| ACADL | -0.002516161 | 0.463753554 | 0.531114405 |
| PLPPR1 | -0.002969221 | 0.464168331 | 0.531440982 |
| MAF1 | -0.004207714 | 0.46545938 | 0.532770368 |
| UROD | -0.003284288 | 0.466460748 | 0.533767532 |
| SLC9A3R1 | -0.00663601 | 0.46840562 | 0.535843483 |
| KIF1C | -0.003100371 | 0.468863814 | 0.53621803 |
| HSPB11 | -0.003478845 | 0.469082521 | 0.536318555 |
| DPP8 | 0.002473299 | 0.469898703 | 0.537101946 |
| UGT2A3 | 0.006798802 | 0.470476534 | 0.537612539 |
| GDI2 | -0.004996928 | 0.470623007 | 0.537630072 |
| FBXW4 | 0.002448267 | 0.470820426 | 0.537661406 |
| SENP6 | 0.003098612 | 0.470912709 | 0.537661406 |
| TULP4 | -0.002440623 | 0.472617832 | 0.539457993 |
| MED29 | -0.002821339 | 0.473719583 | 0.540565069 |
| ABI1 | 0.002538454 | 0.474404358 | 0.541195845 |
| UBL3 | 0.003132801 | 0.4750755 | 0.541810723 |
| HOOK3 | -0.002421858 | 0.47560527 | 0.542264073 |
| CYP4V2 | 0.002983804 | 0.476206768 | 0.542798929 |
| ARID4B | -0.002374071 | 0.476615428 | 0.543113745 |
| LRRC58 | -0.003122913 | 0.479146413 | 0.54584615 |
| LARP7 | -0.003111505 | 0.47988999 | 0.546541378 |
| DDX42 | -0.002328598 | 0.483311 | 0.550199856 |
| UBALD2 | 0.002448323 | 0.483370703 | 0.550199856 |
| AMD1 | 0.003590424 | 0.484962871 | 0.551858943 |
| LRATD2 | -0.003200801 | 0.485213677 | 0.551991142 |
| RTF1 | -0.003329981 | 0.48713222 | 0.554020001 |
| SLC22A8 | -0.008510806 | 0.487986145 | 0.554793593 |
| DECR2 | -0.004096318 | 0.488083046 | 0.554793593 |
| SUPT5H | -0.002483922 | 0.488224616 | 0.5548007 |
| DHRSX | 0.002340681 | 0.490897733 | 0.557683765 |
| EP300 | 0.002541115 | 0.491863507 | 0.558626144 |
| LAMTOR3 | -0.003447263 | 0.492662152 | 0.559378239 |
| VIL1 | 0.002477573 | 0.493420465 | 0.560084138 |
| DMAP1 | 0.00229512 | 0.493786499 | 0.560344491 |
| STAP2 | -0.002570023 | 0.494061804 | 0.56050177 |
| MAIP1 | -0.002794638 | 0.494739042 | 0.56111482 |
| UBA5 | -0.002340348 | 0.495682239 | 0.562029087 |

| | | | |
|---|---|---|---|
| SPPL2A | -0.002920474 | 0.496160577 | 0.562415915 |
| CNOT1 | -0.00272215 | 0.496545286 | 0.562696427 |
| CHCHD7 | -0.002280275 | 0.498438438 | 0.564685713 |
| C6orf62 | 0.003656884 | 0.498623973 | 0.564716483 |
| PHF14 | -0.002183021 | 0.498762677 | 0.564716483 |
| ADD3 | 0.005445348 | 0.498878805 | 0.564716483 |
| SETD2 | 0.00263993 | 0.499114777 | 0.564827652 |
| WASHC4 | 0.002856963 | 0.501796124 | 0.567705328 |
| PNP | -0.004124523 | 0.502204896 | 0.568011055 |
| ANO6 | -0.00261769 | 0.504588305 | 0.57054938 |
| PSMB3 | 0.005152921 | 0.505746724 | 0.571701563 |
| ACOT7 | 0.004802444 | 0.506137897 | 0.571986047 |
| CNOT7 | -0.002994464 | 0.507445961 | 0.573306266 |
| PAPOLA | -0.003804658 | 0.508803469 | 0.574681604 |
| RNF19A | 0.003098699 | 0.509686913 | 0.575520888 |
| PANK3 | 0.002430933 | 0.510414264 | 0.576183503 |
| THOC2 | 0.002558711 | 0.512033227 | 0.57771538 |
| TTC37 | -0.003158109 | 0.51218659 | 0.57771538 |
| PA2G4 | -0.003566874 | 0.512194001 | 0.57771538 |
| INTS11 | -0.002125412 | 0.513468266 | 0.578993369 |
| RIF1 | 0.002231417 | 0.514039294 | 0.579477895 |
| ZMYND11 | -0.002822337 | 0.514491463 | 0.579828202 |
| DPYS | 0.007893016 | 0.514703313 | 0.579839656 |
| KPNA4 | -0.002797069 | 0.514784475 | 0.579839656 |
| RPL7 | -0.008187962 | 0.518430892 | 0.583786502 |
| BCL2L1 | 0.003143238 | 0.519112617 | 0.584393666 |
| TBL1XR1 | 0.003434781 | 0.519917872 | 0.585139521 |
| TXNRD2 | 0.002328655 | 0.522498125 | 0.587882084 |
| MFSD12 | 0.003132047 | 0.52279588 | 0.588055722 |
| SCRN2 | -0.004120994 | 0.523426408 | 0.588603476 |
| ATF4 | 0.003138902 | 0.523807933 | 0.588870996 |
| POLR2A | -0.00224932 | 0.526127656 | 0.591316719 |
| VPS41 | 0.002212775 | 0.526314218 | 0.59136429 |
| ALDH1B1 | -0.003983391 | 0.527200318 | 0.592197617 |
| RANBP2 | -0.00283193 | 0.527470283 | 0.592338581 |
| PCK2 | 0.005823988 | 0.528177267 | 0.592970097 |
| USP10 | -0.002261815 | 0.530580376 | 0.595487259 |
| AMFR | -0.002496084 | 0.530762926 | 0.595487259 |
| CYP27A1 | 0.002852152 | 0.530855105 | 0.595487259 |
| EBLN3P | -0.002276766 | 0.533601834 | 0.598404683 |
| HAT1 | 0.002123028 | 0.533812158 | 0.598476852 |
| RRAS2 | 0.002461487 | 0.534772579 | 0.599389714 |
| RNF212B | -0.004228684 | 0.535219647 | 0.599726852 |
| LHFPL3-AS | 0.002768907 | 0.538123746 | 0.602816219 |
| ILK | -0.002755482 | 0.538561966 | 0.603142327 |
| LONP1 | 0.002507626 | 0.539583909 | 0.604097238 |
| ALDH3A2 | -0.005671094 | 0.539709313 | 0.604097238 |

| | | | |
|---|---|---|---|
| NDUFV1 | 0.005436763 | 0.541135701 | 0.605528486 |
| DHX40 | -0.002422774 | 0.542205601 | 0.606560154 |
| ENY2 | -0.005396612 | 0.542635811 | 0.606875839 |
| PLSCR1 | -0.002106689 | 0.543260461 | 0.607408752 |
| SETX | -0.002379689 | 0.545837693 | 0.610123921 |
| LTA4H | 0.002249433 | 0.54633656 | 0.610515098 |
| PSMC3 | -0.00332398 | 0.548389413 | 0.612642124 |
| WDR33 | -0.002485571 | 0.552020267 | 0.616530399 |
| RBM26 | 0.001999847 | 0.552288401 | 0.616661886 |
| DCAF13 | -0.002496799 | 0.554543662 | 0.619011438 |
| SECISBP2L | -0.002340713 | 0.555321991 | 0.61971153 |
| COLEC11 | 0.002548138 | 0.555537495 | 0.619783327 |
| TNRC6B | 0.003130054 | 0.556629974 | 0.620833214 |
| COQ8A | -0.002171605 | 0.559502923 | 0.623867822 |
| EPC1 | 0.001949394 | 0.559964983 | 0.624150155 |
| STRADB | -0.003034849 | 0.56006059 | 0.624150155 |
| ACAD11 | -0.002884399 | 0.560262794 | 0.62420583 |
| ZFR | -0.002363161 | 0.560517678 | 0.624320152 |
| CAND1 | 0.002498501 | 0.562340176 | 0.62617999 |
| PCCB | 0.002629925 | 0.563907836 | 0.627755125 |
| DCTN1 | 0.001931663 | 0.564719928 | 0.628488519 |
| AL136038. | -0.002397033 | 0.565163661 | 0.628811672 |
| TBRG4 | -0.002089334 | 0.56656026 | 0.630194537 |
| MDP1 | -0.002186158 | 0.568592781 | 0.632283809 |
| VPS51 | 0.002869263 | 0.570612403 | 0.634357606 |
| ASH1L | 0.002956879 | 0.574150679 | 0.638076533 |
| NOP53 | 0.00463729 | 0.574282449 | 0.638076533 |
| PSMD4 | -0.00304876 | 0.574424508 | 0.638076533 |
| ACSF2 | 0.004190629 | 0.575475654 | 0.639052145 |
| FABP3 | -0.004295766 | 0.57561453 | 0.639052145 |
| RAB3GAP1 | 0.001856808 | 0.576138804 | 0.639461044 |
| RAP1A | 0.002423227 | 0.576534862 | 0.639727452 |
| PTPN11 | -0.002093 | 0.578128661 | 0.641322379 |
| STK24 | -0.00218778 | 0.57901043 | 0.642126796 |
| AC018521. | -0.002188367 | 0.579710486 | 0.6424344 |
| USP40 | -0.001917006 | 0.579717823 | 0.6424344 |
| GRAMD1C | 0.001944276 | 0.579757873 | 0.6424344 |
| ITSN2 | 0.001864068 | 0.579994756 | 0.642523237 |
| SVIL | 0.001995417 | 0.580953236 | 0.643411201 |
| LAGE3 | -0.002066865 | 0.581304801 | 0.643626704 |
| PCLO | -0.001996862 | 0.582965362 | 0.645291033 |
| PDPK1 | -0.002081855 | 0.583410349 | 0.645609293 |
| SHOC2 | -0.002181515 | 0.584067197 | 0.646161767 |
| TFDP1 | -0.002268052 | 0.58622542 | 0.648374487 |
| EGFR | -0.002293676 | 0.587459612 | 0.649564296 |
| FMO4 | 0.003101536 | 0.588576925 | 0.650513913 |
| DST | -0.002429332 | 0.58863576 | 0.650513913 |

| | | | |
|---|---|---|---|
| NDUFAF2 | -0.002040515 | 0.590920599 | 0.652862963 |
| MACF1 | -0.002677848 | 0.591942702 | 0.653816024 |
| KDSR | -0.002042753 | 0.592648226 | 0.654418994 |
| STX12 | -0.002045691 | 0.595443296 | 0.657328356 |
| TYMP | -0.002943484 | 0.599094845 | 0.661025448 |
| PIH1D1 | 0.002283841 | 0.599114772 | 0.661025448 |
| FAH | -0.003642625 | 0.600504424 | 0.662380452 |
| ACYP2 | 0.001800682 | 0.600920849 | 0.662661506 |
| UPF1 | -0.002066572 | 0.602320245 | 0.664026083 |
| MPP5 | -0.002184612 | 0.604014919 | 0.665715368 |
| SNRNP70 | -0.002753202 | 0.605280516 | 0.666930964 |
| ASRGL1 | -0.002565029 | 0.607341416 | 0.669021978 |
| CREBL2 | -0.002180438 | 0.608299092 | 0.669778378 |
| DSEL | -0.002027767 | 0.6083548 | 0.669778378 |
| CCDC28A | 0.002621506 | 0.609249259 | 0.670582134 |
| CWF19L2 | 0.001919652 | 0.609411959 | 0.670582134 |
| NUDCD3 | 0.001699439 | 0.611466434 | 0.672662296 |
| TMEM120/ | -0.002222056 | 0.612023331 | 0.673094328 |
| PRKAR2A | 0.002163642 | 0.612732346 | 0.673592291 |
| PCCA | 0.002632277 | 0.612804694 | 0.673592291 |
| ZNF511 | -0.002044838 | 0.613609935 | 0.674296632 |
| PMM1 | 0.002951844 | 0.614218543 | 0.674784573 |
| PARP10 | -0.0017376 | 0.615554636 | 0.676071258 |
| ABCD3 | 0.002856928 | 0.619615429 | 0.680349025 |
| BRD4 | 0.002149276 | 0.620912426 | 0.681590615 |
| STMN1 | 0.002773021 | 0.622698188 | 0.683367926 |
| JAK1 | 0.002906012 | 0.624403901 | 0.685056461 |
| EIF6 | -0.004145728 | 0.626673705 | 0.687362811 |
| DNAJB1 | -0.002288343 | 0.627718323 | 0.688324451 |
| MAPKAPK2 | -0.001899689 | 0.628184751 | 0.688651733 |
| MRPL37 | -0.002270946 | 0.630229229 | 0.690708324 |
| PALM2-AK | 0.001854969 | 0.630675895 | 0.69101314 |
| CUL3 | 0.002165374 | 0.63205217 | 0.692336067 |
| STAU1 | -0.003898755 | 0.634916919 | 0.695288292 |
| HUWE1 | -0.002021421 | 0.638472846 | 0.698995639 |
| PURA | -0.00243809 | 0.639351163 | 0.69977036 |
| FAM151A | 0.005509426 | 0.640199792 | 0.700512182 |
| EPS8 | -0.002907878 | 0.640422605 | 0.700569018 |
| PTBP1 | -0.002191662 | 0.643402452 | 0.703640986 |
| ARFRP1 | -0.001745826 | 0.644394231 | 0.704537692 |
| NSFL1C | -0.001683985 | 0.645372353 | 0.705418995 |
| OXA1L | -0.002038049 | 0.646918419 | 0.706920448 |
| TRIM44 | -0.002280979 | 0.647939812 | 0.707847916 |
| ZC3H7B | -0.001653153 | 0.648918811 | 0.708728589 |
| ATP13A3 | -0.002275772 | 0.649471813 | 0.709143657 |
| EFR3A | -0.001891379 | 0.649841891 | 0.709358827 |
| KLHDC10 | 0.001650702 | 0.652293721 | 0.711845689 |

| | | | |
|---|---|---|---|
| SNHG16 | 0.003190443 | 0.652821862 | 0.712232473 |
| HNRNPUL2 | 0.001737284 | 0.654293437 | 0.713648069 |
| IFT88 | -0.001907841 | 0.654792857 | 0.714002849 |
| ARL8B | -0.001935181 | 0.655592192 | 0.714684389 |
| GNB5 | 0.001899587 | 0.656388245 | 0.715334386 |
| RPA3 | -0.003378901 | 0.656537389 | 0.715334386 |
| PCBP2 | 0.005608129 | 0.660446238 | 0.719402119 |
| PSME1 | -0.003296852 | 0.662176263 | 0.721095001 |
| MPP6 | -0.00154514 | 0.662968142 | 0.721765635 |
| RPL35 | -0.007478593 | 0.663338395 | 0.721817665 |
| UHMK1 | -0.002102795 | 0.66336804 | 0.721817665 |
| NT5DC1 | -0.001938092 | 0.664307717 | 0.722648352 |
| LGALS2 | 0.0063606 | 0.665925373 | 0.724109488 |
| ARSE | 0.002062298 | 0.666116612 | 0.724109488 |
| SRFBP1 | 0.001464478 | 0.666180729 | 0.724109488 |
| UBQLN1 | 0.001702017 | 0.666970623 | 0.724775923 |
| CELF1 | 0.002049776 | 0.667460849 | 0.725116449 |
| GAK | -0.001980133 | 0.668409124 | 0.725954281 |
| UBE2D3 | 0.003236146 | 0.669108274 | 0.726521166 |
| ZNF207 | 0.001932429 | 0.670316088 | 0.727639916 |
| HSP90AA1 | -0.006365071 | 0.672374567 | 0.72968124 |
| YWHAZ | 0.002724169 | 0.673174287 | 0.730355802 |
| MRPL27 | -0.002698507 | 0.674568034 | 0.731674323 |
| MNAT1 | -0.001503334 | 0.675774253 | 0.732788795 |
| C12orf75 | 0.003791359 | 0.676071242 | 0.732917 |
| PHACTR4 | 0.00190834 | 0.678074343 | 0.734894213 |
| PSMB7 | 0.003193195 | 0.679073428 | 0.73578252 |
| YPEL3 | -0.002361251 | 0.67941305 | 0.735956012 |
| AC022613. | -0.001466353 | 0.680123361 | 0.736530845 |
| YWHAG | 0.001808052 | 0.680379093 | 0.736613224 |
| SP100 | -0.001408985 | 0.680752262 | 0.736788332 |
| DMGDH | -0.002651401 | 0.680900242 | 0.736788332 |
| DDX17 | 0.003863593 | 0.681282524 | 0.737007479 |
| TBL1X | 0.001657734 | 0.681931421 | 0.737514858 |
| KCNE3 | 0.001614707 | 0.682496484 | 0.737931325 |
| TUBA4A | 0.001757146 | 0.683956226 | 0.739314666 |
| ATXN2 | 0.00150222 | 0.685367809 | 0.740645234 |
| CCDC6 | -0.002023141 | 0.685943008 | 0.741071498 |
| DDX54 | -0.001405239 | 0.689935999 | 0.74518904 |
| RPLP2 | -0.005695038 | 0.691030035 | 0.746092444 |
| RAF1 | -0.001509914 | 0.691136367 | 0.746092444 |
| INPPL1 | -0.001385243 | 0.692185689 | 0.747028514 |
| RAB6A | 0.00146586 | 0.692422717 | 0.747087668 |
| LUC7L3 | -0.002138096 | 0.69305144 | 0.747352226 |
| EMX1 | -0.001546615 | 0.693159505 | 0.747352226 |
| TMEM37 | -0.002508223 | 0.69321476 | 0.747352226 |
| SNHG9 | 0.002526843 | 0.695850291 | 0.74999637 |

| | | | |
|---|---|---|---|
| PGK1 | 0.003636799 | 0.698079162 | 0.752200937 |
| ERCC1 | 0.001605841 | 0.700488648 | 0.754489798 |
| PCNP | -0.002010953 | 0.70057138 | 0.754489798 |
| CRTAP | -0.001968975 | 0.701135545 | 0.75489909 |
| KRIT1 | 0.001315189 | 0.701838291 | 0.755457336 |
| CNOT6L | -0.00125447 | 0.705280274 | 0.758963025 |
| EFCAB14 | 0.001597183 | 0.705711776 | 0.759228098 |
| PRPSAP2 | 0.00138421 | 0.707379463 | 0.760621092 |
| RCHY1 | -0.001261425 | 0.707391318 | 0.760621092 |
| SNX4 | -0.00208658 | 0.707563132 | 0.760621092 |
| ADGRG6 | -0.001375491 | 0.707769223 | 0.760643202 |
| DUSP3 | 0.001955406 | 0.70850267 | 0.761231904 |
| MRPL55 | 0.002008619 | 0.709089458 | 0.761575115 |
| USP33 | -0.001483779 | 0.709193607 | 0.761575115 |
| CAB39 | -0.001544664 | 0.711071762 | 0.763392046 |
| NME5 | -0.001285132 | 0.711260201 | 0.763394456 |
| EFHD1 | -0.002171571 | 0.713465574 | 0.765561072 |
| TSPAN9 | -0.001302726 | 0.714310018 | 0.766266633 |
| SAFB2 | 0.001592934 | 0.7146815 | 0.766464596 |
| PLIN2 | -0.003206744 | 0.715321841 | 0.766774932 |
| ARF6 | -0.001776943 | 0.715344906 | 0.766774932 |
| NUTM2A-/ | 0.00124721 | 0.716030804 | 0.767309539 |
| RABGAP1 | -0.001239898 | 0.717008785 | 0.768035232 |
| ARHGAP5 | -0.002089422 | 0.717082651 | 0.768035232 |
| ENOSF1 | -0.002209061 | 0.717504544 | 0.768121789 |
| IPO7 | 0.001714758 | 0.717616331 | 0.768121789 |
| AFF1 | 0.002007669 | 0.717725506 | 0.768121789 |
| DDX21 | -0.001398728 | 0.718251337 | 0.76825425 |
| PHACTR2 | 0.001271598 | 0.718285552 | 0.76825425 |
| CTDSP2 | -0.00141658 | 0.718411413 | 0.76825425 |
| GGA2 | 0.001338867 | 0.719093463 | 0.768773567 |
| TRIM33 | 0.001227245 | 0.719272049 | 0.768773567 |
| AMN | 0.003394598 | 0.720866325 | 0.770276761 |
| ETNK1 | 0.001491613 | 0.721823516 | 0.771098597 |
| ZNF33A | -0.001281457 | 0.722253293 | 0.771356734 |
| CISD3 | -0.00286742 | 0.722604393 | 0.771493027 |
| IPO5 | 0.001439051 | 0.722757248 | 0.771493027 |
| C16orf87 | -0.001498493 | 0.726012098 | 0.774692443 |
| UGCG | -0.001726974 | 0.726132453 | 0.774692443 |
| SUSD2 | -0.001527253 | 0.726419055 | 0.774796599 |
| NSA2 | 0.003177045 | 0.730704921 | 0.779165195 |
| BBX | -0.001949946 | 0.733450039 | 0.781889017 |
| CYFIP2 | -0.002004301 | 0.735641082 | 0.784020909 |
| SERPINB6 | 0.00199392 | 0.736023109 | 0.784224207 |
| PNRC1 | 0.002174199 | 0.738024924 | 0.786152816 |
| GNAI3 | -0.001203341 | 0.739979542 | 0.788030162 |
| ATG5 | 0.001080653 | 0.740843213 | 0.788745047 |

| | | | |
|---|---|---|---|
| BCL2L13 | 0.001271703 | 0.741329504 | 0.788944516 |
| ACSS3 | 0.00122772 | 0.741415419 | 0.788944516 |
| BCCIP | -0.001314574 | 0.742349382 | 0.789675527 |
| GPR160 | 0.001863328 | 0.7424876 | 0.789675527 |
| SOD2 | 0.00289395 | 0.742856969 | 0.789863479 |
| ZNF83 | 0.001120894 | 0.744302748 | 0.791195558 |
| DARS | 0.001627517 | 0.745676401 | 0.792450297 |
| PDLIM1 | 0.00119023 | 0.748125737 | 0.794847246 |
| ZNF664 | 0.001146146 | 0.748875595 | 0.795437808 |
| BRMS1 | -0.001098261 | 0.750707353 | 0.797077026 |
| NDUFV3 | -0.002578216 | 0.750807677 | 0.797077026 |
| SNRPC | -0.001465308 | 0.751668022 | 0.797783818 |
| APOOL | 0.00118582 | 0.753514643 | 0.799536759 |
| TRIM28 | 0.001481082 | 0.755509111 | 0.801445629 |
| MED21 | 0.001341035 | 0.755900218 | 0.801653103 |
| ZMPSTE24 | 0.001015659 | 0.758801194 | 0.804521566 |
| DCAF10 | -0.001064715 | 0.759803817 | 0.805376331 |
| TNIP1 | -0.001322822 | 0.760205517 | 0.805593854 |
| ZNF292 | 0.001210506 | 0.760942943 | 0.806166942 |
| GPR137B | 0.001450345 | 0.764336164 | 0.809552641 |
| STARD7 | -0.00160559 | 0.765365208 | 0.810433201 |
| CNOT2 | -0.001024332 | 0.766326655 | 0.811241747 |
| ZER1 | -0.0010602 | 0.76809163 | 0.81290028 |
| MLXIPL | 0.001152009 | 0.773521418 | 0.818435565 |
| ACOT13 | 0.002324985 | 0.774278011 | 0.819024728 |
| KIF12 | -0.001796593 | 0.77509507 | 0.819677531 |
| PTER | 0.001305412 | 0.775963446 | 0.820384252 |
| PSMC4 | -0.001671904 | 0.776188307 | 0.82041043 |
| FDPS | 0.001584477 | 0.776484801 | 0.82051229 |
| AP1B1 | -0.001047169 | 0.77950718 | 0.8234938 |
| MCCC1 | -0.000992984 | 0.783013632 | 0.826855916 |
| PHYHIPL | -0.00147598 | 0.783093054 | 0.826855916 |
| MBP | -0.000911229 | 0.784308559 | 0.827926131 |
| ALCAM | -0.0010317 | 0.785144331 | 0.828595047 |
| PPP2R5E | 0.00105755 | 0.786593377 | 0.829910665 |
| FBXO7 | -0.001571774 | 0.788063913 | 0.831248275 |
| DOK4 | -0.000956071 | 0.788291859 | 0.831274852 |
| RNF115 | -0.000902174 | 0.789154534 | 0.831970581 |
| STAT6 | 0.000945952 | 0.789716648 | 0.832349166 |
| UBE2Q1 | -0.000891625 | 0.789969728 | 0.832401923 |
| AP5M1 | 0.000993998 | 0.791659663 | 0.833777741 |
| AC027644. | -0.001105913 | 0.791713586 | 0.833777741 |
| AUH | -0.00141491 | 0.791885493 | 0.833777741 |
| ZYG11B | 0.00114921 | 0.792551275 | 0.8342645 |
| ZBTB38 | 0.001175052 | 0.793076267 | 0.834602847 |
| GDPD3 | -0.001085183 | 0.796249255 | 0.837726956 |
| NMT1 | -0.001320201 | 0.797144616 | 0.838453804 |

| | | | |
|---|---|---|---|
| API5 | 0.000991173 | 0.798404308 | 0.839459992 |
| DMXL1 | -0.001416319 | 0.798510725 | 0.839459992 |
| SLC22A6 | -0.002468668 | 0.799125263 | 0.839890689 |
| PHIP | -0.000963116 | 0.799599481 | 0.840173725 |
| SEMA3B | -0.001022098 | 0.799920962 | 0.840296168 |
| AP2A2 | 0.001298704 | 0.801742265 | 0.841993669 |
| MIA2 | -0.001717978 | 0.80243698 | 0.842507456 |
| MAN2A1 | 0.00086153 | 0.803562427 | 0.843473105 |
| HNF1B | -0.001270341 | 0.804269181 | 0.843998884 |
| PPHLN1 | -0.000810199 | 0.804999125 | 0.844548724 |
| VPS26A | 0.001044364 | 0.807104952 | 0.846541392 |
| PCNX4 | 0.000783115 | 0.807613405 | 0.846858046 |
| C12orf49 | -0.000999436 | 0.807836703 | 0.846875603 |
| SNTA1 | -0.001133453 | 0.808298443 | 0.847143051 |
| IREB2 | 0.000828819 | 0.808749048 | 0.847398695 |
| PDCD6IP | -0.001292289 | 0.809500772 | 0.847969638 |
| TKFC | 0.001558601 | 0.810232528 | 0.848519378 |
| HOXA-AS2 | -0.000797437 | 0.810453337 | 0.84852053 |
| WDR61 | 0.000954442 | 0.810655007 | 0.84852053 |
| SCO2 | 0.000885084 | 0.811087872 | 0.84852053 |
| ZNHIT1 | -0.002614749 | 0.811098781 | 0.84852053 |
| WNK1 | -0.00094152 | 0.811268409 | 0.84852053 |
| SPEN | 0.000834418 | 0.811646664 | 0.848699649 |
| PRPF31 | 0.000923425 | 0.812204533 | 0.849066442 |
| PRKCE | 0.000943292 | 0.812448205 | 0.849104675 |
| GFPT1 | -0.000839497 | 0.814514525 | 0.851047287 |
| CAPZA2 | 0.001717811 | 0.81505232 | 0.851392232 |
| D2HGDH | 0.001012344 | 0.815868859 | 0.8520281 |
| OGG1 | 0.001094128 | 0.816420851 | 0.852387443 |
| IDH3G | 0.001417184 | 0.816772564 | 0.852537554 |
| LINC01320 | 0.002111646 | 0.817052778 | 0.852612978 |
| AKT2 | -0.000835927 | 0.81849691 | 0.853902629 |
| PCYT2 | -0.001280652 | 0.819309374 | 0.854532799 |
| FMO1 | -0.002001799 | 0.820750534 | 0.855685302 |
| ENTPD3-AS | 0.000970414 | 0.820831779 | 0.855685302 |
| REX1BD | -0.001429682 | 0.822606212 | 0.857294302 |
| BTD | -0.000758216 | 0.822793434 | 0.857294302 |
| ACMSD | -0.00168423 | 0.823176288 | 0.8574753 |
| NELFE | -0.000912579 | 0.825189394 | 0.859353954 |
| ATL2 | 0.000753338 | 0.832527677 | 0.866775896 |
| CACYBP | -0.001185888 | 0.834978196 | 0.869106526 |
| OTUB1 | 0.000827985 | 0.835857514 | 0.869800966 |
| ITFG1 | -0.000845375 | 0.836762575 | 0.870521836 |
| C19orf33 | -0.000978336 | 0.838826349 | 0.872447496 |
| TAOK3 | -0.000709196 | 0.839696112 | 0.873115746 |
| PPP4R2 | 0.000792495 | 0.839908506 | 0.873115746 |
| SPSB3 | -0.000875671 | 0.840107711 | 0.873115746 |

| | | | |
|---|---|---|---|
| RBM23 | -0.000749689 | 0.842875247 | 0.875770024 |
| FMR1 | 0.000923734 | 0.844006734 | 0.876723488 |
| LRRC28 | -0.000748637 | 0.844563835 | 0.87707997 |
| SMIM14 | -0.001007259 | 0.845812359 | 0.878154134 |
| PDZD8 | 0.000856573 | 0.846491062 | 0.878636292 |
| PC | 0.00075196 | 0.84896382 | 0.880979919 |
| PPP6C | -0.000697336 | 0.851629507 | 0.883522515 |
| PIK3C2A | 0.000771655 | 0.852169189 | 0.883858759 |
| AOX1 | 0.00089092 | 0.854004262 | 0.885538056 |
| MAB21L4 | 0.000747921 | 0.854459124 | 0.885785691 |
| VGLL4 | 0.000724753 | 0.855408919 | 0.886541088 |
| GADD45GI | -0.001576442 | 0.855620265 | 0.886541088 |
| IMP3 | -0.001113936 | 0.85832723 | 0.889121183 |
| SELENOO | -0.000640885 | 0.858660465 | 0.889241704 |
| SNX27 | -0.000915418 | 0.859984925 | 0.890306362 |
| OTUD1 | 0.000753673 | 0.860122805 | 0.890306362 |
| MRPL1 | -0.000802028 | 0.860701038 | 0.890680025 |
| AK6 | -0.000887614 | 0.861814021 | 0.891606734 |
| KLF9 | 0.000961756 | 0.862586765 | 0.892181064 |
| SNHG5 | 0.001285442 | 0.86331793 | 0.892712109 |
| ROCK1 | -0.000569579 | 0.863885255 | 0.893073511 |
| ISOC2 | -0.00175234 | 0.864284858 | 0.893261385 |
| SCFD1 | -0.00061817 | 0.865630681 | 0.894426863 |
| RBPMS | 0.001403318 | 0.866831306 | 0.895230411 |
| AGXT2 | 0.001701199 | 0.866845056 | 0.895230411 |
| RUFY3 | -0.000655747 | 0.867244987 | 0.895238883 |
| EAF2 | -0.001040753 | 0.867289962 | 0.895238883 |
| RNF128 | -0.000576618 | 0.867837754 | 0.895578855 |
| TSPAN12 | 0.000818831 | 0.868178788 | 0.895705342 |
| MIER1 | -0.000560821 | 0.871879961 | 0.89929757 |
| MBD3 | -0.000606807 | 0.872224503 | 0.899426676 |
| DCAF11 | 0.000715363 | 0.872925621 | 0.899923321 |
| ZNRD2 | 0.000726636 | 0.873781356 | 0.900579075 |
| ZC3H14 | -0.000604701 | 0.875545176 | 0.902170199 |
| TNRC18 | -0.000558612 | 0.878680488 | 0.905173367 |
| STIP1 | 0.000538991 | 0.87904442 | 0.905320804 |
| FAM50A | -0.000704517 | 0.879398706 | 0.905458235 |
| ARHGAP35 | 0.000615218 | 0.879863979 | 0.905709845 |
| TANK | -0.000483254 | 0.880354915 | 0.905987739 |
| GRTP1 | 0.000529256 | 0.882255983 | 0.907716319 |
| PHF3 | 0.000723924 | 0.884877039 | 0.910184611 |
| KLHDC3 | 0.000557687 | 0.885485966 | 0.910478881 |
| CSNK2B | -0.000975952 | 0.885607263 | 0.910478881 |
| NIT2 | 0.001403141 | 0.886111461 | 0.910544249 |
| RIDA | 0.001940701 | 0.886115013 | 0.910544249 |
| DANCR | -0.000932855 | 0.887040272 | 0.911266629 |
| ATP11A | -0.000479852 | 0.888685959 | 0.912728565 |

| | | | |
|---|---|---|---|
| CERT1 | -0.000556072 | 0.889183655 | 0.913011016 |
| FAM91A1 | 0.000505437 | 0.890472104 | 0.914105064 |
| ABCF1 | -0.00056562 | 0.890865974 | 0.914280473 |
| DHX15 | 0.000512963 | 0.892397628 | 0.915623192 |
| SLAIN2 | -0.000450472 | 0.893264756 | 0.916151233 |
| TRAP1 | -0.000649691 | 0.893359178 | 0.916151233 |
| ACOX2 | -0.000615288 | 0.899736256 | 0.922460277 |
| CBR4 | 0.000551523 | 0.902957062 | 0.925530989 |
| TARDBP | -0.000458594 | 0.903286261 | 0.925637008 |
| RPL22L1 | -0.000557187 | 0.905431428 | 0.927603412 |
| PDE4DIP | -0.000436212 | 0.906880804 | 0.928763103 |
| ANKRD12 | 0.000531547 | 0.907016455 | 0.928763103 |
| SNHG25 | 0.000879196 | 0.907392515 | 0.928916183 |
| GLMP | -0.000417886 | 0.90865166 | 0.929972992 |
| RPL21 | 0.002076735 | 0.909551574 | 0.930661705 |
| RCOR3 | -0.000407578 | 0.910164793 | 0.930837834 |
| MOB1B | 0.000458776 | 0.910177775 | 0.930837834 |
| HIPK2 | 0.000734956 | 0.911076409 | 0.931524508 |
| BTBD7 | -0.000472017 | 0.911743962 | 0.931974631 |
| MRPL21 | -0.00056805 | 0.912742315 | 0.932762585 |
| EIF4EBP3 | -0.000504954 | 0.913097412 | 0.932892945 |
| VIM | -0.001449873 | 0.913726627 | 0.933303232 |
| COMMD1 | -0.000564537 | 0.914227683 | 0.93358244 |
| C22orf39 | 0.000419634 | 0.915729704 | 0.934883413 |
| ADD1 | -0.000603835 | 0.915970851 | 0.934896811 |
| TIA1 | -0.000333436 | 0.91627702 | 0.934976551 |
| NONO | -0.000552274 | 0.919988895 | 0.938251317 |
| ENDOG | 0.000485759 | 0.920085779 | 0.938251317 |
| EBNA1BP2 | 0.000543316 | 0.920172816 | 0.938251317 |
| NDUFA10 | 0.000712392 | 0.921787637 | 0.939664175 |
| GSTA1 | -0.002470017 | 0.922853351 | 0.940365851 |
| PHKB | 0.000389889 | 0.923189789 | 0.940365851 |
| PBLD | -0.001117889 | 0.923386731 | 0.940365851 |
| ZC3H7A | 0.000339905 | 0.923393394 | 0.940365851 |
| HMGCS1 | -0.000315817 | 0.92408148 | 0.940832895 |
| C1GALT1 | 0.000384562 | 0.925001415 | 0.941162805 |
| PSMA4 | 0.000592043 | 0.92507919 | 0.941162805 |
| MFSD3 | 0.00034755 | 0.925094172 | 0.941162805 |
| SHARPIN | 0.00037462 | 0.926333585 | 0.941767492 |
| BAG6 | 0.000427878 | 0.926347194 | 0.941767492 |
| PDXK | 0.000586593 | 0.926377633 | 0.941767492 |
| SMU1 | 0.000325518 | 0.92771101 | 0.942889227 |
| EXOSC4 | -0.000343158 | 0.928012611 | 0.942962009 |
| SAT2 | 0.001258792 | 0.929247864 | 0.943838557 |
| GFM1 | -0.000329777 | 0.929335672 | 0.943838557 |
| KMT2E | -0.000359992 | 0.9299039 | 0.944181771 |
| HPS1 | -0.000302312 | 0.931355674 | 0.945421704 |

| | | | |
|---|---|---|---|
| TMUB1 | 0.000298754 | 0.931906727 | 0.945596534 |
| DIAPH1 | -0.00030256 | 0.931989169 | 0.945596534 |
| NAA60 | 0.000395314 | 0.932562145 | 0.945943789 |
| RSF1 | -0.000322461 | 0.933176051 | 0.946332379 |
| SPG11 | -0.000270745 | 0.935274291 | 0.948225666 |
| CD2AP | 0.000452548 | 0.935725138 | 0.948448224 |
| PRKAA1 | -0.000274093 | 0.937017502 | 0.94952342 |
| MAPKAP1 | 0.000388932 | 0.938234683 | 0.95028827 |
| BIRC2 | 0.000496716 | 0.938235833 | 0.95028827 |
| RNF185 | 0.000269464 | 0.938528255 | 0.950349678 |
| DEK | -0.000412018 | 0.940294535 | 0.951703781 |
| NDFIP2 | -0.000382922 | 0.94032976 | 0.951703781 |
| DDC | 0.000809976 | 0.942799347 | 0.953967749 |
| SMARCA2 | 0.000259571 | 0.943046485 | 0.953982381 |
| RNF11 | 0.000303393 | 0.945993189 | 0.956727202 |
| SPTAN1 | -0.000282952 | 0.947441877 | 0.957956029 |
| FBXO21 | -0.000489271 | 0.948851679 | 0.959144942 |
| RPL18A | 0.001197692 | 0.949334027 | 0.959395985 |
| SETD3 | -0.000379764 | 0.952805885 | 0.962667356 |
| YTHDC1 | -0.000223828 | 0.954083226 | 0.963549714 |
| NUFIP2 | 0.000239053 | 0.954149229 | 0.963549714 |
| IMPDH2 | -0.000192067 | 0.954743363 | 0.963912285 |
| ARPP19 | 0.000292239 | 0.956980121 | 0.965767805 |
| SLC34A3 | 0.00033092 | 0.957082154 | 0.965767805 |
| SLC7A8 | -0.000770123 | 0.957287892 | 0.965767805 |
| C12orf57 | -0.000352423 | 0.957575969 | 0.965820781 |
| PPP1R12A | -0.000178391 | 0.960591509 | 0.968614932 |
| RNH1 | 0.000375104 | 0.960818763 | 0.968614932 |
| AP1M2 | 0.000341033 | 0.963098384 | 0.970674379 |
| FCHO2 | -0.000147555 | 0.965569955 | 0.972926227 |
| PRR34-AS1 | -0.00019736 | 0.968233908 | 0.975370768 |
| RPS26 | 0.000634204 | 0.971191008 | 0.978109343 |
| HSD17B11 | -0.000148144 | 0.971791215 | 0.978473473 |
| H1F0 | -0.000133356 | 0.974283165 | 0.980741708 |
| AMDHD2 | 0.000130016 | 0.975341161 | 0.981565725 |
| RAD23B | -0.000132878 | 0.976426792 | 0.982232125 |
| MAP3K2 | 0.00013879 | 0.976482473 | 0.982232125 |
| ANKRD46 | 9.71817E-05 | 0.978527635 | 0.984047903 |
| ACAP2 | -8.43025E-05 | 0.979737229 | 0.984617711 |
| KIF22 | -0.000158478 | 0.979764414 | 0.984617711 |
| EEF1D | 0.000323925 | 0.979814698 | 0.984617711 |
| NDUFA11 | -0.000266613 | 0.980483465 | 0.985048323 |
| PAIP2 | 0.00017196 | 0.981457442 | 0.985785279 |
| MGMT | 0.000141429 | 0.983655987 | 0.987536139 |
| SBNO1 | -7.30809E-05 | 0.983682339 | 0.987536139 |
| SLC12A6 | 8.27421E-05 | 0.984509482 | 0.988124571 |
| OPA1 | 7.82773E-05 | 0.985500346 | 0.988876999 |

| | | | |
|---|---|---|---|
| MAP7 | 0.000107156 | 0.986733271 | 0.98987189 |
| CHCHD3 | 6.88946E-05 | 0.987287045 | 0.990185148 |
| FLNB | 5.98463E-05 | 0.988286355 | 0.990944988 |
| APOE | 0.000397213 | 0.98863269 | 0.991049885 |
| SCAND1 | 0.000131724 | 0.989138644 | 0.991314701 |
| EML4 | 5.596E-05 | 0.989988535 | 0.991923996 |
| CDC42SE2 | 4.4816E-05 | 0.992148143 | 0.993844951 |
| PLRG1 | -2.83951E-05 | 0.993453056 | 0.99490902 |
| RBPJ | -2.97527E-05 | 0.994274875 | 0.995488886 |
| HSPD1 | 7.52675E-05 | 0.995185727 | 0.996157588 |
| AP1AR | -2.3175E-05 | 0.995696906 | 0.996425998 |
| RNPEP | 1.60777E-05 | 0.996729123 | 0.997215569 |
| ECHDC1 | 1.91231E-05 | 0.997729277 | 0.997972685 |
| POT1-AS1 | -5.70468E-06 | 0.999057894 | 0.999057894 |

**Supplementary Table S3.** Differentially expressed genes Post vs. Pre in the TAL

| gene | logFC_post_pre | P.Value_post_pre | adj.P.Value_post_pre |
|---|---|---|---|
| ERBB4 | 0.712980101 | 1.1361E-211 | 5.727E-208 |
| ESRRG | 0.64091135 | 1.0686E-193 | 2.6933E-190 |
| CCSER1 | 0.512911968 | 7.1851E-187 | 1.2073E-183 |
| PDE4D | 0.330408463 | 2.895E-170 | 3.6484E-167 |
| GPC5 | 0.473598346 | 3.0326E-137 | 3.0575E-134 |
| OXR1 | 0.333473261 | 1.5629E-128 | 1.3131E-125 |
| PTH2R | 0.26889568 | 8.8281E-124 | 6.3575E-121 |
| ADAMTS9-AS2 | 0.249742786 | 7.5078E-123 | 4.7309E-120 |
| NAALADL2 | 0.275858552 | 7.3139E-119 | 4.0966E-116 |
| ZBTB20 | 0.337139044 | 2.1937E-114 | 1.1059E-111 |
| MAML2 | 0.258892488 | 2.4379E-108 | 1.1172E-105 |
| KAZN | 0.223006048 | 9.4063E-102 | 3.9514E-99 |
| IMMP2L | 0.281500886 | 1.1927E-100 | 4.62511E-98 |
| PKHD1 | 0.292149021 | 2.12814E-96 | 7.66282E-94 |
| ARHGAP6 | 0.28266069 | 3.54684E-96 | 1.19198E-93 |
| CACNA2D3 | 0.224329366 | 8.63561E-96 | 2.72076E-93 |
| MECOM | 0.385840379 | 2.45657E-95 | 7.28445E-93 |
| PLCB1 | 0.293091937 | 1.45617E-93 | 4.0781E-91 |
| THRB | 0.205378534 | 6.32199E-90 | 1.67732E-87 |
| PLCL1 | 0.319991561 | 6.91922E-90 | 1.74399E-87 |
| WWOX | 0.215330442 | 2.41032E-87 | 5.78591E-85 |
| RORA | 0.211751398 | 4.06695E-86 | 9.31887E-84 |
| FHIT | 0.222752378 | 2.84239E-85 | 6.22979E-83 |
| BICC1 | 0.250301839 | 1.55754E-84 | 3.27149E-82 |
| NR2F2-AS1 | 0.203761636 | 6.91808E-84 | 1.39496E-81 |
| MALRD1 | 0.102455572 | 6.95135E-79 | 1.34776E-76 |
| MIR99AHG | 0.182361179 | 2.9798E-78 | 5.56339E-76 |
| PDE1A | 0.316721404 | 5.41315E-78 | 9.74559E-76 |
| PRKG1 | 0.150028799 | 2.71277E-74 | 4.71553E-72 |
| LINC01606 | 0.167729855 | 1.89646E-73 | 3.18669E-71 |
| PCDH9 | 0.267734761 | 4.07011E-70 | 6.61853E-68 |
| AC092078.2 | 0.175869378 | 8.91272E-70 | 1.40403E-67 |
| MAGI1 | 0.170250265 | 1.12673E-69 | 1.72116E-67 |
| RBFOX1 | 0.17302363 | 9.8497E-69 | 1.46036E-66 |
| PDE7B | 0.119463102 | 4.30023E-68 | 6.19357E-66 |
| PRKN | 0.155254835 | 1.43656E-67 | 2.01158E-65 |
| TAPT1-AS1 | 0.088932652 | 1.32808E-66 | 1.80943E-64 |
| NR3C2 | 0.17408103 | 4.51864E-66 | 5.99433E-64 |
| LINC01473 | 0.11389118 | 3.64246E-65 | 4.70812E-63 |
| PARD3B | 0.126852318 | 8.26661E-63 | 1.0418E-60 |
| PRKD1 | 0.209870474 | 7.69712E-62 | 9.46371E-60 |
| SOX6 | 0.165878867 | 1.19655E-61 | 1.43614E-59 |
| AC068631.1 | 0.15842288 | 1.77311E-61 | 2.07866E-59 |

| | | | |
|---|---|---|---|
| LRBA | 0.190685456 | 1.11716E-60 | 1.27991E-58 |
| STPG2 | 0.084224125 | 6.22485E-58 | 6.97322E-56 |
| PHACTR1 | 0.177967152 | 1.96884E-57 | 2.15759E-55 |
| DANT2 | 0.099506152 | 3.65052E-56 | 3.91538E-54 |
| AC138305.1 | 0.130192998 | 7.4909E-56 | 7.86701E-54 |
| KLF12 | 0.117736488 | 3.59843E-55 | 3.70198E-53 |
| LRP1B | 0.132157828 | 4.78878E-54 | 4.82804E-52 |
| USP2 | -0.129606949 | 1.47036E-53 | 1.45335E-51 |
| SUPT3H | 0.088738028 | 2.10418E-53 | 2.03984E-51 |
| KIAA1217 | 0.13578451 | 2.22365E-53 | 2.11499E-51 |
| AL159156.1 | 0.117541364 | 3.95941E-53 | 3.69618E-51 |
| CACNB2 | 0.126117955 | 5.47873E-53 | 5.02151E-51 |
| MAST4 | 0.1471925 | 6.48057E-53 | 5.83367E-51 |
| MACROD2 | 0.109092952 | 7.61365E-52 | 6.7334E-50 |
| GLIS3 | 0.118409379 | 4.81451E-51 | 4.18447E-49 |
| PLXDC2 | 0.106394924 | 5.16798E-51 | 4.41556E-49 |
| GPHN | 0.103156903 | 4.1024E-50 | 3.4467E-48 |
| MAPK10 | 0.125210606 | 4.93284E-50 | 4.07646E-48 |
| LINC00472 | 0.237863088 | 8.14519E-50 | 6.62256E-48 |
| FTX | 0.158275605 | 2.4002E-49 | 1.92054E-47 |
| EXOC4 | 0.107833872 | 2.65908E-49 | 2.09444E-47 |
| CLDN10-AS1 | 0.078230562 | 4.78424E-49 | 3.71036E-47 |
| CCDC178 | 0.09396278 | 1.02053E-48 | 7.79472E-47 |
| ARL15 | 0.092994444 | 1.97665E-48 | 1.48721E-46 |
| RERE | 0.146001153 | 2.82847E-46 | 2.09681E-44 |
| PLEKHA5 | 0.125952142 | 3.8908E-46 | 2.84254E-44 |
| LINC01725 | 0.085067275 | 6.12179E-46 | 4.40856E-44 |
| CA10 | 0.09141643 | 1.15524E-45 | 8.20221E-44 |
| LINC01762 | 0.124645433 | 1.28314E-45 | 8.98373E-44 |
| MBD5 | 0.103204037 | 7.69584E-45 | 5.31434E-43 |
| TBC1D5 | 0.114614127 | 5.10534E-44 | 3.47784E-42 |
| BBS9 | 0.079702705 | 1.31774E-43 | 8.85698E-42 |
| TOX | 0.071218345 | 1.73612E-43 | 1.15155E-41 |
| CCDC148 | 0.118527198 | 2.94236E-43 | 1.92629E-41 |
| SMYD3 | 0.087382432 | 2.31339E-42 | 1.4951E-40 |
| PPFIA2 | 0.083248219 | 2.85267E-42 | 1.8203E-40 |
| THSD7A | 0.130998986 | 1.42424E-41 | 8.97448E-40 |
| EHBP1 | 0.081944382 | 3.61653E-41 | 2.25073E-39 |
| RANBP3L | 0.114915045 | 6.60775E-41 | 4.06215E-39 |
| ADK | 0.0949305 | 1.04362E-40 | 6.33841E-39 |
| ZNF385B | 0.10237641 | 4.91773E-40 | 2.95122E-38 |
| AC019197.1 | 0.107636874 | 4.56345E-39 | 2.70639E-37 |
| SBF2 | 0.092481915 | 1.41973E-38 | 8.32192E-37 |
| TCF12 | 0.095470819 | 1.58645E-38 | 9.19231E-37 |
| RAD51B | 0.071117856 | 1.78488E-38 | 1.02245E-36 |
| RABGAP1L | 0.08442121 | 1.98165E-38 | 1.12241E-36 |
| COBLL1 | 0.197036052 | 2.8904E-38 | 1.61894E-36 |

| | | | |
|---|---|---|---|
| MBNL2 | 0.107858965 | 8.84617E-38 | 4.90039E-36 |
| SSBP2 | 0.090247523 | 2.55567E-37 | 1.40034E-35 |
| VPS13B | 0.07835 | 3.3148E-37 | 1.79676E-35 |
| EFNA5 | 0.140391375 | 7.87168E-37 | 4.2214E-35 |
| PARD3 | 0.081227695 | 5.36481E-36 | 2.84674E-34 |
| S100A6 | -0.3954981 | 7.2252E-36 | 3.79398E-34 |
| CRADD | 0.065602697 | 8.98119E-35 | 4.66744E-33 |
| KANSL1L | 0.083139764 | 2.27722E-34 | 1.17138E-32 |
| SGO1-AS1 | 0.081254508 | 3.47324E-34 | 1.76855E-32 |
| PIM3 | -0.086104638 | 4.37307E-34 | 2.20447E-32 |
| EGF | 0.307354578 | 9.57714E-34 | 4.74014E-32 |
| SGIP1 | 0.13783081 | 9.59124E-34 | 4.74014E-32 |
| FAF1 | 0.083274334 | 9.76897E-34 | 4.7811E-32 |
| ZCCHC7 | 0.074943007 | 1.19E-33 | 5.76806E-32 |
| ALDH1A2 | 0.070528568 | 8.66058E-33 | 4.1579E-31 |
| CCNH | 0.077425278 | 1.67499E-32 | 7.96567E-31 |
| CSRNP3 | 0.054922686 | 2.67415E-32 | 1.25985E-30 |
| KCTD16 | 0.061597872 | 3.23709E-32 | 1.51094E-30 |
| VWA8 | 0.087096466 | 6.12531E-32 | 2.83281E-30 |
| SETBP1 | 0.086544217 | 7.4642E-32 | 3.42064E-30 |
| EXT1 | 0.072346465 | 1.40229E-31 | 6.36842E-30 |
| TBCK | 0.068208045 | 1.50102E-31 | 6.73702E-30 |
| SKAP1 | 0.08269441 | 1.51018E-31 | 6.73702E-30 |
| NCOA2 | 0.085839802 | 2.62191E-31 | 1.15939E-29 |
| PTPRG | 0.112034185 | 2.97871E-31 | 1.30571E-29 |
| FREM1 | 0.090117208 | 3.03531E-31 | 1.31905E-29 |
| PELI2 | 0.057954335 | 4.55153E-31 | 1.96105E-29 |
| NEBL | 0.092366948 | 5.17732E-31 | 2.21177E-29 |
| EXOC6B | 0.061031154 | 9.42507E-31 | 3.99258E-29 |
| TMEM232 | 0.060603659 | 1.37519E-30 | 5.77692E-29 |
| MITF | 0.105079486 | 1.55284E-30 | 6.46931E-29 |
| NELL1 | 0.122732189 | 4.96005E-30 | 2.04948E-28 |
| FAM172A | 0.087300753 | 8.01379E-30 | 3.28435E-28 |
| MAGI2 | 0.087754979 | 1.27695E-29 | 5.19122E-28 |
| MPC2 | -0.151043666 | 1.41353E-29 | 5.70048E-28 |
| CDKAL1 | 0.062274828 | 1.50861E-29 | 6.03566E-28 |
| PPP2R2B | 0.064579791 | 1.89584E-29 | 7.52515E-28 |
| SLC3A1 | 0.155349377 | 2.79951E-29 | 1.10253E-27 |
| ARHGEF38 | 0.059426684 | 3.0955E-29 | 1.20965E-27 |
| GMDS-DT | 0.068335455 | 4.32676E-29 | 1.67779E-27 |
| FGD4 | 0.103773968 | 5.9945E-29 | 2.30674E-27 |
| SFRP1 | -0.210145704 | 8.84446E-29 | 3.37765E-27 |
| AC018730.1 | 0.05987864 | 1.11621E-28 | 4.23069E-27 |
| SHROOM3 | 0.077938759 | 1.58811E-28 | 5.97437E-27 |
| CPEB3 | 0.075876994 | 1.70143E-28 | 6.35326E-27 |
| FBXL17 | 0.082815102 | 1.87424E-28 | 6.9471E-27 |
| CAMKMT | 0.066378944 | 2.06295E-28 | 7.58246E-27 |

| | | | |
|---|---|---|---|
| MPPED2 | 0.074183393 | 2.07574E-28 | 7.58246E-27 |
| BTBD9 | 0.064614152 | 2.2847E-28 | 8.28574E-27 |
| SERPINA5 | -0.16288792 | 3.69756E-28 | 1.33139E-26 |
| MYO3B | 0.07243944 | 1.12873E-27 | 4.03541E-26 |
| JMJD1C | 0.102576324 | 1.93991E-27 | 6.88666E-26 |
| PTPRM | 0.066923121 | 2.05787E-27 | 7.25434E-26 |
| SAMD12 | 0.067818352 | 2.24391E-27 | 7.85523E-26 |
| N4BP2L2 | 0.154585218 | 2.96521E-27 | 1.03087E-25 |
| DENND1A | 0.057796673 | 3.00052E-27 | 1.036E-25 |
| PTPN4 | 0.104560904 | 3.91085E-27 | 1.34113E-25 |
| LINC02432 | 0.078358941 | 7.90484E-27 | 2.69245E-25 |
| ANK3 | 0.123686346 | 8.45928E-27 | 2.86196E-25 |
| PKP4 | 0.157977505 | 1.06054E-26 | 3.56414E-25 |
| BCAS3 | 0.062230187 | 1.2562E-26 | 4.19373E-25 |
| TMEM72-AS1 | 0.052003104 | 1.38626E-26 | 4.59746E-25 |
| TEX41 | 0.067256742 | 1.53482E-26 | 5.05687E-25 |
| ENOX1 | 0.094308316 | 2.18011E-26 | 7.1363E-25 |
| LINC02532 | 0.091024203 | 2.37342E-26 | 7.71898E-25 |
| CPEB4 | 0.120958727 | 3.70008E-26 | 1.19565E-24 |
| TRPS1 | 0.072342652 | 4.9904E-26 | 1.60233E-24 |
| PBX1 | 0.113447489 | 5.80814E-26 | 1.85309E-24 |
| SPIDR | 0.056313726 | 6.50992E-26 | 2.06393E-24 |
| NFAT5 | 0.077410916 | 8.62812E-26 | 2.7184E-24 |
| SIK3 | 0.075424178 | 9.00701E-26 | 2.82015E-24 |
| THSD4 | 0.074227636 | 1.09643E-25 | 3.4118E-24 |
| ASB3 | 0.054421857 | 1.27297E-25 | 3.93685E-24 |
| DNAH14 | 0.05724744 | 1.71981E-25 | 5.25533E-24 |
| VAMP2 | -0.111171962 | 1.72015E-25 | 5.25533E-24 |
| GPAT3 | 0.083967246 | 1.87651E-25 | 5.6985E-24 |
| ASXL3 | 0.047186918 | 2.21727E-25 | 6.68383E-24 |
| MYO9A | 0.120354469 | 2.2275E-25 | 6.68383E-24 |
| BAZ2B | 0.083903301 | 2.44252E-25 | 7.28565E-24 |
| ARID1B | 0.102691697 | 2.90397E-25 | 8.61114E-24 |
| INPP4B | 0.105610531 | 3.164E-25 | 9.32731E-24 |
| CASR | 0.133233713 | 3.55558E-25 | 1.04208E-23 |
| FNDC3B | 0.078029524 | 3.60053E-25 | 1.04915E-23 |
| NHS | 0.085584003 | 3.76661E-25 | 1.09124E-23 |
| PTPN13 | 0.101828353 | 3.85787E-25 | 1.11129E-23 |
| KCNMB2 | 0.062836486 | 4.17422E-25 | 1.19558E-23 |
| KDM6A | 0.051672536 | 4.34243E-25 | 1.23673E-23 |
| MED13L | 0.078601323 | 4.47514E-25 | 1.26737E-23 |
| VTI1A | 0.053253694 | 4.9089E-25 | 1.38244E-23 |
| PAN3 | 0.067111021 | 5.22934E-25 | 1.46451E-23 |
| SCGB1D2 | 0.068297434 | 6.61646E-25 | 1.84274E-23 |
| KIAA1328 | 0.047645938 | 1.13811E-24 | 3.15232E-23 |
| PTPRK | 0.052442647 | 1.32218E-24 | 3.64213E-23 |
| APBB2 | 0.066339049 | 1.32979E-24 | 3.6432E-23 |

| | | | |
|---|---|---|---|
| CAMK2N1 | -0.081574627 | 1.38669E-24 | 3.77855E-23 |
| WDFY3 | 0.064710872 | 2.52636E-24 | 6.84698E-23 |
| TCAIM | 0.077935607 | 2.73145E-24 | 7.35967E-23 |
| KCNIP4 | 0.116131378 | 2.74473E-24 | 7.35967E-23 |
| TENT2 | 0.06439025 | 3.58172E-24 | 9.55315E-23 |
| PPM1L | 0.08132742 | 5.82268E-24 | 1.54485E-22 |
| FRAS1 | 0.081994482 | 6.52383E-24 | 1.72181E-22 |
| TNRC6A | 0.094408945 | 9.67899E-24 | 2.54124E-22 |
| KIAA1958 | 0.058255046 | 1.19965E-23 | 3.13338E-22 |
| RBFOX2 | 0.059145169 | 1.39467E-23 | 3.62398E-22 |
| VEGFB | -0.101449086 | 1.42726E-23 | 3.68965E-22 |
| EPB41L4A | 0.054865871 | 1.4583E-23 | 3.75065E-22 |
| ARHGEF28 | 0.071409864 | 4.41188E-23 | 1.12895E-21 |
| KLHL13 | 0.084704565 | 5.12796E-23 | 1.30556E-21 |
| CLASP2 | 0.061763415 | 6.94279E-23 | 1.75872E-21 |
| ITPR2 | 0.113758628 | 8.03637E-23 | 2.02557E-21 |
| PATJ | 0.092595012 | 8.46474E-23 | 2.12292E-21 |
| NOS1AP | 0.048528652 | 1.20984E-22 | 3.0192E-21 |
| NSMCE2 | 0.051792897 | 1.44585E-22 | 3.5904E-21 |
| ATP1B1 | -0.339022184 | 1.918E-22 | 4.73953E-21 |
| BABAM2 | 0.06847996 | 2.08439E-22 | 5.12556E-21 |
| MAPK8 | 0.05383833 | 2.44985E-22 | 5.99499E-21 |
| FAM160A1 | 0.055601315 | 2.53889E-22 | 6.18288E-21 |
| STAG1 | 0.062599829 | 3.39234E-22 | 8.22153E-21 |
| MAP4K3 | 0.062026002 | 4.29967E-22 | 1.03706E-20 |
| AC008014.1 | 0.061394397 | 5.69855E-22 | 1.36792E-20 |
| NPSR1-AS1 | 0.055894877 | 6.90335E-22 | 1.64928E-20 |
| MFSD4B | 0.050926894 | 8.83294E-22 | 2.10032E-20 |
| RFX3 | 0.052131681 | 1.20556E-21 | 2.85316E-20 |
| MBNL1 | 0.078812773 | 1.28254E-21 | 3.02117E-20 |
| CBLB | 0.047157387 | 1.34343E-21 | 3.14987E-20 |
| ANKRD17 | 0.079438528 | 1.63064E-21 | 3.79567E-20 |
| FHOD3 | 0.068226233 | 1.63392E-21 | 3.79567E-20 |
| OSBPL3 | 0.081958298 | 1.72053E-21 | 3.97853E-20 |
| FKBP2 | 0.05535413 | 2.11754E-21 | 4.87421E-20 |
| CYCS | -0.133883843 | 2.65033E-21 | 6.07287E-20 |
| FGF13 | 0.058401712 | 2.79941E-21 | 6.38544E-20 |
| TANC2 | 0.057683491 | 3.61503E-21 | 8.20871E-20 |
| ESRRB | 0.066700456 | 3.67092E-21 | 8.29826E-20 |
| GPC6 | 0.058712672 | 3.70715E-21 | 8.34274E-20 |
| HOXD3 | 0.055480562 | 4.01034E-21 | 8.98495E-20 |
| CPNE8 | 0.048767916 | 5.03705E-21 | 1.12353E-19 |
| NRG1 | 0.049718163 | 5.21084E-21 | 1.15717E-19 |
| STAU2 | 0.05526811 | 5.34984E-21 | 1.18283E-19 |
| LINC02343 | 0.054208289 | 5.69862E-21 | 1.25444E-19 |
| PCCA | 0.089679065 | 6.61751E-21 | 1.45039E-19 |
| NEDD4L | 0.101629784 | 8.06682E-21 | 1.76038E-19 |

| | | | |
|---|---|---|---|
| LRCH1 | 0.059058128 | 2.04265E-20 | 4.43836E-19 |
| SH3RF1 | 0.046826221 | 2.2057E-20 | 4.77208E-19 |
| BCL2 | 0.06690361 | 2.22903E-20 | 4.80193E-19 |
| PLPPR1 | 0.062986745 | 2.61698E-20 | 5.6137E-19 |
| NME7 | 0.04218764 | 3.29404E-20 | 7.03613E-19 |
| BTBD11 | 0.046735104 | 4.61929E-20 | 9.82524E-19 |
| STARD13 | 0.046100954 | 5.62377E-20 | 1.19115E-18 |
| RAP2C-AS1 | 0.046489666 | 5.99257E-20 | 1.26396E-18 |
| KANK1 | 0.065832984 | 6.93597E-20 | 1.45684E-18 |
| CYS1 | -0.123567229 | 8.44888E-20 | 1.76725E-18 |
| FARS2 | 0.043032537 | 8.96459E-20 | 1.86738E-18 |
| RP1 | 0.069782806 | 1.21169E-19 | 2.51159E-18 |
| GALNT18 | 0.074657786 | 1.21569E-19 | 2.51159E-18 |
| DPH6 | 0.061080955 | 1.24706E-19 | 2.56588E-18 |
| DENND4A | 0.041595919 | 1.3766E-19 | 2.8209E-18 |
| DNM3 | 0.046495766 | 1.48982E-19 | 3.04056E-18 |
| ANK2 | 0.09209507 | 1.71482E-19 | 3.48565E-18 |
| GRAMD2B | 0.055385754 | 1.74747E-19 | 3.53776E-18 |
| RASA1 | 0.043370254 | 1.87439E-19 | 3.77951E-18 |
| H3F3A | -0.153968028 | 1.98821E-19 | 3.99306E-18 |
| FOXP1 | 0.081288685 | 2.29119E-19 | 4.58328E-18 |
| BRAF | 0.060292214 | 2.83855E-19 | 5.65578E-18 |
| FAM13A | 0.083704491 | 3.18365E-19 | 6.31841E-18 |
| LYPLAL1 | 0.076337446 | 3.72619E-19 | 7.36617E-18 |
| LINC00645 | 0.073819018 | 4.44229E-19 | 8.7475E-18 |
| CPQ | 0.075473632 | 4.66449E-19 | 9.14931E-18 |
| ZSWIM6 | 0.041504418 | 4.82894E-19 | 9.43514E-18 |
| TMCC1 | 0.04980999 | 5.29421E-19 | 1.03043E-17 |
| CPEB2 | 0.063705888 | 6.50636E-19 | 1.26148E-17 |
| THADA | 0.043640729 | 7.08683E-19 | 1.36876E-17 |
| CLCN5 | 0.141301646 | 7.12058E-19 | 1.37003E-17 |
| SPAG16 | 0.063038395 | 7.53209E-19 | 1.4437E-17 |
| NBEAL1 | 0.074837921 | 8.44146E-19 | 1.61187E-17 |
| HIST1H4C | -0.124233679 | 1.04233E-18 | 1.98279E-17 |
| AF117829.1 | 0.043422057 | 1.17691E-18 | 2.23037E-17 |
| PHF21A | 0.04082867 | 1.2423E-18 | 2.34548E-17 |
| PLEKHA7 | 0.051941501 | 1.37982E-18 | 2.5954E-17 |
| SLC16A12 | 0.118838264 | 1.39273E-18 | 2.60995E-17 |
| LAMA1 | 0.051288045 | 1.53387E-18 | 2.8638E-17 |
| USP25 | 0.062780498 | 1.67583E-18 | 3.1173E-17 |
| DCAF6 | 0.045026261 | 2.08714E-18 | 3.86811E-17 |
| XPR1 | 0.046993853 | 3.55824E-18 | 6.57036E-17 |
| DYM | 0.046787034 | 3.82287E-18 | 7.03325E-17 |
| CLASP1 | 0.062444556 | 3.84323E-18 | 7.04499E-17 |
| AIF1L | -0.096212486 | 3.94829E-18 | 7.21135E-17 |
| WDR70 | 0.043916918 | 4.61076E-18 | 8.39093E-17 |
| ZFAND3 | 0.071740717 | 5.28153E-18 | 9.57705E-17 |

| | | | |
|---|---|---|---|
| CD24 | -0.194353972 | 5.4915E-18 | 9.9221E-17 |
| LAMTOR1 | -0.070523144 | 5.80229E-18 | 1.04462E-16 |
| CCDC91 | 0.048619386 | 6.87097E-18 | 1.23262E-16 |
| CHD9 | 0.093519261 | 7.38081E-18 | 1.31938E-16 |
| MAGI3 | 0.048661963 | 9.07243E-18 | 1.61605E-16 |
| FOCAD | 0.039553565 | 9.45389E-18 | 1.67807E-16 |
| CDK6 | 0.052857915 | 9.83222E-18 | 1.73909E-16 |
| SCAPER | 0.048809261 | 1.24686E-17 | 2.1977E-16 |
| METTL15 | 0.047382554 | 1.3597E-17 | 2.38824E-16 |
| FER | 0.042464979 | 1.39133E-17 | 2.43532E-16 |
| UMAD1 | 0.047490657 | 1.41941E-17 | 2.47587E-16 |
| RICTOR | 0.048908329 | 1.50098E-17 | 2.60912E-16 |
| LINC01320 | 0.085457169 | 1.62694E-17 | 2.81836E-16 |
| VEPH1 | 0.058083023 | 1.77473E-17 | 3.06384E-16 |
| ATP5MC1 | -0.150344372 | 2.01165E-17 | 3.461E-16 |
| PTGER3 | 0.152553236 | 2.6629E-17 | 4.56588E-16 |
| TSC22D3 | -0.06487226 | 3.08961E-17 | 5.27957E-16 |
| KNG1 | 0.250471009 | 3.25999E-17 | 5.5519E-16 |
| DMGDH | 0.045915475 | 3.51212E-17 | 5.96115E-16 |
| RNF111 | 0.036230721 | 3.92181E-17 | 6.63417E-16 |
| ARHGAP24 | 0.121527225 | 4.70118E-17 | 7.92596E-16 |
| AGAP1 | 0.055923606 | 4.98058E-17 | 8.36903E-16 |
| ZNF638 | 0.074532085 | 5.15563E-17 | 8.6344E-16 |
| TPST1 | 0.05155139 | 5.65658E-17 | 9.442E-16 |
| ZNF385D | 0.047561752 | 5.75273E-17 | 9.5708E-16 |
| LIPH | 0.041638108 | 6.0662E-17 | 1.00591E-15 |
| BRWD1 | 0.062658343 | 6.72271E-17 | 1.11112E-15 |
| UBA6-AS1 | 0.047692912 | 6.9462E-17 | 1.14431E-15 |
| SHANK2 | 0.054640764 | 8.17832E-17 | 1.3429E-15 |
| PRKCQ | 0.046266884 | 8.44793E-17 | 1.38266E-15 |
| LCORL | 0.040873162 | 8.54165E-17 | 1.39348E-15 |
| COMMD10 | 0.051216428 | 9.73132E-17 | 1.58244E-15 |
| WDR7 | 0.036117062 | 1.01915E-16 | 1.65194E-15 |
| EIF4G2 | -0.089237875 | 1.10197E-16 | 1.78046E-15 |
| PRRG1 | 0.0419996 | 1.11194E-16 | 1.79084E-15 |
| COL4A3 | 0.082740719 | 1.54424E-16 | 2.47914E-15 |
| TRIO | 0.041469579 | 1.68102E-16 | 2.69016E-15 |
| KDM4C | 0.039220861 | 1.79004E-16 | 2.85556E-15 |
| RASAL2 | 0.04997464 | 1.9195E-16 | 3.05244E-15 |
| HS6ST2 | 0.08261198 | 2.20497E-16 | 3.49537E-15 |
| ATP6V1B1 | -0.054399557 | 2.25893E-16 | 3.56967E-15 |
| CADPS2 | 0.054355523 | 2.73235E-16 | 4.3043E-15 |
| ZNF407 | 0.039355868 | 2.84844E-16 | 4.47321E-15 |
| TSPAN8 | 0.103502517 | 3.34544E-16 | 5.23738E-15 |
| COG5 | 0.0371845 | 3.53854E-16 | 5.52253E-15 |
| MEF2C | 0.05277736 | 3.63008E-16 | 5.64792E-15 |
| CCDC171 | 0.037509045 | 3.7305E-16 | 5.78629E-15 |

| | | | |
|---|---|---|---|
| NCOA1 | 0.066898825 | 3.91581E-16 | 6.05509E-15 |
| MON2 | 0.052857418 | 4.18043E-16 | 6.44451E-15 |
| PUM2 | 0.066103584 | 4.37166E-16 | 6.71877E-15 |
| MIR3936HG | 0.043300832 | 4.55907E-16 | 6.98549E-15 |
| MRTFB | 0.044906592 | 5.12067E-16 | 7.82221E-15 |
| PROX1 | 0.063596034 | 5.47916E-16 | 8.34455E-15 |
| TRMT11 | 0.047329314 | 7.00989E-16 | 1.06436E-14 |
| ACSS3 | 0.049034029 | 7.95571E-16 | 1.20435E-14 |
| TNKS | 0.043982541 | 8.74092E-16 | 1.31925E-14 |
| SYNE1 | 0.06713401 | 8.92508E-16 | 1.34302E-14 |
| JUND | -0.07306423 | 9.39661E-16 | 1.40977E-14 |
| GPATCH8 | 0.042644037 | 1.01485E-15 | 1.51805E-14 |
| AKAP6 | 0.036749394 | 1.03328E-15 | 1.54106E-14 |
| CDK14 | 0.039404346 | 1.04428E-15 | 1.55286E-14 |
| DAPK1 | 0.055857954 | 1.15597E-15 | 1.7139E-14 |
| COP1 | 0.037718465 | 1.49063E-15 | 2.2036E-14 |
| KCTD1 | 0.069897688 | 1.7573E-15 | 2.59022E-14 |
| ADGRG1 | -0.078295072 | 1.77647E-15 | 2.61084E-14 |
| PPP1R14B | -0.069043831 | 1.99041E-15 | 2.91676E-14 |
| WDR72 | 0.061910099 | 2.44017E-15 | 3.56548E-14 |
| SCAF8 | 0.044663261 | 2.47406E-15 | 3.60454E-14 |
| BMPR1A | 0.038338943 | 2.99675E-15 | 4.35349E-14 |
| PPP2R3A | 0.071204681 | 3.82507E-15 | 5.54086E-14 |
| CADM1 | 0.077471387 | 3.96199E-15 | 5.72275E-14 |
| ARF1 | -0.079803425 | 4.16865E-15 | 6.00405E-14 |
| CFAP221 | 0.041638503 | 4.44698E-15 | 6.38667E-14 |
| PCNX1 | 0.048449038 | 4.53696E-15 | 6.49739E-14 |
| RAPGEF2 | 0.038957595 | 5.7798E-15 | 8.25382E-14 |
| ATXN1 | 0.055401379 | 5.88514E-15 | 8.38051E-14 |
| MLLT10 | 0.049341457 | 6.35987E-15 | 9.03101E-14 |
| STXBP4 | 0.062094741 | 6.96424E-15 | 9.86144E-14 |
| DOP1A | 0.036494601 | 7.66153E-15 | 1.08184E-13 |
| ADGRF1 | 0.040738891 | 7.78535E-15 | 1.09626E-13 |
| SOS1 | 0.046833087 | 8.54886E-15 | 1.20041E-13 |
| TACC1 | 0.092255335 | 8.96336E-15 | 1.25512E-13 |
| ITPR1 | 0.071988392 | 1.0342E-14 | 1.44416E-13 |
| RALGAPA1 | 0.05591883 | 1.06172E-14 | 1.47849E-13 |
| SNX29 | 0.058467721 | 1.06641E-14 | 1.48094E-13 |
| SSH2 | 0.036278901 | 1.38799E-14 | 1.92221E-13 |
| AOPEP | 0.065248229 | 1.47115E-14 | 2.0318E-13 |
| CCDC18-AS1 | 0.052339726 | 1.62941E-14 | 2.24422E-13 |
| MLLT3 | 0.046798331 | 1.67564E-14 | 2.3016E-13 |
| PGAM1 | -0.067765985 | 1.73445E-14 | 2.37591E-13 |
| PTBP2 | 0.040025047 | 1.75254E-14 | 2.39419E-13 |
| PTK2 | 0.058387769 | 1.92376E-14 | 2.62099E-13 |
| PRDM16-DT | -0.111954963 | 2.00992E-14 | 2.731E-13 |
| PRICKLE2 | 0.041267629 | 2.09835E-14 | 2.84349E-13 |

| | | | |
|---|---|---|---|
| SENP6 | 0.065940952 | 2.27806E-14 | 3.07874E-13 |
| BCKDHB | 0.052419153 | 2.77652E-14 | 3.74236E-13 |
| CASC15 | 0.03886176 | 3.02234E-14 | 4.06283E-13 |
| CTNND1 | 0.06621592 | 3.07559E-14 | 4.12342E-13 |
| ATG10 | 0.037903402 | 3.62444E-14 | 4.84636E-13 |
| ATAD2B | 0.032831502 | 4.04766E-14 | 5.39795E-13 |
| CFAP70 | 0.045864711 | 4.21948E-14 | 5.61224E-13 |
| ADGRL2 | 0.040211752 | 4.35386E-14 | 5.77574E-13 |
| LINC01278 | 0.041087513 | 4.52636E-14 | 5.98882E-13 |
| AKT3 | 0.040563004 | 5.16115E-14 | 6.81083E-13 |
| ACTG1 | -0.132433401 | 6.42133E-14 | 8.45167E-13 |
| ERC1 | 0.039181187 | 6.48266E-14 | 8.51017E-13 |
| CARMIL1 | 0.054325454 | 6.98615E-14 | 9.14732E-13 |
| CTSD | -0.142659904 | 7.28095E-14 | 9.50862E-13 |
| NF1 | 0.052562076 | 9.39282E-14 | 1.22349E-12 |
| PTEN | 0.055451679 | 1.03936E-13 | 1.34887E-12 |
| GRAMD1C | 0.05101031 | 1.04088E-13 | 1.34887E-12 |
| PPP1R9A | 0.044567363 | 1.08252E-13 | 1.39923E-12 |
| BIRC6 | 0.06239161 | 1.18873E-13 | 1.53258E-12 |
| PDSS2 | 0.036077254 | 1.20645E-13 | 1.55146E-12 |
| COL4A5 | 0.035823965 | 1.20987E-13 | 1.5519E-12 |
| TRHDE | 0.043212033 | 1.26287E-13 | 1.61577E-12 |
| TTC17 | 0.051597567 | 1.34197E-13 | 1.71263E-12 |
| ATP5MC3 | -0.160166685 | 1.34548E-13 | 1.71277E-12 |
| FKBP5 | -0.046868556 | 1.42856E-13 | 1.81394E-12 |
| ANGPTL1 | 0.051170043 | 1.59765E-13 | 2.02355E-12 |
| FGFR2 | 0.043633745 | 1.73398E-13 | 2.19072E-12 |
| ACTB | -0.140338044 | 1.83712E-13 | 2.31523E-12 |
| UGT8 | 0.077899806 | 1.84235E-13 | 2.31603E-12 |
| FAM135A | 0.037945165 | 1.91706E-13 | 2.40396E-12 |
| GUCY1A1 | 0.050909512 | 1.92351E-13 | 2.40606E-12 |
| AEBP2 | 0.04618523 | 2.15669E-13 | 2.69106E-12 |
| ZNF618 | 0.05081591 | 2.37223E-13 | 2.95269E-12 |
| MRPL34 | -0.078139837 | 2.56859E-13 | 3.18923E-12 |
| F11 | 0.040792414 | 2.87967E-13 | 3.56669E-12 |
| USP24 | 0.056467089 | 3.49142E-13 | 4.31378E-12 |
| ANO10 | 0.048026271 | 3.53606E-13 | 4.35825E-12 |
| ZCRB1 | -0.048840693 | 3.66603E-13 | 4.50743E-12 |
| ARHGDIA | -0.058556741 | 5.15111E-13 | 6.31794E-12 |
| LRRFIP2 | 0.037233056 | 5.283E-13 | 6.46399E-12 |
| FMO4 | 0.036620744 | 5.34602E-13 | 6.52525E-12 |
| DENND4C | 0.03663921 | 5.4695E-13 | 6.65985E-12 |
| SLC25A12 | 0.04727134 | 5.70875E-13 | 6.93441E-12 |
| YWHAB | -0.079762885 | 6.43397E-13 | 7.79655E-12 |
| FRYL | 0.041774854 | 6.58095E-13 | 7.95553E-12 |
| GULP1 | 0.04035172 | 6.68653E-13 | 8.06383E-12 |
| GAPDH | -0.143352621 | 6.96656E-13 | 8.38149E-12 |

| | | | |
|---|---|---|---|
| TLE5 | -0.082354729 | 7.09755E-13 | 8.51875E-12 |
| NLK | 0.036195509 | 7.49371E-13 | 8.97287E-12 |
| PIBF1 | 0.038829871 | 7.73165E-13 | 9.23583E-12 |
| GRHL2 | 0.032882767 | 7.81258E-13 | 9.31045E-12 |
| EPC2 | 0.031152374 | 9.10731E-13 | 1.08278E-11 |
| VPS54 | 0.032130972 | 9.35594E-13 | 1.10973E-11 |
| SNHG25 | 0.083028681 | 9.67108E-13 | 1.14441E-11 |
| PCLO | 0.048531519 | 9.72222E-13 | 1.14777E-11 |
| MEF2A | 0.057591393 | 1.10037E-12 | 1.29602E-11 |
| ADAMTS9-AS1 | 0.052521704 | 1.14037E-12 | 1.33958E-11 |
| ZMYM2 | 0.056521857 | 1.14267E-12 | 1.33958E-11 |
| REV3L | 0.042089758 | 1.16761E-12 | 1.36564E-11 |
| NR2F1-AS1 | 0.033833209 | 1.29766E-12 | 1.51423E-11 |
| ARIH1 | 0.043176086 | 1.3416E-12 | 1.56189E-11 |
| PXDNL | 0.041786942 | 1.34587E-12 | 1.56325E-11 |
| TLN2 | 0.043075583 | 1.47387E-12 | 1.70799E-11 |
| PPIC | -0.041686204 | 1.48449E-12 | 1.71636E-11 |
| MAP4K3-DT | 0.043131387 | 1.55603E-12 | 1.79495E-11 |
| PPP6R3 | 0.050665339 | 1.6087E-12 | 1.85148E-11 |
| SEC61B | -0.083766778 | 1.63564E-12 | 1.87819E-11 |
| CCDC146 | 0.03376834 | 1.653E-12 | 1.89381E-11 |
| NMD3 | 0.038147991 | 1.66087E-12 | 1.89851E-11 |
| UQCRFS1 | -0.097024306 | 1.6757E-12 | 1.91113E-11 |
| ERBIN | 0.054212173 | 1.74204E-12 | 1.9823E-11 |
| PTMA | -0.161229224 | 1.75429E-12 | 1.99175E-11 |
| C2CD5 | 0.048165901 | 1.87672E-12 | 2.12596E-11 |
| SLC2A13 | 0.047245841 | 1.94387E-12 | 2.1971E-11 |
| EEF1A1 | -0.239973541 | 1.99641E-12 | 2.25143E-11 |
| SLC44A3 | 0.036715458 | 2.08546E-12 | 2.34661E-11 |
| AC016705.2 | 0.035638969 | 2.10434E-12 | 2.36258E-11 |
| CACNB4 | 0.050201365 | 2.40103E-12 | 2.68968E-11 |
| DENND1B | 0.045299572 | 2.41794E-12 | 2.70263E-11 |
| EPB41L5 | 0.044207355 | 2.55686E-12 | 2.85157E-11 |
| SUCLG2-AS1 | 0.037952758 | 2.74173E-12 | 3.051E-11 |
| UGGT2 | 0.036310605 | 2.77096E-12 | 3.07674E-11 |
| CA2 | -0.060349093 | 3.05456E-12 | 3.38419E-11 |
| CNKSR3 | 0.033011648 | 3.10261E-12 | 3.42988E-11 |
| SIM1 | 0.058585489 | 3.27707E-12 | 3.61482E-11 |
| ISCU | -0.085312666 | 3.46052E-12 | 3.80884E-11 |
| LIFR | 0.062162218 | 3.67915E-12 | 4.03417E-11 |
| CBFA2T2 | 0.035348922 | 3.68125E-12 | 4.03417E-11 |
| TET2 | 0.036021244 | 4.13167E-12 | 4.51794E-11 |
| TRAPPC9 | 0.031206334 | 5.20685E-12 | 5.68132E-11 |
| GNAQ | 0.047573479 | 5.37339E-12 | 5.85038E-11 |
| USP15 | 0.038797233 | 5.52783E-12 | 6.00556E-11 |
| ELF2 | 0.039730736 | 5.66438E-12 | 6.14068E-11 |
| PPARGC1A | 0.083503521 | 5.77529E-12 | 6.24747E-11 |

| | | | |
|---|---|---|---|
| KANSL1 | 0.053621444 | 6.31642E-12 | 6.81822E-11 |
| TRIM8 | -0.045160797 | 6.3909E-12 | 6.88388E-11 |
| TMEM213 | -0.088383247 | 6.91026E-12 | 7.42742E-11 |
| SGMS1 | 0.046138351 | 6.99573E-12 | 7.5033E-11 |
| PGRMC1 | -0.06866889 | 7.04829E-12 | 7.54361E-11 |
| YAP1 | 0.043235272 | 7.29149E-12 | 7.78738E-11 |
| YBX1 | -0.108823757 | 8.3311E-12 | 8.87887E-11 |
| VAV3 | 0.076121306 | 8.89269E-12 | 9.45739E-11 |
| PIAS1 | 0.039259605 | 9.34628E-12 | 9.91886E-11 |
| SDC1 | -0.076962071 | 9.53594E-12 | 1.00989E-10 |
| DMXL1 | 0.04172801 | 9.65025E-12 | 1.01985E-10 |
| TNRC6B | 0.071486504 | 9.75277E-12 | 1.02853E-10 |
| PRR15L | -0.040968543 | 1.01816E-11 | 1.07151E-10 |
| HERC4 | 0.04041201 | 1.02182E-11 | 1.07313E-10 |
| TDRD3 | 0.044616645 | 1.07612E-11 | 1.1278E-10 |
| C20orf194 | 0.039017824 | 1.14534E-11 | 1.19785E-10 |
| BSND | -0.03867792 | 1.16273E-11 | 1.21352E-10 |
| PTMS | -0.074251376 | 1.26759E-11 | 1.32023E-10 |
| UQCRQ | -0.119971695 | 1.27083E-11 | 1.32087E-10 |
| UBR3 | 0.044394872 | 1.29148E-11 | 1.33958E-10 |
| GLRX5 | -0.075242103 | 1.39012E-11 | 1.43893E-10 |
| MAN1A1 | 0.075521646 | 1.4899E-11 | 1.53906E-10 |
| MST1 | 0.045531541 | 1.565E-11 | 1.61333E-10 |
| EIF4G3 | 0.037641121 | 1.66694E-11 | 1.71491E-10 |
| AP3B1 | 0.049926267 | 1.89178E-11 | 1.94225E-10 |
| RALGAPA2 | 0.035072141 | 1.97988E-11 | 2.02857E-10 |
| RELCH | 0.032012268 | 2.12155E-11 | 2.16931E-10 |
| PPA2 | 0.042315878 | 2.20708E-11 | 2.2522E-10 |
| IER3 | -0.0506817 | 2.35792E-11 | 2.40127E-10 |
| CHL1 | 0.058523223 | 2.38593E-11 | 2.42489E-10 |
| KMT2C | 0.052608095 | 2.41166E-11 | 2.44378E-10 |
| KIF1C | -0.040758227 | 2.41421E-11 | 2.44378E-10 |
| RBPMS | 0.049921251 | 2.46464E-11 | 2.48983E-10 |
| NRK | 0.064027795 | 2.66654E-11 | 2.68841E-10 |
| NFYC | 0.043083553 | 2.7336E-11 | 2.75051E-10 |
| UQCC2 | -0.061144835 | 3.03073E-11 | 3.04341E-10 |
| PSME4 | 0.03422299 | 3.31846E-11 | 3.32098E-10 |
| TOB1 | -0.055606301 | 3.32032E-11 | 3.32098E-10 |
| ZDHHC17 | 0.033091294 | 3.43637E-11 | 3.43025E-10 |
| RPS27 | 0.171222312 | 3.50392E-11 | 3.49076E-10 |
| SEC24B | 0.031687485 | 3.61783E-11 | 3.59714E-10 |
| PDGFC | 0.031283437 | 3.78318E-11 | 3.75413E-10 |
| PPP1R12A | 0.040222479 | 3.82391E-11 | 3.7871E-10 |
| BEX3 | -0.109827914 | 4.0531E-11 | 4.00621E-10 |
| MICOS10 | -0.105364225 | 4.10773E-11 | 4.05227E-10 |
| RAPGEF6 | 0.031400267 | 4.3213E-11 | 4.25462E-10 |
| MAP3K1 | 0.039521351 | 4.45312E-11 | 4.37586E-10 |

| | | | |
|---|---|---|---|
| UBR2 | 0.043997313 | 4.46285E-11 | 4.37689E-10 |
| CALM1 | -0.075417021 | 4.97203E-11 | 4.8668E-10 |
| SETD5 | 0.037861411 | 5.13535E-11 | 5.01692E-10 |
| TMEM131 | 0.041739597 | 5.15169E-11 | 5.0218E-10 |
| NIPBL | 0.052772577 | 5.16028E-11 | 5.0218E-10 |
| DYNC2H1 | 0.040485567 | 5.70022E-11 | 5.53657E-10 |
| FARP1 | 0.079608551 | 6.05791E-11 | 5.87268E-10 |
| RNF7 | -0.056511829 | 6.37223E-11 | 6.16553E-10 |
| PFN2 | -0.049761407 | 7.02711E-11 | 6.78614E-10 |
| MNAT1 | 0.0426588 | 7.11781E-11 | 6.86059E-10 |
| SLC20A2 | 0.045331325 | 8.81487E-11 | 8.48011E-10 |
| CRIM1 | 0.065286735 | 8.86914E-11 | 8.51606E-10 |
| CDV3 | -0.036483251 | 9.04382E-11 | 8.66728E-10 |
| ATG2B | 0.035438721 | 9.32078E-11 | 8.90423E-10 |
| FTO | 0.031502359 | 9.32639E-11 | 8.90423E-10 |
| SPP1 | -0.145951434 | 1.04284E-10 | 9.93755E-10 |
| RPS21 | 0.152077172 | 1.07963E-10 | 1.02687E-09 |
| CWC27 | 0.029760703 | 1.13604E-10 | 1.07848E-09 |
| RNF150 | 0.054091505 | 1.19567E-10 | 1.13297E-09 |
| IGFBP2 | -0.061788894 | 1.34342E-10 | 1.27058E-09 |
| FREM2 | 0.031979698 | 1.36632E-10 | 1.28981E-09 |
| TSPYL1 | -0.055681453 | 1.38894E-10 | 1.30872E-09 |
| ARID2 | 0.035946709 | 1.46525E-10 | 1.37804E-09 |
| ZNF292 | 0.053459852 | 1.50338E-10 | 1.41128E-09 |
| AKAP13 | 0.038632852 | 1.60208E-10 | 1.50089E-09 |
| KCNJ3 | 0.033716828 | 1.6048E-10 | 1.50089E-09 |
| TUBA1B | -0.049834863 | 1.67182E-10 | 1.56067E-09 |
| STON2 | 0.034024549 | 1.69502E-10 | 1.57941E-09 |
| SORBS1 | 0.033475755 | 1.78687E-10 | 1.65892E-09 |
| MKNK2 | -0.033256869 | 1.78693E-10 | 1.65892E-09 |
| TMEM65 | 0.030490783 | 1.8879E-10 | 1.74943E-09 |
| MKLN1 | 0.051372805 | 1.94873E-10 | 1.80249E-09 |
| HDAC8 | 0.030239384 | 2.00947E-10 | 1.85526E-09 |
| DLG1 | 0.036906348 | 2.1076E-10 | 1.9423E-09 |
| LPP | 0.053740507 | 2.21241E-10 | 2.03517E-09 |
| LIMCH1 | 0.046437011 | 2.42753E-10 | 2.229E-09 |
| ADGRA3 | 0.032123543 | 2.44682E-10 | 2.24262E-09 |
| SORBS2 | 0.037214389 | 2.51311E-10 | 2.2992E-09 |
| FRK | 0.034926298 | 2.57434E-10 | 2.34986E-09 |
| GAREM1 | 0.028682923 | 2.5778E-10 | 2.34986E-09 |
| CKB | 0.138633467 | 2.63436E-10 | 2.39708E-09 |
| AFF1 | 0.04037062 | 2.73464E-10 | 2.48384E-09 |
| UBE2M | -0.045533711 | 2.75643E-10 | 2.49913E-09 |
| PHIP | 0.043886395 | 2.92227E-10 | 2.64473E-09 |
| PFDN2 | -0.04986171 | 3.00675E-10 | 2.71631E-09 |
| AUTS2 | 0.044043808 | 3.2501E-10 | 2.9309E-09 |
| TBC1D4 | 0.064247806 | 3.32518E-10 | 2.99326E-09 |

| | | | |
|---|---|---|---|
| ITGA2 | 0.051754421 | 3.38704E-10 | 3.04351E-09 |
| CHP1 | -0.05188801 | 3.53978E-10 | 3.1751E-09 |
| ATP6V1G1 | -0.078568566 | 3.72686E-10 | 3.33696E-09 |
| NEK1 | 0.032238048 | 3.88611E-10 | 3.47338E-09 |
| IGF1R | 0.06041837 | 3.90086E-10 | 3.4804E-09 |
| RBMS1 | 0.048043432 | 3.92481E-10 | 3.49557E-09 |
| DYRK1A | 0.043190753 | 4.04925E-10 | 3.60004E-09 |
| SUMF1 | 0.037272481 | 4.17093E-10 | 3.70126E-09 |
| ZNF518A | 0.047205265 | 4.17778E-10 | 3.70126E-09 |
| PPP3CA | 0.038830238 | 4.2195E-10 | 3.73166E-09 |
| LDHA | -0.058716243 | 4.34072E-10 | 3.83215E-09 |
| HIP1 | 0.061953198 | 4.38237E-10 | 3.86216E-09 |
| DDX5 | -0.082537423 | 4.75497E-10 | 4.18322E-09 |
| STK3 | 0.031032573 | 5.37548E-10 | 4.72087E-09 |
| NBAS | 0.03226685 | 5.42935E-10 | 4.75989E-09 |
| PDE10A | 0.0331146 | 5.48886E-10 | 4.8037E-09 |
| ARMH4 | 0.028915073 | 5.78867E-10 | 5.05731E-09 |
| NUBPL | 0.030457221 | 5.87191E-10 | 5.12116E-09 |
| RWDD1 | -0.0443598 | 5.99526E-10 | 5.21971E-09 |
| ATP9B | 0.030120105 | 7.02608E-10 | 6.0987E-09 |
| VDAC1 | -0.072800017 | 7.02906E-10 | 6.0987E-09 |
| SLC5A3 | 0.119408852 | 7.09773E-10 | 6.1477E-09 |
| TLK1 | 0.044219817 | 7.31919E-10 | 6.32865E-09 |
| PIK3R1 | -0.06228781 | 7.7283E-10 | 6.67095E-09 |
| VPS50 | 0.027383139 | 8.03845E-10 | 6.92681E-09 |
| LRP6 | 0.048739636 | 8.59338E-10 | 7.39236E-09 |
| H3F3B | -0.089460625 | 8.73095E-10 | 7.49791E-09 |
| GHR | 0.032343766 | 9.08005E-10 | 7.78444E-09 |
| SIL1 | 0.046856479 | 9.14307E-10 | 7.82516E-09 |
| PTPRJ | 0.027018357 | 9.18962E-10 | 7.85167E-09 |
| SRPK2 | 0.035309812 | 9.99231E-10 | 8.52305E-09 |
| PRDX6 | -0.08438518 | 1.01749E-09 | 8.66415E-09 |
| TST | 0.035932178 | 1.0441E-09 | 8.8757E-09 |
| TNFSF10 | 0.040331135 | 1.07381E-09 | 9.11291E-09 |
| OGDHL | -0.068785732 | 1.13403E-09 | 9.60777E-09 |
| STK32B | 0.042527575 | 1.13957E-09 | 9.63848E-09 |
| SNRPN | -0.06364504 | 1.14147E-09 | 9.63848E-09 |
| S100A2 | -0.098132544 | 1.15385E-09 | 9.72671E-09 |
| NRCAM | 0.038162466 | 1.18022E-09 | 9.93236E-09 |
| MGAT5 | 0.049080117 | 1.2372E-09 | 1.03945E-08 |
| MAN2A1 | 0.041822477 | 1.27362E-09 | 1.06827E-08 |
| CPVL | 0.06712602 | 1.34627E-09 | 1.12734E-08 |
| ADAM10 | 0.037504753 | 1.35673E-09 | 1.13421E-08 |
| CHM | 0.031817801 | 1.41329E-09 | 1.17954E-08 |
| ORC4 | 0.038484281 | 1.43175E-09 | 1.19297E-08 |
| CEP70 | 0.035421052 | 1.44778E-09 | 1.20433E-08 |
| UBE2E2 | 0.034641306 | 1.46405E-09 | 1.21586E-08 |

| | | | |
|---|---|---|---|
| MUC15 | 0.075922823 | 1.47197E-09 | 1.22043E-08 |
| ZNF711 | 0.033742433 | 1.47928E-09 | 1.22448E-08 |
| ID2 | -0.062269616 | 1.52676E-09 | 1.2617E-08 |
| FBXO11 | 0.035976737 | 1.54439E-09 | 1.27418E-08 |
| CD63 | 0.105303868 | 1.60892E-09 | 1.32526E-08 |
| PIGR | -0.042966548 | 1.66451E-09 | 1.3688E-08 |
| HSD11B2 | -0.068609922 | 1.67502E-09 | 1.3752E-08 |
| C4orf19 | 0.029556776 | 1.68823E-09 | 1.3838E-08 |
| NR3C1 | 0.043790022 | 1.74615E-09 | 1.42895E-08 |
| UVRAG | 0.027242722 | 1.77123E-09 | 1.44713E-08 |
| PAQR7 | -0.028348825 | 1.89972E-09 | 1.5496E-08 |
| SENP7 | 0.034521527 | 1.97176E-09 | 1.60576E-08 |
| ZBTB1 | 0.029826803 | 1.98786E-09 | 1.6147E-08 |
| NGRN | -0.039750704 | 1.98914E-09 | 1.6147E-08 |
| PEBP1 | -0.146574961 | 2.01541E-09 | 1.63339E-08 |
| IER3IP1 | -0.038820717 | 2.05086E-09 | 1.65945E-08 |
| CCDC198 | 0.050372718 | 2.09498E-09 | 1.69243E-08 |
| NEK7 | 0.031616102 | 2.10973E-09 | 1.70162E-08 |
| SERF2 | -0.142956227 | 2.16727E-09 | 1.74524E-08 |
| DOCK1 | 0.04407501 | 2.35042E-09 | 1.8897E-08 |
| KAT6B | 0.043763532 | 2.38704E-09 | 1.91609E-08 |
| SDHC | -0.053801726 | 2.45413E-09 | 1.96682E-08 |
| FOXO3 | 0.040259134 | 2.72162E-09 | 2.17773E-08 |
| GRB14 | 0.044218237 | 2.78903E-09 | 2.22813E-08 |
| TAB2 | 0.034263615 | 3.10622E-09 | 2.47761E-08 |
| COX7B | -0.134979609 | 3.20934E-09 | 2.55529E-08 |
| AC118549.1 | 0.035668975 | 3.21376E-09 | 2.55529E-08 |
| POU2F1 | 0.029795552 | 3.34886E-09 | 2.65852E-08 |
| KATNAL2 | 0.026182663 | 3.51276E-09 | 2.78425E-08 |
| CHCHD3 | 0.04268421 | 3.6851E-09 | 2.91626E-08 |
| NEDD9 | 0.031111404 | 3.70432E-09 | 2.92688E-08 |
| ELF1 | 0.032404538 | 3.90618E-09 | 3.08155E-08 |
| SLC25A27 | 0.027283333 | 4.09533E-09 | 3.22571E-08 |
| PSPC1 | 0.029654486 | 4.26561E-09 | 3.35459E-08 |
| PHC3 | 0.034727482 | 4.44801E-09 | 3.49259E-08 |
| NCOA7 | 0.037618087 | 4.55097E-09 | 3.56788E-08 |
| ELP4 | 0.031079419 | 4.59031E-09 | 3.59313E-08 |
| CDK13 | 0.037734024 | 4.93456E-09 | 3.85661E-08 |
| TFCP2 | 0.028653457 | 5.28041E-09 | 4.12052E-08 |
| NFIB | 0.042283832 | 5.41939E-09 | 4.22244E-08 |
| ZNF704 | 0.040831968 | 5.7043E-09 | 4.43756E-08 |
| RPS6KA6 | 0.03864337 | 5.79721E-09 | 4.50289E-08 |
| SKAP2 | 0.044851335 | 5.99129E-09 | 4.64648E-08 |
| EIF4G1 | -0.03873925 | 6.25798E-09 | 4.84585E-08 |
| KLHL24 | 0.040335438 | 6.39851E-09 | 4.94707E-08 |
| KIAA0586 | 0.025366091 | 6.41351E-09 | 4.95107E-08 |
| MIEN1 | -0.043942862 | 7.07419E-09 | 5.45275E-08 |

| | | | |
|---|---|---|---|
| ATP5MG | -0.122122635 | 7.33598E-09 | 5.64591E-08 |
| PAM | 0.04165667 | 7.89514E-09 | 6.06698E-08 |
| ARMCX4 | 0.026534474 | 8.53852E-09 | 6.5514E-08 |
| CREB1 | 0.028453853 | 8.75796E-09 | 6.70956E-08 |
| HSPA2 | -0.070193746 | 8.78054E-09 | 6.71664E-08 |
| HEBP2 | -0.043814655 | 8.90816E-09 | 6.80394E-08 |
| BLNK | 0.034723066 | 9.00938E-09 | 6.87085E-08 |
| TXN | -0.07627616 | 9.07088E-09 | 6.9073E-08 |
| FTH1 | -0.15009469 | 9.56127E-09 | 7.26974E-08 |
| HMGB1 | -0.08409735 | 1.01514E-08 | 7.70683E-08 |
| HSPE1 | -0.086265174 | 1.0302E-08 | 7.8094E-08 |
| EID1 | -0.070603886 | 1.03752E-08 | 7.85302E-08 |
| STARD10 | -0.033076515 | 1.07209E-08 | 8.10256E-08 |
| TGFBR2 | 0.035733197 | 1.08506E-08 | 8.18829E-08 |
| EVI5 | 0.029531903 | 1.11015E-08 | 8.36509E-08 |
| CHD2 | 0.044233473 | 1.12502E-08 | 8.46449E-08 |
| CBR4 | 0.039279436 | 1.13535E-08 | 8.52947E-08 |
| PAXBP1 | 0.031132447 | 1.22949E-08 | 9.22298E-08 |
| CLPTM1L | -0.0297545 | 1.28838E-08 | 9.65037E-08 |
| GPC3 | 0.061755781 | 1.32036E-08 | 9.87527E-08 |
| HNRNPA0 | -0.035166363 | 1.3598E-08 | 1.01552E-07 |
| RABGEF1 | 0.027698579 | 1.37419E-08 | 1.02475E-07 |
| PICALM | 0.041598251 | 1.38821E-08 | 1.03367E-07 |
| ACOT11 | 0.038962057 | 1.42005E-08 | 1.05582E-07 |
| KIAA1109 | 0.048760318 | 1.43626E-08 | 1.0663E-07 |
| PGP | -0.030623359 | 1.4681E-08 | 1.08833E-07 |
| RALYL | 0.056003492 | 1.49647E-08 | 1.10774E-07 |
| KITLG | 0.030219809 | 1.57106E-08 | 1.16125E-07 |
| MROH7 | 0.026516585 | 1.61568E-08 | 1.19248E-07 |
| WWTR1 | 0.030656275 | 1.61806E-08 | 1.19249E-07 |
| MSI2 | 0.064158031 | 1.65207E-08 | 1.21578E-07 |
| COX7A2 | -0.106273521 | 1.66433E-08 | 1.22302E-07 |
| COX7C | -0.146022408 | 1.78473E-08 | 1.30958E-07 |
| NSUN6 | 0.026165481 | 1.83547E-08 | 1.34485E-07 |
| ZBTB7A | -0.035940588 | 1.86236E-08 | 1.36258E-07 |
| NUMB | 0.034860586 | 1.86807E-08 | 1.36477E-07 |
| SDCCAG8 | 0.037310164 | 1.90886E-08 | 1.39255E-07 |
| PHLDB2 | 0.050702694 | 1.94384E-08 | 1.41603E-07 |
| FGF9 | 0.061359231 | 1.96048E-08 | 1.42608E-07 |
| ANKRD9 | -0.046159626 | 1.9875E-08 | 1.44366E-07 |
| UTRN | 0.046066676 | 2.05203E-08 | 1.48839E-07 |
| ACBD6 | 0.030700653 | 2.10218E-08 | 1.52257E-07 |
| FASTKD1 | 0.025991106 | 2.12384E-08 | 1.53471E-07 |
| PPP1R1A | -0.101391789 | 2.12504E-08 | 1.53471E-07 |
| WDR27 | 0.024531381 | 2.15391E-08 | 1.55334E-07 |
| BMP6 | 0.028910939 | 2.22153E-08 | 1.59982E-07 |
| R3HDM1 | 0.025733114 | 2.25946E-08 | 1.62481E-07 |

| | | | |
|---|---|---|---|
| COX17 | -0.061704177 | 2.31689E-08 | 1.66374E-07 |
| KAT2B | 0.028915627 | 2.36168E-08 | 1.69349E-07 |
| PIK3C3 | 0.029917211 | 2.50021E-08 | 1.79028E-07 |
| LINC02121 | 0.039137301 | 2.52403E-08 | 1.80477E-07 |
| CCND1 | -0.053085552 | 2.59932E-08 | 1.85597E-07 |
| GPRC5C | -0.038604381 | 2.62678E-08 | 1.87293E-07 |
| TRIM2 | 0.068986543 | 2.64292E-08 | 1.88177E-07 |
| RAB1B | -0.042396296 | 2.6743E-08 | 1.90143E-07 |
| CENPB | -0.028719224 | 2.69424E-08 | 1.91291E-07 |
| COG6 | 0.026019191 | 2.77699E-08 | 1.96889E-07 |
| CDC42BPA | 0.035599008 | 2.79617E-08 | 1.9797E-07 |
| SET | -0.064592412 | 2.83551E-08 | 2.00474E-07 |
| RBM47 | 0.068299378 | 2.84572E-08 | 2.00914E-07 |
| TRA2A | 0.044722497 | 2.936E-08 | 2.06999E-07 |
| ZNF280D | 0.035116797 | 2.9898E-08 | 2.10497E-07 |
| LSM4 | -0.043888098 | 3.07603E-08 | 2.16266E-07 |
| OSBPL10 | 0.025506293 | 3.10926E-08 | 2.18298E-07 |
| C4orf3 | -0.044300733 | 3.17474E-08 | 2.22585E-07 |
| RAP1GDS1 | 0.024103444 | 3.49365E-08 | 2.44604E-07 |
| DSP | -0.031679934 | 3.62169E-08 | 2.53217E-07 |
| TCF7L2 | 0.033626261 | 3.77001E-08 | 2.63222E-07 |
| IDH2 | -0.081441736 | 3.9176E-08 | 2.73148E-07 |
| PVALB | -0.065131707 | 3.96843E-08 | 2.7631E-07 |
| RPS29 | 0.131970328 | 4.03898E-08 | 2.80835E-07 |
| RPRD2 | 0.031301528 | 4.21948E-08 | 2.9298E-07 |
| ASCC3 | 0.028934042 | 4.25595E-08 | 2.95106E-07 |
| NDUFB3 | -0.079522861 | 4.31404E-08 | 2.98724E-07 |
| YTHDC1 | 0.041769579 | 4.37107E-08 | 3.02257E-07 |
| SMAD2 | 0.034663326 | 4.51355E-08 | 3.11682E-07 |
| ATF7IP | 0.037852241 | 4.75263E-08 | 3.27743E-07 |
| PKM | -0.090569889 | 4.9454E-08 | 3.4057E-07 |
| TSC22D1 | 0.05223402 | 5.01051E-08 | 3.44584E-07 |
| ZNF131 | 0.023412963 | 5.08801E-08 | 3.49437E-07 |
| DYNC2LI1 | 0.034552077 | 5.14467E-08 | 3.52847E-07 |
| TFPI | 0.031528768 | 5.17263E-08 | 3.54283E-07 |
| HLA-B | -0.042831664 | 5.38045E-08 | 3.68017E-07 |
| NFKBIA | -0.029036594 | 5.45454E-08 | 3.72491E-07 |
| MPDZ | 0.033162453 | 5.46064E-08 | 3.72491E-07 |
| SLC25A4 | -0.068243457 | 5.53529E-08 | 3.77073E-07 |
| EIF5 | -0.052030105 | 5.62063E-08 | 3.8237E-07 |
| ST14 | -0.034602706 | 5.7368E-08 | 3.89747E-07 |
| SHPRH | 0.040904392 | 5.8087E-08 | 3.94101E-07 |
| ATP5PF | -0.107926766 | 5.98068E-08 | 4.05223E-07 |
| CLYBL | 0.030967774 | 6.08624E-08 | 4.11822E-07 |
| ST3GAL6 | 0.024239337 | 6.09716E-08 | 4.12008E-07 |
| GRB7 | 0.025882079 | 6.47769E-08 | 4.37136E-07 |
| GSN | -0.033315593 | 6.5206E-08 | 4.39443E-07 |

| | | | |
|---|---|---|---|
| ACACA | 0.023830598 | 7.08215E-08 | 4.76651E-07 |
| COL4A2 | 0.041981126 | 7.25065E-08 | 4.87341E-07 |
| LINC00476 | 0.026955385 | 7.31579E-08 | 4.91064E-07 |
| AGPAT4 | 0.031166155 | 7.39318E-08 | 4.95599E-07 |
| FAM13B | 0.02700883 | 7.40755E-08 | 4.95903E-07 |
| STARD8 | -0.026694274 | 7.45176E-08 | 4.98201E-07 |
| RABGAP1 | 0.032086851 | 7.61158E-08 | 5.08212E-07 |
| NAAA | 0.042787427 | 7.68052E-08 | 5.12136E-07 |
| MRFAP1 | -0.050484385 | 8.01812E-08 | 5.33941E-07 |
| WDFY2 | 0.034751379 | 8.04781E-08 | 5.35211E-07 |
| SOS2 | 0.034079096 | 8.09217E-08 | 5.37042E-07 |
| LINC01184 | 0.026872645 | 8.09665E-08 | 5.37042E-07 |
| LRRC8D | 0.026404283 | 8.11519E-08 | 5.37565E-07 |
| ANKIB1 | 0.034641643 | 8.2335E-08 | 5.44686E-07 |
| ZRANB2 | 0.049332786 | 8.28257E-08 | 5.47214E-07 |
| GLIS2 | -0.026508219 | 8.44344E-08 | 5.57112E-07 |
| PAGR1 | -0.024246313 | 1.07448E-07 | 7.08035E-07 |
| CEP95 | 0.028842912 | 1.08635E-07 | 7.14919E-07 |
| ARMC8 | 0.027197456 | 1.12362E-07 | 7.38487E-07 |
| XIST | 0.081632319 | 1.13937E-07 | 7.47857E-07 |
| ZNF721 | 0.028748739 | 1.16055E-07 | 7.60773E-07 |
| COPRS | -0.030946496 | 1.17514E-07 | 7.69334E-07 |
| UBR5 | 0.033104596 | 1.18396E-07 | 7.74105E-07 |
| MIDN | -0.025429727 | 1.20145E-07 | 7.84522E-07 |
| CLSTN1 | -0.047259923 | 1.23859E-07 | 8.07725E-07 |
| MAP7 | 0.029098037 | 1.27264E-07 | 8.28862E-07 |
| FOXN3 | 0.029844435 | 1.29082E-07 | 8.39613E-07 |
| FAM214A | 0.026293714 | 1.30608E-07 | 8.4845E-07 |
| CEP192 | 0.023858307 | 1.31177E-07 | 8.51046E-07 |
| AZIN1-AS1 | 0.024265097 | 1.31633E-07 | 8.5291E-07 |
| USP34 | 0.043979037 | 1.34176E-07 | 8.68267E-07 |
| ITCH | 0.028146136 | 1.37324E-07 | 8.87497E-07 |
| NT5DC1 | 0.034978929 | 1.39564E-07 | 9.00823E-07 |
| BHLHE40 | -0.037045599 | 1.40456E-07 | 9.05422E-07 |
| MAGEF1 | -0.034779915 | 1.45095E-07 | 9.34133E-07 |
| HOXD8 | -0.047953193 | 1.46874E-07 | 9.4438E-07 |
| METRNL | -0.037080176 | 1.58612E-07 | 1.01855E-06 |
| GCFC2 | 0.024675559 | 1.58902E-07 | 1.01911E-06 |
| LAPTM4A | 0.067082578 | 1.63973E-07 | 1.0503E-06 |
| HNRNPA2B1 | -0.08276401 | 1.66523E-07 | 1.06528E-06 |
| STX8 | 0.028981278 | 1.71767E-07 | 1.09744E-06 |
| RIN2 | 0.028610426 | 1.726E-07 | 1.10137E-06 |
| EIF4H | -0.038848279 | 1.75272E-07 | 1.117E-06 |
| NARS | -0.051450241 | 1.78628E-07 | 1.13695E-06 |
| ATXN7L3B | -0.035300125 | 1.79024E-07 | 1.13803E-06 |
| ITFG1 | 0.041930673 | 1.89301E-07 | 1.20185E-06 |
| BACH1 | 0.024626796 | 1.89717E-07 | 1.20298E-06 |

| | | | |
|---|---|---|---|
| IER5L | -0.02730282 | 1.9283E-07 | 1.22118E-06 |
| XPO1 | 0.038870667 | 2.05056E-07 | 1.29697E-06 |
| CA12 | -0.107516674 | 2.05913E-07 | 1.30076E-06 |
| MGLL | -0.045998394 | 2.07879E-07 | 1.31154E-06 |
| UBE2V1 | -0.036649354 | 2.11965E-07 | 1.33564E-06 |
| ARID4B | 0.034555734 | 2.18264E-07 | 1.37362E-06 |
| NUTM2B-AS1 | 0.027947052 | 2.20078E-07 | 1.38331E-06 |
| COX6C | -0.106384173 | 2.20578E-07 | 1.38473E-06 |
| CANX | -0.076275542 | 2.22164E-07 | 1.39295E-06 |
| GCNT2 | 0.03464646 | 2.22587E-07 | 1.39386E-06 |
| PRKAA2 | 0.051370248 | 2.29828E-07 | 1.43742E-06 |
| ID3 | 0.051231493 | 2.34076E-07 | 1.46218E-06 |
| LAMP1 | -0.048612412 | 2.41807E-07 | 1.5086E-06 |
| COX14 | -0.053333102 | 2.42725E-07 | 1.51246E-06 |
| XRN1 | 0.030621836 | 2.4538E-07 | 1.52538E-06 |
| RAB3GAP1 | 0.0307162 | 2.45404E-07 | 1.52538E-06 |
| HDDC2 | -0.047074607 | 2.46915E-07 | 1.53288E-06 |
| TAOK3 | 0.038923166 | 2.54842E-07 | 1.58014E-06 |
| FRMD4B | 0.028787514 | 2.64148E-07 | 1.63441E-06 |
| GATAD2B | 0.023264893 | 2.64241E-07 | 1.63441E-06 |
| EEA1 | 0.035196574 | 2.65897E-07 | 1.64263E-06 |
| POLR2C | -0.026747247 | 2.77792E-07 | 1.71328E-06 |
| SULT1C2 | 0.051761201 | 2.78013E-07 | 1.71328E-06 |
| DCDC2 | 0.03302543 | 2.81997E-07 | 1.73571E-06 |
| NUCKS1 | -0.068890188 | 2.84715E-07 | 1.7503E-06 |
| PTH1R | -0.074576054 | 2.88145E-07 | 1.76923E-06 |
| ME3 | 0.029890625 | 2.94647E-07 | 1.80695E-06 |
| TMEM59 | 0.083932092 | 2.95216E-07 | 1.80824E-06 |
| SLC4A4 | 0.029560892 | 3.02048E-07 | 1.84785E-06 |
| RAB28 | 0.025635075 | 3.0295E-07 | 1.85112E-06 |
| ARFGEF1 | 0.033463397 | 3.04394E-07 | 1.85769E-06 |
| GSTP1 | 0.103451406 | 3.28432E-07 | 2.00197E-06 |
| PTPN14 | 0.029752738 | 3.32226E-07 | 2.02265E-06 |
| GLCCI1 | 0.02441081 | 3.39683E-07 | 2.06555E-06 |
| PET100 | 0.065267103 | 3.40435E-07 | 2.06763E-06 |
| SMCO4 | -0.028314264 | 3.56326E-07 | 2.16154E-06 |
| SLC2A4RG | -0.042117048 | 3.70411E-07 | 2.24428E-06 |
| CNTN1 | 0.028501986 | 3.724E-07 | 2.25362E-06 |
| PYGB | -0.026485821 | 3.81876E-07 | 2.3082E-06 |
| HOXA-AS2 | 0.033108171 | 3.8672E-07 | 2.33468E-06 |
| FOXO1 | 0.026378798 | 3.91982E-07 | 2.36361E-06 |
| CHMP1B | -0.023894788 | 3.95909E-07 | 2.38444E-06 |
| ZFAND5 | -0.038660858 | 4.00566E-07 | 2.40961E-06 |
| SIM2 | 0.04172753 | 4.19315E-07 | 2.51939E-06 |
| PDLIM5 | 0.026763022 | 4.29357E-07 | 2.57665E-06 |
| RC3H1 | 0.023002203 | 4.37038E-07 | 2.61963E-06 |
| RBBP8 | 0.035437869 | 4.45218E-07 | 2.66549E-06 |

| | | | |
|---|---|---|---|
| LPL | 0.050125269 | 4.50185E-07 | 2.69203E-06 |
| UBE2E1 | 0.02779232 | 4.5843E-07 | 2.73809E-06 |
| MLEC | -0.04215632 | 4.69003E-07 | 2.79792E-06 |
| RPS14 | 0.119041238 | 4.73159E-07 | 2.81938E-06 |
| RALGPS2 | 0.027865158 | 4.91528E-07 | 2.92537E-06 |
| NR2F6 | -0.028820751 | 5.1383E-07 | 3.0545E-06 |
| PCM1 | 0.045475451 | 5.27188E-07 | 3.13022E-06 |
| SMIM8 | 0.028456929 | 5.28091E-07 | 3.13189E-06 |
| THOC2 | 0.032396038 | 5.35424E-07 | 3.17165E-06 |
| ATP8A1 | 0.023155396 | 5.40205E-07 | 3.19621E-06 |
| HIBCH | 0.039394672 | 5.52191E-07 | 3.2633E-06 |
| GCSH | -0.042427546 | 5.60481E-07 | 3.30841E-06 |
| SMARCAD1 | 0.025797454 | 5.84247E-07 | 3.44467E-06 |
| TMEM116 | 0.029410258 | 5.89299E-07 | 3.47039E-06 |
| KRT7 | -0.036909023 | 5.91064E-07 | 3.47673E-06 |
| EPB41L3 | 0.033469494 | 6.00569E-07 | 3.52852E-06 |
| ATP6V0E2 | -0.034210675 | 6.14679E-07 | 3.60721E-06 |
| SYNGR2 | -0.036282619 | 6.53867E-07 | 3.83273E-06 |
| SGCE | 0.029845159 | 6.64597E-07 | 3.8911E-06 |
| TRPM7 | 0.040699931 | 6.75147E-07 | 3.94828E-06 |
| TMEM161B-AS1 | 0.055110032 | 6.77784E-07 | 3.95911E-06 |
| VEZT | 0.027670598 | 6.83003E-07 | 3.98272E-06 |
| ATXN2 | 0.030576306 | 6.83407E-07 | 3.98272E-06 |
| METTL9 | -0.039990107 | 6.87685E-07 | 4.00303E-06 |
| TMEM176B | 0.051767744 | 6.91016E-07 | 4.01778E-06 |
| GNA11 | -0.027611166 | 7.14546E-07 | 4.1498E-06 |
| SPATA6 | 0.022136756 | 7.22493E-07 | 4.19112E-06 |
| ESD | 0.043231779 | 7.32449E-07 | 4.244E-06 |
| INTS6 | 0.031791878 | 7.37207E-07 | 4.26666E-06 |
| TPI1 | -0.074125447 | 7.42037E-07 | 4.28969E-06 |
| NDUFAB1 | -0.063936875 | 7.60113E-07 | 4.38915E-06 |
| EIF3E | 0.043355012 | 7.78896E-07 | 4.49247E-06 |
| UQCRH | -0.100053464 | 8.14612E-07 | 4.68806E-06 |
| BOLA3 | -0.042887296 | 8.14947E-07 | 4.68806E-06 |
| HERC1 | 0.031829592 | 8.15598E-07 | 4.68806E-06 |
| BOLA2 | -0.021952331 | 8.20394E-07 | 4.71026E-06 |
| PLEKHB2 | -0.038751079 | 8.23935E-07 | 4.7252E-06 |
| QTRT1 | 0.039979995 | 8.26185E-07 | 4.73273E-06 |
| NDUFC1 | -0.078855474 | 8.31599E-07 | 4.75833E-06 |
| UMOD | 0.493396596 | 8.59479E-07 | 4.91228E-06 |
| HELZ | 0.031895548 | 8.66701E-07 | 4.94715E-06 |
| PRH1 | 0.024158847 | 8.68033E-07 | 4.94715E-06 |
| ACPP | 0.052448044 | 8.68524E-07 | 4.94715E-06 |
| CDKL1 | 0.026278331 | 9.11217E-07 | 5.18448E-06 |
| SIPA1L1 | 0.02216297 | 9.15582E-07 | 5.20344E-06 |
| ACYP2 | 0.027150547 | 9.20868E-07 | 5.22759E-06 |
| CNOT2 | 0.029647651 | 9.35492E-07 | 5.30463E-06 |

| | | | |
|---|---|---|---|
| SREBF1 | -0.03602217 | 9.44967E-07 | 5.35234E-06 |
| PHF14 | 0.030040232 | 1.00136E-06 | 5.66537E-06 |
| ANKS1A | 0.020893846 | 1.02727E-06 | 5.80544E-06 |
| GSTA4 | 0.027083712 | 1.02957E-06 | 5.81192E-06 |
| PFKM | -0.028057216 | 1.04462E-06 | 5.89028E-06 |
| AHCYL1 | -0.053645376 | 1.0976E-06 | 6.1821E-06 |
| LAMTOR5 | -0.052842678 | 1.11878E-06 | 6.2858E-06 |
| MOB3B | 0.025748377 | 1.11883E-06 | 6.2858E-06 |
| PCBP1 | -0.051640442 | 1.1201E-06 | 6.2858E-06 |
| ADHFE1 | 0.021526718 | 1.121E-06 | 6.2858E-06 |
| POGZ | 0.039225208 | 1.1254E-06 | 6.30349E-06 |
| RUFY3 | 0.02642498 | 1.14678E-06 | 6.41612E-06 |
| ZNF644 | 0.031490838 | 1.16556E-06 | 6.51398E-06 |
| EIF5A | -0.041774778 | 1.18653E-06 | 6.62381E-06 |
| CYB5R3 | -0.032960162 | 1.22704E-06 | 6.84238E-06 |
| STRN3 | 0.025925928 | 1.23999E-06 | 6.90238E-06 |
| ANP32E | -0.030878372 | 1.24054E-06 | 6.90238E-06 |
| PPP3R1 | 0.021432992 | 1.27964E-06 | 7.11208E-06 |
| NDUFB2 | -0.08789969 | 1.34957E-06 | 7.49251E-06 |
| SCFD1 | 0.030190072 | 1.36272E-06 | 7.55715E-06 |
| PCMTD1 | 0.045085225 | 1.37101E-06 | 7.59477E-06 |
| CYSTM1 | -0.08926528 | 1.44231E-06 | 7.98102E-06 |
| NUDT3 | -0.031247917 | 1.51315E-06 | 8.36383E-06 |
| CISD3 | -0.029357099 | 1.53437E-06 | 8.47181E-06 |
| DUSP3 | -0.022663405 | 1.53786E-06 | 8.48181E-06 |
| NDUFB9 | -0.073166344 | 1.62726E-06 | 8.96506E-06 |
| C1orf122 | -0.038265497 | 1.68803E-06 | 9.28693E-06 |
| SRPRA | -0.024888587 | 1.68937E-06 | 9.28693E-06 |
| ARPC5 | -0.030216141 | 1.70381E-06 | 9.3561E-06 |
| HDLBP | -0.043039002 | 1.7936E-06 | 9.83847E-06 |
| TSPAN33 | -0.058879923 | 1.80955E-06 | 9.91517E-06 |
| DMTF1 | 0.031507907 | 1.82368E-06 | 9.98014E-06 |
| CC2D2A | 0.024580755 | 1.82537E-06 | 9.98014E-06 |
| GUK1 | -0.056082164 | 1.91654E-06 | 1.04672E-05 |
| TBC1D24 | -0.033154203 | 2.02347E-06 | 1.10393E-05 |
| BANF1 | -0.041508893 | 2.09224E-06 | 1.14021E-05 |
| WNK4 | -0.052770348 | 2.09907E-06 | 1.1427E-05 |
| ITGB3BP | 0.022904739 | 2.16162E-06 | 1.17548E-05 |
| FTL | -0.129616103 | 2.21708E-06 | 1.20434E-05 |
| CNOT4 | 0.026876411 | 2.25154E-06 | 1.22175E-05 |
| FAXDC2 | 0.024318649 | 2.25922E-06 | 1.22459E-05 |
| CAPN7 | 0.024501395 | 2.33259E-06 | 1.26301E-05 |
| SLC2A12 | 0.021059235 | 2.33901E-06 | 1.26512E-05 |
| ZNF710-AS1 | -0.030423006 | 2.35906E-06 | 1.2746E-05 |
| PPHLN1 | 0.023597725 | 2.40193E-06 | 1.29637E-05 |
| SMC5 | 0.023877133 | 2.4596E-06 | 1.32608E-05 |
| C11orf54 | 0.052090201 | 2.46813E-06 | 1.32925E-05 |

| | | | |
|---|---|---|---|
| ATR | 0.02446731 | 2.52021E-06 | 1.35586E-05 |
| TECR | 0.045355604 | 2.57252E-06 | 1.38252E-05 |
| FOXC1 | -0.031825218 | 2.64883E-06 | 1.42202E-05 |
| CST3 | 0.093657448 | 2.70118E-06 | 1.44858E-05 |
| NDUFB4 | -0.069167443 | 2.71009E-06 | 1.45181E-05 |
| CRIP2 | -0.047304498 | 2.74624E-06 | 1.46962E-05 |
| GPCPD1 | 0.02123736 | 2.78825E-06 | 1.49052E-05 |
| ZNF32 | -0.024836642 | 2.80197E-06 | 1.49626E-05 |
| NME4 | -0.041457294 | 2.85607E-06 | 1.52354E-05 |
| NUPR2 | -0.029186439 | 2.89321E-06 | 1.54047E-05 |
| NUDT4 | -0.075224036 | 2.89391E-06 | 1.54047E-05 |
| MAP2K4 | 0.022499041 | 2.90658E-06 | 1.54558E-05 |
| QKI | 0.040843836 | 2.92693E-06 | 1.55476E-05 |
| PDCD5 | -0.036338627 | 3.00714E-06 | 1.59457E-05 |
| URGCP | 0.022305149 | 3.00821E-06 | 1.59457E-05 |
| LUC7L2 | 0.038669605 | 3.15954E-06 | 1.67225E-05 |
| SLC25A5 | -0.08725186 | 3.16139E-06 | 1.67225E-05 |
| GTF3C6 | -0.035422796 | 3.20675E-06 | 1.69447E-05 |
| MXRA7 | -0.026396335 | 3.23085E-06 | 1.70541E-05 |
| DYNLL1 | -0.065905244 | 3.23424E-06 | 1.70542E-05 |
| GPATCH2L | 0.03081457 | 3.34456E-06 | 1.76175E-05 |
| ARSD | -0.038802413 | 3.41169E-06 | 1.79523E-05 |
| CLK1 | 0.037628238 | 3.4662E-06 | 1.82201E-05 |
| SPPL3 | 0.027353329 | 3.54705E-06 | 1.86257E-05 |
| CTTN | -0.030780878 | 3.68089E-06 | 1.93084E-05 |
| SAP18 | -0.056079035 | 3.69575E-06 | 1.93662E-05 |
| HNF1A-AS1 | 0.027306212 | 3.84283E-06 | 2.00996E-05 |
| ENO1 | -0.077823605 | 3.84368E-06 | 2.00996E-05 |
| ARRDC3 | 0.048002145 | 3.87528E-06 | 2.02421E-05 |
| RSRC1 | 0.023161118 | 3.87896E-06 | 2.02421E-05 |
| KIFC3 | 0.043724506 | 3.93893E-06 | 2.05338E-05 |
| ZHX3 | 0.035753311 | 3.96172E-06 | 2.06312E-05 |
| CCNDBP1 | -0.024655058 | 4.01693E-06 | 2.08971E-05 |
| REV1 | 0.026178264 | 4.02959E-06 | 2.09414E-05 |
| ZNF827 | 0.023293365 | 4.04529E-06 | 2.10014E-05 |
| GPX4 | -0.072611927 | 4.11969E-06 | 2.13656E-05 |
| CHD6 | 0.029885459 | 4.3328E-06 | 2.24477E-05 |
| NAA35 | 0.021288275 | 4.41277E-06 | 2.28386E-05 |
| WWC2 | 0.041721334 | 4.44441E-06 | 2.29787E-05 |
| CLU | 0.038574403 | 4.4492E-06 | 2.29799E-05 |
| PPIP5K2 | 0.029810106 | 4.47007E-06 | 2.30641E-05 |
| MRPS12 | -0.037643859 | 4.49412E-06 | 2.3129E-05 |
| GSK3B | 0.025105775 | 4.49594E-06 | 2.3129E-05 |
| TIMM13 | -0.04910297 | 4.49642E-06 | 2.3129E-05 |
| RB1 | 0.023101404 | 4.53793E-06 | 2.33188E-05 |
| NFIA | 0.038166256 | 4.57863E-06 | 2.3504E-05 |
| CWF19L2 | 0.023994423 | 4.61151E-06 | 2.36486E-05 |

| | | | |
|---|---|---|---|
| TFCP2L1 | -0.05031451 | 4.64861E-06 | 2.38147E-05 |
| RNF38 | 0.022048124 | 4.68357E-06 | 2.39694E-05 |
| CYP20A1 | 0.023120739 | 4.72743E-06 | 2.41693E-05 |
| DUSP15 | -0.028371062 | 4.78035E-06 | 2.44152E-05 |
| GGT6 | -0.031558571 | 4.9394E-06 | 2.5202E-05 |
| GNAI1 | 0.049489614 | 5.12697E-06 | 2.61325E-05 |
| ARID5B | 0.030318698 | 5.25343E-06 | 2.67501E-05 |
| RPL38 | 0.078780223 | 5.27499E-06 | 2.68327E-05 |
| PAWR | 0.032870362 | 5.28884E-06 | 2.68761E-05 |
| ZMYM4 | 0.028036437 | 5.39362E-06 | 2.73809E-05 |
| PIGN | 0.020231816 | 5.47358E-06 | 2.77589E-05 |
| VPS13A | 0.029916502 | 5.49918E-06 | 2.78607E-05 |
| MSH3 | 0.022386798 | 5.73046E-06 | 2.90033E-05 |
| PDLIM4 | -0.022565744 | 5.81577E-06 | 2.93999E-05 |
| ATP1B2 | -0.028169956 | 5.82049E-06 | 2.93999E-05 |
| SURF4 | -0.031984231 | 5.86505E-06 | 2.95953E-05 |
| FAM184A | 0.033243712 | 5.90178E-06 | 2.97509E-05 |
| STK39 | 0.022564784 | 5.95876E-06 | 3.00081E-05 |
| KLHDC2 | 0.031226457 | 6.01098E-06 | 3.02408E-05 |
| MIA2 | 0.035292268 | 6.17267E-06 | 3.10234E-05 |
| MAP4K5 | 0.020926631 | 6.38143E-06 | 3.20406E-05 |
| TRAPPC8 | 0.022277555 | 6.40006E-06 | 3.21022E-05 |
| PYURF | -0.034733875 | 6.59036E-06 | 3.30162E-05 |
| SDHD | -0.052989007 | 6.59538E-06 | 3.30162E-05 |
| HIBADH | 0.052867626 | 6.60734E-06 | 3.30433E-05 |
| RNMT | 0.030286681 | 6.74731E-06 | 3.37098E-05 |
| MCCD1 | -0.030183656 | 6.779E-06 | 3.38087E-05 |
| NTN4 | 0.037860032 | 6.78052E-06 | 3.38087E-05 |
| CTSB | -0.029762728 | 6.82459E-06 | 3.39948E-05 |
| KMT2E | 0.035807334 | 6.9915E-06 | 3.47918E-05 |
| SMG1 | 0.037857272 | 7.2052E-06 | 3.58007E-05 |
| XRRA1 | 0.019873529 | 7.20928E-06 | 3.58007E-05 |
| CCNT2 | 0.021810512 | 7.21554E-06 | 3.58007E-05 |
| EPC1 | 0.027978774 | 7.22679E-06 | 3.58213E-05 |
| IRX2 | -0.04806666 | 7.23724E-06 | 3.58378E-05 |
| RNPS1 | -0.029060666 | 7.29772E-06 | 3.61019E-05 |
| PPCS | -0.035812794 | 7.3333E-06 | 3.62423E-05 |
| POLK | 0.025105385 | 7.39985E-06 | 3.65354E-05 |
| CDK2AP1 | -0.021779839 | 7.41019E-06 | 3.65507E-05 |
| CAPN2 | -0.047493746 | 7.44126E-06 | 3.6668E-05 |
| FRS2 | 0.020633575 | 7.57048E-06 | 3.72683E-05 |
| DENND2A | 0.027682698 | 7.67816E-06 | 3.77616E-05 |
| COL4A4 | 0.03681702 | 7.91683E-06 | 3.88764E-05 |
| TMA7 | -0.082408565 | 7.92026E-06 | 3.88764E-05 |
| SPINT1-AS1 | -0.032287022 | 8.0448E-06 | 3.94493E-05 |
| USP22 | -0.031301927 | 8.43466E-06 | 4.13208E-05 |
| MACF1 | 0.035777428 | 8.53081E-06 | 4.17513E-05 |

| | | | |
|---|---|---|---|
| MRPL41 | -0.06992017 | 8.61535E-06 | 4.21241E-05 |
| OAZ1 | -0.060459271 | 8.65533E-06 | 4.22786E-05 |
| CXXC5 | -0.037753791 | 8.93253E-06 | 4.35904E-05 |
| LCOR | 0.026146714 | 8.95504E-06 | 4.3658E-05 |
| CLDN10 | 0.092164344 | 9.01011E-06 | 4.3884E-05 |
| FNDC3A | 0.025373333 | 9.02052E-06 | 4.38923E-05 |
| ZNF33A | 0.025855721 | 9.1044E-06 | 4.42577E-05 |
| RCOR3 | 0.028790523 | 9.17968E-06 | 4.45807E-05 |
| ECH1 | -0.040122397 | 9.40353E-06 | 4.56238E-05 |
| BORCS7 | -0.029091608 | 9.54965E-06 | 4.62882E-05 |
| NUTM2A-AS1 | 0.027845673 | 9.76744E-06 | 4.72984E-05 |
| HIGD1A | -0.039413014 | 9.92071E-06 | 4.79945E-05 |
| PINK1 | -0.033682699 | 1.03169E-05 | 4.98635E-05 |
| SGK3 | 0.020074178 | 1.03583E-05 | 5.00154E-05 |
| RPL36AL | -0.0666572 | 1.04108E-05 | 5.02209E-05 |
| SRSF3 | -0.047155993 | 1.05292E-05 | 5.07434E-05 |
| PANTR1 | 0.042855697 | 1.05484E-05 | 5.07875E-05 |
| PSENEN | 0.027730034 | 1.05691E-05 | 5.08385E-05 |
| COBL | 0.024033202 | 1.06605E-05 | 5.12295E-05 |
| TSPAN3 | -0.034392493 | 1.06818E-05 | 5.12343E-05 |
| MAT2A | -0.027796406 | 1.06819E-05 | 5.12343E-05 |
| UPF2 | 0.024763059 | 1.08596E-05 | 5.20374E-05 |
| PRKACA | -0.022055033 | 1.09031E-05 | 5.2196E-05 |
| RAP1A | 0.031927527 | 1.10353E-05 | 5.27791E-05 |
| HSP90AA1 | -0.072351064 | 1.10652E-05 | 5.28717E-05 |
| ZC3H6 | 0.021840479 | 1.10789E-05 | 5.2887E-05 |
| ARHGAP29 | 0.040172311 | 1.12275E-05 | 5.35457E-05 |
| SNX14 | 0.028719352 | 1.13457E-05 | 5.40581E-05 |
| OSBPL9 | 0.042118489 | 1.14142E-05 | 5.43332E-05 |
| ALDH9A1 | -0.030085474 | 1.16022E-05 | 5.51761E-05 |
| ZDHHC21 | 0.027816249 | 1.1832E-05 | 5.62159E-05 |
| RPS11 | 0.07122314 | 1.18529E-05 | 5.62623E-05 |
| SP100 | 0.023081664 | 1.19494E-05 | 5.66669E-05 |
| TUSC1 | -0.023135569 | 1.20257E-05 | 5.69753E-05 |
| AGO3 | 0.022999013 | 1.23842E-05 | 5.86187E-05 |
| ZDHHC14 | 0.034368672 | 1.26482E-05 | 5.98121E-05 |
| CRYBG3 | 0.023412509 | 1.29183E-05 | 6.10318E-05 |
| KIFAP3 | 0.021372663 | 1.31489E-05 | 6.20634E-05 |
| PRKACB | 0.02725843 | 1.34743E-05 | 6.34862E-05 |
| TOM1L1 | 0.03202368 | 1.34756E-05 | 6.34862E-05 |
| CCDC66 | 0.024811149 | 1.35089E-05 | 6.35807E-05 |
| IRX1 | -0.032093406 | 1.35208E-05 | 6.35807E-05 |
| GNG5 | -0.057159014 | 1.36116E-05 | 6.39479E-05 |
| NPY1R | 0.028788122 | 1.36283E-05 | 6.3967E-05 |
| RAB5IF | -0.027371971 | 1.36519E-05 | 6.40181E-05 |
| RNF13 | 0.034841026 | 1.36668E-05 | 6.40281E-05 |
| CHD1 | 0.023119472 | 1.38169E-05 | 6.46713E-05 |

| | | | |
|---|---|---|---|
| ATP10D | 0.020028271 | 1.38366E-05 | 6.47036E-05 |
| SEC16A | -0.020331059 | 1.39025E-05 | 6.49513E-05 |
| CEP350 | 0.027734713 | 1.40152E-05 | 6.54171E-05 |
| KCNJ16 | 0.071253262 | 1.46997E-05 | 6.85487E-05 |
| PER3 | -0.025729921 | 1.47676E-05 | 6.88018E-05 |
| C11orf49 | 0.0192022 | 1.47816E-05 | 6.88036E-05 |
| SLC35B2 | -0.019059523 | 1.48177E-05 | 6.8908E-05 |
| KRT10 | -0.046232011 | 1.49179E-05 | 6.92729E-05 |
| SERINC2 | -0.038990759 | 1.49237E-05 | 6.92729E-05 |
| CCNI | -0.057231115 | 1.53985E-05 | 7.1411E-05 |
| MAL2 | -0.03559913 | 1.56088E-05 | 7.22574E-05 |
| SMARCA4 | -0.026488977 | 1.56097E-05 | 7.22574E-05 |
| WDR11 | 0.024248004 | 1.56878E-05 | 7.25526E-05 |
| MAGED1 | 0.035319432 | 1.59357E-05 | 7.36316E-05 |
| CRK | -0.022618431 | 1.5964E-05 | 7.36946E-05 |
| CHD7 | 0.018995889 | 1.59926E-05 | 7.37592E-05 |
| PIK3CB | 0.022736016 | 1.63105E-05 | 7.51567E-05 |
| ARFIP1 | 0.022275543 | 1.6341E-05 | 7.52284E-05 |
| MAPRE1 | -0.020910151 | 1.64713E-05 | 7.57588E-05 |
| TUBB2A | -0.03270074 | 1.6769E-05 | 7.70581E-05 |
| ASXL1 | 0.022233239 | 1.68433E-05 | 7.73291E-05 |
| HOXA3 | 0.023335052 | 1.72045E-05 | 7.89153E-05 |
| CMC4 | -0.019891909 | 1.7272E-05 | 7.9153E-05 |
| STK11 | -0.024538616 | 1.7684E-05 | 8.09673E-05 |
| PDIA6 | -0.044691228 | 1.77545E-05 | 8.12163E-05 |
| CCSER2 | 0.024808932 | 1.82815E-05 | 8.35511E-05 |
| FNIP1 | 0.021051969 | 1.83768E-05 | 8.39106E-05 |
| NDUFA8 | -0.044109062 | 1.84629E-05 | 8.42276E-05 |
| AP1B1 | -0.02021772 | 1.85281E-05 | 8.44486E-05 |
| MYEF2 | 0.030734137 | 1.88365E-05 | 8.57769E-05 |
| TBC1D16 | -0.024159392 | 1.89702E-05 | 8.63073E-05 |
| RYK | 0.022587332 | 1.91731E-05 | 8.71521E-05 |
| COASY | -0.021384513 | 1.96574E-05 | 8.92727E-05 |
| SUMO2 | -0.058633308 | 1.9681E-05 | 8.92996E-05 |
| OST4 | -0.06512689 | 1.98535E-05 | 9.00014E-05 |
| TEX261 | -0.020238775 | 2.00082E-05 | 9.06212E-05 |
| RAB11B | -0.031836284 | 2.01418E-05 | 9.11445E-05 |
| MYH9 | -0.032954498 | 2.03155E-05 | 9.18478E-05 |
| YWHAE | -0.05932729 | 2.03384E-05 | 9.18689E-05 |
| ARF5 | 0.031879927 | 2.04766E-05 | 9.24103E-05 |
| UNC13B | 0.021000232 | 2.1024E-05 | 9.47962E-05 |
| ZNF148 | 0.030614532 | 2.11851E-05 | 9.54369E-05 |
| KLF13 | -0.021656595 | 2.13974E-05 | 9.63075E-05 |
| DDR1 | -0.033808377 | 2.21354E-05 | 9.95401E-05 |
| PTPRF | -0.028358948 | 2.22248E-05 | 9.98531E-05 |
| SERP1 | -0.042284888 | 2.24849E-05 | 0.000100932 |
| UBE2E3 | 0.038887195 | 2.35526E-05 | 0.000105582 |

| | | | |
|---|---|---|---|
| UBE2W | 0.021157002 | 2.35627E-05 | 0.000105582 |
| SLC25A11 | -0.031678931 | 2.36147E-05 | 0.000105721 |
| CHCHD7 | -0.021876626 | 2.37955E-05 | 0.000106436 |
| GLO1 | -0.034432651 | 2.38177E-05 | 0.000106441 |
| THBS1 | 0.020368588 | 2.40408E-05 | 0.000107343 |
| COX5A | -0.062397448 | 2.40646E-05 | 0.000107354 |
| MYCBP2 | 0.029488948 | 2.41583E-05 | 0.000107586 |
| CNNM2 | 0.020054456 | 2.41593E-05 | 0.000107586 |
| FKBP4 | -0.032631287 | 2.43542E-05 | 0.000108358 |
| PTPN11 | -0.024706251 | 2.44414E-05 | 0.00010865 |
| CNOT6L | 0.026093493 | 2.46911E-05 | 0.000109663 |
| ZFPM1 | -0.021362147 | 2.49832E-05 | 0.000110863 |
| ATRN | 0.026176214 | 2.50437E-05 | 0.000111034 |
| HYAL1 | -0.020052956 | 2.58549E-05 | 0.000114529 |
| CLINT1 | -0.035650635 | 2.64893E-05 | 0.000117237 |
| TMX3 | 0.020949508 | 2.6667E-05 | 0.00011792 |
| NCL | -0.04524847 | 2.6984E-05 | 0.000119217 |
| MXD4 | -0.028406873 | 2.70801E-05 | 0.000119537 |
| SMARCA2 | 0.032414454 | 2.72515E-05 | 0.000120188 |
| SPATA13 | 0.027405946 | 2.7565E-05 | 0.000121368 |
| RPAP2 | 0.021743936 | 2.75673E-05 | 0.000121368 |
| KIF16B | 0.022230587 | 2.7661E-05 | 0.000121674 |
| ACADVL | -0.048741061 | 2.79065E-05 | 0.000122647 |
| TMEM203 | -0.022243921 | 2.82661E-05 | 0.00012412 |
| SELENOH | -0.040141446 | 2.84164E-05 | 0.000124671 |
| NINL | 0.025319648 | 2.84436E-05 | 0.000124682 |
| TAPBP | -0.025049618 | 2.94622E-05 | 0.000129034 |
| WDR33 | 0.025586165 | 2.96908E-05 | 0.000129923 |
| ATPAF1 | -0.026449112 | 2.98561E-05 | 0.000130533 |
| STRBP | 0.025861887 | 3.0813E-05 | 0.000134571 |
| TSC22D2 | 0.028316078 | 3.08331E-05 | 0.000134571 |
| SNHG8 | 0.048381173 | 3.12407E-05 | 0.000136232 |
| RARS2 | 0.018066718 | 3.13693E-05 | 0.000136674 |
| CNP | -0.030001122 | 3.14883E-05 | 0.000137075 |
| ADGRV1 | 0.021373254 | 3.15434E-05 | 0.000137196 |
| SEC14L1 | -0.029436331 | 3.1772E-05 | 0.000138071 |
| SAT1 | -0.050474779 | 3.22047E-05 | 0.000139831 |
| CALM3 | -0.031126938 | 3.24537E-05 | 0.000140791 |
| TAF10 | -0.029764563 | 3.26074E-05 | 0.000141336 |
| EPS15 | 0.025409769 | 3.35816E-05 | 0.000145434 |
| CENPC | 0.023643906 | 3.39726E-05 | 0.000147001 |
| PHACTR4 | 0.024245115 | 3.46397E-05 | 0.000149759 |
| DTWD1 | 0.022347655 | 3.47667E-05 | 0.000150179 |
| TPT1-AS1 | 0.021023633 | 3.48555E-05 | 0.000150434 |
| COMMD1 | 0.024991063 | 3.50624E-05 | 0.000151197 |
| PSMD12 | -0.025201726 | 3.52433E-05 | 0.000151848 |
| IFIT1 | 0.023585327 | 3.56179E-05 | 0.00015333 |

| | | | |
|---|---|---|---|
| ATP5F1A | -0.063647477 | 3.67032E-05 | 0.000157867 |
| PACS1 | 0.01904943 | 3.75667E-05 | 0.000161444 |
| NUP214 | 0.022289382 | 3.78026E-05 | 0.000162319 |
| TRPM4 | -0.021815615 | 3.8005E-05 | 0.000163049 |
| ATP6V0C | -0.057395438 | 3.81826E-05 | 0.000163672 |
| FGL2 | -0.028482573 | 3.8307E-05 | 0.000164066 |
| TMEM176A | 0.036154098 | 3.90762E-05 | 0.000167218 |
| RPL36A | 0.073072947 | 3.93849E-05 | 0.000168396 |
| HS2ST1 | 0.020758047 | 3.97429E-05 | 0.000169783 |
| RPL14 | -0.082521208 | 4.03002E-05 | 0.000172018 |
| SLMAP | 0.022341789 | 4.06888E-05 | 0.00017353 |
| PPP6R2 | 0.031246903 | 4.13961E-05 | 0.000176397 |
| HEATR5B | 0.017897959 | 4.15852E-05 | 0.000177053 |
| ARHGEF7 | 0.022961793 | 4.25246E-05 | 0.0001809 |
| DNASE1 | 0.053013097 | 4.27545E-05 | 0.000181725 |
| PPM1A | 0.02417724 | 4.33585E-05 | 0.000184137 |
| CNDP2 | -0.034128802 | 4.34915E-05 | 0.000184546 |
| TBL1XR1 | 0.02777903 | 4.35764E-05 | 0.000184751 |
| CBX5 | -0.036953357 | 4.39818E-05 | 0.000186313 |
| ITM2B | 0.110304762 | 4.42921E-05 | 0.00018747 |
| DCXR | 0.050121506 | 4.44909E-05 | 0.000188153 |
| ZNF664 | -0.026161792 | 4.45785E-05 | 0.000188366 |
| TOM1L2 | 0.029826955 | 4.54659E-05 | 0.000191955 |
| ATXN10 | 0.026788178 | 4.59239E-05 | 0.000193726 |
| LAMTOR3 | -0.024658053 | 4.59696E-05 | 0.000193756 |
| KIDINS220 | 0.035934073 | 4.61497E-05 | 0.000194353 |
| SYTL2 | 0.023867519 | 4.69444E-05 | 0.000197535 |
| ASH1L | 0.036412354 | 4.79692E-05 | 0.000201537 |
| EIF1 | -0.083354462 | 4.79756E-05 | 0.000201537 |
| MFF | 0.030916901 | 4.8513E-05 | 0.000203626 |
| NDUFAF4 | -0.024632932 | 4.91455E-05 | 0.000205988 |
| PIK3CA | 0.017853806 | 4.91576E-05 | 0.000205988 |
| NSD1 | 0.028553928 | 4.95926E-05 | 0.000207638 |
| PRKAR1A | -0.040452404 | 5.04764E-05 | 0.000211163 |
| NBDY | -0.032144163 | 5.10268E-05 | 0.000213179 |
| FAM120B | 0.017732949 | 5.1043E-05 | 0.000213179 |
| LARP1 | -0.030911556 | 5.14516E-05 | 0.000214708 |
| ASAP2 | 0.03070314 | 5.15219E-05 | 0.000214716 |
| WWC1 | 0.031598183 | 5.15387E-05 | 0.000214716 |
| LMAN1 | -0.045203974 | 5.18819E-05 | 0.000215968 |
| SLC25A33 | -0.022850246 | 5.23583E-05 | 0.000217771 |
| ROCK2 | 0.020488588 | 5.30406E-05 | 0.000220427 |
| CTDSPL2 | 0.017483767 | 5.30986E-05 | 0.000220441 |
| ATP6V1A | -0.037941378 | 5.31315E-05 | 0.000220441 |
| MIPEP | 0.018393166 | 5.4317E-05 | 0.000225175 |
| ZNF780B | 0.01812081 | 5.45787E-05 | 0.000226073 |
| CNBP | -0.034514665 | 5.51228E-05 | 0.000228139 |

| | | | |
|---|---|---|---|
| PHF3 | 0.034420545 | 5.54129E-05 | 0.000229152 |
| WDR1 | -0.028661287 | 5.57134E-05 | 0.000230206 |
| RPS2 | 0.06870724 | 5.66754E-05 | 0.000233989 |
| CLDN14 | 0.022128262 | 5.68222E-05 | 0.000234403 |
| SMIM15 | -0.023022112 | 5.72885E-05 | 0.000235952 |
| TMBIM6 | -0.071528181 | 5.72913E-05 | 0.000235952 |
| SRSF9 | -0.041911581 | 5.79897E-05 | 0.000238442 |
| HOXB2 | -0.025624854 | 5.79905E-05 | 0.000238442 |
| ZNF83 | 0.024967074 | 5.83042E-05 | 0.000239537 |
| RHOQ | -0.02070369 | 5.89246E-05 | 0.000241888 |
| EMC10 | -0.03286039 | 5.91315E-05 | 0.00024254 |
| GRSF1 | -0.022377258 | 5.93581E-05 | 0.000243272 |
| CCNB1IP1 | 0.02710539 | 5.95836E-05 | 0.000243998 |
| BLCAP | -0.026864052 | 5.96395E-05 | 0.000244028 |
| ZNF106 | -0.027514743 | 5.97326E-05 | 0.000244211 |
| DNAJC10 | -0.025180854 | 5.98053E-05 | 0.00024431 |
| TMEM30B | -0.019720983 | 6.01534E-05 | 0.000245533 |
| ID4 | 0.030248342 | 6.08799E-05 | 0.000248297 |
| PKD2 | 0.025886287 | 6.10155E-05 | 0.000248649 |
| VPS41 | 0.022422809 | 6.14812E-05 | 0.000250345 |
| COX7A2L | -0.041687274 | 6.28822E-05 | 0.000255747 |
| RTL8C | -0.029164867 | 6.29094E-05 | 0.000255747 |
| MPC1 | -0.072562028 | 6.32726E-05 | 0.000257016 |
| ZMAT2 | -0.021630797 | 6.35412E-05 | 0.0002579 |
| ISOC2 | -0.024095712 | 6.3709E-05 | 0.000258373 |
| LYST | 0.018000175 | 6.39497E-05 | 0.00025914 |
| TEAD1 | 0.027125822 | 6.42705E-05 | 0.000260231 |
| MARCH7 | 0.021637052 | 6.46372E-05 | 0.000261506 |
| LAP3 | -0.020300216 | 6.49466E-05 | 0.000262547 |
| TANK | 0.020288956 | 6.54341E-05 | 0.000264305 |
| SNX24 | 0.018367605 | 6.60372E-05 | 0.000266404 |
| SHLD2 | 0.025415593 | 6.60594E-05 | 0.000266404 |
| TGOLN2 | -0.038495987 | 6.63136E-05 | 0.000267216 |
| NAT8L | -0.031842593 | 6.67693E-05 | 0.000268837 |
| CCDC8 | -0.021582521 | 6.69329E-05 | 0.000269281 |
| NEMF | 0.024325806 | 6.7163E-05 | 0.000269991 |
| SNRK | 0.02678853 | 6.72919E-05 | 0.000270294 |
| CBX6 | -0.022338779 | 6.74417E-05 | 0.00027068 |
| ANAPC10 | 0.017406152 | 6.80521E-05 | 0.000272912 |
| SUMO3 | -0.023617422 | 6.96037E-05 | 0.000278913 |
| PTPRA | 0.031724736 | 6.96932E-05 | 0.000279049 |
| CCDC14 | 0.02341536 | 6.99045E-05 | 0.000279674 |
| DAB2IP | -0.019742734 | 6.99993E-05 | 0.000279831 |
| ZMAT1 | 0.028093332 | 7.03696E-05 | 0.000281088 |
| GABARAPL2 | -0.048898171 | 7.16537E-05 | 0.000285991 |
| TSC22D4 | -0.027810855 | 7.21519E-05 | 0.000287751 |
| METTL5 | -0.021749533 | 7.40708E-05 | 0.000295171 |

| | | | |
|---|---|---|---|
| PUM3 | 0.017359998 | 7.46577E-05 | 0.000297274 |
| MICU2 | 0.033157024 | 7.53353E-05 | 0.000299736 |
| ICMT | -0.020069295 | 7.69616E-05 | 0.000305965 |
| AKAP10 | 0.017814648 | 7.71708E-05 | 0.000306555 |
| MAZ | -0.023389983 | 7.8078E-05 | 0.000309915 |
| WSB1 | -0.055026622 | 7.84271E-05 | 0.000311055 |
| PPP3CB | 0.02129881 | 7.87015E-05 | 0.000311898 |
| TBL1X | 0.024536727 | 7.96842E-05 | 0.000315544 |
| IFT88 | 0.022961487 | 7.98546E-05 | 0.000315971 |
| COPB1 | 0.028927527 | 8.04658E-05 | 0.00031814 |
| ANKRD28 | 0.023238556 | 8.26214E-05 | 0.000326406 |
| NDUFB5 | -0.048368591 | 8.43434E-05 | 0.000332948 |
| TXNL4A | -0.030049619 | 8.82222E-05 | 0.000347987 |
| COA4 | -0.024129734 | 9.07887E-05 | 0.000357831 |
| TPST2 | -0.026576579 | 9.3508E-05 | 0.000368261 |
| PPIF | -0.029138627 | 9.38953E-05 | 0.000369497 |
| RRBP1 | -0.039702714 | 9.41424E-05 | 0.000370181 |
| CD2AP | 0.028315926 | 9.5184E-05 | 0.000373985 |
| SPINT1 | -0.028785794 | 9.55435E-05 | 0.000375105 |
| CPT1A | -0.026510181 | 9.57855E-05 | 0.000375763 |
| CTDNEP1 | -0.020797226 | 9.72799E-05 | 0.000381328 |
| GPRIN3 | 0.018199485 | 9.75572E-05 | 0.000382118 |
| RNPC3 | 0.032387586 | 9.92749E-05 | 0.000388544 |
| CEBPD | -0.024891982 | 0.000100833 | 0.000394334 |
| TYRP1 | 0.030939665 | 0.000101849 | 0.000397999 |
| FAM200B | -0.034029812 | 0.000102479 | 0.000400151 |
| SEPHS2 | -0.033543625 | 0.0001029 | 0.000401486 |
| DST | 0.041350485 | 0.000103655 | 0.000404119 |
| SETD2 | 0.027466383 | 0.000107229 | 0.000417729 |
| DZIP1 | 0.025682827 | 0.000107374 | 0.000417972 |
| AKAP9 | 0.052529123 | 0.000109304 | 0.000425138 |
| RPS10 | -0.065752348 | 0.000109384 | 0.000425138 |
| LARP4B | 0.022541344 | 0.000111949 | 0.000434772 |
| RAC1 | -0.040042242 | 0.00011285 | 0.000437933 |
| SNRPG | -0.040810762 | 0.000113267 | 0.000438902 |
| PIP4K2C | -0.019297042 | 0.000113273 | 0.000438902 |
| ILF2 | -0.02511956 | 0.00011343 | 0.00043917 |
| TRAK1 | 0.017081893 | 0.000113965 | 0.000440905 |
| PBRM1 | 0.021220353 | 0.0001146 | 0.000443019 |
| HAT1 | 0.018441869 | 0.00011581 | 0.000447355 |
| C19orf53 | -0.042443058 | 0.00011937 | 0.000460755 |
| BPGM | -0.017775212 | 0.000120794 | 0.000465895 |
| ERICH1 | 0.028900439 | 0.000122096 | 0.000470557 |
| MED23 | 0.018958134 | 0.000123115 | 0.000474121 |
| SDF4 | -0.028688973 | 0.000123468 | 0.000475112 |
| ATP2C1 | 0.031673568 | 0.000123561 | 0.000475112 |
| MAP3K7 | 0.016531203 | 0.000124286 | 0.000477534 |

| | | | |
|---|---|---|---|
| RASSF8 | 0.026868518 | 0.00012701 | 0.000487628 |
| LMBRD1 | 0.039192383 | 0.000127626 | 0.000489622 |
| NME5 | 0.017189966 | 0.000127788 | 0.00048987 |
| NT5C2 | 0.017904965 | 0.000130192 | 0.000498707 |
| XBP1 | -0.027567238 | 0.000130331 | 0.000498859 |
| VPS45 | 0.018628843 | 0.000131027 | 0.000501142 |
| DBI | -0.045937409 | 0.00013232 | 0.000505706 |
| RDH11 | -0.024643938 | 0.000132585 | 0.000506336 |
| SOD2 | -0.037778815 | 0.000132875 | 0.000507056 |
| PIGT | -0.022515734 | 0.000133035 | 0.000507285 |
| SLC4A7 | -0.027245239 | 0.000133411 | 0.000508335 |
| BAG1 | -0.039510636 | 0.000134417 | 0.000511781 |
| NCOA4 | -0.030949533 | 0.000134695 | 0.000512452 |
| SMG7 | 0.021795373 | 0.000138223 | 0.000525479 |
| CPLANE1 | 0.020417129 | 0.000139138 | 0.000528558 |
| FNBP1L | 0.034895265 | 0.000142305 | 0.000540179 |
| COA1 | 0.023975426 | 0.000142962 | 0.000542264 |
| TMEM167A | -0.031269565 | 0.000145159 | 0.000550187 |
| EI24 | -0.027610894 | 0.000146197 | 0.000553702 |
| NCOR1 | 0.032993253 | 0.000146907 | 0.000555974 |
| MAPKAPK2 | -0.020635081 | 0.00014827 | 0.000560712 |
| TRIP12 | 0.025754828 | 0.000149025 | 0.000563146 |
| LNX1 | 0.025002945 | 0.000150346 | 0.00056771 |
| C12orf57 | -0.040336939 | 0.000160413 | 0.000605272 |
| KIAA1191 | -0.022008942 | 0.0001613 | 0.000608162 |
| OIP5-AS1 | -0.036095327 | 0.000162054 | 0.00061055 |
| TEF | -0.017828731 | 0.000163302 | 0.000614792 |
| TMEM164 | 0.018582759 | 0.000164768 | 0.000619849 |
| MLF2 | -0.03332987 | 0.000165092 | 0.000620602 |
| FIP1L1 | 0.020863004 | 0.000165546 | 0.000621845 |
| ANKRD26 | 0.023033911 | 0.000166774 | 0.000625993 |
| PPP2R1A | -0.03078247 | 0.000167614 | 0.000628676 |
| SPIN1 | 0.030463745 | 0.000168039 | 0.000629803 |
| BMPR2 | 0.019604629 | 0.000168377 | 0.000630601 |
| SMURF2 | 0.018599207 | 0.000169867 | 0.00063571 |
| ITGB1BP1 | -0.037178552 | 0.000171893 | 0.000642815 |
| MAPKAPK3 | -0.018821005 | 0.000173591 | 0.000648683 |
| HSBP1 | -0.049207871 | 0.000173935 | 0.000649486 |
| EFHD1 | -0.051560881 | 0.000174773 | 0.000652132 |
| MGME1 | 0.018840703 | 0.000176138 | 0.000656738 |
| HEATR5A | 0.017345332 | 0.000178639 | 0.000665572 |
| ACADM | -0.037645258 | 0.000179998 | 0.000670141 |
| ZBTB4 | -0.018920393 | 0.000180173 | 0.000670296 |
| PSMB1 | 0.039264805 | 0.000181617 | 0.000674757 |
| SH3BGRL3 | -0.025308783 | 0.00018164 | 0.000674757 |
| NDUFS6 | -0.061876404 | 0.000182068 | 0.000675851 |
| CLN8 | -0.019915708 | 0.00018248 | 0.00067688 |

| | | | |
|---|---|---|---|
| PBLD | -0.025198002 | 0.000184813 | 0.000685032 |
| CUL3 | 0.027076904 | 0.000184953 | 0.000685047 |
| FBXO2 | -0.018539947 | 0.000186294 | 0.000689193 |
| LAMB1 | 0.036078518 | 0.000186346 | 0.000689193 |
| TNS3 | 0.02284224 | 0.000186925 | 0.000690829 |
| MALAT1 | 0.421463832 | 0.000190927 | 0.000705101 |
| AUH | 0.030218852 | 0.000191467 | 0.000706576 |
| EIF3M | 0.02447909 | 0.000193318 | 0.000712886 |
| GTF3A | -0.021072405 | 0.000193707 | 0.0007138 |
| GBF1 | 0.01685437 | 0.000194345 | 0.000715627 |
| ODF2L | 0.020746221 | 0.000195482 | 0.000719287 |
| H2AFZ | -0.033985048 | 0.000198604 | 0.000730243 |
| SUGP2 | -0.023279093 | 0.000200931 | 0.000738259 |
| EMC2 | 0.021259337 | 0.000206869 | 0.000759525 |
| SBDS | -0.026517799 | 0.000207166 | 0.000760059 |
| SECISBP2L | 0.025753784 | 0.000208175 | 0.000763209 |
| C9orf16 | -0.025863103 | 0.000209215 | 0.000766464 |
| KIAA1522 | -0.024512508 | 0.000210414 | 0.000770296 |
| POLDIP2 | -0.027240165 | 0.000211645 | 0.000774241 |
| MIR503HG | -0.024755686 | 0.000212757 | 0.000777463 |
| SMCHD1 | 0.022586437 | 0.000212835 | 0.000777463 |
| TNIP1 | -0.025426832 | 0.000214618 | 0.000783411 |
| MSANTD2 | 0.016202091 | 0.000216039 | 0.000788025 |
| CMPK1 | -0.02223005 | 0.000218097 | 0.000794957 |
| C1orf43 | -0.030318221 | 0.000218773 | 0.000796845 |
| ID1 | 0.035445695 | 0.000219094 | 0.000797437 |
| ATF6 | 0.021952867 | 0.00022054 | 0.000802123 |
| PPDPF | 0.063390871 | 0.000220842 | 0.000802561 |
| CTBP1 | -0.021986949 | 0.000220979 | 0.000802561 |
| UBE2N | -0.02728461 | 0.000222279 | 0.000806702 |
| SPCS1 | -0.043865109 | 0.000223202 | 0.000809467 |
| RPL39 | 0.104677423 | 0.000224109 | 0.000812175 |
| ELP2 | 0.018381644 | 0.000225604 | 0.000817004 |
| ZNF503 | -0.036621256 | 0.000229662 | 0.000831102 |
| RBM33 | 0.018502193 | 0.000230746 | 0.000834427 |
| RPL37A | 0.073799872 | 0.000231929 | 0.000838102 |
| RPS26 | 0.07814316 | 0.000234311 | 0.000846103 |
| DCAF7 | -0.021850271 | 0.000236285 | 0.000852622 |
| IFITM2 | -0.024153762 | 0.000236813 | 0.000853915 |
| ATP5MD | -0.072203834 | 0.000239323 | 0.000862349 |
| COX6B1 | -0.078442393 | 0.000239674 | 0.000862997 |
| BTBD7 | 0.024036536 | 0.000242201 | 0.000870853 |
| CMSS1 | 0.01661504 | 0.000242201 | 0.000870853 |
| HLA-A | -0.045686586 | 0.000242808 | 0.000872412 |
| MRPS7 | -0.026628816 | 0.000243799 | 0.000875349 |
| FBXW11 | 0.025448179 | 0.000245378 | 0.000880393 |
| PLIN5 | 0.022847395 | 0.000249412 | 0.000894229 |

| | | | |
|---|---|---|---|
| PDHB | -0.027792211 | 0.000249877 | 0.000895261 |
| BACE2 | 0.047247595 | 0.000256226 | 0.000917355 |
| B4GALT1 | -0.022813207 | 0.000256444 | 0.000917483 |
| INSR | 0.029089134 | 0.000258861 | 0.000925473 |
| SH3GLB2 | -0.024394076 | 0.000261634 | 0.000934726 |
| CMTM6 | -0.021741905 | 0.000262224 | 0.000936168 |
| MRPL18 | -0.023264798 | 0.000266987 | 0.000952498 |
| FBXL4 | 0.025043215 | 0.000268297 | 0.000956496 |
| NGLY1 | 0.01728907 | 0.000270929 | 0.000965197 |
| CYBA | 0.033998006 | 0.000274358 | 0.000976724 |
| PHF10 | -0.019566911 | 0.00027456 | 0.000976753 |
| ADIRF | 0.074669814 | 0.000274816 | 0.000976974 |
| ARL1 | -0.028678823 | 0.000279379 | 0.000992493 |
| SDF2L1 | -0.020359487 | 0.000279989 | 0.000993963 |
| TRMT9B | 0.021178588 | 0.000282676 | 0.001002109 |
| CIAO2B | -0.031418364 | 0.000282682 | 0.001002109 |
| UBAC2 | 0.021977035 | 0.000282904 | 0.00100219 |
| MCF2L | 0.016618495 | 0.000285005 | 0.001008924 |
| P3H2 | 0.02407267 | 0.000286273 | 0.001012702 |
| TPT1 | 0.093332343 | 0.000287763 | 0.001017261 |
| GPS1 | -0.019899032 | 0.000288179 | 0.001018018 |
| MGAT4B | -0.020419957 | 0.000290313 | 0.001024839 |
| SUSD6 | -0.020509697 | 0.00029284 | 0.001032525 |
| NORAD | -0.03905189 | 0.0002929 | 0.001032525 |
| ZFP36L2 | -0.029235272 | 0.000293377 | 0.001033481 |
| DUSP9 | -0.056740042 | 0.000294638 | 0.001036559 |
| NPEPPS | 0.025168974 | 0.000294662 | 0.001036559 |
| IRF2BPL | -0.023895792 | 0.000296643 | 0.001042801 |
| DDB1 | -0.026079598 | 0.0003039 | 0.001067567 |
| PHC2 | -0.024132708 | 0.000304531 | 0.00106904 |
| LINC00671 | 0.026960243 | 0.000308369 | 0.00108176 |
| BICDL1 | 0.04640502 | 0.000314937 | 0.001104031 |
| MFSD14C | 0.01625776 | 0.000322106 | 0.001127672 |
| PIGS | -0.016584564 | 0.000322128 | 0.001127672 |
| USP47 | 0.023376752 | 0.000329185 | 0.001151578 |
| SMG6 | 0.01797239 | 0.000330232 | 0.00115412 |
| ATP5PO | -0.052780683 | 0.00033037 | 0.00115412 |
| FLNA | -0.021192099 | 0.00033124 | 0.001156359 |
| BEX4 | -0.032253218 | 0.000337343 | 0.001176847 |
| PURA | -0.030550921 | 0.000338266 | 0.001179254 |
| RHBDD2 | -0.018498215 | 0.00033987 | 0.001184027 |
| SEC61A1 | -0.023368393 | 0.000340435 | 0.001185174 |
| AOC1 | -0.019324677 | 0.000341644 | 0.001188562 |
| ACSL4 | -0.031238013 | 0.000343704 | 0.001194905 |
| GIGYF2 | 0.026382065 | 0.00034747 | 0.001207163 |
| KIAA2026 | 0.021725797 | 0.000351376 | 0.001219404 |
| RB1CC1 | 0.022243453 | 0.000351477 | 0.001219404 |

| | | | |
|---|---|---|---|
| PURB | -0.019785809 | 0.000352633 | 0.001222576 |
| SPTSSA | -0.020281398 | 0.000357057 | 0.001236374 |
| ANKHD1 | 0.024736505 | 0.000357104 | 0.001236374 |
| NDUFS1 | -0.036997067 | 0.000358035 | 0.001238748 |
| CFDP1 | 0.026923426 | 0.000360039 | 0.001244827 |
| ARHGAP5 | 0.032461795 | 0.000360853 | 0.001246785 |
| PNPO | -0.018062025 | 0.00036416 | 0.001257348 |
| REEP3 | 0.017801423 | 0.000365305 | 0.001260439 |
| STAT3 | -0.026004106 | 0.000367172 | 0.001266015 |
| HSPA12A | 0.018022915 | 0.000367558 | 0.00126648 |
| AMFR | -0.028274994 | 0.000368481 | 0.001268792 |
| CDC16 | 0.017665248 | 0.000369541 | 0.001271575 |
| RPS9 | 0.078246828 | 0.000370639 | 0.001274484 |
| ARHGAP12 | 0.020618767 | 0.000373463 | 0.001283319 |
| PRRC2A | -0.023073536 | 0.000378125 | 0.001298451 |
| NDUFA12 | -0.040683225 | 0.000378959 | 0.00130043 |
| ZADH2 | -0.019748106 | 0.000386615 | 0.0013258 |
| USP3 | 0.019384873 | 0.000392766 | 0.001345976 |
| EIF2AK1 | -0.022525029 | 0.000402786 | 0.001379379 |
| HSF1 | -0.024870114 | 0.000404028 | 0.001381431 |
| TMEM230 | -0.035865617 | 0.000404188 | 0.001381431 |
| WASHC4 | 0.021005386 | 0.000404208 | 0.001381431 |
| KCNJ10 | -0.030648477 | 0.000406539 | 0.001388457 |
| SLC35B1 | -0.015938364 | 0.000408864 | 0.001395454 |
| CIPC | -0.017226514 | 0.000410577 | 0.001400353 |
| HK1 | -0.017727552 | 0.00041427 | 0.00141199 |
| OCIAD1 | 0.040885189 | 0.000415797 | 0.001414948 |
| MED13 | 0.022880012 | 0.000415951 | 0.001414948 |
| RPRD1A | 0.019418455 | 0.00041598 | 0.001414948 |
| P4HB | -0.032925952 | 0.000418846 | 0.001423738 |
| SLC25A39 | -0.028290671 | 0.000420891 | 0.001429725 |
| IER2 | -0.020744936 | 0.000421491 | 0.0014308 |
| MFN2 | -0.019794157 | 0.000423784 | 0.001437345 |
| AASDH | 0.01959132 | 0.00042399 | 0.001437345 |
| SGK2 | 0.025760431 | 0.000425629 | 0.001441934 |
| SYTL4 | 0.018889035 | 0.00044284 | 0.001499233 |
| EPHX2 | -0.035058191 | 0.000443615 | 0.001500849 |
| GAS5 | 0.023225475 | 0.000444177 | 0.00150174 |
| OLA1 | 0.020933148 | 0.000446143 | 0.001507378 |
| KIF3B | -0.020402665 | 0.000452977 | 0.001529441 |
| NDUFS5 | -0.052666999 | 0.000454906 | 0.001534928 |
| ERMP1 | 0.021332428 | 0.000467253 | 0.001575535 |
| OMA1 | 0.019685285 | 0.000467828 | 0.001576417 |
| C1GALT1C1 | 0.016374308 | 0.00047427 | 0.001596812 |
| CMBL | -0.033183787 | 0.000474514 | 0.001596812 |
| GHITM | -0.038479934 | 0.000476847 | 0.001603593 |
| AK3 | 0.038806444 | 0.000480566 | 0.001615024 |

| | | | |
|---|---|---|---|
| ARPC5L | -0.018609253 | 0.000486414 | 0.001633587 |
| RPS27L | -0.037375359 | 0.000492231 | 0.001652021 |
| AK1 | -0.026501791 | 0.000495775 | 0.00166281 |
| SYNE2 | 0.051308739 | 0.000497469 | 0.00166738 |
| PPFIBP1 | 0.015720043 | 0.000502802 | 0.001684137 |
| ZMYND8 | 0.024175096 | 0.000504861 | 0.001689298 |
| PDS5B | 0.021797093 | 0.000505013 | 0.001689298 |
| SMAP1 | 0.016111448 | 0.000508092 | 0.001698468 |
| HNRNPF | -0.023837195 | 0.000515484 | 0.001722039 |
| VCP | -0.021721849 | 0.000515928 | 0.001722379 |
| LGR4 | 0.034524282 | 0.000518861 | 0.001730305 |
| ZMYND11 | 0.026698682 | 0.000518989 | 0.001730305 |
| CLDN3 | 0.020839956 | 0.00051964 | 0.001731301 |
| USP33 | 0.02046366 | 0.000519974 | 0.001731301 |
| NRIP1 | 0.023636631 | 0.000523335 | 0.00174134 |
| LMNA | -0.027108462 | 0.000529055 | 0.001759213 |
| HSD17B12 | -0.029554724 | 0.000532078 | 0.001768098 |
| RAB21 | -0.022656154 | 0.000540427 | 0.001794658 |
| SRA1 | -0.016241157 | 0.000541145 | 0.00179586 |
| DYNC1I2 | 0.025984147 | 0.000541794 | 0.001796832 |
| IGF2BP2 | 0.015327732 | 0.000548926 | 0.001819287 |
| DDX3X | -0.025593831 | 0.00055243 | 0.001829698 |
| ZNF33B | 0.018410018 | 0.000564119 | 0.001867186 |
| TMSB4X | -0.027749039 | 0.000564574 | 0.001867466 |
| SCYL2 | 0.016854457 | 0.000567222 | 0.001874993 |
| LMO7 | 0.032334611 | 0.000569253 | 0.001880474 |
| SLIRP | -0.039551767 | 0.000569748 | 0.001880878 |
| KLHDC10 | 0.019944506 | 0.000577272 | 0.001904469 |
| C1orf56 | -0.01722907 | 0.000582351 | 0.00191997 |
| RNF187 | -0.022897381 | 0.000585431 | 0.00192886 |
| PDGFA | -0.019314501 | 0.000590294 | 0.001943612 |
| LRPPRC | 0.03121134 | 0.000591401 | 0.001945987 |
| NDUFA5 | -0.04357141 | 0.000591851 | 0.001946199 |
| IDH3A | -0.016767475 | 0.000592246 | 0.001946226 |
| CSDE1 | -0.037089805 | 0.000596302 | 0.001958279 |
| RBM26 | 0.025348989 | 0.000598125 | 0.001962988 |
| EXOC1 | 0.019856902 | 0.000601242 | 0.001971932 |
| CIB1 | -0.028647802 | 0.000604427 | 0.001981089 |
| TSGA10 | 0.016561684 | 0.000605845 | 0.001984448 |
| KCTD3 | 0.017683158 | 0.000612872 | 0.002006161 |
| SLC16A7 | 0.05335072 | 0.000617453 | 0.002019845 |
| NAPEPLD | 0.017630677 | 0.000618525 | 0.00202204 |
| ATP5IF1 | -0.05319826 | 0.000620212 | 0.002026239 |
| CLDN16 | -0.06921932 | 0.000624665 | 0.002039465 |
| GBE1 | 0.023155965 | 0.000633722 | 0.002065636 |
| NDUFAF8 | -0.02969181 | 0.000633823 | 0.002065636 |
| RC3H2 | 0.015777103 | 0.00063391 | 0.002065636 |

| | | | |
|---|---|---|---|
| MIB1 | 0.022293069 | 0.000638671 | 0.002079805 |
| TUBB4B | -0.028567237 | 0.000642256 | 0.002090132 |
| HNRNPH2 | -0.021010698 | 0.000644578 | 0.002096334 |
| AL158152.1 | 0.017717475 | 0.000645688 | 0.002098589 |
| PPM1G | -0.020285907 | 0.00065037 | 0.002112445 |
| BMI1 | -0.018333201 | 0.000654482 | 0.002124433 |
| WWP1 | 0.021940986 | 0.000657762 | 0.002133705 |
| HLA-C | -0.034182829 | 0.000670545 | 0.002173774 |
| NDUFA6 | -0.039469372 | 0.000673799 | 0.002182918 |
| PREPL | 0.030131227 | 0.000674865 | 0.002183441 |
| RAB18 | -0.022766395 | 0.000674915 | 0.002183441 |
| ZNF44 | 0.024463273 | 0.000675568 | 0.002183441 |
| AKIRIN1 | -0.022780141 | 0.000675693 | 0.002183441 |
| NCBP2 | -0.016270947 | 0.000677253 | 0.002186177 |
| PKN2 | 0.02733185 | 0.000677407 | 0.002186177 |
| SELENOW | -0.04315742 | 0.000684482 | 0.002207598 |
| AFMID | -0.016244826 | 0.000686814 | 0.002213701 |
| SLC15A2 | 0.023189748 | 0.000689287 | 0.002219917 |
| SLC22A2 | 0.040105681 | 0.000689623 | 0.002219917 |
| RPL4 | -0.049519238 | 0.00069197 | 0.002226051 |
| RPL37 | 0.07430819 | 0.00070715 | 0.002273434 |
| MDN1 | 0.014652544 | 0.000711787 | 0.002286884 |
| POMP | -0.036838456 | 0.000714588 | 0.00229442 |
| SERBP1 | -0.033698295 | 0.000716763 | 0.002299936 |
| CPM | 0.033923518 | 0.000725997 | 0.002328085 |
| GPNMB | 0.027764935 | 0.000727025 | 0.002329901 |
| NUP58 | 0.014817464 | 0.000729323 | 0.002335781 |
| JAK1 | 0.02398436 | 0.000732256 | 0.002343684 |
| OCLN | 0.030906606 | 0.000733745 | 0.00234684 |
| URI1 | 0.023583467 | 0.000734173 | 0.00234684 |
| ARCN1 | -0.018197713 | 0.000739611 | 0.002362726 |
| SLC25A1 | -0.019169192 | 0.000743714 | 0.002374326 |
| COX5B | -0.082795438 | 0.000746744 | 0.002382491 |
| RBIS | -0.028049096 | 0.000747608 | 0.00238374 |
| CCDC71L | -0.018493145 | 0.000750394 | 0.00239111 |
| SNX13 | 0.021796547 | 0.000753118 | 0.002398273 |
| DNPH1 | -0.03151487 | 0.000754518 | 0.002401214 |
| AVL9 | 0.014770954 | 0.000756731 | 0.002406738 |
| ATE1 | 0.015714685 | 0.000781505 | 0.002483965 |
| CDH16 | -0.056896347 | 0.000782882 | 0.002486773 |
| SIPA1L3 | 0.01626221 | 0.000785229 | 0.002492656 |
| SON | -0.041246744 | 0.00078922 | 0.002503749 |
| VDAC2 | -0.038335226 | 0.000791647 | 0.002509868 |
| TUBB2B | -0.03262775 | 0.000795518 | 0.002520556 |
| SETDB2 | 0.016228499 | 0.000796567 | 0.002522296 |
| MYO10 | 0.026270503 | 0.000807823 | 0.002556331 |
| ERCC6L2 | 0.017439523 | 0.000808823 | 0.002556741 |

| | | | |
|---|---|---|---|
| ARNT2 | 0.020142063 | 0.000808967 | 0.002556741 |
| FAM168B | -0.022764412 | 0.000816029 | 0.002577446 |
| RPS28 | 0.100447624 | 0.000821227 | 0.002592239 |
| FKBP1A | -0.025164723 | 0.000825227 | 0.002602844 |
| MGMT | 0.021484916 | 0.000825619 | 0.002602844 |
| AIG1 | 0.026063955 | 0.000829953 | 0.002614871 |
| HRAS | -0.018983332 | 0.000837872 | 0.00263817 |
| SMAD4 | 0.022773002 | 0.000842876 | 0.002652271 |
| BRI3 | -0.042045857 | 0.000851659 | 0.002678235 |
| KAT6A | 0.018307692 | 0.000858288 | 0.002697401 |
| NHSL1 | 0.016256748 | 0.000866036 | 0.002720054 |
| PROM2 | -0.033972565 | 0.000867041 | 0.002721515 |
| GOLGA2 | -0.01828278 | 0.000870448 | 0.00273051 |
| OLIG1 | -0.016393041 | 0.000872346 | 0.002734761 |
| BRD8 | 0.016884949 | 0.00087312 | 0.002735488 |
| DIP2C | 0.014365511 | 0.000879094 | 0.002752491 |
| UQCRC1 | -0.038739815 | 0.000881238 | 0.002757492 |
| ACTR2 | -0.025965995 | 0.000887215 | 0.002774472 |
| SQSTM1 | -0.033003378 | 0.000887961 | 0.002775086 |
| CHPT1 | 0.021602844 | 0.000892986 | 0.002789059 |
| DCPS | 0.019614877 | 0.000895795 | 0.002796102 |
| ZXDC | 0.014394466 | 0.000903028 | 0.002816934 |
| CYC1 | -0.040824955 | 0.000908857 | 0.002833362 |
| ELAVL1 | -0.01886247 | 0.000916451 | 0.00285527 |
| MRPS33 | -0.025540583 | 0.000931982 | 0.002901867 |
| TOMM6 | -0.038180506 | 0.000942492 | 0.002932778 |
| NUDT8 | -0.018119929 | 0.00094397 | 0.002935565 |
| DNAJC21 | -0.021382714 | 0.000946396 | 0.002941296 |
| RNF141 | -0.017008635 | 0.000950409 | 0.002951948 |
| TMEM109 | -0.018566159 | 0.000956406 | 0.00296856 |
| SRP14 | -0.062900346 | 0.000956935 | 0.00296856 |
| CHKA | 0.018525209 | 0.000959948 | 0.002976076 |
| ATP11B | 0.014736854 | 0.000968482 | 0.003000686 |
| GID8 | -0.016594263 | 0.000974435 | 0.003017276 |
| FAM49B | 0.017737398 | 0.000980303 | 0.003033583 |
| ATP5PB | -0.0370431 | 0.000981706 | 0.003036061 |
| DBN1 | -0.019893136 | 0.000982652 | 0.003037125 |
| STOML2 | -0.024746971 | 0.000985793 | 0.003044964 |
| NUCB1 | -0.029531816 | 0.000989568 | 0.003054755 |
| PTP4A2 | -0.025520521 | 0.000992027 | 0.003060469 |
| GSAP | 0.017827537 | 0.000996949 | 0.003073772 |
| EMX2 | -0.030252198 | 0.001004992 | 0.003096677 |
| TMED4 | -0.026886066 | 0.00101234 | 0.003117414 |
| SEPTIN9 | -0.017222783 | 0.001021166 | 0.003142673 |
| FECH | -0.034759213 | 0.001024242 | 0.003150215 |
| F11R | -0.016014806 | 0.001024975 | 0.003150549 |
| KIF5B | -0.026033761 | 0.001041101 | 0.003198166 |

| | | | |
|---|---|---|---|
| ALKBH5 | -0.018400256 | 0.001052995 | 0.003232733 |
| PXN | -0.019564748 | 0.001055605 | 0.003238774 |
| YPEL3 | 0.027126904 | 0.001063446 | 0.003260846 |
| SURF2 | -0.017445753 | 0.001081625 | 0.003314572 |
| CS | -0.021554198 | 0.001092816 | 0.003346831 |
| SLC22A3 | 0.01799828 | 0.001096186 | 0.003355115 |
| INTU | 0.018321148 | 0.001100256 | 0.00336553 |
| PSMG3 | -0.01459146 | 0.00110478 | 0.003377317 |
| POU3F3 | -0.040088455 | 0.001106647 | 0.003380974 |
| MPP7 | 0.017579117 | 0.001112774 | 0.003394622 |
| ENDOG | -0.023204564 | 0.001112894 | 0.003394622 |
| TXN2 | -0.030005481 | 0.001113134 | 0.003394622 |
| ATG3 | -0.016309852 | 0.001141737 | 0.003479743 |
| FDX1 | -0.023923576 | 0.0011473 | 0.003494587 |
| PRSS8 | -0.029517308 | 0.001155875 | 0.003518579 |
| PIGF | 0.015755653 | 0.001165647 | 0.003546183 |
| DVL1 | -0.0150196 | 0.001170708 | 0.003559434 |
| EIF3B | -0.017974764 | 0.001172221 | 0.003561885 |
| SRSF11 | 0.031237558 | 0.001173031 | 0.003562199 |
| REPIN1 | -0.01920338 | 0.001176275 | 0.003569899 |
| SEC62 | -0.040693531 | 0.001178953 | 0.003575874 |
| MCCC1 | 0.019420287 | 0.001180614 | 0.003577196 |
| ATP6V1H | 0.025145429 | 0.001181039 | 0.003577196 |
| SPINT2 | -0.042847301 | 0.001181518 | 0.003577196 |
| CAT | -0.030712881 | 0.001182558 | 0.003578195 |
| THOC7 | -0.020981137 | 0.001208031 | 0.003651458 |
| NCOA3 | 0.016646131 | 0.001208219 | 0.003651458 |
| DCTN1 | -0.01687367 | 0.001214894 | 0.003669431 |
| TMEM134 | -0.02185848 | 0.001217374 | 0.003673204 |
| ACAP2 | 0.018305742 | 0.001217601 | 0.003673204 |
| PTPN2 | 0.015230412 | 0.001255256 | 0.003784535 |
| NCKAP1 | 0.025589947 | 0.001270687 | 0.00382877 |
| SRP9 | -0.036732723 | 0.001275729 | 0.003841667 |
| COQ9 | -0.025182121 | 0.001278469 | 0.003847618 |
| TATDN1 | 0.01868105 | 0.001282706 | 0.003858067 |
| CMTM4 | -0.029580645 | 0.001298199 | 0.00390234 |
| COPS7A | -0.018830653 | 0.001301437 | 0.00390974 |
| UBE3C | 0.017329011 | 0.001312794 | 0.00394151 |
| RAD23A | -0.022467627 | 0.001315795 | 0.003946683 |
| WASF2 | -0.023553189 | 0.001316271 | 0.003946683 |
| TMX2 | -0.022111989 | 0.001316866 | 0.003946683 |
| SZRD1 | -0.016552154 | 0.001329173 | 0.003981201 |
| EP300 | 0.020083063 | 0.001348556 | 0.004036859 |
| ZFR | 0.022464912 | 0.001355951 | 0.004056586 |
| MLLT1 | -0.014254931 | 0.0013686 | 0.004091735 |
| SLC12A1 | 0.157776706 | 0.001369323 | 0.004091735 |
| CDC26 | -0.017841199 | 0.001372747 | 0.004099537 |

| | | | |
|---|---|---|---|
| PLAU | -0.05128961 | 0.001375964 | 0.004106251 |
| GSTO2 | 0.020632231 | 0.001376624 | 0.004106251 |
| TPP2 | 0.015068348 | 0.001387378 | 0.004135879 |
| PNN | -0.025930758 | 0.001413085 | 0.004210024 |
| LSM14A | 0.020800835 | 0.001419049 | 0.004225295 |
| ZNF706 | -0.024325875 | 0.001426273 | 0.004244298 |
| RAB3IP | 0.028310275 | 0.00144185 | 0.004288121 |
| GLRX | -0.034085254 | 0.00145166 | 0.004313549 |
| FDFT1 | 0.01616429 | 0.001452111 | 0.004313549 |
| DICER1 | 0.02574604 | 0.001471819 | 0.004369516 |
| SRSF8 | -0.018099208 | 0.001485169 | 0.004406554 |
| STX18 | 0.018535594 | 0.0015072 | 0.004469292 |
| MED21 | 0.014249682 | 0.001513231 | 0.004484537 |
| SRGAP3 | 0.015405464 | 0.001541681 | 0.004566167 |
| SPTBN1 | -0.037575775 | 0.001548799 | 0.004584554 |
| SMIM7 | -0.023636556 | 0.001549766 | 0.004584723 |
| DNAJC8 | -0.023779404 | 0.001552713 | 0.004590749 |
| ELOC | -0.023515771 | 0.001557385 | 0.004601863 |
| EEF2 | -0.045968534 | 0.00156027 | 0.004606445 |
| SPEF2 | 0.015311801 | 0.001560763 | 0.004606445 |
| ILF3-DT | -0.022394031 | 0.00156635 | 0.004618686 |
| S100A10 | 0.06284327 | 0.001566743 | 0.004618686 |
| ODC1 | -0.015738099 | 0.00157296 | 0.004633877 |
| TMEM38B | 0.024445552 | 0.001573735 | 0.004633877 |
| CITED2 | -0.021652096 | 0.001583909 | 0.004661112 |
| IFT122 | 0.013926411 | 0.001587291 | 0.004668341 |
| TMEM251 | -0.018587997 | 0.001608089 | 0.004726751 |
| DCAF5 | 0.015977386 | 0.001620657 | 0.004760915 |
| TCEAL4 | -0.028896468 | 0.001624654 | 0.004769877 |
| RPS20 | -0.035080875 | 0.00162639 | 0.004772196 |
| ETF1 | -0.015601579 | 0.001633729 | 0.004790942 |
| HNRNPDL | -0.036122468 | 0.001638418 | 0.004801897 |
| OPA1 | 0.018920315 | 0.001641167 | 0.004805503 |
| TMUB1 | -0.01621137 | 0.001641554 | 0.004805503 |
| CYFIP1 | -0.014932196 | 0.001649014 | 0.004824537 |
| MBD3 | -0.016319298 | 0.001654275 | 0.004836743 |
| FAM120AOS | -0.015516391 | 0.001655284 | 0.004836743 |
| CTSL | 0.032454407 | 0.001656064 | 0.004836743 |
| ACSS1 | -0.02862647 | 0.001662542 | 0.00485211 |
| SIK2 | 0.015723334 | 0.001663251 | 0.00485211 |
| HTT | 0.014164353 | 0.001666499 | 0.004858775 |
| NDUFS4 | 0.031877062 | 0.001686098 | 0.004913075 |
| FUCA1 | -0.019748115 | 0.001692836 | 0.004929859 |
| ATF7IP2 | 0.016624646 | 0.001709667 | 0.004975998 |
| MPHOSPH8 | -0.021410858 | 0.001716521 | 0.004993064 |
| UACA | 0.014627722 | 0.0017429 | 0.005066873 |
| TALDO1 | 0.025735302 | 0.001749615 | 0.005083464 |

| | | | |
|---|---|---|---|
| RPARP-AS1 | -0.016369902 | 0.001751794 | 0.005084274 |
| TNRC6C | 0.014226003 | 0.001751911 | 0.005084274 |
| VPS35 | -0.021428621 | 0.001754518 | 0.005088911 |
| MRPS31 | 0.020833131 | 0.001760483 | 0.005103273 |
| IVD | -0.021306984 | 0.001763569 | 0.005109282 |
| ABHD17C | 0.01857337 | 0.001771524 | 0.005129381 |
| CDH1 | -0.021077606 | 0.001778088 | 0.005145432 |
| FASTKD2 | 0.016058525 | 0.001782328 | 0.005154743 |
| THAP7 | -0.014025132 | 0.001878251 | 0.005429049 |
| ARF3 | -0.019174239 | 0.001904216 | 0.005500947 |
| PDIA4 | -0.020727159 | 0.001911907 | 0.005520003 |
| GANAB | -0.020939397 | 0.001923102 | 0.005549144 |
| SNRNP70 | 0.026900229 | 0.001926407 | 0.005555503 |
| NSRP1 | 0.019031096 | 0.00193444 | 0.005575479 |
| DCAF10 | 0.01624728 | 0.001940924 | 0.005590971 |
| SLC39A13 | -0.019944376 | 0.001945068 | 0.005597195 |
| CD81 | -0.046570844 | 0.001945306 | 0.005597195 |
| RPLP0 | -0.048065316 | 0.001961178 | 0.005639645 |
| GATM | -0.016946263 | 0.001965077 | 0.005647634 |
| SSR4 | 0.042025452 | 0.001970452 | 0.005659858 |
| TMEM238 | -0.027549842 | 0.001972929 | 0.005660966 |
| DERA | 0.017010304 | 0.001973084 | 0.005660966 |
| GOLGA7 | -0.016979611 | 0.0019911 | 0.005709406 |
| SRRM1 | -0.027611701 | 0.002003425 | 0.00574148 |
| C5orf24 | -0.019373666 | 0.002013434 | 0.005766886 |
| PDE9A | 0.01887631 | 0.002032989 | 0.005819591 |
| KATNBL1 | 0.014107676 | 0.002035924 | 0.005824684 |
| IRS1 | 0.017836882 | 0.002047801 | 0.00585534 |
| PTTG1IP | -0.031285546 | 0.002063008 | 0.005895477 |
| C6orf226 | -0.013883685 | 0.002082159 | 0.005946835 |
| WLS | 0.030762984 | 0.00213233 | 0.006086679 |
| CHCHD10 | -0.061330389 | 0.002141689 | 0.006109933 |
| ACO2 | -0.028181582 | 0.002143678 | 0.00611215 |
| UBR1 | 0.014543504 | 0.002146097 | 0.006115587 |
| RRAGD | -0.029730716 | 0.002156686 | 0.00614229 |
| SLC35A1 | 0.013273848 | 0.002171503 | 0.006180998 |
| POLR2B | 0.017446465 | 0.002180393 | 0.0062028 |
| NFE2L1 | -0.028625803 | 0.002191884 | 0.006231973 |
| MRPL33 | -0.047670442 | 0.002193568 | 0.006233245 |
| TMEM161B | 0.015794088 | 0.002211566 | 0.006280848 |
| PLOD2 | 0.023078904 | 0.00221439 | 0.006285327 |
| SRRM2 | -0.033690189 | 0.002245308 | 0.006367145 |
| FZD6 | 0.015799212 | 0.002245742 | 0.006367145 |
| ARFGEF3 | 0.021094919 | 0.002252969 | 0.006384044 |
| TMEM127 | -0.017314667 | 0.002258015 | 0.006393291 |
| PDE8A | 0.013921838 | 0.002258769 | 0.006393291 |
| DBP | -0.01551343 | 0.002266688 | 0.006412107 |

| | | | |
|---|---|---|---|
| AGPAT3 | -0.017016696 | 0.002288949 | 0.006471448 |
| RPL10 | 0.087558707 | 0.00229786 | 0.006492999 |
| KLHL36 | -0.015087497 | 0.00231802 | 0.006546295 |
| CPTP | -0.016406022 | 0.002343968 | 0.006615869 |
| SNX10 | -0.020827195 | 0.00234891 | 0.006626109 |
| VAMP8 | -0.034218975 | 0.002354219 | 0.006637369 |
| AHI1 | 0.016274994 | 0.0023718 | 0.006683199 |
| TPM3 | -0.020385643 | 0.002394829 | 0.006744321 |
| FUNDC1 | -0.014548731 | 0.002408448 | 0.006778888 |
| FAM234A | -0.018185893 | 0.002417567 | 0.006800756 |
| COL6A1 | -0.031344006 | 0.002426138 | 0.006821059 |
| C12orf49 | -0.015580067 | 0.002461462 | 0.006916517 |
| PFKL | -0.029608019 | 0.002463917 | 0.006919558 |
| ATM | 0.017277753 | 0.00247947 | 0.00695936 |
| ACTN4 | -0.028435215 | 0.002498321 | 0.007008369 |
| MRPL50 | -0.01493308 | 0.00251736 | 0.007057849 |
| PAQR5 | 0.025412542 | 0.0025284 | 0.007084861 |
| ZBED5-AS1 | 0.01616803 | 0.00253239 | 0.007092098 |
| CGN | -0.01586289 | 0.002559288 | 0.007163449 |
| SVIP | -0.021615369 | 0.002571398 | 0.007193351 |
| KDM2A | 0.016355028 | 0.002601523 | 0.007272215 |
| IMMP1L | 0.019714083 | 0.002602824 | 0.007272215 |
| KRIT1 | 0.015992935 | 0.002603917 | 0.007272215 |
| RHOBTB3 | -0.036483805 | 0.002610593 | 0.00728682 |
| LINC01578 | -0.028385687 | 0.002622029 | 0.007314693 |
| PILRB | 0.017164138 | 0.002628721 | 0.007329304 |
| RPL7L1 | -0.019262085 | 0.002633942 | 0.007339802 |
| TXNIP | 0.047506186 | 0.002653829 | 0.007391134 |
| TUBA1C | -0.01541922 | 0.002668553 | 0.007428038 |
| KAT5 | -0.013988542 | 0.002674114 | 0.007436977 |
| EIF3I | -0.020202886 | 0.002674715 | 0.007436977 |
| YBEY | 0.01362758 | 0.002687636 | 0.007468783 |
| MPRIP | -0.020651246 | 0.002746346 | 0.00762773 |
| UNG | -0.013816169 | 0.002751363 | 0.007637456 |
| CD47 | 0.021556231 | 0.002753227 | 0.007638425 |
| SSRP1 | -0.013616822 | 0.002761319 | 0.007656661 |
| FOXJ3 | 0.014864087 | 0.002771206 | 0.007679851 |
| RACK1 | 0.053540878 | 0.002787175 | 0.007719863 |
| ATRX | 0.029107673 | 0.002793437 | 0.007732958 |
| MACO1 | 0.01342415 | 0.002799518 | 0.007745539 |
| RAB5C | -0.022353773 | 0.002826024 | 0.007814584 |
| MRRF | 0.013843324 | 0.002845645 | 0.007864526 |
| SMAD5 | 0.019958867 | 0.002859087 | 0.007897347 |
| LDHB | 0.069554474 | 0.002864952 | 0.007909213 |
| PCK1 | -0.033808898 | 0.002887571 | 0.007967294 |
| PMM1 | 0.025074714 | 0.002893244 | 0.007978579 |
| ATP5F1B | -0.053765179 | 0.002899992 | 0.007992817 |

| | | | |
|---|---|---|---|
| PARK7 | -0.03816654 | 0.002904148 | 0.007999896 |
| SNRPF | -0.024253893 | 0.002908822 | 0.008008396 |
| MOK | 0.014435559 | 0.002930106 | 0.008062589 |
| SLC25A13 | 0.015625064 | 0.002933652 | 0.008067942 |
| BEX2 | -0.031416562 | 0.002938845 | 0.008077818 |
| NOP10 | -0.025921868 | 0.00295705 | 0.008123428 |
| BLOC1S6 | -0.013830244 | 0.002972571 | 0.008161618 |
| ABHD17A | -0.016916542 | 0.00297737 | 0.008170344 |
| TFDP2 | 0.024942922 | 0.002981876 | 0.008178257 |
| DDX18 | -0.019213873 | 0.002990508 | 0.008197471 |
| SDHA | -0.029386735 | 0.003023591 | 0.008283654 |
| SLC39A10 | 0.015722487 | 0.003033546 | 0.008306412 |
| BRD2 | -0.023138511 | 0.003037797 | 0.008313537 |
| PIAS2 | 0.021409428 | 0.003044451 | 0.008327226 |
| STX7 | 0.018197334 | 0.00306329 | 0.008374212 |
| BEX5 | -0.014586357 | 0.003072889 | 0.008395899 |
| GABARAPL1 | -0.033149811 | 0.003081778 | 0.008415624 |
| SSR3 | -0.027437577 | 0.003095727 | 0.008449138 |
| DNAJA2 | -0.018528801 | 0.003099486 | 0.008454821 |
| RPL18A | 0.060585798 | 0.003102782 | 0.008459233 |
| UBE2H | 0.024078776 | 0.003105981 | 0.00846338 |
| RPS13 | -0.065520677 | 0.003125168 | 0.00851106 |
| GOLIM4 | -0.02218501 | 0.003151207 | 0.008577341 |
| CHCHD1 | -0.02342843 | 0.003152931 | 0.008577403 |
| FABP3 | 0.038075876 | 0.003159941 | 0.008591834 |
| PTPN3 | 0.020405796 | 0.003168598 | 0.00861073 |
| DUS1L | -0.015026172 | 0.003189095 | 0.00866176 |
| GLYR1 | -0.019507482 | 0.003197452 | 0.008679783 |
| MYL12A | -0.028054729 | 0.003199549 | 0.008680799 |
| EFR3A | 0.01706071 | 0.00320645 | 0.008694844 |
| PLEKHA6 | 0.015298718 | 0.003228229 | 0.008747385 |
| SYNRG | 0.014587545 | 0.003229296 | 0.008747385 |
| TASOR | 0.017945862 | 0.0032369 | 0.008763272 |
| SNRPD2 | 0.026815415 | 0.003294904 | 0.00891552 |
| ATG5 | 0.014189048 | 0.003315464 | 0.008966337 |
| CLTB | -0.018567854 | 0.003319489 | 0.00897241 |
| NRDC | 0.015876202 | 0.003330794 | 0.008998142 |
| TFEB | -0.01444811 | 0.003335887 | 0.009007073 |
| RPS24 | 0.078153077 | 0.003352242 | 0.009046388 |
| ALDH4A1 | -0.015397949 | 0.003387079 | 0.009135507 |
| MSN | -0.016204862 | 0.00342374 | 0.009226029 |
| AQR | 0.01502712 | 0.003424301 | 0.009226029 |
| SCARB2 | -0.020571993 | 0.003432093 | 0.009242084 |
| KMT5B | 0.017985014 | 0.003436158 | 0.009248089 |
| SNRPD1 | -0.019504746 | 0.003442719 | 0.00925957 |
| SUCLA2 | 0.022139088 | 0.003444097 | 0.00925957 |
| ARID4A | 0.013992054 | 0.003454438 | 0.00928242 |

| | | | |
|---|---|---|---|
| SPG7 | -0.018035437 | 0.00348927 | 0.009371023 |
| FAM32A | -0.014946795 | 0.003492459 | 0.009374593 |
| GAL3ST1 | -0.015077545 | 0.003535239 | 0.009484376 |
| NDUFB6 | -0.02855992 | 0.003585073 | 0.009612853 |
| SYPL1 | 0.023767924 | 0.003586942 | 0.009612853 |
| GPBP1 | 0.026827976 | 0.003589791 | 0.009615375 |
| ATP6V0D1 | -0.019845645 | 0.003595035 | 0.009624309 |
| HSPA9 | -0.029615883 | 0.003624849 | 0.009698972 |
| ITGB1 | -0.025518264 | 0.00364492 | 0.009747501 |
| TMEM8A | -0.019022906 | 0.003654738 | 0.009768577 |
| DZIP3 | 0.015411047 | 0.00366177 | 0.009782185 |
| FRMD4A | 0.018567833 | 0.003668476 | 0.00979491 |
| UBTF | -0.018302386 | 0.003679034 | 0.009817899 |
| FLOT2 | -0.013249638 | 0.003702832 | 0.009876177 |
| VPS13D | 0.018356194 | 0.003732012 | 0.009948742 |
| FUBP1 | 0.018089238 | 0.003743302 | 0.009973566 |
| TNS2 | -0.01321701 | 0.00376028 | 0.010013509 |
| TSN | -0.015347571 | 0.00381988 | 0.010166851 |
| AMMECR1 | 0.014077438 | 0.003828703 | 0.010181041 |
| COLCA1 | -0.016603951 | 0.003829251 | 0.010181041 |
| RSL24D1 | 0.024500866 | 0.003837108 | 0.010196552 |
| CUL1 | 0.013852782 | 0.003850346 | 0.01022298 |
| ARHGAP21 | 0.016460892 | 0.003851109 | 0.01022298 |
| SRSF4 | 0.018064169 | 0.00385611 | 0.01023087 |
| HINT1 | -0.048514481 | 0.003868 | 0.010257015 |
| ZNF326 | 0.016583634 | 0.003881756 | 0.010288082 |
| RNF19A | 0.014337667 | 0.003891668 | 0.010308932 |
| CD9 | 0.042520931 | 0.003899155 | 0.010318717 |
| EPCAM | 0.038136344 | 0.003901284 | 0.010318717 |
| ZMIZ2 | -0.012748204 | 0.003902927 | 0.010318717 |
| ZBED5 | 0.014793649 | 0.003904034 | 0.010318717 |
| GARS-DT | 0.013867662 | 0.003905596 | 0.010318717 |
| RPL36 | 0.066669491 | 0.003916163 | 0.010341213 |
| MCUR1 | -0.016641577 | 0.003935252 | 0.010386182 |
| PPP1R13B | 0.01268898 | 0.003938336 | 0.010388881 |
| NDUFAF3 | -0.026529718 | 0.003943765 | 0.010397762 |
| TRAM1 | -0.026354323 | 0.003959248 | 0.010433124 |
| MRPL49 | -0.014457278 | 0.003975774 | 0.010471199 |
| CCNG2 | 0.021395925 | 0.003987316 | 0.010493134 |
| ZBTB8OS | 0.014119291 | 0.003988265 | 0.010493134 |
| ERGIC1 | -0.015972996 | 0.003996324 | 0.010508852 |
| CCDC30 | 0.013862583 | 0.004000258 | 0.010513714 |
| MFSD14A | 0.013159972 | 0.004019284 | 0.010558214 |
| DYNLL2 | -0.019912544 | 0.004042974 | 0.010614914 |
| VPS4A | -0.013364043 | 0.004059209 | 0.010651989 |
| HSPB1 | 0.035795571 | 0.004137776 | 0.010852512 |
| GATAD1 | -0.016199664 | 0.004161582 | 0.010909274 |

| | | | |
|---|---|---|---|
| MAP3K4 | 0.012658857 | 0.004166173 | 0.010915634 |
| UBN2 | 0.014814993 | 0.004171682 | 0.01092439 |
| JPX | 0.02250974 | 0.004186759 | 0.01095818 |
| CLCNKA | 0.039167712 | 0.00420492 | 0.011 |
| TRIM33 | 0.016594381 | 0.004207291 | 0.011000494 |
| SP3 | 0.014734272 | 0.004234674 | 0.01106635 |
| SYAP1 | -0.018747732 | 0.004243658 | 0.011084084 |
| TCAF1 | 0.013225827 | 0.004246957 | 0.011086954 |
| ARHGAP18 | 0.024200982 | 0.004255728 | 0.011104101 |
| HECTD4 | 0.013545142 | 0.004261543 | 0.011112122 |
| BRK1 | -0.023619379 | 0.00426321 | 0.011112122 |
| SUPT20H | 0.014803408 | 0.004281088 | 0.011152954 |
| PGK1 | -0.033393065 | 0.004367211 | 0.011371441 |
| SEMA6D | 0.034088506 | 0.00438121 | 0.011400449 |
| METTL23 | -0.014853117 | 0.004382874 | 0.011400449 |
| MFAP1 | -0.013682438 | 0.004431773 | 0.011521695 |
| DEK | 0.02111635 | 0.004457328 | 0.011582161 |
| NME1 | -0.017324935 | 0.004477965 | 0.011624294 |
| HSPH1 | -0.01553773 | 0.004479352 | 0.011624294 |
| POLR2I | 0.026639081 | 0.004480461 | 0.011624294 |
| MRPS23 | -0.016761341 | 0.004504289 | 0.011680102 |
| SCP2 | -0.031397364 | 0.004509781 | 0.011688331 |
| COL4A1 | 0.020103658 | 0.00453487 | 0.011747318 |
| FAM199X | -0.014149243 | 0.004544105 | 0.011765193 |
| BOLA2B | -0.013208641 | 0.00455317 | 0.011776946 |
| SARS | 0.01670619 | 0.004553316 | 0.011776946 |
| RAP1GAP2 | 0.013444019 | 0.004566736 | 0.011805598 |
| G3BP2 | -0.017546091 | 0.004614742 | 0.011923585 |
| MYSM1 | 0.016354929 | 0.004627777 | 0.011951139 |
| CHCHD2 | -0.048416186 | 0.004632211 | 0.011956465 |
| ZNF207 | 0.023234297 | 0.004636222 | 0.011960693 |
| ABCA2 | -0.019523449 | 0.004662959 | 0.012023516 |
| USP14 | 0.016705197 | 0.004701791 | 0.012117449 |
| MYLK | 0.018288146 | 0.004711766 | 0.01213091 |
| GPBP1L1 | 0.019314932 | 0.004711827 | 0.01213091 |
| CCDC90B | -0.012989435 | 0.004730121 | 0.012171792 |
| MRPL12 | -0.023965605 | 0.004773934 | 0.012278266 |
| HBP1 | 0.01557606 | 0.004828271 | 0.012411686 |
| WSB2 | -0.013639619 | 0.004834518 | 0.01242141 |
| RPN1 | -0.024119714 | 0.004837 | 0.012421454 |
| ISCA1 | -0.020332756 | 0.004845193 | 0.012436161 |
| ANKRD10 | 0.018372215 | 0.004866837 | 0.012485356 |
| ARHGAP8 | 0.015446799 | 0.004875419 | 0.012494315 |
| APC | 0.015507842 | 0.00487615 | 0.012494315 |
| LTN1 | 0.016027817 | 0.004877765 | 0.012494315 |
| UROS | -0.015351941 | 0.004883208 | 0.012501905 |
| RPL35A | 0.061874163 | 0.004894769 | 0.012525142 |

| | | | |
|---|---|---|---|
| MANF | -0.021243283 | 0.004926583 | 0.012600154 |
| UQCRB | -0.062315879 | 0.004953504 | 0.012662582 |
| AURKAIP1 | -0.034960728 | 0.00496113 | 0.012675649 |
| COPS9 | -0.033960623 | 0.004991573 | 0.01274697 |
| CHD3 | -0.01429613 | 0.005002019 | 0.012767179 |
| SELENOP | 0.028524159 | 0.005033569 | 0.012841205 |
| PRPS2 | -0.013152729 | 0.005096527 | 0.012995243 |
| APH1A | -0.019978444 | 0.005109071 | 0.013020641 |
| WDR48 | 0.013863363 | 0.00512373 | 0.013051402 |
| WNK1 | 0.05241047 | 0.005151328 | 0.013111473 |
| TMEM61 | -0.017143991 | 0.005152515 | 0.013111473 |
| TMEM9 | -0.02022453 | 0.005197175 | 0.013218447 |
| DAZAP2 | -0.028631266 | 0.005274014 | 0.013407113 |
| UBE2V2 | -0.014002964 | 0.005304171 | 0.013476979 |
| RNF43 | 0.015823871 | 0.00531084 | 0.013486171 |
| TMEM168 | 0.01322904 | 0.005313139 | 0.013486171 |
| EVA1B | -0.017141024 | 0.005331129 | 0.013525022 |
| COPS2 | 0.015342272 | 0.005337816 | 0.013535177 |
| KCNJ1 | -0.043930792 | 0.00534849 | 0.013555423 |
| HINT3 | -0.012549334 | 0.005365267 | 0.01359111 |
| TXNDC17 | -0.024633513 | 0.005408064 | 0.013692642 |
| NACA | -0.053602019 | 0.005412843 | 0.013697862 |
| ZNF277 | 0.012839029 | 0.00543082 | 0.013736459 |
| NUDT5 | 0.015203439 | 0.005441548 | 0.013756691 |
| SNX3 | -0.025706992 | 0.005499046 | 0.013892326 |
| PLEKHJ1 | -0.023571207 | 0.005500711 | 0.013892326 |
| RIMKLB | 0.013313536 | 0.00551203 | 0.013913944 |
| CWC22 | 0.012252333 | 0.00553493 | 0.013964755 |
| BCAT2 | 0.012451203 | 0.005539921 | 0.013969048 |
| CCT5 | -0.017022596 | 0.005542173 | 0.013969048 |
| HNRNPM | 0.024751139 | 0.005585357 | 0.014070856 |
| NUDCD2 | -0.015095176 | 0.005610628 | 0.01412746 |
| ATXN3 | 0.012153472 | 0.005620696 | 0.014145745 |
| ANKLE2 | -0.012134937 | 0.005631328 | 0.014165432 |
| TMED7 | -0.021489993 | 0.005668494 | 0.01425181 |
| DCUN1D4 | 0.013485143 | 0.005730774 | 0.014401213 |
| AKR1B1 | -0.014458963 | 0.005794561 | 0.014554251 |
| ITFG2 | 0.012670109 | 0.005797822 | 0.01455519 |
| SUCNR1 | 0.032109388 | 0.005802733 | 0.014560268 |
| PITPNA | -0.013117412 | 0.005812063 | 0.014576422 |
| TRAK2 | 0.016148454 | 0.005826535 | 0.014605452 |
| NAP1L4 | -0.020786573 | 0.00588023 | 0.014732724 |
| PLEKHH2 | 0.02067709 | 0.005902353 | 0.014780805 |
| TAPT1 | 0.01357423 | 0.005906015 | 0.014782632 |
| ATAD1 | 0.018908588 | 0.005954878 | 0.01489754 |
| NDUFB10 | -0.04123006 | 0.005962605 | 0.01490947 |
| ZHX1 | -0.013650146 | 0.006001176 | 0.014998478 |

| | | | |
|---|---|---|---|
| ABI3 | -0.014991055 | 0.006009339 | 0.015011437 |
| PLSCR1 | 0.013414 | 0.00602407 | 0.015040781 |
| DHX15 | 0.01912973 | 0.006061174 | 0.015125931 |
| KRCC1 | 0.012941652 | 0.00606627 | 0.015131156 |
| OCIAD2 | -0.019543453 | 0.006092925 | 0.015190127 |
| RIF1 | 0.016623414 | 0.006120835 | 0.015252164 |
| KIAA0355 | 0.012140674 | 0.006123999 | 0.015252511 |
| CSTF3 | 0.013063074 | 0.006128322 | 0.015255738 |
| YWHAH | -0.015730078 | 0.006194443 | 0.015412728 |
| COA8 | -0.01486365 | 0.006208827 | 0.015440897 |
| MBD6 | 0.012909778 | 0.006221378 | 0.015464481 |
| GLTP | -0.021971748 | 0.006235839 | 0.015485407 |
| MAPK8IP3 | 0.012011171 | 0.006235941 | 0.015485407 |
| MICU3 | 0.014213105 | 0.00628167 | 0.015591285 |
| GRPEL1 | -0.014277864 | 0.006326509 | 0.015690899 |
| STX17 | 0.01354872 | 0.00632803 | 0.015690899 |
| MRPL57 | -0.027444453 | 0.006394629 | 0.015848243 |
| SELENOT | -0.020621507 | 0.006486629 | 0.016068353 |
| EZR | -0.025384434 | 0.0065398 | 0.016192109 |
| MYH10 | -0.018074418 | 0.006556319 | 0.016225038 |
| NPHP3 | 0.014504222 | 0.006579215 | 0.016272553 |
| USF2 | -0.014781015 | 0.006581975 | 0.016272553 |
| ANKRD13A | -0.01285068 | 0.006635553 | 0.016396972 |
| TOGARAM1 | 0.012926269 | 0.006647645 | 0.016418804 |
| CEBPZOS | 0.017745498 | 0.006676711 | 0.016482516 |
| NR1H2 | -0.013013829 | 0.006690619 | 0.016508766 |
| SRPRB | -0.013553609 | 0.006715998 | 0.016563281 |
| ABCC5 | 0.017188552 | 0.006726321 | 0.016580629 |
| FAR1 | 0.014253295 | 0.006758337 | 0.016651407 |
| HTATSF1 | -0.015026119 | 0.006803969 | 0.016755647 |
| HOXB3 | 0.02114993 | 0.006808573 | 0.016758796 |
| LINC00467 | 0.014991545 | 0.006825248 | 0.016791642 |
| EPHA4 | 0.017079979 | 0.006848514 | 0.016840662 |
| GALNT14 | 0.017574469 | 0.006956291 | 0.01709735 |
| LYPLA1 | -0.02089085 | 0.006981365 | 0.017150616 |
| USP40 | 0.014307645 | 0.006985123 | 0.017151488 |
| ATP6V1F | -0.030444617 | 0.006993441 | 0.017163552 |
| NDUFB7 | -0.045559798 | 0.007045892 | 0.017283863 |
| HDGF | -0.017553843 | 0.007062308 | 0.017315709 |
| MRPS35 | -0.018834051 | 0.007093474 | 0.017383667 |
| SPG21 | -0.015541817 | 0.007121031 | 0.017442719 |
| MRPL51 | -0.026726906 | 0.007137978 | 0.017475739 |
| GALK2 | 0.011950323 | 0.00719759 | 0.017613133 |
| MAP3K2 | 0.016353526 | 0.007235614 | 0.017697588 |
| RETREG2 | -0.018348625 | 0.007244379 | 0.017710033 |
| COA6 | -0.016107793 | 0.007247728 | 0.017710033 |
| CERS2 | -0.019037172 | 0.007267581 | 0.017749941 |

| | | | |
|---|---|---|---|
| POP5 | -0.01658076 | 0.00737665 | 0.018004218 |
| AKAP11 | 0.014617819 | 0.007381807 | 0.018004218 |
| PCCB | 0.012450683 | 0.007382408 | 0.018004218 |
| TNKS2 | 0.013657485 | 0.00740247 | 0.018044414 |
| DESI1 | -0.012661658 | 0.007425439 | 0.018091657 |
| RBX1 | -0.028969739 | 0.007474205 | 0.018201676 |
| RALGPS1 | 0.015421578 | 0.00756468 | 0.018413111 |
| HOXB7 | -0.014759359 | 0.007572602 | 0.018423497 |
| TMEM126B | -0.015131534 | 0.007576299 | 0.0184236 |
| MKKS | -0.012731985 | 0.0076074 | 0.01849031 |
| CLCN3 | 0.023785966 | 0.007630106 | 0.01853656 |
| HNRNPK | -0.033662492 | 0.007643235 | 0.018559512 |
| ETFRF1 | -0.017089762 | 0.007663089 | 0.01859511 |
| PITPNB | 0.012553494 | 0.007665273 | 0.01859511 |
| PRDX5 | -0.0447601 | 0.007704707 | 0.018681783 |
| HPCAL1 | -0.014868467 | 0.007728068 | 0.018729418 |
| PGD | -0.013979319 | 0.007880946 | 0.019090749 |
| SLC48A1 | -0.012880235 | 0.007905986 | 0.019142207 |
| UBA52 | 0.042830421 | 0.007940977 | 0.019217697 |
| ITGB8 | 0.015463765 | 0.007957666 | 0.019248847 |
| MRPS36 | -0.025682258 | 0.0080095 | 0.019364934 |
| APMAP | -0.014087873 | 0.008036566 | 0.01942106 |
| KIAA0319L | 0.016321533 | 0.008063907 | 0.019477793 |
| CIR1 | 0.015148538 | 0.008129794 | 0.019627534 |
| LRRFIP1 | -0.020966536 | 0.008172588 | 0.019721405 |
| FAM3C | -0.021048253 | 0.008183815 | 0.019739049 |
| ZMAT3 | -0.012087101 | 0.008230154 | 0.019841322 |
| LSM12 | -0.012746489 | 0.008247242 | 0.019873015 |
| AC244090.1 | -0.012904419 | 0.008308795 | 0.020011771 |
| USO1 | 0.018890798 | 0.008339994 | 0.020077322 |
| TSPAN6 | -0.022898878 | 0.008353966 | 0.020101358 |
| EXOSC6 | -0.012094372 | 0.008376133 | 0.020135739 |
| SSU72 | -0.021372171 | 0.008376244 | 0.020135739 |
| DIPK1B | -0.01315934 | 0.008406311 | 0.020198386 |
| M6PR | -0.017309543 | 0.008452178 | 0.020298919 |
| TOMM5 | -0.020962313 | 0.008491506 | 0.020379805 |
| STAG2 | 0.019224157 | 0.008493944 | 0.020379805 |
| XRCC6 | -0.021062307 | 0.00852494 | 0.020439746 |
| CDC73 | 0.014584547 | 0.008527035 | 0.020439746 |
| MRPS26 | -0.022215169 | 0.008535126 | 0.020449416 |
| GOLM1 | -0.028268442 | 0.008539731 | 0.020450729 |
| SEL1L | -0.017742811 | 0.008560187 | 0.020482205 |
| EPB41L4A-AS1 | 0.016569715 | 0.008561001 | 0.020482205 |
| RAPGEF1 | 0.012893627 | 0.008568767 | 0.02049106 |
| PPM1K | 0.02464502 | 0.008576535 | 0.020499911 |
| CREBRF | 0.019394441 | 0.008617765 | 0.020582467 |
| GBA2 | -0.016033215 | 0.00861924 | 0.020582467 |

| | | | |
|---|---|---|---|
| DNAJB12 | -0.011589684 | 0.008679602 | 0.020716797 |
| HNF1B | 0.017254104 | 0.008747553 | 0.020869103 |
| KIF12 | 0.028416731 | 0.008754921 | 0.0208768 |
| EIF3H | 0.023779526 | 0.008763103 | 0.020886432 |
| DPY19L4 | 0.014037573 | 0.008832907 | 0.021042857 |
| FMC1 | -0.027514974 | 0.008862117 | 0.021102472 |
| RPL34 | 0.071442764 | 0.008953802 | 0.021310726 |
| HNRNPA3 | -0.028098603 | 0.008981504 | 0.021358034 |
| SPTY2D1 | -0.012067215 | 0.008982153 | 0.021358034 |
| AP002360.1 | -0.016861658 | 0.008993119 | 0.021374027 |
| RBBP4 | 0.020224632 | 0.009015167 | 0.021416332 |
| TAOK1 | 0.016781434 | 0.009039284 | 0.021463509 |
| PFDN6 | 0.011943989 | 0.009077407 | 0.021543882 |
| APPBP2 | 0.012692635 | 0.009133162 | 0.021666009 |
| CAPN1 | -0.015023364 | 0.009171905 | 0.021747681 |
| MDH2 | -0.027086984 | 0.009199161 | 0.021802054 |
| MAN1A2 | 0.018538591 | 0.009208441 | 0.021813793 |
| AKT1 | -0.014112049 | 0.009234673 | 0.021865658 |
| IP6K1 | 0.012462023 | 0.009250805 | 0.021893573 |
| RMDN1 | 0.016548165 | 0.009259441 | 0.021903727 |
| CETN2 | -0.014878642 | 0.00929839 | 0.021985545 |
| FBXW5 | -0.016555258 | 0.009329875 | 0.022049649 |
| UQCR11 | -0.053808027 | 0.009393218 | 0.022188947 |
| TMEM184C | 0.011457713 | 0.009400751 | 0.022196339 |
| ZFX | 0.012218898 | 0.00945262 | 0.02230836 |
| SYNJ2BP | -0.019199041 | 0.009471498 | 0.022342454 |
| JADE1 | -0.015019581 | 0.009643794 | 0.022738244 |
| ALDH2 | -0.02376553 | 0.009649337 | 0.022740678 |
| WWP2 | 0.013158591 | 0.00970377 | 0.022858274 |
| PPP1CA | -0.017631414 | 0.009749928 | 0.022956277 |
| ROCK1 | 0.015166475 | 0.009786278 | 0.023031106 |
| CPSF6 | 0.015571599 | 0.009791955 | 0.023033711 |
| LSM10 | -0.01278503 | 0.00982096 | 0.023091166 |
| SGCB | -0.013468282 | 0.009874194 | 0.023205507 |
| CIT | 0.01269546 | 0.009886703 | 0.023224077 |
| CEPT1 | 0.012247422 | 0.009893321 | 0.023228798 |
| GALNT10 | 0.013243459 | 0.009947235 | 0.023344512 |
| C1orf115 | -0.012839833 | 0.009978122 | 0.023406103 |
| UBE2Q1 | -0.012851222 | 0.00998608 | 0.023413873 |
| PFN1 | -0.030169569 | 0.010022171 | 0.023487569 |
| BRI3BP | -0.013284903 | 0.010034339 | 0.02350516 |
| INPPL1 | -0.011723342 | 0.010071911 | 0.023582213 |
| CCDC12 | 0.015784461 | 0.010135924 | 0.023721074 |
| FAM162A | -0.028536557 | 0.010230162 | 0.023930508 |
| TUSC3 | 0.014357755 | 0.010239409 | 0.02394103 |
| TBRG1 | 0.012202723 | 0.010247577 | 0.023943793 |
| SORT1 | -0.020015198 | 0.01025009 | 0.023943793 |

| | | | |
|---|---|---|---|
| ESRRA | -0.018254445 | 0.010295429 | 0.024038562 |
| TM9SF3 | -0.026680768 | 0.01042825 | 0.02433741 |
| SPTLC2 | 0.015204085 | 0.010455219 | 0.024389061 |
| SNRPE | -0.023843506 | 0.010471771 | 0.024416374 |
| RAB3GAP2 | 0.014279678 | 0.010486839 | 0.024429997 |
| HMGN3 | -0.032756779 | 0.010487307 | 0.024429997 |
| KIAA2013 | -0.011400289 | 0.010502047 | 0.024453034 |
| TCERG1 | 0.013002671 | 0.010657113 | 0.024802635 |
| TSR2 | -0.012871208 | 0.010686361 | 0.024859227 |
| FAM133B | 0.014862019 | 0.010723295 | 0.02493364 |
| VPS13C | 0.019860482 | 0.010751611 | 0.024987954 |
| CLPTM1 | -0.017894431 | 0.010778023 | 0.025037795 |
| CHD4 | -0.019629634 | 0.010788289 | 0.0250501 |
| PSMB8 | -0.011860136 | 0.010818875 | 0.025109553 |
| BROX | 0.014482739 | 0.01092647 | 0.025333733 |
| PRKX | -0.015384033 | 0.010929095 | 0.025333733 |
| SNX17 | -0.014224759 | 0.010930544 | 0.025333733 |
| HNRNPAB | -0.012386567 | 0.010963171 | 0.02539703 |
| GMCL1 | -0.012945723 | 0.01096793 | 0.02539703 |
| TUBB | -0.023021471 | 0.010984491 | 0.0254237 |
| VPS39 | 0.010850017 | 0.011052352 | 0.025569025 |
| ZBTB38 | -0.015301607 | 0.011137995 | 0.025755336 |
| RPL10A | 0.046895532 | 0.011146414 | 0.025762987 |
| PDS5A | 0.013361067 | 0.011180391 | 0.025829675 |
| CD74 | -0.022337679 | 0.011188202 | 0.02583588 |
| CFI | 0.013664029 | 0.011286247 | 0.026050353 |
| ZBTB44 | 0.020420608 | 0.011301578 | 0.026073802 |
| COPS4 | 0.012272057 | 0.011392021 | 0.026270438 |
| FGD5-AS1 | -0.022080896 | 0.011561897 | 0.026649989 |
| CALR | -0.040960084 | 0.011608417 | 0.026744986 |
| HOXB-AS3 | -0.017889361 | 0.011702549 | 0.026949544 |
| COPS6 | 0.017698232 | 0.011735574 | 0.027013255 |
| ATP2A2 | -0.018421672 | 0.011744656 | 0.027021821 |
| TMBIM4 | 0.029680005 | 0.011832756 | 0.02720738 |
| FCHO2 | 0.012466813 | 0.011836101 | 0.02720738 |
| APOOL | 0.014451616 | 0.011856664 | 0.027242226 |
| TMED10 | -0.023767492 | 0.011881024 | 0.027285759 |
| ARL16 | -0.013910044 | 0.011982787 | 0.027506936 |
| NUDT7 | -0.011863541 | 0.012124961 | 0.027820632 |
| KDM3B | 0.012262559 | 0.012158297 | 0.027884428 |
| MRPS18A | -0.011274096 | 0.012234348 | 0.028042796 |
| CSRP2 | 0.024414498 | 0.012238475 | 0.028042796 |
| NOVA1 | 0.013239028 | 0.012321895 | 0.028221113 |
| CASD1 | 0.011388107 | 0.012329495 | 0.028225696 |
| ZNHIT3 | -0.013051923 | 0.012359794 | 0.028282216 |
| OSBPL1A | 0.016908783 | 0.012392949 | 0.028345217 |
| GATD3B | -0.013363008 | 0.012410071 | 0.028371504 |

| | | | |
|---|---|---|---|
| POLR3GL | -0.011958547 | 0.012421714 | 0.028384111 |
| EIF4A3 | -0.012732289 | 0.012426847 | 0.028384111 |
| SUB1 | -0.0345822 | 0.012450645 | 0.028425589 |
| MZT2A | -0.019273156 | 0.012471084 | 0.028459363 |
| FYTTD1 | -0.012596485 | 0.01251387 | 0.02854408 |
| TASOR2 | 0.012442963 | 0.012592424 | 0.028710272 |
| ING4 | 0.011258643 | 0.012736449 | 0.029018026 |
| SF3B1 | 0.02426134 | 0.012738919 | 0.029018026 |
| ABCA5 | 0.017538116 | 0.012838576 | 0.029231826 |
| ITSN2 | 0.012056263 | 0.012883879 | 0.029321731 |
| TAF1C | -0.010913098 | 0.012909877 | 0.029367641 |
| BSG | -0.045887223 | 0.012970801 | 0.02948813 |
| IL6ST | 0.019002374 | 0.012974543 | 0.02948813 |
| STRN | 0.013340495 | 0.013029254 | 0.029599129 |
| TOLLIP | -0.015926023 | 0.013040395 | 0.029611095 |
| NUP153 | 0.011317312 | 0.013068303 | 0.029661106 |
| HLA-E | -0.015152185 | 0.013093861 | 0.02970574 |
| NTPCR | -0.014044793 | 0.013129223 | 0.029772566 |
| SRP54 | 0.014129449 | 0.013149049 | 0.029804117 |
| CLK4 | 0.016087388 | 0.013165071 | 0.029827021 |
| C14orf119 | -0.012255599 | 0.013193317 | 0.029877589 |
| GPX3 | -0.020884181 | 0.013212137 | 0.029906772 |
| OTUD6B-AS1 | -0.018616618 | 0.013250343 | 0.029979793 |
| ACIN1 | 0.015208304 | 0.013291624 | 0.030059703 |
| PANK3 | -0.013233507 | 0.013326709 | 0.030125534 |
| MRPS14 | -0.012759509 | 0.013394886 | 0.030266079 |
| YLPM1 | 0.013654458 | 0.013479616 | 0.030443882 |
| MRPL54 | -0.023019801 | 0.013565471 | 0.030624067 |
| STRAP | -0.018791449 | 0.0135939 | 0.030666592 |
| ERLEC1 | -0.013644074 | 0.013596476 | 0.030666592 |
| ABL1 | 0.012140104 | 0.013629625 | 0.030727611 |
| UBE4B | 0.010753064 | 0.013711183 | 0.030897664 |
| CLDN7 | -0.016573157 | 0.01376027 | 0.030989902 |
| HOXD9 | -0.015068684 | 0.01376441 | 0.030989902 |
| ETFDH | 0.012699556 | 0.013773991 | 0.030991906 |
| MORF4L1 | -0.028727392 | 0.013777596 | 0.030991906 |
| ANXA11 | -0.028409017 | 0.013886483 | 0.031215849 |
| SPSB3 | 0.017131223 | 0.013889536 | 0.031215849 |
| STIP1 | -0.012247224 | 0.014033058 | 0.031524352 |
| TMPRSS2 | -0.013411176 | 0.014058393 | 0.031567197 |
| RPLP1 | 0.070670394 | 0.01410776 | 0.031663943 |
| SELENOF | -0.020904768 | 0.014119087 | 0.031675264 |
| ACP1 | -0.018490931 | 0.014127467 | 0.031679964 |
| VOPP1 | 0.011710875 | 0.014182463 | 0.031789149 |
| IFT80 | 0.012248371 | 0.014214792 | 0.031847452 |
| C21orf62 | -0.014260743 | 0.014248071 | 0.03190783 |
| DPP7 | 0.020304514 | 0.014256356 | 0.031912206 |

| | | | |
|---|---|---|---|
| RBM3 | -0.017579586 | 0.01434513 | 0.03209667 |
| PLEKHA3 | -0.010973283 | 0.014403234 | 0.032212379 |
| MRPL15 | -0.017766332 | 0.014472032 | 0.03235189 |
| TAF15 | 0.013130782 | 0.014521512 | 0.032448112 |
| KLF9 | -0.014998968 | 0.014578615 | 0.032561276 |
| RABL6 | -0.016095185 | 0.014707301 | 0.032834147 |
| EXOSC8 | 0.011783145 | 0.014717933 | 0.032843337 |
| LMBR1 | 0.01189787 | 0.014754805 | 0.032911051 |
| PTAR1 | 0.015208145 | 0.014780913 | 0.032954703 |
| DDIT3 | -0.015016039 | 0.014900356 | 0.03320632 |
| HOXB8 | -0.013495076 | 0.014915592 | 0.033225584 |
| TCF25 | -0.019259392 | 0.015050191 | 0.033510606 |
| STUB1 | -0.021405057 | 0.015090534 | 0.033585599 |
| GMDS | 0.02043953 | 0.015104228 | 0.033601242 |
| CTSO | 0.016677407 | 0.015121512 | 0.033624854 |
| SS18 | 0.015404679 | 0.015133381 | 0.033636409 |
| IFI30 | 0.024210972 | 0.015154116 | 0.03366765 |
| SMDT1 | -0.030899475 | 0.015192577 | 0.033738228 |
| DMAC2L | 0.011770142 | 0.015237975 | 0.033824145 |
| ADIPOR1 | -0.013560509 | 0.015347742 | 0.034052802 |
| SIGIRR | -0.013182781 | 0.015359232 | 0.034063303 |
| PRDM16 | 0.014051347 | 0.01538623 | 0.034108173 |
| HSP90AB1 | -0.035797294 | 0.01543858 | 0.03420918 |
| MRPS16 | -0.016947011 | 0.015460232 | 0.034242105 |
| ANP32B | -0.018054714 | 0.015470068 | 0.034248842 |
| CAPNS1 | -0.022027959 | 0.015507518 | 0.034316681 |
| EBLN3P | -0.015158838 | 0.015522378 | 0.034334492 |
| ARL2BP | -0.011038438 | 0.015584397 | 0.034456555 |
| POMGNT1 | -0.010937929 | 0.015603851 | 0.034484443 |
| PCYOX1 | -0.019996078 | 0.015735821 | 0.034746934 |
| CDK16 | -0.015038275 | 0.015736412 | 0.034746934 |
| ZNF467 | -0.011958187 | 0.015767923 | 0.034801269 |
| SYVN1 | -0.0116561 | 0.015780944 | 0.034814764 |
| PSMG2 | 0.015913915 | 0.0158852 | 0.035027338 |
| SMC1A | -0.011805593 | 0.015891196 | 0.035027338 |
| DDX52 | 0.011286288 | 0.015911236 | 0.03505618 |
| COMMD8 | -0.012513559 | 0.016024585 | 0.03529049 |
| KRTCAP2 | 0.024846048 | 0.016155387 | 0.035563017 |
| ZNF580 | -0.014541362 | 0.016189064 | 0.035621595 |
| RING1 | 0.011415172 | 0.016212999 | 0.035658695 |
| LINC01089 | 0.015293619 | 0.016241837 | 0.035706543 |
| PPP4R3B | 0.015453718 | 0.016268667 | 0.035749935 |
| IARS | -0.012575405 | 0.016291056 | 0.035783536 |
| SPART | 0.01472152 | 0.016320326 | 0.035832215 |
| TMEM120A | 0.01100942 | 0.016419811 | 0.036034945 |
| DYNLT1 | -0.021659542 | 0.016514854 | 0.036216385 |
| RPS17 | -0.032024079 | 0.016516856 | 0.036216385 |

| | | | |
|---|---|---|---|
| HPN | 0.024451242 | 0.016526534 | 0.036221852 |
| FNBP4 | 0.014105231 | 0.016617661 | 0.036405748 |
| CYP7B1 | 0.014535322 | 0.016632306 | 0.036417574 |
| FNTA | 0.012374304 | 0.016637507 | 0.036417574 |
| BTF3 | 0.032598136 | 0.016686346 | 0.036495745 |
| PRR13 | -0.030056669 | 0.016687699 | 0.036495745 |
| ERG28 | -0.011608257 | 0.016849255 | 0.036833085 |
| ZNF254 | 0.0109135 | 0.01690285 | 0.036934229 |
| ARMCX3 | -0.015656326 | 0.016935739 | 0.03699006 |
| RSL1D1 | -0.015511416 | 0.017078857 | 0.037286496 |
| SNHG7 | 0.02242678 | 0.017098119 | 0.037312389 |
| CHMP2B | -0.012398192 | 0.017139252 | 0.037370641 |
| GSE1 | -0.011994633 | 0.017144496 | 0.037370641 |
| BZW2 | 0.014157195 | 0.017147053 | 0.037370641 |
| TOMM22 | -0.016063577 | 0.017172635 | 0.037410222 |
| IGBP1 | 0.013345128 | 0.017357339 | 0.037796261 |
| ZNF667-AS1 | -0.014682058 | 0.017388318 | 0.03784737 |
| GNS | -0.018380224 | 0.017444138 | 0.037952482 |
| PWWP3A | 0.013523914 | 0.017563895 | 0.038196546 |
| MMP24OS | -0.023580893 | 0.017646866 | 0.038360437 |
| TIMM17A | -0.01109508 | 0.017710709 | 0.038482622 |
| ZFAS1 | 0.021170676 | 0.017733108 | 0.038514691 |
| NDRG2 | -0.028587633 | 0.017776864 | 0.038593097 |
| TNFRSF21 | -0.011582489 | 0.017800808 | 0.038628442 |
| CDK12 | 0.0121373 | 0.017812778 | 0.038637785 |
| SAV1 | -0.011895834 | 0.017916564 | 0.038846193 |
| ANP32A | -0.014737598 | 0.018139663 | 0.039313002 |
| TDRP | 0.012289599 | 0.018177622 | 0.039378338 |
| ECHDC1 | 0.015936077 | 0.018201869 | 0.039413928 |
| COMMD9 | -0.012911265 | 0.018219636 | 0.039420816 |
| MORN2 | -0.016157586 | 0.018220691 | 0.039420816 |
| TCP1 | -0.01614501 | 0.018243868 | 0.039454029 |
| CDC42 | -0.02471607 | 0.018256751 | 0.039464959 |
| UBE2I | -0.012925732 | 0.018354175 | 0.039650579 |
| UBC | 0.031161352 | 0.018358352 | 0.039650579 |
| RABAC1 | 0.024989105 | 0.018542712 | 0.040031612 |
| FBXO34 | 0.010821893 | 0.018724939 | 0.040407712 |
| CREBBP | 0.01374462 | 0.018794117 | 0.040539641 |
| ARL8A | -0.010279897 | 0.018832627 | 0.040590266 |
| HIPK2 | -0.02768851 | 0.01883369 | 0.040590266 |
| SYT13 | -0.012877923 | 0.018843473 | 0.040593995 |
| ICE2 | 0.013088851 | 0.018974103 | 0.040857948 |
| PRDX3 | -0.032261492 | 0.01902324 | 0.040946265 |
| CD151 | -0.019782322 | 0.019112677 | 0.041110285 |
| BPTF | 0.020658489 | 0.019115752 | 0.041110285 |
| MRPS18B | -0.018780138 | 0.019244277 | 0.041369041 |
| YTHDC2 | 0.011650354 | 0.019344219 | 0.04156616 |

| | | | |
|---|---|---|---|
| C16orf89 | -0.023412941 | 0.019378743 | 0.0416226 |
| OGA | 0.019708209 | 0.019503395 | 0.041872492 |
| YWHAG | -0.012504589 | 0.019568497 | 0.041994378 |
| TRAPPC12 | 0.011132637 | 0.019627906 | 0.042103946 |
| EIF3G | 0.01909256 | 0.019684832 | 0.042208099 |
| FAM171A1 | 0.019564281 | 0.019793859 | 0.042423829 |
| EFCAB14 | -0.017873537 | 0.019814855 | 0.04245078 |
| SUMO1 | -0.024374711 | 0.019844145 | 0.042495469 |
| ABI1 | 0.013425669 | 0.019871517 | 0.042536017 |
| HMGCR | 0.012126175 | 0.019907769 | 0.042586583 |
| TCEA3 | 0.019893053 | 0.019912036 | 0.042586583 |
| TMEM9B | -0.01474322 | 0.019942054 | 0.042622663 |
| UBE2K | 0.016739693 | 0.019945817 | 0.042622663 |
| TSPAN9 | 0.015873392 | 0.019964306 | 0.042644096 |
| EIF3A | -0.020765125 | 0.019991887 | 0.042684924 |
| HCFC1R1 | -0.017739431 | 0.020180532 | 0.043069458 |
| PCNX4 | 0.014774754 | 0.020191145 | 0.043073873 |
| SLCO4C1 | 0.014829684 | 0.020325328 | 0.043341785 |
| PPP2R5E | 0.013332664 | 0.02047388 | 0.043640098 |
| RBM6 | 0.019335057 | 0.020570598 | 0.043827719 |
| CCS | 0.01333959 | 0.020587167 | 0.04384449 |
| KPNA4 | 0.013772533 | 0.020628163 | 0.043913248 |
| FLII | -0.01180722 | 0.020732663 | 0.044117077 |
| SLC6A8 | -0.016511564 | 0.020770463 | 0.044178862 |
| MRPL20 | -0.02536676 | 0.020828578 | 0.044283789 |
| PLEC | -0.012493755 | 0.020929601 | 0.044479814 |
| LINC00379 | 0.012833692 | 0.021007183 | 0.044625878 |
| CACUL1 | 0.010832058 | 0.021099416 | 0.04480293 |
| ZNF141 | 0.010190055 | 0.021230912 | 0.045057836 |
| EZH1 | 0.010742048 | 0.021237338 | 0.045057836 |
| RAB13 | -0.019538537 | 0.021270709 | 0.045109654 |
| SUSD4 | 0.015691153 | 0.02129192 | 0.045135647 |
| EEF1E1 | -0.016692691 | 0.021361832 | 0.045264816 |
| KDELR2 | -0.015057742 | 0.021494325 | 0.045526426 |
| PDPR | -0.010116348 | 0.021697051 | 0.045936511 |
| NIPSNAP3A | -0.010705621 | 0.021818515 | 0.046174279 |
| CAP1 | -0.014876237 | 0.021917432 | 0.046364152 |
| CEP290 | 0.012982573 | 0.021934288 | 0.046380346 |
| AFTPH | 0.017497797 | 0.022000728 | 0.046501329 |
| OS9 | -0.016000757 | 0.022173646 | 0.046847171 |
| DNAJC1 | 0.012371577 | 0.022190038 | 0.046862162 |
| DAG1 | -0.016374277 | 0.022205074 | 0.046874279 |
| RALGAPB | 0.011399036 | 0.022247867 | 0.046944954 |
| HNRNPC | 0.023239054 | 0.022304144 | 0.047044012 |
| PPP2R2D | -0.010717829 | 0.022443754 | 0.04731868 |
| COA6-AS1 | 0.011139684 | 0.022554066 | 0.047531375 |
| U2SURP | 0.019262512 | 0.022563828 | 0.047532076 |

| | | | |
|---|---|---|---|
| CCDC47 | -0.017641144 | 0.022620289 | 0.04763111 |
| COA3 | -0.027808947 | 0.022640385 | 0.047638072 |
| S100A11 | -0.028118004 | 0.022643837 | 0.047638072 |
| SMARCB1 | 0.010454906 | 0.022651946 | 0.047638072 |
| ANAPC15 | -0.011650789 | 0.022670718 | 0.047657669 |
| GAS6 | 0.021686712 | 0.022743895 | 0.047791568 |
| LRP10 | -0.014925115 | 0.022769718 | 0.047825896 |
| MPDU1 | -0.014503196 | 0.022800713 | 0.04787105 |
| MARK3 | 0.012098844 | 0.02282733 | 0.047906981 |
| MYO1C | -0.01336517 | 0.022922343 | 0.048086364 |
| SARAF | -0.026467075 | 0.022939695 | 0.048102746 |
| SGTA | -0.011489113 | 0.022977696 | 0.048162398 |
| YTHDF3 | 0.011908382 | 0.023042097 | 0.048277312 |
| CYP4V2 | 0.013319482 | 0.023088008 | 0.048353406 |
| RBM27 | 0.011100968 | 0.023108819 | 0.048376892 |
| TRMT10C | -0.011065344 | 0.023279381 | 0.048713724 |
| TRA2B | -0.01551171 | 0.023386183 | 0.048916908 |
| NFIC | -0.022103772 | 0.023573143 | 0.049287521 |
| TRABD | -0.011415902 | 0.023648878 | 0.049425371 |
| RSRP1 | -0.02119704 | 0.023706823 | 0.049525941 |
| USP48 | 0.012071305 | 0.023771347 | 0.049640166 |
| ACAA1 | -0.016121858 | 0.023826822 | 0.049735408 |
| TOMM40 | -0.01071908 | 0.023929992 | 0.049930086 |
| SRSF7 | -0.019664566 | 0.02414821 | 0.050364553 |
| ARPC2 | -0.01695072 | 0.024207184 | 0.050466673 |
| CSE1L | 0.01101108 | 0.024260502 | 0.050553514 |
| LSM5 | -0.016494872 | 0.024268896 | 0.050553514 |
| MCRIP2 | -0.021361113 | 0.02436089 | 0.050724182 |
| TACSTD2 | -0.020471642 | 0.024429998 | 0.050847077 |
| ZNF358 | -0.0119077 | 0.024567573 | 0.051112313 |
| PLCXD2 | -0.017217377 | 0.024604927 | 0.05116891 |
| GPR107 | -0.010549046 | 0.024736994 | 0.051422345 |
| GIGYF1 | -0.011871909 | 0.024780939 | 0.051492462 |
| TOMM7 | -0.038935061 | 0.024975704 | 0.051868435 |
| CISD1 | -0.023785842 | 0.024982456 | 0.051868435 |
| IMPA2 | -0.024853802 | 0.02500791 | 0.051899907 |
| NAPRT | 0.010584273 | 0.02535468 | 0.052597919 |
| E2F4 | 0.010503935 | 0.025371392 | 0.052610936 |
| SERPING1 | 0.019140491 | 0.025537386 | 0.052933372 |
| PCGF5 | 0.014440029 | 0.025586111 | 0.05301257 |
| RDH10 | -0.010520674 | 0.025892853 | 0.053626077 |
| ERI3 | 0.01227235 | 0.025917302 | 0.05365467 |
| PUS7L | 0.011760261 | 0.025933373 | 0.053665899 |
| PSMD11 | -0.012074931 | 0.02626331 | 0.054308083 |
| ATP5PD | -0.031239951 | 0.026268978 | 0.054308083 |
| ZC3H7B | -0.010952867 | 0.02627602 | 0.054308083 |
| C11orf1 | -0.014550952 | 0.026299529 | 0.054334396 |

| | | | |
|---|---|---|---|
| CPNE1 | -0.011721779 | 0.026380806 | 0.054473021 |
| C17orf49 | -0.010587197 | 0.02638824 | 0.054473021 |
| PCBD1 | -0.022413516 | 0.026443797 | 0.054565362 |
| DENR | -0.012684171 | 0.02647186 | 0.05460092 |
| HLTF | 0.014291009 | 0.026603183 | 0.054849345 |
| GTF2H5 | -0.020859896 | 0.026638278 | 0.054899247 |
| NDUFA1 | -0.043115452 | 0.026885085 | 0.055375431 |
| FBXO9 | 0.013364844 | 0.026891303 | 0.055375431 |
| WDR45B | -0.013168387 | 0.026903832 | 0.055378611 |
| IMP3 | -0.013973641 | 0.027279868 | 0.05612972 |
| MICAL3 | 0.011420901 | 0.027337371 | 0.056225086 |
| C11orf74 | 0.011660261 | 0.02755173 | 0.056642852 |
| CYB5A | -0.042514107 | 0.027582804 | 0.056683619 |
| COMMD3 | -0.01317875 | 0.027628294 | 0.056753965 |
| TPM1 | -0.01409115 | 0.027658172 | 0.056773998 |
| LLGL2 | -0.017116081 | 0.027660571 | 0.056773998 |
| FKBP8 | -0.01952186 | 0.027705851 | 0.056840775 |
| ITGA3 | 0.009676723 | 0.027715657 | 0.056840775 |
| SCAMP4 | -0.011993378 | 0.027877519 | 0.057129816 |
| PLRG1 | 0.010876081 | 0.027879259 | 0.057129816 |
| BCAS2 | -0.010635639 | 0.027937318 | 0.057225526 |
| MRPL52 | -0.022532789 | 0.028001597 | 0.057333896 |
| B3GALNT1 | -0.010629551 | 0.028094182 | 0.057500111 |
| HSPA8 | -0.027025623 | 0.028152747 | 0.057596591 |
| DARS | 0.016148773 | 0.028351478 | 0.057979635 |
| GRN | -0.016490744 | 0.028383615 | 0.058021818 |
| LUC7L | -0.011459228 | 0.028408196 | 0.058045722 |
| CLOCK | 0.011211802 | 0.028418338 | 0.058045722 |
| STK24 | 0.013096363 | 0.028542893 | 0.058276519 |
| LRRC58 | -0.011726364 | 0.028611736 | 0.058393425 |
| TMBIM1 | -0.009757963 | 0.028690144 | 0.058520657 |
| FBRSL1 | -0.013594035 | 0.028697295 | 0.058520657 |
| CBY1 | -0.009795432 | 0.0288862 | 0.058877525 |
| RAN | -0.019354038 | 0.028905874 | 0.058877525 |
| HNMT | 0.013559026 | 0.028907335 | 0.058877525 |
| UBAC1 | -0.011909769 | 0.028981638 | 0.059005023 |
| ANTKMT | -0.016290685 | 0.029050951 | 0.059122262 |
| TBC1D15 | 0.010316864 | 0.029117106 | 0.059232984 |
| SOX4 | -0.019923102 | 0.029193786 | 0.059365016 |
| STK25 | -0.011255307 | 0.029293069 | 0.05952974 |
| TBC1D9 | 0.010706921 | 0.02929841 | 0.05952974 |
| HECTD1 | 0.017593571 | 0.029320439 | 0.059550496 |
| RNF8 | -0.010907867 | 0.029416548 | 0.059721634 |
| RSPRY1 | 0.009907296 | 0.029482616 | 0.059831669 |
| DNAJB2 | -0.012139918 | 0.029519254 | 0.059881916 |
| CXCL12 | -0.03714793 | 0.029542484 | 0.059904933 |
| RALBP1 | -0.014242997 | 0.029595064 | 0.059987421 |

| | | | |
|---|---|---|---|
| CLTA | 0.022626128 | 0.029617743 | 0.060009261 |
| RAP1GAP | -0.027060079 | 0.029734208 | 0.060221029 |
| SEPTIN2 | -0.02193491 | 0.02977421 | 0.060277829 |
| CPD | 0.017126085 | 0.029829309 | 0.060355453 |
| ZNF24 | -0.012341802 | 0.029838947 | 0.060355453 |
| SOD1 | -0.034852254 | 0.029848471 | 0.060355453 |
| TMEM41B | 0.010351517 | 0.029985643 | 0.060608512 |
| TRMT112 | -0.023061569 | 0.030138236 | 0.060882312 |
| SVBP | -0.010444848 | 0.030145259 | 0.060882312 |
| KIF1B | 0.011642255 | 0.030173284 | 0.060914506 |
| SPEN | 0.013507672 | 0.030272715 | 0.061090776 |
| YME1L1 | -0.015223828 | 0.030334537 | 0.061191037 |
| SLC35A4 | -0.010167536 | 0.03034838 | 0.061194474 |
| ANXA5 | -0.022380946 | 0.030395274 | 0.061232956 |
| TMEM8B | -0.009660842 | 0.030399485 | 0.061232956 |
| GGCT | -0.010912507 | 0.030403906 | 0.061232956 |
| AHCTF1 | 0.011417382 | 0.030416453 | 0.061233763 |
| MIGA1 | 0.012860098 | 0.030480933 | 0.061339074 |
| MED31 | 0.010571625 | 0.030563285 | 0.061480256 |
| APIP | -0.013883009 | 0.030602621 | 0.06153374 |
| SYMPK | -0.0104579 | 0.030617706 | 0.06153374 |
| COL18A1 | -0.026518738 | 0.030626494 | 0.06153374 |
| PRDX4 | 0.011903946 | 0.030723117 | 0.061703279 |
| UBE2G2 | -0.013222863 | 0.03081652 | 0.061866219 |
| VKORC1 | 0.018695571 | 0.030979457 | 0.062168568 |
| NAA60 | -0.012448405 | 0.031235247 | 0.062656935 |
| RARRES2 | 0.025609643 | 0.03125486 | 0.062671341 |
| RPS19 | 0.048403602 | 0.031300205 | 0.062737309 |
| MICU1 | 0.010797364 | 0.031313413 | 0.062738838 |
| SCAF11 | 0.01984003 | 0.031414671 | 0.06291671 |
| DECR1 | 0.019655724 | 0.031432441 | 0.062927298 |
| MFHAS1 | 0.011885216 | 0.031529581 | 0.063096712 |
| SERPINB6 | 0.010378299 | 0.031668576 | 0.063349718 |
| CARNMT1 | 0.009534212 | 0.031754579 | 0.063496562 |
| CHURC1 | -0.017450804 | 0.031861309 | 0.063684718 |
| CHMP1A | -0.010650684 | 0.031890021 | 0.063716843 |
| WDR91 | -0.01143507 | 0.031932124 | 0.063775688 |
| ACACB | 0.015093828 | 0.03203901 | 0.063940647 |
| TUT4 | 0.015019203 | 0.032040086 | 0.063940647 |
| RPL15 | -0.046550657 | 0.032055348 | 0.06394579 |
| POP7 | -0.010126236 | 0.032079352 | 0.06396836 |
| HNRNPA1 | -0.028799841 | 0.032159605 | 0.064103032 |
| NUBP2 | -0.012270869 | 0.032198429 | 0.064155052 |
| TRAF4 | -0.010743369 | 0.032327865 | 0.064387501 |
| PANK1 | 0.012405666 | 0.032476051 | 0.064657098 |
| JKAMP | 0.011652806 | 0.032598843 | 0.064866443 |
| PTBP1 | -0.014824707 | 0.032606936 | 0.064866443 |

| | | | |
|---|---|---|---|
| PEX3 | 0.009690606 | 0.032620325 | 0.064867478 |
| RPP25L | -0.011157072 | 0.032973488 | 0.06554391 |
| POLR1D | 0.013854658 | 0.03310303 | 0.065775472 |
| MCRS1 | -0.009690285 | 0.03329671 | 0.066134245 |
| TMEM129 | -0.012583641 | 0.033383631 | 0.066280773 |
| RBM14 | 0.010219792 | 0.033425921 | 0.066338609 |
| TCEAL2 | -0.010253222 | 0.033457313 | 0.06637478 |
| RPL22L1 | -0.011065876 | 0.03347274 | 0.066379261 |
| EMC7 | -0.01519212 | 0.033738257 | 0.066879494 |
| TTC14 | 0.018833969 | 0.033777793 | 0.066931546 |
| AC024022.1 | 0.01077781 | 0.033830672 | 0.067009987 |
| SNHG9 | 0.016616917 | 0.033858792 | 0.067039345 |
| TMEM258 | 0.025537099 | 0.034104337 | 0.067499005 |
| TRIM56 | -0.011070894 | 0.034128421 | 0.067520161 |
| LAPTM4B | -0.015948342 | 0.034209416 | 0.067646178 |
| SEM1 | -0.022363532 | 0.034218955 | 0.067646178 |
| S100A4 | -0.01553974 | 0.034375485 | 0.067928976 |
| EMD | -0.011273966 | 0.034413426 | 0.067977305 |
| PNKD | 0.025858658 | 0.0344824 | 0.068086871 |
| VPS28 | 0.021418075 | 0.034549416 | 0.068192484 |
| HSPA4 | -0.013112611 | 0.034596749 | 0.068259182 |
| POLR2K | -0.016971742 | 0.0346304 | 0.068298844 |
| TMX4 | -0.019556885 | 0.034748044 | 0.068480257 |
| PDAP1 | -0.014473284 | 0.034749553 | 0.068480257 |
| MAEA | -0.012266638 | 0.034808825 | 0.068570256 |
| SIN3B | -0.009646402 | 0.034886543 | 0.068696508 |
| RAP2C | 0.019081224 | 0.034919725 | 0.068728242 |
| G6PC3 | -0.010899976 | 0.034929926 | 0.068728242 |
| BLOC1S1 | -0.025166356 | 0.034987313 | 0.068814297 |
| EBP | 0.014064782 | 0.035052856 | 0.068916321 |
| SELENOI | 0.009347204 | 0.035102694 | 0.068987399 |
| SHISA3 | -0.010474177 | 0.035204267 | 0.069139916 |
| TRIR | -0.020051416 | 0.035207729 | 0.069139916 |
| SF3B2 | -0.014029464 | 0.035533485 | 0.069739475 |
| NME2 | -0.037539811 | 0.035544655 | 0.069739475 |
| CTNNB1 | -0.019228626 | 0.035554543 | 0.069739475 |
| YWHAQ | -0.018446957 | 0.035583292 | 0.069768719 |
| RBL2 | 0.012882758 | 0.03565729 | 0.069886625 |
| RPL21 | 0.042983283 | 0.035730217 | 0.070002341 |
| TCEA1 | -0.015031781 | 0.035769034 | 0.070051165 |
| PAK4 | -0.011045713 | 0.035990575 | 0.070457665 |
| STARD7 | -0.016524903 | 0.036422587 | 0.071275722 |
| APP | -0.038903877 | 0.036474792 | 0.071350185 |
| DBT | 0.016397523 | 0.036494701 | 0.071361439 |
| KRR1 | -0.012201008 | 0.036632897 | 0.07160389 |
| ADAR | -0.012757429 | 0.036701036 | 0.071709272 |
| NREP | 0.011266252 | 0.036844782 | 0.071962241 |

| | | | |
|---|---|---|---|
| LEMD3 | 0.009756916 | 0.037092087 | 0.072417201 |
| NUTF2 | -0.01125061 | 0.037297726 | 0.072790491 |
| ATP5MPL | -0.034636099 | 0.037419119 | 0.072999141 |
| SLC4A2 | -0.014344999 | 0.037505291 | 0.073138945 |
| CCDC130 | 0.009122329 | 0.037557681 | 0.073212788 |
| CERS6 | 0.009417536 | 0.037844396 | 0.073743178 |
| NR1D2 | -0.010385149 | 0.037879192 | 0.073782461 |
| NR2F2 | -0.023700823 | 0.038276117 | 0.074526807 |
| ZNF770 | -0.011098864 | 0.038326144 | 0.074595402 |
| EMC4 | -0.01302311 | 0.038366063 | 0.074644278 |
| AL031710.1 | -0.010166816 | 0.038407985 | 0.074681048 |
| CASK | 0.013827791 | 0.038414592 | 0.074681048 |
| IFT20 | -0.010968538 | 0.038640116 | 0.075090526 |
| TMEM207 | 0.022574166 | 0.038697597 | 0.07517325 |
| NPC2 | -0.02138065 | 0.038795557 | 0.075334515 |
| FEZ2 | 0.011617203 | 0.038880706 | 0.075470788 |
| NASP | 0.010316125 | 0.039309226 | 0.076273213 |
| ZNF493 | -0.012700067 | 0.039506733 | 0.07662695 |
| ZDHHC7 | -0.010118752 | 0.039526593 | 0.076635983 |
| MRPL36 | -0.022365198 | 0.039574574 | 0.076699511 |
| CCT8 | 0.015173266 | 0.039650689 | 0.076817495 |
| CCDC124 | -0.010528566 | 0.039803612 | 0.077084136 |
| OPLAH | -0.010993721 | 0.039869065 | 0.077181244 |
| MOB1A | -0.014026783 | 0.040527963 | 0.078426665 |
| ACSM3 | 0.011610375 | 0.040675438 | 0.078681682 |
| MARCH6 | 0.019905226 | 0.040701177 | 0.078681682 |
| LINC00662 | 0.009157313 | 0.040706571 | 0.078681682 |
| IDH1 | 0.009684818 | 0.040787509 | 0.078807909 |
| GOLGA8B | 0.014978981 | 0.040962607 | 0.079110953 |
| MXI1 | 0.011124285 | 0.040975739 | 0.079110953 |
| RBBP6 | 0.012455673 | 0.041101201 | 0.079286724 |
| JMJD8 | -0.012226767 | 0.041101934 | 0.079286724 |
| KCNQ1OT1 | 0.016264144 | 0.041113965 | 0.079286724 |
| SPX | 0.011037914 | 0.041182733 | 0.07938897 |
| TPGS2 | -0.009883297 | 0.041345031 | 0.079671369 |
| PSMD9 | -0.013741919 | 0.041637505 | 0.080204304 |
| SMAD7 | -0.009948171 | 0.041744358 | 0.080379416 |
| CNOT1 | 0.012977352 | 0.041814584 | 0.080483894 |
| GSTM3 | 0.029045239 | 0.042020432 | 0.080848141 |
| RNF115 | 0.011436759 | 0.042035901 | 0.080848141 |
| PA2G4 | -0.013729278 | 0.042146169 | 0.081011558 |
| SMIM10L1 | -0.00923303 | 0.042153009 | 0.081011558 |
| ANKMY2 | 0.009170543 | 0.042346306 | 0.08135203 |
| GALK1 | -0.009696548 | 0.042526358 | 0.081666808 |
| MIB2 | -0.009568971 | 0.042555229 | 0.08169113 |
| MRPS6 | 0.036004525 | 0.042692358 | 0.081923174 |
| FMR1 | 0.012651946 | 0.042726756 | 0.081957982 |

| | | | |
|---|---|---|---|
| SLAIN2 | 0.010284019 | 0.042786717 | 0.08204178 |
| HIPK1 | -0.010379183 | 0.042898343 | 0.082224543 |
| FAM126B | 0.011482166 | 0.043159419 | 0.082693513 |
| YIPF5 | -0.011357041 | 0.043379058 | 0.083082764 |
| FCGRT | 0.016966632 | 0.043644455 | 0.083559323 |
| TMED9 | -0.015752765 | 0.043734524 | 0.083699976 |
| RPS3 | 0.042782804 | 0.043806126 | 0.083774651 |
| MSL1 | -0.00892033 | 0.043810249 | 0.083774651 |
| NDUFV3 | -0.018410432 | 0.043823399 | 0.083774651 |
| RIOK3 | -0.010750825 | 0.044127923 | 0.084315775 |
| PPP1R2 | -0.010920517 | 0.044158045 | 0.084315775 |
| YTHDF2 | -0.01062416 | 0.04416185 | 0.084315775 |
| PPP6C | -0.010685803 | 0.044173371 | 0.084315775 |
| PLAGL1 | 0.009371226 | 0.0442615 | 0.084434985 |
| DDX46 | -0.013651449 | 0.044278053 | 0.084434985 |
| PRKCI | 0.011349617 | 0.044298293 | 0.084434985 |
| SCAMP2 | -0.011638621 | 0.044302824 | 0.084434985 |
| SMIM30 | -0.013376845 | 0.044355148 | 0.08450276 |
| ZNF626 | 0.009480027 | 0.044449527 | 0.084650573 |
| HSD17B10 | 0.015093325 | 0.044506006 | 0.084726123 |
| SYF2 | 0.017058908 | 0.044561579 | 0.084799894 |
| SINHCAF | 0.009639929 | 0.044623015 | 0.084884761 |
| RCC1L | -0.009337298 | 0.044669687 | 0.08494149 |
| SNX19 | -0.008744384 | 0.044785703 | 0.085124202 |
| MRPL19 | 0.014009008 | 0.044799545 | 0.085124202 |
| ILF3 | -0.014821151 | 0.044836887 | 0.085145508 |
| CIRBP | -0.030529352 | 0.044848654 | 0.085145508 |
| GALNT11 | -0.01237671 | 0.04486143 | 0.085145508 |
| CAPZA1 | 0.010703711 | 0.044951503 | 0.085253611 |
| TTC37 | 0.015299085 | 0.044952212 | 0.085253611 |
| PGGT1B | -0.009007431 | 0.04498023 | 0.085274667 |
| AIP | 0.010902784 | 0.045046346 | 0.085367905 |
| RAB9A | -0.009323604 | 0.045118866 | 0.085467402 |
| NENF | -0.01782021 | 0.045132756 | 0.085467402 |
| LTBP3 | -0.016264286 | 0.045375757 | 0.085895303 |
| DSG2 | -0.016113429 | 0.045514317 | 0.086125252 |
| RSBN1L | 0.01139316 | 0.045565586 | 0.086189914 |
| DDOST | 0.010830137 | 0.045668194 | 0.0863516 |
| RPL28 | 0.047561387 | 0.045897418 | 0.086752487 |
| R3HDM2 | 0.012564917 | 0.045927699 | 0.086777185 |
| CDK5RAP3 | 0.011811867 | 0.045948326 | 0.086783632 |
| IWS1 | 0.009362978 | 0.046061379 | 0.086905081 |
| TMEM260 | 0.009780321 | 0.046061853 | 0.086905081 |
| DNAJC4 | -0.01561819 | 0.046064348 | 0.086905081 |
| PAFAH1B2 | -0.010276817 | 0.046228107 | 0.087181403 |
| MAPKAP1 | 0.010728183 | 0.046317394 | 0.087317122 |
| TSC2 | 0.010275015 | 0.046372948 | 0.087378691 |

| | | | |
|---|---|---|---|
| PALM | -0.016308335 | 0.046390595 | 0.087378691 |
| ASPH | -0.015490069 | 0.046402054 | 0.087378691 |
| SGPP2 | -0.012662144 | 0.046571829 | 0.087665643 |
| FGFR1OP2 | -0.010492568 | 0.046683668 | 0.087843363 |
| ACAD8 | -0.009694951 | 0.046944999 | 0.088299069 |
| USP12 | 0.010128748 | 0.046960881 | 0.088299069 |
| AKAP1 | -0.015384974 | 0.047001837 | 0.088343125 |
| NEU1 | -0.011011161 | 0.04702878 | 0.088360821 |
| TXNL1 | 0.015123294 | 0.047422474 | 0.089067321 |
| PTGES3 | -0.020739152 | 0.047703578 | 0.089561914 |
| IVNS1ABP | 0.027754743 | 0.047799832 | 0.089709216 |
| VWA1 | -0.009183223 | 0.048494662 | 0.090965611 |
| DCTN4 | 0.009073285 | 0.048505368 | 0.090965611 |
| MYL6 | -0.036941542 | 0.048808017 | 0.09149915 |
| HMGN1 | -0.023709482 | 0.048885945 | 0.09161117 |
| CTSF | 0.016514561 | 0.048933858 | 0.091666882 |
| PLPBP | -0.010930285 | 0.049026145 | 0.091805645 |
| TRPT1 | -0.011753308 | 0.049087169 | 0.091857917 |
| ZFAND6 | 0.013113128 | 0.049090504 | 0.091857917 |
| PTRHD1 | -0.010006795 | 0.049126066 | 0.091890353 |
| TMEM223 | -0.011093793 | 0.049263388 | 0.092113033 |
| BTF3L4 | -0.012280293 | 0.049554056 | 0.092622172 |
| PDK2 | -0.011436158 | 0.049639848 | 0.092722182 |
| PPP2R5A | 0.011138677 | 0.04964435 | 0.092722182 |
| UQCR10 | -0.035229734 | 0.04980803 | 0.092993438 |
| PSMB7 | 0.01540681 | 0.050108064 | 0.093518974 |
| PKD1 | -0.009025423 | 0.050159343 | 0.093580033 |
| PHYKPL | 0.008764644 | 0.050302822 | 0.093789595 |
| SELENON | -0.011461814 | 0.05030888 | 0.093789595 |
| ACADSB | 0.013404271 | 0.050357647 | 0.093845804 |
| TIA1 | 0.014539584 | 0.050431327 | 0.093935296 |
| BECN1 | -0.010573518 | 0.050442937 | 0.093935296 |
| PPP2R2A | 0.010856221 | 0.050472265 | 0.093955202 |
| CTSC | 0.021774843 | 0.050597292 | 0.094153175 |
| ITGAV | 0.013118337 | 0.050698586 | 0.094306853 |
| MRPL11 | -0.011709999 | 0.050877277 | 0.094604336 |
| CREBL2 | -0.010351231 | 0.050972219 | 0.094745928 |
| IDI1 | -0.010824819 | 0.051006459 | 0.094774625 |
| CREG1 | -0.010123621 | 0.051043495 | 0.094808496 |
| SMARCC2 | -0.011719416 | 0.051078452 | 0.094838481 |
| ZNRD1 | -0.009202777 | 0.051099261 | 0.094842185 |
| INTS10 | 0.012716723 | 0.051232503 | 0.095054489 |
| GPI | -0.020105201 | 0.051300672 | 0.095145948 |
| WAPL | 0.0104375 | 0.05140916 | 0.09531209 |
| PCSK1N | 0.035880648 | 0.051436453 | 0.095327633 |
| IPO8 | 0.009555467 | 0.051463686 | 0.095343052 |
| TAX1BP1 | 0.01837248 | 0.051692437 | 0.095731659 |

| | | | |
|---|---|---|---|
| BCAR1 | -0.00926754 | 0.052168358 | 0.096577558 |
| MRPL17 | -0.010594202 | 0.052434695 | 0.097032215 |
| RPL30 | -0.045246496 | 0.052452447 | 0.097032215 |
| RSF1 | 0.01311463 | 0.052580969 | 0.09722643 |
| HGSNAT | 0.010560591 | 0.052596008 | 0.09722643 |
| DRAP1 | -0.014483612 | 0.052700804 | 0.097364009 |
| ZER1 | 0.008126171 | 0.052709062 | 0.097364009 |
| CNIH1 | -0.009973402 | 0.052852693 | 0.097593563 |
| SLF2 | 0.008735065 | 0.052993376 | 0.097817507 |
| CAAP1 | 0.010423887 | 0.053142363 | 0.098056607 |
| NDUFB8 | 0.02844199 | 0.053302605 | 0.098316295 |
| ATP6V0A1 | -0.008863504 | 0.05337324 | 0.098410572 |
| LTA4H | 0.010369501 | 0.053682593 | 0.098944772 |
| CRTAP | -0.010023169 | 0.053710855 | 0.09896068 |
| PARP1 | -0.011362526 | 0.053742707 | 0.098983188 |
| RAB2A | -0.01924941 | 0.053826517 | 0.099101342 |
| RAB14 | -0.015802365 | 0.054086968 | 0.099517599 |
| NIPSNAP2 | -0.016090111 | 0.054092089 | 0.099517599 |
| MLXIP | -0.008509123 | 0.05456397 | 0.10033842 |
| GLOD4 | -0.011604642 | 0.05457805 | 0.10033842 |
| TP53I3 | 0.012797794 | 0.054762163 | 0.100640198 |
| CDIPT | -0.011481577 | 0.054833608 | 0.100734773 |
| PRKAR2A | -0.010612448 | 0.054990348 | 0.100985918 |
| ASXL2 | 0.009015566 | 0.055096697 | 0.101144373 |
| INTS11 | -0.010291757 | 0.055156532 | 0.101217356 |
| DNAJA3 | -0.008531068 | 0.055180941 | 0.1012253 |
| CETN3 | 0.00948929 | 0.055231884 | 0.101281894 |
| RBPJ | 0.01206002 | 0.055262499 | 0.101301185 |
| PCNX3 | -0.008362135 | 0.055314525 | 0.101342795 |
| UBE2L3 | -0.013012231 | 0.055325406 | 0.101342795 |
| NDUFA7 | -0.021971401 | 0.05550273 | 0.101589424 |
| TMEM123 | 0.0184656 | 0.055534137 | 0.101589424 |
| SESTD1 | 0.009119257 | 0.055540274 | 0.101589424 |
| GP2 | -0.017569252 | 0.055540657 | 0.101589424 |
| MGST2 | 0.011868653 | 0.055701083 | 0.101845905 |
| RNASEK | 0.029106596 | 0.056061166 | 0.102467128 |
| ZFP36L1 | -0.021625094 | 0.056125638 | 0.102487635 |
| CELF6 | 0.010463586 | 0.05613321 | 0.102487635 |
| AIFM1 | -0.011245522 | 0.05614307 | 0.102487635 |
| AP2B1 | 0.015448628 | 0.056153709 | 0.102487635 |
| GADD45GIP1 | -0.0173044 | 0.056203056 | 0.102540573 |
| IGFBP3 | -0.012686973 | 0.056248837 | 0.10258697 |
| GOLGA4 | 0.01839195 | 0.056328576 | 0.102695245 |
| SPPL2B | -0.010947794 | 0.056423001 | 0.102819785 |
| ERP44 | 0.011101883 | 0.05643768 | 0.102819785 |
| AMD1 | -0.015633484 | 0.056563455 | 0.102984067 |
| CCT2 | -0.01293304 | 0.056568713 | 0.102984067 |

| | | | |
|---|---|---|---|
| SLC25A36 | -0.01674618 | 0.05671728 | 0.103199388 |
| LAGE3 | -0.010783295 | 0.056727932 | 0.103199388 |
| NSFL1C | -0.008777929 | 0.056757715 | 0.103216321 |
| ENTPD3-AS1 | 0.008705755 | 0.057211661 | 0.104004321 |
| GUCD1 | -0.009054902 | 0.057394828 | 0.104299685 |
| TPR | -0.013928438 | 0.057458448 | 0.10437767 |
| PRPF4B | 0.016088252 | 0.05760819 | 0.104611991 |
| TMEM106C | -0.01005231 | 0.057659662 | 0.104667756 |
| SPG11 | 0.010469817 | 0.057725432 | 0.104749425 |
| UBXN6 | -0.013889063 | 0.05798504 | 0.105146245 |
| CCT6A | -0.013997414 | 0.057985828 | 0.105146245 |
| PPP2CB | -0.013497074 | 0.05801487 | 0.105158344 |
| GCC2 | -0.016715217 | 0.058034222 | 0.105158344 |
| RPS5 | 0.033507685 | 0.058105569 | 0.105249793 |
| JTB | -0.017570298 | 0.058178102 | 0.105343323 |
| SUCLG1 | -0.022259939 | 0.058244347 | 0.105425406 |
| RPL3 | -0.047456396 | 0.058467766 | 0.105791819 |
| PABPN1 | -0.018339949 | 0.0589061 | 0.1065467 |
| MRPL13 | -0.013836901 | 0.058967523 | 0.106619542 |
| KDM1A | 0.009030519 | 0.059014683 | 0.106666553 |
| C1D | 0.01374537 | 0.059036238 | 0.106667267 |
| ARF6 | -0.012127808 | 0.059222938 | 0.106966259 |
| SRSF2 | -0.019007977 | 0.05970484 | 0.107798029 |
| CUTA | 0.024459312 | 0.059765436 | 0.107868802 |
| RPS8 | 0.044564808 | 0.059962745 | 0.108186183 |
| H2AFY | -0.013314865 | 0.06030371 | 0.108762433 |
| NHLRC2 | 0.009593438 | 0.06044244 | 0.108973654 |
| RAB11FIP2 | 0.010247258 | 0.060840146 | 0.109623076 |
| WAC | 0.014995465 | 0.060846135 | 0.109623076 |
| FAM120A | -0.01589331 | 0.061052546 | 0.109955657 |
| PDK3 | -0.009820985 | 0.061085946 | 0.109976519 |
| ENTPD6 | -0.008832384 | 0.061206644 | 0.110154477 |
| DAAM1 | 0.01355446 | 0.061240342 | 0.110175789 |
| ATP5F1D | -0.036514226 | 0.061619238 | 0.110782395 |
| AFDN | 0.015753447 | 0.061623846 | 0.110782395 |
| CDC42SE1 | -0.010153196 | 0.061643447 | 0.110782395 |
| ANKRD2 | 0.011038104 | 0.061732327 | 0.110902588 |
| PBXIP1 | -0.012039829 | 0.061766421 | 0.110924306 |
| SASH1 | 0.008284554 | 0.061860168 | 0.1110531 |
| DHX40 | 0.009707432 | 0.06188375 | 0.111055886 |
| KIAA0100 | -0.008675063 | 0.061969417 | 0.111170046 |
| COG2 | 0.008330438 | 0.062260805 | 0.111653048 |
| MFSD10 | 0.008308481 | 0.062417111 | 0.111893548 |
| MAGED2 | 0.017186453 | 0.062590801 | 0.11216503 |
| RPL32 | 0.047133096 | 0.062645488 | 0.112223136 |
| P4HTM | 0.012291017 | 0.06267469 | 0.112235565 |
| SPIRE1 | 0.009977888 | 0.062998822 | 0.112775945 |

| | | | |
|---|---|---|---|
| CERT1 | 0.010807504 | 0.063204119 | 0.113103289 |
| CCDC50 | 0.013473542 | 0.0633047 | 0.113243078 |
| C8orf33 | -0.009480657 | 0.063484683 | 0.113524756 |
| NSD3 | 0.013064891 | 0.063556733 | 0.11358489 |
| MACROD1 | 0.015818586 | 0.063563375 | 0.11358489 |
| ZC3H13 | 0.01241581 | 0.064522667 | 0.115258243 |
| ARAF | -0.009126908 | 0.0648059 | 0.115723182 |
| ADSS | 0.010961789 | 0.064852948 | 0.115766187 |
| CYB5R1 | -0.010103817 | 0.064896031 | 0.115802087 |
| SLC25A46 | 0.009264621 | 0.064926555 | 0.115815556 |
| SACM1L | 0.01022498 | 0.065358095 | 0.116544095 |
| ISOC1 | -0.010943561 | 0.065534814 | 0.116771271 |
| KDSR | -0.011418522 | 0.065539167 | 0.116771271 |
| MECP2 | 0.011187917 | 0.065554988 | 0.116771271 |
| KCNQ1 | 0.011859891 | 0.065796433 | 0.117145251 |
| BAG6 | -0.011188119 | 0.065811417 | 0.117145251 |
| SLC39A9 | -0.011484266 | 0.06589285 | 0.117248803 |
| SMARCC1 | 0.010259081 | 0.065926641 | 0.117267536 |
| GSTO1 | 0.015120045 | 0.066296578 | 0.117883968 |
| CAST | 0.016556928 | 0.066360493 | 0.117956011 |
| MAX | 0.008177029 | 0.066767765 | 0.118638105 |
| LSM2 | -0.010170803 | 0.067035659 | 0.119046282 |
| G3BP1 | -0.01442017 | 0.067044712 | 0.119046282 |
| MIF | -0.041144332 | 0.067215742 | 0.119274774 |
| PRKRA | -0.008916521 | 0.067220717 | 0.119274774 |
| RHOT2 | -0.011211394 | 0.067276165 | 0.119331156 |
| PRKAG2 | 0.009476895 | 0.067465007 | 0.119624024 |
| HOXB6 | -0.010673558 | 0.067553296 | 0.119686153 |
| MGA | 0.009006359 | 0.067555642 | 0.119686153 |
| PSMA7 | -0.017917764 | 0.067571274 | 0.119686153 |
| PAK2 | -0.009638676 | 0.067793862 | 0.119996089 |
| LNPEP | 0.01506935 | 0.067793233 | 0.119996089 |
| NOMO1 | -0.008297859 | 0.068088777 | 0.12047579 |
| PCP4 | -0.01792809 | 0.068198643 | 0.120618771 |
| STT3A | -0.009505981 | 0.06821744 | 0.120618771 |
| FGFR3 | 0.013645776 | 0.068279115 | 0.12068549 |
| COX11 | -0.012496349 | 0.068397692 | 0.120852704 |
| COPZ1 | -0.013731248 | 0.06891562 | 0.12169101 |
| ATOX1 | 0.020221818 | 0.068920419 | 0.12169101 |
| SRSF10 | 0.015870468 | 0.069345939 | 0.122399467 |
| SLK | -0.011471546 | 0.069390317 | 0.122405427 |
| CUX1 | 0.011448294 | 0.069397879 | 0.122405427 |
| MRPL35 | -0.010988438 | 0.069512408 | 0.12256455 |
| NINJ1 | -0.014604298 | 0.06955055 | 0.122588925 |
| CEP170B | -0.008119955 | 0.069601591 | 0.122636009 |
| MRPS2 | -0.009222184 | 0.069688748 | 0.122711236 |
| SIAE | -0.009720812 | 0.069692971 | 0.122711236 |

| | | | |
|---|---|---|---|
| ISCA2 | -0.011754566 | 0.069778242 | 0.122818477 |
| SNU13 | -0.019631978 | 0.069915829 | 0.123017695 |
| COX7A1 | -0.026898405 | 0.069975253 | 0.123079291 |
| TSR3 | -0.010540681 | 0.070349306 | 0.123694054 |
| DCUN1D1 | 0.009317271 | 0.070535014 | 0.123977337 |
| TBC1D1 | 0.011675991 | 0.070892503 | 0.124544865 |
| SNX2 | 0.011899431 | 0.070907313 | 0.124544865 |
| TYW3 | -0.008845754 | 0.070977276 | 0.12459457 |
| GRHPR | -0.011756948 | 0.070985044 | 0.12459457 |
| SF3A1 | -0.008137739 | 0.071199802 | 0.124928021 |
| EIF2AK2 | 0.009751855 | 0.071426336 | 0.125281893 |
| ZNF593 | -0.01017516 | 0.071505522 | 0.125377161 |
| MRPL3 | -0.011561375 | 0.071820037 | 0.125851109 |
| ELP5 | -0.008820437 | 0.071825757 | 0.125851109 |
| IGSF3 | -0.011803009 | 0.072143454 | 0.126363847 |
| FXR1 | 0.013971953 | 0.072213145 | 0.126441981 |
| CRBN | 0.010162734 | 0.072431294 | 0.126779914 |
| CFL1 | -0.022148282 | 0.072564922 | 0.126969723 |
| ARL14EP | -0.008906086 | 0.073108253 | 0.127876024 |
| CHKB | -0.0087682 | 0.073201498 | 0.12799471 |
| WSCD1 | -0.008416095 | 0.073401836 | 0.128256925 |
| USP7 | -0.009705847 | 0.073402347 | 0.128256925 |
| MAGT1 | -0.011898865 | 0.073545822 | 0.128463094 |
| ZNF37A | 0.009443765 | 0.073820432 | 0.128898094 |
| PTPA | -0.012305406 | 0.073949917 | 0.129079477 |
| TOP2B | 0.013415431 | 0.073998786 | 0.129120069 |
| HNRNPR | -0.013620851 | 0.074055355 | 0.129174064 |
| ALDH3A2 | -0.012585024 | 0.074243034 | 0.129456637 |
| TNS1 | -0.014656467 | 0.074401617 | 0.129688295 |
| MAP2K3 | -0.011688528 | 0.074505718 | 0.129824862 |
| SCNN1A | 0.020854246 | 0.074641891 | 0.130007303 |
| SEC11C | -0.015617964 | 0.074662 | 0.130007303 |
| SMIM12 | -0.009406457 | 0.074698899 | 0.13002664 |
| TBC1D9B | -0.008249654 | 0.07474468 | 0.13006142 |
| ZNF22 | -0.008355698 | 0.074946815 | 0.130368148 |
| NUS1 | -0.008545068 | 0.075120126 | 0.130624544 |
| CASC3 | -0.008305379 | 0.075166137 | 0.130659481 |
| LYPLA2 | -0.009061863 | 0.075315959 | 0.130874784 |
| RPS23 | -0.048348966 | 0.075483327 | 0.131120417 |
| RPL18 | 0.037098914 | 0.075747174 | 0.131533415 |
| SH2D4A | 0.007989045 | 0.075881292 | 0.131720934 |
| EFHC1 | 0.007784452 | 0.076249405 | 0.132314372 |
| RPL13 | 0.046396118 | 0.076411178 | 0.132549466 |
| SLC20A1 | -0.009130067 | 0.076533456 | 0.13271591 |
| UBL5 | -0.032107376 | 0.076576258 | 0.132744469 |
| ANAPC13 | -0.013548022 | 0.076608855 | 0.132755324 |
| UCKL1 | -0.008729745 | 0.077002373 | 0.133391395 |

| | | | |
|---|---|---|---|
| SNHG29 | 0.035751398 | 0.07703503 | 0.133402125 |
| SLC35E1 | -0.008316203 | 0.077343273 | 0.133889917 |
| NT5DC2 | -0.009212493 | 0.077741931 | 0.134533839 |
| GOLGA8A | 0.013223639 | 0.077833522 | 0.134646117 |
| SNX9 | 0.008688598 | 0.078095388 | 0.13505278 |
| ATF4 | -0.01133334 | 0.078170978 | 0.13513714 |
| DCP2 | 0.008346064 | 0.078238727 | 0.135207893 |
| CBWD1 | -0.008376583 | 0.078308511 | 0.135273698 |
| MCL1 | -0.007971607 | 0.078330475 | 0.135273698 |
| GOSR1 | -0.010504256 | 0.078689549 | 0.135847266 |
| FAM136A | -0.008719817 | 0.078851636 | 0.136057566 |
| ZNRF3 | 0.008349555 | 0.078865346 | 0.136057566 |
| PMPCB | 0.010614308 | 0.079035453 | 0.136304386 |
| B4GALT5 | 0.007575522 | 0.079427896 | 0.136934344 |
| RPLP2 | 0.028772519 | 0.079682585 | 0.137326465 |
| TMCO1 | 0.016643805 | 0.079863013 | 0.137590379 |
| PARVA | 0.008926027 | 0.080364486 | 0.13840703 |
| TMOD3 | -0.009659278 | 0.080478351 | 0.138555794 |
| PNRC1 | 0.015165711 | 0.08090957 | 0.139217186 |
| PPP5C | -0.009258684 | 0.080917745 | 0.139217186 |
| CREB3L2 | 0.015619567 | 0.081269051 | 0.139773895 |
| CHPF | -0.010172745 | 0.081402863 | 0.139956286 |
| MAP2K2 | -0.012791978 | 0.081484014 | 0.140048044 |
| CHMP3 | -0.01208999 | 0.08158142 | 0.140167668 |
| MRPS10 | -0.008702596 | 0.081724807 | 0.140366185 |
| OXA1L | -0.009474892 | 0.081876012 | 0.14057799 |
| MPHOSPH9 | 0.008273001 | 0.081933905 | 0.140629491 |
| RBM42 | -0.009039146 | 0.082076136 | 0.140825664 |
| SNRPB2 | -0.012873043 | 0.08248996 | 0.141487542 |
| PRR34-AS1 | -0.013606131 | 0.082620207 | 0.141619994 |
| HSDL2 | 0.0111489 | 0.082623369 | 0.141619994 |
| GNPTAB | -0.009136348 | 0.082851538 | 0.141962815 |
| SLC50A1 | -0.007971879 | 0.082926992 | 0.142043821 |
| SLC44A1 | 0.00987587 | 0.083083458 | 0.142232779 |
| KHSRP | -0.00975026 | 0.083093738 | 0.142232779 |
| TMEM14A | -0.009982049 | 0.083136371 | 0.142257449 |
| DDT | -0.02423281 | 0.0833188 | 0.14244462 |
| PXMP2 | 0.01500513 | 0.083319295 | 0.14244462 |
| CAPZB | -0.013916774 | 0.083330527 | 0.14244462 |
| GOLPH3 | 0.010254859 | 0.083405038 | 0.14252366 |
| RTF2 | -0.011431749 | 0.083519322 | 0.142662808 |
| IREB2 | 0.008866005 | 0.083543069 | 0.142662808 |
| NAA10 | -0.009925529 | 0.083963845 | 0.143332794 |
| PDCD4 | 0.010088325 | 0.084494997 | 0.144190685 |
| STAM2 | 0.011083575 | 0.084660169 | 0.144423658 |
| MRPL4 | -0.012090886 | 0.084710394 | 0.144460451 |
| FAF2 | 0.00749528 | 0.084863485 | 0.144614144 |

| | | | |
|---|---|---|---|
| CIAO1 | -0.010073679 | 0.084905993 | 0.144614144 |
| SYNGR1 | -0.010486612 | 0.084913882 | 0.144614144 |
| WTAP | -0.012041031 | 0.084915268 | 0.144614144 |
| NDUFA4 | -0.04367335 | 0.085082028 | 0.144849207 |
| B3GAT3 | -0.009968579 | 0.085450531 | 0.145427457 |
| TMTC3 | 0.00759236 | 0.085813531 | 0.145995954 |
| CSKMT | 0.007805138 | 0.086193591 | 0.146593082 |
| FAM20B | -0.008238848 | 0.086471289 | 0.147015774 |
| BRD7 | -0.010325767 | 0.086783027 | 0.147496035 |
| EPRS | -0.010923025 | 0.086865679 | 0.147541911 |
| PPP1CB | -0.018251714 | 0.086868556 | 0.147541911 |
| SUCLG2 | 0.016185444 | 0.087126334 | 0.147929893 |
| PCBP2 | -0.02563691 | 0.087208544 | 0.14801962 |
| TMEM14C | -0.017670239 | 0.087307661 | 0.148119128 |
| MRPL16 | -0.013037146 | 0.087325937 | 0.148119128 |
| PHKB | 0.0111243 | 0.087364781 | 0.148135171 |
| ZDHHC5 | -0.008142039 | 0.087415256 | 0.148170917 |
| GON7 | -0.008085808 | 0.087457651 | 0.148192948 |
| VCL | -0.009288751 | 0.087542215 | 0.148286393 |
| EWSR1 | 0.012814542 | 0.087601111 | 0.148336311 |
| UGP2 | -0.013291573 | 0.087682104 | 0.148423602 |
| TMEM183A | -0.007629989 | 0.088309957 | 0.149436219 |
| TMEM248 | -0.01104348 | 0.088387847 | 0.149517831 |
| NDUFB1 | -0.032522672 | 0.088727569 | 0.150042159 |
| NTRK2 | -0.022529317 | 0.088993981 | 0.150442206 |
| FKBP9 | -0.012492042 | 0.089092044 | 0.150557491 |
| PAFAH1B3 | 0.009440451 | 0.089191873 | 0.150675681 |
| MEA1 | -0.009189658 | 0.089651656 | 0.151401674 |
| UBE2Z | -0.009001271 | 0.089790579 | 0.151585501 |
| MIR29B2CHG | 0.012421989 | 0.089845638 | 0.151627674 |
| GTF2I | -0.020426618 | 0.089927715 | 0.151715398 |
| SLC38A10 | -0.009880503 | 0.089996522 | 0.151780685 |
| QDPR | -0.007969067 | 0.090245898 | 0.152150358 |
| SSBP3 | -0.011599773 | 0.090445376 | 0.152435688 |
| IDS | -0.010901143 | 0.090818914 | 0.153014086 |
| SDCBP | -0.012407765 | 0.091117973 | 0.153466656 |
| ZFHX3 | 0.008819726 | 0.091286937 | 0.153699883 |
| TYK2 | -0.009059793 | 0.091593618 | 0.15416475 |
| UBE3A | 0.012878696 | 0.091641057 | 0.154193113 |
| RPS27A | 0.043681627 | 0.09188997 | 0.15456034 |
| PAIP2 | 0.0141915 | 0.092498473 | 0.155531956 |
| ITGAE | -0.008059895 | 0.092564592 | 0.155591233 |
| ZC3H7A | 0.00904699 | 0.092603329 | 0.15560446 |
| COPG1 | -0.009253014 | 0.09265568 | 0.155640548 |
| RPL27 | -0.026809489 | 0.092694548 | 0.155653969 |
| PNRC2 | -0.012445413 | 0.092921875 | 0.15598374 |
| NELFCD | -0.009306541 | 0.092959815 | 0.155995482 |

| | | | |
|---|---|---|---|
| PRKAA1 | 0.008953972 | 0.093237819 | 0.156364549 |
| IRF2BP2 | -0.014498107 | 0.093241784 | 0.156364549 |
| TOX4 | -0.008055577 | 0.093281799 | 0.156379631 |
| ALG5 | 0.010189318 | 0.093375324 | 0.156484378 |
| TIMM17B | -0.011244201 | 0.093466431 | 0.156585004 |
| CALM2 | -0.026568989 | 0.093605515 | 0.156765914 |
| SEPHS1 | -0.008895389 | 0.093714697 | 0.156896641 |
| MRPS25 | -0.012237342 | 0.093871117 | 0.157054469 |
| TMC4 | -0.010801473 | 0.093871279 | 0.157054469 |
| CTSH | 0.012336459 | 0.093991719 | 0.1572038 |
| SPPL2A | 0.016662809 | 0.094224535 | 0.157505211 |
| RAB17 | -0.0113059 | 0.094234421 | 0.157505211 |
| YIPF6 | -0.009563947 | 0.094440365 | 0.15779711 |
| MZT2B | -0.021859312 | 0.095188379 | 0.15899424 |
| SNX18 | -0.007711823 | 0.095395322 | 0.159287121 |
| RPL26 | 0.045754344 | 0.095558547 | 0.159506833 |
| PRPF40B | 0.007213667 | 0.096312453 | 0.160712041 |
| AC027682.6 | -0.007817056 | 0.096440048 | 0.160871702 |
| WDR26 | 0.009576087 | 0.096848686 | 0.16149991 |
| TMEM87A | 0.009574605 | 0.097594492 | 0.16268976 |
| ELOVL7 | 0.007353032 | 0.097692823 | 0.162799841 |
| LAMP2 | 0.013822015 | 0.098182338 | 0.163561522 |
| SH3D21 | 0.007665381 | 0.099110177 | 0.165007963 |
| TOMM70 | 0.007949242 | 0.09911607 | 0.165007963 |
| HINT2 | -0.018748123 | 0.099480071 | 0.165559273 |
| MGST1 | 0.015316379 | 0.099643688 | 0.165776841 |
| EEF1G | -0.026747107 | 0.099735467 | 0.16587479 |
| TMCO3 | -0.008934337 | 0.099794001 | 0.1659174 |
| ANO6 | 0.010892424 | 0.100253079 | 0.166625708 |
| C15orf40 | -0.008406943 | 0.100832411 | 0.16753335 |
| TMEM128 | 0.007763591 | 0.100955721 | 0.167682963 |
| SLC38A2 | 0.010085792 | 0.101423703 | 0.168404772 |
| SHTN1 | 0.008054479 | 0.10222066 | 0.169672159 |
| PAIP1 | -0.009347139 | 0.102538119 | 0.170143073 |
| EXOSC4 | -0.007644343 | 0.103355288 | 0.171442582 |
| DVL3 | -0.008209586 | 0.103652354 | 0.171878788 |
| RNF40 | -0.008818674 | 0.103852234 | 0.172153605 |
| RTN4 | 0.023625988 | 0.103908237 | 0.172189817 |
| POM121C | -0.007679809 | 0.104168754 | 0.1725648 |
| UXT | 0.013480451 | 0.104771161 | 0.173505723 |
| USP9X | 0.011119696 | 0.104887265 | 0.173640953 |
| CTNNA1 | 0.015164045 | 0.105483727 | 0.174571065 |
| AMZ2 | -0.007210088 | 0.105707299 | 0.174883655 |
| GNB1 | -0.011808001 | 0.105756007 | 0.174906835 |
| NHLRC3 | 0.011296502 | 0.105926265 | 0.175130961 |
| USP13 | 0.009164914 | 0.106083179 | 0.175332888 |
| ATXN2L | -0.007563396 | 0.106377915 | 0.175762395 |

| | | | |
|---|---|---|---|
| SSR1 | -0.012437849 | 0.106547394 | 0.175927108 |
| RPS7 | -0.034975236 | 0.106556369 | 0.175927108 |
| TBC1D14 | -0.008208137 | 0.106582303 | 0.175927108 |
| ZSWIM8 | 0.007355806 | 0.106874126 | 0.176351053 |
| SLITRK4 | -0.009852042 | 0.107148346 | 0.176745684 |
| DNMBP | 0.007034812 | 0.10740756 | 0.177115313 |
| FH | -0.009852296 | 0.107720885 | 0.177573898 |
| AC025159.1 | 0.008914075 | 0.108377204 | 0.178597412 |
| LETMD1 | -0.010447605 | 0.108512008 | 0.178761122 |
| UQCRC2 | -0.018146691 | 0.10896601 | 0.179450394 |
| ACVR1B | -0.012165086 | 0.109054032 | 0.1795367 |
| EIF4B | -0.016974568 | 0.109092559 | 0.179541493 |
| C6orf89 | -0.009515849 | 0.109180725 | 0.179627948 |
| MAVS | -0.008239347 | 0.110172552 | 0.181200598 |
| BCR | -0.008187371 | 0.110423801 | 0.181554592 |
| ZNF655 | -0.010190274 | 0.110552526 | 0.181706972 |
| TIMM8B | -0.015598779 | 0.110595457 | 0.181718285 |
| ADAM9 | -0.00729867 | 0.110844844 | 0.182034078 |
| PIGK | 0.007699158 | 0.110859873 | 0.182034078 |
| AC027031.2 | 0.007786297 | 0.111200911 | 0.18248957 |
| ATP6V0B | -0.024530368 | 0.111213219 | 0.18248957 |
| SH3YL1 | -0.011213592 | 0.111258869 | 0.18248957 |
| AAK1 | 0.013260682 | 0.111282074 | 0.18248957 |
| ATP5MF | -0.024135409 | 0.111551235 | 0.182869318 |
| RPS4X | -0.038970947 | 0.111586198 | 0.182869318 |
| PSMD2 | -0.009782051 | 0.111833403 | 0.183182733 |
| KDM5B | 0.007186426 | 0.111871652 | 0.183182733 |
| ARL3 | 0.012888324 | 0.111886458 | 0.183182733 |
| SNRNP200 | -0.010507683 | 0.112376974 | 0.183926081 |
| HOXC-AS3 | 0.007587343 | 0.11255004 | 0.184149547 |
| PTPN18 | -0.007128841 | 0.112823351 | 0.18453683 |
| VAPB | -0.007811526 | 0.112950366 | 0.184684657 |
| LONP2 | 0.010561636 | 0.113166142 | 0.184977471 |
| CCDC115 | -0.008561965 | 0.113216562 | 0.184999899 |
| CDC42SE2 | 0.008596398 | 0.113280454 | 0.185044319 |
| C5orf38 | -0.010361389 | 0.113383851 | 0.185153221 |
| PRKCSH | -0.01108519 | 0.113711461 | 0.185628068 |
| ATF2 | 0.008617159 | 0.113985558 | 0.18601332 |
| RNF149 | 0.007914091 | 0.114021258 | 0.18601332 |
| RASSF7 | -0.013123803 | 0.114104408 | 0.186088748 |
| MAN1B1 | -0.006867483 | 0.114535912 | 0.186708651 |
| HSPB11 | 0.008609396 | 0.114584597 | 0.186708651 |
| DNAJC15 | -0.010648729 | 0.114595629 | 0.186708651 |
| TUSC2 | -0.008945762 | 0.114841107 | 0.187021335 |
| AC008124.1 | -0.007870778 | 0.114880835 | 0.187021335 |
| ITPRID2 | -0.011674353 | 0.114898844 | 0.187021335 |
| BBS1 | -0.007041209 | 0.11500275 | 0.18713004 |

| | | | |
|---|---|---|---|
| JAGN1 | -0.007586344 | 0.115481154 | 0.187847853 |
| CHMP4A | -0.007549216 | 0.115906783 | 0.188479385 |
| PPA1 | -0.009861642 | 0.115987013 | 0.188549026 |
| YIPF3 | 0.012186264 | 0.11624272 | 0.188903788 |
| ADNP | 0.01058219 | 0.116304165 | 0.188942731 |
| DGUOK | -0.008643292 | 0.116366608 | 0.188983271 |
| VLDLR | 0.008943836 | 0.116573631 | 0.18925851 |
| PGRMC2 | -0.009610052 | 0.116650497 | 0.189322329 |
| EPN2 | -0.007013266 | 0.116780205 | 0.189471842 |
| KRAS | -0.008802898 | 0.117194437 | 0.19008274 |
| MRPL1 | 0.010494858 | 0.117296122 | 0.190186474 |
| BABAM1 | -0.008136045 | 0.117569143 | 0.190567861 |
| GLB1 | 0.009724184 | 0.117693916 | 0.190708784 |
| THUMPD3-AS1 | -0.010536579 | 0.117988929 | 0.191125382 |
| DMTN | -0.009038841 | 0.118425099 | 0.191770293 |
| TMEM126A | -0.007628314 | 0.118788991 | 0.192297786 |
| DNAJC11 | -0.012427404 | 0.118852827 | 0.192339358 |
| STEAP2 | 0.007260197 | 0.118964697 | 0.192458613 |
| TMED3 | -0.008287544 | 0.119276001 | 0.192900327 |
| SYS1 | -0.007431072 | 0.119723105 | 0.193561312 |
| LITAF | -0.015542458 | 0.120140771 | 0.194174295 |
| AC027644.3 | 0.009194856 | 0.12019151 | 0.194194039 |
| AP5M1 | -0.009698639 | 0.120602035 | 0.194794892 |
| RO60 | -0.009276186 | 0.120697453 | 0.194886566 |
| PIK3C2A | 0.011769541 | 0.121013847 | 0.195334871 |
| LONP1 | -0.008210868 | 0.121261787 | 0.19567243 |
| PDCD10 | 0.008968539 | 0.121462617 | 0.195933777 |
| METAP2 | -0.010492497 | 0.121568419 | 0.196041715 |
| DANCR | -0.017044713 | 0.121690617 | 0.196151112 |
| SMPD1 | -0.007292949 | 0.12171408 | 0.196151112 |
| AARS | -0.007785349 | 0.122011847 | 0.196568143 |
| MRPS17 | -0.007600526 | 0.122426817 | 0.19717367 |
| SFT2D1 | 0.013542467 | 0.122562585 | 0.197329284 |
| ZDHHC12 | 0.0072967 | 0.12266847 | 0.197436704 |
| RAB5A | 0.008515083 | 0.122747803 | 0.197501333 |
| SLC25A3 | -0.024089566 | 0.123209124 | 0.198180342 |
| CRIPT | 0.008852193 | 0.123367676 | 0.198372075 |
| XRCC5 | 0.013035544 | 0.123612159 | 0.198701816 |
| LYRM7 | -0.007206748 | 0.123667045 | 0.198726673 |
| IPO5 | -0.009283443 | 0.123757108 | 0.198791948 |
| RIDA | -0.008646564 | 0.123786535 | 0.198791948 |
| CORO1B | -0.007262102 | 0.123842883 | 0.198819099 |
| RPL35 | 0.030971963 | 0.124494194 | 0.199801092 |
| REX1BD | 0.013396703 | 0.124587505 | 0.19988721 |
| NAGLU | -0.007471249 | 0.125495364 | 0.201249624 |
| USP53 | 0.0121615 | 0.125516528 | 0.201249624 |
| SRFBP1 | 0.007060843 | 0.125686972 | 0.201458832 |

| | | | |
|---|---|---|---|
| HIGD2A | -0.015744544 | 0.12586645 | 0.201682383 |
| RAB7A | -0.011295685 | 0.125948047 | 0.201749 |
| BBS2 | 0.007800782 | 0.126051787 | 0.201851035 |
| SND1 | 0.00902073 | 0.126137108 | 0.201923519 |
| DBNL | -0.007069301 | 0.126187671 | 0.201940333 |
| NPEPL1 | 0.008284517 | 0.126410425 | 0.202163729 |
| RIC3 | -0.009566743 | 0.126418699 | 0.202163729 |
| TKT | 0.008984105 | 0.126457614 | 0.202163729 |
| MBD2 | -0.00800864 | 0.126487681 | 0.202163729 |
| HEBP1 | -0.009472079 | 0.126589805 | 0.202262824 |
| H1F0 | -0.010936827 | 0.126679982 | 0.202342772 |
| CAPRIN1 | -0.011401738 | 0.126820357 | 0.202502825 |
| RAB11FIP1 | -0.007916077 | 0.127211891 | 0.203063693 |
| TMEM222 | -0.007466017 | 0.127258206 | 0.20307332 |
| AKIRIN2 | 0.006838174 | 0.127837209 | 0.203932712 |
| GFM1 | -0.008174772 | 0.127906604 | 0.203978865 |
| PDCD6 | -0.011553719 | 0.128237001 | 0.204395677 |
| RTRAF | -0.011749847 | 0.128249063 | 0.204395677 |
| ITM2C | -0.017537212 | 0.128599582 | 0.204889537 |
| TAF7 | -0.010601126 | 0.128732094 | 0.20501806 |
| ERGIC2 | 0.008526663 | 0.128761591 | 0.20501806 |
| EIF1B | -0.014291603 | 0.129146071 | 0.205565312 |
| THAP4 | -0.011703919 | 0.129283911 | 0.205719759 |
| EIF4A1 | -0.01287877 | 0.129329593 | 0.205727509 |
| VBP1 | -0.007735157 | 0.129453503 | 0.205859656 |
| ARFGAP2 | -0.008357771 | 0.129583561 | 0.206001492 |
| AC005261.1 | -0.007245114 | 0.129644801 | 0.206033872 |
| FAM168A | 0.006704747 | 0.129757712 | 0.206083403 |
| YPEL5 | -0.008482785 | 0.129757731 | 0.206083403 |
| LEPROT | -0.014832086 | 0.129892639 | 0.206149751 |
| ALDH1A1 | 0.019761274 | 0.129909792 | 0.206149751 |
| LARS | 0.011615657 | 0.12992219 | 0.206149751 |
| OXLD1 | -0.00730684 | 0.130335424 | 0.206740363 |
| HOOK3 | 0.008271343 | 0.130380516 | 0.206746833 |
| ZNF146 | 0.007261023 | 0.130577778 | 0.206994521 |
| SMIM26 | -0.013776129 | 0.130652212 | 0.207047407 |
| TPD52 | 0.011412335 | 0.130695229 | 0.207050487 |
| PSMD1 | -0.008394556 | 0.130834455 | 0.207205934 |
| PPP4C | -0.009303998 | 0.130881393 | 0.207215171 |
| NELFB | -0.007318033 | 0.130979166 | 0.207278149 |
| AHNAK | 0.013925478 | 0.131003409 | 0.207278149 |
| SLC43A1 | -0.007413404 | 0.131213847 | 0.207545969 |
| DCTN2 | -0.00833598 | 0.131304469 | 0.207624161 |
| MED25 | -0.006677397 | 0.131515134 | 0.207892063 |
| NAE1 | 0.007197017 | 0.131631713 | 0.208011118 |
| LGALS3 | -0.010346828 | 0.131952161 | 0.20845216 |
| NEDD8 | -0.016592588 | 0.132264576 | 0.208880241 |

| | | | |
|---|---|---|---|
| NBR1 | -0.008947757 | 0.133117041 | 0.210160665 |
| C18orf32 | -0.017897455 | 0.133763836 | 0.211115685 |
| GOPC | 0.007366973 | 0.133822839 | 0.211142702 |
| THAP5 | -0.007226221 | 0.133963866 | 0.211299076 |
| SGMS2 | 0.008651939 | 0.134300878 | 0.211764382 |
| DHPS | 0.007311178 | 0.13447894 | 0.211978842 |
| PMVK | -0.012033917 | 0.134626895 | 0.212145725 |
| COA5 | -0.009653524 | 0.135142489 | 0.212891653 |
| SDHB | -0.011837913 | 0.135204881 | 0.2129234 |
| POLR2J | -0.012891992 | 0.135407051 | 0.213175185 |
| VPS36 | -0.009795586 | 0.135740097 | 0.213632792 |
| NUDCD3 | 0.007745444 | 0.135882601 | 0.213790322 |
| PSME1 | 0.011292877 | 0.135976163 | 0.213870776 |
| COPB2 | 0.0088026 | 0.136363361 | 0.214412883 |
| MAGI2-AS3 | -0.007588573 | 0.136599477 | 0.21471717 |
| EMC3 | 0.009034808 | 0.136811379 | 0.214983218 |
| ALDH6A1 | -0.014705781 | 0.13693332 | 0.21510778 |
| LUC7L3 | 0.016871188 | 0.13774472 | 0.216314995 |
| SLBP | -0.006734743 | 0.137838575 | 0.216394973 |
| CEP57 | 0.00682607 | 0.137941155 | 0.216443236 |
| AC245297.3 | 0.007276927 | 0.137960206 | 0.216443236 |
| AC010913.1 | 0.007741781 | 0.137998128 | 0.216443236 |
| STMN1 | 0.011806333 | 0.13833379 | 0.2168248 |
| MDM4 | 0.012435396 | 0.138357254 | 0.2168248 |
| PPM1B | 0.007354804 | 0.138370439 | 0.2168248 |
| HAX1 | -0.010182897 | 0.138437493 | 0.216862462 |
| ZMPSTE24 | 0.008744662 | 0.138529176 | 0.21693867 |
| ZSWIM7 | -0.007991216 | 0.138898299 | 0.217449169 |
| DIP2A | 0.007091719 | 0.139031777 | 0.217590558 |
| HBS1L | 0.006794398 | 0.139102157 | 0.217633139 |
| SCAMP3 | 0.007449858 | 0.139610268 | 0.218360335 |
| LRRK2 | 0.00802135 | 0.13988046 | 0.218715074 |
| NDFIP2 | 0.007979517 | 0.140392288 | 0.219447294 |
| NCOA6 | 0.006898305 | 0.140591513 | 0.219690581 |
| SMIM14 | -0.009572958 | 0.14150211 | 0.221044976 |
| PIGQ | -0.007789935 | 0.141729135 | 0.221331031 |
| MBOAT2 | 0.009713159 | 0.141780719 | 0.221343018 |
| SNHG32 | -0.009523983 | 0.141853155 | 0.22138754 |
| MED10 | 0.006813925 | 0.142118773 | 0.221733438 |
| ECHDC2 | 0.012139556 | 0.142310959 | 0.221964587 |
| PTK7 | -0.00735326 | 0.142360384 | 0.221972997 |
| PARL | 0.007657038 | 0.142472897 | 0.222079739 |
| ENSA | -0.011190367 | 0.142752919 | 0.222447439 |
| HNRNPU | -0.01734402 | 0.143283298 | 0.223204916 |
| IARS2 | -0.009922938 | 0.143855486 | 0.224027033 |
| MARVELD2 | 0.01041325 | 0.144831719 | 0.22547767 |
| DERL2 | -0.007609649 | 0.145142846 | 0.225892278 |

| | | | |
|---|---|---|---|
| TRIP11 | -0.011436212 | 0.145531056 | 0.22642656 |
| BIRC2 | 0.008517465 | 0.145943446 | 0.226998121 |
| UBA5 | 0.007860205 | 0.146133474 | 0.227193299 |
| ZC3H14 | 0.008360616 | 0.146159069 | 0.227193299 |
| CFAP298 | -0.007694495 | 0.146405875 | 0.227506787 |
| TCEAL3 | -0.009068243 | 0.146703226 | 0.227898602 |
| RPL29 | -0.030212863 | 0.147232453 | 0.228650276 |
| MBIP | 0.006613195 | 0.147477028 | 0.228959563 |
| HNRNPUL2 | -0.008508963 | 0.147543055 | 0.228991545 |
| KIF3A | 0.006557455 | 0.147662255 | 0.229106011 |
| PACSIN2 | -0.007580175 | 0.148248658 | 0.229945073 |
| MLH3 | 0.007287239 | 0.148561396 | 0.23022696 |
| TFG | -0.00965986 | 0.148561889 | 0.23022696 |
| AC007405.3 | -0.007106271 | 0.148567407 | 0.23022696 |
| PPTC7 | -0.007439594 | 0.148733307 | 0.230413214 |
| GLRX3 | -0.006965637 | 0.149408263 | 0.231387728 |
| HSPD1 | -0.017279724 | 0.149480015 | 0.231406908 |
| RAD21 | -0.010772157 | 0.149512458 | 0.231406908 |
| ARMC10 | -0.006607797 | 0.1497125 | 0.231645399 |
| MRPS9 | 0.00804072 | 0.149775478 | 0.231671735 |
| EIF2B5 | -0.006751004 | 0.150304745 | 0.232419085 |
| UBE2A | -0.007422797 | 0.150483437 | 0.232624043 |
| WBP11 | -0.007964802 | 0.150624638 | 0.23269993 |
| ROMO1 | -0.015937093 | 0.150624851 | 0.23269993 |
| TRAPPC1 | -0.011114959 | 0.150778029 | 0.23286521 |
| NIPSNAP1 | -0.006710316 | 0.150885375 | 0.232959625 |
| DNAJA1 | -0.011183429 | 0.15137804 | 0.233648714 |
| ZNF652 | 0.007752597 | 0.152225909 | 0.234830616 |
| PSMD14 | 0.007168594 | 0.152236948 | 0.234830616 |
| EPN1 | -0.013922964 | 0.152696907 | 0.235468065 |
| VPS29 | -0.010437263 | 0.153659982 | 0.236880725 |
| ARFGEF2 | 0.008823441 | 0.153758309 | 0.23695984 |
| BCL2L11 | 0.007124205 | 0.153899018 | 0.237104202 |
| NOP53 | -0.013221118 | 0.154108222 | 0.237353971 |
| USP11 | 0.009463618 | 0.154261633 | 0.237510578 |
| ARID1A | 0.009824112 | 0.154304135 | 0.237510578 |
| CTCF | -0.007011843 | 0.15436633 | 0.237533782 |
| EXOC5 | -0.00630976 | 0.155023507 | 0.23847223 |
| CDIP1 | -0.006511808 | 0.155078015 | 0.238475916 |
| ITGA6 | -0.009953553 | 0.155120517 | 0.238475916 |
| RORC | 0.006993865 | 0.155254863 | 0.238609684 |
| SNHG5 | -0.009585373 | 0.155552642 | 0.238939958 |
| AHSA1 | -0.007596747 | 0.155564559 | 0.238939958 |
| GAMT | 0.007829469 | 0.155615361 | 0.238945182 |
| GGA1 | -0.006761258 | 0.155689939 | 0.238986901 |
| PABPC4 | -0.008602789 | 0.155965414 | 0.239281048 |
| PRPF38B | -0.007951709 | 0.155976498 | 0.239281048 |

| | | | |
|---|---|---|---|
| TMED1 | -0.008716634 | 0.156139952 | 0.239453263 |
| SDHAF3 | -0.00850653 | 0.15618823 | 0.239453263 |
| RHOA | -0.013013273 | 0.15623126 | 0.239453263 |
| AASS | 0.007382764 | 0.156845777 | 0.240322056 |
| ATRAID | 0.01412004 | 0.157012503 | 0.240502839 |
| RPS6 | 0.033244753 | 0.157059184 | 0.240502839 |
| MAGOH | -0.008505773 | 0.157281568 | 0.240770236 |
| CIAPIN1 | -0.006520655 | 0.157445341 | 0.240947772 |
| MRFAP1L1 | -0.00685014 | 0.157876322 | 0.241534003 |
| ERH | 0.01535748 | 0.158088136 | 0.241784676 |
| EIF6 | -0.010128382 | 0.158198012 | 0.241879338 |
| SC5D | 0.007163093 | 0.158299574 | 0.241961236 |
| TMEM47 | -0.006834651 | 0.158517419 | 0.242220767 |
| MED7 | 0.00692477 | 0.158822647 | 0.242613625 |
| SMU1 | 0.007047869 | 0.15933446 | 0.243321725 |
| PHF20L1 | 0.008374329 | 0.159996492 | 0.244258727 |
| CGGBP1 | 0.009833435 | 0.160139925 | 0.244403681 |
| SPECC1L | 0.006849731 | 0.160278959 | 0.244541837 |
| SRP19 | -0.00765924 | 0.160518705 | 0.244833522 |
| TRAPPC4 | -0.007358214 | 0.160610088 | 0.244898806 |
| RBM28 | -0.006910743 | 0.160893177 | 0.245256277 |
| GTF2A2 | -0.010248246 | 0.161310073 | 0.245817435 |
| BAG5 | -0.00716714 | 0.161970685 | 0.246749539 |
| NAA38 | -0.017983521 | 0.162407862 | 0.247340795 |
| ECHS1 | 0.014520093 | 0.163183301 | 0.248360472 |
| PDHA1 | -0.013568282 | 0.163215329 | 0.248360472 |
| DTD1 | 0.006139824 | 0.163225202 | 0.248360472 |
| PAPSS2 | 0.009051462 | 0.163444987 | 0.248619849 |
| SNRNP35 | -0.006233268 | 0.16394321 | 0.249302481 |
| PRPSAP1 | 0.008324087 | 0.164229145 | 0.249661978 |
| MRAS | -0.006247955 | 0.164473917 | 0.249958702 |
| FOLR3 | -0.012151198 | 0.164719014 | 0.250255742 |
| ILK | 0.006958321 | 0.164772461 | 0.250261517 |
| LINC01116 | -0.007579753 | 0.164899791 | 0.250379472 |
| TMEM192 | -0.006947355 | 0.165187362 | 0.250740587 |
| PEF1 | -0.006548073 | 0.16543539 | 0.251041482 |
| QRSL1 | 0.006189785 | 0.165597538 | 0.251211914 |
| GDI1 | -0.0070002 | 0.165701631 | 0.2512942 |
| SNRPD3 | -0.011090258 | 0.165857867 | 0.25145549 |
| ATP5ME | 0.028856742 | 0.166609851 | 0.252519621 |
| COMTD1 | 0.011683644 | 0.166739207 | 0.252639718 |
| TFDP1 | -0.006104071 | 0.166852223 | 0.252734993 |
| CAMTA1 | -0.013697228 | 0.167001369 | 0.252858718 |
| UBL3 | 0.010267704 | 0.167034226 | 0.252858718 |
| GATAD2A | -0.006124686 | 0.16772962 | 0.253783182 |
| PLS3 | -0.010654509 | 0.167745598 | 0.253783182 |
| CSNK1D | -0.006852801 | 0.167803872 | 0.253795175 |

| | | | |
|---|---|---|---|
| LSM6 | -0.007571352 | 0.167891319 | 0.253851272 |
| RPS15 | 0.032141096 | 0.168008787 | 0.253952712 |
| WBP1 | -0.009271041 | 0.168241886 | 0.254228821 |
| FAAP20 | -0.009674675 | 0.168562874 | 0.254563553 |
| SLC39A7 | -0.008261837 | 0.1685644 | 0.254563553 |
| ATL2 | 0.00740508 | 0.168661335 | 0.25463366 |
| ALDOA | 0.00675973 | 0.169051689 | 0.255146577 |
| GTF2F1 | 0.006282315 | 0.169198952 | 0.255292403 |
| DDRGK1 | 0.008440775 | 0.169500317 | 0.255670586 |
| ZFC3H1 | 0.006491636 | 0.170165841 | 0.256597668 |
| OSTC | 0.010997631 | 0.170406619 | 0.256883901 |
| ZNF451 | 0.007487418 | 0.170575089 | 0.257060994 |
| UGCG | 0.006018152 | 0.170911097 | 0.257433535 |
| TUBGCP2 | 0.00790146 | 0.170924428 | 0.257433535 |
| DPP4 | -0.006486928 | 0.171474649 | 0.258185098 |
| CREM | 0.007122402 | 0.171701157 | 0.25844895 |
| SLC39A1 | -0.007106153 | 0.172754973 | 0.259957558 |
| CLCN7 | -0.007594535 | 0.172892221 | 0.260086447 |
| INO80E | -0.005997517 | 0.172958543 | 0.260108597 |
| SLC66A2 | -0.006148643 | 0.173625243 | 0.261033359 |
| NUDT14 | -0.008748836 | 0.173759297 | 0.261103692 |
| GAD1 | 0.007532328 | 0.173775618 | 0.261103692 |
| SPTAN1 | -0.009660612 | 0.173877875 | 0.261179489 |
| DCTN3 | 0.010656967 | 0.174060958 | 0.261376613 |
| REST | 0.008637159 | 0.174154571 | 0.261439307 |
| HOXA5 | 0.00818025 | 0.174233255 | 0.261479559 |
| PRELID1 | -0.011411908 | 0.174509499 | 0.261816186 |
| KHDC4 | 0.006674121 | 0.174623121 | 0.261848914 |
| ANKRD11 | -0.009110969 | 0.174635201 | 0.261848914 |
| DEPTOR | 0.010486645 | 0.175107724 | 0.262450054 |
| INO80D | 0.006795116 | 0.175140246 | 0.262450054 |
| RNH1 | -0.010700598 | 0.175975676 | 0.263623591 |
| AP1M2 | -0.008363241 | 0.176333108 | 0.264080569 |
| TCTEX1D2 | 0.006868299 | 0.176747942 | 0.264623218 |
| LAMB2 | 0.00784552 | 0.177101664 | 0.265074076 |
| BDP1 | -0.008483878 | 0.177567037 | 0.265691728 |
| ST13 | 0.016357415 | 0.178151783 | 0.266487577 |
| SRSF1 | -0.011279122 | 0.178238309 | 0.266537917 |
| HEIH | -0.006508414 | 0.178870869 | 0.267404523 |
| MIR4458HG | -0.010055328 | 0.179095657 | 0.267661194 |
| POLE4 | -0.006970486 | 0.179342434 | 0.267932059 |
| MPLKIP | -0.008323958 | 0.179383198 | 0.267932059 |
| PLPP3 | -0.008559036 | 0.179506786 | 0.268037235 |
| C11orf58 | -0.01231282 | 0.179570344 | 0.26805274 |
| SRI | -0.01289684 | 0.180481051 | 0.269332438 |
| GSTA1 | 0.010272166 | 0.180766046 | 0.26960924 |
| MIER1 | 0.007733416 | 0.180773503 | 0.26960924 |

| | | | |
|---|---|---|---|
| CNIH4 | 0.007974598 | 0.181228754 | 0.270208267 |
| U2AF2 | 0.006991873 | 0.182239201 | 0.271634481 |
| ATP1A1 | -0.044167339 | 0.182373906 | 0.27175491 |
| OAZ2 | -0.007046806 | 0.183264556 | 0.273001367 |
| TRAPPC6A | 0.009923774 | 0.183443161 | 0.273123142 |
| CEBPZ | -0.008650553 | 0.183454664 | 0.273123142 |
| RBM8A | 0.009493849 | 0.183661292 | 0.273350036 |
| PRMT1 | 0.008798005 | 0.183740004 | 0.27338647 |
| PSME3 | -0.005998945 | 0.183934603 | 0.273595259 |
| MRPL2 | 0.009338749 | 0.184064477 | 0.273707678 |
| WBP2 | -0.008898024 | 0.184448259 | 0.274197485 |
| AGFG1 | 0.007024011 | 0.184519238 | 0.274222135 |
| HSBP1L1 | -0.007335663 | 0.185650609 | 0.275822198 |
| TOR1AIP2 | -0.008339319 | 0.185739271 | 0.275872618 |
| CRLS1 | 0.00655255 | 0.185840614 | 0.275941836 |
| NR2F1 | 0.006743292 | 0.186809308 | 0.277298504 |
| EXOC3 | -0.00750882 | 0.187220449 | 0.277826989 |
| BOD1L1 | -0.009068934 | 0.187337915 | 0.277909925 |
| CDC37L1 | 0.005885858 | 0.187386597 | 0.277909925 |
| PTGES2 | -0.008739053 | 0.187745632 | 0.278360509 |
| EPM2AIP1 | -0.008654702 | 0.187873294 | 0.278467885 |
| DMKN | -0.010832307 | 0.187987467 | 0.278555209 |
| MARCH5 | -0.007119677 | 0.188085267 | 0.278618228 |
| CMC2 | -0.009009827 | 0.188604614 | 0.279305481 |
| POLR2F | 0.008635806 | 0.188803695 | 0.279518188 |
| PPIA | -0.025548538 | 0.189218467 | 0.280049998 |
| VAPA | -0.014462057 | 0.189574271 | 0.280421281 |
| EXOC7 | -0.007034172 | 0.189580585 | 0.280421281 |
| WDR82 | 0.006341199 | 0.189695102 | 0.280508363 |
| SF3B6 | -0.013486818 | 0.189909324 | 0.280742786 |
| DPM3 | 0.01336393 | 0.19012025 | 0.280972201 |
| PHB | -0.013549731 | 0.190185404 | 0.280986114 |
| ZFPL1 | 0.005943712 | 0.190605619 | 0.281524443 |
| COMMD6 | -0.019538261 | 0.190671267 | 0.281538915 |
| SREK1 | 0.009361639 | 0.191165125 | 0.282185474 |
| BLOC1S2 | -0.00611012 | 0.191867587 | 0.283120798 |
| ANXA4 | 0.01052773 | 0.191927159 | 0.283120798 |
| PSMB3 | 0.011096159 | 0.191967246 | 0.283120798 |
| BDH1 | 0.007611087 | 0.192306959 | 0.283538866 |
| PFKFB2 | -0.006169468 | 0.19285412 | 0.284255906 |
| SUDS3 | -0.007289665 | 0.192906061 | 0.284255906 |
| REXO2 | -0.01061369 | 0.193150389 | 0.284532762 |
| PEX1 | 0.006545791 | 0.19331751 | 0.284695754 |
| NUB1 | 0.006490308 | 0.193611911 | 0.285046041 |
| EEF1D | 0.018043314 | 0.193979005 | 0.285475929 |
| TMEM30A | -0.009850556 | 0.194031357 | 0.285475929 |
| PI4KB | 0.006454412 | 0.194073797 | 0.285475929 |

| | | | |
|---|---|---|---|
| YIF1A | 0.008176017 | 0.194615365 | 0.286189048 |
| ATP6V1C1 | 0.006383885 | 0.194748402 | 0.286301165 |
| UHRF2 | 0.006018664 | 0.194940766 | 0.286500408 |
| HIP1R | -0.008149397 | 0.195077824 | 0.286618278 |
| AUP1 | 0.007573662 | 0.195275016 | 0.286824405 |
| ASAH1 | -0.011439876 | 0.196005868 | 0.287814034 |
| PABPC1 | 0.013499925 | 0.196205495 | 0.288023267 |
| CTDSPL | 0.011404967 | 0.19674324 | 0.28872858 |
| CALCOCO1 | 0.006589959 | 0.196935119 | 0.288926057 |
| TSFM | 0.00675928 | 0.197405163 | 0.289531402 |
| MYO15B | 0.005688523 | 0.19815725 | 0.290513194 |
| SFPQ | 0.01422449 | 0.198209439 | 0.290513194 |
| SLC7A8 | -0.007695515 | 0.198247448 | 0.290513194 |
| CD59 | -0.020702015 | 0.198740103 | 0.291096688 |
| DMAC1 | -0.011933139 | 0.198761119 | 0.291096688 |
| CRY2 | -0.005559129 | 0.199032009 | 0.291408759 |
| GOT2 | -0.008628845 | 0.20024072 | 0.293093342 |
| SSB | -0.009726083 | 0.200570358 | 0.293490616 |
| TMEM141 | -0.016910893 | 0.201885374 | 0.295329126 |
| POLR2E | -0.008132083 | 0.201984456 | 0.29538835 |
| H2AFJ | 0.008483111 | 0.202674935 | 0.296312166 |
| MYL6B | -0.011431283 | 0.202895716 | 0.296548944 |
| DYNC1H1 | -0.010079011 | 0.202978201 | 0.29658351 |
| SDC2 | 0.0081789 | 0.203706689 | 0.297561698 |
| TJP1 | 0.006491399 | 0.203967228 | 0.297855966 |
| LYRM2 | -0.008244051 | 0.204270748 | 0.29818924 |
| CBR1 | -0.017065013 | 0.204313754 | 0.29818924 |
| RCHY1 | 0.006541409 | 0.204555229 | 0.298455256 |
| SLC38A1 | -0.00713943 | 0.204724804 | 0.298616244 |
| KIF13B | 0.007420364 | 0.204826423 | 0.298678045 |
| GLUL | -0.009752183 | 0.205363461 | 0.299336666 |
| DDX17 | 0.02232773 | 0.205396851 | 0.299336666 |
| TRMU | 0.005799286 | 0.205796254 | 0.299832056 |
| RAB30-DT | 0.006893567 | 0.205981444 | 0.300015158 |
| ILKAP | 0.005799568 | 0.206146954 | 0.300169496 |
| ALDH7A1 | 0.007582973 | 0.206616733 | 0.300766662 |
| MRPS18C | -0.009067381 | 0.206853209 | 0.301023968 |
| WASHC1 | 0.005651929 | 0.206950934 | 0.301064722 |
| HADH | -0.009124593 | 0.20700066 | 0.301064722 |
| NME3 | 0.012812575 | 0.207261083 | 0.301356539 |
| OGT | 0.009031904 | 0.207542426 | 0.301678596 |
| SLCO3A1 | 0.006023444 | 0.208545762 | 0.303049636 |
| EBAG9 | -0.007005656 | 0.208676823 | 0.303152699 |
| IBTK | -0.007918096 | 0.208835929 | 0.303296433 |
| NOTCH3 | -0.007541205 | 0.209350566 | 0.303948693 |
| MINDY2 | -0.008571745 | 0.209405636 | 0.303948693 |
| ARHGEF18 | -0.006478607 | 0.209467709 | 0.303951273 |

| | | | |
|---|---|---|---|
| FDPS | 0.00834921 | 0.209889738 | 0.30447602 |
| RAB11FIP3 | -0.006149455 | 0.210617689 | 0.305444122 |
| TERF2IP | -0.008593895 | 0.210772019 | 0.305580025 |
| EIF3L | -0.010301927 | 0.211361491 | 0.306346544 |
| CDC42BPB | -0.00689108 | 0.211951015 | 0.307103731 |
| C8orf82 | -0.007804613 | 0.212021204 | 0.307103731 |
| NDUFC2 | 0.019841875 | 0.212066671 | 0.307103731 |
| SLC44A2 | -0.010979561 | 0.212363041 | 0.307444597 |
| IMPA1 | -0.005621304 | 0.212686162 | 0.307823986 |
| OTUD4 | 0.005333401 | 0.21312985 | 0.308377605 |
| RPAIN | -0.007429284 | 0.214404527 | 0.310132919 |
| HACD3 | 0.011207394 | 0.214482052 | 0.31015606 |
| CTSA | -0.009809908 | 0.215331387 | 0.311294959 |
| SH3BGRL | -0.011033259 | 0.215552673 | 0.311525523 |
| FBXO7 | 0.006979579 | 0.215657949 | 0.31158834 |
| MESD | -0.007186024 | 0.215974083 | 0.311943933 |
| CLTC | -0.012264659 | 0.216027826 | 0.311943933 |
| PSME2 | -0.008330329 | 0.216235741 | 0.312154746 |
| FAM192A | 0.007563004 | 0.21636337 | 0.312206407 |
| THUMPD1 | -0.008108638 | 0.216395395 | 0.312206407 |
| CDK4 | -0.005949808 | 0.216725292 | 0.312592903 |
| PDXDC1 | -0.007709007 | 0.216951412 | 0.312829539 |
| FLNB | 0.011051948 | 0.217075045 | 0.312889369 |
| DHX29 | 0.007103658 | 0.217117043 | 0.312889369 |
| CUL4A | -0.006060026 | 0.21779059 | 0.313770324 |
| HSP90B1 | 0.022543102 | 0.217857986 | 0.313777745 |
| AGPAT1 | 0.006640688 | 0.218031312 | 0.313937688 |
| EFEMP1 | 0.006092112 | 0.218300268 | 0.314235195 |
| PSMB2 | 0.007444464 | 0.219230587 | 0.315484268 |
| KHDRBS3 | 0.007908136 | 0.220233142 | 0.31683655 |
| BAZ1B | -0.006003079 | 0.2203669 | 0.316861097 |
| FOLR1 | 0.011887931 | 0.220396195 | 0.316861097 |
| ACTR10 | 0.008171454 | 0.220438775 | 0.316861097 |
| GNG11 | -0.007145141 | 0.220803143 | 0.317294369 |
| POLD2 | 0.006447389 | 0.220999905 | 0.317486612 |
| IK | 0.00743284 | 0.221241823 | 0.317743598 |
| SF1 | -0.011734776 | 0.221550049 | 0.318095642 |
| TNRC18 | 0.007494562 | 0.221786782 | 0.318344865 |
| HP1BP3 | -0.011730524 | 0.222319068 | 0.319018053 |
| B2M | -0.025592627 | 0.223150332 | 0.320119756 |
| ANAPC11 | -0.012202772 | 0.223750139 | 0.32088889 |
| GADD45A | -0.007803051 | 0.224510405 | 0.321887643 |
| DHX30 | -0.007372555 | 0.224751569 | 0.322141786 |
| RETREG3 | 0.006049352 | 0.225025347 | 0.322442517 |
| UBA1 | 0.011033377 | 0.225167431 | 0.322554424 |
| RAB40B | 0.006844132 | 0.225400468 | 0.322796522 |
| MCCC2 | -0.007871899 | 0.225478953 | 0.322817211 |

| | | | |
|---|---|---|---|
| PHF1 | -0.005347728 | 0.2257593 | 0.323126811 |
| ABHD2 | -0.006422693 | 0.226369698 | 0.323908501 |
| BOD1 | -0.00541965 | 0.226786637 | 0.324413008 |
| RNASET2 | 0.006986473 | 0.227460611 | 0.325284806 |
| ORMDL1 | -0.008360154 | 0.227841329 | 0.325736852 |
| PIN1 | -0.007494016 | 0.228812574 | 0.327032658 |
| MARF1 | 0.006816128 | 0.228973726 | 0.327170225 |
| TTC3 | -0.014280824 | 0.229128218 | 0.3272982 |
| EDEM3 | 0.005831678 | 0.229421476 | 0.327556707 |
| BNIP2 | 0.005536261 | 0.229439146 | 0.327556707 |
| SIKE1 | -0.005524118 | 0.229682548 | 0.32781136 |
| SNX4 | 0.006317163 | 0.229862107 | 0.327974776 |
| HAGH | -0.006629714 | 0.229935809 | 0.3279871 |
| TP53TG1 | -0.007279535 | 0.230215875 | 0.328293699 |
| ATP6V0E1 | -0.017285872 | 0.230632845 | 0.328762555 |
| STAU1 | -0.007232151 | 0.230675096 | 0.328762555 |
| EIF4A2 | -0.014849274 | 0.231090168 | 0.329237888 |
| TPMT | -0.006161207 | 0.231204106 | 0.329237888 |
| NDUFS3 | 0.011937699 | 0.231204548 | 0.329237888 |
| NCSTN | -0.006341967 | 0.23132779 | 0.329320359 |
| TNPO1 | 0.007433007 | 0.231422287 | 0.329361872 |
| HSPBP1 | 0.005386191 | 0.231896336 | 0.329887067 |
| RGN | -0.00589579 | 0.231922191 | 0.329887067 |
| POLR2L | -0.02005423 | 0.232228108 | 0.330148204 |
| HDAC1 | -0.006248306 | 0.232236765 | 0.330148204 |
| DGAT1 | -0.005572343 | 0.232592337 | 0.330560465 |
| DNAJB14 | 0.007496895 | 0.233092675 | 0.331178178 |
| PLXNB2 | -0.00892437 | 0.233416872 | 0.331545351 |
| EIF2S3 | -0.010033564 | 0.233653614 | 0.331730936 |
| TSC1 | 0.005597206 | 0.233679142 | 0.331730936 |
| MFAP3L | 0.008015454 | 0.234261463 | 0.332396472 |
| SHARPIN | 0.006360466 | 0.234279838 | 0.332396472 |
| PLA2G12A | -0.009281969 | 0.234524608 | 0.332650126 |
| TUBA4A | -0.005442549 | 0.235000525 | 0.333231405 |
| CCNL2 | 0.00776187 | 0.235532256 | 0.33389148 |
| TEX264 | -0.010408966 | 0.235888488 | 0.334302465 |
| HOOK2 | -0.006771607 | 0.236032059 | 0.334306067 |
| DERL1 | -0.005250287 | 0.236075475 | 0.334306067 |
| ERLIN2 | -0.007299779 | 0.236089982 | 0.334306067 |
| JPT2 | -0.006302309 | 0.237086047 | 0.335622231 |
| RNF146 | -0.005240272 | 0.237159717 | 0.335632267 |
| DNAJC13 | 0.005648444 | 0.237317664 | 0.335761534 |
| KARS | -0.006741369 | 0.237720912 | 0.336237688 |
| OFD1 | -0.006293666 | 0.238493645 | 0.337236034 |
| NFU1 | -0.006967076 | 0.238664105 | 0.337382432 |
| RYBP | 0.005991091 | 0.238750623 | 0.337410118 |
| PRRC1 | 0.005377864 | 0.239592443 | 0.338504907 |

| | | | |
|---|---|---|---|
| ADI1 | -0.013079827 | 0.239820419 | 0.338676139 |
| ABCD3 | 0.008843813 | 0.239848009 | 0.338676139 |
| WFDC2 | -0.014911571 | 0.240229147 | 0.33911933 |
| COX10 | 0.005234351 | 0.240665577 | 0.339640307 |
| NOSIP | 0.006646097 | 0.240781184 | 0.339708354 |
| PSMF1 | -0.005984179 | 0.241145553 | 0.340127234 |
| ZNF483 | 0.005523532 | 0.241263257 | 0.340198064 |
| DNAJB1 | -0.005466932 | 0.241447879 | 0.340363187 |
| HGS | -0.00553846 | 0.242048821 | 0.34111493 |
| RARS | 0.005934174 | 0.242142071 | 0.341150973 |
| ZSCAN16-AS1 | 0.010794471 | 0.242237804 | 0.341190491 |
| CYFIP2 | -0.020685673 | 0.242535946 | 0.341515001 |
| SF3B5 | -0.010856345 | 0.243437483 | 0.342688733 |
| SMIM4 | -0.010806933 | 0.243888809 | 0.343228221 |
| MYO6 | 0.010746093 | 0.244074186 | 0.343393237 |
| TIMM44 | -0.005649293 | 0.244234151 | 0.34352242 |
| MKRN1 | -0.007611126 | 0.244376986 | 0.343627444 |
| TMEM18 | 0.005569685 | 0.244712403 | 0.34400313 |
| SALL1 | 0.007810259 | 0.245595404 | 0.345148155 |
| POM121 | 0.005049625 | 0.24567378 | 0.345162075 |
| SEMA4D | -0.005223439 | 0.246190679 | 0.345791924 |
| UFL1 | 0.007141228 | 0.247009533 | 0.346845419 |
| ZDHHC2 | -0.007562268 | 0.247140011 | 0.346931995 |
| PAN2 | -0.005181146 | 0.247586042 | 0.347410169 |
| IMMT | -0.010621577 | 0.247618476 | 0.347410169 |
| SH3GLB1 | 0.007040764 | 0.24827762 | 0.348175223 |
| TCTA | -0.006130153 | 0.24830191 | 0.348175223 |
| SH3BP4 | -0.006747357 | 0.248430972 | 0.348259324 |
| HEXA | 0.009876121 | 0.24861656 | 0.348422596 |
| ANKH | 0.005422573 | 0.248848161 | 0.348650245 |
| ACAT1 | 0.014071362 | 0.250295791 | 0.350581018 |
| HES4 | 0.008095102 | 0.25064083 | 0.350966784 |
| TMEM52B | -0.031484638 | 0.252172979 | 0.352968064 |
| TLN1 | -0.005925692 | 0.252219465 | 0.352968064 |
| GOLGB1 | -0.010746275 | 0.25228009 | 0.352968064 |
| NDUFA2 | -0.015313712 | 0.252476028 | 0.353144188 |
| PCMT1 | 0.006516717 | 0.252627562 | 0.353258125 |
| NAV2 | 0.009946531 | 0.253645599 | 0.354583324 |
| MBP | 0.006063978 | 0.253840287 | 0.354703533 |
| ALKBH7 | -0.011614826 | 0.253872316 | 0.354703533 |
| RAB25 | -0.006837887 | 0.254058755 | 0.35484519 |
| TCTN3 | 0.005747798 | 0.254114488 | 0.35484519 |
| PSMA5 | -0.007299969 | 0.254519282 | 0.355312019 |
| NOL7 | -0.0087035 | 0.254756516 | 0.355544739 |
| LSM3 | -0.009801422 | 0.255050823 | 0.355788503 |
| JOSD2 | -0.005495967 | 0.255072337 | 0.355788503 |
| CCNY | 0.007300641 | 0.255143209 | 0.355788912 |

| | | | |
|---|---|---|---|
| ACOX1 | -0.006007925 | 0.255662485 | 0.356414433 |
| TXNDC5 | -0.005618773 | 0.255794742 | 0.356489297 |
| DUSP23 | -0.006835325 | 0.255857623 | 0.356489297 |
| APOE | -0.008375858 | 0.256148762 | 0.356796328 |
| FRA10AC1 | 0.005900163 | 0.256639319 | 0.357380887 |
| SH2B1 | 0.005382411 | 0.256990314 | 0.357770829 |
| NCOR2 | -0.006277382 | 0.25764714 | 0.358586205 |
| ARFGAP3 | -0.00605714 | 0.258119867 | 0.359108541 |
| LINC00665 | -0.005745944 | 0.258164918 | 0.359108541 |
| OBSL1 | 0.011809331 | 0.258354781 | 0.359273504 |
| ANXA7 | -0.008751637 | 0.258532334 | 0.359349451 |
| BLVRB | 0.009698836 | 0.258551965 | 0.359349451 |
| HOXA9 | -0.007462504 | 0.258777287 | 0.359563479 |
| SEPTIN6 | -0.006825646 | 0.259047848 | 0.359600707 |
| CHTOP | 0.005925558 | 0.259076292 | 0.359600707 |
| RPS4Y1 | 0.009470641 | 0.259088625 | 0.359600707 |
| SWI5 | 0.005281735 | 0.25908942 | 0.359600707 |
| TMEM160 | -0.009563378 | 0.259192615 | 0.359644914 |
| ATP6V0A4 | 0.009785892 | 0.259590584 | 0.360098001 |
| OSBPL8 | 0.006949327 | 0.25967003 | 0.360109111 |
| SF3A3 | -0.005022734 | 0.25996442 | 0.360418218 |
| RPL19 | -0.027283588 | 0.260710352 | 0.361331628 |
| SLC36A4 | 0.005808091 | 0.260822674 | 0.361331628 |
| MEPCE | -0.005009963 | 0.260892984 | 0.361331628 |
| ARL2 | -0.009098847 | 0.260909964 | 0.361331628 |
| VGLL4 | 0.007624044 | 0.2610329 | 0.361388556 |
| MFN1 | 0.005720277 | 0.26109445 | 0.361388556 |
| CPNE3 | 0.010031716 | 0.261357797 | 0.361653762 |
| MRPL32 | -0.007795812 | 0.263146456 | 0.364028892 |
| PSMC2 | -0.005855167 | 0.263247904 | 0.364069323 |
| DCBLD2 | 0.00521378 | 0.263338721 | 0.364095034 |
| APOO | -0.006621449 | 0.263597609 | 0.364353043 |
| C6orf62 | 0.008693981 | 0.264069791 | 0.364905651 |
| ZC3H15 | -0.007529343 | 0.264628641 | 0.365577687 |
| SNX5 | -0.00741473 | 0.264934961 | 0.365844647 |
| TRMT13 | 0.005269918 | 0.264967032 | 0.365844647 |
| FBXL3 | 0.00653057 | 0.265239261 | 0.36612024 |
| ATP5F1C | 0.016395477 | 0.265697695 | 0.366625 |
| ISYNA1 | 0.008667846 | 0.265750397 | 0.366625 |
| UROD | 0.005474373 | 0.265900269 | 0.366695817 |
| MRPL48 | 0.005363971 | 0.265947214 | 0.366695817 |
| COX8A | -0.024837284 | 0.26667058 | 0.36759267 |
| CELF1 | 0.008739636 | 0.266751332 | 0.367603462 |
| TM2D3 | -0.005606489 | 0.267379041 | 0.36836779 |
| PDZD8 | -0.005695642 | 0.2678361 | 0.368896661 |
| ITM2A | 0.005906511 | 0.268380339 | 0.369545285 |
| RAD23B | 0.005933213 | 0.268704073 | 0.369890014 |

| | | | |
|---|---|---|---|
| PFDN1 | 0.007052729 | 0.268871079 | 0.370018867 |
| FBXO17 | -0.004887926 | 0.268972325 | 0.370057176 |
| GINM1 | -0.009094384 | 0.269390528 | 0.37053142 |
| WDR6 | 0.007292455 | 0.27019643 | 0.371538517 |
| FIS1 | 0.013770136 | 0.27033394 | 0.371626231 |
| PATZ1 | -0.005856932 | 0.271529326 | 0.373011686 |
| DHRS7 | 0.007630746 | 0.271548409 | 0.373011686 |
| MED4 | -0.006259215 | 0.271563754 | 0.373011686 |
| PPT1 | 0.007526155 | 0.271756199 | 0.373174339 |
| ACAA2 | -0.009619658 | 0.271986748 | 0.373389215 |
| CLDND1 | 0.004801427 | 0.272099686 | 0.373442558 |
| LIN7C | -0.006812307 | 0.272185711 | 0.373458946 |
| RASSF8-AS1 | -0.007017346 | 0.272589481 | 0.373911176 |
| TPP1 | -0.005746099 | 0.272748642 | 0.374027722 |
| RAB34 | -0.006220587 | 0.274276837 | 0.376021087 |
| LPIN1 | -0.0058511 | 0.27478193 | 0.376611123 |
| LSM7 | -0.009082738 | 0.275732794 | 0.377811637 |
| UFSP2 | 0.006962703 | 0.275934102 | 0.37798473 |
| DDX21 | -0.005418486 | 0.276107493 | 0.378119498 |
| SLC11A2 | 0.004781158 | 0.276684335 | 0.378806554 |
| ADAL | 0.005694614 | 0.276805933 | 0.378870135 |
| RFC1 | 0.006685241 | 0.278003109 | 0.380405448 |
| STXBP3 | 0.006358724 | 0.278320906 | 0.380736958 |
| MMADHC | 0.006122581 | 0.279096351 | 0.381694168 |
| THRA | -0.005973901 | 0.27929542 | 0.381862818 |
| KDELR1 | -0.009948642 | 0.279437679 | 0.381922567 |
| TUFM | 0.011771416 | 0.279490646 | 0.381922567 |
| AP2M1 | -0.009725191 | 0.27977706 | 0.382210341 |
| RPS15A | 0.024132394 | 0.28051964 | 0.383120971 |
| PRXL2A | -0.012499758 | 0.280969278 | 0.38363113 |
| NDRG1 | -0.009274621 | 0.281639426 | 0.384442011 |
| SMS | -0.012548802 | 0.281908259 | 0.3847048 |
| CHCHD5 | -0.008111482 | 0.282411658 | 0.38528746 |
| DCTD | 0.005216248 | 0.282581205 | 0.385414462 |
| METTL26 | -0.008387414 | 0.282704218 | 0.385477946 |
| GCGR | 0.010298123 | 0.283234356 | 0.386096373 |
| SMC3 | 0.006642487 | 0.283457964 | 0.386122857 |
| DNAJC19 | -0.013623112 | 0.283496503 | 0.386122857 |
| MRPS15 | -0.007352677 | 0.283505052 | 0.386122857 |
| CD46 | 0.012992274 | 0.28356017 | 0.386122857 |
| PCGF2 | -0.005423173 | 0.283940521 | 0.386536366 |
| NEPRO | -0.004918484 | 0.284126411 | 0.386684999 |
| PPP2R5C | 0.006213974 | 0.284335766 | 0.386865478 |
| STAT5B | 0.004771028 | 0.285108211 | 0.387811789 |
| RNF114 | -0.004652469 | 0.286153121 | 0.389128105 |
| SURF1 | -0.008962103 | 0.286310374 | 0.389236946 |
| NSA2 | 0.009523181 | 0.286805272 | 0.38970108 |

| | | | |
|---|---|---|---|
| UBQLN1 | 0.005969228 | 0.286806389 | 0.38970108 |
| SUCO | 0.005042186 | 0.287039674 | 0.38991296 |
| MRPL42 | -0.008020646 | 0.287319476 | 0.390187898 |
| RAD50 | -0.007162351 | 0.28759906 | 0.390462391 |
| PRKDC | -0.007696331 | 0.288032379 | 0.390945402 |
| TMEM35B | 0.005321204 | 0.28824301 | 0.391125979 |
| STT3B | 0.007741548 | 0.288769288 | 0.391734655 |
| GLT8D1 | 0.005059746 | 0.288888121 | 0.391790427 |
| MRPL46 | 0.004808555 | 0.289322932 | 0.392188226 |
| ECE1 | 0.005063827 | 0.289337039 | 0.392188226 |
| GTPBP6 | -0.00520494 | 0.289985416 | 0.39296142 |
| TRAPPC5 | 0.011225637 | 0.290234056 | 0.393192657 |
| NQO2 | -0.006159547 | 0.290391282 | 0.39329844 |
| MCFD2 | -0.006595159 | 0.29046818 | 0.39329844 |
| DDAH2 | -0.007211787 | 0.291504403 | 0.394426747 |
| AKTIP | -0.005537517 | 0.291515088 | 0.394426747 |
| SEC22B | 0.007408739 | 0.291536215 | 0.394426747 |
| RDX | 0.008185296 | 0.292062908 | 0.395033303 |
| CGNL1 | 0.013938811 | 0.292382526 | 0.395359527 |
| NSL1 | -0.005222683 | 0.292712367 | 0.395699395 |
| ZMIZ1 | -0.006064152 | 0.294102827 | 0.39747248 |
| NUCB2 | 0.004697597 | 0.295169009 | 0.398806479 |
| GSTM4 | -0.00562197 | 0.296061405 | 0.399905021 |
| ZMYM6 | 0.004984913 | 0.296188755 | 0.399969867 |
| PTGR1 | 0.007076902 | 0.297106542 | 0.401047179 |
| HNRNPH3 | -0.008341663 | 0.297145648 | 0.401047179 |
| NDUFS2 | -0.009952169 | 0.297611846 | 0.401568875 |
| ACYP1 | 0.004977292 | 0.297984337 | 0.401963886 |
| RNASEH2C | -0.007435171 | 0.298218733 | 0.402172454 |
| C2orf74 | -0.006221952 | 0.29848184 | 0.402419618 |
| RANBP1 | -0.007674722 | 0.30014604 | 0.404555131 |
| ABI2 | 0.006725437 | 0.300471696 | 0.404885811 |
| ARPC1A | -0.006576854 | 0.30074931 | 0.405151595 |
| SLC43A2 | -0.008516076 | 0.301091313 | 0.405503956 |
| GIPC1 | -0.005154355 | 0.301444878 | 0.405871695 |
| GORASP2 | -0.004762815 | 0.301714458 | 0.40612619 |
| BBX | 0.008720036 | 0.302252871 | 0.406742318 |
| EBPL | 0.005012109 | 0.302551629 | 0.407035698 |
| D2HGDH | 0.005067477 | 0.302788441 | 0.407245606 |
| TFAP2B | -0.008782296 | 0.303029956 | 0.407461725 |
| TARDBP | -0.006329305 | 0.303342205 | 0.407735747 |
| RPL31 | 0.011643908 | 0.303408739 | 0.407735747 |
| IFNGR1 | 0.00465553 | 0.303526345 | 0.407735747 |
| PPP1R16A | 0.007073728 | 0.303557282 | 0.407735747 |
| C9orf78 | -0.00556824 | 0.30435285 | 0.40869545 |
| SMIM27 | 0.004807941 | 0.304466616 | 0.408739337 |
| BCKDK | -0.005090764 | 0.304606768 | 0.408818615 |

| | | | |
|---|---|---|---|
| ROGDI | -0.004801468 | 0.305307412 | 0.409599819 |
| ACTR3 | -0.006570802 | 0.305351343 | 0.409599819 |
| ITPKB | 0.004493667 | 0.305748966 | 0.410024076 |
| NELFE | -0.005016169 | 0.305830296 | 0.410024076 |
| NPRL3 | 0.004609436 | 0.306318573 | 0.410569509 |
| WDR83OS | 0.012039034 | 0.307180767 | 0.411615696 |
| GMPR2 | -0.005019264 | 0.307299705 | 0.411665642 |
| CABP1 | -0.008515886 | 0.30786584 | 0.412314479 |
| NHP2 | -0.008168924 | 0.308525994 | 0.412996717 |
| TM9SF4 | -0.005125408 | 0.308539107 | 0.412996717 |
| FBXL5 | 0.006132586 | 0.308803517 | 0.413240916 |
| DDX42 | -0.005688086 | 0.309332394 | 0.4138388 |
| RPL24 | 0.02292986 | 0.309719755 | 0.414247091 |
| TMEM256 | -0.008029037 | 0.309875497 | 0.414345459 |
| RNF213 | -0.005568145 | 0.310126703 | 0.414499516 |
| SLC30A9 | 0.00653162 | 0.310155163 | 0.414499516 |
| DUT | -0.007766867 | 0.310256588 | 0.414509367 |
| AP2S1 | -0.008636345 | 0.310333422 | 0.414509367 |
| TRIM28 | -0.006813016 | 0.310409217 | 0.414509367 |
| AP3D1 | -0.005864195 | 0.31063122 | 0.41469597 |
| SECISBP2 | 0.006179012 | 0.311680741 | 0.415986925 |
| UPK3BL1 | 0.006103182 | 0.312168433 | 0.416527546 |
| NT5C | -0.007641437 | 0.312841192 | 0.417314752 |
| NDUFA11 | 0.014094542 | 0.314719376 | 0.419709093 |
| DDAH1 | -0.007932084 | 0.315023434 | 0.420003473 |
| CLUH | -0.006660509 | 0.315881147 | 0.420952081 |
| ZFP91 | -0.005285174 | 0.315917829 | 0.420952081 |
| ARV1 | 0.005183532 | 0.315985454 | 0.420952081 |
| EPS8L2 | 0.007835839 | 0.316194094 | 0.421118739 |
| PPP2CA | -0.005574978 | 0.31631285 | 0.42116563 |
| PPIL4 | 0.004649775 | 0.317004198 | 0.421974693 |
| MRPS11 | -0.005836441 | 0.317909405 | 0.423025531 |
| PSMD6 | -0.004953142 | 0.317961463 | 0.423025531 |
| PAPSS1 | 0.004685318 | 0.320070786 | 0.425719481 |
| BCAP31 | -0.008902328 | 0.320922144 | 0.426679591 |
| MGAT1 | -0.004888889 | 0.320961914 | 0.426679591 |
| DCTPP1 | -0.005231776 | 0.32112887 | 0.426788989 |
| AC100810.1 | -0.006096466 | 0.321463197 | 0.427120711 |
| APRT | -0.009532663 | 0.322015358 | 0.427701051 |
| TMED2 | -0.008323679 | 0.322069667 | 0.427701051 |
| MRPL27 | -0.007878752 | 0.322883024 | 0.428594277 |
| RAF1 | -0.005489342 | 0.322912332 | 0.428594277 |
| PSMA3 | -0.006571161 | 0.323093652 | 0.428722059 |
| TIAL1 | -0.006531481 | 0.323605983 | 0.429288884 |
| NNT-AS1 | -0.006190004 | 0.323997845 | 0.429626755 |
| VPS4B | 0.005152933 | 0.324031129 | 0.429626755 |
| NDUFS8 | -0.01273639 | 0.32413309 | 0.429648937 |

| | | | |
|---|---|---|---|
| MAP4 | 0.006622357 | 0.324641634 | 0.430209904 |
| ZNF511 | -0.004582997 | 0.324788869 | 0.430264571 |
| RHOC | -0.004671145 | 0.324932942 | 0.430264571 |
| HDAC2 | -0.005579811 | 0.324938945 | 0.430264571 |
| MVB12A | -0.004752371 | 0.325151402 | 0.430432831 |
| SCYL1 | -0.004473196 | 0.325245593 | 0.430444482 |
| METTL7A | -0.011068244 | 0.325557453 | 0.430744126 |
| SMO | -0.004762531 | 0.326303347 | 0.43161773 |
| SAMD4B | -0.005021666 | 0.326854098 | 0.432232819 |
| HYOU1 | -0.005271074 | 0.327264163 | 0.43266159 |
| ABHD14B | -0.005992688 | 0.327405218 | 0.432734585 |
| RPL6 | -0.019636812 | 0.327629405 | 0.432917387 |
| RAB5B | -0.005024991 | 0.32784327 | 0.433086458 |
| SSNA1 | -0.006349066 | 0.328316341 | 0.433597766 |
| MBTPS1 | -0.007197385 | 0.328495179 | 0.433720324 |
| AASDHPPT | 0.004624333 | 0.330631535 | 0.436326182 |
| SAR1B | 0.005798195 | 0.330641939 | 0.436326182 |
| FAM3B | 0.004534828 | 0.331178672 | 0.436920096 |
| MARK2 | 0.004262986 | 0.332389883 | 0.438403297 |
| ADCY5 | 0.005251569 | 0.332545615 | 0.438493969 |
| HMGCS1 | 0.004643505 | 0.333285979 | 0.439355288 |
| PIGH | 0.004504471 | 0.334460771 | 0.440755401 |
| GPAA1 | -0.007708521 | 0.334522944 | 0.440755401 |
| SUPT4H1 | -0.005467129 | 0.334758639 | 0.440950692 |
| HMG20B | 0.005529236 | 0.335127778 | 0.441321612 |
| RPL22 | -0.017311441 | 0.33547311 | 0.441660995 |
| GMPR | -0.005895376 | 0.335692455 | 0.441834377 |
| CPSF2 | 0.004321229 | 0.336057681 | 0.44214749 |
| IFT22 | -0.004879786 | 0.336105769 | 0.44214749 |
| MRPL38 | -0.006277121 | 0.336799107 | 0.442943986 |
| CD164 | -0.008425052 | 0.337167399 | 0.443312691 |
| PMPCA | -0.004576108 | 0.338144577 | 0.444481568 |
| SIVA1 | -0.007431966 | 0.338361374 | 0.444584564 |
| RPN2 | -0.008087374 | 0.338422393 | 0.444584564 |
| ATP6AP1 | 0.008873714 | 0.338487514 | 0.444584564 |
| YBX3 | -0.005752633 | 0.339497403 | 0.445794844 |
| HSPA5 | -0.01368713 | 0.339611861 | 0.445829008 |
| ING5 | 0.004152932 | 0.341069155 | 0.447625517 |
| RPL26L1 | -0.005908995 | 0.341251516 | 0.447748281 |
| IFI27L2 | 0.005888969 | 0.341481692 | 0.4479337 |
| XPA | 0.007084189 | 0.342343402 | 0.448947214 |
| RPA3 | -0.006976477 | 0.342629424 | 0.449205442 |
| ATP5MC2 | 0.017072078 | 0.342896146 | 0.44943824 |
| SPAG7 | -0.006956119 | 0.343201184 | 0.449709481 |
| RAB6A | -0.005295797 | 0.343281508 | 0.449709481 |
| DLAT | -0.004980406 | 0.343603993 | 0.450014999 |
| PPP1R7 | -0.004918159 | 0.343820283 | 0.450181311 |

| | | | |
|---|---|---|---|
| MYO5C | 0.004604035 | 0.345094459 | 0.451632051 |
| SDF2 | -0.005309339 | 0.345107451 | 0.451632051 |
| LATS1 | 0.004494839 | 0.345362291 | 0.451775314 |
| DHRS4L2 | 0.004761451 | 0.345462598 | 0.451775314 |
| BTG1 | 0.009807828 | 0.345533981 | 0.451775314 |
| ASB8 | -0.004410137 | 0.345575404 | 0.451775314 |
| MAP9 | -0.005494372 | 0.345775916 | 0.451920247 |
| RDH14 | -0.004595102 | 0.346346072 | 0.452548094 |
| ATP5F1E | -0.02306842 | 0.34644648 | 0.452561986 |
| PDIA3 | -0.011124652 | 0.346744979 | 0.45283457 |
| SFSWAP | 0.004594078 | 0.347482662 | 0.45368042 |
| ASRGL1 | 0.006795774 | 0.347792086 | 0.453966833 |
| MAP2K6 | -0.004890474 | 0.348962268 | 0.455376338 |
| KLHL9 | 0.004661711 | 0.34964358 | 0.456064792 |
| SNW1 | 0.004410332 | 0.349670784 | 0.456064792 |
| PTOV1 | 0.005896315 | 0.350034075 | 0.45642053 |
| ADD3 | 0.008575588 | 0.350702688 | 0.457058309 |
| HNRNPL | 0.005001526 | 0.350704531 | 0.457058309 |
| NUDC | 0.007136083 | 0.350849316 | 0.45712882 |
| NCBP2AS2 | -0.005723069 | 0.352083964 | 0.458618931 |
| DDX24 | -0.008216341 | 0.352190493 | 0.458639182 |
| PAFAH1B1 | -0.006661724 | 0.352796977 | 0.459271508 |
| TXNDC11 | 0.005051521 | 0.352866612 | 0.459271508 |
| MPST | 0.005139713 | 0.352951877 | 0.459271508 |
| CMC1 | -0.007708661 | 0.353040487 | 0.459271508 |
| EBNA1BP2 | -0.004293855 | 0.35362097 | 0.459907975 |
| SNHG14 | 0.011862872 | 0.35392636 | 0.460186428 |
| HOXD10 | -0.004987858 | 0.355201664 | 0.461725526 |
| ACSL3 | 0.008127758 | 0.355936696 | 0.462561713 |
| ME2 | -0.005048991 | 0.356185944 | 0.462764773 |
| RAB1A | -0.008161695 | 0.35636512 | 0.462764773 |
| DGCR2 | -0.005251222 | 0.356368349 | 0.462764773 |
| ENY2 | -0.008433784 | 0.35711915 | 0.463620303 |
| PSAP | -0.016230057 | 0.357413317 | 0.46387297 |
| NSMCE1 | -0.004757282 | 0.357497816 | 0.46387297 |
| UXS1 | -0.009553961 | 0.357816402 | 0.464166877 |
| NDUFB11 | 0.013131165 | 0.358318619 | 0.464675874 |
| RAB4A | 0.004775962 | 0.35842493 | 0.464675874 |
| CSNK1A1 | 0.008143169 | 0.358487361 | 0.464675874 |
| TCIM | -0.008015791 | 0.358577495 | 0.464675874 |
| TTC19 | -0.006401156 | 0.358700903 | 0.464716332 |
| CRYL1 | -0.008825475 | 0.358963081 | 0.464893104 |
| UQCC1 | 0.004093007 | 0.359021792 | 0.464893104 |
| BRD3 | 0.004200138 | 0.359186027 | 0.464986328 |
| TBCA | -0.011433245 | 0.359679876 | 0.465506099 |
| FXYD2 | -0.052358788 | 0.35979457 | 0.465535017 |
| CTIF | 0.004094768 | 0.359987038 | 0.465664526 |

| | | | |
|---|---|---|---|
| UFC1 | -0.007332372 | 0.360123926 | 0.465722091 |
| LONRF2 | 0.004354055 | 0.360242031 | 0.465748409 |
| MRPL14 | -0.005508842 | 0.360369449 | 0.465748409 |
| RPS12 | 0.026024445 | 0.360421453 | 0.465748409 |
| LINC00958 | 0.004215788 | 0.36127682 | 0.466734097 |
| MAPK1IP1L | -0.005854148 | 0.361687355 | 0.467025195 |
| FAM27C | -0.004196483 | 0.361687435 | 0.467025195 |
| GMFB | -0.004009368 | 0.362438026 | 0.467874542 |
| TMEM245 | 0.007475529 | 0.362839866 | 0.468273365 |
| TFRC | 0.005135897 | 0.363574655 | 0.46910157 |
| PDPK1 | -0.004529574 | 0.363953639 | 0.469470392 |
| DAZAP1 | -0.004785006 | 0.364113579 | 0.469556549 |
| CYB5D2 | -0.004603654 | 0.3648354 | 0.470367071 |
| CRTC1 | 0.004101244 | 0.365722366 | 0.471390041 |
| GSDMD | 0.004426693 | 0.366022751 | 0.471656618 |
| THEM4 | 0.004406426 | 0.366251483 | 0.471830751 |
| SUPT16H | -0.005039097 | 0.366916953 | 0.472567287 |
| TRAPPC2L | 0.006357329 | 0.367022341 | 0.472582279 |
| UGGT1 | 0.003988638 | 0.368181958 | 0.473954354 |
| DPM1 | 0.00437048 | 0.368362213 | 0.474065335 |
| PRMT2 | 0.005089817 | 0.368923377 | 0.474666346 |
| ARHGEF10L | 0.004396055 | 0.369239441 | 0.474951778 |
| CLN5 | 0.006580959 | 0.369557475 | 0.47515723 |
| CSAD | -0.004449261 | 0.36958768 | 0.47515723 |
| C17orf75 | 0.004370537 | 0.370234618 | 0.475867596 |
| AZI2 | 0.005047542 | 0.370651664 | 0.476282192 |
| APEH | -0.005055524 | 0.370893134 | 0.476471022 |
| TOR1AIP1 | 0.004047677 | 0.371339099 | 0.476922395 |
| HSD17B4 | 0.006769164 | 0.371659212 | 0.477211944 |
| PNPLA8 | -0.005579057 | 0.372763249 | 0.478507649 |
| SNX1 | 0.004383271 | 0.373642389 | 0.479514074 |
| UHMK1 | -0.006038108 | 0.374144887 | 0.480036746 |
| KLC1 | -0.00398953 | 0.374660199 | 0.480575589 |
| ZNF737 | 0.004087663 | 0.375165778 | 0.481101676 |
| PEX2 | -0.005406644 | 0.375329643 | 0.481189402 |
| SCRN1 | -0.004537317 | 0.375637386 | 0.481433127 |
| DHX36 | 0.00568136 | 0.375710756 | 0.481433127 |
| FAM177A1 | 0.004742867 | 0.376327503 | 0.482100875 |
| THY1 | 0.01188524 | 0.376636249 | 0.482260606 |
| AKAP8L | -0.005077423 | 0.376841746 | 0.482260606 |
| ESCO1 | 0.00413952 | 0.376852896 | 0.482260606 |
| ETFA | 0.006450671 | 0.376909817 | 0.482260606 |
| ICA1 | -0.003951396 | 0.376930527 | 0.482260606 |
| FLYWCH2 | -0.004476302 | 0.377156465 | 0.482427237 |
| CYP51A1 | -0.005954981 | 0.37752132 | 0.482771429 |
| BCLAF1 | 0.006900414 | 0.377949114 | 0.483195912 |
| CYB5B | 0.004673801 | 0.378322588 | 0.483540324 |

| | | | |
|---|---|---|---|
| HADHA | -0.00791797 | 0.378410351 | 0.483540324 |
| CTBP2 | 0.006379946 | 0.378953583 | 0.484111762 |
| MAOA | -0.005274431 | 0.379353249 | 0.484499551 |
| MRPS24 | -0.011650314 | 0.379567872 | 0.484650872 |
| SNAPIN | -0.003992467 | 0.379778848 | 0.484682714 |
| GNG7 | 0.006191054 | 0.379785106 | 0.484682714 |
| CCAR1 | 0.005689516 | 0.380514608 | 0.485490797 |
| CACNA1H | 0.00433405 | 0.380732547 | 0.485645943 |
| UBXN2A | 0.003908098 | 0.381061578 | 0.485864699 |
| GET1 | -0.00404061 | 0.38109681 | 0.485864699 |
| ACSL1 | -0.010753916 | 0.38136722 | 0.485981338 |
| DNAJC7 | 0.005633569 | 0.38138111 | 0.485981338 |
| MAP1LC3B | -0.006658362 | 0.382207972 | 0.4869119 |
| TP53BP1 | -0.004453694 | 0.382460056 | 0.48710994 |
| RPL41 | -0.03074358 | 0.382561905 | 0.487116585 |
| ZSCAN18 | -0.004453683 | 0.382859602 | 0.487287562 |
| TMEM50A | 0.006400627 | 0.382977375 | 0.487287562 |
| COPS5 | 0.004830773 | 0.382986178 | 0.487287562 |
| ARL6IP1 | -0.00935887 | 0.383223527 | 0.487466515 |
| ESYT2 | 0.004055253 | 0.383753649 | 0.487905953 |
| EML4 | 0.003933307 | 0.383762567 | 0.487905953 |
| UBAP2L | -0.004218147 | 0.384689251 | 0.488960796 |
| LINC00667 | -0.00448747 | 0.384907364 | 0.489114702 |
| EMC6 | -0.00592953 | 0.385589846 | 0.489858471 |
| OBSCN | -0.003854019 | 0.385865725 | 0.490085442 |
| DNAJC3 | -0.006531356 | 0.386025691 | 0.490165115 |
| CLPP | 0.005060754 | 0.386639931 | 0.490821429 |
| GPR108 | 0.004950136 | 0.387157898 | 0.491355228 |
| PSMA6 | -0.005921992 | 0.388012101 | 0.492315379 |
| MDH1 | 0.009636668 | 0.388117636 | 0.492325365 |
| MMAB | -0.005447152 | 0.388444805 | 0.492616418 |
| PRRC2C | -0.007394362 | 0.388596921 | 0.492685383 |
| DIS3 | -0.004024235 | 0.389259702 | 0.493401599 |
| NNT | -0.00917812 | 0.38945972 | 0.493531032 |
| KCMF1 | 0.003999209 | 0.390127195 | 0.494234271 |
| DLG3 | 0.004724084 | 0.390210752 | 0.494234271 |
| CCDC6 | 0.004622497 | 0.390911796 | 0.494997831 |
| KTN1 | -0.011126761 | 0.391030837 | 0.495024221 |
| MAP3K13 | -0.007134307 | 0.391197246 | 0.495110549 |
| GLUD1 | -0.0072008 | 0.392033703 | 0.496044653 |
| SLC39A3 | -0.003972055 | 0.39229475 | 0.496230242 |
| FKBP3 | -0.005892792 | 0.392377255 | 0.496230242 |
| NDUFV2 | -0.010736454 | 0.39295659 | 0.496838267 |
| EIF5B | 0.006320483 | 0.393963657 | 0.497986659 |
| DEAF1 | -0.003949722 | 0.39465289 | 0.49873282 |
| TFAM | -0.005173121 | 0.394759054 | 0.498741953 |
| CCNC | 0.004736022 | 0.39515664 | 0.499119174 |

| | | | |
|---|---|---|---|
| ADCY6 | 0.004168869 | 0.395591594 | 0.4994299 |
| SETD3 | 0.005258026 | 0.395600792 | 0.4994299 |
| POLD4 | 0.003872961 | 0.396207094 | 0.500070095 |
| PHYH | -0.005356828 | 0.396737495 | 0.500614196 |
| ZDHHC6 | 0.003783766 | 0.397051796 | 0.500885411 |
| GLG1 | 0.008128776 | 0.398039953 | 0.501904838 |
| TMEM170A | -0.004369943 | 0.398059024 | 0.501904838 |
| ZNF428 | 0.005684296 | 0.400280823 | 0.504580052 |
| ERCC1 | 0.005323902 | 0.400784846 | 0.505089102 |
| GMPS | 0.003830261 | 0.401407189 | 0.505746973 |
| TRIB2 | 0.004365737 | 0.401615166 | 0.505882572 |
| ZKSCAN1 | -0.005200717 | 0.40241546 | 0.506764011 |
| PEPD | -0.004394806 | 0.402853983 | 0.507189543 |
| PRKAB1 | -0.005111426 | 0.403363254 | 0.507703911 |
| ADD1 | -0.004848001 | 0.403661809 | 0.507952865 |
| YWHAZ | -0.007468271 | 0.40377687 | 0.507970851 |
| LSM1 | -0.004448894 | 0.404092369 | 0.508240926 |
| STX10 | 0.003813953 | 0.406698509 | 0.511391165 |
| SPOP | -0.004353977 | 0.406909474 | 0.511528842 |
| TSTD1 | -0.010719838 | 0.40886113 | 0.513854141 |
| MED29 | -0.003789984 | 0.409186078 | 0.514134352 |
| HUWE1 | 0.006249779 | 0.409820275 | 0.514802893 |
| MDP1 | -0.004223454 | 0.410750716 | 0.515843138 |
| NIPA2 | -0.0037804 | 0.410865755 | 0.515859096 |
| UBXN4 | -0.007772797 | 0.411843197 | 0.516957559 |
| VMP1 | 0.004591471 | 0.411990392 | 0.517013584 |
| DLST | -0.00458607 | 0.413247265 | 0.518461788 |
| NAP1L1 | -0.00799605 | 0.41431916 | 0.519608155 |
| PSMG4 | -0.004249259 | 0.414367146 | 0.519608155 |
| UNC50 | -0.005574668 | 0.41502164 | 0.52029945 |
| RNF5 | -0.005871413 | 0.416648941 | 0.522175764 |
| TMEM165 | -0.005139467 | 0.416725471 | 0.522175764 |
| PSMB4 | 0.00615933 | 0.416944725 | 0.522320665 |
| IPO7 | -0.005577542 | 0.417622811 | 0.523040146 |
| PDE4DIP | -0.006329182 | 0.418421632 | 0.523830328 |
| OTUD1 | 0.004130432 | 0.418543049 | 0.523830328 |
| CAB39 | 0.005070329 | 0.418565476 | 0.523830328 |
| UCHL1 | -0.00963297 | 0.419746059 | 0.525177434 |
| AC124017.1 | 0.008303159 | 0.421128597 | 0.526663197 |
| RPL7A | 0.018408809 | 0.421218385 | 0.526663197 |
| USP16 | -0.0048448 | 0.421246977 | 0.526663197 |
| LARGE2 | 0.004047581 | 0.421476241 | 0.526819174 |
| LAMTOR2 | -0.006374369 | 0.423152652 | 0.528738019 |
| ZNF330 | 0.003720947 | 0.423221168 | 0.528738019 |
| ARF4 | -0.005938492 | 0.424462922 | 0.530146106 |
| TSEN34 | -0.003949577 | 0.424558586 | 0.530146106 |
| CASC4 | -0.005025769 | 0.424761047 | 0.530267568 |

| | | | |
|---|---|---|---|
| TMEM147 | 0.00667156 | 0.425078898 | 0.530468604 |
| ECHDC3 | 0.004620371 | 0.425132545 | 0.530468604 |
| SUMF2 | 0.005912089 | 0.425780375 | 0.531082615 |
| PIP5K1A | 0.003727995 | 0.425835336 | 0.531082615 |
| TRIP6 | -0.004457933 | 0.427009041 | 0.532414687 |
| ARL6IP4 | -0.008560803 | 0.427197907 | 0.532518459 |
| IFT57 | -0.004342338 | 0.427571841 | 0.532852819 |
| HADHB | -0.009412285 | 0.42772059 | 0.532906449 |
| RNF216 | 0.003529296 | 0.427978034 | 0.533095446 |
| HOXA7 | -0.004859159 | 0.428170677 | 0.533203651 |
| ICE1 | -0.004629553 | 0.42896498 | 0.53406087 |
| RIOX2 | 0.003593776 | 0.429310182 | 0.534266474 |
| PRKCQ-AS1 | -0.004706217 | 0.429379221 | 0.534266474 |
| TOM1 | -0.003678854 | 0.429448076 | 0.534266474 |
| DAB2 | -0.003579512 | 0.429657026 | 0.534368344 |
| TARS | -0.003713818 | 0.429741969 | 0.534368344 |
| SELENOS | -0.00584925 | 0.430080246 | 0.534657095 |
| MRPS28 | 0.005850318 | 0.430317287 | 0.534741102 |
| NBN | -0.003597463 | 0.430359978 | 0.534741102 |
| SGPL1 | 0.003560569 | 0.430833084 | 0.535197037 |
| CLDN4 | 0.005570197 | 0.432076455 | 0.536609365 |
| DALRD3 | 0.003479194 | 0.433281206 | 0.537933852 |
| SNRPC | 0.004506857 | 0.433356353 | 0.537933852 |
| VPS51 | -0.00500497 | 0.433576342 | 0.538053818 |
| SAT2 | 0.008429239 | 0.433724934 | 0.538053818 |
| CHMP2A | 0.007589405 | 0.433779991 | 0.538053818 |
| PCNA | 0.003988396 | 0.433879939 | 0.538053818 |
| H2AFV | -0.006774269 | 0.43456823 | 0.538774827 |
| PPP1R35 | 0.003669965 | 0.435403394 | 0.539677528 |
| CNN3 | -0.006969743 | 0.435556823 | 0.539734991 |
| ATN1 | -0.005082056 | 0.436118636 | 0.54021022 |
| GGNBP2 | -0.004419236 | 0.436154651 | 0.54021022 |
| RPL23 | -0.011993187 | 0.436903004 | 0.541001852 |
| MCEE | 0.004185913 | 0.437008439 | 0.541001852 |
| IDH3G | 0.005197207 | 0.437387945 | 0.541338726 |
| UCHL5 | -0.003815611 | 0.437872399 | 0.541805293 |
| BAD | -0.005286622 | 0.438126361 | 0.5419865 |
| NDUFA10 | 0.007694823 | 0.438397384 | 0.542056142 |
| ABCF1 | -0.003735885 | 0.438397717 | 0.542056142 |
| CCDC106 | -0.003928803 | 0.438679785 | 0.542206312 |
| QSER1 | 0.003798737 | 0.438734288 | 0.542206312 |
| NAA50 | 0.004746312 | 0.440669364 | 0.544367863 |
| OXSR1 | 0.004140429 | 0.440699315 | 0.544367863 |
| ALG13 | 0.003743425 | 0.441548923 | 0.545283714 |
| DDX1 | 0.005682033 | 0.44222487 | 0.54598471 |
| MAOB | -0.004228296 | 0.442674413 | 0.546312335 |
| CAPZA2 | -0.006900078 | 0.442706981 | 0.546312335 |

| | | | |
|---|---|---|---|
| TMEM263 | 0.005251574 | 0.442953869 | 0.546483225 |
| AFF4 | 0.004730916 | 0.443214533 | 0.546671021 |
| DAP3 | -0.003833438 | 0.443351795 | 0.546698545 |
| ATP1B3 | -0.006354979 | 0.443453749 | 0.546698545 |
| MYOF | 0.003730192 | 0.444932858 | 0.548387906 |
| PNPT1 | -0.00341637 | 0.445095341 | 0.548454073 |
| NDFIP1 | -0.008065908 | 0.445306822 | 0.54858057 |
| ATG4B | -0.004170507 | 0.447372666 | 0.550990865 |
| PSMB5 | 0.005840334 | 0.447723256 | 0.551287966 |
| AP1S1 | -0.005698987 | 0.44814501 | 0.551672527 |
| HMGCL | -0.004136408 | 0.448327608 | 0.551762566 |
| ZNF528-AS1 | 0.003511869 | 0.449248928 | 0.552761496 |
| POP4 | -0.003430536 | 0.450028875 | 0.553586031 |
| EGLN1 | -0.004038597 | 0.450425568 | 0.553938836 |
| TSNAX | -0.004389421 | 0.450603185 | 0.554022111 |
| FAM174C | -0.004012706 | 0.451204253 | 0.554625857 |
| IGF2R | 0.006286135 | 0.453554844 | 0.557379319 |
| SDHAF2 | -0.003641436 | 0.454027529 | 0.557824219 |
| SLC3A2 | -0.00510366 | 0.45485712 | 0.558707295 |
| RBM25 | 0.006155459 | 0.455429049 | 0.559273529 |
| MARCKS | -0.004647111 | 0.455742476 | 0.559522119 |
| DGLUCY | 0.004312669 | 0.456020337 | 0.559726934 |
| SFT2D2 | -0.003570143 | 0.456242567 | 0.559863383 |
| RBM10 | -0.003324107 | 0.456626166 | 0.560185588 |
| ZFAND1 | 0.003355338 | 0.456727389 | 0.560185588 |
| CD320 | -0.004185399 | 0.456875381 | 0.560230795 |
| BNIP3 | -0.005835465 | 0.457196969 | 0.560488794 |
| PYROXD1 | 0.003514152 | 0.457875949 | 0.561184697 |
| MFSD4A | -0.0114046 | 0.458981858 | 0.562403389 |
| OXCT1 | 0.00682487 | 0.459411426 | 0.562792953 |
| NDUFA9 | -0.005724488 | 0.459968681 | 0.563338708 |
| G0S2 | -0.008535486 | 0.461344521 | 0.564886502 |
| PACS2 | -0.003320321 | 0.461580729 | 0.565038478 |
| TMEM205 | 0.007853267 | 0.461779891 | 0.565145043 |
| COMMD7 | -0.003956582 | 0.46197218 | 0.565243145 |
| SKA2 | -0.003465457 | 0.462762164 | 0.56607233 |
| AGGF1 | -0.003487465 | 0.462925845 | 0.566135173 |
| PSMD4 | 0.004355305 | 0.463325532 | 0.566486541 |
| BSCL2 | -0.005603666 | 0.463825904 | 0.566960811 |
| PKIG | -0.003802897 | 0.4647109 | 0.567806531 |
| FLOT1 | 0.004308144 | 0.464839492 | 0.567806531 |
| IQGAP1 | -0.005052746 | 0.464855694 | 0.567806531 |
| KPNB1 | -0.005132413 | 0.465911872 | 0.568958757 |
| BCKDHA | -0.00482855 | 0.466779819 | 0.569880617 |
| C19orf25 | -0.003720875 | 0.467045958 | 0.569940636 |
| SCD5 | -0.008111869 | 0.467157689 | 0.569940636 |
| RNF181 | -0.00501844 | 0.467247481 | 0.569940636 |

| | | | |
|---|---|---|---|
| MALSU1 | -0.003918221 | 0.467281224 | 0.569940636 |
| TMEM33 | -0.004582103 | 0.467504147 | 0.570074602 |
| TRAP1 | -0.003863908 | 0.467903316 | 0.570423366 |
| ENOSF1 | 0.005357393 | 0.46805797 | 0.570473943 |
| LYRM9 | -0.004202088 | 0.468364502 | 0.570709562 |
| SLC25A29 | -0.004515919 | 0.468755604 | 0.571048091 |
| EIF3F | 0.006343077 | 0.469099599 | 0.571329085 |
| ZCCHC14 | 0.003226417 | 0.469251514 | 0.571376059 |
| SUPT5H | -0.00403866 | 0.470867569 | 0.573205364 |
| CABIN1 | -0.003334887 | 0.471752995 | 0.573952607 |
| TIMM10 | -0.004886672 | 0.471810823 | 0.573952607 |
| PSMC5 | -0.005658196 | 0.471995125 | 0.573952607 |
| CALCOCO2 | -0.003550784 | 0.472049559 | 0.573952607 |
| TOMM20 | 0.008389583 | 0.472050686 | 0.573952607 |
| TCEAL9 | 0.005909887 | 0.472781775 | 0.574702901 |
| CKAP5 | 0.003161899 | 0.473786339 | 0.575666348 |
| GYPC | -0.005022993 | 0.473802753 | 0.575666348 |
| CTNNAL1 | -0.005722394 | 0.473918248 | 0.575667925 |
| SLC18B1 | -0.003664903 | 0.474119677 | 0.575773859 |
| COX16 | 0.005977074 | 0.476497394 | 0.578522005 |
| PRRG4 | -0.003236015 | 0.477357739 | 0.579314412 |
| PRDX1 | 0.010003623 | 0.477487944 | 0.579314412 |
| PPIG | -0.005321853 | 0.477494819 | 0.579314412 |
| TXNRD2 | 0.00320406 | 0.478458985 | 0.58022865 |
| NPTN | 0.00608621 | 0.478531307 | 0.58022865 |
| EIF2S2 | -0.004833017 | 0.478593678 | 0.58022865 |
| GNG12 | -0.003737716 | 0.478832511 | 0.580378622 |
| YIPF2 | -0.004379032 | 0.479269435 | 0.5806909 |
| KRT18 | 0.005128769 | 0.479482642 | 0.5806909 |
| RBM39 | 0.008357134 | 0.479533478 | 0.5806909 |
| CXCL14 | -0.01071516 | 0.479550926 | 0.5806909 |
| OSBP | -0.004717395 | 0.479760833 | 0.580805562 |
| MEAF6 | -0.004149319 | 0.480224848 | 0.581227722 |
| MRI1 | -0.003329091 | 0.480497336 | 0.581417924 |
| PEX19 | -0.003472809 | 0.481139125 | 0.582054794 |
| KLHDC7A | -0.00350552 | 0.481932703 | 0.582745731 |
| ADRM1 | -0.00384535 | 0.48194147 | 0.582745731 |
| RPL17 | 0.015102555 | 0.482531687 | 0.583319481 |
| RPS16 | -0.010836494 | 0.484300576 | 0.585317479 |
| GFM2 | 0.003228495 | 0.484786105 | 0.585763843 |
| MPPE1 | 0.004471489 | 0.485004713 | 0.585887553 |
| SMARCE1 | 0.004399718 | 0.485181887 | 0.585961163 |
| CCDC25 | 0.003646218 | 0.486300251 | 0.587171154 |
| SPTLC1 | 0.003891015 | 0.487091399 | 0.587985571 |
| TIMM21 | -0.003167672 | 0.487370348 | 0.588181452 |
| ELK4 | -0.00318081 | 0.487887459 | 0.588664595 |
| WDR60 | 0.003821034 | 0.488546308 | 0.589194917 |

| | | | |
|---|---|---|---|
| SERINC1 | -0.006493386 | 0.488560752 | 0.589194917 |
| PPIL2 | -0.003405843 | 0.488919443 | 0.589486465 |
| KLHL21 | -0.003826549 | 0.489076991 | 0.589535416 |
| PNISR | -0.009083808 | 0.489481632 | 0.58988212 |
| ATP13A3 | 0.003412571 | 0.489796099 | 0.590120013 |
| SLC9A3-AS1 | 0.003787984 | 0.490116497 | 0.590364937 |
| FBXW4 | 0.003650336 | 0.4906877 | 0.590911777 |
| DENND10 | -0.003649314 | 0.49144672 | 0.591684479 |
| CXADR | 0.003214433 | 0.491601312 | 0.591729277 |
| PSMC4 | 0.003613178 | 0.491894262 | 0.591839829 |
| KRT8 | 0.005044878 | 0.491927967 | 0.591839829 |
| CCNL1 | -0.00391692 | 0.492092074 | 0.591896003 |
| TSPAN31 | -0.00374846 | 0.492306596 | 0.592012775 |
| DHX9 | 0.004878658 | 0.492799622 | 0.59241558 |
| TMPO | 0.003329065 | 0.4928766 | 0.59241558 |
| VEZF1 | -0.004480329 | 0.493009432 | 0.59243398 |
| COX4I1 | -0.013522219 | 0.49482407 | 0.594472864 |
| VPS25 | -0.004400556 | 0.4956042 | 0.595268232 |
| FERMT2 | 0.003815027 | 0.495884956 | 0.595463569 |
| SCOC | 0.005392155 | 0.496006029 | 0.59546711 |
| EMX2OS | 0.004380072 | 0.496748404 | 0.596137865 |
| PSMA4 | 0.004769158 | 0.496801264 | 0.596137865 |
| H1FX | -0.005037149 | 0.498625337 | 0.598184275 |
| DAD1 | 0.008766216 | 0.499751198 | 0.599306631 |
| AHDC1 | -0.003649272 | 0.499798667 | 0.599306631 |
| PAM16 | 0.004481349 | 0.50262485 | 0.602552168 |
| FIBP | -0.004166736 | 0.503708775 | 0.60370802 |
| BUB3 | 0.003611415 | 0.504925121 | 0.604917512 |
| PFKP | -0.003712544 | 0.504957923 | 0.604917512 |
| SCAP | -0.003601087 | 0.505982477 | 0.606000871 |
| TIMMDC1 | -0.004201215 | 0.5065172 | 0.60642608 |
| C12orf10 | -0.003586555 | 0.506603002 | 0.60642608 |
| BST2 | 0.004583718 | 0.506698403 | 0.60642608 |
| AAMDC | 0.004867382 | 0.507128163 | 0.606759483 |
| EPOR | -0.003091738 | 0.507217707 | 0.606759483 |
| HES1 | -0.005969672 | 0.507733509 | 0.607232413 |
| PCMTD2 | 0.003549392 | 0.509742228 | 0.609490174 |
| SNX27 | 0.003859117 | 0.510261663 | 0.609966574 |
| FDX2 | -0.003014138 | 0.510659517 | 0.610297445 |
| FAM111A | 0.003457031 | 0.511235044 | 0.61084045 |
| SDSL | 0.003479237 | 0.511412343 | 0.610907494 |
| SHMT1 | 0.002969896 | 0.511589197 | 0.610973973 |
| RSRC2 | -0.005161903 | 0.511913364 | 0.611216311 |
| ECSIT | -0.003403811 | 0.512467652 | 0.61173323 |
| IRF3 | 0.002906915 | 0.515008268 | 0.614620426 |
| ATP6V1D | -0.00524808 | 0.515395681 | 0.614900538 |
| DCTN6 | -0.003368757 | 0.515585756 | 0.614900538 |

| | | | |
|---|---|---|---|
| FAM107B | 0.005318075 | 0.515654077 | 0.614900538 |
| TRAPPC3 | -0.003178872 | 0.515730901 | 0.614900538 |
| AK6 | -0.00400341 | 0.51650434 | 0.615677082 |
| PGLS | 0.004423032 | 0.516632294 | 0.615684017 |
| NPR3 | -0.004141212 | 0.517071906 | 0.616062273 |
| MRPL37 | -0.003158136 | 0.517759524 | 0.616735766 |
| HNRNPH1 | 0.009810093 | 0.518495985 | 0.617467106 |
| CPPED1 | 0.002806575 | 0.519874687 | 0.618962753 |
| U2AF1L4 | 0.002923694 | 0.520373567 | 0.619410425 |
| CENPX | 0.004118709 | 0.520764567 | 0.619668667 |
| DPM2 | -0.003125879 | 0.52083637 | 0.619668667 |
| CNN2 | -0.003130735 | 0.521417302 | 0.620151929 |
| BAZ2A | -0.003150645 | 0.521488599 | 0.620151929 |
| IMPDH2 | 0.003238779 | 0.521802006 | 0.620378281 |
| HOOK1 | 0.00392075 | 0.522081088 | 0.620563726 |
| XIAP | -0.003870304 | 0.522697198 | 0.621149594 |
| SPCS2 | -0.007321024 | 0.523751667 | 0.622255987 |
| GIHCG | -0.002989873 | 0.524057838 | 0.622473035 |
| MLLT6 | -0.004931145 | 0.525242131 | 0.623674566 |
| IFT43 | 0.002789525 | 0.525316843 | 0.623674566 |
| POLR2J3 | 0.003952431 | 0.526037008 | 0.624382519 |
| HSPA4L | 0.002877037 | 0.526461521 | 0.624739295 |
| LCN12 | -0.003717793 | 0.52786159 | 0.626253301 |
| PCCA-DT | -0.003100561 | 0.52838915 | 0.626679465 |
| COX6A1 | -0.012131061 | 0.528469432 | 0.626679465 |
| MRPL22 | 0.003992017 | 0.529237443 | 0.627442604 |
| TCEA2 | -0.003556843 | 0.529571535 | 0.627691067 |
| MCTS1 | 0.003521826 | 0.529725881 | 0.627726414 |
| IMP4 | -0.002891842 | 0.530756982 | 0.628800457 |
| COMMD4 | 0.003713233 | 0.531554023 | 0.629488592 |
| NUDT12 | -0.003275795 | 0.531587569 | 0.629488592 |
| GNAS | -0.012252153 | 0.532258565 | 0.63013514 |
| UBB | -0.011128803 | 0.532506939 | 0.630281164 |
| GSPT1 | -0.00451254 | 0.532864276 | 0.63055606 |
| BOLA1 | -0.002796911 | 0.534021404 | 0.631777023 |
| OAT | -0.003857656 | 0.534284609 | 0.631940102 |
| APLP2 | -0.010940541 | 0.534487525 | 0.632007604 |
| LAMA5 | -0.003172131 | 0.534592427 | 0.632007604 |
| RTCB | 0.002986138 | 0.534767294 | 0.632066103 |
| SARNP | 0.00361123 | 0.535119142 | 0.632333707 |
| TM7SF3 | -0.002878329 | 0.535905436 | 0.633114437 |
| RFXANK | -0.002937308 | 0.536210791 | 0.633326756 |
| HERPUD1 | 0.007328509 | 0.53651081 | 0.633532676 |
| ZNRD2 | 0.00327579 | 0.537710847 | 0.634801026 |
| POLR2A | -0.003655533 | 0.539509081 | 0.636774825 |
| NOP58 | 0.002971148 | 0.540055197 | 0.637270189 |
| DNM1L | 0.00425789 | 0.540248116 | 0.637348644 |

| | | | |
|---|---|---|---|
| TMEM179B | -0.004311563 | 0.540437055 | 0.637422366 |
| ETNK1 | 0.005460305 | 0.541302532 | 0.638293817 |
| DLD | -0.005126799 | 0.541984303 | 0.638923478 |
| BHMT2 | -0.004631508 | 0.542090005 | 0.638923478 |
| FAN1 | -0.003389447 | 0.542419563 | 0.639088532 |
| RHOT1 | 0.003106114 | 0.542577085 | 0.639088532 |
| KAT2A | 0.003711435 | 0.542610378 | 0.639088532 |
| PPP1CC | -0.003499398 | 0.54317123 | 0.639599666 |
| CHD8 | 0.002945145 | 0.543916837 | 0.640328066 |
| NT5C3B | -0.003264958 | 0.544060682 | 0.640347863 |
| RBM17 | 0.004167426 | 0.545381836 | 0.641752996 |
| PPP4R2 | 0.003331936 | 0.545826455 | 0.642126292 |
| KIF9 | -0.002962514 | 0.547028837 | 0.643390659 |
| FBXO44 | 0.003360166 | 0.547404288 | 0.643666371 |
| COQ4 | 0.004320926 | 0.547518627 | 0.643666371 |
| ARPC3 | -0.005754729 | 0.547791904 | 0.643834147 |
| RPL11 | 0.015665819 | 0.547916781 | 0.643834147 |
| MBD4 | 0.00337367 | 0.548667044 | 0.644565502 |
| BZW1 | -0.004378373 | 0.549119282 | 0.644946482 |
| ATP6AP2 | 0.005546735 | 0.549659319 | 0.645430382 |
| SELENOK | -0.004601738 | 0.550091531 | 0.645787473 |
| GDI2 | -0.004288335 | 0.550577761 | 0.64608023 |
| CLDN19 | -0.004989368 | 0.550597237 | 0.64608023 |
| CBX3 | -0.005135952 | 0.551004143 | 0.646372082 |
| PTS | -0.004176899 | 0.551102402 | 0.646372082 |
| TMEM167B | -0.002717481 | 0.551548813 | 0.646728763 |
| C6orf136 | 0.002815233 | 0.551663099 | 0.646728763 |
| PIGP | -0.003958572 | 0.55184705 | 0.646793997 |
| SREK1IP1 | -0.0029399 | 0.55230972 | 0.647185798 |
| PRADC1 | -0.002855753 | 0.553146561 | 0.648015759 |
| YY1 | -0.004103622 | 0.554099969 | 0.648981864 |
| LINC01159 | -0.005172311 | 0.554506012 | 0.649306575 |
| NEK9 | 0.002758628 | 0.554907331 | 0.649591766 |
| BTBD2 | -0.00287756 | 0.555007288 | 0.649591766 |
| MRPL44 | -0.002745737 | 0.555671491 | 0.650218195 |
| NECTIN2 | -0.002762187 | 0.555899946 | 0.650334562 |
| ATF6B | -0.002884523 | 0.556074288 | 0.650387584 |
| DEFB1 | 0.034521045 | 0.556209413 | 0.650394723 |
| CNPPD1 | -0.003879195 | 0.557304108 | 0.651419969 |
| DCAF8 | 0.003861704 | 0.557344639 | 0.651419969 |
| SMARCA1 | 0.003614424 | 0.558335806 | 0.652427166 |
| UBXN1 | 0.004364114 | 0.558995133 | 0.653014876 |
| FBXL15 | -0.003801341 | 0.559117834 | 0.653014876 |
| RPL13A | -0.009701707 | 0.55922738 | 0.653014876 |
| DSTYK | -0.002727551 | 0.559900054 | 0.653648952 |
| SSBP1 | -0.005120024 | 0.560264599 | 0.653923094 |
| ST3GAL4 | 0.005306014 | 0.560781038 | 0.654374355 |

| | | | |
|---|---|---|---|
| SHOC2 | 0.003318627 | 0.561078017 | 0.654569379 |
| NAMPT | 0.003822438 | 0.561292676 | 0.654668297 |
| UFM1 | -0.003862181 | 0.561470541 | 0.654724265 |
| TSG101 | 0.003660476 | 0.561742616 | 0.654831346 |
| UFD1 | 0.002672985 | 0.561822173 | 0.654831346 |
| CASZ1 | -0.003012139 | 0.562731029 | 0.655739048 |
| HNRNPD | -0.005243053 | 0.56352389 | 0.656511193 |
| TMEM243 | -0.003058066 | 0.564757334 | 0.657796146 |
| LARP6 | -0.0028327 | 0.56504926 | 0.657984135 |
| RANBP2 | 0.004262058 | 0.56536929 | 0.658140999 |
| TPD52L2 | -0.002813075 | 0.565445084 | 0.658140999 |
| MRPL28 | -0.004311754 | 0.565877533 | 0.658222007 |
| WDR34 | -0.00362923 | 0.565889061 | 0.658222007 |
| PNPLA4 | -0.003114733 | 0.565906403 | 0.658222007 |
| GAK | -0.002915824 | 0.566840801 | 0.659156742 |
| HSPA1A | -0.004710704 | 0.568006282 | 0.660359701 |
| RXRA | 0.002528128 | 0.569159674 | 0.661548055 |
| PDXK | -0.007012298 | 0.56968512 | 0.661979349 |
| BDH2 | 0.004974687 | 0.569793373 | 0.661979349 |
| RNF11 | -0.002943808 | 0.570509382 | 0.662658479 |
| AP1G1 | 0.003355525 | 0.571484744 | 0.663638469 |
| ACO1 | -0.002612798 | 0.571802218 | 0.66385421 |
| IFT27 | -0.002874761 | 0.572286043 | 0.664262939 |
| RPL5 | -0.011244208 | 0.573149983 | 0.665112584 |
| PGM1 | -0.003466599 | 0.573329561 | 0.665167852 |
| FBXO3 | -0.002846826 | 0.573922307 | 0.665702335 |
| MAP1LC3A | -0.002686015 | 0.574075956 | 0.665727374 |
| LIAS | 0.002538582 | 0.574839354 | 0.666459334 |
| LSR | 0.00254284 | 0.575357958 | 0.666862451 |
| RPS3A | -0.013313949 | 0.575451629 | 0.666862451 |
| ECPAS | 0.003087094 | 0.57589724 | 0.667225462 |
| CLMN | 0.004998067 | 0.576136168 | 0.667348902 |
| IDH3B | 0.003416456 | 0.576330348 | 0.667420465 |
| AGPAT2 | -0.005042222 | 0.576582849 | 0.667559518 |
| QARS | -0.003281711 | 0.5785756 | 0.669504845 |
| NDUFV1 | 0.006693474 | 0.578655902 | 0.669504845 |
| TM9SF1 | -0.003296585 | 0.578661498 | 0.669504845 |
| LZIC | -0.002772733 | 0.579171324 | 0.669940946 |
| DNAJB6 | -0.004278721 | 0.580088734 | 0.670848202 |
| MYDGF | -0.003769675 | 0.58061023 | 0.671297286 |
| ERGIC3 | -0.004191698 | 0.581706909 | 0.672280297 |
| MFSD1 | 0.002873908 | 0.581727168 | 0.672280297 |
| TUG1 | -0.003846891 | 0.581992567 | 0.672432851 |
| OGDH | -0.004434529 | 0.582427281 | 0.672492682 |
| TXNRD1 | 0.002575426 | 0.582524306 | 0.672492682 |
| ANKRD12 | 0.004345575 | 0.582555631 | 0.672492682 |
| RPL9 | -0.011116548 | 0.582577969 | 0.672492682 |

| | | | |
|---|---|---|---|
| TPCN1 | -0.002431959 | 0.583205051 | 0.673062422 |
| CCT7 | -0.003518564 | 0.583529934 | 0.673283222 |
| HIPK3 | 0.003377363 | 0.583822342 | 0.673466459 |
| CCT3 | -0.004357535 | 0.584071487 | 0.673599717 |
| TM7SF2 | 0.003378203 | 0.586832162 | 0.676474132 |
| VEGFA | -0.004871291 | 0.586832252 | 0.676474132 |
| CHMP5 | 0.004484796 | 0.587033688 | 0.676551628 |
| ZC3H8 | 0.002462756 | 0.588141752 | 0.677673731 |
| MORF4L2 | -0.004482402 | 0.58832878 | 0.677734319 |
| SNRPA | 0.002481919 | 0.588473867 | 0.677746576 |
| AK2 | -0.004273514 | 0.588783579 | 0.677881983 |
| UBA2 | 0.002605775 | 0.588860386 | 0.677881983 |
| PRPF8 | -0.003796756 | 0.591347732 | 0.680589935 |
| HOXC10 | 0.003121735 | 0.591592333 | 0.680716035 |
| EIF4EBP2 | 0.003161083 | 0.592525829 | 0.681634574 |
| CTBS | 0.003997721 | 0.5928556 | 0.681858334 |
| TSPO | -0.005315771 | 0.593292425 | 0.68220509 |
| THEM6 | 0.002369399 | 0.594210136 | 0.683104515 |
| THRAP3 | 0.003829294 | 0.594953117 | 0.683802705 |
| MRPS21 | -0.005660643 | 0.597275087 | 0.686314956 |
| PAICS | 0.002792626 | 0.597620034 | 0.686554658 |
| DEGS2 | -0.002519251 | 0.597903438 | 0.686554658 |
| NKTR | 0.004683731 | 0.597939538 | 0.686554658 |
| GNAI2 | -0.002628755 | 0.598028467 | 0.686554658 |
| DELE1 | -0.002333889 | 0.598300097 | 0.686710107 |
| GCA | 0.002621878 | 0.598523952 | 0.686810662 |
| DYNC1LI2 | 0.003790178 | 0.599031809 | 0.687236994 |
| PIH1D1 | 0.002555121 | 0.601061617 | 0.689286889 |
| MRS2 | -0.002810603 | 0.601190799 | 0.689286889 |
| CISD2 | -0.002941572 | 0.601228814 | 0.689286889 |
| TRAPPC6B | -0.002312519 | 0.601383228 | 0.689307151 |
| SETX | 0.003196495 | 0.602165148 | 0.690046491 |
| RTN3 | -0.004689592 | 0.602764391 | 0.690576203 |
| AGO2 | -0.002340345 | 0.603297717 | 0.691030174 |
| PARD6B | -0.004099621 | 0.603757601 | 0.69138463 |
| ZYG11B | -0.002750203 | 0.603881477 | 0.69138463 |
| TMEM70 | -0.002712279 | 0.60422332 | 0.691618927 |
| RBM5 | 0.003824823 | 0.604518444 | 0.691799654 |
| SLC30A5 | 0.002704665 | 0.604776883 | 0.691938327 |
| ACBD5 | -0.002519433 | 0.604919952 | 0.691944969 |
| RTL8A | -0.002837592 | 0.605588589 | 0.692552649 |
| KHDRBS1 | -0.00404795 | 0.606409013 | 0.693294275 |
| PMF1 | 0.003193055 | 0.606512151 | 0.693294275 |
| YIPF4 | 0.003398895 | 0.607199767 | 0.693922926 |
| SPCS3 | -0.004188994 | 0.608469125 | 0.69521597 |
| SLC35A3 | 0.002673631 | 0.610065401 | 0.696881868 |
| CCT4 | 0.003690635 | 0.611468938 | 0.698326896 |

| | | | |
|---|---|---|---|
| PLD3 | -0.003451389 | 0.612037585 | 0.698778657 |
| PAPOLA | 0.004402741 | 0.612141747 | 0.698778657 |
| RREB1 | 0.002394793 | 0.612304775 | 0.698806514 |
| EIF2A | 0.003008364 | 0.612789726 | 0.699201677 |
| EVL | 0.002415735 | 0.613409356 | 0.699750297 |
| SMTNL2 | 0.002604575 | 0.614001451 | 0.700267266 |
| SDC4 | -0.005922976 | 0.614616528 | 0.700810206 |
| SREBF2 | 0.003378741 | 0.615268431 | 0.701214966 |
| NMT1 | 0.002902652 | 0.615381374 | 0.701214966 |
| PSMC6 | 0.003227614 | 0.615388814 | 0.701214966 |
| ERP29 | 0.004987207 | 0.61650108 | 0.702323603 |
| SLC41A3 | 0.002310607 | 0.617492783 | 0.703294424 |
| KLHDC3 | 0.002708189 | 0.618606004 | 0.704403177 |
| SEC61G | 0.006558522 | 0.6189587 | 0.704645621 |
| EIF1AX | 0.004619812 | 0.619438035 | 0.704794109 |
| NUFIP2 | -0.002974699 | 0.619445458 | 0.704794109 |
| CRACR2B | -0.002213879 | 0.619508569 | 0.704794109 |
| XRN2 | 0.003118767 | 0.619847267 | 0.704932316 |
| RBM23 | -0.002930092 | 0.619909732 | 0.704932316 |
| NCBP3 | 0.002538367 | 0.620564929 | 0.705518225 |
| SOD3 | 0.003407883 | 0.621196668 | 0.706077205 |
| CXXC1 | -0.002248539 | 0.621947468 | 0.706771232 |
| TMEM106B | -0.004167938 | 0.623122776 | 0.707817332 |
| SMIM20 | 0.002864481 | 0.623148843 | 0.707817332 |
| CDK10 | 0.002261264 | 0.624237158 | 0.708893786 |
| SPAG9 | 0.003102878 | 0.624548351 | 0.709087441 |
| RGL3 | 0.002976883 | 0.624743528 | 0.709149318 |
| EPB41 | -0.002416737 | 0.625510026 | 0.709859532 |
| CUL5 | 0.003254256 | 0.626136357 | 0.710410393 |
| CFLAR | 0.004055844 | 0.626480879 | 0.710519758 |
| FBXO25 | 0.002225846 | 0.626514645 | 0.710519758 |
| PRPF31 | 0.002507936 | 0.626920957 | 0.710820635 |
| FBL | 0.002503043 | 0.62768155 | 0.711522981 |
| ACBD3 | 0.002437191 | 0.628579996 | 0.712381241 |
| ITPA | 0.002378163 | 0.63007922 | 0.713919835 |
| GNPTG | 0.002869361 | 0.630379319 | 0.714099359 |
| ABHD11 | -0.002416903 | 0.631281743 | 0.714960967 |
| TRPC4AP | -0.002280771 | 0.632329712 | 0.71578706 |
| SUPT6H | 0.002176244 | 0.632356132 | 0.71578706 |
| WDR61 | -0.002707005 | 0.632485657 | 0.71578706 |
| CAMLG | 0.002390816 | 0.632579121 | 0.71578706 |
| MOB4 | 0.002313563 | 0.633475266 | 0.716297175 |
| PCF11 | -0.002780505 | 0.633496831 | 0.716297175 |
| MRPL47 | -0.00316405 | 0.633577494 | 0.716297175 |
| AGL | -0.00343257 | 0.633598315 | 0.716297175 |
| ABCE1 | -0.002337609 | 0.63401844 | 0.716611425 |
| TM9SF2 | 0.004407951 | 0.634782583 | 0.717314279 |

| | | | |
|---|---|---|---|
| APPL1 | -0.003347033 | 0.635022577 | 0.717424655 |
| FUS | 0.00457014 | 0.635867772 | 0.718218561 |
| PCNP | 0.003167758 | 0.636451523 | 0.71862867 |
| ODR4 | -0.002342629 | 0.636515971 | 0.71862867 |
| TMX1 | 0.002404043 | 0.636679753 | 0.718652627 |
| HEXD | 0.002154969 | 0.637623172 | 0.719448239 |
| RBBP7 | 0.002902526 | 0.637670052 | 0.719448239 |
| TSHZ1 | 0.002185792 | 0.637948906 | 0.719558443 |
| GET3 | 0.002825481 | 0.638053212 | 0.719558443 |
| TMEM72 | 0.005800815 | 0.638600285 | 0.719998674 |
| MCRIP1 | -0.002884655 | 0.638729234 | 0.719998674 |
| ATP2B1 | -0.002939592 | 0.639371657 | 0.720561709 |
| TADA3 | -0.002173471 | 0.639628427 | 0.720689964 |
| SLC7A5 | 0.002692071 | 0.639820166 | 0.720744907 |
| JAG1 | 0.003500794 | 0.640467193 | 0.721312583 |
| EPB41L1 | 0.003142227 | 0.641329814 | 0.722122759 |
| PSMD3 | -0.002250941 | 0.641573935 | 0.722236312 |
| TMEM177 | 0.002074199 | 0.641780617 | 0.722285839 |
| AC018521.5 | 0.002283624 | 0.641904495 | 0.722285839 |
| CLIC1 | -0.005279509 | 0.642138345 | 0.722387725 |
| EXOSC7 | -0.002222451 | 0.642474995 | 0.722605187 |
| LBR | 0.002648548 | 0.642683648 | 0.722678624 |
| VMA21 | 0.002565126 | 0.643010867 | 0.722789511 |
| EAPP | -0.002828697 | 0.643069026 | 0.722789511 |
| C22orf39 | 0.002443605 | 0.643571836 | 0.723193407 |
| NAPA | -0.0032394 | 0.643848142 | 0.723259044 |
| NEFL | -0.002876075 | 0.644024936 | 0.723259044 |
| AKR1A1 | -0.002165274 | 0.644166914 | 0.723259044 |
| MAGEH1 | -0.002491909 | 0.644204148 | 0.723259044 |
| EEF1AKMT2 | -0.002184263 | 0.644427944 | 0.723349202 |
| PSMB6 | -0.004380456 | 0.646236557 | 0.725217829 |
| LGALS3BP | -0.004573509 | 0.646543011 | 0.725400249 |
| PERP | -0.003554259 | 0.647362383 | 0.726157938 |
| DSTN | -0.007455758 | 0.648035655 | 0.726751443 |
| WASL | 0.003054785 | 0.648942621 | 0.7274683 |
| GPR155 | -0.002132811 | 0.648963488 | 0.7274683 |
| SDR39U1 | 0.002896519 | 0.649400883 | 0.727796765 |
| CDK2AP2 | -0.002878946 | 0.65075318 | 0.72904978 |
| GGA2 | 0.002902213 | 0.650808175 | 0.72904978 |
| MGAT4A | 0.003625075 | 0.652066567 | 0.730139411 |
| EMP1 | 0.003182828 | 0.652070547 | 0.730139411 |
| CHID1 | 0.003689367 | 0.652264026 | 0.730193861 |
| GON4L | -0.002096686 | 0.653209411 | 0.730985734 |
| PSMD13 | -0.00237594 | 0.653261402 | 0.730985734 |
| ARHGAP35 | -0.002611581 | 0.654004084 | 0.731654369 |
| NAA20 | 0.003369594 | 0.655123251 | 0.732730059 |
| PAX2 | 0.003391353 | 0.65525632 | 0.732730059 |

| | | | |
|---|---|---|---|
| FAU | 0.010024191 | 0.65541374 | 0.73274355 |
| RNF10 | -0.004097833 | 0.656361619 | 0.733640559 |
| ESF1 | 0.002911696 | 0.657091478 | 0.734293536 |
| FUCA2 | -0.002443861 | 0.657647177 | 0.734751644 |
| RPS18 | 0.0124609 | 0.658529013 | 0.735573843 |
| AL390719.2 | -0.002001728 | 0.659407274 | 0.736391685 |
| RER1 | -0.003838213 | 0.65978939 | 0.73665522 |
| SNRNP25 | 0.002690353 | 0.660316672 | 0.737080678 |
| PRDX2 | -0.00726428 | 0.661759603 | 0.738527819 |
| BRD4 | -0.002823729 | 0.66196562 | 0.738594221 |
| BCAR3 | 0.001984269 | 0.662422941 | 0.738940927 |
| CAND1 | -0.00302121 | 0.663421899 | 0.739891547 |
| SSR2 | -0.004078788 | 0.664720928 | 0.741176332 |
| UBE2D2 | -0.003002097 | 0.665721751 | 0.742128118 |
| HOGA1 | 0.002212903 | 0.666969393 | 0.743354568 |
| SLC1A5 | 0.002372418 | 0.667833045 | 0.744152604 |
| MOCS2 | -0.004686212 | 0.668059667 | 0.744240615 |
| STMP1 | -0.003804677 | 0.668637945 | 0.744720256 |
| ECI1 | -0.004231174 | 0.670607434 | 0.746748856 |
| NFS1 | 0.001907347 | 0.670885064 | 0.746893023 |
| PNPLA2 | 0.002465733 | 0.671065145 | 0.746928549 |
| RNF170 | 0.002095103 | 0.673922362 | 0.749943185 |
| PSMA2 | -0.003756705 | 0.67413848 | 0.750018115 |
| RAB22A | 0.001958285 | 0.674304646 | 0.75003745 |
| HMGB2 | -0.003062201 | 0.675388422 | 0.751077219 |
| GABPB1-IT1 | -0.001854648 | 0.67564128 | 0.751134568 |
| MRPL55 | 0.002592368 | 0.675874852 | 0.751134568 |
| SELENOM | 0.004889086 | 0.675887007 | 0.751134568 |
| GLS | 0.003288742 | 0.676230659 | 0.751350838 |
| FAM229B | 0.002493247 | 0.676525296 | 0.75135674 |
| PSMC1 | 0.002412475 | 0.676550015 | 0.75135674 |
| BBIP1 | 0.002255949 | 0.676683119 | 0.75135674 |
| ELOB | -0.007177261 | 0.677147916 | 0.751707255 |
| RAB10 | 0.003159085 | 0.677764468 | 0.752226042 |
| ARL5A | 0.003199064 | 0.678600921 | 0.75282682 |
| UBE2B | 0.003326797 | 0.678604457 | 0.75282682 |
| KIF22 | 0.002102408 | 0.679184125 | 0.753304109 |
| UPF1 | -0.002192039 | 0.679628952 | 0.753631664 |
| SERINC3 | -0.002766726 | 0.680292854 | 0.754201952 |
| RPL7 | -0.006992282 | 0.684577743 | 0.758785488 |
| ECI2 | 0.003992115 | 0.685445769 | 0.759580594 |
| EIF3J | -0.002649278 | 0.68625994 | 0.760237287 |
| HYI | 0.002065264 | 0.686341958 | 0.760237287 |
| WIPI2 | -0.002206388 | 0.686490802 | 0.760237287 |
| ADH5 | 0.003758606 | 0.688425506 | 0.762090947 |
| DNAJC5 | -0.001802522 | 0.688467005 | 0.762090947 |
| EIF2S1 | -0.001856694 | 0.689155618 | 0.762685724 |

| | | | |
|---|---|---|---|
| DIDO1 | 0.002013254 | 0.689951252 | 0.763398653 |
| ESAM | -0.002483841 | 0.691803944 | 0.765280597 |
| DMAP1 | 0.001829366 | 0.692492189 | 0.765873875 |
| POLR2H | -0.002386272 | 0.693775986 | 0.76712541 |
| BUD23 | -0.002358106 | 0.694387261 | 0.767632935 |
| USP10 | -0.001969482 | 0.695142771 | 0.768299651 |
| STIM2 | 0.001758616 | 0.695836033 | 0.768897291 |
| CDC5L | -0.001867323 | 0.697331329 | 0.770362065 |
| ZNHIT1 | -0.003727788 | 0.697467261 | 0.770362065 |
| DNMT3A | 0.001786011 | 0.698530793 | 0.771367739 |
| SMIM19 | -0.002828333 | 0.701514459 | 0.774492858 |
| DHTKD1 | -0.001814853 | 0.702677388 | 0.7756069 |
| NUDT21 | 0.002397398 | 0.703579439 | 0.776427135 |
| LANCL1 | 0.002393787 | 0.70378032 | 0.776427135 |
| VTI1B | -0.003365386 | 0.703882564 | 0.776427135 |
| CWC15 | 0.002431818 | 0.704268914 | 0.776683351 |
| ZNF91 | -0.002352321 | 0.7052978 | 0.777647902 |
| LGMN | -0.001752749 | 0.706869976 | 0.779161627 |
| PHPT1 | -0.005509308 | 0.706979822 | 0.779161627 |
| CSNK2B | -0.002963201 | 0.70738614 | 0.779408878 |
| CSTB | -0.004379275 | 0.707513395 | 0.779408878 |
| AP2A2 | 0.002787099 | 0.708581386 | 0.780378869 |
| SAFB2 | -0.002329609 | 0.708703523 | 0.780378869 |
| SLC40A1 | 0.002010167 | 0.709604995 | 0.781200869 |
| B4GAT1 | -0.001663141 | 0.712509392 | 0.784227041 |
| CDC40 | -0.002125381 | 0.714693724 | 0.786459521 |
| UBE2J1 | 0.001897877 | 0.715156835 | 0.786797382 |
| GPS2 | -0.002519699 | 0.715878261 | 0.787419226 |
| CLIP1 | 0.00197941 | 0.716308227 | 0.787582826 |
| SLC35F5 | -0.003050522 | 0.716450824 | 0.787582826 |
| PTPMT1 | 0.002277771 | 0.716495703 | 0.787582826 |
| HDHD3 | 0.001603175 | 0.716931106 | 0.787794171 |
| MET | -0.001847898 | 0.717000527 | 0.787794171 |
| FASTK | -0.002363212 | 0.717402554 | 0.788005803 |
| ABHD10 | -0.001668289 | 0.71750578 | 0.788005803 |
| INTS3 | -0.001926277 | 0.71800647 | 0.788252468 |
| SAR1A | 0.002318508 | 0.718189974 | 0.788252468 |
| LIMS1 | -0.001958327 | 0.718199481 | 0.788252468 |
| OSBPL2 | -0.001747902 | 0.718399308 | 0.788300155 |
| DDX6 | 0.002568669 | 0.718689485 | 0.788446941 |
| TM2D1 | 0.00235413 | 0.719805809 | 0.789499801 |
| HCG18 | 0.001813766 | 0.721663115 | 0.791292042 |
| C16orf58 | -0.001767641 | 0.72175378 | 0.791292042 |
| DYNLRB1 | -0.003689349 | 0.722742943 | 0.792204213 |
| NISCH | -0.002160631 | 0.723441547 | 0.792797574 |
| CYP1B1 | -0.004090056 | 0.723866485 | 0.79309084 |
| GOT1 | -0.003291109 | 0.724035587 | 0.793103736 |

| | | | |
|---|---|---|---|
| PEG10 | 0.001865494 | 0.725724447 | 0.794701876 |
| TMEM208 | -0.002829598 | 0.725809847 | 0.794701876 |
| WEE1 | 0.001715946 | 0.726297883 | 0.795063546 |
| BCL7C | -0.003518083 | 0.726871547 | 0.795518773 |
| TRRAP | 0.001589379 | 0.727594195 | 0.796015739 |
| RAP1B | -0.001872071 | 0.727641445 | 0.796015739 |
| MRPL40 | 0.002897983 | 0.728474332 | 0.796607814 |
| SUZ12 | -0.001917046 | 0.72854386 | 0.796607814 |
| GRINA | -0.002721719 | 0.728656741 | 0.796607814 |
| IFITM3 | -0.003183835 | 0.729074737 | 0.796891966 |
| BCAM | -0.004736326 | 0.729934625 | 0.797658887 |
| C4orf48 | 0.003483128 | 0.730381407 | 0.797864171 |
| TSPAN7 | -0.002407919 | 0.73043903 | 0.797864171 |
| AAMP | -0.002174436 | 0.730848877 | 0.798138906 |
| KANSL3 | -0.001685215 | 0.73123931 | 0.798301378 |
| CLNS1A | -0.001920795 | 0.731349276 | 0.798301378 |
| KL | 0.001827478 | 0.731472736 | 0.798301378 |
| SUGT1 | -0.00212709 | 0.732981005 | 0.799774295 |
| DDHD2 | 0.001584404 | 0.733621488 | 0.800299918 |
| MRPS27 | 0.001535983 | 0.73436045 | 0.800767422 |
| HOXB5 | -0.001780229 | 0.734367743 | 0.800767422 |
| ARHGEF12 | -0.002816995 | 0.734552601 | 0.800795774 |
| VDAC3 | 0.002905026 | 0.735525603 | 0.801413563 |
| SLTM | -0.002547024 | 0.735581444 | 0.801413563 |
| CTR9 | -0.001741353 | 0.735596222 | 0.801413563 |
| PON2 | -0.002029815 | 0.736102625 | 0.801680541 |
| PDCD6IP | 0.00256838 | 0.736159338 | 0.801680541 |
| PUM1 | 0.002376926 | 0.737086395 | 0.802516743 |
| HNRNPUL1 | 0.002008448 | 0.737725879 | 0.802961139 |
| MOB1B | 0.001700366 | 0.737813131 | 0.802961139 |
| TMEM50B | 0.002456342 | 0.738223177 | 0.803233981 |
| HIF1A | -0.002664113 | 0.73842559 | 0.803280837 |
| CNOT7 | -0.00204139 | 0.740176352 | 0.805011648 |
| SLC33A1 | 0.001638704 | 0.741314963 | 0.805728422 |
| EEF1B2 | -0.004427881 | 0.741392141 | 0.805728422 |
| ATP11A | -0.001616622 | 0.741407563 | 0.805728422 |
| SEC13 | -0.001501624 | 0.741567688 | 0.805728422 |
| LRATD2 | -0.001712472 | 0.741634572 | 0.805728422 |
| SNRPB | -0.002127949 | 0.742071779 | 0.806029701 |
| ARPP19 | -0.002429703 | 0.74337293 | 0.807269052 |
| TCEAL8 | -0.002203787 | 0.745240952 | 0.809123333 |
| EIF3D | 0.002134741 | 0.745659521 | 0.809403455 |
| HACD2 | 0.001806723 | 0.746636639 | 0.810289623 |
| CLEC11A | 0.002042213 | 0.746862472 | 0.81036025 |
| TXNDC12 | 0.001475488 | 0.747036214 | 0.810374339 |
| MED28 | 0.001520106 | 0.74769979 | 0.810919673 |
| LARP7 | 0.002115696 | 0.747873686 | 0.810933803 |

| | | | |
|---|---|---|---|
| AZIN1 | 0.001540556 | 0.749358544 | 0.812172371 |
| CRYZ | 0.002371738 | 0.749414142 | 0.812172371 |
| APEX1 | 0.002110938 | 0.74949928 | 0.812172371 |
| C7orf50 | -0.002969605 | 0.751825133 | 0.814517623 |
| BAG4 | 0.001402553 | 0.752756848 | 0.815351799 |
| HOTAIRM1 | -0.002479149 | 0.753819774 | 0.816327708 |
| IAH1 | -0.001785617 | 0.75473095 | 0.8171389 |
| ALDH1L1 | 0.002507834 | 0.755739416 | 0.817997843 |
| LMAN2 | -0.002648667 | 0.75584883 | 0.817997843 |
| SIRT5 | -0.001490196 | 0.756101041 | 0.818095159 |
| GADD45B | -0.002116209 | 0.756533231 | 0.818387129 |
| FAM242C | 0.001535576 | 0.756843469 | 0.818545547 |
| LARP4 | -0.001848409 | 0.757004432 | 0.818545547 |
| RPL8 | 0.007455711 | 0.7574033 | 0.818673279 |
| AC073896.2 | -0.001701137 | 0.757447366 | 0.818673279 |
| CCDC28A | 0.001742841 | 0.757815501 | 0.818895592 |
| TRIM26 | -0.001475731 | 0.758132215 | 0.819062258 |
| GTF2H2 | -0.001671979 | 0.758504858 | 0.819289263 |
| TMEM45B | -0.001713504 | 0.759214257 | 0.819879835 |
| KAT8 | -0.00134144 | 0.759455562 | 0.819964765 |
| GNG10 | -0.001976624 | 0.761202493 | 0.821674896 |
| TMEM80 | -0.001492526 | 0.761400754 | 0.821712952 |
| LENG8 | -0.002370998 | 0.761738061 | 0.821901021 |
| RPS25 | 0.006619078 | 0.762088975 | 0.822046056 |
| RABGGTB | 0.001799543 | 0.762198624 | 0.822046056 |
| NUDT16 | -0.001473667 | 0.762804918 | 0.822523977 |
| ARHGEF17 | -0.001340802 | 0.764260532 | 0.82391731 |
| ELP6 | 0.001427063 | 0.764570929 | 0.824075701 |
| MAT2B | 0.001812379 | 0.764882685 | 0.824235488 |
| TSPAN12 | 0.003092926 | 0.765587248 | 0.824818405 |
| SLC39A6 | -0.001518857 | 0.767752611 | 0.826974553 |
| CARS2 | -0.001479327 | 0.769373863 | 0.828485251 |
| KPNA6 | 0.001421903 | 0.769483822 | 0.828485251 |
| SNRNP27 | 0.001389822 | 0.770405158 | 0.829143655 |
| LMO4 | 0.001596357 | 0.770424297 | 0.829143655 |
| HPF1 | -0.001439413 | 0.771041986 | 0.829466926 |
| SKP1 | 0.004628883 | 0.771053762 | 0.829466926 |
| GABPB1-AS1 | 0.002002854 | 0.772113378 | 0.8304296 |
| BCCIP | 0.001659053 | 0.772965877 | 0.831169152 |
| ATP6V1B2 | 0.001313967 | 0.773809547 | 0.831898896 |
| MUC1 | -0.004666903 | 0.774120497 | 0.832055741 |
| TRIOBP | -0.00159623 | 0.775315808 | 0.833162863 |
| CFAP36 | -0.001469376 | 0.775523876 | 0.833208836 |
| PSMA3-AS1 | 0.001803565 | 0.776370439 | 0.833940631 |
| CDC37 | -0.00182917 | 0.779333968 | 0.836862905 |
| STX12 | 0.001299486 | 0.779422999 | 0.836862905 |
| TRNT1 | 0.001244626 | 0.779676645 | 0.836956977 |

| | | | |
|---|---|---|---|
| RRAGA | 0.001656169 | 0.780313928 | 0.837462744 |
| RBM12B | 0.001508994 | 0.780789156 | 0.837794409 |
| HAGHL | -0.001267688 | 0.781747207 | 0.838643897 |
| NDUFA13 | -0.005555929 | 0.78278268 | 0.839505667 |
| PRPF6 | -0.001617276 | 0.782995508 | 0.839505667 |
| HM13 | 0.001776839 | 0.783050118 | 0.839505667 |
| GNB2 | -0.002326174 | 0.783967047 | 0.84030999 |
| ITGB5 | 0.001994049 | 0.784886262 | 0.841116422 |
| KIAA0232 | 0.001876766 | 0.785132483 | 0.841201455 |
| SNF8 | -0.001744474 | 0.785377228 | 0.841284872 |
| UBE2J2 | 0.001224802 | 0.785737746 | 0.841323051 |
| IGSF8 | 0.001270404 | 0.785746662 | 0.841323051 |
| HSPA1B | 0.001820334 | 0.78657347 | 0.842029488 |
| MGST3 | 0.004777962 | 0.788541314 | 0.84366875 |
| ADIPOR2 | 0.001306395 | 0.788642807 | 0.84366875 |
| MRPL23 | 0.001514067 | 0.788956817 | 0.84366875 |
| MAPK1 | -0.001880628 | 0.78904637 | 0.84366875 |
| USP8 | 0.001864026 | 0.789055067 | 0.84366875 |
| EIF3K | 0.004249432 | 0.789108938 | 0.84366875 |
| PPP4R3A | 0.001491764 | 0.7894703 | 0.84387612 |
| MAF1 | -0.001811035 | 0.789949158 | 0.844208968 |
| ARL6IP5 | -0.001918964 | 0.790731239 | 0.844865659 |
| PDHX | -0.00127771 | 0.791234524 | 0.84522425 |
| CALU | -0.001438746 | 0.792166663 | 0.84604071 |
| NEAT1 | -0.004858153 | 0.792944981 | 0.846692575 |
| TCTN1 | 0.001267907 | 0.793219475 | 0.846806305 |
| LMBRD2 | 0.001213827 | 0.795648869 | 0.849219976 |
| FAM210B | 0.001174433 | 0.797015313 | 0.850498347 |
| MICOS13 | -0.003574546 | 0.799060136 | 0.852436571 |
| LSM8 | 0.001678378 | 0.799169854 | 0.852436571 |
| SNX6 | -0.001920769 | 0.800136914 | 0.85325309 |
| DIAPH1 | 0.001660743 | 0.800273876 | 0.85325309 |
| CYHR1 | 0.001855466 | 0.800532326 | 0.853348161 |
| IFNAR1 | -0.001613106 | 0.800706555 | 0.853353434 |
| TAF1D | -0.001677747 | 0.801382704 | 0.853893513 |
| MAP2K1 | -0.001103568 | 0.801843897 | 0.854204371 |
| FAHD2A | 0.001149349 | 0.802953187 | 0.85520537 |
| IMPAD1 | 0.001457419 | 0.803852596 | 0.855982454 |
| TMEM150C | -0.001199483 | 0.804597051 | 0.856574705 |
| GAA | -0.001469391 | 0.804748622 | 0.856574705 |
| SLC25A16 | -0.001145324 | 0.805534467 | 0.857230156 |
| VPS13B-DT | 0.001159101 | 0.806290933 | 0.857854072 |
| CCPG1 | 0.001672873 | 0.807485034 | 0.858607859 |
| STX16 | 0.001657977 | 0.807615813 | 0.858607859 |
| B3GNT2 | -0.001489072 | 0.807636891 | 0.858607859 |
| DEGS1 | -0.001195093 | 0.807680712 | 0.858607859 |
| ATG101 | -0.001125297 | 0.808085403 | 0.858856951 |

| | | | |
|---|---|---|---|
| DDX39B | 0.001883347 | 0.808575258 | 0.859196432 |
| TMEM37 | -0.002612033 | 0.808872002 | 0.859330614 |
| RTF1 | 0.001598621 | 0.810398587 | 0.860631963 |
| PPIB | -0.00307777 | 0.810438391 | 0.860631963 |
| LETM1 | -0.001360427 | 0.8109561 | 0.861000358 |
| PLXNB1 | 0.002220662 | 0.81211558 | 0.862049829 |
| HRG | 0.001594534 | 0.812707074 | 0.862496075 |
| CZIB | -0.0013605 | 0.814364141 | 0.86407275 |
| LINC02381 | 0.002159605 | 0.814837613 | 0.864393184 |
| PTCD3 | -0.00139044 | 0.817084293 | 0.866594135 |
| TSEN15 | 0.001048634 | 0.817266699 | 0.866605265 |
| FAM3A | 0.001149454 | 0.817467213 | 0.866635588 |
| AIMP1 | 0.00168658 | 0.817898213 | 0.866910196 |
| PAX8 | 0.001676626 | 0.81881722 | 0.867618756 |
| TSPAN14 | -0.001445288 | 0.819008456 | 0.867618756 |
| IST1 | 0.001505417 | 0.819083051 | 0.867618756 |
| MYL12B | 0.003142597 | 0.81984105 | 0.868140915 |
| CSNK2A1 | -0.001625669 | 0.82012459 | 0.868140915 |
| PHGDH | 0.001410456 | 0.820185674 | 0.868140915 |
| WDR18 | 0.00110135 | 0.820264864 | 0.868140915 |
| SRSF5 | 0.002920056 | 0.820733073 | 0.868199931 |
| TMEM14B | 0.00238347 | 0.820748035 | 0.868199931 |
| ZBTB10 | 0.001076632 | 0.820837308 | 0.868199931 |
| UQCC3 | 0.001643804 | 0.822484718 | 0.869588668 |
| HPRT1 | -0.001671332 | 0.822495292 | 0.869588668 |
| NIT2 | -0.001046125 | 0.822883315 | 0.869722415 |
| PHB2 | -0.001816496 | 0.822966856 | 0.869722415 |
| HSD17B8 | 0.00114583 | 0.823412704 | 0.870011201 |
| WDR13 | -0.001518376 | 0.825670238 | 0.871856432 |
| PAXX | -0.001676756 | 0.825801969 | 0.871856432 |
| ABHD14A | 0.001230635 | 0.825955038 | 0.871856432 |
| PPARA | -0.001624952 | 0.826157557 | 0.871856432 |
| SEC23B | 0.001105346 | 0.826159982 | 0.871856432 |
| MRPS5 | -0.0016461 | 0.826196821 | 0.871856432 |
| SYNCRIP | -0.001547964 | 0.826505576 | 0.871862771 |
| FUNDC2 | -0.001576255 | 0.82667695 | 0.871862771 |
| RNF152 | -0.001284597 | 0.826721691 | 0.871862771 |
| GSTK1 | 0.002184563 | 0.82704694 | 0.872023348 |
| WBP4 | 0.001036036 | 0.827287927 | 0.872095031 |
| SAFB | 0.001294862 | 0.827653131 | 0.872297603 |
| FUBP3 | -0.000933594 | 0.828522515 | 0.873031354 |
| TPGS1 | -0.001067174 | 0.829655936 | 0.874042962 |
| SLC25A23 | -0.001144151 | 0.832530179 | 0.876887721 |
| PRKAR1B | 0.001053224 | 0.832879399 | 0.877072289 |
| SUN1 | 0.001408827 | 0.833301689 | 0.877333712 |
| CCDC59 | 0.001038352 | 0.833926361 | 0.877808058 |
| RAD17 | -0.000999962 | 0.834680214 | 0.878400438 |

| | | | |
|---|---|---|---|
| SEPTIN7 | 0.001644255 | 0.834837631 | 0.878400438 |
| PLEKHG3 | 0.001019511 | 0.836316052 | 0.879772375 |
| CMYA5 | 0.00137343 | 0.83668406 | 0.87997587 |
| PTGR2 | 0.001045937 | 0.83690735 | 0.880027108 |
| RNF130 | 0.001180679 | 0.837487179 | 0.880453154 |
| SRP72 | -0.001094722 | 0.83784869 | 0.880475256 |
| LIPA | -0.00108795 | 0.837857529 | 0.880475256 |
| CAPG | 0.001339748 | 0.838177144 | 0.880627549 |
| DGCR6L | -0.00162892 | 0.838379838 | 0.880656963 |
| BIVM | -0.000924249 | 0.838826016 | 0.880942072 |
| DNM2 | 0.001496847 | 0.839391291 | 0.881267017 |
| ILVBL | -0.001376151 | 0.839485066 | 0.881267017 |
| MIA3 | 0.001131202 | 0.84013119 | 0.881761676 |
| ZDHHC4 | -0.000941382 | 0.840960476 | 0.882309863 |
| MRPL21 | 0.001253336 | 0.841003549 | 0.882309863 |
| ABLIM1 | 0.002301991 | 0.842159205 | 0.883338442 |
| PPFIA1 | 0.001018525 | 0.842780896 | 0.883684467 |
| NUMA1 | -0.00144462 | 0.842839698 | 0.883684467 |
| GFPT1 | 0.001024657 | 0.844342092 | 0.885075585 |
| ARL8B | 0.001142725 | 0.845477084 | 0.886081077 |
| TMEM219 | 0.00162817 | 0.845677899 | 0.886107314 |
| SF3B3 | 0.000926433 | 0.846097656 | 0.886362902 |
| SELENBP1 | -0.001708401 | 0.847093099 | 0.887221341 |
| FARSA | 0.000864497 | 0.847682172 | 0.887653891 |
| NIBAN2 | -0.000839261 | 0.848335989 | 0.888154043 |
| SP1 | -0.000934845 | 0.849275195 | 0.888952712 |
| BUD31 | 0.001465557 | 0.852086111 | 0.891709795 |
| COPS8 | -0.000894971 | 0.85259006 | 0.892051991 |
| FRG1 | 0.000904735 | 0.853200223 | 0.892505151 |
| TMED5 | 0.000975315 | 0.853997464 | 0.89315378 |
| AL451165.2 | 0.000912848 | 0.85433757 | 0.893324143 |
| AKT2 | -0.000943574 | 0.855884101 | 0.894755652 |
| BCL7A | 0.001078731 | 0.860359155 | 0.899046154 |
| CFAP410 | 0.000969482 | 0.86041633 | 0.899046154 |
| PI4KA | -0.000874242 | 0.860523248 | 0.899046154 |
| ALG2 | -0.000841319 | 0.860909139 | 0.899262944 |
| CUEDC2 | -0.001036727 | 0.861694157 | 0.899896466 |
| PRSS23 | 0.000818344 | 0.863648624 | 0.901750769 |
| RPL12 | -0.003914584 | 0.863908142 | 0.901834944 |
| TP53I13 | -0.000874867 | 0.866048327 | 0.903748293 |
| NDUFS7 | -0.002084204 | 0.866099584 | 0.903748293 |
| PRRC2B | -0.001177246 | 0.866370014 | 0.903843386 |
| PIN4 | 0.001195754 | 0.866766016 | 0.904069415 |
| NAXE | 0.001220155 | 0.867203012 | 0.904338102 |
| RCN1 | 0.000858177 | 0.867626118 | 0.904592194 |
| PSIP1 | 0.000930411 | 0.867889551 | 0.904679741 |
| PRCP | 0.001440839 | 0.868728032 | 0.905366551 |

| | | | |
|---|---|---|---|
| KBTBD11 | -0.000904121 | 0.869044565 | 0.905509229 |
| MARC2 | 0.000983879 | 0.869243112 | 0.905528937 |
| SBNO1 | 0.000918808 | 0.869478987 | 0.905587516 |
| IGFBP7 | 0.004583275 | 0.86991908 | 0.905858724 |
| BCL2L1 | -0.000970433 | 0.870948936 | 0.906547554 |
| ARGLU1 | 0.001859707 | 0.871269632 | 0.906547554 |
| SMARCA5 | -0.001067653 | 0.871365719 | 0.906547554 |
| TTC1 | -0.000907969 | 0.871392131 | 0.906547554 |
| MMUT | 0.001124938 | 0.871479756 | 0.906547554 |
| PIP4P2 | 0.000789497 | 0.871765985 | 0.906627659 |
| C15orf61 | -0.000900434 | 0.871916463 | 0.906627659 |
| ARPC4 | 0.001013911 | 0.872269901 | 0.90680812 |
| RAB11A | 0.001395657 | 0.874715986 | 0.909038704 |
| MAL | -0.003131278 | 0.874870537 | 0.909038704 |
| ACTR6 | -0.000709442 | 0.874956515 | 0.909038704 |
| SEC63 | -0.000970194 | 0.875666166 | 0.909289554 |
| FAM174A | -0.000746521 | 0.87583724 | 0.909289554 |
| UBE2G1 | 0.000703274 | 0.875849077 | 0.909289554 |
| FBXO21 | -0.001237996 | 0.875919475 | 0.909289554 |
| LRPAP1 | 0.001398734 | 0.876736874 | 0.909950707 |
| NUP42 | -0.00070722 | 0.877053778 | 0.910092239 |
| ZFYVE21 | -0.000753479 | 0.878268755 | 0.911165424 |
| PSMD7 | 0.000681143 | 0.87877337 | 0.911501349 |
| MRPS30 | 0.000704824 | 0.880130317 | 0.91272103 |
| RBCK1 | -0.000849545 | 0.881847427 | 0.914313632 |
| PTER | 0.000707909 | 0.883037665 | 0.91520214 |
| PFDN5 | 0.00255437 | 0.883067488 | 0.91520214 |
| GK5 | 0.000656603 | 0.884451133 | 0.916447721 |
| OARD1 | -0.000908445 | 0.88463493 | 0.916449791 |
| WASHC3 | 0.000899968 | 0.885213895 | 0.916861156 |
| SCIN | 0.001057569 | 0.885764121 | 0.91690404 |
| CLCNKB | -0.002328242 | 0.885778701 | 0.91690404 |
| CACYBP | -0.000916532 | 0.885800967 | 0.91690404 |
| ABHD12 | -0.000830838 | 0.886201764 | 0.917130588 |
| KMT2A | -0.001010144 | 0.886695279 | 0.917452977 |
| SNHG6 | 0.001270054 | 0.887269094 | 0.917710387 |
| SNHG16 | -0.001011339 | 0.887308159 | 0.917710387 |
| ZNF844 | 0.000677434 | 0.887702565 | 0.917929976 |
| SLC2A11 | 0.000659911 | 0.888101604 | 0.918129079 |
| TRHDE-AS1 | 0.000701163 | 0.888259377 | 0.918129079 |
| PSMD8 | -0.001154168 | 0.889794041 | 0.919450408 |
| MRPL24 | -0.000766264 | 0.889902508 | 0.919450408 |
| PLGRKT | -0.000887698 | 0.890683748 | 0.920069011 |
| CD2BP2 | -0.00060998 | 0.891134466 | 0.920346004 |
| UBE2R2 | 0.000736238 | 0.891691597 | 0.920732761 |
| SULT1A1 | 0.00081189 | 0.892414986 | 0.921290998 |
| RNF145 | -0.000720205 | 0.893737872 | 0.922467775 |

| | | | |
|---|---|---|---|
| EDF1 | 0.002200661 | 0.894456662 | 0.923020682 |
| NFYB | 0.000723513 | 0.8970345 | 0.925491387 |
| MRPL43 | -0.000980924 | 0.897846968 | 0.926140079 |
| CREBZF | -0.00070564 | 0.898502089 | 0.926626234 |
| AL136038.5 | -0.000614655 | 0.899181559 | 0.927137296 |
| CBX1 | -0.000703445 | 0.900007018 | 0.927671756 |
| FAM92A | -0.000594832 | 0.900067955 | 0.927671756 |
| NBEAL2 | 0.000563401 | 0.90030805 | 0.927729534 |
| REEP5 | 0.001297695 | 0.900896537 | 0.928123436 |
| GNPAT | -0.000583207 | 0.901058539 | 0.928123436 |
| COPE | 0.001109663 | 0.901494581 | 0.928382877 |
| TXNDC15 | 0.000652432 | 0.901973935 | 0.928686807 |
| WIPF2 | 0.000590429 | 0.902202205 | 0.928720042 |
| AGRN | -0.000901148 | 0.902538844 | 0.928720042 |
| GRB2 | 0.000643662 | 0.902558914 | 0.928720042 |
| CLPX | 0.000666843 | 0.903212319 | 0.929202715 |
| RSBN1 | 0.000612415 | 0.904451456 | 0.930287654 |
| SCAND1 | -0.001216487 | 0.905274758 | 0.930862905 |
| C5orf15 | -0.000574135 | 0.905405262 | 0.930862905 |
| TMF1 | 0.000689193 | 0.905841399 | 0.930862905 |
| CUEDC1 | 0.000540395 | 0.905930072 | 0.930862905 |
| UBE2D3 | -0.001179644 | 0.905934023 | 0.930862905 |
| METRN | -0.000943079 | 0.906185211 | 0.930931251 |
| PJA2 | -0.000946159 | 0.90641661 | 0.930979244 |
| HMGN2 | 0.001373904 | 0.90698997 | 0.931378374 |
| KDM5A | 0.000711982 | 0.908340942 | 0.9325757 |
| AMT | -0.000564496 | 0.909672937 | 0.933753059 |
| RPSA | -0.00166748 | 0.910366666 | 0.934274911 |
| MBOAT7 | 0.000497066 | 0.910652467 | 0.934377994 |
| GABARAP | -0.001749622 | 0.91104188 | 0.934587325 |
| ZNF506 | -0.000511677 | 0.911886017 | 0.935262953 |
| COMMD2 | 0.000563154 | 0.912473908 | 0.935675543 |
| POLR2G | -0.000496774 | 0.913018061 | 0.936043125 |
| API5 | -0.000607697 | 0.913236797 | 0.93607656 |
| CCDC186 | 0.000782488 | 0.913422059 | 0.93607656 |
| IP6K2 | 0.000646042 | 0.914858537 | 0.937316274 |
| PHF6 | -0.000492908 | 0.915120575 | 0.937316274 |
| USF3 | 0.000486258 | 0.915276251 | 0.937316274 |
| COMT | -0.001215965 | 0.915375524 | 0.937316274 |
| CDS1 | 0.000517923 | 0.917744062 | 0.939550734 |
| TOP1 | 0.000769138 | 0.9185828 | 0.939975791 |
| NUDT22 | -0.000675955 | 0.918614451 | 0.939975791 |
| MRPS34 | 0.000917493 | 0.918718652 | 0.939975791 |
| PPP1R10 | 0.000570536 | 0.920306541 | 0.941228985 |
| EIF4E | -0.000603197 | 0.920316935 | 0.941228985 |
| ATP6V1E1 | -0.000693399 | 0.921754206 | 0.942507699 |
| LAMTOR4 | -0.00112356 | 0.922768011 | 0.94335298 |

| | | | |
|---|---|---|---|
| NDUFA3 | -0.00152743 | 0.923236537 | 0.943640589 |
| SCAMP1 | -0.000530009 | 0.925038326 | 0.945065814 |
| SOSTDC1 | -0.000672997 | 0.925062851 | 0.945065814 |
| ARMCX2 | -0.000419154 | 0.925193373 | 0.945065814 |
| NFE2L2 | -0.000933236 | 0.925448895 | 0.945135308 |
| ANAPC16 | -0.001121265 | 0.925703544 | 0.945203882 |
| NONO | 0.000636718 | 0.926185144 | 0.945330012 |
| RFNG | -0.000429771 | 0.926206115 | 0.945330012 |
| FAT1 | -0.000548977 | 0.926389656 | 0.945330012 |
| SAMM50 | 0.000426407 | 0.927000907 | 0.94576231 |
| ASL | -0.000459923 | 0.927567918 | 0.946149306 |
| ZNF302 | 0.000478727 | 0.928012986 | 0.94636609 |
| TMEM218 | 0.000391224 | 0.928155911 | 0.94636609 |
| AKR7A2 | 0.000558106 | 0.929149744 | 0.947070322 |
| SEC11A | 0.000836704 | 0.929222339 | 0.947070322 |
| SCPEP1 | -0.000655435 | 0.930138375 | 0.94781232 |
| RALY | 0.000475938 | 0.930616975 | 0.948108362 |
| AC010642.2 | -0.000405288 | 0.931042197 | 0.948349913 |
| FNIP2 | -0.000920396 | 0.931536378 | 0.948381235 |
| HDHD2 | -0.000412555 | 0.931643299 | 0.948381235 |
| RPP21 | -0.000526226 | 0.931784466 | 0.948381235 |
| CPSF7 | -0.00040669 | 0.931825483 | 0.948381235 |
| SEPTIN8 | 0.000373818 | 0.932322184 | 0.948695222 |
| SRSF6 | -0.000754777 | 0.933713173 | 0.949918891 |
| TSPAN4 | 0.000422589 | 0.934232117 | 0.950255065 |
| STXBP2 | -0.000356697 | 0.935172418 | 0.9510196 |
| TMEM259 | -0.000588638 | 0.935709734 | 0.951374096 |
| PLPP5 | 0.000535413 | 0.939470115 | 0.955004809 |
| ATMIN | -0.000339447 | 0.939779176 | 0.955126376 |
| PPIE | -0.00034743 | 0.942083701 | 0.957275537 |
| ANAPC5 | -0.00047891 | 0.943429689 | 0.958450033 |
| RNF24 | -0.000318439 | 0.94411598 | 0.958953991 |
| VPS26A | -0.00035227 | 0.944512007 | 0.959076453 |
| CCNG1 | 0.000382078 | 0.944617058 | 0.959076453 |
| RPL23A | -0.001242227 | 0.945121755 | 0.959395644 |
| TPD52L1 | 0.000422647 | 0.945321774 | 0.959405489 |
| DAP | 0.000554924 | 0.946639457 | 0.960549417 |
| CNPY2 | 0.000588363 | 0.947057347 | 0.960780053 |
| AFG3L2 | 0.000549817 | 0.94755326 | 0.961089735 |
| RHEB | -0.000581651 | 0.948050609 | 0.961400748 |
| SFXN4 | 0.000286572 | 0.94849347 | 0.961597013 |
| RNF167 | 0.000347128 | 0.948987136 | 0.961597013 |
| PCYT2 | -0.000401842 | 0.949002925 | 0.961597013 |
| EIF4E2 | -0.000304388 | 0.949007169 | 0.961597013 |
| AMOTL2 | -0.000317322 | 0.949527238 | 0.961886678 |
| XPNPEP3 | 0.000275068 | 0.949674667 | 0.961886678 |
| C1QBP | -0.000552945 | 0.951261854 | 0.963300725 |

| | | | |
|---|---|---|---|
| INTS1 | 0.000261901 | 0.953456349 | 0.965329074 |
| DCAF11 | -0.000314715 | 0.954087034 | 0.965773642 |
| TBCB | 0.000352419 | 0.954473622 | 0.965970996 |
| IMPACT | 0.000262034 | 0.954955492 | 0.96626468 |
| RBMX | -0.000491256 | 0.955724271 | 0.966803126 |
| ERBB2 | -0.000485138 | 0.955871212 | 0.966803126 |
| DTX3 | -0.000242788 | 0.956513772 | 0.967205369 |
| ARFRP1 | -0.000250897 | 0.956652642 | 0.967205369 |
| ULK3 | -0.000267624 | 0.957069976 | 0.967433277 |
| CTSZ | -0.000303503 | 0.958147149 | 0.968327943 |
| RBM4 | 0.000390522 | 0.95856252 | 0.968489002 |
| MSMO1 | 0.000258839 | 0.95869076 | 0.968489002 |
| NUDT16L1 | -0.000311452 | 0.959197359 | 0.968806629 |
| CIAO2A | 0.000314396 | 0.959525084 | 0.968943499 |
| NPM1 | 0.000726651 | 0.960902475 | 0.970140071 |
| CHMP4B | -0.000256639 | 0.962603837 | 0.971663185 |
| PHYHD1 | 0.00023797 | 0.963343011 | 0.972214638 |
| MSRB2 | -0.000297621 | 0.963742942 | 0.972423573 |
| FAM50A | 0.000207409 | 0.965101891 | 0.973599887 |
| TERF1 | 0.000197843 | 0.965771111 | 0.974080066 |
| JUP | -0.00037694 | 0.966136603 | 0.974253774 |
| CTDSP1 | 0.00025127 | 0.967945977 | 0.97587471 |
| GPN3 | -0.000181082 | 0.968131209 | 0.97587471 |
| CTDSP2 | -0.000245614 | 0.968730712 | 0.97628379 |
| TRIM44 | 0.000262919 | 0.970352392 | 0.977722648 |
| MAN2C1 | -0.000157292 | 0.972079221 | 0.979266858 |
| PRPF40A | 0.000226535 | 0.973145743 | 0.980145392 |
| YES1 | -0.000150416 | 0.974186676 | 0.980997809 |
| GNAI3 | 0.000165594 | 0.975438682 | 0.982062392 |
| PUF60 | -0.000173602 | 0.976433389 | 0.982867554 |
| THYN1 | -0.000142028 | 0.976695851 | 0.982935473 |
| COPA | -0.000203789 | 0.977252772 | 0.983079858 |
| UBE4A | 0.000147738 | 0.97733756 | 0.983079858 |
| S100A16 | -0.000138246 | 0.97742437 | 0.983079858 |
| ETFB | 0.000301993 | 0.978652843 | 0.984107298 |
| PLEKHA1 | 0.000121633 | 0.978836341 | 0.984107298 |
| PSMA1 | -0.000183567 | 0.980549099 | 0.985489131 |
| CFAP97 | -0.000130388 | 0.980601762 | 0.985489131 |
| COX20 | 0.000261798 | 0.98130192 | 0.985981934 |
| HERC2 | -0.000144114 | 0.981659554 | 0.985981934 |
| UPF3A | 0.000164438 | 0.981678898 | 0.985981934 |
| AAAS | 0.000101933 | 0.982382002 | 0.986491568 |
| IFNGR2 | -9.65863E-05 | 0.982596411 | 0.986506513 |
| SLC25A6 | 0.000343994 | 0.982788277 | 0.986506513 |
| NAPG | -9.59392E-05 | 0.983189437 | 0.986712712 |
| PSMC3 | -0.000122088 | 0.984277801 | 0.987608359 |
| CCND3 | 8.36333E-05 | 0.985739781 | 0.988878455 |

| | | | |
|---|---|---|---|
| ZFYVE16 | -0.000105751 | 0.986590736 | 0.989486819 |
| SVIL | -9.80737E-05 | 0.98673879 | 0.989486819 |
| DPP8 | 6.8839E-05 | 0.988422582 | 0.990941531 |
| SORL1 | -0.000135045 | 0.988582614 | 0.990941531 |
| RPL27A | 0.000235815 | 0.989064127 | 0.99122709 |
| S100A13 | -0.000111786 | 0.990667487 | 0.992636613 |
| RCN2 | 9.62964E-05 | 0.991179063 | 0.99295184 |
| SEC31A | -8.77727E-05 | 0.991404912 | 0.992980759 |
| TJP2 | -4.00797E-05 | 0.993934821 | 0.995263585 |
| RABEP1 | -3.99513E-05 | 0.994078982 | 0.995263585 |
| RPS19BP1 | -5.11946E-05 | 0.995442212 | 0.996430539 |
| FAM204A | -2.98792E-05 | 0.99575187 | 0.99654262 |
| ARIH2 | 1.756E-05 | 0.997303666 | 0.997897535 |
| TMSB10 | 5.08604E-05 | 0.997613787 | 0.998009744 |
| OTUB1 | 1.47286E-05 | 0.998005719 | 0.998203736 |
| DPY30 | -1.56798E-05 | 0.998407625 | 0.998407625 |

**Supplementary Table S4. Shared enhanced and suppressed pathways in PT and TAL. KEGG 2021 enrichment analysis of 863 suppressed and 222 enhanced genes in both PT and TAL.**

| **Enhanced pathways** | **Representative genes** |
|---|---|
| Ubiquitin associated proteolysis | HERC4; UBE2W; MAP3K1; HERC1; UBE2E2; NEDD4L; WWP1; BIRC6; PIAS1 |
| Neurotrophin signaling/GH secretion | MAPK10; MAP3K1; GNAQ; ITPR2; PIK3CB; SOS1; SOS2:PTK2 |
| FOXO signaling | MAPK10; PRKAA2; INSR; PIK3CB; FOXO3; SOS1; SOS2; IGF1R |
| AMPK signaling | PRKAA2; INSR; PIK3CB; PPP2R3A; FOXO3; PPARGC1A; IGF1R |
| **Suppressed pathways** | Representative genes |
| Oxidative phosphorylation | NDUFB9; COX7B; NDUFB7; NDUFB10; UQCRB; NDUFB6; NDUFA12; NDUFB5; NDUFB4; NDUFB3; NDUFB2; ATP5MC3; COX7A2; UQCR11; COX5B; COX7C; COX5A; UQCRH; ATP5MC1; ATP5F1A; ATP5F1B; UQCRFS1; CYC1; ATP6V0E2; ATP5MG; ATP6V1F; ATP5PF; NDUFA8; ATP6V1G1; NDUFA6; NDUFA5; SDHC; NDUFC1; SDHD; SDHA; COX6C; COX6B1; COX7A2L; UQCRQ; NDUFS6; NDUFS5; NDUFAB1; UQCRC1; ATP5PO; NDUFS1; ATP6V0D1; ATP6V0C |
| TCA Cycle | MDH2; IDH2; OGDHL; SDHC; PDHB; SDHD; PCK1; SDHA |
| Glycolysis/Gluconeogenesis | LDHA; PFKL; TPI1; PKM; ALDH2; PGAM1; ENO1; PDHB; PCK1; GAPDH; ALDH9A1 |
| Glutathione metabolism | GGT6; GPX4; ODC1; IDH2; LAP3; PGD; PRDX6 |

ABL1 - ABL Proto-Oncogene 1, Non-Receptor Tyrosine Kinase; BCL2 - B-Cell CLL/Lymphoma 2; BIRC6 - Baculoviral IAP Repeat Containing 6; FOXO3 - Forkhead Box O3; HERC1 - HECT And RLD Domain Containing E3 Ubiquitin Protein Ligase Family Member 1; HERC4 - HECT And RLD Domain Containing E3 Ubiquitin Protein Ligase Family Member 4; IGF1R - Insulin-Like Growth Factor 1 Receptor; INSR - Insulin Receptor; MAP3K1 - Mitogen-Activated Protein Kinase Kinase 1; MAPK10 - Mitogen-Activated Protein Kinase 10; NEDD4L - Neural Precursor Cell Expressed, Developmentally Down-Regulated 4-Like; PIAS - Protein Inhibitor of Activated STAT; PIK3CB - Phosphatidylinositol-4,5-Bisphosphate 3-Kinase Catalytic Subunit Beta; PPARGC1A - Peroxisome Proliferator-Activated Receptor Gamma Coactivator 1 Alpha; PRKAA2 - Protein Kinase AMP-Activated Catalytic Subunit Alpha 2; PPP2R3A - Protein Phosphatase 2 Regulatory Subunit B'' Alpha; SOS1 - Son of Sevenless Homolog 1; SOS2 - Son of Sevenless Homolog 2; UBE2E2 - Ubiquitin Conjugating Enzyme E2 E2; UBE2W - Ubiquitin Conjugating Enzyme E2 W; WWP1 - WW Domain Containing E3 Ubiquitin Protein Ligase 1 ALDH2 - Aldehyde Dehydrogenase 2; ALDH9A1 - Aldehyde Dehydrogenase 9 Family Member A1; ATP5F1A - ATP Synthase F1 Subunit Alpha; ATP5F1B - ATP Synthase F1 Subunit Beta; ATP5MC1 - ATP Synthase Membrane Subunit C1; ATP5MC3 - ATP Synthase Membrane Subunit C3; ATP5MG - ATP Synthase Membrane Subunit G; ATP5PF - ATP Synthase Peripheral Stalk Subunit F6; ATP5PO - ATP Synthase Peripheral Stalk Subunit OSCP; ATP6V0C - ATPase H+ Transporting V0 Subunit C; ATP6V0D1 - ATPase H+ Transporting V0 Subunit D1; ATP6V0E2 - ATPase H+ Transporting V0 Subunit E2; ATP6V1F - ATPase H+ Transporting V1 Subunit F; ATP6V1G1 - ATPase H+ Transporting V1 Subunit G1; COX5A - Cytochrome C Oxidase Subunit 5A; COX5B - Cytochrome C Oxidase Subunit 5B; COX6B1 - Cytochrome C Oxidase Subunit 6B1; COX6C - Cytochrome C Oxidase Subunit 6C; COX7A2 - Cytochrome C Oxidase Subunit 7A2; COX7A2L - Cytochrome C Oxidase Subunit 7A2 Like; COX7B - Cytochrome C Oxidase Subunit 7B; COX7C - Cytochrome C Oxidase Subunit 7C; CYC1 - Cytochrome C1; ENO1 - Enolase 1; GAPDH - Glyceraldehyde-3-Phosphate Dehydrogenase; GGT6 - Gamma-Glutamyltransferase 6; GPX4 - Glutathione Peroxidase 4; IDH2 - Isocitrate Dehydrogenase 2; LAP3 - Leucine Aminopeptidase 3; LDHA - Lactate Dehydrogenase A; MDH2 - Malate Dehydrogenase 2; NDUFA12 - NADH:Ubiquinone Oxidoreductase Subunit A12; NDUFA5 - NADH:Ubiquinone Oxidoreductase Subunit A5; NDUFA6 - NADH:Ubiquinone Oxidoreductase Subunit A6; NDUFA8 - NADH:Ubiquinone Oxidoreductase Subunit A8; NDUFAB1 - NADH:Ubiquinone Oxidoreductase Complex Assembly Factor 1; NDUFB10 - NADH:Ubiquinone Oxidoreductase Subunit B10; NDUFB2 - NADH:Ubiquinone Oxidoreductase Subunit B2; NDUFB3 - NADH:Ubiquinone Oxidoreductase Subunit B3; NDUFB4 - NADH:Ubiquinone Oxidoreductase Subunit B4; NDUFB5 - NADH:Ubiquinone Oxidoreductase Subunit B5; NDUFB6 - NADH:Ubiquinone Oxidoreductase Subunit B6; NDUFB7 - NADH:Ubiquinone Oxidoreductase Subunit B7; NDUFB9 - NADH:Ubiquinone Oxidoreductase Subunit B9; NDUFC1 - NADH:Ubiquinone Oxidoreductase Subunit C1; NDUFS1 - NADH:Ubiquinone Oxidoreductase Core Subunit S1; NDUFS5 - NADH:Ubiquinone Oxidoreductase Subunit S5; NDUFS6 - NADH:Ubiquinone Oxidoreductase Subunit S6; ODC1 - Ornithine Decarboxylase 1; OGDHL - Oxoglutarate Dehydrogenase Like; PCK1 - Phosphoenolpyruvate Carboxykinase 1; PDHB - Pyruvate Dehydrogenase E1 Beta Subunit; PFKL - Phosphofructokinase, Liver Type; PGAM1 - Phosphoglycerate Mutase 1; PGD - Phosphogluconate Dehydrogenase; PIAS - Protein Inhibitor of Activated STAT; PKM - Pyruvate Kinase, Muscle; PRDX6 - Peroxiredoxin 6; PPP2R3A - Protein Phosphatase 2 Regulatory Subunit B'' Alpha; SDHA - Succinate Dehydrogenase Complex Flavoprotein Subunit A; SDHC - Succinate Dehydrogenase Complex Subunit C; SDHD - Succinate Dehydrogenase Complex Subunit D; SOS1 - Son of Sevenless Homolog 1; SOS2 - Son of Sevenless Homolog 2; TPI1 - Triosephosphate Isomerase 1; UBE2E2 - Ubiquitin Conjugating Enzyme E2 E2; UBE2W - Ubiquitin Conjugating Enzyme E2 W; UQCR11 - Ubiquinol-Cytochrome C Reductase Complex Assembly Factor 11; UQCRB - Ubiquinol-Cytochrome C Reductase Binding Protein; UQCRC1 - Ubiquinol-Cytochrome C Reductase Core Protein 1; UQCRFS1 - Ubiquinol-Cytochrome C Reductase Rieske Iron-Sulfur Polypeptide 1; UQCRH - Ubiquinol-Cytochrome C Reductase Complex Assembly Factor H; UQCRQ - Ubiquinol-Cytochrome C Reductase Complex Assembly Factor Q; WWP1 - WW Domain Containing E3 Ubiquitin Protein Ligase

**Supplementary Table S5a. All suppressed genes in the PT only, TAL only, and shared to identify overlap**

| Common genes | Only PT | Only TAL |
|---|---|---|
| ABHD17A | A4GALT | ABCA2 |
| AC244090.1 | AAK1 | ABI3 |
| ACAA1 | AAMDC | ACADM |
| ACADVL | ABCB1 | ACO2 |
| ACP1 | ABCD4 | ACSL4 |
| ACTB | ABCE1 | ACSS1 |
| ACTG1 | ABHD10 | ACTR2 |
| ACTN4 | ABI2 | AFMID |
| ADGRG1 | ABRACL | AHCYL1 |
| ADIPOR1 | AC087482.1 | AK1 |
| AGPAT3 | AC100810.1 | AKR1B1 |
| AIF1L | ACAA2 | AKT1 |
| AKIRIN1 | ACAD8 | AMFR |
| ALDH2 | ACBD3 | ANKLE2 |
| ALDH4A1 | ACE2 | ANKRD13A |
| ALDH9A1 | ACO1 | ANKRD9 |
| ALKBH5 | ACOT2 | ANXA11 |
| ANAPC15 | ACOX1 | AP002360.1 |
| ANP32A | ACP5 | AP1B1 |
| ANP32B | ACSL1 | APMAP |
| ANP32E | ACTR3 | ARCN1 |
| AOC1 | ADAM9 | ARHGDIA |
| APH1A | ADAR | ATG3 |
| ARF1 | ADH5 | ATP1B2 |
| ARF3 | ADH6 | ATP5MD |
| ARL1 | ADI1 | ATP5PB |
| ARL16 | ADIRF | ATP6V1A |
| ARL2BP | ADM2 | ATP6V1B1 |
| ARL8A | ADRM1 | BEX5 |
| ARMCX3 | AFG3L2 | BMI1 |
| ARPC5 | AGGF1 | BOLA2 |
| ARPC5L | AGMAT | BOLA2B |
| ARSD | AGPAT1 | BPGM |
| ATP1B1 | AGPAT5 | BRI3 |
| ATP2A2 | AGRN | BRI3BP |
| ATP5F1A | AGTRAP | BSND |
| ATP5F1B | AHNAK | C12orf49 |
| ATP5IF1 | AHSA1 | C12orf57 |
| ATP5MC1 | AIFM1 | C16orf89 |
| ATP5MC3 | AIG1 | C1orf115 |
| ATP5MG | AIMP1 | C1orf56 |
| ATP5PF | AK3 | C21orf62 |
| ATP5PO | AKAP12 | C5orf24 |
| ATP6V0C | AKAP9 | C6orf226 |
| ATP6V0D1 | AKR7A2 | CA2 |
| ATP6V0E2 | AKR7A3 | CCDC71L |

| | | |
|---|---|---|
| ATP6V1F | ALDH1A1 | CCDC8 |
| ATP6V1G1 | ALDH8A1 | CCDC90B |
| ATPAF1 | ALG5 | CCNDBP1 |
| ATXN7L3B | ALPK2 | CDH1 |
| AURKAIP1 | AMACR | CDK16 |
| B4GALT1 | AMT | CDK2AP1 |
| BAG1 | ANAPC11 | CENPB |
| BANF1 | ANAPC13 | CGN |
| BEX2 | ANAPC16 | CHCHD7 |
| BEX3 | ANAPC5 | CHD3 |
| BEX4 | ANPEP | CIB1 |
| BHLHE40 | ANTKMT | CIPC |
| BLCAP | ANXA2 | CISD3 |
| BLOC1S6 | ANXA5 | CLDN16 |
| BOLA3 | ANXA6 | CLN8 |
| BORCS7 | ANXA7 | CMC4 |
| BRD2 | AP1G1 | CMTM4 |
| BRK1 | AP1S1 | COASY |
| BSG | AP2A1 | COL6A1 |
| C14orf119 | AP2B1 | COLCA1 |
| C19orf53 | AP2M1 | COX17 |
| C1orf122 | AP2S1 | CPT1A |
| C1orf43 | AP3S2 | CPTP |
| C4orf3 | APEX1 | CRIP2 |
| C9orf16 | APIP | CRK |
| CA12 | APLP2 | CS |
| CALM1 | APOC2 | CTDNEP1 |
| CALM3 | APOM | DAB2IP |
| CALR | APP | DAG1 |
| CAMK2N1 | AQP1 | DAZAP2 |
| CANX | ARF4 | DBN1 |
| CAP1 | ARF5 | DCAF7 |
| CAPN1 | ARG2 | DCTN1 |
| CAPN2 | ARGLU1 | DDR1 |
| CAPNS1 | ARHGAP1 | DDX3X |
| CAT | ARHGAP24 | DESI1 |
| CBX5 | ARHGAP29 | DIPK1B |
| CBX6 | ARHGEF10L | DNAJB12 |
| CCDC47 | ARL2 | DNAJC10 |
| CCND1 | ARL3 | DUSP3 |
| CCNI | ARL5A | DUSP9 |
| CCT5 | ARL6IP1 | DVL1 |
| CD151 | ARL6IP5 | DYNLL2 |
| CD24 | ARMC10 | EBLN3P |
| CD74 | ARMT1 | EFCAB14 |
| CD81 | ARPC1A | EFHD1 |
| CDC26 | ARPC1B | EIF3A |
| CDC42 | ARPC2 | EIF3B |
| CDH16 | ARPC3 | EIF4G1 |
| CDV3 | ARPC4 | ELAVL1 |

| | | |
|---|---|---|
| CEBPD | ARRDC3 | EMX2 |
| CERS2 | ASAH1 | ENDOG |
| CETN2 | ASB8 | ERG28 |
| CHCHD1 | ASB9 | ERLEC1 |
| CHCHD10 | ASPH | ESRRA |
| CHCHD2 | ATG101 | EVA1B |
| CHD4 | ATN1 | EXOSC6 |
| CHMP1B | ATP1A1 | F11R |
| CHMP2B | ATP2B1 | FAM199X |
| CHP1 | ATP2C1 | FBXO2 |
| CIAO2B | ATP5MC2 | FECH |
| CITED2 | ATP5MF | FGL2 |
| CLDN7 | ATP5PD | FLII |
| CLINT1 | ATP6AP1 | FLOT2 |
| CLPTM1 | ATP6AP2 | FOXC1 |
| CLPTM1L | ATP6V0A1 | FUCA1 |
| CLSTN1 | ATP6V0B | FUNDC1 |
| CLTB | ATP6V0E1 | GAL3ST1 |
| CMBL | ATP6V1D | GATM |
| CMPK1 | ATP6V1E1 | GBA2 |
| CMTM6 | ATP6V1H | GHITM |
| CNBP | ATRAID | GID8 |
| CNDP2 | AUP1 | GLTP |
| CNP | B2M | GMCL1 |
| COA3 | BAD | GNS |
| COA4 | BAIAP2 | GOLGA2 |
| COA6 | BAX | GOLM1 |
| COA8 | BBIP1 | GPS1 |
| COMMD8 | BBOX1 | GPX3 |
| COMMD9 | BCAM | GRPEL1 |
| COPRS | BCAP31 | GRSF1 |
| COPS7A | BCAR1 | GSE1 |
| COPS9 | BCAS2 | GSN |
| COQ9 | BCKDK | H2AFZ |
| COX14 | BCL7A | HINT3 |
| COX5A | BCL7C | HIPK2 |
| COX5B | BHMT | HK1 |
| COX6B1 | BHMT2 | HNRNPF |
| COX6C | BIN1 | HNRNPH2 |
| COX7A2 | BIVM | HNRNPK |
| COX7A2L | BLOC1S2 | HOXB-AS3 |
| COX7B | BLOC1S5 | HOXB8 |
| COX7C | BNIP3 | HOXD8 |
| CSDE1 | BNIP3L | HOXD9 |
| CTBP1 | BOD1 | HSF1 |
| CTSB | BOD1L1 | HSP90AA1 |
| CTSD | BPHL | HSPA2 |
| CTTN | BRD7 | HSPA9 |
| CXXC5 | BSCL2 | HSPH1 |
| CYB5R3 | BTF3 | HYAL1 |

| | | |
|---|---|---|
| CYC1 | BTF3L4 | ID2 |
| CYCS | BTG1 | IDH3A |
| CYFIP1 | BUD23 | IER2 |
| CYS1 | BUD31 | IER3 |
| CYSTM1 | BX284668.5 | IFITM2 |
| DBI | BZW1 | IGFBP2 |
| DBP | C11orf1 | INPPL1 |
| DDB1 | C11orf52 | IRF2BPL |
| DDIT3 | C11orf58 | IRX1 |
| DDX18 | C15orf40 | IRX2 |
| DDX5 | C15orf61 | ISOC2 |
| DNAJA2 | C17orf49 | JADE1 |
| DNAJC21 | C18orf32 | KAT5 |
| DNAJC8 | C19orf25 | KCNJ1 |
| DNPH1 | C1D | KCNJ10 |
| DSP | C1orf210 | KIF1C |
| DUS1L | C1QBP | KIF3B |
| DUSP15 | C2orf74 | KLF9 |
| DYNLL1 | C3orf85 | LAMTOR3 |
| DYNLT1 | C5orf15 | LARP1 |
| ECH1 | C7orf50 | LYPLA1 |
| EEF1A1 | C8orf82 | MANF |
| EEF1E1 | C9orf78 | MAPKAPK2 |
| EEF2 | CALD1 | MAPKAPK3 |
| EI24 | CALM2 | MAT2A |
| EID1 | CALML3 | MBD3 |
| EIF1 | CALML4 | MFAP1 |
| EIF2AK1 | CAMLG | MFN2 |
| EIF3I | CAMTA1 | MIR503HG |
| EIF4A3 | CAPG | MKKS |
| EIF4G2 | CAPZA1 | MLLT1 |
| EIF4H | CAPZB | MPRIP |
| EIF5 | CASC4 | MRPL54 |
| EIF5A | CBR1 | MRPS12 |
| ELOC | CBWD1 | MRPS18A |
| EMC10 | CBX1 | MRPS33 |
| ENO1 | CBY1 | MXRA7 |
| EPHX2 | CCAR1 | MYH10 |
| ERGIC1 | CCDC107 | MYH9 |
| ETF1 | CCDC115 | MYO1C |
| ETFRF1 | CCDC124 | MZT2A |
| EZR | CCDC167 | NAT8L |
| FAM120AOS | CCDC186 | NDRG2 |
| FAM162A | CCDC198 | NFIC |
| FAM168B | CCDC25 | NIPSNAP3A |
| FAM200B | CCDC50 | NUDT7 |
| FAM234A | CCDC58 | NUDT8 |
| FAM32A | CCNC | NUPR2 |
| FAM3C | CCNG1 | OLIG1 |
| FBXW5 | CCNG2 | PAGR1 |

| | | |
|---|---|---|
| FDX1 | CCNL2 | PANK3 |
| FGD5-AS1 | CCPG1 | PBLD |
| FKBP1A | CCS | PCBP1 |
| FKBP4 | CCT2 | PDGFA |
| FKBP5 | CCT3 | PDLIM4 |
| FLNA | CCT6A | PDPR |
| FMC1 | CD164 | PER3 |
| FTH1 | CD2BP2 | PFKM |
| FTL | CD46 | PGK1 |
| FYTTD1 | CD59 | PGP |
| G3BP2 | CD63 | PHF10 |
| GABARAPL1 | CD9 | PIGS |
| GABARAPL2 | CDC123 | PIGT |
| GANAB | CDC37 | PIP4K2C |
| GAPDH | CDC37L1 | PITPNA |
| GATAD1 | CDC42BPB | PLAU |
| GATD3B | CDH2 | PLEC |
| GCSH | CDH6 | POLR3GL |
| GGT6 | CDHR5 | POMGNT1 |
| GLIS2 | CDIP1 | PPP1CA |
| GLO1 | CDIPT | PRDM16-DT |
| GLRX | CDK10 | PRKACA |
| GLRX5 | CDK2AP2 | PRKX |
| GLYR1 | CDK4 | PROM2 |
| GNA11 | CDK5RAP3 | PRPS2 |
| GNG5 | CDKN2AIPNL | PRR15L |
| GOLGA7 | CEBPZ | PRRC2A |
| GOLIM4 | CENPS | PTP4A2 |
| GPRC5C | CENPX | PTPN11 |
| GPX4 | CES2 | PURA |
| GTF3A | CFAP298 | PURB |
| GTF3C6 | CFAP36 | PVALB |
| GUK1 | CFAP410 | PXN |
| H3F3A | CFI | PYGB |
| H3F3B | CFL1 | RAD23A |
| HCFC1R1 | CFLAR | RHOBTB3 |
| HDDC2 | CHCHD5 | RNF141 |
| HDGF | CHDH | RNPS1 |
| HDLBP | CHID1 | RPARP-AS1 |
| HEBP2 | CHL1 | RPS17 |
| HIGD1A | CHMP2A | RRAGD |
| HINT1 | CHMP4A | RTL8C |
| HIST1H4C | CHMP4B | S100A2 |
| HLA-A | CHMP5 | SAV1 |
| HLA-B | CHPT1 | SEC14L1 |
| HLA-C | CHURC1 | SEC16A |
| HLA-E | CIAO1 | SEPTIN9 |
| HMGB1 | CIAO2A | SERPINA5 |
| HMGN3 | CINP | SFRP1 |
| HNRNPA0 | CIR1 | SH3GLB2 |

| | | |
|---|---|---|
| HNRNPA2B1 | CIRBP | SIGIRR |
| HNRNPA3 | CISD1 | SLC25A33 |
| HNRNPAB | CISD2 | SLC35B1 |
| HNRNPDL | CITED4 | SLC35B2 |
| HOXB2 | CKB | SLC39A13 |
| HOXB7 | CLCN3 | SLC4A7 |
| HPCAL1 | CLDN10 | SMC1A |
| HRAS | CLDN2 | SMDT1 |
| HSBP1 | CLDN3 | SNX10 |
| HSD11B2 | CLDN4 | SOD2 |
| HSD17B12 | CLIC1 | SORT1 |
| HSP90AB1 | CLN5 | SPTBN1 |
| HSPE1 | CLNS1A | SPTY2D1 |
| HTATSF1 | CLRN3 | SQSTM1 |
| IARS | CLTA | SREBF1 |
| ICMT | CLU | SRPRA |
| IDH2 | CMAS | SRPRB |
| IER3IP1 | CMC2 | SSRP1 |
| IER5L | CMYA5 | ST14 |
| ILF2 | CNIH1 | STARD10 |
| ILF3-DT | CNIH4 | STARD8 |
| ISCA1 | CNN3 | STIP1 |
| ISCU | CNPY2 | STK11 |
| ITGB1 | COA1 | SUGP2 |
| ITGB1BP1 | COA5 | SVIP |
| IVD | COL18A1 | SYT13 |
| JUND | COL27A1 | SYVN1 |
| KDELR2 | COL4A1 | SZRD1 |
| KIAA1191 | COL4A2 | TAF1C |
| KIAA1522 | COMMD2 | TBC1D16 |
| KIAA2013 | COMMD3 | TBC1D24 |
| KIF5B | COMMD4 | TEF |
| KLF13 | COMMD6 | TEX261 |
| KLHL36 | COMMD7 | TFEB |
| KRT10 | COMT | THAP7 |
| KRT7 | COPB2 | TMEM109 |
| LAMP1 | COPE | TMEM127 |
| LAMTOR1 | COPS5 | TMEM203 |
| LAMTOR5 | COPZ1 | TMEM213 |
| LAP3 | COQ4 | TMEM30B |
| LDHA | COQ5 | TMEM61 |
| LINC01578 | COTL1 | TMEM8A |
| LMAN1 | COX11 | TMEM9B |
| LMNA | COX20 | TMPRSS2 |
| LRP10 | COX4I1 | TMUB1 |
| LRRFIP1 | COX6A1 | TNIP1 |
| LSM10 | CPD | TNS2 |
| LSM12 | CPE | TOLLIP |
| LSM4 | CPT2 | TOMM40 |
| M6PR | CPVL | TPST2 |

| | | |
|---|---|---|
| MAGEF1 | CREB3 | TRA2B |
| MAL2 | CREG1 | TRABD |
| MAPRE1 | CRIM1 | TRPM4 |
| MAZ | CRIPT | TSC22D4 |
| MCCD1 | CRYAB | TUBB |
| MCUR1 | CRYM | TUBB2A |
| MDH2 | CSNK1A1 | TUBB2B |
| METRNL | CSNK1D | TUSC1 |
| METTL23 | CSNK2A1 | UBE2Q1 |
| METTL5 | CSRP2 | UNG |
| METTL9 | CST3 | VPS4A |
| MGAT4B | CSTB | WNK4 |
| MGLL | CTDSPL | YWHAG |
| MICOS10 | CTNNB1 | ZADH2 |
| MIDN | CTSA | ZBTB38 |
| MIEN1 | CTSC | ZBTB4 |
| MKNK2 | CTSF | ZFPM1 |
| MLEC | CTSH | ZHX1 |
| MLF2 | CTSL | ZMAT3 |
| MMP24OS | CTSZ | ZMIZ2 |
| MORF4L1 | CTXN3 | ZNF467 |
| MORN2 | CUEDC2 | ZNF503 |
| MPC1 | CUTA | ZNF664 |
| MPC2 | CWC15 | ZNF710-AS1 |
| MPDU1 | CXCL14 | |
| MPHOSPH8 | CYB5A | |
| MRFAP1 | CYB5B | |
| MRPL12 | CYB5R1 | |
| MRPL15 | CYBA | |
| MRPL18 | CYHR1 | |
| MRPL20 | CYP4F3 | |
| MRPL33 | CYREN | |
| MRPL34 | CZIB | |
| MRPL41 | DAB2 | |
| MRPL49 | DAD1 | |
| MRPL50 | DALRD3 | |
| MRPL51 | DAO | |
| MRPL57 | DAP3 | |
| MRPS14 | DCAF8 | |
| MRPS16 | DCTD | |
| MRPS18B | DCTN2 | |
| MRPS23 | DCTN3 | |
| MRPS26 | DCTN6 | |
| MRPS35 | DDOST | |
| MRPS36 | DDRGK1 | |
| MRPS7 | DDT | |
| MSN | DDX24 | |
| MXD4 | DDX39B | |
| MYL12A | DDX46 | |
| NACA | DDX6 | |

| | |
|---|---|
| NAP1L4 | DECR1 |
| NARS | DEFB1 |
| NBDY | DENR |
| NCBP2 | DERA |
| NCL | DEXI |
| NCOA4 | DFFA |
| NDUFA12 | DGCR6L |
| NDUFA5 | DGUOK |
| NDUFA6 | DHRS12 |
| NDUFA8 | DHRS4 |
| NDUFAB1 | DHRS4-AS1 |
| NDUFAF3 | DHRS7 |
| NDUFAF4 | DHX29 |
| NDUFAF8 | DHX36 |
| NDUFB10 | DLST |
| NDUFB2 | DMAC1 |
| NDUFB3 | DMKN |
| NDUFB4 | DNAJB9 |
| NDUFB5 | DNAJC1 |
| NDUFB6 | DNAJC12 |
| NDUFB7 | DNAJC15 |
| NDUFB9 | DNAJC19 |
| NDUFC1 | DNAJC3 |
| NDUFS1 | DNAJC30 |
| NDUFS5 | DNAJC4 |
| NDUFS6 | DNAJC7 |
| NFE2L1 | DNALI1 |
| NFKBIA | DNMT3L |
| NGRN | DOCK8-AS1 |
| NME1 | DPCD |
| NME4 | DPEP1 |
| NOP10 | DPM2 |
| NORAD | DPM3 |
| NR1H2 | DPP4 |
| NR2F6 | DPP7 |
| NTPCR | DPY30 |
| NUCB1 | DR1 |
| NUCKS1 | DRAP1 |
| NUDCD2 | DSG2 |
| NUDT3 | DSTN |
| NUDT4 | DTD1 |
| OAZ1 | DUT |
| OCIAD2 | DYNC1H1 |
| ODC1 | DYNC1I2 |
| OGDHL | DYNC2LI1 |
| OIP5-AS1 | DYNLT3 |
| OS9 | EAPP |
| OST4 | EBAG9 |
| OTUD6B-AS1 | EBPL |
| P4HB | ECHDC3 |

| | |
|---|---|
| PAQR7 | ECHS1 |
| PARK7 | ECI1 |
| PCK1 | ECI2 |
| PCYOX1 | EDF1 |
| PDCD5 | EEF1B2 |
| PDHB | EEF1G |
| PDIA4 | EHHADH |
| PDIA6 | EIF1AX |
| PEBP1 | EIF1B |
| PFDN2 | EIF2AK2 |
| PFKL | EIF2S1 |
| PFN1 | EIF2S3 |
| PFN2 | EIF3D |
| PGAM1 | EIF3H |
| PGD | EIF3K |
| PGRMC1 | EIF3L |
| PHC2 | EIF3M |
| PIGR | EIF4A1 |
| PIK3R1 | EIF5B |
| PIM3 | ELF3 |
| PINK1 | ELOB |
| PKM | ELP5 |
| PLEKHA3 | EMC3 |
| PLEKHB2 | EMC4 |
| PLEKHJ1 | EMC6 |
| PNN | EMC7 |
| PNPO | EMX2OS |
| POLDIP2 | ENPP2 |
| POLR2C | ENTPD5 |
| POMP | EPCAM |
| POP5 | EPHX1 |
| POU3F3 | EPN1 |
| PPCS | EPRS |
| PPIC | EPS8L2 |
| PPIF | ERBB2 |
| PPM1G | ERBB3 |
| PPP1R14B | ERGIC2 |
| PPP1R1A | ERGIC3 |
| PPP2R1A | ERH |
| PPP2R2D | ERI3 |
| PRDX3 | ERP29 |
| PRDX5 | ERP44 |
| PRDX6 | ESF1 |
| PRKAR1A | ETNK2 |
| PRR13 | EXOC7 |
| PRSS8 | FAAP20 |
| PSMB8 | FABP1 |
| PSMD12 | FAHD1 |
| PSMG3 | FAM104B |
| PTH1R | FAM107B |

| | |
|---|---|
| PTMA | FAM136A |
| PTMS | FAM174C |
| PTPRF | FAM177A1 |
| PTTG1IP | FAM210B |
| PYURF | FAM229B |
| RAB11B | FAM3A |
| RAB13 | FAM92A |
| RAB18 | FASTK |
| RAB1B | FAT1 |
| RAB21 | FAU |
| RAB5C | FBP1 |
| RAB5IF | FBXO17 |
| RABL6 | FBXO22 |
| RAC1 | FCGRT |
| RBIS | FDX2 |
| RBM3 | FERMT2 |
| RBX1 | FGFR3 |
| RDH11 | FH |
| REPIN1 | FIBP |
| RETREG2 | FIS1 |
| RHBDD2 | FKBP3 |
| RHOQ | FKBP8 |
| RNF187 | FLRT3 |
| RNF7 | FLYWCH2 |
| RPL14 | FNBP1L |
| RPL36AL | FNTA |
| RPL4 | FOLR1 |
| RPL7L1 | FUCA2 |
| RPLP0 | FUNDC2 |
| RPN1 | FUOM |
| RPS10 | FUS |
| RPS13 | FYN |
| RPS20 | FZD1 |
| RPS27L | G0S2 |
| RRBP1 | GAA |
| RSL1D1 | GADD45A |
| RSRP1 | GALE |
| RWDD1 | GALK1 |
| S100A11 | GALNT11 |
| S100A6 | GATAD2A |
| SAP18 | GATD3A |
| SARAF | GCA |
| SAT1 | GCC2 |
| SBDS | GCLM |
| SCARB2 | GET1 |
| SCP2 | GET3 |
| SDC1 | GGCT |
| SDF2L1 | GGNBP2 |
| SDF4 | GINM1 |
| SDHA | GIPC1 |

| | |
|---|---|
| SDHC | GLB1 |
| SDHD | GLG1 |
| SEC61A1 | GLOD4 |
| SEC61B | GLRX3 |
| SEC62 | GLUD1 |
| SEL1L | GLUL |
| SELENOF | GMDS |
| SELENOH | GMPR2 |
| SELENOT | GNAI2 |
| SELENOW | GNAS |
| SEPHS2 | GNB1 |
| SERBP1 | GNB2 |
| SERF2 | GNG10 |
| SERINC2 | GNG11 |
| SERP1 | GNG12 |
| SET | GNPDA1 |
| SGCB | GNPTG |
| SGTA | GOLGB1 |
| SH3BGRL3 | GOSR1 |
| SLC25A1 | GOSR2 |
| SLC25A11 | GOT1 |
| SLC25A39 | GPAA1 |
| SLC25A4 | GPANK1 |
| SLC25A5 | GPC4 |
| SLC2A4RG | GPD1 |
| SLC48A1 | GPI |
| SLC6A8 | GPN3 |
| SLIRP | GPR108 |
| SMARCA4 | GPT2 |
| SMCO4 | GRB2 |
| SMIM15 | GRN |
| SMIM7 | GSK3A |
| SNRPD1 | GSR |
| SNRPE | GSTA2 |
| SNRPF | GSTK1 |
| SNRPG | GSTM4 |
| SNRPN | GSTP1 |
| SNX17 | GSTZ1 |
| SNX3 | GTF2A2 |
| SON | GTF2B |
| SPCS1 | GTF2H5 |
| SPG21 | GTPBP6 |
| SPG7 | GUCD1 |
| SPINT1 | H1FX |
| SPINT1-AS1 | H2AFJ |
| SPINT2 | H2AFV |
| SPP1 | H2AFY |
| SPTSSA | HABP4 |
| SRA1 | HACD3 |
| SRP14 | HADHA |

| | |
|---|---|
| SRP9 | HADHB |
| SRRM1 | HAGH |
| SRRM2 | HAX1 |
| SRSF3 | HDDC3 |
| SRSF8 | HDHD2 |
| SRSF9 | HDHD3 |
| SSR3 | HEBP1 |
| SSU72 | HERPUD1 |
| STAT3 | HEXA |
| STOML2 | HEXB |
| STRAP | HIGD2A |
| STUB1 | HINT2 |
| SUB1 | HIP1R |
| SUMO1 | HIST1H1C |
| SUMO2 | HLA-DRA |
| SUMO3 | HLA-DRB1 |
| SURF2 | HM13 |
| SURF4 | HMG20B |
| SUSD6 | HMGB2 |
| SYAP1 | HMGN1 |
| SYNGR2 | HMGN2 |
| SYNJ2BP | HMGN4 |
| TAF10 | HMOX1 |
| TAPBP | HNF4A |
| TCEAL4 | HNRNPA1 |
| TCF25 | HNRNPC |
| TCP1 | HNRNPH1 |
| TFCP2L1 | HNRNPH3 |
| TGOLN2 | HNRNPR |
| THOC7 | HNRNPU |
| TIMM13 | HOMER3 |
| TIMM17A | HOOK1 |
| TLE5 | HOOK2 |
| TM9SF3 | HOXA9 |
| TMA7 | HP1BP3 |
| TMBIM6 | HPD |
| TMED10 | HPF1 |
| TMED4 | HPGD |
| TMED7 | HPN |
| TMEM126B | HSBP1L1 |
| TMEM134 | HSD17B10 |
| TMEM167A | HSD17B4 |
| TMEM230 | HSDL2 |
| TMEM238 | HSP90B1 |
| TMEM251 | HSPA4 |
| TMEM9 | HSPA4L |
| TMSB4X | HSPA5 |
| TMX2 | HSPA8 |
| TNFRSF21 | IAH1 |
| TOB1 | IARS2 |

| | |
|---|---|
| TOMM22 | ID4 |
| TOMM5 | IDI1 |
| TOMM6 | IDS |
| TPI1 | IFI27L2 |
| TPM3 | IFITM3 |
| TRAM1 | IFNAR1 |
| TRIM8 | IFNGR2 |
| TRMT10C | IFRD2 |
| TSC22D3 | IFT20 |
| TSN | IFT22 |
| TSPAN3 | IFT43 |
| TSPAN33 | IFT57 |
| TSPAN6 | IGBP1 |
| TSPYL1 | IGF2R |
| TSR2 | IGFBP7 |
| TUBA1B | IK |
| TUBA1C | IL6ST |
| TUBB4B | ILF3 |
| TXN | ILVBL |
| TXN2 | IMMP1L |
| TXNDC17 | IMMT |
| TXNL4A | IMP4 |
| UBE2I | IMPA2 |
| UBE2M | IMPACT |
| UBE2N | INO80C |
| UBE2V1 | INTS10 |
| UBE2V2 | IP6K2 |
| UBTF | IQGAP1 |
| UQCC2 | ISCA2 |
| UQCR11 | ISOC1 |
| UQCRB | ITGA1 |
| UQCRC1 | ITGAE |
| UQCRFS1 | ITGB8 |
| UQCRH | ITM2B |
| UQCRQ | ITM2C |
| UROS | JAGN1 |
| USF2 | JMJD8 |
| USP2 | JOSD2 |
| USP22 | JPT1 |
| VAMP2 | JPX |
| VAMP8 | JTB |
| VCP | JUNB |
| VDAC1 | KCMF1 |
| VDAC2 | KCNJ16 |
| VEGFB | KCNK5 |
| VPS35 | KDELR1 |
| WASF2 | KHK |
| WDR1 | KHSRP |
| WSB1 | KIAA0232 |
| WSB2 | KIF9 |

| | |
|---|---|
| XBP1 | KLF10 |
| XRCC6 | KLHDC2 |
| YBX1 | KMT2A |
| YWHAB | KMT5B |
| YWHAE | KPNA6 |
| YWHAH | KRR1 |
| ZBTB7A | KRT18 |
| ZCRB1 | KRT19 |
| ZFAND5 | KRT8 |
| ZFP36L2 | KRTCAP2 |
| ZMAT2 | KTN1 |
| ZNF106 | LACTB2 |
| ZNF32 | LAMA5 |
| ZNF580 | LAMB1 |
| ZNF667-AS1 | LAMTOR2 |
| ZNF706 | LAPTM4A |
| ZNHIT3 | LAPTM4B |
| | LBR |
| | LCMT1 |
| | LDHB |
| | LEPROT |
| | LEPROTL1 |
| | LETM1 |
| | LGALS3 |
| | LGALS3BP |
| | LGMN |
| | LIFR |
| | LIMS1 |
| | LINC00665 |
| | LINC00667 |
| | LINC00671 |
| | LINC01697 |
| | LITAF |
| | LMAN2 |
| | LMO7 |
| | LRP2 |
| | LRPAP1 |
| | LRRC19 |
| | LSM1 |
| | LSM14A |
| | LSM2 |
| | LSM3 |
| | LSM5 |
| | LSM6 |
| | LSM7 |
| | LTBP3 |
| | LTBR |
| | LY6E |
| | LYRM1 |
| | LYRM2 |

LZIC
MACC1
MAEA
MAGED2
MAGI2-AS3
MAGOH
MAGT1
MAL
MALAT1
MALSU1
MANBAL
MAOA
MAP1LC3A
MAP2K2
MAP2K3
MAP3K11
MAPK1IP1L
MAPK6
MARC2
MARCH5
MARCH7
MARCKS
MBD2
MBTPS1
MCAT
MCEE
MCFD2
MCL1
MCRIP1
MCTS1
MDH1
MDK
MDM2
MDM4
MEA1
MEAF6
MED10
MED4
MED8
MESD
MET
METRN
MFF
MFSD10
MGST1
MGST3
MIA3
MICOS13
MIGA1
MIR4458HG

MKRN1
MMAB
MMUT
MORF4L2
MPG
MPV17
MRPL11
MRPL13
MRPL14
MRPL16
MRPL17
MRPL19
MRPL22
MRPL28
MRPL3
MRPL32
MRPL36
MRPL4
MRPL40
MRPL42
MRPL43
MRPL44
MRPL47
MRPL52
MRPL9
MRPS10
MRPS11
MRPS15
MRPS17
MRPS18C
MRPS21
MRPS22
MRPS24
MRPS25
MRPS28
MRPS6
MSMO1
MSRA
MSRB1
MSRB2
MST1
MVB12A
MYDGF
MYL12B
MYL6
MYL6B
MYOM3
MZT2B
N4BP2L2
NAA20

NAA38
NAPA
NASP
NAT8
NBN
NCBP2AS2
NCOR1
NCSTN
NDFIP1
NDUFA1
NDUFA13
NDUFA2
NDUFA4
NDUFA7
NDUFA9
NDUFB8
NDUFC2
NDUFS2
NDUFS4
NDUFS7
NDUFS8
NDUFV2
NEDD8
NENF
NEO1
NEU1
NFS1
NFYB
NHP2
NIFK
NINJ1
NIPSNAP1
NIPSNAP2
NIT1
NKAIN4
NME2
NME3
NMRK1
NNT
NOL7
NPC2
NPL
NPM1
NPR3
NQO2
NR1H4
NR2F2
NRBP1
NREP
NSD1

NSL1
NSMCE1
NT5C
NT5C3B
NUB1
NUBP2
NUDC
NUDT16
NUDT16L1
NUDT22
NUDT5
NUDT9
NUTF2
OAF
OARD1
OCIAD1
OGA
OLA1
ORMDL1
OSBP
OSER1
OSTC
P4HA2
PAFAH1B1
PAIP1
PAPLN
PARL
PARP4
PAX2
PAXX
PBDC1
PCBD1
PCMT1
PCMTD2
PCNA
PCSK1N
PDAP1
PDCD2
PDCD4
PDCD6
PDHA1
PDIA3
PDK2
PDZD11
PDZK1
PDZK1IP1
PECR
PEF1
PERP
PEX2

PFDN1
PFDN5
PGLS
PHAX
PHB
PHB2
PHPT1
PHYHD1
PIGP
PIN1
PITHD1
PKIG
PKLR
PKN2
PLA2G12A
PLAAT3
PLAAT4
PLCXD2
PLD3
PLEKHF2
PLG
PLGRKT
PLLP
PLPBP
PLPP3
PLS1
PLXNB1
PLXNB2
PMPCB
PNISR
PNKD
PNPLA4
PNRC2
POLD2
POLR1D
POLR2H
POLR2J
POLR2K
POLR2L
PON2
POP4
POP7
POR
PPA1
PPDPF
PPFIBP1
PPIA
PPIB
PPID
PPIG

PPIL4
PPP1CB
PPP1R11
PPP1R2
PPP1R35
PPP2CA
PPP2R5C
PPP4C
PRAP1
PRCP
PRDX2
PRDX4
PRKAG1
PRKCSH
PRKDC
PRKRA
PRMT1
PROC
PROM1
PROS1
PRPF40A
PRPF6
PRRC2C
PRXL2A
PSAP
PSAT1
PSENEN
PSIP1
PSMA1
PSMA2
PSMA3-AS1
PSMA5
PSMA6
PSMA7
PSMB1
PSMB5
PSMC1
PSMC2
PSMD1
PSMD11
PSMD13
PSMD14
PSMD2
PSMD6
PSMD7
PSMD8
PSMD9
PSME2
PSMF1
PSMG1

PSMG2
PTCD3
PTEN
PTGES2
PTGES3
PTGR1
PTOV1
PTPMT1
PTPRA
PTRH1
PTRHD1
PTS
QDPR
QTRT1
R3HCC1
RAB10
RAB11FIP5
RAB14
RAB2A
RAB34
RAB4A
RAB8A
RABAC1
RACK1
RAD50
RALBP1
RAN
RANBP1
RAP1B
RARRES2
RASSF4
RASSF7
RBBP7
RBCK1
RBM25
RBM39
RBM4
RBMX
RBP4
RBP5
RCC1L
RCN2
RDH10
RDH14
RDH5
REEP5
REEP6
RER1
RETREG1
REXO2

RFC1
RFK
RFXANK
RGN
RHEB
RHOA
RILPL2
RIOK3
RMDN1
RNASE4
RNASEK
RNF10
RNF114
RNF13
RNF130
RNF14
RNF146
RNF167
RNF181
RNF213
RO60
ROMO1
RPAIN
RPL10
RPL10A
RPL11
RPL15
RPL17
RPL18
RPL19
RPL22
RPL23
RPL23A
RPL24
RPL26
RPL26L1
RPL27
RPL27A
RPL28
RPL29
RPL3
RPL30
RPL31
RPL34
RPL35A
RPL36A
RPL37
RPL37A
RPL41
RPL5

RPL6
RPL7A
RPL8
RPL9
RPN2
RPP21
RPP25L
RPS12
RPS14
RPS15A
RPS18
RPS19
RPS2
RPS21
RPS23
RPS25
RPS27A
RPS29
RPS3
RPS3A
RPS4X
RPS4Y1
RPS5
RPS6
RPS7
RPS8
RPS9
RPSA
RRAGA
RREB1
RSRC2
RTCA
RTCB
RTF2
RTL8A
RTN4
RTRAF
RUFY1
RUVBL1
RWDD4
RXRA
S100A1
S100A10
S100A13
S100A16
SAR1A
SARNP
SARS
SCAF11
SCAMP2

SCCPDH
SCD5
SCO1
SCOC
SDC2
SDC4
SDCBP
SDF2
SDHAF1
SDHAF3
SDR39U1
SDSL
SEC11A
SEC11C
SEC13
SEC22B
SEC23B
SEC31A
SEC61G
SEC63
SELENOK
SELENOM
SELENOS
SEM1
SEPHS1
SEPTIN11
SERINC1
SERPINB1
SERPINE2
SERPINF2
SESN2
SESN3
SF1
SF3B2
SF3B4
SF3B6
SFT2D1
SFT2D2
SFXN1
SFXN4
SGK1
SH3BGRL
SH3BP2
SH3YL1
SHISA5
SIKE1
SIL1
SIVA1
SKIL
SKP1

SLBP
SLC13A2
SLC16A9
SLC22A12
SLC22A18AS
SLC22A2
SLC22A5
SLC25A10
SLC25A23
SLC25A3
SLC25A42
SLC27A2
SLC30A9
SLC35D2
SLC35F5
SLC35F6
SLC37A4
SLC39A1
SLC39A4
SLC3A1
SLC3A2
SLC40A1
SLC43A2
SLC44A1
SLC44A4
SLC47A2
SLC51B
SLC5A2
SLC66A2
SLC6A13
SMARCA5
SMARCB1
SMARCE1
SMC3
SMIM10L1
SMIM12
SMIM19
SMIM20
SMIM24
SMIM27
SMIM30
SMIM32
SMS
SNAPC5
SNF8
SNHG29
SNHG32
SNHG6
SNHG8
SNRNP200

SNRNP25
SNRNP35
SNRPA1
SNRPD3
SNU13
SNX1
SNX2
SNX6
SNX7
SNX9
SOD1
SOD3
SOX4
SPAG16
SPAG7
SPART
SPATA20
SPATS2L
SPCS2
SPCS3
SPRYD4
SPRYD7
SPX
SREK1
SREK1IP1
SRI
SRM
SRP19
SRP54
SRP72
SRSF10
SRSF2
SRSF5
SRSF6
SRSF7
SS18L2
SSB
SSBP1
SSNA1
SSR1
SSR2
SSR4
SSX2IP
STAT1
STK19
STK25
STMP1
STOM
STT3B
STX16

STX8
SUCLG1
SUCLG2
SUDS3
SULT1C2
SUMF2
SUOX
SUPT16H
SUPT4H1
SURF1
SVBP
SWI5
SYNE2
SYNRG
SYPL1
TACO1
TACSTD2
TAF1D
TAF7
TAF9
TALDO1
TATDN1
TAX1BP3
TBC1D1
TBCA
TBCB
TBX2
TCEA1
TCEA2
TCEAL8
TCN2
TCTA
TCTN1
TECR
TERF2IP
TEX264
TFAM
TFG
TFPI
TGIF1
THAP5
THNSL2
THRAP3
THUMPD1
THUMPD3-AS1
THYN1
TIMM17B
TIMM8B
TIMMDC1
TIMP1

TINAGL1
TIPRL
TK2
TKT
TLK1
TLN1
TM2D2
TM7SF2
TM7SF3
TM9SF1
TM9SF2
TMBIM4
TMCO1
TMED1
TMED2
TMED5
TMED9
TMEM106B
TMEM106C
TMEM123
TMEM126A
TMEM139
TMEM141
TMEM147
TMEM14A
TMEM14B
TMEM14C
TMEM150A
TMEM165
TMEM176A
TMEM176B
TMEM179B
TMEM18
TMEM183A
TMEM192
TMEM205
TMEM208
TMEM219
TMEM243
TMEM248
TMEM258
TMEM259
TMEM263
TMEM30A
TMEM33
TMEM35B
TMEM38B
TMEM50A
TMEM50B
TMEM59

TMEM70
TMEM98
TMPO
TMSB10
TMX4
TNFRSF11B
TNFRSF19
TNFRSF1A
TNFSF10
TOB2
TOMM20
TOMM7
TOP2B
TOR1AIP2
TOX4
TP53I13
TP53I3
TP53TG1
TPCN1
TPD52L2
TPM1
TPMT
TPR
TPRG1L
TRAPPC1
TRAPPC3
TRAPPC4
TRAPPC5
TRAPPC6A
TRIOBP
TRIP11
TRIP6
TRIR
TRMT112
TRPT1
TSC22D1
TSEN34
TSFM
TSNAX
TSPAN1
TSPAN4
TSPO
TSR3
TSSC4
TSTA3
TSTD1
TTC1
TTC19
TTC3
TUBG1

TUFM
TUG1
TUSC3
TWF1
TXNDC5
TXNDC9
TXNIP
TXNL1
TYW3
UBAC2
UBB
UBE2A
UBE2B
UBE2D2
UBE2E3
UBE2H
UBE2R2
UBE2Z
UBL5
UBXN1
UBXN4
UCHL3
UFC1
UFD1
UFL1
UFSP2
UGT3A1
UNC50
UPB1
UPF3A
UPK3BL1
UQCC3
UQCR10
UQCRC2
URI1
URM1
USP14
USP16
VAMP3
VAMP5
VAPB
VAT1
VBP1
VCAM1
VDAC3
VEGFA
VKORC1
VMP1
VPS25
VPS29

VPS36
VPS4B
WASHC3
WASL
WBP1
WBP11
WBP2
WDR45
WDR45B
WDR83OS
WFDC2
WIPI1
WLS
WTAP
XIST
XPC
XPO1
XRCC5
XRN2
YBX3
YDJC
YIF1A
YIPF3
YIPF4
YWHAQ
YY1
ZBED5-AS1
ZBTB16
ZBTB8OS
ZC3H15
ZDHHC12
ZDHHC2
ZDHHC4
ZFAS1
ZFP36L1
ZFP91
ZFYVE21
ZKSCAN1
ZNF24
ZNF330
ZNF428
ZNF91
ZNRD1
ZNRF2
ZRANB2
ZSWIM7

**Supplementary Table S5b. All enhanced genes in the PT only, TAL only, and shared to identify overlap**

| Common | Only PT | Only TAL |
|---|---|---|
| ABL1 | ABCC4 | AASDH |
| AC118549.1 | ABLIM1 | ABCA5 |
| ADAMTS9-AS1 | AC026803.3 | ABCC5 |
| AGAP1 | AC137056.1 | ABHD17C |
| AKAP13 | ACAT1 | ABI1 |
| ANK2 | ACLY | AC008014.1 |
| ANK3 | ACSM2A | AC016705.2 |
| ANKHD1 | ACSM2B | AC018730.1 |
| ANKIB1 | ACY1 | AC019197.1 |
| ANKRD17 | ACY3 | AC068631.1 |
| AOPEP | AFM | AC092078.2 |
| ARFGEF1 | AFP | AC138305.1 |
| ARHGEF28 | AK4 | ACACA |
| ARID1B | AKR1C1 | ACAP2 |
| ARID5B | AL138826.1 | ACBD6 |
| ATE1 | ALB | ACIN1 |
| ATF7IP | ALDH1L1 | ACOT11 |
| AUTS2 | ALDH6A1 | ACPP |
| BCAS3 | ARFGEF2 | ACSS3 |
| BCKDHB | ARHGAP42 | ACYP2 |
| BCL2 | ARHGEF12 | ADAM10 |
| BICC1 | ASPA | ADAMTS9-AS2 |
| BICDL1 | ATP5ME | ADGRA3 |
| BIRC6 | ATXN7L1 | ADGRF1 |
| C11orf54 | AVP | ADGRL2 |
| CDC42BPA | BNC2 | ADGRV1 |
| CDK14 | BRPF3 | ADHFE1 |
| CFDP1 | CA2 | ADIRF |
| CHD9 | CAMK2D | ADK |
| CNKSR3 | CAPN3 | AEBP2 |
| CPEB4 | CARHSP1 | AF117829.1 |
| CPM | CCNY | AFF1 |
| CREBRF | CERS4 | AFTPH |
| DAPK1 | CGNL1 | AGO3 |
| DCDC2 | CLIC4 | AGPAT4 |
| DCXR | CLMN | AHI1 |
| DENND1A | CLTRN | AIG1 |
| DIP2C | COMTD1 | AK3 |
| DLG1 | CREB5 | AKAP10 |
| EHBP1 | DAZAP2 | AKAP11 |
| ELF1 | DHDH | AKAP6 |
| EPB41L4A | DYNLL2 | AKAP9 |
| EPB41L5 | DYNLRB1 | AKT3 |

| | | |
|---|---|---|
| ERBIN | EGLN1 | AL158152.1 |
| ESRRG | ENPEP | AL159156.1 |
| EXOC4 | EPHA7 | ALDH1A2 |
| FAF1 | ERRFI1 | AMMECR1 |
| FAM13A | FGGY | ANAPC10 |
| FAM172A | FNIP2 | ANGPTL1 |
| FBXO11 | FRMD3 | ANKRD10 |
| FGD4 | FTCD | ANKRD26 |
| FHIT | FUT6 | ANKRD28 |
| FKBP2 | G6PC | ANKS1A |
| FNDC3B | GCDH | ANO10 |
| FOXO3 | GGACT | AP3B1 |
| FOXP1 | GIPC2 | APBB2 |
| FRAS1 | GK | APC |
| FREM2 | GLS | APOOL |
| FTO | GLTPD2 | APPBP2 |
| FTX | GLYAT | AQR |
| GAREM1 | GOLPH3 | ARF5 |
| GBE1 | GPT | ARFGEF3 |
| GLIS3 | GRHPR | ARFIP1 |
| GNAQ | GSN | ARHGAP12 |
| GPC6 | HIPK3 | ARHGAP18 |
| GPHN | HNF4G | ARHGAP21 |
| HBP1 | HNMT | ARHGAP24 |
| HERC1 | HOGA1 | ARHGAP29 |
| HERC4 | IVNS1ABP | ARHGAP5 |
| HNRNPM | KCNJ15 | ARHGAP6 |
| HSPB1 | KIF1B | ARHGAP8 |
| IGF1R | KL | ARHGEF38 |
| IMMP2L | KLF6 | ARHGEF7 |
| INSR | KMO | ARID2 |
| ITPR2 | LIME1 | ARID4A |
| JMJD1C | LINC01874 | ARID4B |
| KANK1 | LINC02027 | ARIH1 |
| KANSL1 | LINC02294 | ARL15 |
| KANSL1L | LRMDA | ARMC8 |
| KAZN | LRRK2 | ARMCX4 |
| KIAA1109 | MACROD1 | ARMH4 |
| KIAA1217 | MAF | ARNT2 |
| KIAA1958 | MAOB | ARRDC3 |
| KIFC3 | MAPK1 | ASAP2 |
| KMT2C | MARF1 | ASB3 |
| LCOR | MED31 | ASCC3 |
| LINC00472 | METTL7A | ASH1L |
| LPP | MIOX | ASXL1 |
| LRBA | MPST | ASXL3 |
| LRRC8D | MRLN | ATAD1 |

| | | |
|---|---|---|
| LYPLAL1 | MYO15B | ATAD2B |
| MAGI1 | NAPRT | ATF6 |
| MAML2 | NDUFB1 | ATF7IP2 |
| MAN1A1 | NOX4 | ATG10 |
| MAP3K1 | NR2F1 | ATG2B |
| MAPK10 | OSBPL8 | ATG5 |
| MAST4 | OXT | ATM |
| MBD5 | PAH | ATP10D |
| MBNL1 | PCBP1 | ATP11B |
| MBNL2 | PHGDH | ATP2C1 |
| MED13L | PPP1R16A | ATP6V1H |
| MFSD4B | PPP1R16B | ATP8A1 |
| MIR99AHG | PPP1R21 | ATP9B |
| MITF | PRKCA | ATR |
| MKLN1 | PRLR | ATRN |
| MLLT10 | PRODH2 | ATRX |
| MSH3 | PRUNE2 | ATXN1 |
| MYO9A | PTBP3 | ATXN10 |
| NAPEPLD | PTPRD | ATXN2 |
| NBEAL1 | QARS | ATXN3 |
| NCOA2 | RAB11FIP3 | AUH |
| NCOA7 | RENBP | AVL9 |
| NEDD4L | RERG | AZIN1-AS1 |
| NF1 | RGL1 | BABAM2 |
| NFAT5 | RHOBTB1 | BACE2 |
| NFIA | RNF152 | BACH1 |
| NFIB | RPL13A | BAZ2B |
| NPEPPS | RPS17 | BBS9 |
| NRIP1 | SEMA5A | BCAT2 |
| NSRP1 | SERPINA1 | BLNK |
| NTN4 | SH3D19 | BMP6 |
| NUMB | SIGIRR | BMPR1A |
| OXR1 | SLC16A10 | BMPR2 |
| P3H2 | SLC17A1 | BPTF |
| PARD3 | SLC1A1 | BRAF |
| PATJ | SLC25A30 | BRD8 |
| PBX1 | SLC25A36 | BROX |
| PDE4D | SLC28A1 | BRWD1 |
| PDE8A | SLC2A2 | BTBD11 |
| PDGFC | SLC2A9 | BTBD7 |
| PDLIM5 | SLC34A1 | BTBD9 |
| PDS5A | SLC36A2 | BTF3 |
| PFDN6 | SLC38A2 | BZW2 |
| PHLDB2 | SMAD3 | C11orf49 |
| PIAS1 | SNX30 | C1GALT1C1 |
| PICALM | SPNS2 | C20orf194 |
| PIK3CB | ST13 | C2CD5 |

| | | |
|---|---|---|
| PKD2 | SUGCT | C4orf19 |
| PKHD1 | TBC1D19 | CA10 |
| PKP4 | TGFBR3 | CACNA2D3 |
| PLCL1 | THY1 | CACNB2 |
| PLEKHA5 | TM4SF5 | CACNB4 |
| PLEKHA7 | TMEM245 | CACUL1 |
| PLXDC2 | TNS1 | CADM1 |
| POLR2I | TRPM3 | CADPS2 |
| PPARGC1A | TTC28 | CAMKMT |
| PPM1K | TTC36 | CAPN7 |
| PPP1R9A | TTC38 | CARMIL1 |
| PPP2R3A | UGT2B7 | CASC15 |
| PPP3CA | USE1 | CASD1 |
| PPP6R2 | UTP4 | CASR |
| PRKAA2 | ZCCHC10 | CBFA2T2 |
| PTH2R | ZCCHC14 | CBLB |
| PTK2 | ZFAND6 | CBR4 |
| PTPN13 | ZGPAT | CC2D2A |
| PTPRG | | CCDC12 |
| PTPRK | | CCDC14 |
| PTPRM | | CCDC146 |
| PUM2 | | CCDC148 |
| RABGAP1L | | CCDC171 |
| RALGAPA1 | | CCDC178 |
| RAPGEF2 | | CCDC18-AS1 |
| RBFOX2 | | CCDC198 |
| RBM47 | | CCDC30 |
| RBM6 | | CCDC66 |
| RBMS1 | | CCDC91 |
| RERE | | CCNB1IP1 |
| RORA | | CCNG2 |
| RPRD2 | | CCNH |
| RPS28 | | CCNT2 |
| SBF2 | | CCS |
| SIK2 | | CCSER1 |
| SIPA1L1 | | CCSER2 |
| SLC16A12 | | CD2AP |
| SLC4A4 | | CD47 |
| SLCO4C1 | | CD63 |
| SMAD5 | | CD9 |
| SNX29 | | CDC16 |
| SORBS2 | | CDC73 |
| SOS1 | | CDK12 |
| SOS2 | | CDK13 |
| SOX6 | | CDK6 |
| SPIN1 | | CDKAL1 |
| SPPL3 | | CDKL1 |

| | |
|---|---|
| SSH2 | CEBPZOS |
| STAG1 | CENPC |
| STAU2 | CEP192 |
| STRN3 | CEP290 |
| SUMF1 | CEP350 |
| SYNE1 | CEP70 |
| TAB2 | CEP95 |
| TACC1 | CEPT1 |
| TBC1D4 | CFAP221 |
| TBC1D5 | CFAP70 |
| TCF12 | CFI |
| TEAD1 | CHCHD3 |
| THBS1 | CHD1 |
| TNRC6A | CHD2 |
| TNS3 | CHD6 |
| TRHDE | CHD7 |
| TRPM7 | CHKA |
| TRPS1 | CHL1 |
| TST | CHM |
| UBE2E2 | CHPT1 |
| UBE2W | CIR1 |
| URGCP | CIT |
| USP34 | CKB |
| VWA8 | CLASP1 |
| WDFY3 | CLASP2 |
| WDR72 | CLCN3 |
| WWC1 | CLCN5 |
| WWOX | CLCNKA |
| WWP1 | CLDN10 |
| WWTR1 | CLDN10-AS1 |
| XPR1 | CLDN14 |
| YAP1 | CLDN3 |
| ZBTB20 | CLK1 |
| ZBTB44 | CLK4 |
| ZFAND3 | CLU |
| ZNF280D | CLYBL |
| ZNF385B | CMSS1 |
| ZNF638 | CNNM2 |
| | CNOT2 |
| | CNOT4 |
| | CNOT6L |
| | CNTN1 |
| | COA1 |
| | COA6-AS1 |
| | COBL |
| | COBLL1 |
| | COG5 |

COG6
COL4A1
COL4A2
COL4A3
COL4A4
COL4A5
COMMD1
COMMD10
COP1
COPB1
COPS2
COPS4
COPS6
CPEB2
CPEB3
CPLANE1
CPNE8
CPQ
CPSF6
CPVL
CRADD
CREB1
CREBBP
CRIM1
CRYBG3
CSRNP3
CSRP2
CST3
CSTF3
CTDSPL2
CTNND1
CTSL
CTSO
CUL1
CUL3
CWC22
CWC27
CWF19L2
CYBA
CYP20A1
CYP4V2
CYP7B1
DANT2
DCAF10
DCAF5
DCAF6
DCPS

DCUN1D4
DDX52
DEK
DENND1B
DENND2A
DENND4A
DENND4C
DERA
DHX15
DICER1
DMAC2L
DMGDH
DMTF1
DMXL1
DNAH14
DNAJC1
DNASE1
DNM3
DOCK1
DOP1A
DPH6
DPP7
DPY19L4
DST
DTWD1
DYM
DYNC1I2
DYNC2H1
DYNC2LI1
DYRK1A
DZIP1
DZIP3
ECHDC1
EEA1
EFNA5
EFR3A
EGF
EIF3E
EIF3G
EIF3H
EIF3M
EIF4G3
ELF2
ELP2
ELP4
EMC2
ENOX1

EP300
EPB41L3
EPB41L4A-AS1
EPC1
EPC2
EPCAM
EPHA4
EPS15
ERBB4
ERC1
ERCC6L2
ERICH1
ERMP1
ESD
ESRRB
ETFDH
EVI5
EXOC1
EXOC6B
EXOSC8
EXT1
EZH1
F11
FABP3
FAM120B
FAM133B
FAM135A
FAM13B
FAM160A1
FAM171A1
FAM184A
FAM214A
FAM49B
FAR1
FARP1
FARS2
FASTKD1
FASTKD2
FAXDC2
FBXL17
FBXL4
FBXO34
FBXW11
FCHO2
FDFT1
FER
FGF13

FGF9
FGFR2
FHOD3
FIP1L1
FMO4
FNBP1L
FNBP4
FNDC3A
FNIP1
FNTA
FOCAD
FOXJ3
FOXN3
FOXO1
FREM1
FRK
FRMD4A
FRMD4B
FRS2
FRYL
FUBP1
FZD6
GALK2
GALNT10
GALNT14
GALNT18
GARS-DT
GAS5
GAS6
GATAD2B
GBF1
GCFC2
GCNT2
GHR
GIGYF2
GLCCI1
GMDS
GMDS-DT
GNAI1
GPAT3
GPATCH2L
GPATCH8
GPBP1
GPBP1L1
GPC3
GPC5
GPCPD1

GPNMB
GPRIN3
GRAMD1C
GRAMD2B
GRB14
GRB7
GRHL2
GSAP
GSK3B
GSTA4
GSTO2
GSTP1
GUCY1A1
GULP1
HAT1
HDAC8
HEATR5A
HEATR5B
HECTD4
HELZ
HIBADH
HIBCH
HIP1
HMGCR
HNF1A-AS1
HNF1B
HNRNPC
HOXA-AS2
HOXA3
HOXB3
HOXD3
HPN
HS2ST1
HS6ST2
HSPA12A
HTT
ICE2
ID1
ID3
ID4
IFI30
IFIT1
IFT122
IFT80
IFT88
IGBP1
IGF2BP2

IL6ST
IMMP1L
ING4
INPP4B
INTS6
INTU
IP6K1
IRS1
ITCH
ITFG1
ITFG2
ITGA2
ITGB3BP
ITGB8
ITM2B
ITPR1
ITSN2
JAK1
JPX
KAT2B
KAT6A
KAT6B
KATNAL2
KATNBL1
KCNIP4
KCNJ16
KCNJ3
KCNMB2
KCTD1
KCTD16
KCTD3
KDM2A
KDM3B
KDM4C
KDM6A
KIAA0319L
KIAA0355
KIAA0586
KIAA1328
KIAA2026
KIDINS220
KIF12
KIF16B
KIFAP3
KITLG
KLF12
KLHDC10

KLHDC2
KLHL13
KLHL24
KMT2E
KMT5B
KNG1
KPNA4
KRCC1
KRIT1
KRTCAP2
LAMA1
LAMB1
LAPTM4A
LARP4B
LCORL
LDHB
LGR4
LIFR
LIMCH1
LINC00379
LINC00467
LINC00476
LINC00645
LINC00671
LINC01089
LINC01184
LINC01278
LINC01320
LINC01473
LINC01606
LINC01725
LINC01762
LINC02121
LINC02343
LINC02432
LINC02532
LIPH
LMBR1
LMBRD1
LMO7
LNX1
LPL
LRCH1
LRP1B
LRP6
LRPPRC
LRRFIP2

LSM14A
LTN1
LUC7L2
LYST
MACF1
MACO1
MACROD2
MAGED1
MAGI2
MAGI3
MALAT1
MALRD1
MAN1A2
MAN2A1
MAP2K4
MAP3K2
MAP3K4
MAP3K7
MAP4K3
MAP4K3-DT
MAP4K5
MAP7
MAPK8
MAPK8IP3
MARCH7
MARK3
MBD6
MCCC1
MCF2L
MDN1
ME3
MECOM
MED13
MED21
MED23
MEF2A
MEF2C
METTL15
MFF
MFSD14A
MFSD14C
MGAT5
MGME1
MGMT
MIA2
MIB1
MICU2

MICU3
MIPEP
MIR3936HG
MLLT3
MNAT1
MOB3B
MOK
MON2
MPDZ
MPP7
MPPED2
MROH7
MRPS31
MRRF
MRTFB
MSANTD2
MSI2
MST1
MUC15
MYCBP2
MYEF2
MYLK
MYO10
MYO3B
MYSM1
N4BP2L2
NAA35
NAAA
NAALADL2
NBAS
NCKAP1
NCOA1
NCOA3
NCOR1
NDUFS4
NEBL
NEDD9
NEK1
NEK7
NELL1
NEMF
NFYC
NGLY1
NHS
NHSL1
NINL
NIPBL

NLK
NMD3
NME5
NME7
NOS1AP
NOVA1
NPHP3
NPSR1-AS1
NPY1R
NR2F1-AS1
NR2F2-AS1
NR3C1
NR3C2
NRCAM
NRDC
NRG1
NRK
NSD1
NSMCE2
NSUN6
NT5C2
NT5DC1
NUBPL
NUDT5
NUP153
NUP214
NUP58
NUTM2A-AS1
NUTM2B-AS1
OCIAD1
OCLN
ODF2L
OGA
OLA1
OMA1
OPA1
ORC4
OSBPL10
OSBPL1A
OSBPL3
OSBPL9
PACS1
PAM
PAN3
PANTR1
PAQR5
PARD3B

PAWR
PAXBP1
PBRM1
PCCA
PCCB
PCDH9
PCLO
PCM1
PCMTD1
PCNX1
PCNX4
PDE10A
PDE1A
PDE7B
PDE9A
PDS5B
PDSS2
PELI2
PET100
PHACTR1
PHACTR4
PHC3
PHF14
PHF21A
PHF3
PHIP
PIAS2
PIBF1
PIGF
PIGN
PIK3C3
PIK3CA
PILRB
PITPNB
PKN2
PLCB1
PLEKHA6
PLEKHH2
PLIN5
PLOD2
PLPPR1
PLSCR1
PMM1
POGZ
POLK
POLR2B
POU2F1

PPA2
PPDPF
PPFIA2
PPFIBP1
PPHLN1
PPIP5K2
PPM1A
PPM1L
PPP1R12A
PPP1R13B
PPP2R2B
PPP2R5E
PPP3CB
PPP3R1
PPP4R3B
PPP6R3
PRDM16
PREPL
PRH1
PRICKLE2
PRKACB
PRKCQ
PRKD1
PRKG1
PRKN
PROX1
PRRG1
PSENEN
PSMB1
PSME4
PSMG2
PSPC1
PTAR1
PTBP2
PTEN
PTGER3
PTPN14
PTPN2
PTPN3
PTPN4
PTPRA
PTPRJ
PUM3
PWWP3A
PXDNL
QKI
QTRT1

R3HDM1
RAB28
RAB3GAP1
RAB3GAP2
RAB3IP
RABAC1
RABGAP1
RABGEF1
RACK1
RAD51B
RALGAPA2
RALGAPB
RALGPS1
RALGPS2
RALYL
RANBP3L
RAP1A
RAP1GAP2
RAP1GDS1
RAP2C-AS1
RAPGEF1
RAPGEF6
RARS2
RASA1
RASAL2
RASSF8
RB1
RB1CC1
RBBP4
RBBP8
RBFOX1
RBM26
RBM27
RBM33
RBPMS
RC3H1
RC3H2
RCOR3
REEP3
RELCH
REV1
REV3L
RFX3
RICTOR
RIF1
RIMKLB
RIN2

RING1
RMDN1
RNF111
RNF13
RNF150
RNF19A
RNF38
RNF43
RNMT
RNPC3
ROCK1
ROCK2
RP1
RPAP2
RPL10
RPL10A
RPL18A
RPL34
RPL35A
RPL36
RPL36A
RPL37
RPL37A
RPL38
RPL39
RPLP1
RPRD1A
RPS11
RPS14
RPS2
RPS21
RPS24
RPS26
RPS27
RPS29
RPS6KA6
RPS9
RSL24D1
RSRC1
RUFY3
RYK
S100A10
SAMD12
SARS
SCAF8
SCAPER
SCFD1

SCGB1D2
SCYL2
SDCCAG8
SEC24B
SECISBP2L
SELENOP
SEMA6D
SENP6
SENP7
SETBP1
SETD2
SETD5
SETDB2
SF3B1
SGCE
SGIP1
SGK2
SGK3
SGMS1
SGO1-AS1
SH3RF1
SHANK2
SHLD2
SHPRH
SHROOM3
SIK3
SIL1
SIM1
SIM2
SIPA1L3
SKAP1
SKAP2
SLC12A1
SLC15A2
SLC16A7
SLC20A2
SLC22A2
SLC22A3
SLC25A12
SLC25A13
SLC25A27
SLC2A12
SLC2A13
SLC35A1
SLC39A10
SLC3A1
SLC44A3

SLC5A3
SLMAP
SMAD2
SMAD4
SMAP1
SMARCA2
SMARCAD1
SMARCB1
SMC5
SMCHD1
SMG1
SMG6
SMG7
SMIM8
SMURF2
SMYD3
SNHG25
SNHG7
SNHG8
SNRK
SNRNP70
SNRPD2
SNX13
SNX14
SNX24
SORBS1
SP100
SP3
SPAG16
SPART
SPATA13
SPATA6
SPEF2
SPIDR
SPSB3
SPTLC2
SRGAP3
SRP54
SRPK2
SRSF11
SRSF4
SS18
SSBP2
SSR4
ST3GAL6
STAG2
STARD13

STK3
STK32B
STK39
STON2
STPG2
STRBP
STRN
STX17
STX18
STX7
STX8
STXBP4
SUCLA2
SUCLG2-AS1
SUCNR1
SULT1C2
SUPT20H
SUPT3H
SUSD4
SYNE2
SYNRG
SYPL1
SYTL2
SYTL4
TAF15
TALDO1
TANC2
TANK
TAOK1
TAOK3
TAPT1
TAPT1-AS1
TASOR
TASOR2
TATDN1
TBCK
TBL1X
TBL1XR1
TBRG1
TCAF1
TCAIM
TCEA3
TCERG1
TCF7L2
TDRD3
TDRP
TECR

TENT2
TET2
TEX41
TFCP2
TFDP2
TFPI
TGFBR2
THADA
THOC2
THRB
THSD4
THSD7A
TLK1
TLN2
TMBIM4
TMCC1
TMEM116
TMEM120A
TMEM131
TMEM161B
TMEM161B-AS1
TMEM164
TMEM168
TMEM176A
TMEM176B
TMEM184C
TMEM232
TMEM38B
TMEM59
TMEM65
TMEM72-AS1
TMX3
TNFSF10
TNKS
TNKS2
TNRC6B
TNRC6C
TOGARAM1
TOM1L1
TOM1L2
TOX
TPP2
TPST1
TPT1
TPT1-AS1
TRA2A
TRAK1

TRAK2
TRAPPC12
TRAPPC8
TRAPPC9
TRIM2
TRIM33
TRIO
TRIP12
TRMT11
TRMT9B
TSC22D1
TSC22D2
TSGA10
TSPAN8
TSPAN9
TTC17
TUSC3
TXNIP
TYRP1
U2SURP
UACA
UBA52
UBA6-AS1
UBAC2
UBC
UBE2E1
UBE2E3
UBE2H
UBE2K
UBE3C
UBE4B
UBN2
UBR1
UBR2
UBR3
UBR5
UGGT2
UGT8
UMAD1
UMOD
UNC13B
UPF2
URI1
USO1
USP14
USP15
USP24

USP25
USP3
USP33
USP40
USP47
USP48
UTRN
UVRAG
VAV3
VEPH1
VEZT
VOPP1
VPS13A
VPS13B
VPS13C
VPS13D
VPS39
VPS41
VPS45
VPS50
VPS54
VTI1A
WASHC4
WDFY2
WDR11
WDR27
WDR33
WDR48
WDR7
WDR70
WLS
WNK1
WWC2
WWP2
XIST
XPO1
XRN1
XRRA1
YBEY
YLPM1
YPEL3
YTHDC1
YTHDC2
YTHDF3
ZBED5
ZBED5-AS1
ZBTB1

ZBTB8OS
ZC3H6
ZCCHC7
ZDHHC14
ZDHHC17
ZDHHC21
ZFAS1
ZFR
ZFX
ZHX3
ZMAT1
ZMYM2
ZMYM4
ZMYND11
ZMYND8
ZNF131
ZNF141
ZNF148
ZNF207
ZNF254
ZNF277
ZNF292
ZNF326
ZNF33A
ZNF33B
ZNF385D
ZNF407
ZNF44
ZNF518A
ZNF618
ZNF644
ZNF704
ZNF711
ZNF721
ZNF780B
ZNF827
ZNF83
ZRANB2
ZSWIM6
ZXDC

Supplementary Table S6a. Enhanced pathways using KEGG 2021

| Term | Overlap | P-value | Adjusted P-value | Odds Ratio | Combined Score | Genes |
|---|---|---|---|---|---|---|
| **Ubiquitin mediated proteolysis** | 9/140 | 2.70799588038769E-05 | 0.00357399 | 6.34 | 66.645 | HERC4;UBE2W;MAP3K1;HERC1;UBE2E2;NEDD4L;WWP1;BIRC6;PIAS1 |
| **Growth hormone synthesis, secretion and action** | 8/119 | 5.53931613348499E-05 | 0.00357399 | 6.62 | 64.918 | MAPK10;MAP3K1;GNAQ;ITPR2;PIK3CB;SOS1;SOS2;PTK2 |
| **Neurotrophin signaling pathway** | 8/119 | 5.53931613348499E-05 | 0.00357399 | 6.62 | 64.918 | MAPK10;MAP3K1;ABL1;BCL2;PIK3CB;FOXO3;SOS1;SOS2 |
| **FoxO signaling pathway** | 8/131 | 1.09067726806049E-04 | 0.00357399 | 5.97 | 54.501 | MAPK10;PRKAA2;INSR;PIK3CB;FOXO3;SOS1;SOS2;IGF1R |
| **MAPK signaling pathway** | 12/294 | 1.16099451618994E-04 | 0.00357399 | 3.95 | 35.796 | MAPK10;PPP3CA;MAP3K1;INSR;PDGFC;NF1;RAPGEF2;HSPB1;TAB2;SOS1;SOS2;IGF1R |
| **Tight junction** | 9/169 | 1.1699849659254E-04 | 0.00357399 | 5.18 | 46.904 | MAGI1;MAPK10;PATJ;DLG1;PRKAA2;MAP3K1;PARD3;NEDD4L;RAPGEF2 |
| **Rap1 signaling pathway** | 10/210 | 1.24467142803137E-04 | 0.00357399 | 4.62 | 41.518 | MAGI1;SIPA1L1;PARD3;INSR;GNAQ;PDGFC;RAPGEF2;PIK3CB;THBS1;IGF1R |
| **PI3K-Akt signaling pathway** | 13/354 | 1.73493407616751E-04 | 0.00435902 | 3.55 | 30.701 | MAGI1;PRKAA2;INSR;PIK3CB;PPP2R3A;FOXO3;THBS1;PTK2;IGF1R;PDGFC;BCL2;SOS1;SOS2 |
| **ErbB signaling pathway** | 6/85 | 3.68404985275854E-04 | 0.00712658 | 6.93 | 54.763 | MAPK10;ABL1;PIK3CB;SOS1;SOS2;PTK2 |
| **AMPK signaling pathway** | 7/120 | 3.88809691139241E-04 | 0.00712658 | 5.67 | 44.492 | PRKAA2;INSR;PIK3CB;PPP2R3A;FOXO3;PPARGC1A;IGF1R |
| **Colorectal cancer** | 6/86 | 3.92416522657156E-04 | 0.00712658 | 6.84 | 53.644 | MAPK10;MSH3;BCL2;PIK3CB;SOS1;SOS2 |
| **Focal adhesion** | 9/201 | 4.25467255406281E-04 | 0.00712658 | 4.31 | 33.458 | MAPK10;PDGFC;BCL2;PIK3CB;SOS1;SOS2;THBS1;PTK2;IGF1R |
| **Proteoglycans in cancer** | 9/205 | 4.90836573134632E-04 | 0.00758909 | 4.22 | 32.165 | ITPR2;ANK2;ANK3;PIK3CB;SOS1;SOS2;THBS1;PTK2;IGF1R |
| **GnRH signaling pathway** | 6/93 | 5.96643713532902E-04 | 0.0085661 | 6.29 | 46.676 | MAPK10;MAP3K1;GNAQ;ITPR2;SOS1;SOS2 |
| **Prostate cancer** | 6/97 | 7.45649269257899E-04 | 0.0099917 | 6.01 | 43.276 | PDGFC;BCL2;PIK3CB;SOS1;SOS2;IGF1R |
| **Estrogen signaling pathway** | 7/137 | 8.58091299498443E-04 | 0.01014567 | 4.92 | 34.745 | NCOA2;GNAQ;BCL2;ITPR2;PIK3CB;SOS1;SOS2 |
| **Insulin signaling pathway** | 7/137 | 8.58091299498443E-04 | 0.01014567 | 4.92 | 34.745 | MAPK10;PRKAA2;INSR;PIK3CB;SOS1;PPARGC1A;SOS2 |
| **Longevity regulating pathway** | 6/102 | 9.70606413426139E-04 | 0.01042225 | 5.70 | 39.510 | PRKAA2;INSR;PIK3CB;FOXO3;PPARGC1A;IGF1R |
| **T cell receptor signaling pathway** | 6/104 | 0.001073913 | 0.01042225 | 5.58 | 38.135 | MAPK10;PPP3CA;DLG1;PIK3CB;SOS1;SOS2 |

| | | | | | | |
|---|---|---|---|---|---|---|
| **Prolactin signaling pathway** | 5/70 | 0.00108504 | 0.01042225 | 6.99 | 47.701 | MAPK10;PIK3CB;SOS1;FOXO3;SOS2 |
| **Ras signaling pathway** | 9/232 | 0.001181327 | 0.01042225 | 3.71 | 24.977 | MAPK10;INSR;PDGFC;ABL1;NF1;PIK3CB;SOS1;SOS2;IGF1R |
| **Human papillomavirus infection** | 11/331 | 0.001231974 | 0.01042225 | 3.17 | 21.236 | MAGI1;PATJ;DLG1;MAML2;PARD3;PIK3CB;PPP2R3A;SOS1;SOS2;THBS1;PTK2 |
| **Non-small cell lung cancer** | 5/72 | 0.001232109 | 0.01042225 | 6.78 | 45.410 | PIK3CB;SOS1;FOXO3;SOS2;FHIT |
| **Glucagon signaling pathway** | 6/107 | 0.001244448 | 0.01042225 | 5.41 | 36.199 | PPP3CA;PRKAA2;GNAQ;ITPR2;SIK2;PPARGC1A |
| **Insulin resistance** | 6/108 | 0.001305661 | 0.01049751 | 5.36 | 35.585 | MAPK10;PRKAA2;TBC1D4;INSR;PIK3CB;PPARGC1A |
| **Hepatitis B** | 7/162 | 0.002253753 | 0.01736916 | 4.12 | 25.123 | MAPK10;MAP3K1;BCL2;TAB2;PIK3CB;SOS1;SOS2 |
| **Hippo signaling pathway** | 7/163 | 0.002333171 | 0.01736916 | 4.10 | 24.819 | YAP1;WWTR1;PATJ;DLG1;WWC1;PARD3;TEAD1 |
| **Human immunodeficiency virus 1 infection** | 8/212 | 0.002604384 | 0.01869576 | 3.59 | 21.344 | MAPK10;PPP3CA;GNAQ;BCL2;ITPR2;TAB2;PIK3CB;PTK2 |
| **Gap junction** | 5/88 | 0.002990885 | 0.02072992 | 5.47 | 31.778 | GNAQ;PDGFC;ITPR2;SOS1;SOS2 |
| **Human cytomegalovirus infection** | 8/225 | 0.003741402 | 0.02472444 | 3.37 | 18.832 | AKAP13;PPP3CA;GNAQ;ITPR2;PIK3CB;SOS1;SOS2;PTK2 |
| **Endometrial cancer** | 4/58 | 0.003923461 | 0.02472444 | 6.70 | 37.134 | PIK3CB;SOS1;FOXO3;SOS2 |
| **VEGF signaling pathway** | 4/59 | 0.004172782 | 0.02472444 | 6.58 | 36.052 | PPP3CA;HSPB1;PIK3CB;PTK2 |
| **Axon guidance** | 7/182 | 0.004293571 | 0.02472444 | 3.65 | 19.879 | PPP3CA;PARD3;ABL1;NTN4;PIK3CB;SSH2;PTK2 |
| **Autophagy** | 6/137 | 0.004305251 | 0.02472444 | 4.17 | 22.696 | MAPK10;PRKAA2;DAPK1;BCL2;PIK3CB;IGF1R |
| **Yersinia infection** | 6/137 | 0.004305251 | 0.02472444 | 4.17 | 22.696 | MAPK10;ARHGEF28;GNAQ;TAB2;PIK3CB;PTK2 |
| **Choline metabolism in cancer** | 5/98 | 0.004743578 | 0.02648498 | 4.88 | 26.097 | MAPK10;PDGFC;PIK3CB;SOS1;SOS2 |
| **Apoptosis** | 6/142 | 0.005121587 | 0.02782267 | 4.01 | 21.160 | MAPK10;BCL2;ITPR2;BIRC6;PIK3CB;PTPN13 |
| **Spinocerebellar ataxia** | 6/143 | 0.005297591 | 0.02802147 | 3.98 | 20.870 | MAPK10;GNAQ;ITPR2;RORA;PIK3CB;PUM2 |
| **Chemokine signaling pathway** | 7/192 | 0.005728421 | 0.0295234 | 3.45 | 17.801 | PARD3;GNAQ;PIK3CB;FOXO3;SOS1;SOS2;PTK2 |
| **Cortisol synthesis and secretion** | 4/65 | 0.005895766 | 0.02962622 | 5.93 | 30.446 | GNAQ;ITPR2;PDE8A;PBX1 |
| **Parathyroid hormone synthesis, secretion and action** | 5/106 | 0.006593802 | 0.03232571 | 4.49 | 22.542 | AKAP13;GNAQ;PDE4D;BCL2;ITPR2 |

| | | | | | | |
|---|---|---|---|---|---|---|
| **Fc epsilon RI signaling pathway** | 4/68 | 0.006911793 | 0.03307786 | 5.65 | 28.116 | MAPK10;PIK3CB;SOS1;SOS2 |
| **mTOR signaling pathway** | 6/154 | 0.007537423 | 0.03523307 | 3.68 | 18.008 | PRKAA2;INSR;PIK3CB;SOS1;SOS2;IGF1R |
| **Aldosterone-regulated sodium reabsorption** | 3/37 | 0.007940195 | 0.03590009 | 7.95 | 38.468 | INSR;NEDD4L;PIK3CB |
| **Adherens junction** | 4/71 | 0.008037334 | 0.03590009 | 5.40 | 26.038 | PARD3;INSR;PTPRM;IGF1R |
| **Melanoma** | 4/72 | 0.008437642 | 0.03686883 | 5.32 | 25.396 | PDGFC;MITF;PIK3CB;IGF1R |
| **Glioma** | 4/75 | 0.009716205 | 0.04155228 | 5.09 | 23.600 | PIK3CB;SOS1;SOS2;IGF1R |
| **Chronic myeloid leukemia** | 4/76 | 0.010168785 | 0.04258179 | 5.02 | 23.043 | ABL1;PIK3CB;SOS1;SOS2 |
| **Sphingolipid signaling pathway** | 5/119 | 0.0105929 | 0.04345251 | 3.97 | 18.074 | MAPK10;GNAQ;BCL2;PIK3CB;PPP2R3A |
| **B cell receptor signaling pathway** | 4/81 | 0.01263615 | 0.05079732 | 4.69 | 20.521 | PPP3CA;PIK3CB;SOS1;SOS2 |
| **Osteoclast differentiation** | 5/127 | 0.013741231 | 0.05415662 | 3.71 | 15.916 | MAPK10;PPP3CA;MITF;TAB2;PIK3CB |
| **Type II diabetes mellitus** | 3/46 | 0.014424525 | 0.05575634 | 6.29 | 26.650 | MAPK10;INSR;PIK3CB |
| **NOD-like receptor signaling pathway** | 6/181 | 0.015804892 | 0.05846955 | 3.11 | 12.905 | MAPK10;BCL2;ERBIN;ITPR2;TRPM7;TAB2 |
| **Pathways in cancer** | 12/531 | 0.01587166 | 0.05846955 | 2.12 | 8.785 | MAPK10;DAPK1;MSH3;GNAQ;ABL1;BCL2;MITF;PIK3CB;SOS1;SOS2;PTK2;IGF1R |
| **Dopaminergic synapse** | 5/132 | 0.01599913 | 0.05846955 | 3.57 | 14.743 | MAPK10;PPP3CA;GNAQ;ITPR2;PPP2R3A |
| **Small cell lung cancer** | 4/92 | 0.019326914 | 0.06896298 | 4.11 | 16.201 | BCL2;PIK3CB;FHIT;PTK2 |
| **Fluid shear stress and atherosclerosis** | 5/139 | 0.019556665 | 0.06896298 | 3.38 | 13.290 | MAPK10;PRKAA2;BCL2;PIK3CB;PTK2 |
| **Shigellosis** | 7/246 | 0.020276602 | 0.0698312 | 2.66 | 10.376 | MAPK10;BCL2;ITPR2;TAB2;PIK3CB;FOXO3;PTK2 |
| **Transcriptional misregulation in cancer** | 6/192 | 0.020497714 | 0.0698312 | 2.93 | 11.374 | JMJD1C;MITF;CDK14;PBX1;PTK2;IGF1R |
| **Inflammatory mediator regulation of TRP channels** | 4/98 | 0.023747349 | 0.07955362 | 3.84 | 14.371 | MAPK10;GNAQ;ITPR2;PIK3CB |
| **Phospholipase D signaling pathway** | 5/148 | 0.02484423 | 0.08186377 | 3.16 | 11.691 | INSR;PDGFC;PIK3CB;SOS1;SOS2 |
| **Progesterone-mediated oocyte maturation** | 4/100 | 0.025345932 | 0.08216987 | 3.76 | 13.825 | MAPK10;PIK3CB;IGF1R;CPEB4 |
| **Amoebiasis** | 4/102 | 0.027007904 | 0.08616808 | 3.68 | 13.308 | GNAQ;HSPB1;PIK3CB;PTK2 |
| **C-type lectin receptor signaling pathway** | 4/104 | 0.028733684 | 0.08986525 | 3.61 | 12.817 | MAPK10;PPP3CA;ITPR2;PIK3CB |

| | | | | | | |
|---|---|---|---|---|---|---|
| **Long-term depression** | 3/60 | 0.029060903 | 0.08986525 | 4.74 | 16.770 | GNAQ;ITPR2;IGF1R |
| **Cellular senescence** | 5/156 | 0.03024941 | 0.0921232 | 2.99 | 10.477 | PPP3CA;ITPR2;TRPM7;PIK3CB;FOXO3 |
| **Lipid and atherosclerosis** | 6/215 | 0.033177651 | 0.09851809 | 2.60 | 8.858 | MAPK10;PPP3CA;BCL2;TAB2;PIK3CB;PTK2 |
| **HIF-1 signaling pathway** | 4/109 | 0.033329505 | 0.09851809 | 3.44 | 11.693 | INSR;BCL2;PIK3CB;IGF1R |
| **GnRH secretion** | 3/64 | 0.034270538 | 0.09947083 | 4.43 | 14.937 | GNAQ;ITPR2;PIK3CB |
| **JAK-STAT signaling pathway** | 5/162 | 0.034753801 | 0.09947083 | 2.88 | 9.674 | BCL2;PIK3CB;SOS1;SOS2;PIAS1 |
| **Regulation of actin cytoskeleton** | 6/218 | 0.035136464 | 0.09947083 | 2.56 | 8.585 | PDGFC;PIK3CB;SSH2;SOS1;SOS2;PTK2 |
| **Cholinergic synapse** | 4/113 | 0.037297611 | 0.1041225 | 3.31 | 10.889 | GNAQ;BCL2;ITPR2;PIK3CB |
| **Acute myeloid leukemia** | 3/67 | 0.038473421 | 0.10450213 | 4.22 | 13.747 | PIK3CB;SOS1;SOS2 |
| **Long-term potentiation** | 3/67 | 0.038473421 | 0.10450213 | 4.22 | 13.747 | PPP3CA;GNAQ;ITPR2 |
| **Hepatocellular carcinoma** | 5/168 | 0.039652958 | 0.10537096 | 2.77 | 8.949 | PIK3CB;SOS1;ARID1B;SOS2;IGF1R |
| **Mitophagy** | 3/68 | 0.039930113 | 0.10537096 | 4.15 | 13.380 | MAPK10;MITF;FOXO3 |
| **Adipocytokine signaling pathway** | 3/69 | 0.041414458 | 0.10537096 | 4.09 | 13.027 | MAPK10;PRKAA2;PPARGC1A |
| **Renal cell carcinoma** | 3/69 | 0.041414458 | 0.10537096 | 4.09 | 13.027 | PIK3CB;SOS1;SOS2 |
| **Renin secretion** | 3/69 | 0.041414458 | 0.10537096 | 4.09 | 13.027 | PPP3CA;GNAQ;ITPR2 |
| **Thyroid hormone signaling pathway** | 4/121 | 0.04601508 | 0.11463854 | 3.08 | 9.493 | NCOA2;TBC1D4;PIK3CB;MED13L |
| **Circadian rhythm** | 2/31 | 0.04619762 | 0.11463854 | 6.19 | 19.036 | PRKAA2;RORA |
| **Oocyte meiosis** | 4/129 | 0.055772074 | 0.13506249 | 2.88 | 8.327 | PPP3CA;ITPR2;IGF1R;CPEB4 |
| **Relaxin signaling pathway** | 4/129 | 0.055772074 | 0.13506249 | 2.88 | 8.327 | MAPK10;PIK3CB;SOS1;SOS2 |
| **MicroRNAs in cancer** | 7/310 | 0.058313274 | 0.13803369 | 2.09 | 5.947 | ABL1;BCL2;PIK3CB;SOS1;SOS2;THBS1;FOXP1 |
| **Natural killer cell mediated cytotoxicity** | 4/131 | 0.058372454 | 0.13803369 | 2.84 | 8.066 | PPP3CA;PIK3CB;SOS1;SOS2 |
| **Endocytosis** | 6/252 | 0.062584977 | 0.14627419 | 2.21 | 6.112 | ARFGEF1;PARD3;AGAP1;NEDD4L;WWP1;IGF1R |
| **Apelin signaling pathway** | 4/137 | 0.066555408 | 0.15376594 | 2.71 | 7.344 | PRKAA2;GNAQ;ITPR2;PPARGC1A |
| **Measles** | 4/139 | 0.069408997 | 0.15853646 | 2.67 | 7.122 | MAPK10;BCL2;TAB2;PIK3CB |
| **ECM-receptor interaction** | 3/88 | 0.074645435 | 0.16858126 | 3.17 | 8.236 | FRAS1;THBS1;FREM2 |
| **Bladder cancer** | 2/41 | 0.075856116 | 0.16941199 | 4.60 | 11.866 | DAPK1;THBS1 |
| **Breast cancer** | 4/147 | 0.08144008 | 0.17941344 | 2.52 | 6.318 | PIK3CB;SOS1;SOS2;IGF1R |
| **Th1 and Th2 cell differentiation** | 3/92 | 0.082783583 | 0.17941344 | 3.03 | 7.551 | MAPK10;PPP3CA;MAML2 |

| | | | | | | |
|---|---|---|---|---|---|---|
| **Retrograde endocannabinoid signaling** | 4/148 | 0.083012188 | 0.17941344 | 2.50 | 6.226 | MAPK10;GNAQ;NAPEPLD;ITPR2 |
| **Gastric cancer** | 4/149 | 0.084599202 | 0.18077248 | 2.48 | 6.136 | BCL2;PIK3CB;SOS1;SOS2 |
| **Sulfur relay system** | 1/8 | 0.085439731 | 0.18077248 | 12.78 | 31.439 | TST |
| **Oxytocin signaling pathway** | 4/154 | 0.092754917 | 0.19367699 | 2.40 | 5.709 | PPP3CA;PRKAA2;GNAQ;ITPR2 |
| **Cushing syndrome** | 4/155 | 0.094429577 | 0.19367699 | 2.38 | 5.628 | GNAQ;ITPR2;PDE8A;PBX1 |
| **Non-alcoholic fatty liver disease** | 4/155 | 0.094429577 | 0.19367699 | 2.38 | 5.628 | MAPK10;PRKAA2;INSR;PIK3CB |
| **Human T-cell leukemia virus 1 infection** | 5/219 | 0.097547703 | 0.19662158 | 2.11 | 4.903 | MAPK10;PPP3CA;DLG1;MAP3K1;PIK3CB |
| **Hepatitis C** | 4/157 | 0.097821681 | 0.19662158 | 2.35 | 5.471 | PIK3CB;SOS1;SOS2;PIAS1 |
| **AGE-RAGE signaling pathway in diabetic complications** | 3/100 | 0.100133328 | 0.19927524 | 2.78 | 6.396 | MAPK10;BCL2;PIK3CB |
| **Necroptosis** | 4/159 | 0.101270194 | 0.19956185 | 2.32 | 5.319 | MAPK10;FAF1;BCL2;TRPM7 |
| **Chagas disease** | 3/102 | 0.104682081 | 0.20222282 | 2.72 | 6.145 | MAPK10;GNAQ;PIK3CB |
| **Pancreatic secretion** | 3/102 | 0.104682081 | 0.20222282 | 2.72 | 6.145 | GNAQ;ITPR2;SLC4A4 |
| **Sulfur metabolism** | 1/10 | 0.105638789 | 0.20222282 | 9.94 | 22.341 | TST |
| **Toll-like receptor signaling pathway** | 3/104 | 0.109311009 | 0.20644812 | 2.67 | 5.908 | MAPK10;TAB2;PIK3CB |
| **Ovarian steroidogenesis** | 2/51 | 0.109900243 | 0.20644812 | 3.66 | 8.083 | INSR;IGF1R |
| **cGMP-PKG signaling pathway** | 4/167 | 0.115612232 | 0.21344223 | 2.21 | 4.764 | PPP3CA;INSR;GNAQ;ITPR2 |
| **Th17 cell differentiation** | 3/107 | 0.116399868 | 0.21344223 | 2.59 | 5.573 | MAPK10;PPP3CA;RORA |
| **Thermogenesis** | 5/232 | 0.116809179 | 0.21344223 | 1.98 | 4.261 | PRKAA2;SOS1;PPARGC1A;ARID1B;SOS2 |
| **Regulation of lipolysis in adipocytes** | 2/55 | 0.124458637 | 0.22537105 | 3.38 | 7.050 | INSR;PIK3CB |
| **TNF signaling pathway** | 3/112 | 0.128583829 | 0.22871991 | 2.47 | 5.070 | MAPK10;TAB2;PIK3CB |
| **Toxoplasmosis** | 3/112 | 0.128583829 | 0.22871991 | 2.47 | 5.070 | MAPK10;BCL2;TAB2 |
| **Glutamatergic synapse** | 3/114 | 0.133580028 | 0.23552268 | 2.43 | 4.886 | PPP3CA;GNAQ;ITPR2 |
| **Notch signaling pathway** | 2/59 | 0.139446601 | 0.24372841 | 3.15 | 6.196 | MAML2;NUMB |
| **Salmonella infection** | 5/249 | 0.144480824 | 0.25035039 | 1.84 | 3.569 | MAPK10;EXOC4;BCL2;TAB2;PIK3CB |
| **Platelet activation** | 3/124 | 0.159515217 | 0.27403896 | 2.23 | 4.085 | GNAQ;ITPR2;PIK3CB |

| | | | | | | |
|---|---|---|---|---|---|---|
| **Kaposi sarcoma-associated herpesvirus infection** | 4/193 | 0.167682376 | 0.28562845 | 1.90 | 3.396 | MAPK10;PPP3CA;ITPR2;PIK3CB |
| **Purine metabolism** | 3/129 | 0.17301881 | 0.29224186 | 2.14 | 3.748 | PDE4D;PDE8A;FHIT |
| **RIG-I-like receptor signaling pathway** | 2/70 | 0.182366113 | 0.30546324 | 2.64 | 4.484 | MAPK10;MAP3K1 |
| **Epstein-Barr virus infection** | 4/202 | 0.187374118 | 0.31125783 | 1.81 | 3.039 | MAPK10;BCL2;TAB2;PIK3CB |
| **Diabetic cardiomyopathy** | 4/203 | 0.189607224 | 0.31238567 | 1.81 | 3.002 | MAPK10;TBC1D4;INSR;PIK3CB |
| **p53 signaling pathway** | 2/73 | 0.194393749 | 0.31766783 | 2.52 | 4.133 | BCL2;THBS1 |
| **Thyroid hormone synthesis** | 2/75 | 0.202467631 | 0.3281935 | 2.45 | 3.919 | GNAQ;ITPR2 |
| **Gastric acid secretion** | 2/76 | 0.206519015 | 0.329447 | 2.42 | 3.818 | GNAQ;ITPR2 |
| **Pancreatic cancer** | 2/76 | 0.206519015 | 0.329447 | 2.42 | 3.818 | MAPK10;PIK3CB |
| **Bacterial invasion of epithelial cells** | 2/77 | 0.210579085 | 0.33327871 | 2.39 | 3.721 | PIK3CB;PTK2 |
| **Signaling pathways regulating pluripotency of stem cells** | 3/143 | 0.21237065 | 0.33348829 | 1.92 | 2.977 | PIK3CB;SMAD5;IGF1R |
| **Mismatch repair** | 1/23 | 0.226530474 | 0.3469425 | 4.06 | 6.034 | MSH3 |
| **Protein export** | 1/23 | 0.226530474 | 0.3469425 | 4.06 | 6.034 | IMMP2L |
| **Proximal tubule bicarbonate reclamation** | 1/23 | 0.226530474 | 0.3469425 | 4.06 | 6.034 | SLC4A4 |
| **Alzheimer disease** | 6/369 | 0.227842838 | 0.3469425 | 1.49 | 2.197 | MAPK10;PPP3CA;INSR;GNAQ;ITPR2;PIK3CB |
| **Adrenergic signaling in cardiomyocytes** | 3/150 | 0.232726563 | 0.35171458 | 1.83 | 2.667 | GNAQ;BCL2;PPP2R3A |
| **Folate biosynthesis** | 1/26 | 0.252032394 | 0.37804859 | 3.58 | 4.927 | GPHN |
| **Coronavirus disease** | 4/232 | 0.257536565 | 0.3810794 | 1.57 | 2.134 | MAPK10;RPS28;TAB2;PIK3CB |
| **GABAergic synapse** | 2/89 | 0.259740685 | 0.3810794 | 2.06 | 2.774 | PLCL1;GPHN |
| **PD-L1 expression and PD-1 checkpoint pathway in cancer** | 2/89 | 0.259740685 | 0.3810794 | 2.06 | 2.774 | PPP3CA;PIK3CB |
| **Morphine addiction** | 2/91 | 0.267971515 | 0.39030634 | 2.01 | 2.648 | PDE4D;PDE8A |
| **Chemical carcinogenesis** | 4/239 | 0.274644681 | 0.39400867 | 1.53 | 1.972 | BCL2;PIK3CB;SOS1;SOS2 |
| **Salivary secretion** | 2/93 | 0.276201336 | 0.39400867 | 1.97 | 2.530 | GNAQ;ITPR2 |

| | | | | | | |
|---|---|---|---|---|---|---|
| **Calcium signaling pathway** | 4/240 | 0.277104844 | 0.39400867 | 1.52 | 1.950 | PPP3CA;GNAQ;PDGFC;ITPR2 |
| **IL-17 signaling pathway** | 2/94 | 0.280314627 | 0.39400867 | 1.95 | 2.474 | MAPK10;TAB2 |
| **TGF-beta signaling pathway** | 2/94 | 0.280314627 | 0.39400867 | 1.95 | 2.474 | THBS1;SMAD5 |
| **Phosphatidylinositol signaling system** | 2/97 | 0.292642325 | 0.40572722 | 1.88 | 2.315 | ITPR2;PIK3CB |
| **RNA polymerase** | 1/31 | 0.292688794 | 0.40572722 | 2.98 | 3.660 | POLR2I |
| **Protein processing in endoplasmic reticulum** | 3/171 | 0.295475333 | 0.40575471 | 1.60 | 1.949 | MAPK10;BCL2;MAN1A1 |
| **Aldosterone synthesis and secretion** | 2/98 | 0.296745979 | 0.40575471 | 1.86 | 2.264 | GNAQ;ITPR2 |
| **Melanogenesis** | 2/101 | 0.3090341 | 0.41970172 | 1.81 | 2.122 | GNAQ;MITF |
| **Pentose and glucuronate interconversions** | 1/34 | 0.316018657 | 0.423465 | 2.71 | 3.119 | DCXR |
| **Propanoate metabolism** | 1/34 | 0.316018657 | 0.423465 | 2.71 | 3.119 | BCKDHB |
| **NF-kappa B signaling pathway** | 2/104 | 0.321279324 | 0.4268044 | 1.75 | 1.991 | BCL2;TAB2 |
| **Tuberculosis** | 3/180 | 0.322757559 | 0.4268044 | 1.52 | 1.715 | MAPK10;PPP3CA;BCL2 |
| **Starch and sucrose metabolism** | 1/36 | 0.331144692 | 0.43503322 | 2.55 | 2.821 | GBE1 |
| **African trypanosomiasis** | 1/37 | 0.338582361 | 0.44191594 | 2.48 | 2.687 | GNAQ |
| **Various types of N-glycan biosynthesis** | 1/39 | 0.353211592 | 0.45803568 | 2.35 | 2.446 | MAN1A1 |
| **Serotonergic synapse** | 2/113 | 0.357656535 | 0.46008379 | 1.61 | 1.656 | GNAQ;ITPR2 |
| **Prion disease** | 4/273 | 0.359617274 | 0.46008379 | 1.33 | 1.361 | MAPK10;PPP3CA;ITPR2;PIK3CB |
| **Leukocyte transendothelial migration** | 2/114 | 0.361657901 | 0.46008379 | 1.60 | 1.623 | PIK3CB;PTK2 |
| **Pathogenic Escherichia coli infection** | 3/197 | 0.374228104 | 0.47308081 | 1.38 | 1.359 | MAPK10;ABL1;TAB2 |
| **Cell cycle** | 2/124 | 0.401117865 | 0.50390432 | 1.46 | 1.338 | STAG1;ABL1 |
| **Carbohydrate digestion and absorption** | 1/47 | 0.408576927 | 0.51008672 | 1.94 | 1.737 | PIK3CB |
| **Valine, leucine and isoleucine degradation** | 1/48 | 0.415157174 | 0.51510242 | 1.90 | 1.670 | BCKDHB |

| | | | | | | |
|---|---|---|---|---|---|---|
| **Cysteine and methionine metabolism** | 1/50 | 0.428099812 | 0.52150341 | 1.82 | 1.546 | TST |
| **Malaria** | 1/50 | 0.428099812 | 0.52150341 | 1.82 | 1.546 | THBS1 |
| **N-Glycan biosynthesis** | 1/50 | 0.428099812 | 0.52150341 | 1.82 | 1.546 | MAN1A1 |
| **cAMP signaling pathway** | 3/216 | 0.430793268 | 0.52162317 | 1.26 | 1.060 | MAPK10;PDE4D;PIK3CB |
| **Vascular smooth muscle contraction** | 2/133 | 0.435633218 | 0.52432501 | 1.36 | 1.133 | GNAQ;ITPR2 |
| **Huntington disease** | 4/306 | 0.442034048 | 0.52886216 | 1.18 | 0.966 | MAPK10;GNAQ;POLR2I;PPARGC1A |
| **Endocrine and other factor-regulated calcium reabsorption** | 1/53 | 0.446981052 | 0.53161652 | 1.72 | 1.382 | GNAQ |
| **Hedgehog signaling pathway** | 1/56 | 0.465241644 | 0.55007983 | 1.62 | 1.242 | BCL2 |
| **Mineral absorption** | 1/60 | 0.488658657 | 0.57104878 | 1.51 | 1.083 | TRPM7 |
| **Viral myocarditis** | 1/60 | 0.488658657 | 0.57104878 | 1.51 | 1.083 | ABL1 |
| **Lysine degradation** | 1/63 | 0.505548918 | 0.58737186 | 1.44 | 0.981 | KMT2C |
| **Inflammatory bowel disease** | 1/65 | 0.516499358 | 0.59664581 | 1.39 | 0.921 | RORA |
| **Amphetamine addiction** | 1/69 | 0.537681194 | 0.61643485 | 1.31 | 0.814 | PPP3CA |
| **Central carbon metabolism in cancer** | 1/70 | 0.542830685 | 0.61643485 | 1.29 | 0.790 | PIK3CB |
| **Epithelial cell signaling in Helicobacter pylori infection** | 1/70 | 0.542830685 | 0.61643485 | 1.29 | 0.790 | MAPK10 |
| **Wnt signaling pathway** | 2/166 | 0.55210705 | 0.62344673 | 1.09 | 0.646 | MAPK10;PPP3CA |
| **Inositol phosphate metabolism** | 1/73 | 0.557939062 | 0.62651258 | 1.24 | 0.723 | PIK3CB |
| **Pertussis** | 1/76 | 0.572550315 | 0.63934785 | 1.19 | 0.663 | MAPK10 |
| **Leishmaniasis** | 1/77 | 0.577313092 | 0.64110459 | 1.17 | 0.644 | TAB2 |
| **Pathways of neurodegeneration** | 5/475 | 0.609647036 | 0.67329151 | 0.95 | 0.468 | MAPK10;PPP3CA;GNAQ;BCL2;ITPR2 |
| **Alcoholism** | 2/186 | 0.614048311 | 0.67444651 | 0.97 | 0.472 | SOS1;SOS2 |
| **Insulin secretion** | 1/86 | 0.617869314 | 0.67495507 | 1.05 | 0.505 | GNAQ |
| **Bile secretion** | 1/90 | 0.634627544 | 0.68580718 | 1.00 | 0.455 | SLC4A4 |
| **Hypertrophic cardiomyopathy** | 1/90 | 0.634627544 | 0.68580718 | 1.00 | 0.455 | PRKAA2 |
| **Viral carcinogenesis** | 2/203 | 0.661395045 | 0.70425809 | 0.89 | 0.366 | DLG1;PIK3CB |

| | | | | | | |
|---|---|---|---|---|---|---|
| **Circadian entrainment** | 1/97 | 0.662212829 | 0.70425809 | 0.93 | 0.382 | GNAQ |
| **Fc gamma R-mediated phagocytosis** | 1/97 | 0.662212829 | 0.70425809 | 0.93 | 0.382 | PIK3CB |
| **mRNA surveillance pathway** | 1/98 | 0.665980525 | 0.70453729 | 0.92 | 0.373 | PPP2R3A |
| **Lysosome** | 1/128 | 0.76148455 | 0.80135285 | 0.70 | 0.191 | SUMF1 |
| **Parkinson disease** | 2/249 | 0.766330896 | 0.80225266 | 0.72 | 0.191 | MAPK10;ITPR2 |
| **Cell adhesion molecules** | 1/148 | 0.809502265 | 0.8430477 | 0.60 | 0.128 | PTPRM |
| **Spliceosome** | 1/150 | 0.813739091 | 0.8430477 | 0.60 | 0.123 | HNRNPM |
| **Phagosome** | 1/152 | 0.8178821 | 0.8430477 | 0.59 | 0.118 | THBS1 |
| **Ribosome** | 1/158 | 0.829768685 | 0.85093625 | 0.57 | 0.106 | RPS28 |
| **Influenza A** | 1/172 | 0.854585587 | 0.87193758 | 0.52 | 0.082 | PIK3CB |
| **Neutrophil extracellular trap formation** | 1/189 | 0.879926734 | 0.89325896 | 0.47 | 0.060 | PIK3CB |
| **Neuroactive ligand-receptor interaction** | 2/341 | 0.894710113 | 0.90370217 | 0.52 | 0.058 | PARD3;PTH2R |
| **Amyotrophic lateral sclerosis** | 2/364 | 0.914492716 | 0.91717222 | 0.49 | 0.044 | PPP3CA;BCL2 |
| **Herpes simplex virus 1 infection** | 3/498 | 0.917172223 | 0.91717222 | 0.53 | 0.046 | BCL2;TAB2;PIK3CB |

Supplementary Table S6b. Suppressed pathways using KEGG 2021

| Term | Overlap | P-value | Adjusted P-value | Odds Ratio | Combined Score | Genes |
|---|---|---|---|---|---|---|
| **Oxidative phosphorylation** | 47/133 | 9.701136E-36 | 2.64841022879219E-33 | 17.08 | 1376.95 | NDUFB9;COX7B;NDUFB7;NDUFB10;UQCRB;NDUFB6;NDUFA12;NDUFB5;NDUFB4;NDUFB3;NDUFB2;ATP5MC3;COX7A2;UQCR11;COX5B;COX7C;COX5A;UQCRH;ATP5MC1;ATP5F1A;ATP5F1B;UQCRFS1;CYC1;ATP6V0E2;ATP5MG;ATP6V1F;ATP5PF;NDUFA8;ATP6V1G1;NDUFA6;NDUFA5;SDHC;NDUFC1;SDHD;SDHA;COX6C;COX6B1;COX7A2L;UQCRQ;NDUFS6;NDUFS5;NDUFAB1;UQCRC1;ATP5PO;NDUFS1;ATP6V0D1;ATP6V0C |
| **Parkinson disease** | 60/249 | 1.1657281E-34 | 1.59121881109305E-32 | 10.08 | 787.66 | COX7B;NDUFA12;PARK7;COX7C;TXN2;TUBA1C;TUBA1B;KIF5B;UQCRFS1;SDHC;NDUFC1;SDHD;SDHA;COX6B1;COX7A2L;NDUFS6;DDIT3;NDUFS5;UQCRC1;PPIF;VDAC2;VDAC1;NDUFS1;SLC25A5;SLC25A4;NDUFB9;PSMD12;NDUFB7;NDUFB10;UQCRB;NDUFB6;NDUFB5;NDUFB4;NDUFB3;NDUFB2;ATP5MC3;UQCR11;COX7A2;TXN;COX5B;COX5A;UQCRH;ATP5MC1;ATP5F1A;ATP5F1B;CYC1;ATP5PF;NDUFA8;XBP1;NDUFA6;NDUFA5;COX6C;TUBB4B;PINK1;UQCRQ;NDUFAB1;ATP5PO;CYCS;CALM3;CALM1 |
| **Diabetic cardiomyopathy** | 54/203 | 1.1640613E-33 | 1.05929582191234E-31 | 11.42 | 865.93 | COX7B;NDUFA12;ATP2A2;COX7C;MPC1;MPC2;UQCRFS1;RAC1;CTSD;SDHC;NDUFC1;SDHD;SDHA;COX6B1;COX7A2L;NDUFS6;NDUFS5;UQCRC1;PPIF;VDAC2;VDAC1;NDUFS1;SLC25A5;GAPDH;SLC25A4;NDUFB9;NDUFB7;NDUFB10;UQCRB;NDUFB6;NDUFB5;NDUFB4;NDUFB3;NDUFB2;ATP5MC3;UQCR11;COX7A2;PIK3R1;PDHB;COX5B;COX5A;UQCRH;ATP5MC1;ATP5F1A;ATP5F1B;CYC1;ATP5PF;NDUFA8;NDUFA6;NDUFA5;COX6C;UQCRQ;NDUFAB1;ATP5PO |

| | | | | | | |
|---|---|---|---|---|---|---|
| **Thermogenesis** | 54/232 | 1.8722222E-30 | 1.27779167416531E-28 | 9.54 | 653.29 | COX7B;NDUFA12;COX7C;ACTB;ACTG1;UQCRFS1;HRAS;COA6;SDHC;NDUFC1;SDHD;SDHA;COX6B1;COX7A2L;NDUFS6;NDUFS5;UQCRC1;NDUFS1;MGLL;NDUFB9;NDUFB7;NDUFB10;UQCRB;NDUFB6;NDUFB5;NDUFB4;NDUFB3;NDUFB2;ATP5MC3;UQCR11;COX7A2;COX5B;COX5A;UQCRH;ATP5MC1;ATP5F1A;ATP5F1B;COX14;CYC1;ATP5MG;COA4;ATP5PF;COA3;NDUFA8;NDUFA6;NDUFA5;COX6C;SMARCA4;NDUFAF8;UQCRQ;NDUFAF4;NDUFAB1;ATP5PO;NDUFAF3 |
| **Amyotrophic lateral sclerosis** | 65/364 | 2.8867312E-29 | 1.57615526168033E-27 | 6.92 | 454.81 | COX7B;NDUFA12;COX7C;ACTB;ACTG1;TUBA1C;TUBA1B;KIF5B;UQCRFS1;RAC1;GABARAPL2;GABARAPL1;SDHC;NDUFC1;SDHD;SDHA;COX6B1;COX7A2L;NDUFS6;DDIT3;NDUFS5;CAT;UQCRC1;SRSF3;VDAC1;NDUFS1;PFN1;PFN2;NDUFB9;PSMD12;VCP;NDUFB7;NDUFB10;UQCRB;NDUFB6;NDUFB5;NDUFB4;NDUFB3;NDUFB2;ATP5MC3;UQCR11;COX7A2;COX5B;COX5A;UQCRH;ATP5MC1;ATP5F1A;ATP5F1B;CYC1;ATP5PF;NDUFA8;XBP1;HNRNPA3;NDUFA6;NDUFA5;CHCHD10;COX6C;TUBB4B;PINK1;UQCRQ;HNRNPA2B1;NDUFAB1;CHMP2B;ATP5PO;CYCS |
| **Prion disease** | 55/273 | 1.3038388E-27 | 5.93246637868899E-26 | 7.93 | 491.12 | COX7B;NDUFA12;COX7C;TUBA1C;TUBA1B;KIF5B;UQCRFS1;RAC1;SDHC;NDUFC1;SDHD;SDHA;COX6B1;COX7A2L;NDUFS6;DDIT3;NDUFS5;UQCRC1;PPIF;VDAC2;VDAC1;NDUFS1;SLC25A5;SLC25A4;NDUFB9;PSMD12;NDUFB7;NDUFB10;UQCRB;NDUFB6;NDUFB5;NDUFB4;NDUFB3;NDUFB2;ATP5MC3;UQCR11;COX7A2;PIK3R1;COX5B;COX5A;UQCRH;ATP5MC1;ATP5F1A;ATP5F1B;CYC1;ATP5PF;NDUFA8;NDUFA6;NDUFA5;COX6C;TUBB4B;UQCRQ;NDUFAB1;ATP5PO;CYCS |

| | | | | | | |
|---|---|---|---|---|---|---|
| **Alzheimer disease** | 63/369 | 2.9744575E-27 | 1.16003843079567E-25 | 6.53 | 398.87 | COX7B;NDUFA12;ATP2A2;COX7C;TUBA1C;TUBA1B;KIF5B;CAPN2;UQCRFS1;CAPN1;HRAS;SDHC;NDUFC1;SDHD;SDHA;COX6B1;COX7A2L;NDUFS6;DDIT3;NDUFS5;UQCRC1;PPIF;VDAC2;VDAC1;NDUFS1;SLC25A5;GAPDH;SLC25A4;NDUFB9;PSMD12;NDUFB7;NDUFB10;UQCRB;NDUFB6;NDUFB5;NDUFB4;NDUFB3;NDUFB2;ATP5MC3;UQCR11;COX7A2;PIK3R1;COX5B;COX5A;UQCRH;ATP5MC1;APH1A;ATP5F1A;ATP5F1B;CYC1;ATP5PF;NDUFA8;XBP1;NDUFA6;NDUFA5;COX6C;TUBB4B;UQCRQ;NDUFAB1;ATP5PO;CYCS;CALM3;CALM1 |
| **Non-alcoholic fatty liver disease** | 42/155 | 9.3083614E-27 | 3.17647833366842E-25 | 11.51 | 689.65 | NDUFB9;COX7B;NDUFB7;NDUFB10;UQCRB;NDUFB6;NDUFA12;NDUFB5;NDUFB4;NDUFB3;NDUFB2;COX7A2;UQCR11;PIK3R1;ADIPOR1;COX5B;COX7C;COX5A;UQCRH;CDC42;UQCRFS1;CYC1;RAC1;NDUFA8;XBP1;NDUFA6;NDUFA5;SDHC;NDUFC1;SDHD;SDHA;COX6C;COX6B1;COX7A2L;UQCRQ;NDUFS6;DDIT3;NDUFS5;NDUFAB1;UQCRC1;CYCS;NDUFS1 |
| **Pathways of neurodegeneration** | 68/475 | 1.0078635E-24 | 3.05718596942679E-23 | 5.32 | 293.71 | COX7B;NDUFA12;ATP2A2;PARK7;COX7C;TUBA1C;TUBA1B;KIF5B;CAPN2;UQCRFS1;RAC1;CAPN1;HRAS;GABARAPL2;GABARAPL1;NDUFC1;SDHC;SDHD;SDHA;COX6B1;COX7A2L;NDUFS6;DDIT3;NDUFS5;CAT;UQCRC1;PPIF;VDAC2;VDAC1;NDUFS1;SLC25A5;SLC25A4;NDUFB9;PSMD12;VCP;NDUFB7;UQCRB;NDUFB6;NDUFB10;NDUFB5;NDUFB4;NDUFB3;NDUFB2;ATP5MC3;UQCR11;COX7A2;COX5B;COX5A;UQCRH;ATP5MC1;ATP5F1A;ATP5F1B;CYC1;NDUFA8;ATP5PF;XBP1;NDUFA6;NDUFA5;TUBB4B;COX6C;PINK1;UQCRQ;NDUFAB1;CHMP2B;ATP5PO;CYCS;CALM3;CALM1 |

| | | | | | | |
|---|---|---|---|---|---|---|
| **Huntington disease** | 54/306 | 3.3926793E-24 | 9.26201455137755E-23 | 6.72 | 362.90 | COX7B;NDUFA12;CLTB;COX7C;TUBA1C;TUBA1B;KIF5B;UQCRFS1;SDHC;NDUFC1;SDHD;SDHA;COX6B1;COX7A2L;NDUFS6;NDUFS5;UQCRC1;PPIF;VDAC2;VDAC1;NDUFS1;SLC25A5;SLC25A4;NDUFB9;PSMD12;NDUFB7;NDUFB10;UQCRB;NDUFB6;NDUFB5;NDUFB4;NDUFB3;NDUFB2;ATP5MC3;UQCR11;COX7A2;COX5B;COX5A;UQCRH;ATP5MC1;ATP5F1A;ATP5F1B;POLR2C;CYC1;ATP5PF;NDUFA8;NDUFA6;NDUFA5;COX6C;TUBB4B;UQCRQ;NDUFAB1;ATP5PO;CYCS |
| **Protein processing in endoplasmic reticulum** | 27/171 | 1.5458014E-11 | 3.83639790068343E-10 | 5.66 | 140.85 | VCP;TRAM1;HSP90AB1;RPN1;SEL1L;RRBP1;SEC61A1;LMAN1;GANAB;OS9;BAG1;CAPN2;CAPN1;SEC61B;SEC62;XBP1;EIF2AK1;SSR3;PDIA6;PDIA4;RBX1;DDIT3;CANX;DNAJA2;STUB1;CALR;P4HB |
| **Cardiac muscle contraction** | 18/87 | 4.2247582E-10 | 9.61132498007029E-09 | 7.79 | 168.21 | COX7B;UQCRB;TPM3;ATP2A2;COX7A2;UQCR11;ATP1B1;COX5B;COX6C;COX7C;COX5A;UQCRH;COX6B1;COX7A2L;UQCRQ;UQCRC1;UQCRFS1;CYC1 |
| **Phagosome** | 23/152 | 1.1800821E-09 | 2.47817242633477E-08 | 5.35 | 110.01 | ITGB1;ATP6V1G1;RAB5C;HLA-B;M6PR;HLA-C;HLA-A;TUBB4B;ACTB;ACTG1;HLA-E;SEC61A1;TUBA1C;TUBA1B;LAMP1;CANX;CALR;RAC1;SEC61B;ATP6V0D1;ATP6V0E2;ATP6V0C;ATP6V1F |
| **Salmonella infection** | 29/249 | 4.5611834E-09 | 8.89430770726853E-08 | 3.97 | 76.34 | CYFIP1;ARF1;RAB5C;HSP90AB1;ARPC5L;BRK1;TXN;ACTB;MYL12A;ACTG1;TXN2;CDC42;TUBA1C;TUBA1B;KIF5B;FLNA;RAC1;HRAS;DYNLT1;M6PR;ARPC5;DYNLL1;TUBB4B;NFKBIA;CYCS;PFN1;GAPDH;ARL8A;PFN2 |
| **Shigellosis** | 27/246 | 5.4499919E-08 | 9.9189851737526E-07 | 3.71 | 61.98 | ITGB1;ARF1;ARPC5L;PIK3R1;ACTB;MYL12A;ACTG1;CDC42;CAPNS1;CAPN2;RAC1;CAPN1;WASF2;GABARAPL2;GABARAPL1;ACTN4;ARPC5;RBX1;NFKBIA;CTTN;UBE2V2;UBE2N;CYCS;VDAC1;UBE2V1;PFN1;PFN2 |
| **Vibrio cholerae infection** | 12/50 | 6.1029182E-08 | 1.04131041290949E-06 | 9.36 | 155.51 | SEC61A1;ARF1;ATP6V1G1;KDELR2;SEC61B;ATP6V0D1;ATP6V0E2;ATP6V0C;ACTB;ACTG1;PDIA4;ATP6V1F |
| **Pathogenic Escherichia coli infection** | 23/197 | 1.7212812E-07 | 2.7641751712821E-06 | 3.96 | 61.64 | ITGB1;CYFIP1;ARF1;TMED10;ARPC5L;BRK1;ARPC5;TUBB4B;ACTB;ACTG1;NFKBIA;CDC42;TUBA1C;TUBA1B;CTTN;NCL;CLDN7;CYCS;TMBIM6;RAC1;EZR;GAPDH;WASF2 |

| | | | | | | |
|---|---|---|---|---|---|---|
| **Retrograde endocannabinoid signaling** | 19/148 | 4.6578467E-07 | 7.06440090169338E-06 | 4.39 | 64.05 | NDUFB9;NDUFA8;NDUFB7;NDUFA6;NDUFB10;NDUFB6;NDUFA5;NDUFA12;NDUFB5;NDUFB4;NDUFB3;NDUFB2;NDUFC1;GNG5;NDUFS6;NDUFS5;NDUFAB1;NDUFS1;MGLL |
| **Tight junction** | 19/169 | 3.5415756E-06 | 5.08868498869928E-05 | 3.77 | 47.37 | ITGB1;ARPC5L;MSN;ACTN4;ARPC5;ACTB;MYL12A;ACTG1;CDC42;TUBA1C;TUBA1B;CCND1;CTTN;PPP2R1A;CLDN7;PPP2R2D;RAB13;RAC1;EZR |
| **Viral myocarditis** | 11/60 | 3.8202664E-06 | 5.21466362942114E-05 | 6.64 | 82.85 | CCND1;SGCB;HLA-B;HLA-C;CYCS;RAC1;HLA-A;ACTB;EIF4G2;ACTG1;HLA-E |
| **Citrate cycle (TCA cycle)** | 8/30 | 4.2859987E-06 | 5.57179826788421E-05 | 10.72 | 132.54 | MDH2;IDH2;OGDHL;SDHC;PDHB;SDHD;PCK1;SDHA |
| **Glycolysis / Gluconeogenesis** | 11/67 | 1.1608471E-05 | 1.44050566247126E-04 | 5.81 | 66.01 | LDHA;PFKL;TPI1;PKM;ALDH2;PGAM1;ENO1;PDHB;PCK1;GAPDH;ALDH9A1 |
| **Viral carcinogenesis** | 20/203 | 1.4674673E-05 | 1.74181984036005E-04 | 3.26 | 36.23 | YWHAE;YWHAB;STAT3;HLA-B;MRPS18B;HLA-C;CHD4;ACTN4;HLA-A;PIK3R1;HLA-E;DDB1;NFKBIA;CDC42;PKM;CCND1;RAC1;ATP6V0D1;HRAS;YWHAH |
| **Ribosome** | 17/158 | 2.0423956E-05 | 2.32322502475866E-04 | 3.58 | 38.69 | RPL4;MRPS16;MRPS14;MRPL18;RPLP0;RPS27L;MRPL15;MRPL12;MRPL34;MRPS7;MRPL33;MRPL20;RPL36AL;RPL14;RPS20;RPS10;RPS13 |
| **Bacterial invasion of epithelial cells** | 11/77 | 4.4722358E-05 | 4.88368154286146E-04 | 4.93 | 49.34 | ITGB1;CDC42;CTTN;ARPC5L;CLTB;RAC1;PIK3R1;ARPC5;WASF2;ACTB;ACTG1 |
| **RNA transport** | 18/186 | 4.9191993E-05 | 5.16515924085583E-04 | 3.18 | 31.59 | CYFIP1;POP5;UBE2I;NCBP2;EIF4A3;THOC7;SRRM1;EIF1;EEF1A1;PNN;EIF5;SUMO1;SUMO3;EIF3I;SUMO2;STRAP;SAP18;EIF4G2 |
| **Cellular senescence** | 16/156 | 6.3687244E-05 | 6.43948797489665E-04 | 3.39 | 32.76 | HLA-B;HLA-C;HLA-A;PIK3R1;ZFP36L2;HLA-E;CCND1;CAPN2;VDAC2;VDAC1;CALM3;CAPN1;CALM1;SLC25A5;HRAS;SLC25A4 |
| **Protein export** | 6/23 | 8.0817332E-05 | 7.87968990907119E-04 | 10.38 | 97.80 | SEC61A1;SPCS1;SEC61B;SRP14;SEC62;SRP9 |
| **Human immunodeficiency virus 1 infection** | 19/212 | 8.7133405E-05 | 8.20255847879549E-04 | 2.93 | 27.36 | HLA-B;HLA-C;HLA-A;PIK3R1;RNF7;HLA-E;RBX1;TAPBP;DDB1;NFKBIA;GNG5;GNA11;CYCS;CALM3;CALR;ELOC;RAC1;CALM1;HRAS |
| **Regulation of actin cytoskeleton** | 19/218 | 1.2608535E-04 | 0.001147377 | 2.84 | 25.48 | ITGB1;CYFIP1;ARPC5L;MSN;BRK1;ACTN4;PIK3R1;ARPC5;ACTB;MYL12A;ACTG1;CDC42;TMSB4X;RAC1;EZR;PFN1;HRAS;WASF2;PFN2 |
| **Pyruvate metabolism** | 8/47 | 1.4009991E-04 | 0.001233783 | 6.04 | 53.63 | LDHA;PKM;ALDH2;MDH2;GLO1;PDHB;PCK1;ALDH9A1 |

| | | | | | | |
|---|---|---|---|---|---|---|
| **Arginine and proline metabolism** | 8/50 | 2.1919148E-04 | 0.001869977 | 5.61 | 47.28 | ALDH4A1;AOC1;ALDH2;ODC1;LAP3;SAT1;CNDP2;ALDH9A1 |
| **Antigen processing and presentation** | 10/78 | 2.4708302E-04 | 0.00204405 | 4.34 | 36.04 | CD74;HSP90AB1;CANX;HLA-B;HLA-C;HLA-A;CALR;CTSB;HLA-E;TAPBP |
| **Apoptosis** | 14/142 | 2.7218468E-04 | 0.002185483 | 3.24 | 26.57 | PIK3R1;ACTB;ACTG1;NFKBIA;TUBA1C;TUBA1B;DDIT3;LMNA;CAPN2;CYCS;CAPN1;CTSD;HRAS;CTSB |
| **Spliceosome** | 14/150 | 4.7743423E-04 | 0.003723987 | 3.05 | 23.29 | DDX5;HNRNPA3;NCBP2;EIF4A3;LSM4;ZMAT2;SNRPD1;SNRPG;SRSF3;SNRPE;SNRPF;TXNL4A;SRSF8;SRSF9 |
| **Kaposi sarcoma-associated herpesvirus infection** | 16/193 | 7.2734720E-04 | 0.005411745 | 2.68 | 19.34 | GABARAPL2;GABARAPL1;STAT3;HLA-B;HLA-C;HLA-A;PIK3R1;HLA-E;NFKBIA;CCND1;GNG5;CYCS;CALM3;RAC1;CALM1;HRAS |
| **Fluid shear stress and atherosclerosis** | 13/139 | 7.3677544E-04 | 0.005411745 | 3.05 | 22.00 | HSP90AB1;PIK3R1;TXN;ACTB;ACTG1;TXN2;SUMO1;SUMO3;SUMO2;SDC1;CALM3;RAC1;CALM1 |
| **Hepatitis C** | 14/157 | 7.5328318E-04 | 0.005411745 | 2.90 | 20.82 | YWHAE;CD81;YWHAB;EIF2AK1;STAT3;PIK3R1;NFKBIA;CCND1;PPP2R1A;CLDN7;PPP2R2D;CYCS;HRAS;YWHAH |
| **Endocytosis** | 19/252 | 7.8081616E-04 | 0.005465713 | 2.42 | 17.31 | ARF3;ARF1;RAB5C;ARPC5L;HLA-B;CLTB;HLA-C;HLA-A;ARPC5;HLA-E;RAB11B;SNX3;CDC42;CHMP1B;KIF5B;CHMP2B;VPS35;HRAS;SPG21 |
| **Human cytomegalovirus infection** | 17/225 | 1.4121916E-03 | 0.009520779 | 2.42 | 15.88 | STAT3;HLA-B;HLA-C;HLA-A;PIK3R1;HLA-E;TAPBP;NFKBIA;CCND1;GNG5;GNA11;CYCS;CALM3;CALR;RAC1;CALM1;HRAS |
| **Leukocyte transendothelial migration** | 11/114 | 1.4298606E-03 | 0.009520779 | 3.15 | 20.64 | ITGB1;CDC42;CLDN7;MSN;ACTN4;PIK3R1;RAC1;EZR;ACTB;MYL12A;ACTG1 |
| **Collecting duct acid secretion** | 5/27 | 1.7364647E-03 | 0.011287021 | 6.67 | 42.40 | ATP6V1G1;ATP6V0E2;ATP6V0D1;ATP6V0C;ATP6V1F |
| **Central carbon metabolism in cancer** | 8/70 | 2.1595407E-03 | 0.013164108 | 3.80 | 23.31 | LDHA;PFKL;PKM;PGAM1;IDH2;PDHB;PIK3R1;HRAS |
| **Epithelial cell signaling in Helicobacter pylori infection** | 8/70 | 2.1595407E-03 | 0.013164108 | 3.80 | 23.31 | CDC42;NFKBIA;ATP6V1G1;RAC1;ATP6V0D1;ATP6V0E2;ATP6V0C;ATP6V1F |
| **Ferroptosis** | 6/41 | 2.1699079E-03 | 0.013164108 | 5.04 | 30.89 | GPX4;FTH1;NCOA4;VDAC2;SAT1;FTL |
| **Adherens junction** | 8/71 | 2.3646218E-03 | 0.014033517 | 3.74 | 22.60 | CDC42;RAC1;ACTN4;ACP1;WASF2;ACTB;PTPRF;ACTG1 |
| **Necroptosis** | 13/159 | 2.5031844E-03 | 0.014539773 | 2.63 | 15.75 | HSP90AB1;STAT3;HMGB1;FTH1;CHMP1B;CHMP2B;CAPN2;VDAC2;VDAC1;CAPN1;SLC25A5;SLC25A4;FTL |

| | | | | | | |
|---|---|---|---|---|---|---|
| **Glutathione metabolism** | 7/57 | 2.6733636E-03 | 0.015204756 | 4.12 | 24.39 | GGT6;GPX4;ODC1;IDH2;LAP3;PGD;PRDX6 |
| **HIF-1 signaling pathway** | 10/109 | 3.3572446E-03 | 0.018704648 | 2.98 | 16.95 | LDHA;PFKL;MKNK2;STAT3;ENO1;PIK3R1;ELOC;PDHB;GAPDH;RBX1 |
| **Proteoglycans in cancer** | 15/205 | 3.5621024E-03 | 0.019449079 | 2.33 | 13.15 | ITGB1;DDX5;STAT3;MSN;PIK3R1;ACTB;ACTG1;CDC42;CTTN;CCND1;SDC1;FLNA;RAC1;EZR;HRAS |
| **mRNA surveillance pathway** | 9/98 | 5.2374305E-03 | 0.028035658 | 2.98 | 15.63 | PNN;PPP2R1A;NCBP2;EIF4A3;PPP2R2D;SSU72;SAP18;ETF1;SRRM1 |
| **Histidine metabolism** | 4/22 | 5.4385680E-03 | 0.028552482 | 6.51 | 33.97 | AOC1;ALDH2;CNDP2;ALDH9A1 |
| **Human T-cell leukemia virus 1 infection** | 15/219 | 6.5202616E-03 | 0.033585498 | 2.17 | 10.93 | HLA-B;HLA-C;HLA-A;PIK3R1;HLA-E;NFKBIA;CCND1;CDC26;CANX;VDAC2;VDAC1;CALR;SLC25A5;HRAS;SLC25A4 |
| **Focal adhesion** | 14/201 | 7.3044388E-03 | 0.036927996 | 2.21 | 10.87 | ITGB1;VEGFB;ACTN4;PIK3R1;ACTB;MYL12A;ACTG1;CDC42;CCND1;SPP1;CAPN2;FLNA;RAC1;HRAS |
| **Legionellosis** | 6/57 | 1.1227037E-02 | 0.055726929 | 3.45 | 15.50 | EEF1A1;NFKBIA;ARF1;VCP;RAB1B;CYCS |
| **Arrhythmogenic right ventricular cardiomyopathy** | 7/77 | 1.3823270E-02 | 0.06738844 | 2.94 | 12.57 | ITGB1;DSP;SGCB;LMNA;ATP2A2;ACTB;ACTG1 |
| **Synaptic vesicle cycle** | 7/78 | 1.4766051E-02 | 0.070034178 | 2.90 | 12.21 | ATP6V1G1;CLTB;ATP6V0D1;ATP6V0E2;ATP6V0C;VAMP2;ATP6V1F |
| **Human papillomavirus infection** | 19/331 | 1.4879056E-02 | 0.070034178 | 1.80 | 7.57 | ITGB1;ATP6V1G1;HLA-B;HLA-C;CHD4;HLA-A;PIK3R1;HLA-E;CDC42;PKM;CCND1;PPP2R1A;PPP2R2D;SPP1;ATP6V0D1;ATP6V0E2;HRAS;ATP6V0C;ATP6V1F |
| **Yersinia infection** | 10/137 | 1.5960264E-02 | 0.073850034 | 2.32 | 9.58 | NFKBIA;ITGB1;CDC42;ARPC5L;PIK3R1;ARPC5;RAC1;WASF2;ACTB;ACTG1 |
| **Epstein-Barr virus infection** | 13/202 | 1.7474811E-02 | 0.078611799 | 2.03 | 8.20 | PSMD12;STAT3;HLA-B;HLA-C;HLA-A;PIK3R1;HLA-E;TAPBP;NFKBIA;CCND1;CYCS;CALR;RAC1 |
| **Neurotrophin signaling pathway** | 9/119 | 1.7565274E-02 | 0.078611799 | 2.41 | 9.72 | NFKBIA;YWHAE;CDC42;BEX3;CALM3;PIK3R1;RAC1;CALM1;HRAS |
| **AMPK signaling pathway** | 9/120 | 1.8452336E-02 | 0.081249803 | 2.38 | 9.52 | PFKL;CCND1;PPP2R1A;PPP2R2D;PIK3R1;ADIPOR1;PCK1;EEF2;RAB11B |
| **Peroxisome** | 7/82 | 1.8996904E-02 | 0.082319917 | 2.74 | 10.86 | PRDX5;SCP2;EPHX2;ECH1;CAT;IDH2;ACAA1 |
| **Spinocerebellar ataxia** | 10/143 | 2.0894058E-02 | 0.089126216 | 2.21 | 8.55 | PSMD12;XBP1;PPIF;VDAC2;CYCS;ATP2A2;VDAC1;PIK3R1;SLC25A5;SLC25A4 |
| **Glucagon signaling pathway** | 8/107 | 2.5923458E-02 | 0.108878523 | 2.37 | 8.67 | LDHA;PFKL;PKM;PGAM1;CALM3;PDHB;PCK1;CALM1 |

| | | | | | | |
|---|---|---|---|---|---|---|
| **Renal cell carcinoma** | 6/69 | 2.6668612E-02 | 0.110311079 | 2.79 | 10.13 | CDC42;ELOC;RAC1;PIK3R1;HRAS;RBX1 |
| **Hypertrophic cardiomyopathy** | 7/90 | 2.9867694E-02 | 0.121699708 | 2.48 | 8.69 | ITGB1;SGCB;TPM3;LMNA;ATP2A2;ACTB;ACTG1 |
| **Thyroid cancer** | 4/37 | 3.3282966E-02 | 0.13362132 | 3.55 | 12.08 | CCND1;TPM3;NCOA4;HRAS |
| **Allograft rejection** | 4/38 | 3.6256640E-02 | 0.143450186 | 3.45 | 11.43 | HLA-B;HLA-C;HLA-A;HLA-E |
| **Autophagy** | 9/137 | 3.8900644E-02 | 0.147498275 | 2.07 | 6.71 | VAMP8;GABARAPL2;GABARAPL1;LAMP1;PIK3R1;HMGB1;CTSD;HRAS;CTSB |
| **Estrogen signaling pathway** | 9/137 | 3.8900644E-02 | 0.147498275 | 2.07 | 6.71 | HSP90AB1;CALM3;PIK3R1;KRT10;FKBP4;CALM1;CTSD;HRAS;FKBP5 |
| **Insulin signaling pathway** | 9/137 | 3.8900644E-02 | 0.147498275 | 2.07 | 6.71 | PRKAR1A;MKNK2;CALM3;PIK3R1;PCK1;CALM1;HRAS;PTPRF;RHOQ |
| **Gastric acid secretion** | 6/76 | 4.0184693E-02 | 0.148822395 | 2.51 | 8.08 | CALM3;ATP1B1;EZR;CALM1;ACTB;ACTG1 |
| **Dilated cardiomyopathy** | 7/96 | 4.0340136E-02 | 0.148822395 | 2.31 | 7.41 | ITGB1;SGCB;TPM3;LMNA;ATP2A2;ACTB;ACTG1 |
| **Ubiquitin mediated proteolysis** | 9/140 | 4.3667846E-02 | 0.15895096 | 2.02 | 6.32 | DDB1;UBE2I;CDC26;UBE2N;STUB1;ELOC;RNF7;RBX1;UBE2M |
| **Rap1 signaling pathway** | 12/210 | 4.7729512E-02 | 0.169379679 | 1.78 | 5.42 | ITGB1;CDC42;VEGFB;CALM3;PIK3R1;RAC1;PFN1;CALM1;HRAS;ACTB;PFN2;ACTG1 |
| **Thyroid hormone signaling pathway** | 8/121 | 4.8259852E-02 | 0.169379679 | 2.08 | 6.30 | PFKL;CCND1;ATP2A2;PIK3R1;ATP1B1;HRAS;ACTB;ACTG1 |
| **AGE-RAGE signaling pathway in diabetic complications** | 7/100 | 4.8505039E-02 | 0.169379679 | 2.21 | 6.68 | CDC42;CCND1;STAT3;VEGFB;PIK3R1;RAC1;HRAS |
| **Graft-versus-host disease** | 4/42 | 4.9635071E-02 | 0.169379679 | 3.08 | 9.26 | HLA-B;HLA-C;HLA-A;HLA-E |
| **Tryptophan metabolism** | 4/42 | 4.9635071E-02 | 0.169379679 | 3.08 | 9.26 | AOC1;ALDH2;CAT;ALDH9A1 |
| **cGMP-PKG signaling pathway** | 10/167 | 5.1835339E-02 | 0.174704292 | 1.87 | 5.54 | GNA11;PPIF;VDAC2;ATP2A2;VDAC1;CALM3;ATP1B1;CALM1;SLC25A5;SLC25A4 |
| **Fatty acid degradation** | 4/43 | 5.3349662E-02 | 0.175475394 | 3.00 | 8.80 | ACADVL;ALDH2;ACAA1;ALDH9A1 |
| **Type I diabetes mellitus** | 4/43 | 5.3349662E-02 | 0.175475394 | 3.00 | 8.80 | HLA-B;HLA-C;HLA-A;HLA-E |
| **Lipid and atherosclerosis** | 12/215 | 5.5168304E-02 | 0.179296987 | 1.74 | 5.03 | NFKBIA;CDC42;XBP1;HSP90AB1;DDIT3;STAT3;CYCS;CALM3;PIK3R1;RAC1;CALM1;HRAS |
| **Vasopressin-regulated water reabsorption** | 4/44 | 5.7210989E-02 | 0.183748234 | 2.93 | 8.38 | RAB5C;DYNLL1;VAMP2;RAB11B |

| | | | | | | |
|---|---|---|---|---|---|---|
| **Biosynthesis of unsaturated fatty acids** | 3/27 | 5.8837991E-02 | 0.186776414 | 3.66 | 10.36 | SCP2;HSD17B12;ACAA1 |
| **Lysosome** | 8/128 | 6.3059181E-02 | 0.197875362 | 1.96 | 5.41 | SCARB2;LAMP1;M6PR;CLTB;ATP6V0D1;CTSD;ATP6V0C;CTSB |
| **Oocyte meiosis** | 8/129 | 6.5380449E-02 | 0.202827983 | 1.94 | 5.29 | YWHAE;PPP2R1A;YWHAB;CDC26;CALM3;CALM1;RBX1;YWHAH |
| **FoxO signaling pathway** | 8/131 | 7.0179934E-02 | 0.215271032 | 1.91 | 5.07 | GABARAPL2;GABARAPL1;CCND1;CAT;STAT3;PIK3R1;PCK1;HRAS |
| **Valine, leucine and isoleucine degradation** | 4/48 | 7.4099611E-02 | 0.22312225 | 2.66 | 6.93 | ALDH2;IVD;ACAA1;ALDH9A1 |
| **Mitophagy** | 5/68 | 7.4813066E-02 | 0.22312225 | 2.32 | 6.03 | GABARAPL2;GABARAPL1;PINK1;CITED2;HRAS |
| **Glyoxylate and dicarboxylate metabolism** | 3/30 | 7.6008678E-02 | 0.22312225 | 3.25 | 8.38 | GCSH;MDH2;CAT |
| **beta-Alanine metabolism** | 3/30 | 7.6008678E-02 | 0.22312225 | 3.25 | 8.38 | ALDH2;CNDP2;ALDH9A1 |
| **NOD-like receptor signaling pathway** | 10/181 | 7.9521090E-02 | 0.230949547 | 1.72 | 4.35 | NFKBIA;YWHAE;GABARAPL2;GABARAPL1;HSP90AB1;VDAC2;VDAC1;TXN;TXN2;CTSB |
| **N-Glycan biosynthesis** | 4/50 | 8.3387333E-02 | 0.239628861 | 2.55 | 6.32 | GANAB;B4GALT1;RPN1;MGAT4B |
| **Apelin signaling pathway** | 8/137 | 8.5837920E-02 | 0.2439313 | 1.82 | 4.47 | GABARAPL2;GABARAPL1;CCND1;GNG5;SPP1;CALM3;CALM1;HRAS |
| **Thiamine metabolism** | 2/15 | 8.6671561E-02 | 0.2439313 | 4.50 | 11.00 | NTPCR;ACP1 |
| **Non-small cell lung cancer** | 5/72 | 9.0452620E-02 | 0.251975156 | 2.19 | 5.25 | CCND1;KIF5B;STAT3;PIK3R1;HRAS |
| **Autoimmune thyroid disease** | 4/53 | 9.8326605E-02 | 0.269682747 | 2.39 | 5.54 | HLA-B;HLA-C;HLA-A;HLA-E |
| **Neutrophil extracellular trap formation** | 10/189 | 9.8784889E-02 | 0.269682747 | 1.64 | 3.79 | PPIF;VDAC2;VDAC1;PIK3R1;HMGB1;RAC1;SLC25A5;SLC25A4;ACTB;ACTG1 |
| **Sphingolipid signaling pathway** | 7/119 | 1.0085510E-01 | 0.270909926 | 1.83 | 4.20 | PPP2R1A;PPP2R2D;PIK3R1;RAC1;CTSD;HRAS;CERS2 |
| **Glioma** | 5/75 | 1.0320177E-01 | 0.270909926 | 2.09 | 4.75 | CCND1;CALM3;PIK3R1;CALM1;HRAS |
| **Fc gamma R-mediated phagocytosis** | 6/97 | 1.0320378E-01 | 0.270909926 | 1.93 | 4.39 | CDC42;ARPC5L;PIK3R1;ARPC5;RAC1;WASF2 |
| **Prostate cancer** | 6/97 | 1.0320378E-01 | 0.270909926 | 1.93 | 4.39 | NFKBIA;HSP90AB1;CCND1;SPINT1;PIK3R1;HRAS |
| **Chronic myeloid leukemia** | 5/76 | 1.0763960E-01 | 0.277222742 | 2.06 | 4.60 | NFKBIA;CCND1;CTBP1;PIK3R1;HRAS |
| **Pancreatic cancer** | 5/76 | 1.0763960E-01 | 0.277222742 | 2.06 | 4.60 | CDC42;CCND1;STAT3;RAC1;PIK3R1 |

| | | | | | | |
|---|---|---|---|---|---|---|
| **Influenza A** | 9/172 | 1.1901748E-01 | 0.3018244 | 1.62 | 3.45 | NFKBIA;CYCS;VDAC1;PIK3R1;SLC25A5;SLC25A4;ACTB;ACTG1;RAB11B |
| **Cell adhesion molecules** | 8/148 | 1.1940306E-01 | 0.3018244 | 1.67 | 3.56 | ITGB1;CLDN7;HLA-B;HLA-C;SDC1;HLA-A;PTPRF;HLA-E |
| **Chagas disease** | 6/102 | 1.2313191E-01 | 0.305828958 | 1.83 | 3.83 | NFKBIA;PPP2R1A;GNA11;PPP2R2D;PIK3R1;CALR |
| **Aldosterone-regulated sodium reabsorption** | 3/37 | 1.2322779E-01 | 0.305828958 | 2.58 | 5.40 | HSD11B2;PIK3R1;ATP1B1 |
| **Adrenergic signaling in cardiomyocytes** | 8/150 | 1.2616327E-01 | 0.310293437 | 1.65 | 3.42 | PPP1R1A;PPP2R1A;TPM3;PPP2R2D;ATP2A2;CALM3;ATP1B1;CALM1 |
| **B cell receptor signaling pathway** | 5/81 | 1.3117650E-01 | 0.31786855 | 1.93 | 3.91 | NFKBIA;CD81;RAC1;PIK3R1;HRAS |
| **VEGF signaling pathway** | 4/59 | 1.3157196E-01 | 0.31786855 | 2.13 | 4.32 | CDC42;RAC1;PIK3R1;HRAS |
| **Various types of N-glycan biosynthesis** | 3/39 | 1.3829112E-01 | 0.331170829 | 2.44 | 4.82 | B4GALT1;RPN1;MGAT4B |
| **Glycerolipid metabolism** | 4/61 | 1.4356280E-01 | 0.338907159 | 2.05 | 3.99 | ALDH2;MGLL;AGPAT3;ALDH9A1 |
| **Tuberculosis** | 9/180 | 1.4489408E-01 | 0.338907159 | 1.54 | 2.98 | CD74;RAB5C;LAMP1;CYCS;CALM3;ATP6V0D1;CALM1;CTSD;ATP6V0C |
| **Natural killer cell mediated cytotoxicity** | 7/131 | 1.4524593E-01 | 0.338907159 | 1.65 | 3.19 | HLA-B;HLA-C;HLA-A;PIK3R1;RAC1;HRAS;HLA-E |
| **Insulin resistance** | 6/108 | 1.4932380E-01 | 0.343730202 | 1.72 | 3.27 | NFKBIA;NR1H2;STAT3;PIK3R1;PCK1;PTPRF |
| **Coronavirus disease** | 11/232 | 1.4983111E-01 | 0.343730202 | 1.46 | 2.77 | RPL4;NFKBIA;RPL36AL;RPLP0;STAT3;RPL14;RPS27L;RPS20;PIK3R1;RPS10;RPS13 |
| **Pantothenate and CoA biosynthesis** | 2/21 | 1.5256526E-01 | 0.347085963 | 3.08 | 5.78 | ALDH2;PPCS |
| **Colorectal cancer** | 5/86 | 1.5680954E-01 | 0.35379343 | 1.81 | 3.35 | CCND1;CYCS;RAC1;PIK3R1;HRAS |
| **GnRH secretion** | 4/64 | 1.6230974E-01 | 0.363201296 | 1.95 | 3.55 | GNA11;SPP1;PIK3R1;HRAS |
| **Terpenoid backbone biosynthesis** | 2/22 | 1.6432672E-01 | 0.364725164 | 2.92 | 5.28 | ICMT;PCYOX1 |
| **Gap junction** | 5/88 | 1.6759864E-01 | 0.368987319 | 1.76 | 3.15 | TUBA1C;TUBA1B;GNA11;TUBB4B;HRAS |
| **Hippo signaling pathway** | 8/163 | 1.7468497E-01 | 0.381511979 | 1.51 | 2.64 | YWHAE;CCND1;PPP2R1A;YWHAB;PPP2R2D;ACTB;ACTG1;YWHAH |
| **Proximal tubule bicarbonate reclamation** | 2/23 | 1.7624058E-01 | 0.381854593 | 2.78 | 4.83 | ATP1B1;PCK1 |
| **Measles** | 7/139 | 1.7920103E-01 | 0.38460972 | 1.55 | 2.67 | NFKBIA;CCND1;EIF2AK1;STAT3;MSN;CYCS;PIK3R1 |

| | | | | | | |
|---|---|---|---|---|---|---|
| **Acute myeloid leukemia** | 4/67 | 1.8188451E-01 | 0.38460972 | 1.86 | 3.16 | CCND1;STAT3;PIK3R1;HRAS |
| **Long-term potentiation** | 4/67 | 1.8188451E-01 | 0.38460972 | 1.86 | 3.16 | PPP1R1A;CALM3;CALM1;HRAS |
| **Vitamin B6 metabolism** | 1/6 | 1.8314749E-01 | 0.38460972 | 5.84 | 9.91 | PNPO |
| **Small cell lung cancer** | 5/92 | 1.9000725E-01 | 0.395890656 | 1.68 | 2.79 | ITGB1;NFKBIA;CCND1;CYCS;PIK3R1 |
| **Proteasome** | 3/46 | 1.9520438E-01 | 0.395890656 | 2.04 | 3.33 | PSMD12;POMP;PSMB8 |
| **Adipocytokine signaling pathway** | 4/69 | 1.9534584E-01 | 0.395890656 | 1.80 | 2.94 | NFKBIA;STAT3;ADIPOR1;PCK1 |
| **GnRH signaling pathway** | 5/93 | 1.9577010E-01 | 0.395890656 | 1.66 | 2.71 | CDC42;GNA11;CALM3;CALM1;HRAS |
| **Rheumatoid arthritis** | 5/93 | 1.9577010E-01 | 0.395890656 | 1.66 | 2.71 | ATP6V1G1;ATP6V0D1;ATP6V0E2;ATP6V0C;ATP6V1F |
| **PI3K-Akt signaling pathway** | 15/354 | 1.9944243E-01 | 0.400351358 | 1.30 | 2.09 | ITGB1;YWHAE;HSP90AB1;YWHAB;VEGFB;PIK3R1;CCND1;GNG5;PPP2R1A;PPP2R2D;SPP1;RAC1;PCK1;HRAS;YWHAH |
| **Prolactin signaling pathway** | 4/70 | 2.0218891E-01 | 0.402901983 | 1.77 | 2.83 | CCND1;STAT3;PIK3R1;HRAS |
| **Nucleotide excision repair** | 3/47 | 2.0376396E-01 | 0.403098273 | 1.99 | 3.17 | DDB1;CETN2;RBX1 |
| **Cell cycle** | 6/124 | 2.2947838E-01 | 0.445753365 | 1.49 | 2.19 | YWHAE;CCND1;YWHAB;CDC26;RBX1;YWHAH |
| **Platelet activation** | 6/124 | 2.2947838E-01 | 0.445753365 | 1.49 | 2.19 | ITGB1;VAMP8;PIK3R1;ACTB;MYL12A;ACTG1 |
| **PPAR signaling pathway** | 4/74 | 2.3022427E-01 | 0.445753365 | 1.67 | 2.45 | SCP2;DBI;ACAA1;PCK1 |
| **Riboflavin metabolism** | 1/8 | 2.3642410E-01 | 0.453098822 | 4.17 | 6.02 | ACP1 |
| **Phototransduction** | 2/28 | 2.3733748E-01 | 0.453098822 | 2.25 | 3.23 | CALM3;CALM1 |
| **Oxytocin signaling pathway** | 7/154 | 2.5051336E-01 | 0.474931575 | 1.39 | 1.93 | CCND1;CALM3;EEF2;CALM1;HRAS;ACTB;ACTG1 |
| **Endocrine and other factor-regulated calcium reabsorption** | 3/53 | 2.5665125E-01 | 0.483212355 | 1.75 | 2.38 | CLTB;PTH1R;ATP1B1 |
| **Ascorbate and aldarate metabolism** | 2/30 | 2.6213679E-01 | 0.484444712 | 2.09 | 2.79 | ALDH2;ALDH9A1 |
| **Pentose phosphate pathway** | 2/30 | 2.6213679E-01 | 0.484444712 | 2.09 | 2.79 | PFKL;PGD |

| | | | | | | |
|---|---|---|---|---|---|---|
| **C-type lectin receptor signaling pathway** | 5/104 | 2.6262937E-01 | 0.484444712 | 1.48 | 1.97 | NFKBIA;CALM3;PIK3R1;CALM1;HRAS |
| **RNA degradation** | 4/79 | 2.6648551E-01 | 0.488258685 | 1.56 | 2.06 | PFKL;ENO1;TOB1;LSM4 |
| **Dopaminergic synapse** | 6/132 | 2.7383725E-01 | 0.490531844 | 1.39 | 1.80 | GNG5;PPP2R1A;KIF5B;PPP2R2D;CALM3;CALM1 |
| **Circadian rhythm** | 2/31 | 2.7454833E-01 | 0.490531844 | 2.01 | 2.60 | BHLHE40;RBX1 |
| **Galactose metabolism** | 2/31 | 2.7454833E-01 | 0.490531844 | 2.01 | 2.60 | PFKL;B4GALT1 |
| **Calcium signaling pathway** | 10/240 | 2.7491345E-01 | 0.490531844 | 1.27 | 1.64 | GNA11;VEGFB;PPIF;VDAC2;ATP2A2;VDAC1;CALM3;CALM1;SLC25A5;SLC25A4 |
| **Pyrimidine metabolism** | 3/56 | 2.8377098E-01 | 0.503048553 | 1.65 | 2.08 | CMPK1;NME4;NME1 |
| **Hepatitis B** | 7/162 | 2.9161536E-01 | 0.513619315 | 1.32 | 1.63 | NFKBIA;DDB1;YWHAB;STAT3;CYCS;PIK3R1;HRAS |
| **Fructose and mannose metabolism** | 2/33 | 2.9932607E-01 | 0.520484179 | 1.88 | 2.27 | PFKL;TPI1 |
| **SNARE interactions in vesicular transport** | 2/33 | 2.9932607E-01 | 0.520484179 | 1.88 | 2.27 | VAMP8;VAMP2 |
| **Endometrial cancer** | 3/58 | 3.0198448E-01 | 0.521783309 | 1.59 | 1.91 | CCND1;PIK3R1;HRAS |
| **Taurine and hypotaurine metabolism** | 1/11 | 3.0990406E-01 | 0.529843649 | 2.92 | 3.42 | GGT6 |
| **Pathways in cancer** | 20/531 | 3.1053108E-01 | 0.529843649 | 1.15 | 1.34 | ITGB1;HSP90AB1;CTBP1;TPM3;STAT3;NCOA4;VEGFB;PIK3R1;RBX1;CDC42;NFKBIA;CCND1;GNG5;GNA11;CYCS;CALM3;ELOC;RAC1;CALM1;HRAS |
| **Toxoplasmosis** | 5/112 | 3.1411157E-01 | 0.532623975 | 1.37 | 1.58 | NFKBIA;ITGB1;STAT3;PPIF;CYCS |
| **Long-term depression** | 3/60 | 3.2025053E-01 | 0.536370515 | 1.54 | 1.75 | PPP2R1A;GNA11;HRAS |
| **Mineral absorption** | 3/60 | 3.2025053E-01 | 0.536370515 | 1.54 | 1.75 | FTH1;ATP1B1;FTL |
| **PD-L1 expression and PD-1 checkpoint pathway in cancer** | 4/89 | 3.4139194E-01 | 0.568292679 | 1.37 | 1.48 | NFKBIA;STAT3;PIK3R1;HRAS |
| **Non-homologous end-joining** | 1/13 | 3.5492444E-01 | 0.58723862 | 2.43 | 2.52 | XRCC6 |
| **Ras signaling pathway** | 9/232 | 3.6335186E-01 | 0.59756059 | 1.18 | 1.19 | CDC42;RAB5C;GNG5;VEGFB;CALM3;PIK3R1;RAC1;CALM1;HRAS |

| | | | | | | |
|---|---|---|---|---|---|---|
| **Salivary secretion** | 4/93 | 3.7166532E-01 | 0.607572643 | 1.31 | 1.30 | CALM3;ATP1B1;CALM1;VAMP2 |
| **Glycine, serine and threonine metabolism** | 2/40 | 3.8438819E-01 | 0.624630801 | 1.54 | 1.47 | GCSH;PGAM1 |
| **Fc epsilon RI signaling pathway** | 3/68 | 3.9301141E-01 | 0.634864578 | 1.35 | 1.26 | RAC1;PIK3R1;HRAS |
| **Bladder cancer** | 2/41 | 3.9620610E-01 | 0.636260377 | 1.50 | 1.39 | CCND1;HRAS |
| **Renin secretion** | 3/69 | 4.0199620E-01 | 0.639357598 | 1.33 | 1.21 | CALM3;CALM1;CTSB |
| **mTOR signaling pathway** | 6/154 | 4.0281871E-01 | 0.639357598 | 1.18 | 1.08 | ATP6V1G1;PIK3R1;LAMTOR1;HRAS;ATP6V1F;LAMTOR5 |
| **Aldosterone synthesis and secretion** | 4/98 | 4.0934037E-01 | 0.642240925 | 1.24 | 1.11 | GNA11;CALM3;ATP1B1;CALM1 |
| **Choline metabolism in cancer** | 4/98 | 4.0934037E-01 | 0.642240925 | 1.24 | 1.11 | PIK3R1;RAC1;HRAS;WASF2 |
| **Purine metabolism** | 5/129 | 4.2603247E-01 | 0.65512287 | 1.18 | 1.00 | PKM;GUK1;NME4;NTPCR;NME1 |
| **Relaxin signaling pathway** | 5/129 | 4.2603247E-01 | 0.65512287 | 1.18 | 1.00 | NFKBIA;GNG5;VEGFB;PIK3R1;HRAS |
| **Melanoma** | 3/72 | 4.2870693E-01 | 0.65512287 | 1.27 | 1.08 | CCND1;PIK3R1;HRAS |
| **Nitrogen metabolism** | 1/17 | 4.3635730E-01 | 0.65512287 | 1.82 | 1.51 | CA12 |
| **Primary bile acid biosynthesis** | 1/17 | 4.3635730E-01 | 0.65512287 | 1.82 | 1.51 | SCP2 |
| **Selenocompound metabolism** | 1/17 | 4.3635730E-01 | 0.65512287 | 1.82 | 1.51 | SEPHS2 |
| **p53 signaling pathway** | 3/73 | 4.3751765E-01 | 0.65512287 | 1.25 | 1.03 | CCND1;EI24;CYCS |
| **Amoebiasis** | 4/102 | 4.3914830E-01 | 0.65512287 | 1.19 | 0.98 | RAB5C;GNA11;ACTN4;PIK3R1 |
| **Longevity regulating pathway** | 4/102 | 4.3914830E-01 | 0.65512287 | 1.19 | 0.98 | CAT;PIK3R1;ADIPOR1;HRAS |
| **Chemokine signaling pathway** | 7/192 | 4.5331067E-01 | 0.660689801 | 1.10 | 0.87 | NFKBIA;CDC42;GNG5;STAT3;PIK3R1;RAC1;HRAS |
| **Type II diabetes mellitus** | 2/46 | 4.5359775E-01 | 0.660689801 | 1.33 | 1.05 | PKM;PIK3R1 |
| **T cell receptor signaling pathway** | 4/104 | 4.5389113E-01 | 0.660689801 | 1.17 | 0.92 | NFKBIA;CDC42;PIK3R1;HRAS |
| **Toll-like receptor signaling pathway** | 4/104 | 4.5389113E-01 | 0.660689801 | 1.17 | 0.92 | NFKBIA;SPP1;PIK3R1;RAC1 |
| **Thyroid hormone synthesis** | 3/75 | 4.5498052E-01 | 0.660689801 | 1.22 | 0.96 | CANX;ATP1B1;PDIA4 |

| | | | | | | |
|---|---|---|---|---|---|---|
| **Pertussis** | 3/76 | 4.6362678E-01 | 0.667702643 | 1.20 | 0.92 | ITGB1;CALM3;CALM1 |
| **Carbohydrate digestion and absorption** | 2/47 | 4.6470147E-01 | 0.667702643 | 1.30 | 0.99 | PIK3R1;ATP1B1 |
| **Parathyroid hormone synthesis, secretion and action** | 4/106 | 4.6850446E-01 | 0.669642501 | 1.14 | 0.87 | JUND;NACA;GNA11;PTH1R |
| **Leishmaniasis** | 3/77 | 4.7221255E-01 | 0.671427226 | 1.18 | 0.89 | ITGB1;EEF1A1;NFKBIA |
| **Hepatocellular carcinoma** | 6/168 | 4.8492533E-01 | 0.685930647 | 1.08 | 0.78 | CCND1;PIK3R1;HRAS;ACTB;ACTG1;SMARCA4 |
| **Cholesterol metabolism** | 2/50 | 4.9719309E-01 | 0.692518946 | 1.22 | 0.85 | VDAC2;VDAC1 |
| **Cysteine and methionine metabolism** | 2/50 | 4.9719309E-01 | 0.692518946 | 1.22 | 0.85 | LDHA;MDH2 |
| **Malaria** | 2/50 | 4.9719309E-01 | 0.692518946 | 1.22 | 0.85 | CD81;SDC1 |
| **Cholinergic synapse** | 4/113 | 5.1842181E-01 | 0.718422096 | 1.07 | 0.70 | GNG5;GNA11;PIK3R1;HRAS |
| **Mannose type O-glycan biosynthesis** | 1/23 | 5.3966765E-01 | 0.74408721 | 1.33 | 0.82 | B4GALT1 |
| **Insulin secretion** | 3/86 | 5.4638186E-01 | 0.74864456 | 1.05 | 0.64 | GNA11;ATP1B1;VAMP2 |
| **Regulation of lipolysis in adipocytes** | 2/55 | 5.4845755E-01 | 0.74864456 | 1.10 | 0.66 | PIK3R1;MGLL |
| **Growth hormone synthesis, secretion and action** | 4/119 | 5.5938661E-01 | 0.757383913 | 1.01 | 0.59 | GNA11;STAT3;PIK3R1;HRAS |
| **ECM-receptor interaction** | 3/88 | 5.6202224E-01 | 0.757383913 | 1.03 | 0.59 | ITGB1;SPP1;SDC1 |
| **Axon guidance** | 6/182 | 5.6318291E-01 | 0.757383913 | 0.99 | 0.57 | ITGB1;CDC42;PIK3R1;RAC1;HRAS;MYL12A |
| **alpha-Linolenic acid metabolism** | 1/25 | 5.6971645E-01 | 0.758698489 | 1.22 | 0.68 | ACAA1 |
| **GABAergic synapse** | 3/89 | 5.6971865E-01 | 0.758698489 | 1.02 | 0.57 | GABARAPL2;GABARAPL1;GNG5 |
| **cAMP signaling pathway** | 7/216 | 5.7765938E-01 | 0.765538882 | 0.98 | 0.54 | NFKBIA;ATP2A2;CALM3;PIK3R1;RAC1;CALM1;ATP1B1 |
| **Notch signaling pathway** | 2/59 | 5.8675244E-01 | 0.773832933 | 1.02 | 0.55 | APH1A;CTBP1 |
| **Fatty acid elongation** | 1/27 | 5.9780653E-01 | 0.784621067 | 1.12 | 0.58 | HSD17B12 |
| **Steroid hormone biosynthesis** | 2/61 | 6.0497783E-01 | 0.788997229 | 0.99 | 0.50 | HSD11B2;HSD17B12 |
| **IL-17 signaling pathway** | 3/94 | 6.0692095E-01 | 0.788997229 | 0.96 | 0.48 | NFKBIA;HSP90AB1;JUND |

| | | | | | | |
|---|---|---|---|---|---|---|
| **Osteoclast differentiation** | 4/127 | 6.1092785E-01 | 0.790442189 | 0.95 | 0.47 | NFKBIA;JUND;PIK3R1;RAC1 |
| **Lysine degradation** | 2/63 | 6.2258762E-01 | 0.801382959 | 0.96 | 0.45 | ALDH2;ALDH9A1 |
| **Circadian entrainment** | 3/97 | 6.2819030E-01 | 0.801382959 | 0.93 | 0.43 | GNG5;CALM3;CALM1 |
| **Phosphatidylinositol signaling system** | 3/97 | 6.2819030E-01 | 0.801382959 | 0.93 | 0.43 | CALM3;PIK3R1;CALM1 |
| **Inflammatory mediator regulation of TRP channels** | 3/98 | 6.3510135E-01 | 0.806431016 | 0.92 | 0.42 | CALM3;PIK3R1;CALM1 |
| **RNA polymerase** | 1/31 | 6.4861224E-01 | 0.81604762 | 0.97 | 0.42 | POLR2C |
| **Progesterone-mediated oocyte maturation** | 3/100 | 6.4865324E-01 | 0.81604762 | 0.90 | 0.39 | HSP90AB1;CDC26;PIK3R1 |
| **Melanogenesis** | 3/101 | 6.5529364E-01 | 0.820620011 | 0.89 | 0.38 | CALM3;CALM1;HRAS |
| **Pancreatic secretion** | 3/102 | 6.6184349E-01 | 0.825037773 | 0.88 | 0.36 | ATP2A2;RAC1;ATP1B1 |
| **Base excision repair** | 1/33 | 6.7155855E-01 | 0.829822632 | 0.91 | 0.36 | HMGB1 |
| **Amphetamine addiction** | 2/69 | 6.7176118E-01 | 0.829822632 | 0.87 | 0.35 | CALM3;CALM1 |
| **Propanoate metabolism** | 1/34 | 6.8246433E-01 | 0.835630511 | 0.88 | 0.34 | LDHA |
| **Chemical carcinogenesis** | 7/239 | 6.8258463E-01 | 0.835630511 | 0.88 | 0.34 | PAQR7;HSP90AB1;CCND1;EPHX2;STAT3;PIK3R1;HRAS |
| **Th17 cell differentiation** | 3/107 | 6.9323471E-01 | 0.844659804 | 0.84 | 0.31 | NFKBIA;HSP90AB1;STAT3 |
| **Drug metabolism** | 3/108 | 6.9924218E-01 | 0.844659804 | 0.83 | 0.30 | CMPK1;NME4;NME1 |
| **Ribosome biogenesis in eukaryotes** | 3/108 | 6.9924218E-01 | 0.844659804 | 0.83 | 0.30 | POP5;SBDS;NOP10 |
| **Alanine, aspartate and glutamate metabolism** | 1/37 | 7.1305960E-01 | 0.857556254 | 0.81 | 0.27 | ALDH4A1 |
| **Phospholipase D signaling pathway** | 4/148 | 7.2725848E-01 | 0.870796336 | 0.81 | 0.26 | ARF1;PIK3R1;HRAS;AGPAT3 |
| **MAPK signaling pathway** | 8/294 | 7.6248273E-01 | 0.908854074 | 0.81 | 0.22 | CDC42;JUND;DDIT3;MKNK2;VEGFB;FLNA;RAC1;HRAS |
| **Porphyrin and chlorophyll metabolism** | 1/43 | 7.6570123E-01 | 0.908854074 | 0.69 | 0.19 | UROS |
| **Herpes simplex virus 1 infection** | 14/498 | 7.7248816E-01 | 0.911178804 | 0.84 | 0.22 | CD74;EIF2AK1;HLA-B;HLA-C;HLA-A;PIK3R1;HLA-E;TAPBP;NFKBIA;SRSF3;CYCS;CALR;SRSF8;SRSF9 |

| | | | | | | |
|---|---|---|---|---|---|---|
| **ErbB signaling pathway** | 2/85 | 7.7770477E-01 | 0.911178804 | 0.70 | 0.18 | PIK3R1;HRAS |
| **Basal transcription factors** | 1/45 | 7.8101040E-01 | 0.911178804 | 0.66 | 0.16 | TAF10 |
| **Glycosphingolipid biosynthesis** | 1/45 | 7.8101040E-01 | 0.911178804 | 0.66 | 0.16 | B4GALT1 |
| **JAK-STAT signaling pathway** | 4/162 | 7.8909558E-01 | 0.916694018 | 0.74 | 0.17 | CCND1;STAT3;PIK3R1;HRAS |
| **Other types of O-glycan biosynthesis** | 1/47 | 7.9532067E-01 | 0.919009292 | 0.63 | 0.15 | B4GALT1 |
| **Amino sugar and nucleotide sugar metabolism** | 1/48 | 8.0212172E-01 | 0.919009292 | 0.62 | 0.14 | CYB5R3 |
| **Intestinal immune network for IgA production** | 1/48 | 8.0212172E-01 | 0.919009292 | 0.62 | 0.14 | PIGR |
| **Wnt signaling pathway** | 4/166 | 8.0455392E-01 | 0.919009292 | 0.72 | 0.16 | CCND1;CTBP1;RAC1;RBX1 |
| **Sphingolipid metabolism** | 1/49 | 8.0869711E-01 | 0.919892964 | 0.61 | 0.13 | CERS2 |
| **Vascular smooth muscle contraction** | 3/133 | 8.2163351E-01 | 0.928547324 | 0.67 | 0.13 | GNA11;CALM3;CALM1 |
| **TGF-beta signaling pathway** | 2/94 | 8.2310788E-01 | 0.928547324 | 0.63 | 0.12 | PPP2R1A;RBX1 |
| **Glycosaminoglycan biosynthesis** | 1/53 | 8.3288798E-01 | 0.935713658 | 0.56 | 0.10 | B4GALT1 |
| **Hedgehog signaling pathway** | 1/56 | 8.4900432E-01 | 0.949910576 | 0.53 | 0.09 | CCND1 |
| **Signaling pathways regulating pluripotency of stem cells** | 3/143 | 8.5716533E-01 | 0.955127087 | 0.62 | 0.10 | STAT3;PIK3R1;HRAS |
| **NF-kappa B signaling pathway** | 2/104 | 8.6364213E-01 | 0.955665727 | 0.57 | 0.08 | NFKBIA;UBE2I |
| **Alcoholism** | 4/186 | 8.6853553E-01 | 0.955665727 | 0.64 | 0.09 | GNG5;CALM3;CALM1;HRAS |
| **Breast cancer** | 3/147 | 8.6954826E-01 | 0.955665727 | 0.61 | 0.08 | CCND1;PIK3R1;HRAS |
| **Arachidonic acid metabolism** | 1/61 | 8.7248996E-01 | 0.955665727 | 0.49 | 0.07 | EPHX2 |
| **Gastric cancer** | 3/149 | 8.7537722E-01 | 0.955665727 | 0.60 | 0.08 | CCND1;PIK3R1;HRAS |

| | | | | | | |
|---|---|---|---|---|---|---|
| **Cytosolic DNA-sensing pathway** | 1/63 | 8.8082884E-01 | 0.955665727 | 0.47 | 0.06 | NFKBIA |
| **Cortisol synthesis and secretion** | 1/65 | 8.8862314E-01 | 0.955665727 | 0.45 | 0.05 | GNA11 |
| **Inflammatory bowel disease** | 1/65 | 8.8862314E-01 | 0.955665727 | 0.45 | 0.05 | STAT3 |
| **TNF signaling pathway** | 2/112 | 8.8973951E-01 | 0.955665727 | 0.53 | 0.06 | NFKBIA;PIK3R1 |
| **Serotonergic synapse** | 2/113 | 8.9265480E-01 | 0.955665727 | 0.52 | 0.06 | GNG5;HRAS |
| **Retinol metabolism** | 1/68 | 8.9937054E-01 | 0.95909437 | 0.43 | 0.05 | RDH11 |
| **RIG-I-like receptor signaling pathway** | 1/70 | 9.0595374E-01 | 0.962355527 | 0.42 | 0.04 | NFKBIA |
| **Inositol phosphate metabolism** | 1/73 | 9.1503098E-01 | 0.96823045 | 0.40 | 0.04 | TPI1 |
| **Systemic lupus erythematosus** | 2/135 | 9.4116696E-01 | 0.983645047 | 0.44 | 0.03 | SNRPD1;ACTN4 |
| **MicroRNAs in cancer** | 6/310 | 9.4686138E-01 | 0.983645047 | 0.57 | 0.03 | UBE2I;CCND1;STAT3;PIK3R1;EZR;HRAS |
| **Bile secretion** | 1/90 | 9.5220922E-01 | 0.983645047 | 0.33 | 0.02 | ATP1B1 |
| **Morphine addiction** | 1/91 | 9.5380058E-01 | 0.983645047 | 0.32 | 0.02 | GNG5 |
| **Th1 and Th2 cell differentiation** | 1/92 | 9.5533902E-01 | 0.983645047 | 0.32 | 0.01 | NFKBIA |
| **Transcriptional misregulation in cancer** | 3/192 | 9.5568508E-01 | 0.983645047 | 0.46 | 0.02 | DDX5;SPINT1;DDIT3 |
| **Staphylococcus aureus infection** | 1/95 | 9.5965412E-01 | 0.983645047 | 0.31 | 0.01 | KRT10 |
| **Glycerophospholipid metabolism** | 1/98 | 9.6355285E-01 | 0.983645047 | 0.30 | 0.01 | AGPAT3 |
| **Hematopoietic cell lineage** | 1/99 | 9.6476695E-01 | 0.983645047 | 0.30 | 0.01 | CD24 |
| **Cushing syndrome** | 2/155 | 9.6650223E-01 | 0.983645047 | 0.38 | 0.01 | CCND1;GNA11 |
| **Protein digestion and absorption** | 1/103 | 9.6923267E-01 | 0.983645047 | 0.28 | 0.01 | ATP1B1 |
| **Glutamatergic synapse** | 1/114 | 9.7880831E-01 | 0.989683958 | 0.26 | 0.01 | GNG5 |
| **Cytokine-cytokine receptor interaction** | 1/295 | 9.9995223E-01 | 0.9999912 | 0.10 | 0.00 | TNFRSF21 |

| | | | | | | |
|---|---|---|---|---|---|---|
| **Neuroactive ligand-receptor interaction** | 1/341 | 9.9998734E-01 | 0.9999912 | 0.08 | 0.00 | PTH1R |
| **Olfactory transduction** | 2/440 | 9.9999120E-01 | 0.9999912 | 0.13 | 0.00 | CALM3;CALM1 |

**Supplementary Table S7. Gene signature for JAK-STAT comprising 17 genes**

| Gene Symbol | Entrez Gene ID |
|---|---|
| APOL1 | 8542 |
| APOL6 | 80830 |
| CCDC68 | 80323 |
| CCL2 | 6347 |
| CTSS | 1520 |
| CXCL9 | 4283 |
| CXCL10 | 3627 |
| GBP1 | 2633 |
| GBP2 | 2634 |
| ICAM1 | 3383 |
| IDO1 | 3620 |
| IRF1 | 3659 |
| ITK | 3702 |
| PDCD1LG2 | 80380 |
| PSMB9 | 5698 |
| TMEM140 | 55281 |
| TRIM21 | 6737 |

A STAT1-dependent signature was derived from the 20 most prominently up-regulated STAT1 direct target genes in response to IFN-gamma (From.Gene Regul Syst Bio. 2013 Mar 26:7:41-56. doi: 10.4137/GRSB.S11433. Print 2013.

A Comprehensive Profile of ChIP-Seq-Based STAT1 Target Genes Suggests the Complexity of STAT1-Mediated Gene Regulatory Mechanisms, PMID: 23645984). Of the 20 genes, 17 were expressed and carried forward as a JAK-STAT activity score in kidneys (Tao et al., Kidney Int., 2018, PMID: PMID: 30093081; Tao, et al., CJASN, 2020, PMID: 32354727)

**Supplementary Table S8.** TEEN-Labs demographics and clinical characteristics

| Visit | Month 1, N = 64 | Month 6, N = 51 | Year 1, N = 52 | Year 2, N = 46 | Year 3, N = 45 | Year 4, N = 38 | Year 5, N = 35 |
|---|---|---|---|---|---|---|---|
| **SEX** | | | | | | | |
| **Male** | 17 (27%) | 11 (22%) | 10 (19%) | 12 (26%) | 12 (27%) | 9 (24%) | 7 (20%) |
| **Female** | 47 (73%) | 40 (78%) | 42 (81%) | 34 (74%) | 33 (73%) | 29 (76%) | 28 (80%) |
| **Calculated Age** | 16.47 (1.54) | 17.06 (1.46) | 17.43 (1.69) | 18.67 (1.38) | 19.73 (1.62) | 20.58 (1.45) | 21.71 (1.45) |
| **Missing** | 2 | 2 | 1 | 1 | 0 | 0 | 0 |
| **ETHN** | | | | | | | |
| **Hispanic** | 3 (4.7%) | 3 (5.9%) | 3 (5.8%) | 3 (6.5%) | 3 (6.7%) | 3 (7.9%) | 3 (8.6%) |
| **Non-Hispanic** | 61 (95%) | 48 (94%) | 49 (94%) | 43 (93%) | 42 (93%) | 35 (92%) | 32 (91%) |
| **RACE** | | | | | | | |
| **White or Caucasian** | 39 (61%) | 31 (61%) | 33 (63%) | 27 (59%) | 28 (62%) | 22 (58%) | 22 (63%) |
| **Black or African-American** | 20 (31%) | 16 (31%) | 15 (29%) | 16 (35%) | 14 (31%) | 12 (32%) | 10 (29%) |
| **Asian** | 0 (0%) | 0 (0%) | 0 (0%) | 0 (0%) | 0 (0%) | 0 (0%) | 0 (0%) |
| **American Indian or Alaska Native** | 0 (0%) | 0 (0%) | 0 (0%) | 0 (0%) | 0 (0%) | 0 (0%) | 0 (0%) |
| **Native Hawaiian or other Pacific Islander** | 0 (0%) | 0 (0%) | 0 (0%) | 0 (0%) | 0 (0%) | 0 (0%) | 0 (0%) |
| **Other** | 0 (0%) | 0 (0%) | 0 (0%) | 0 (0%) | 0 (0%) | 0 (0%) | 0 (0%) |
| **Unknown** | 0 (0%) | 0 (0%) | 0 (0%) | 0 (0%) | 0 (0%) | 0 (0%) | 0 (0%) |
| **More than one race** | 5 (7.8%) | 4 (7.8%) | 4 (7.7%) | 3 (6.5%) | 3 (6.7%) | 4 (11%) | 3 (8.6%) |
| **SURG** | | | | | | | |
| **Gastric bypass** | 47 (73%) | 39 (76%) | 36 (69%) | 34 (74%) | 34 (76%) | 29 (76%) | 28 (80%) |
| **Laparoscopic adjustable gastric band** | 3 (4.7%) | 1 (2.0%) | 2 (3.8%) | 2 (4.3%) | 2 (4.4%) | 1 (2.6%) | 2 (5.7%) |
| **Sleeve gastrectomy - initial stage** | 14 (22%) | 11 (22%) | 14 (27%) | 10 (22%) | 9 (20%) | 8 (21%) | 5 (14%) |
| **Diabetes** | 23 (37%) | 6 (13%) | 2 (4.0%) | 2 (4.7%) | 1 (2.3%) | 2 (5.3%) | 2 (5.7%) |
| **Missing** | 2 | 3 | 2 | 3 | 1 | 0 | 0 |
| **Diabetes resolved?** | | | | | | | |
| **No** | 2 (3.1%) | 2 (3.9%) | 2 (3.8%) | 2 (4.3%) | 2 (4.4%) | 2 (5.3%) | 2 (5.7%) |
| **Yes** | 21 (33%) | 18 (35%) | 17 (33%) | 16 (35%) | 16 (36%) | 13 (34%) | 11 (31%) |
| **Non-diabetic** | 41 (64%) | 31 (61%) | 33 (63%) | 28 (61%) | 27 (60%) | 23 (61%) | 22 (63%) |
| **Diabetes duration (yrs)** | 3.27 (2.85) | NA (NA) | NA (NA) | NA (NA) | NA (NA) | NA (NA) | NA (NA) |
| **Missing** | 47 | 51 | 52 | 46 | 45 | 38 | 35 |
| **Systolic blood pressure** | 127 (14) | 120 (14) | 117 (13) | 120 (14) | 119 (16) | 122 (15) | 124 (14) |
| **Missing** | 2 | 2 | 2 | 2 | 1 | 0 | 0 |
| **Systolic blood pressure percentile** | 80 (24) | 63 (30) | 58 (28) | 64 (30) | 62 (29) | 57 (31) | 99 (NA) |
| **Missing** | 2 | 4 | 6 | 16 | 27 | 27 | 34 |
| **Diastolic blood pressure** | 76 (10) | 71 (10) | 70 (9) | 72 (9) | 73 (10) | 74 (10) | 75 (11) |
| **Missing** | 2 | 2 | 2 | 2 | 1 | 0 | 0 |
| **Diastolic blood pressure percentile** | 73 (23) | 61 (26) | 59 (23) | 63 (26) | 62 (20) | 68 (20) | 72 (NA) |
| **Missing** | 2 | 4 | 6 | 16 | 27 | 27 | 34 |
| **map** | 93 (10) | 87 (11) | 86 (9) | 88 (10) | 89 (11) | 90 (11) | 91 (11) |
| **Missing** | 2 | 2 | 2 | 2 | 1 | 0 | 0 |
| **Heart rate** | 83 (13) | 74 (12) | 73 (12) | 75 (13) | 74 (11) | 77 (15) | 79 (15) |
| **Missing** | 4 | 3 | 6 | 4 | 2 | 3 | 1 |
| **Body fat %** | 54 (7) | 44 (8) | 40 (10) | 41 (11) | 43 (11) | 44 (10) | 43 (9) |
| **Missing** | 5 | 5 | 7 | 3 | 0 | 1 | 1 |
| **BMI** | 54 (10) | 40 (8) | 39 (10) | 39 (11) | 42 (12) | 42 (12) | 40 (10) |
| **Missing** | 2 | 3 | 2 | 2 | 0 | 0 | 0 |
| **Waist circumference (Iliac waist circumference )** | 150 (17) | 125 (17) | 121 (20) | 121 (22) | 125 (22) | 127 (25) | 122 (22) |
| **Missing** | 2 | 3 | 3 | 3 | 1 | 0 | 0 |
| **Umbilical Waist Circumference** | 142 (16) | 115 (18) | 112 (19) | 113 (24) | 116 (23) | 116 (23) | 109 (20) |
| **Missing** | 2 | 3 | 3 | 3 | 1 | 0 | 0 |
| **Neck circumference** | 44.1 (4.6) | 38.5 (3.7) | 37.5 (3.7) | 37.8 (4.7) | 38.4 (4.9) | 38.6 (4.8) | 37.4 (4.3) |
| **Missing** | 2 | 3 | 3 | 2 | 1 | 0 | 0 |
| **Sagittal Abdominal Diameter** | 32.5 (4.5) | 25.6 (4.4) | 24.8 (4.9) | 24.7 (5.5) | 26.7 (6.1) | 26.9 (6.2) | 25.3 (4.8) |

| | | | | | | | |
|---|---|---|---|---|---|---|---|
| **Missing** | 2 | 4 | 5 | 3 | 1 | 0 | 0 |
| **HbA1c** | 5.75 (1.32) | 5.13 (0.99) | 5.19 (0.95) | 5.24 (0.95) | 5.29 (1.09) | 5.39 (1.35) | 5.53 (1.59) |
| **Missing** | 2 | 4 | 2 | 2 | 0 | 0 | 0 |
| **HDL Cholesterol** | 40 (9) | 45 (11) | 49 (10) | 52 (13) | 54 (15) | 55 (16) | 56 (16) |
| **Missing** | 2 | 3 | 1 | 2 | 0 | 0 | 0 |
| **LDL Cholesterol - Derived** | 96 (27) | 87 (21) | 87 (26) | 87 (23) | 83 (24) | 88 (28) | 81 (21) |
| **Missing** | 2 | 3 | 1 | 2 | 0 | 0 | 0 |
| **TRIGLYCERIDE** | 128 (62) | 91 (33) | 85 (47) | 87 (54) | 82 (53) | 84 (44) | 84 (50) |
| **Missing** | 2 | 3 | 1 | 2 | 0 | 0 | 0 |
| **Insulin (Tosoh)** | 56 (164) | 21 (21) | 14 (12) | 15 (15) | 14 (13) | 15 (12) | 11 (7) |
| **Missing** | 2 | 4 | 1 | 2 | 0 | 0 | 0 |
| **Glucose** | 102 (34) | 88 (23) | 84 (16) | 88 (11) | 87 (12) | 90 (37) | 95 (45) |
| **Missing** | 2 | 3 | 1 | 2 | 0 | 0 | 0 |
| **Weight** | 155 (33) | 114 (27) | 110 (31) | 112 (33) | 119 (38) | 121 (37) | 113 (28) |
| **Missing** | 2 | 3 | 1 | 1 | 0 | 0 | 0 |
| **Height** | 169 (9) | 168 (9) | 168 (8) | 169 (10) | 169 (8) | 170 (9) | 169 (8) |
| **Missing** | 2 | 3 | 2 | 2 | 0 | 0 | 0 |
| **UACR (mg/g)** | 42 (116) | 14 (20) | 33 (128) | 25 (46) | 17 (36) | 21 (49) | 32 (78) |
| **Missing** | 2 | 6 | 6 | 5 | 3 | 0 | 0 |
| **Albuminuria level** | | | | | | | |
| **A1** | 52 (85%) | 38 (86%) | 40 (89%) | 33 (83%) | 36 (92%) | 30 (83%) | 29 (88%) |
| **A2** | 7 (11%) | 6 (14%) | 4 (8.9%) | 7 (18%) | 3 (7.7%) | 6 (17%) | 3 (9.1%) |
| **A3** | 2 (3.3%) | 0 (0%) | 1 (2.2%) | 0 (0%) | 0 (0%) | 0 (0%) | 1 (3.0%) |
| **Missing** | 3 | 7 | 7 | 6 | 6 | 2 | 2 |
| **Cystatin C** | 0.80 (0.15) | 0.73 (0.14) | 0.74 (0.13) | 0.72 (0.13) | 0.73 (0.15) | 0.76 (0.16) | 0.75 (0.13) |
| **Missing** | 2 | 3 | 1 | 2 | 0 | 0 | 0 |
| **Serum Creatinine** | 0.69 (0.13) | 0.65 (0.10) | 0.74 (0.53) | 0.69 (0.16) | 0.67 (0.10) | 0.69 (0.13) | 0.69 (0.14) |
| **Missing** | 2 | 3 | 1 | 2 | 0 | 0 | 0 |
| **eGFR FAS Cr Cys-C** | 112 (17) | 120 (17) | 116 (20) | 121 (19) | 122 (18) | 119 (19) | 119 (18) |
| **Missing** | 2 | 3 | 1 | 2 | 0 | 0 | 0 |

[1] **n (%); Mean (SD)**
