# Supplementary figures and images for: Metabolic surgery mitigates early kidney injury in obese youth with diabetes by suppressing mTORC1/JAK/STAT signaling
Source: J Clin Invest. 2026 Feb 3;136(7):e198545. doi: 10.1172/JCI198545 (PMC13038200; doi:10.1172/JCI198545)

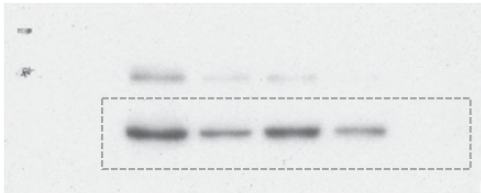

pS6K1

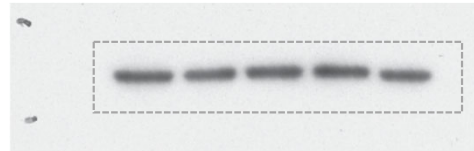

pAkt

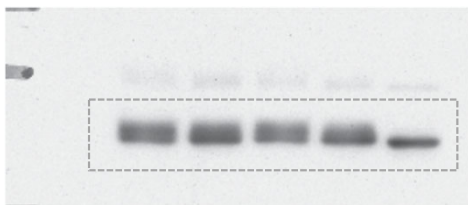

S6K1

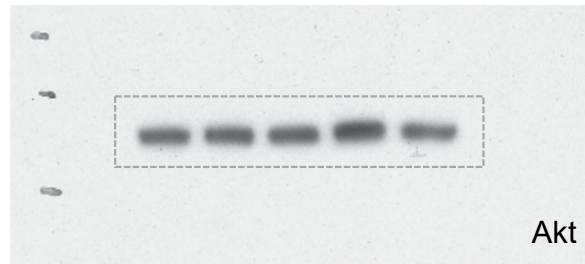

Akt

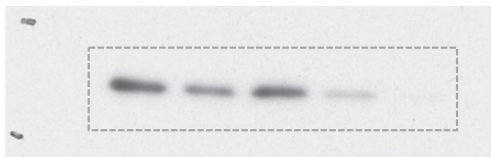

pS6

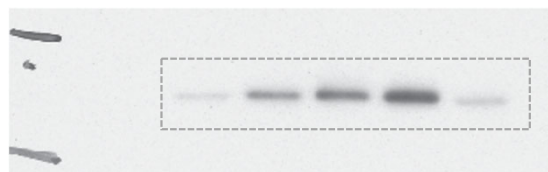

pAMPK

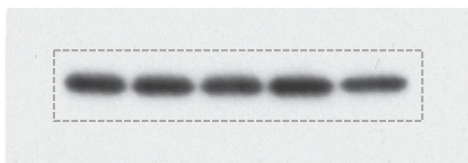

S6

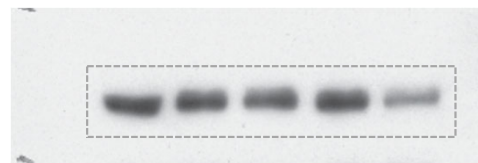

AMPK

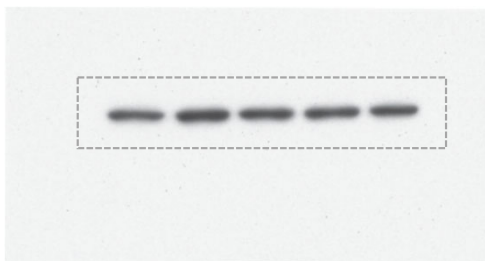

Actin

Supplement: Unedited blot and gel images [file jci-136-198545-s128.pdf]
